# Supplementary material for: Inhibitory proteins block substrate access by occupying the active site cleft of Bacillus subtilis intramembrane protease SpoIVFB
Source: eLife. 2022 Apr 26;11:e74275. doi: 10.7554/eLife.74275 (PMC9042235; doi:10.7554/eLife.74275)
Supplement: Figure 1—source data 1. [file elife-74275-fig1-data1.zip › Figure 1-source data 1/Figure 1B/Fig1B annotated blot.pptx]

## Slide 1
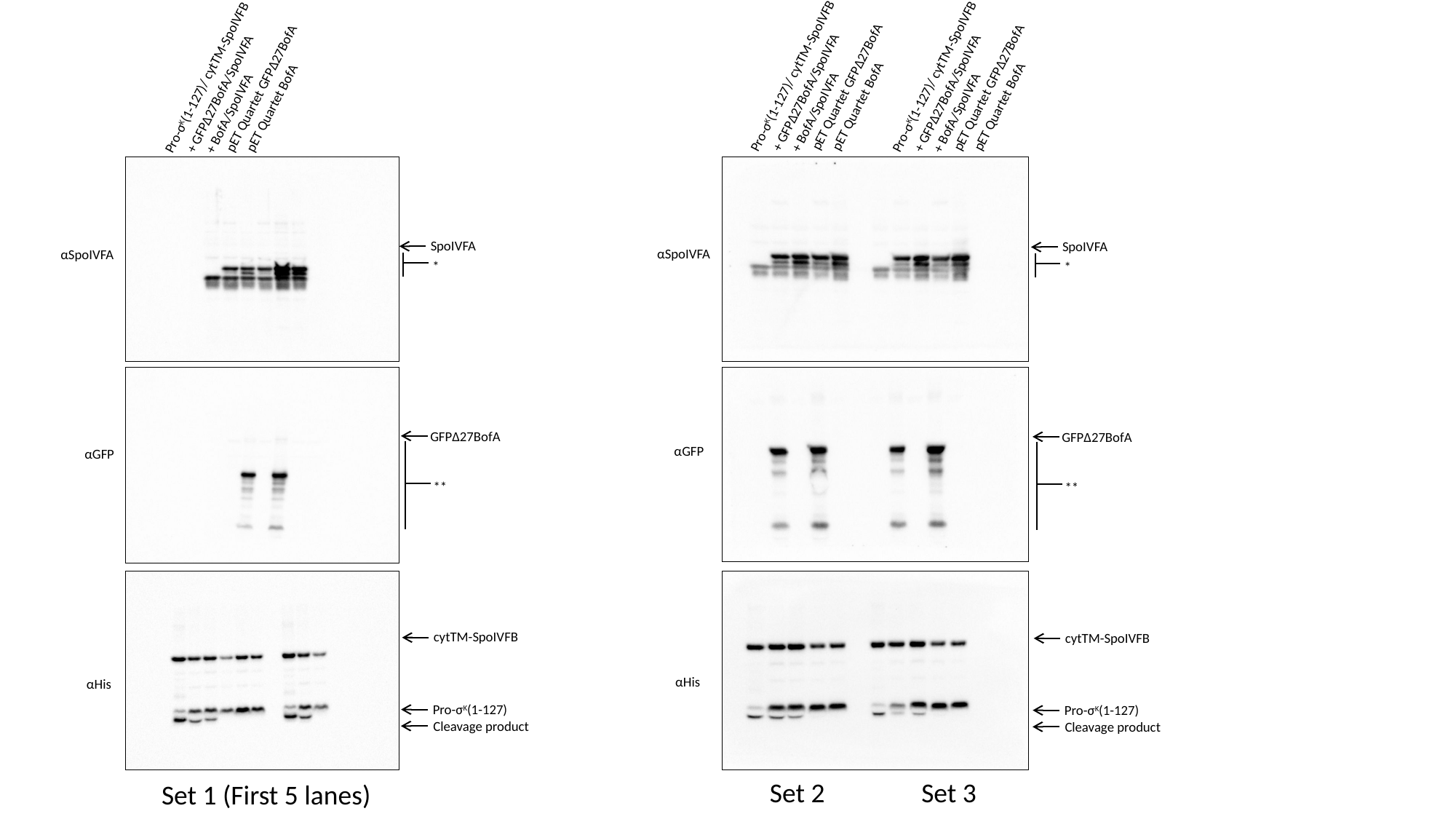

Pro-σK(1-127)/ cytTM-SpoIVFB
Pro-σK(1-127)/ cytTM-SpoIVFB
Pro-σK(1-127)/ cytTM-SpoIVFB
pET Quartet GFPΔ27BofA
pET Quartet GFPΔ27BofA
+ GFPΔ27BofA/SpoIVFA
pET Quartet GFPΔ27BofA
+ GFPΔ27BofA/SpoIVFA
+ GFPΔ27BofA/SpoIVFA
pET Quartet BofA
pET Quartet BofA
pET Quartet BofA
+ BofA/SpoIVFA
+ BofA/SpoIVFA
+ BofA/SpoIVFA
SpoIVFA
SpoIVFA
αSpoIVFA
αSpoIVFA
*
*
GFPΔ27BofA
GFPΔ27BofA
αGFP
αGFP
**
**
cytTM-SpoIVFB
cytTM-SpoIVFB
αHis
αHis
Pro-σK(1-127)
Pro-σK(1-127)
Cleavage product
Cleavage product
Set 2
Set 3
Set 1 (First 5 lanes)

## Slide 2
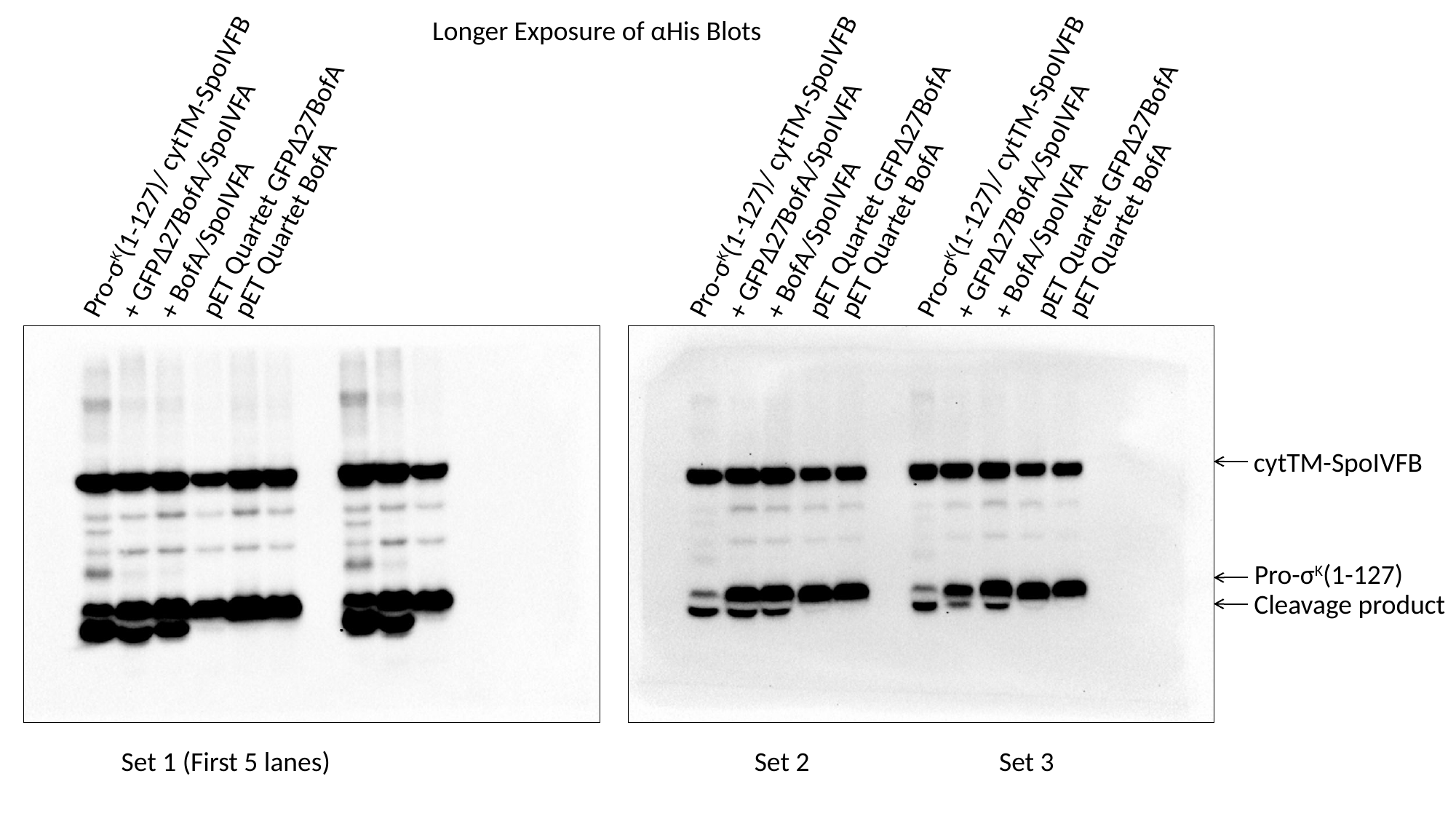

Longer Exposure of αHis Blots
Pro-σK(1-127)/ cytTM-SpoIVFB
Pro-σK(1-127)/ cytTM-SpoIVFB
Pro-σK(1-127)/ cytTM-SpoIVFB
pET Quartet GFPΔ27BofA
pET Quartet GFPΔ27BofA
pET Quartet GFPΔ27BofA
+ GFPΔ27BofA/SpoIVFA
+ GFPΔ27BofA/SpoIVFA
+ GFPΔ27BofA/SpoIVFA
pET Quartet BofA
pET Quartet BofA
pET Quartet BofA
+ BofA/SpoIVFA
+ BofA/SpoIVFA
+ BofA/SpoIVFA
cytTM-SpoIVFB
Pro-σK(1-127)
Cleavage product
Set 1 (First 5 lanes)
Set 2
Set 3
