# Supplementary material for: Inhibitory proteins block substrate access by occupying the active site cleft of Bacillus subtilis intramembrane protease SpoIVFB
Source: eLife. 2022 Apr 26;11:e74275. doi: 10.7554/eLife.74275 (PMC9042235; doi:10.7554/eLife.74275)
Supplement: Figure 1—source data 1. [file elife-74275-fig1-data1.zip › Figure 1-source data 1/Figure 1C/Fig1C annotated blots.pptx]

## Slide 1
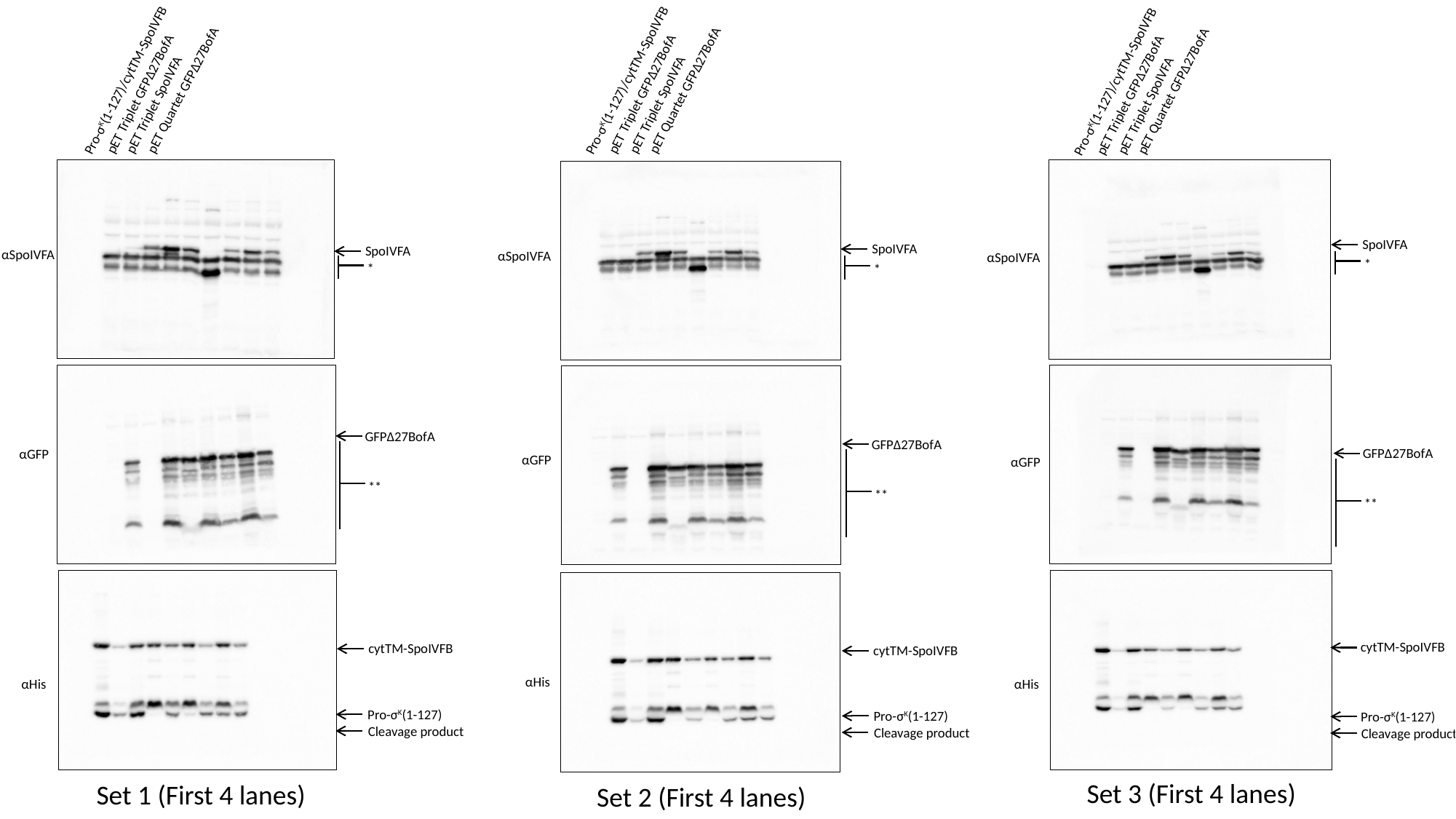

Pro-σK(1-127)/cytTM-SpoIVFB
Pro-σK(1-127)/cytTM-SpoIVFB
Pro-σK(1-127)/cytTM-SpoIVFB
pET Quartet GFPΔ27BofA
pET Quartet GFPΔ27BofA
pET Quartet GFPΔ27BofA
pET Triplet SpoIVFA
pET Triplet SpoIVFA
pET Triplet GFPΔ27BofA
pET Triplet GFPΔ27BofA
pET Triplet SpoIVFA
pET Triplet GFPΔ27BofA
SpoIVFA
SpoIVFA
SpoIVFA
αSpoIVFA
αSpoIVFA
αSpoIVFA
*
*
*
GFPΔ27BofA
GFPΔ27BofA
GFPΔ27BofA
αGFP
αGFP
αGFP
**
**
**
cytTM-SpoIVFB
cytTM-SpoIVFB
cytTM-SpoIVFB
αHis
αHis
αHis
Pro-σK(1-127)
Pro-σK(1-127)
Pro-σK(1-127)
Cleavage product
Cleavage product
Cleavage product
Set 3 (First 4 lanes)
Set 1 (First 4 lanes)
Set 2 (First 4 lanes)
