# Supplementary figures and images for: Inhibitory proteins block substrate access by occupying the active site cleft of Bacillus subtilis intramembrane protease SpoIVFB (part 1 of 3)
Source: eLife. 2022 Apr 26;11:e74275. doi: 10.7554/eLife.74275 (PMC9042235; doi:10.7554/eLife.74275)

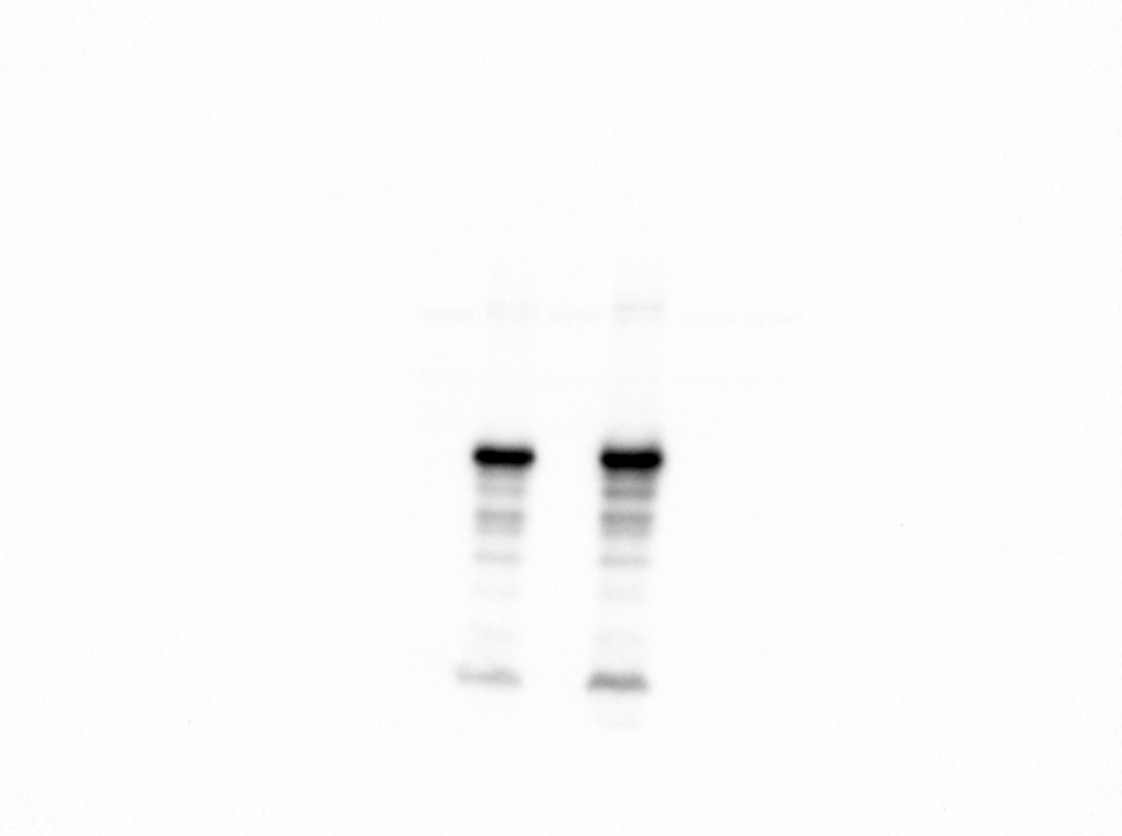

Supplement: Figure 1—source data 1. [file elife-74275-fig1-data1.zip › Figure 1-source data 1/Figure 1B/Fig1B Set1 anti-GFP.tif]

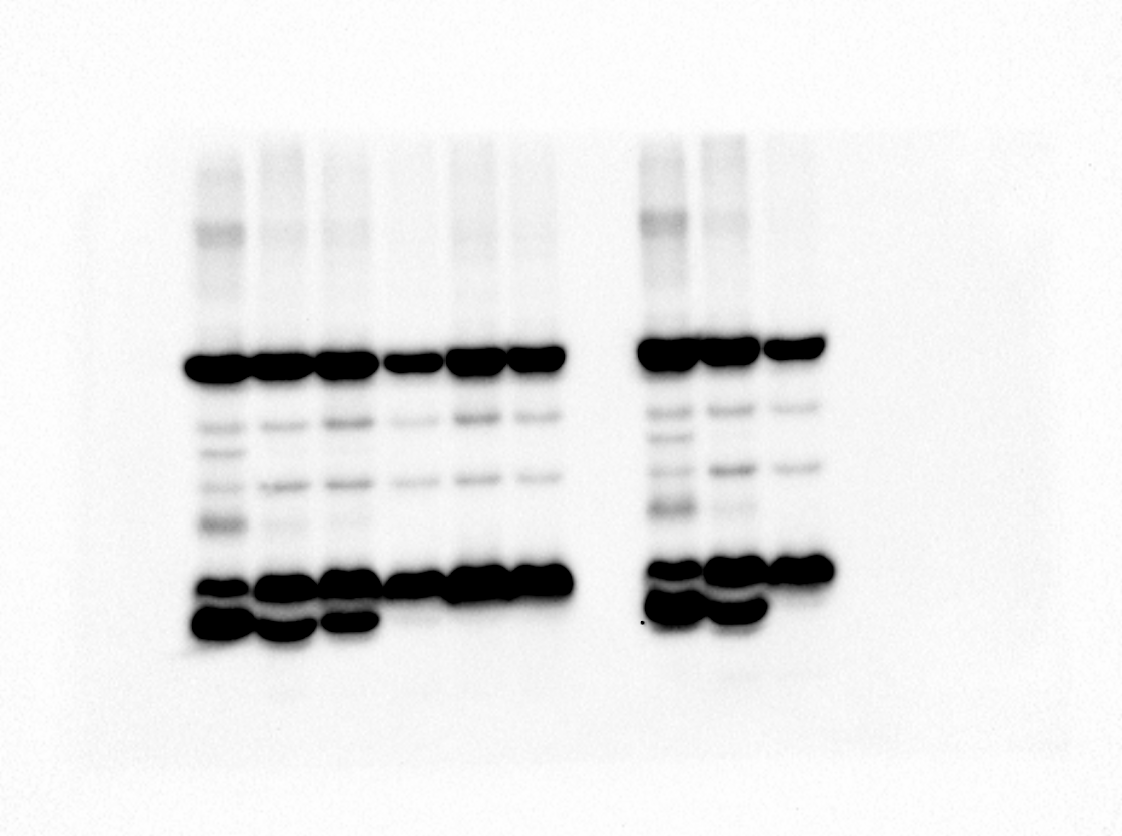

Supplement: Figure 1—source data 1. [file elife-74275-fig1-data1.zip › Figure 1-source data 1/Figure 1B/Fig1B Set1 anti-His long exposure.tif]

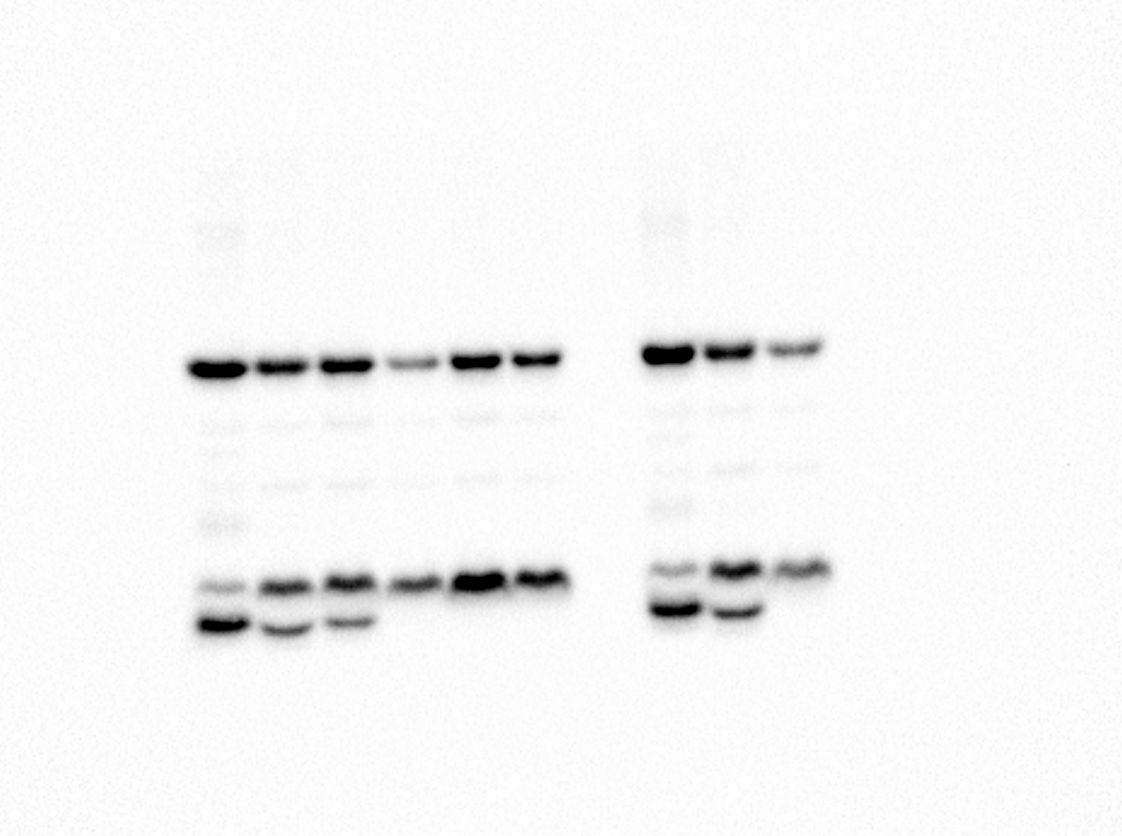

Supplement: Figure 1—source data 1. [file elife-74275-fig1-data1.zip › Figure 1-source data 1/Figure 1B/Fig1B Set1 anti-His.tif]

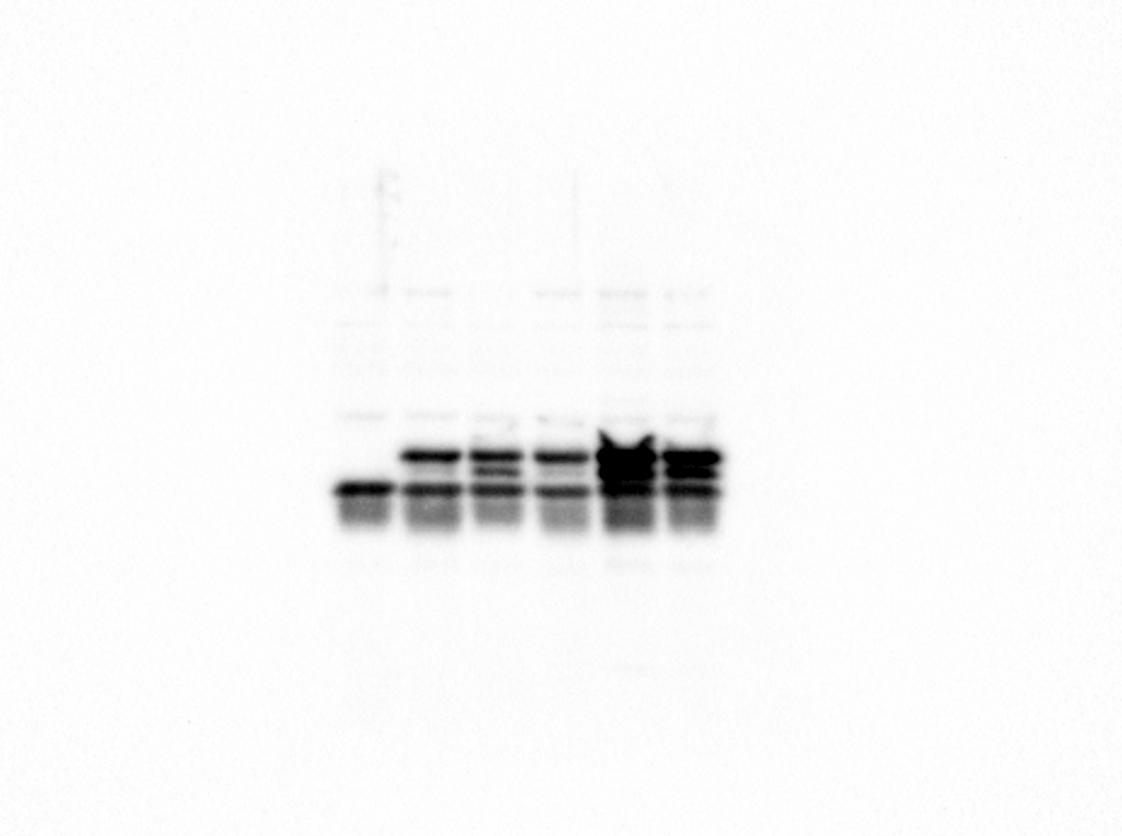

Supplement: Figure 1—source data 1. [file elife-74275-fig1-data1.zip › Figure 1-source data 1/Figure 1B/Fig1B Set1 anti-IVFA.tif]

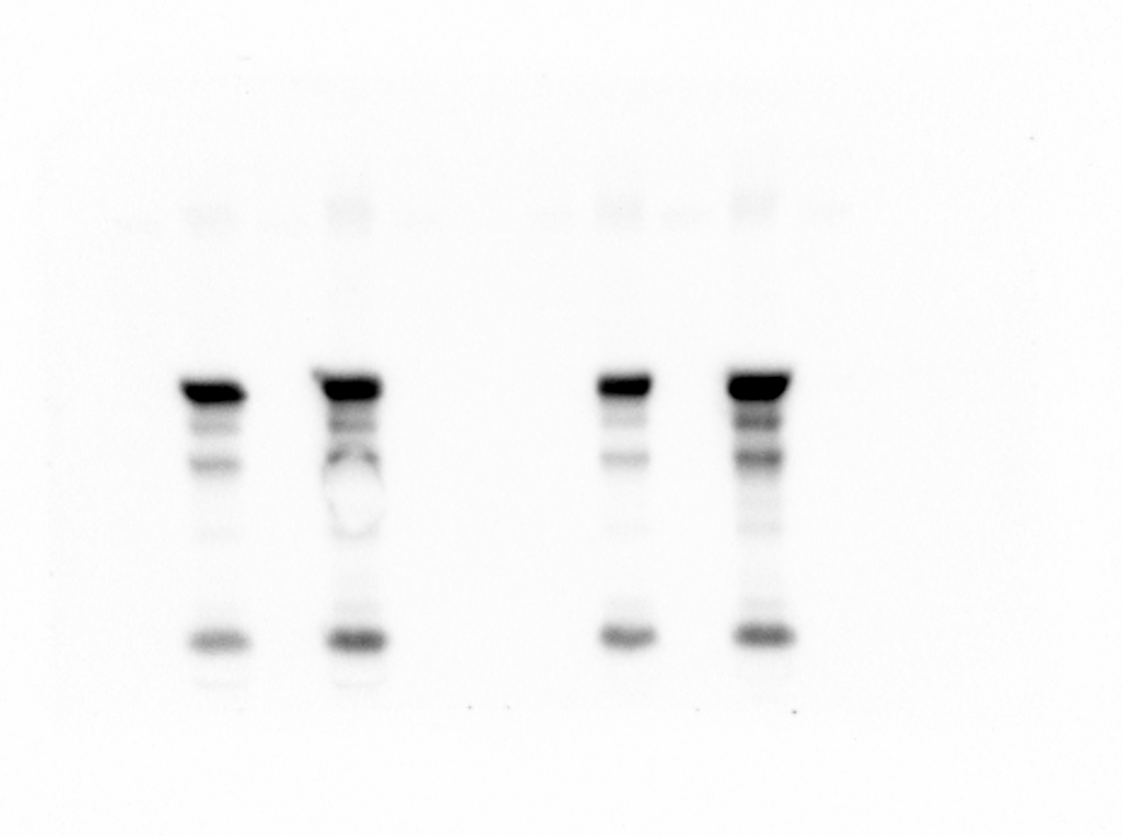

Supplement: Figure 1—source data 1. [file elife-74275-fig1-data1.zip › Figure 1-source data 1/Figure 1B/Fig1B Sets 2 and 3 anti-GFP.tif]

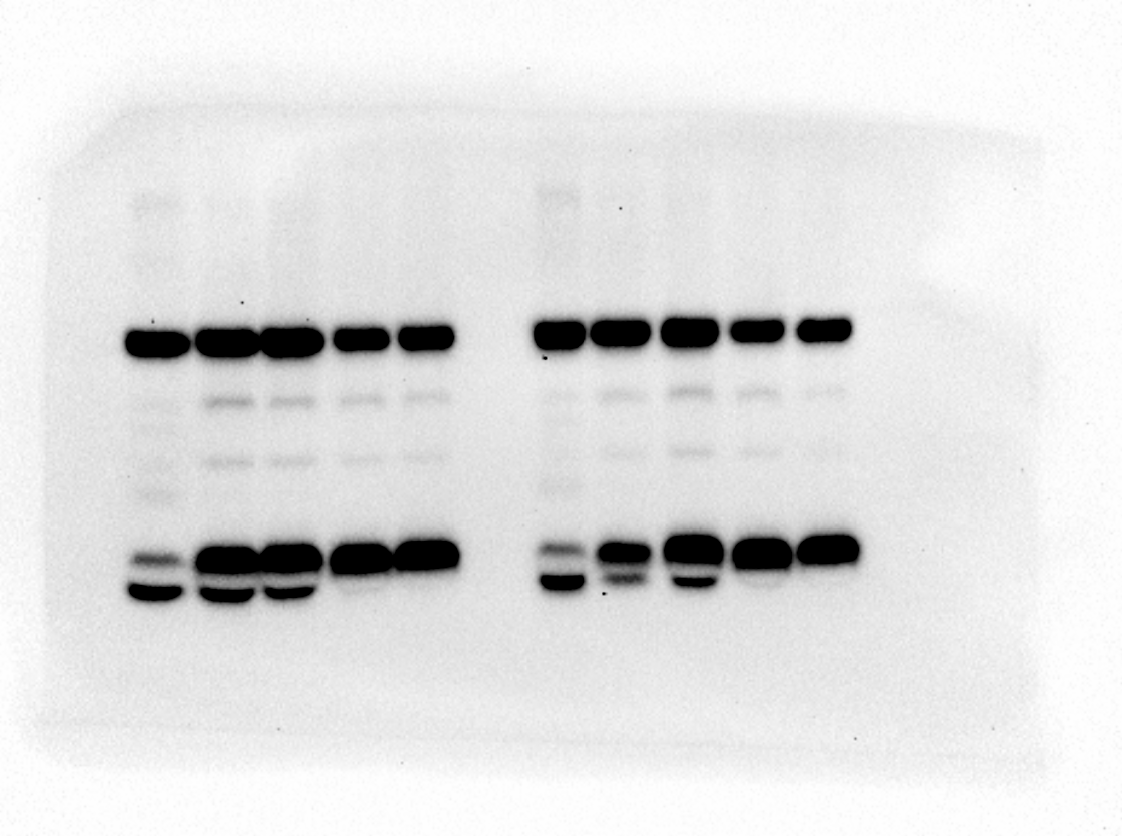

Supplement: Figure 1—source data 1. [file elife-74275-fig1-data1.zip › Figure 1-source data 1/Figure 1B/Fig1B Sets 2 and 3 anti-His long exposure.tif]

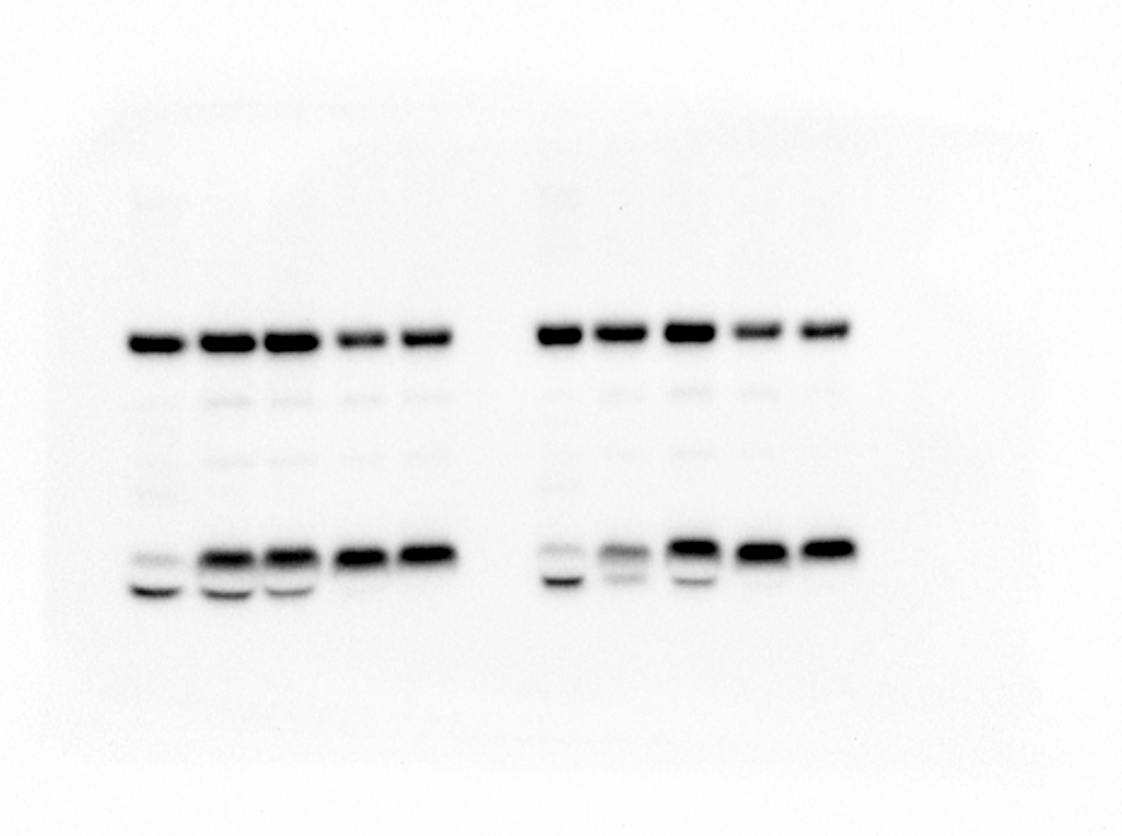

Supplement: Figure 1—source data 1. [file elife-74275-fig1-data1.zip › Figure 1-source data 1/Figure 1B/Fig1B Sets 2 and 3 anti-His.tif]

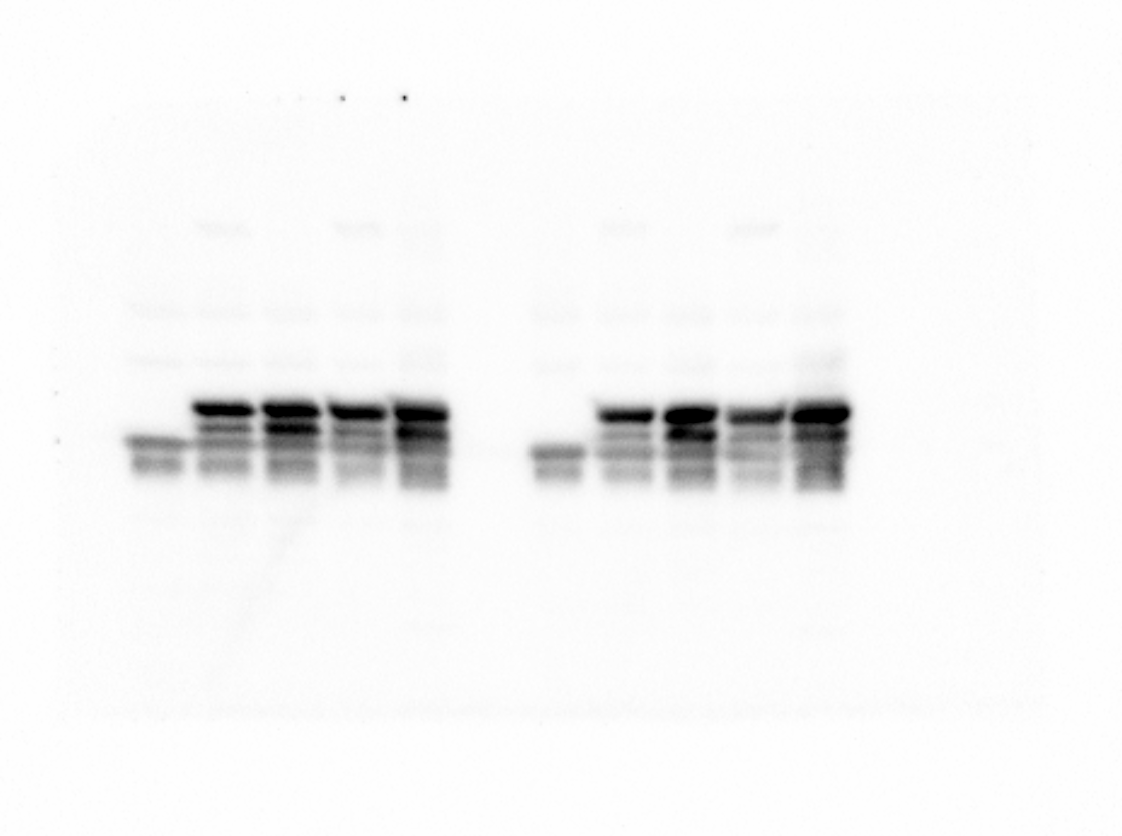

Supplement: Figure 1—source data 1. [file elife-74275-fig1-data1.zip › Figure 1-source data 1/Figure 1B/Fig1B Sets 2 and 3 anti-IVFA.tif]

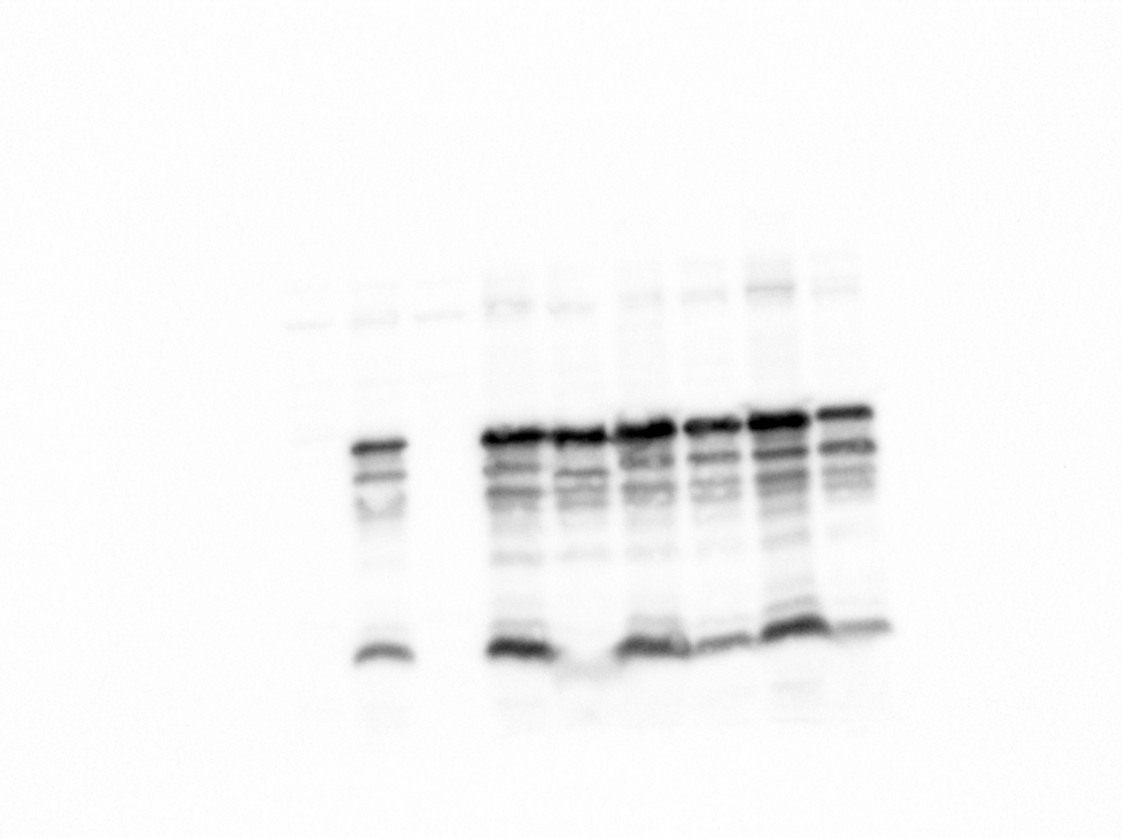

Supplement: Figure 1—source data 1. [file elife-74275-fig1-data1.zip › Figure 1-source data 1/Figure 1C/Fig1C Set1 anti-GFP.tif]

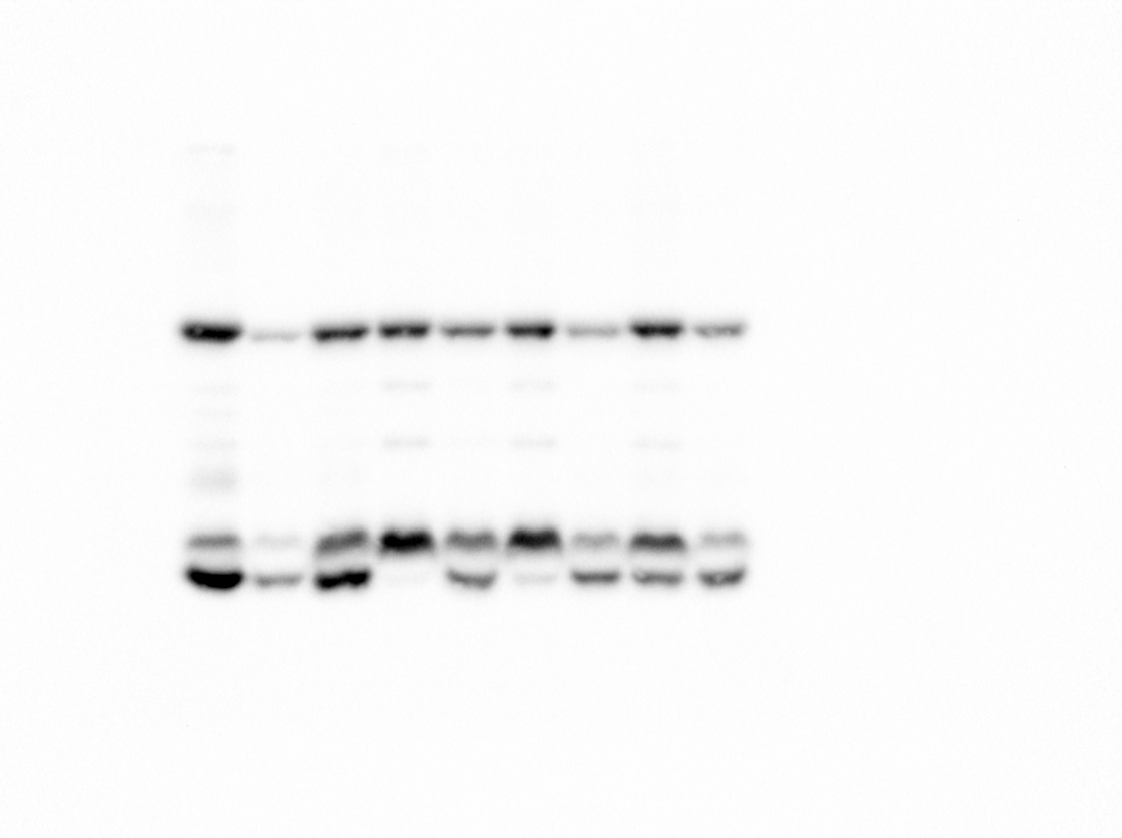

Supplement: Figure 1—source data 1. [file elife-74275-fig1-data1.zip › Figure 1-source data 1/Figure 1C/Fig1C Set1 anti-His.tif]

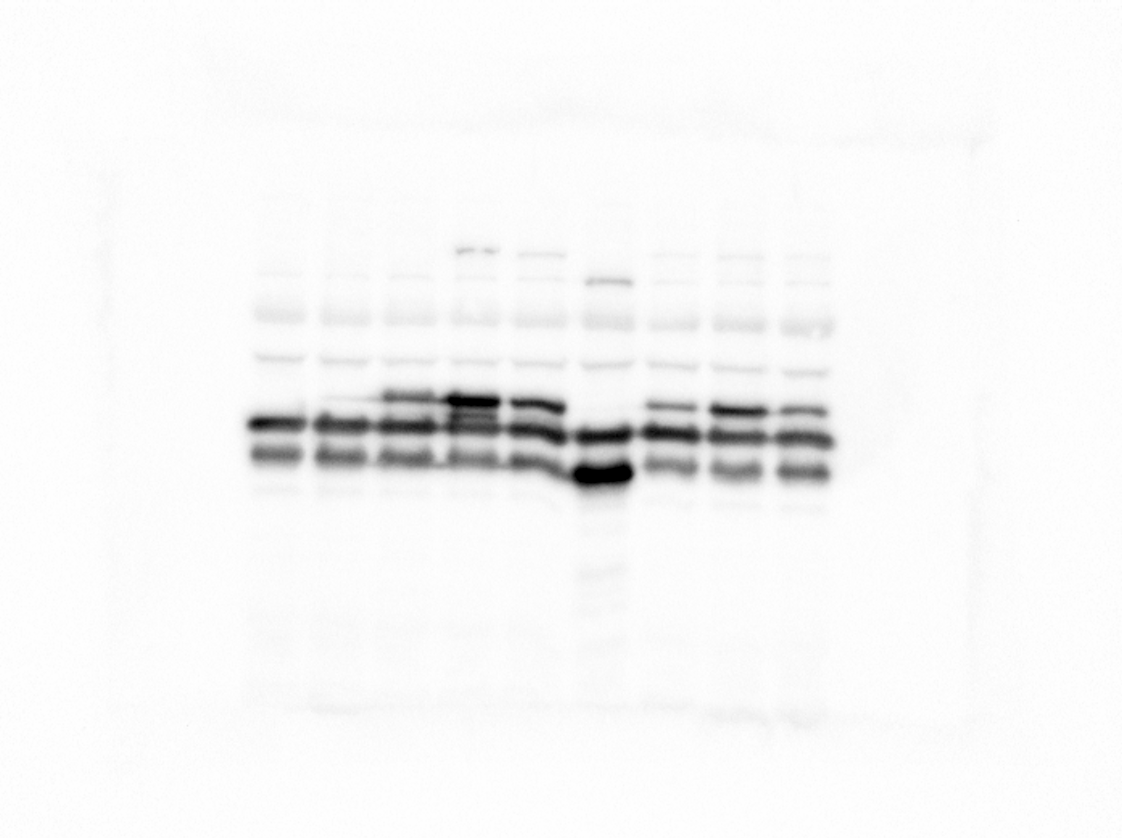

Supplement: Figure 1—source data 1. [file elife-74275-fig1-data1.zip › Figure 1-source data 1/Figure 1C/Fig1C Set1 anti-IVFA.tif]

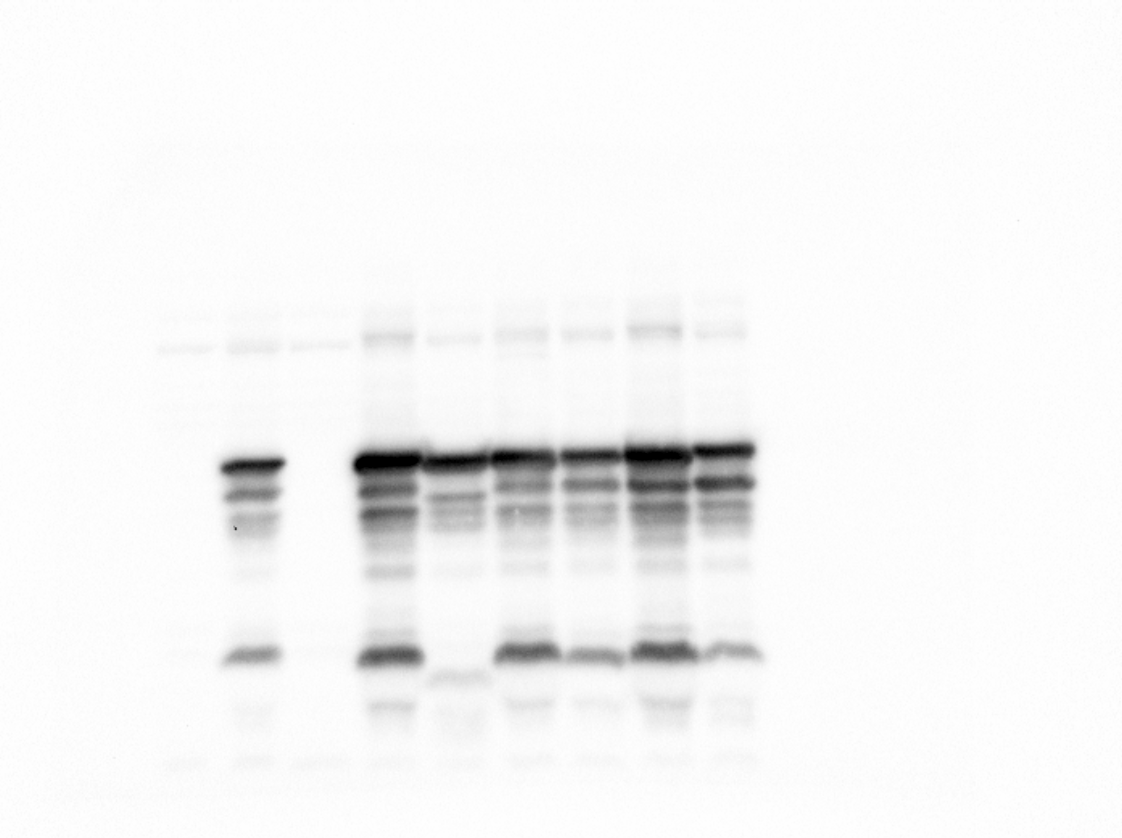

Supplement: Figure 1—source data 1. [file elife-74275-fig1-data1.zip › Figure 1-source data 1/Figure 1C/Fig1C Set2 anti-GFP.tif]

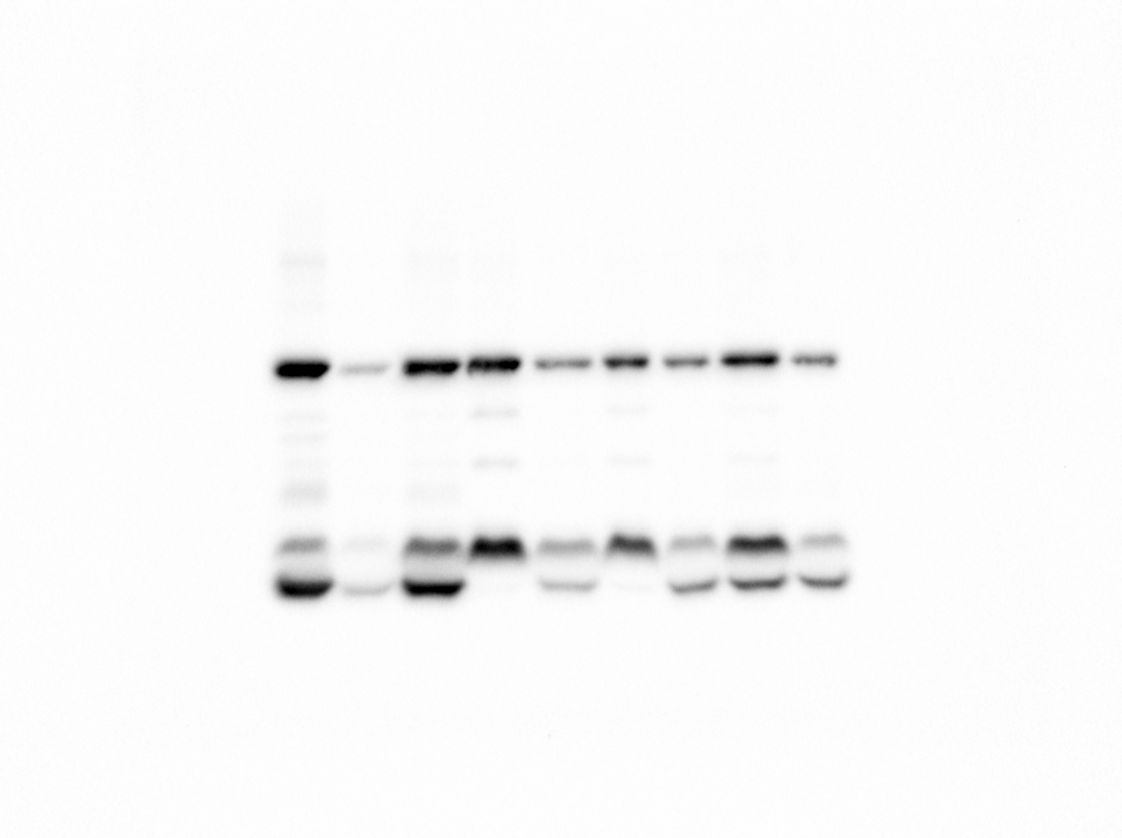

Supplement: Figure 1—source data 1. [file elife-74275-fig1-data1.zip › Figure 1-source data 1/Figure 1C/Fig1C Set2 anti-His.tif]

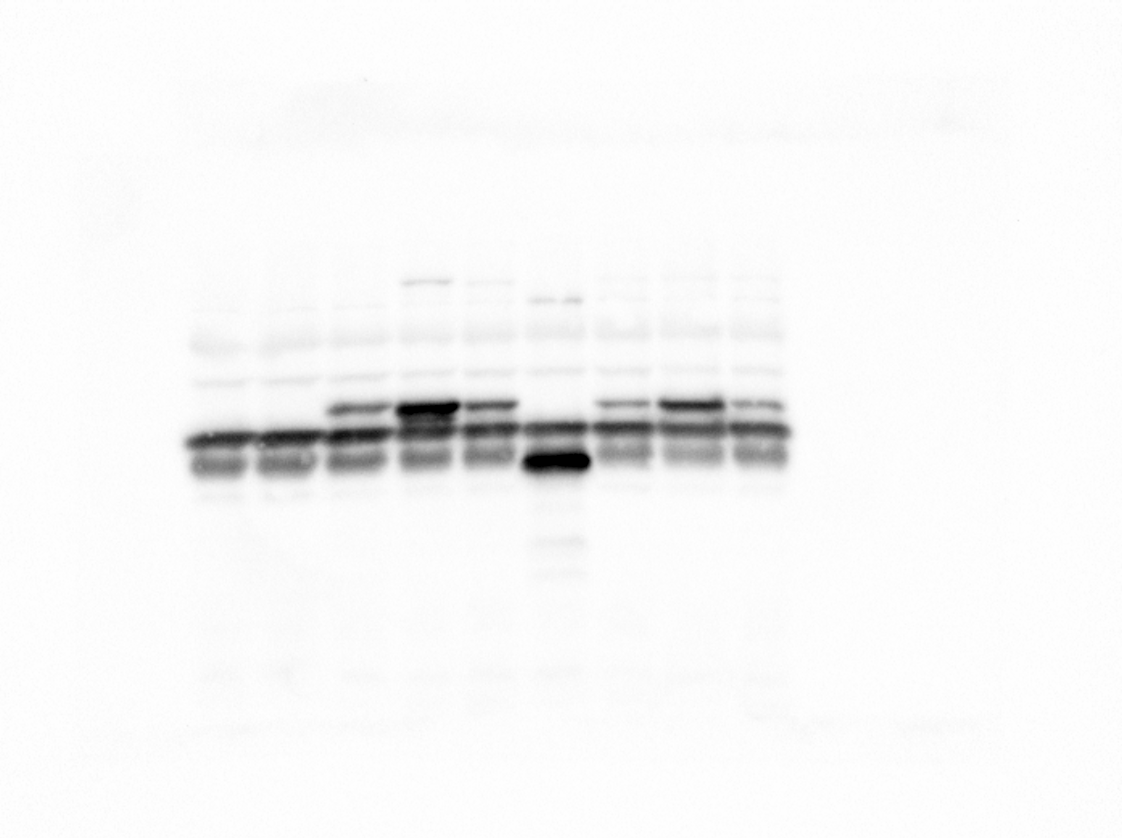

Supplement: Figure 1—source data 1. [file elife-74275-fig1-data1.zip › Figure 1-source data 1/Figure 1C/Fig1C Set2 anti-IVFA.tif]

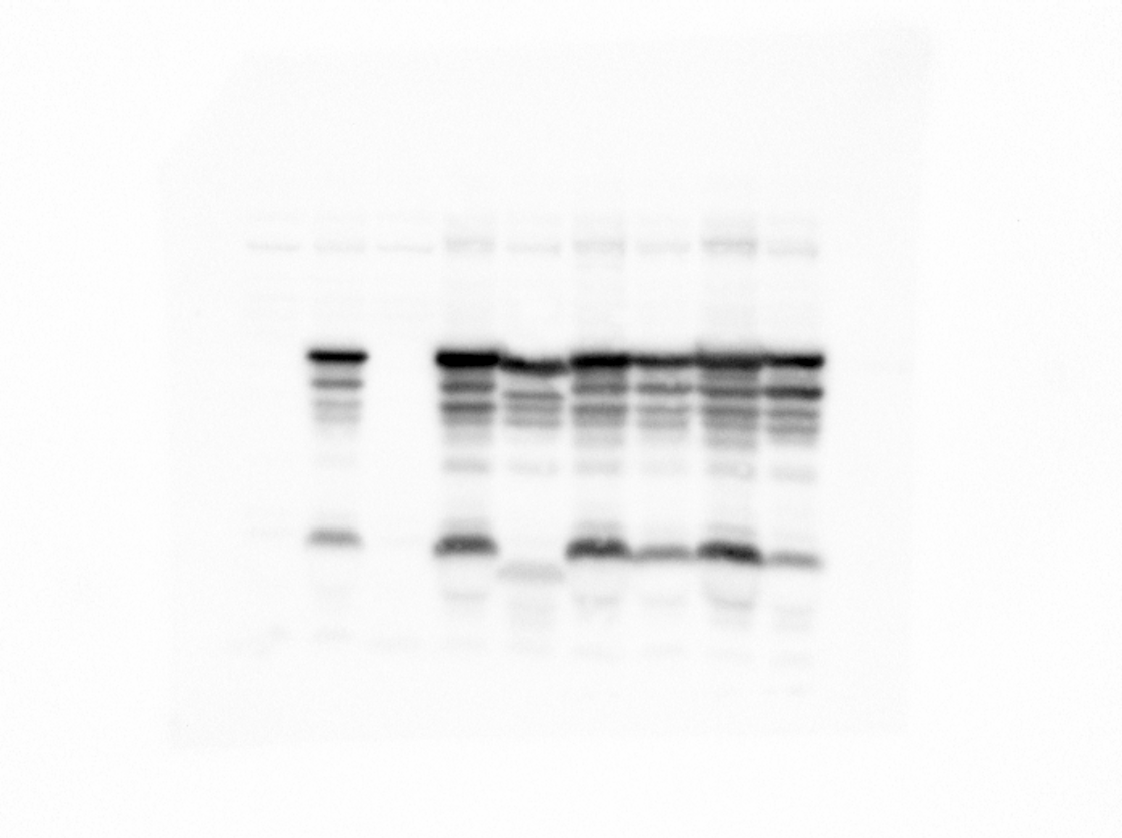

Supplement: Figure 1—source data 1. [file elife-74275-fig1-data1.zip › Figure 1-source data 1/Figure 1C/Fig1C Set3 anti-GFP.tif]

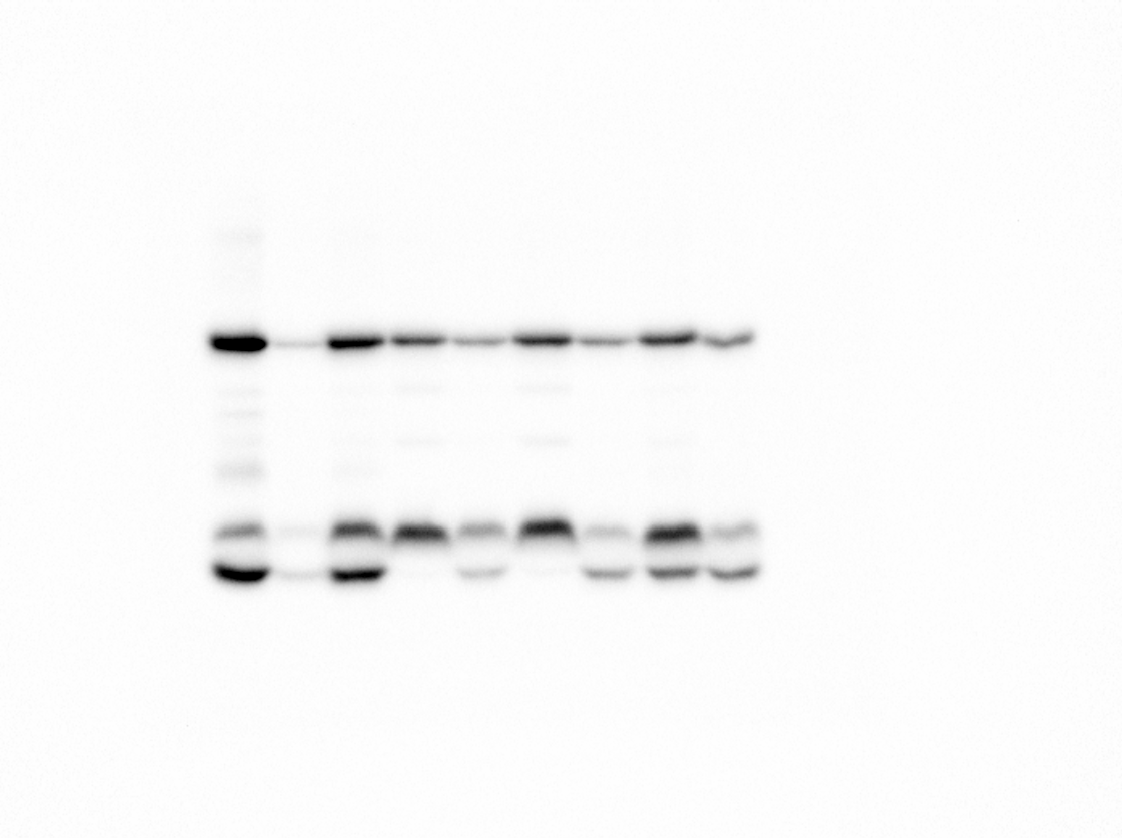

Supplement: Figure 1—source data 1. [file elife-74275-fig1-data1.zip › Figure 1-source data 1/Figure 1C/Fig1C Set3 anti-His.tif]

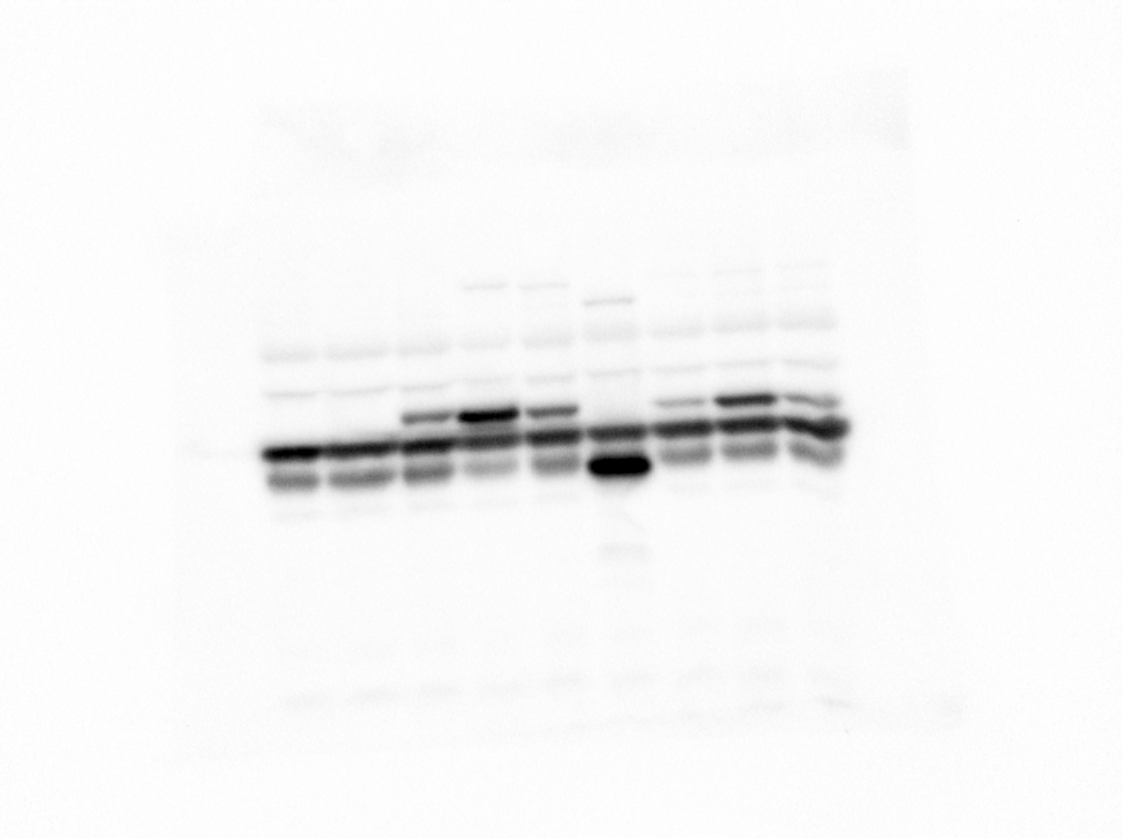

Supplement: Figure 1—source data 1. [file elife-74275-fig1-data1.zip › Figure 1-source data 1/Figure 1C/Fig1C Set3 anti-IVFA.tif]

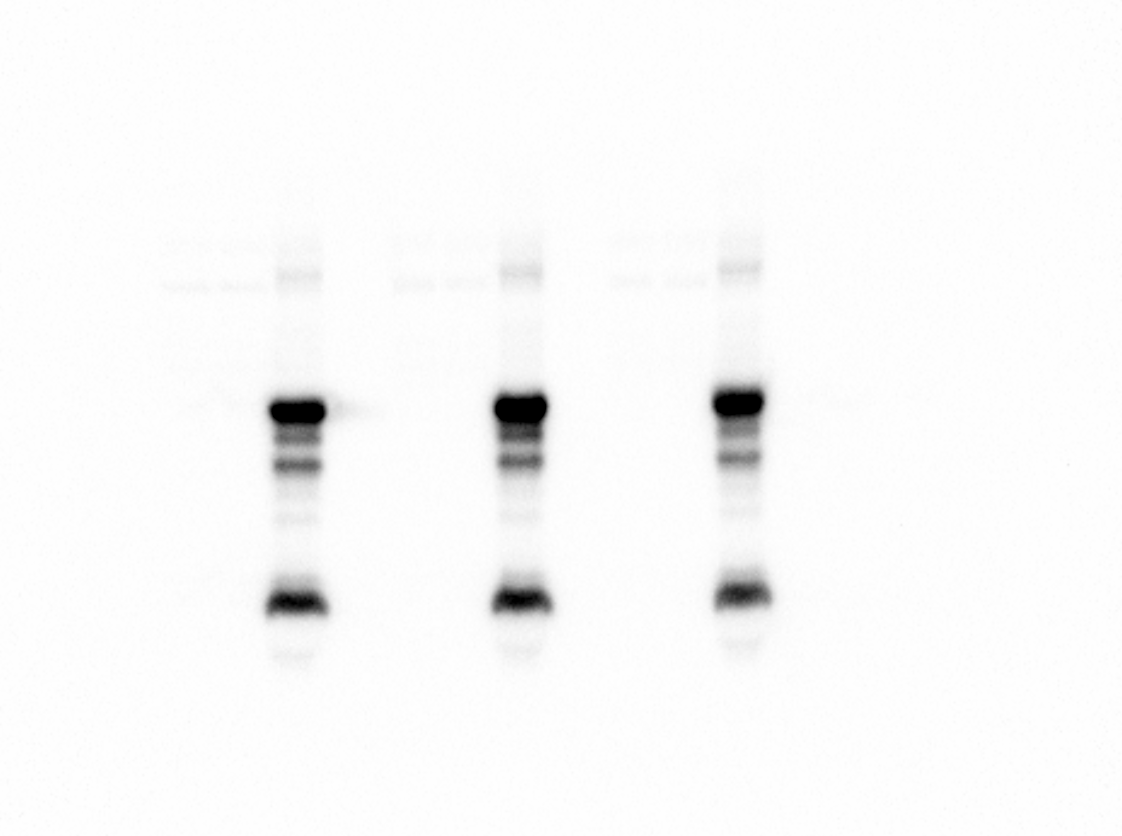

Supplement: Figure 1—figure supplement 2—source data 1. [file elife-74275-fig1-figsupp2-data1.zip › Figure 1-figure supplement 2-source data 1/figure supplement 2A/fig sup 2A anti-GFP.tif]

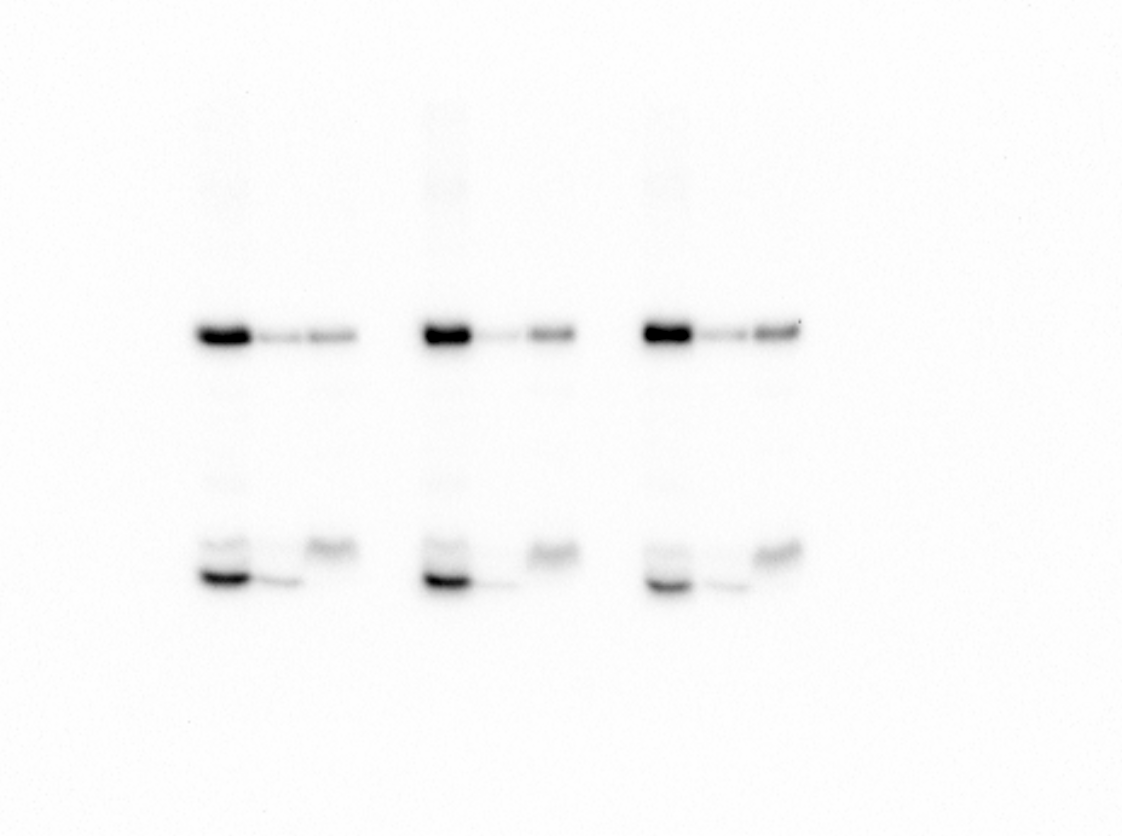

Supplement: Figure 1—figure supplement 2—source data 1. [file elife-74275-fig1-figsupp2-data1.zip › Figure 1-figure supplement 2-source data 1/figure supplement 2A/fig sup 2A anti-His.tif]

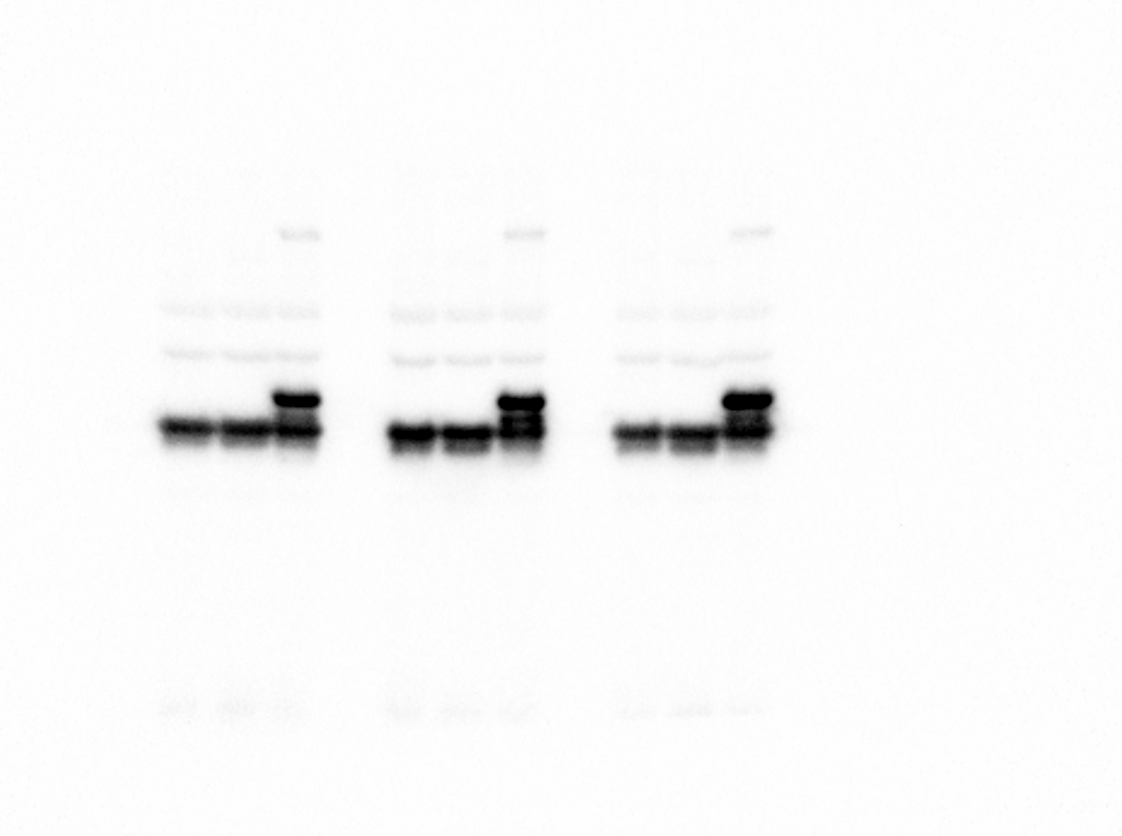

Supplement: Figure 1—figure supplement 2—source data 1. [file elife-74275-fig1-figsupp2-data1.zip › Figure 1-figure supplement 2-source data 1/figure supplement 2A/fig sup 2A anti-IVFA.tif]

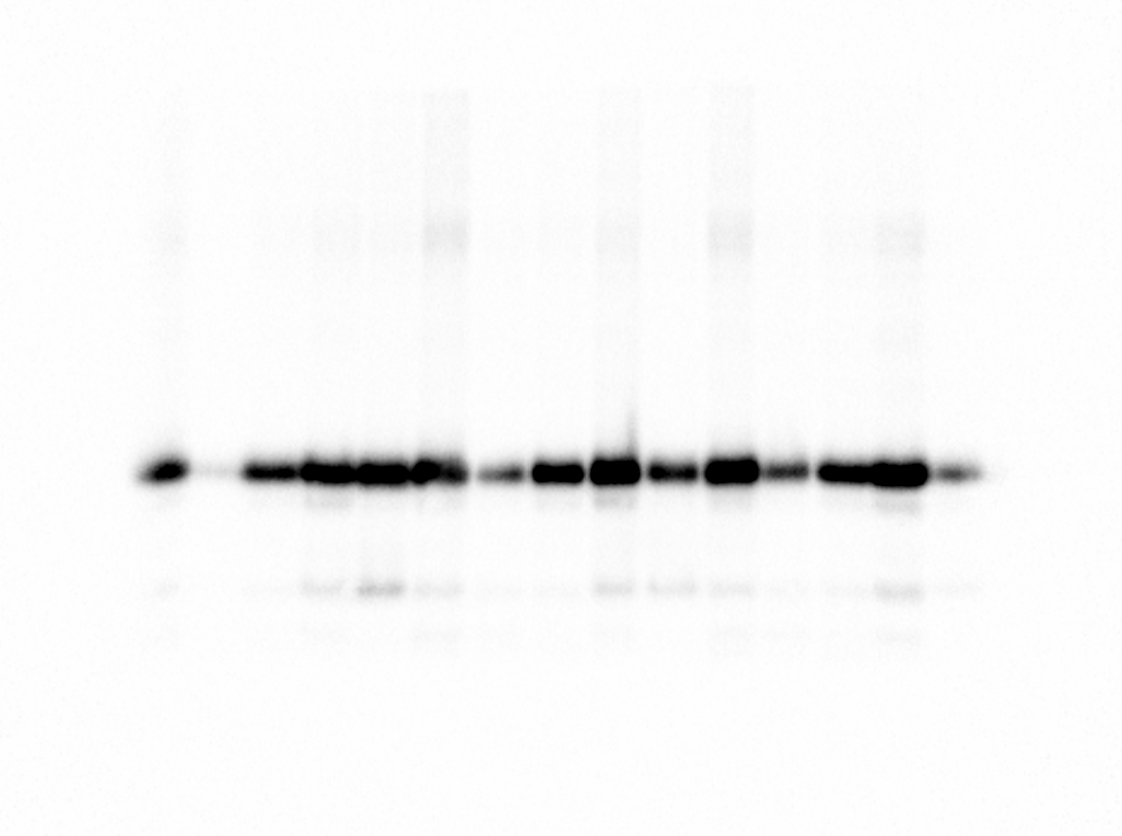

Supplement: Figure 1—figure supplement 2—source data 1. [file elife-74275-fig1-figsupp2-data1.zip › Figure 1-figure supplement 2-source data 1/figure supplement 2B/fig sup 2B anti-FLAG.tif]

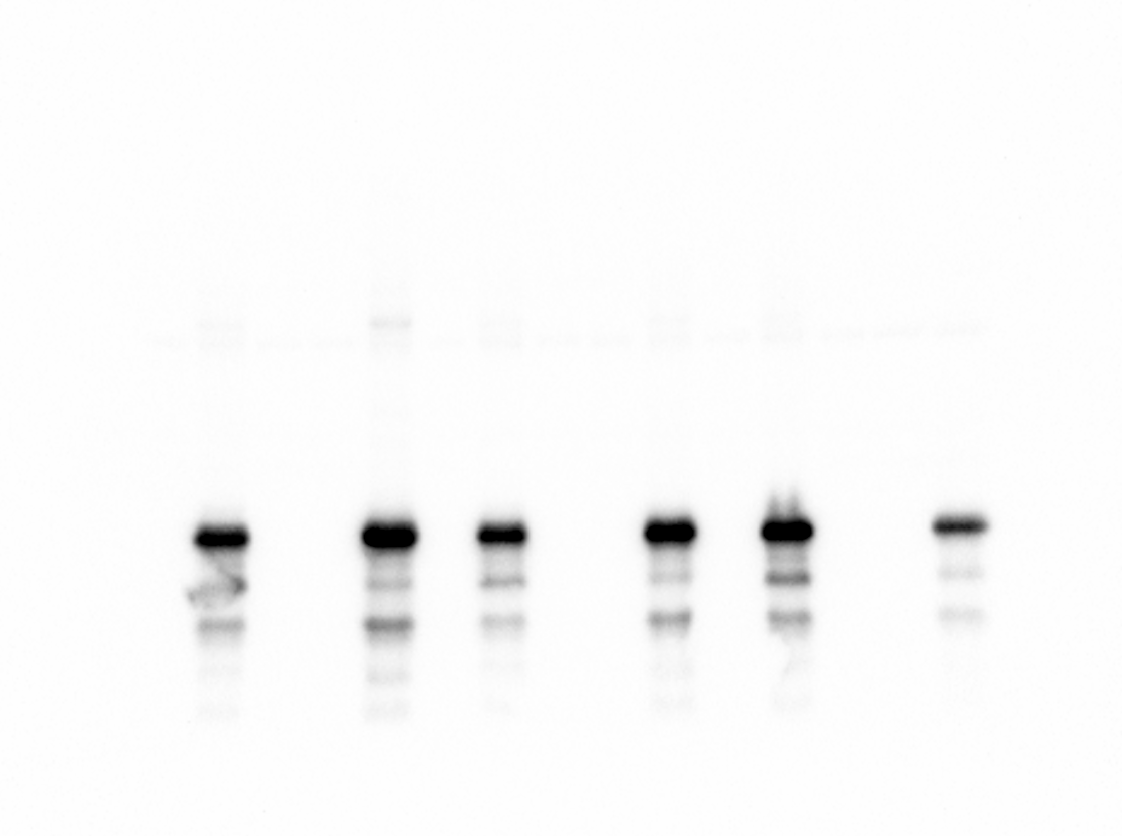

Supplement: Figure 1—figure supplement 2—source data 1. [file elife-74275-fig1-figsupp2-data1.zip › Figure 1-figure supplement 2-source data 1/figure supplement 2B/fig sup 2B anti-GFP.tif]

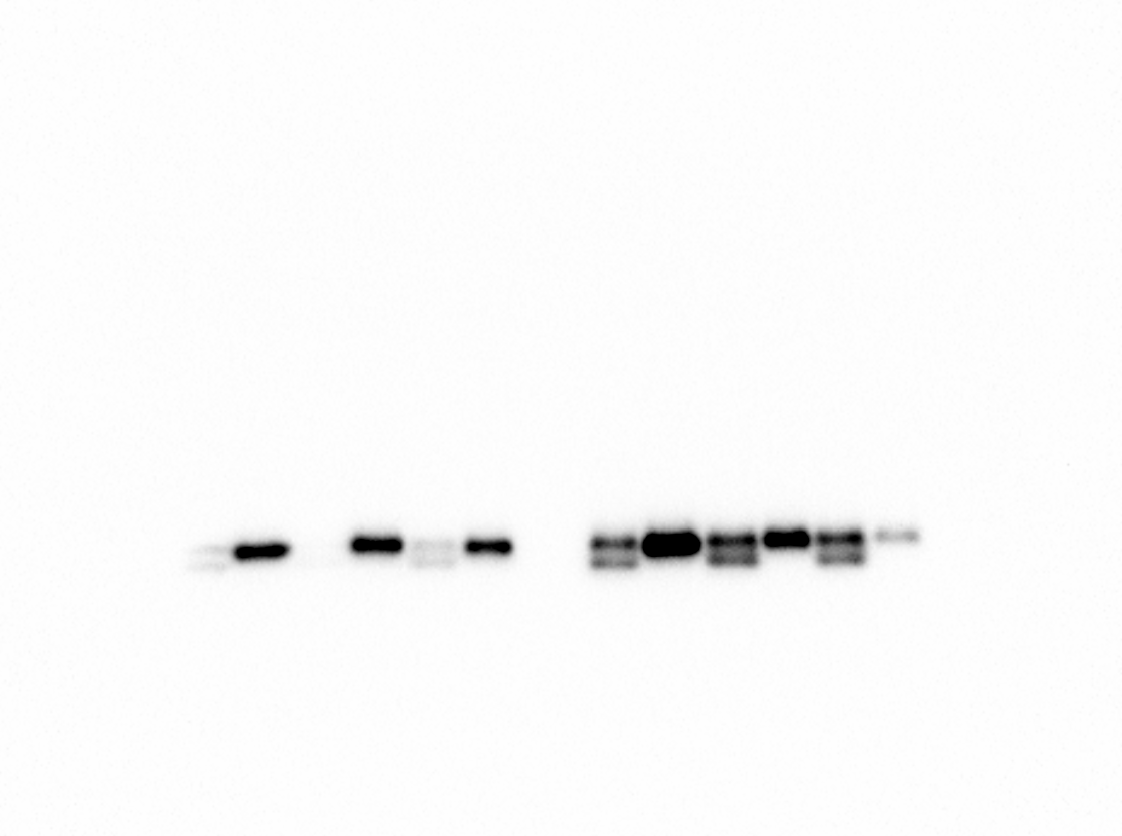

Supplement: Figure 1—figure supplement 2—source data 1. [file elife-74275-fig1-figsupp2-data1.zip › Figure 1-figure supplement 2-source data 1/figure supplement 2B/fig sup 2B anti-His previous blot.tif]

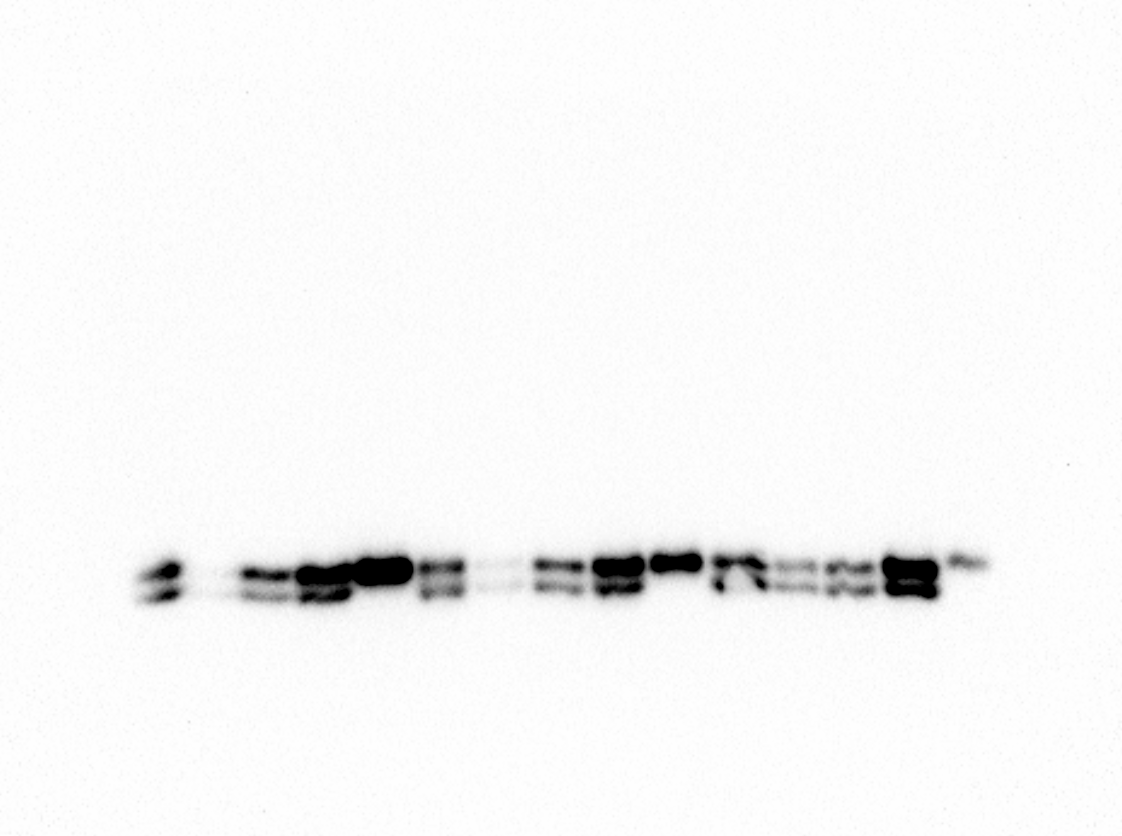

Supplement: Figure 1—figure supplement 2—source data 1. [file elife-74275-fig1-figsupp2-data1.zip › Figure 1-figure supplement 2-source data 1/figure supplement 2B/fig sup 2B anti-His.tif]

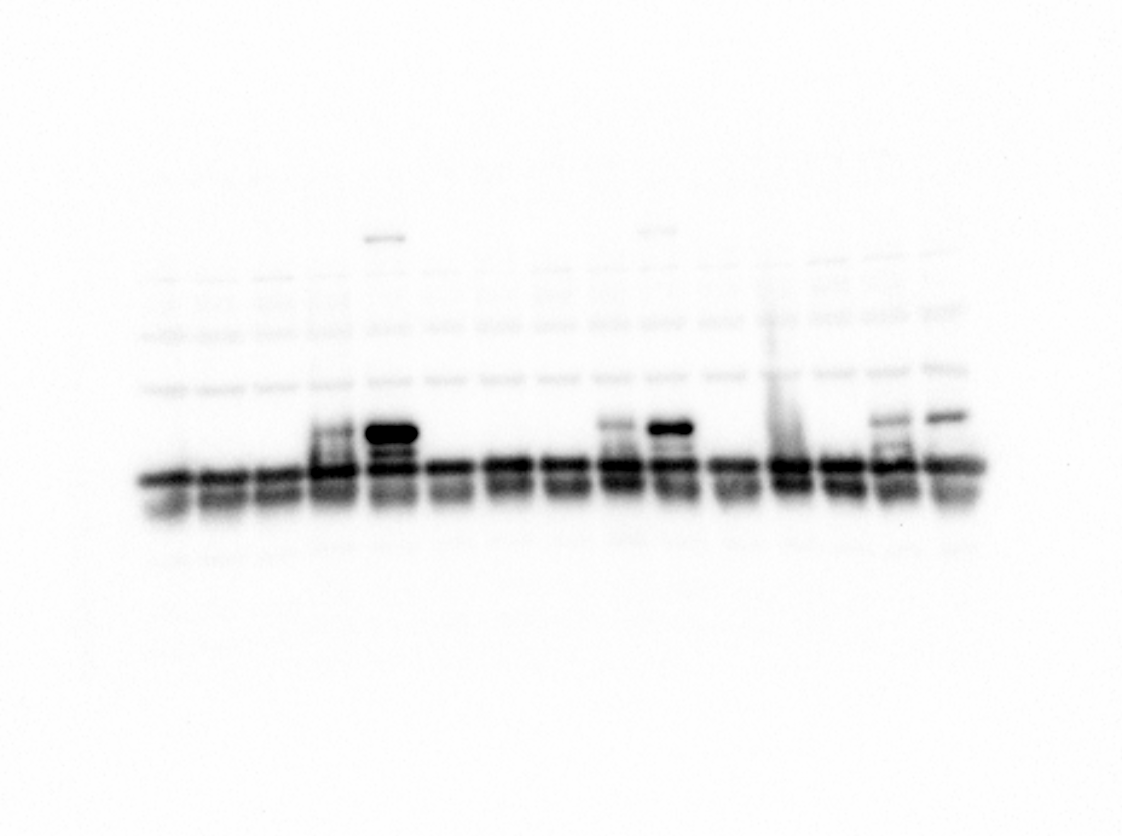

Supplement: Figure 1—figure supplement 2—source data 1. [file elife-74275-fig1-figsupp2-data1.zip › Figure 1-figure supplement 2-source data 1/figure supplement 2B/fig sup 2B anti-IVFA.tif]

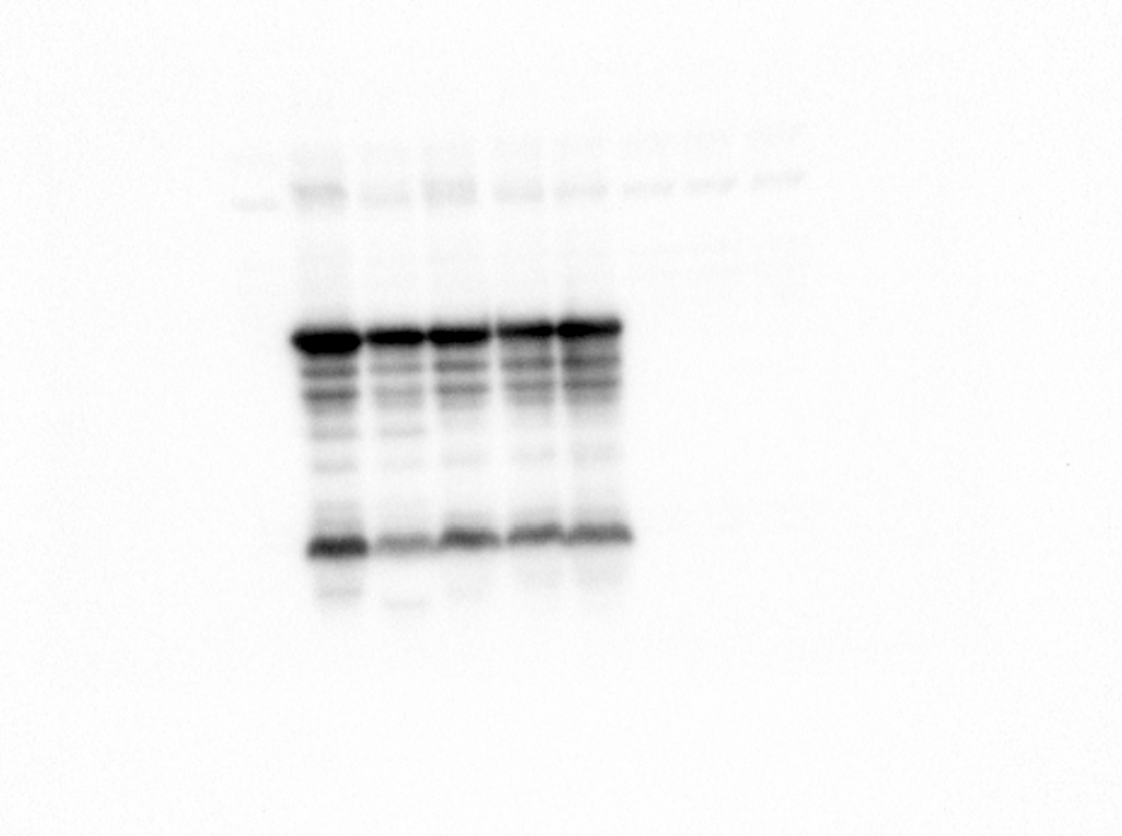

Supplement: Figure 1—figure supplement 3—source data 1. [file elife-74275-fig1-figsupp3-data1.zip › Figure 1-figure supplement 3-source data 1/fig sup 3 Set1 anti-GFP.tif]

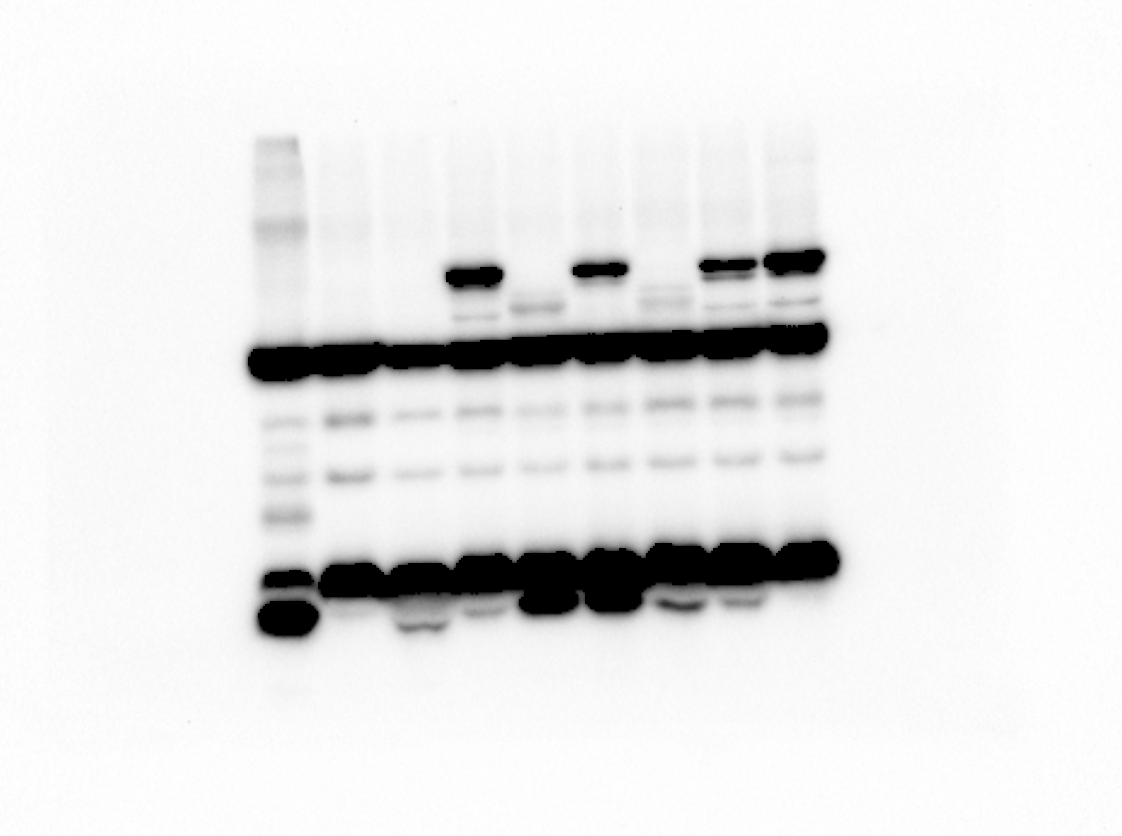

Supplement: Figure 1—figure supplement 3—source data 1. [file elife-74275-fig1-figsupp3-data1.zip › Figure 1-figure supplement 3-source data 1/fig sup 3 Set1 anti-His long exposure.tif]

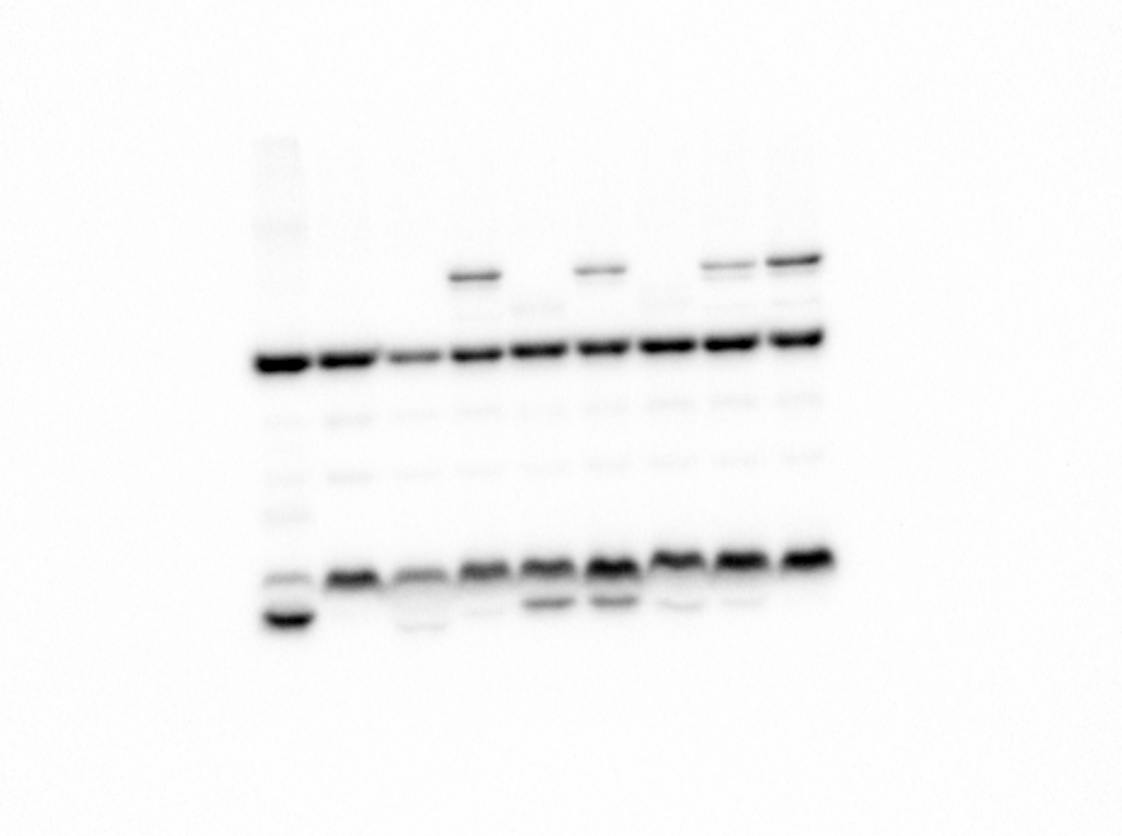

Supplement: Figure 1—figure supplement 3—source data 1. [file elife-74275-fig1-figsupp3-data1.zip › Figure 1-figure supplement 3-source data 1/fig sup 3 Set1 anti-His.tif]

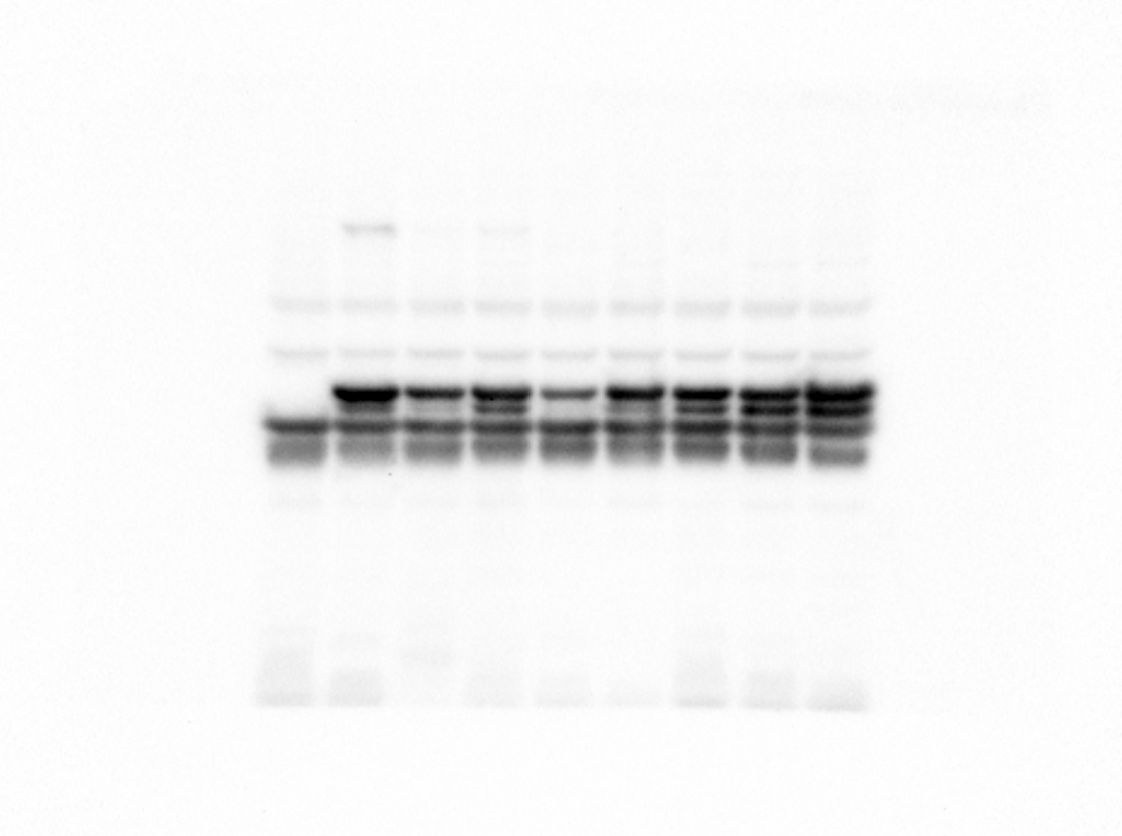

Supplement: Figure 1—figure supplement 3—source data 1. [file elife-74275-fig1-figsupp3-data1.zip › Figure 1-figure supplement 3-source data 1/fig sup 3 Set1 anti-IVFA.tif]

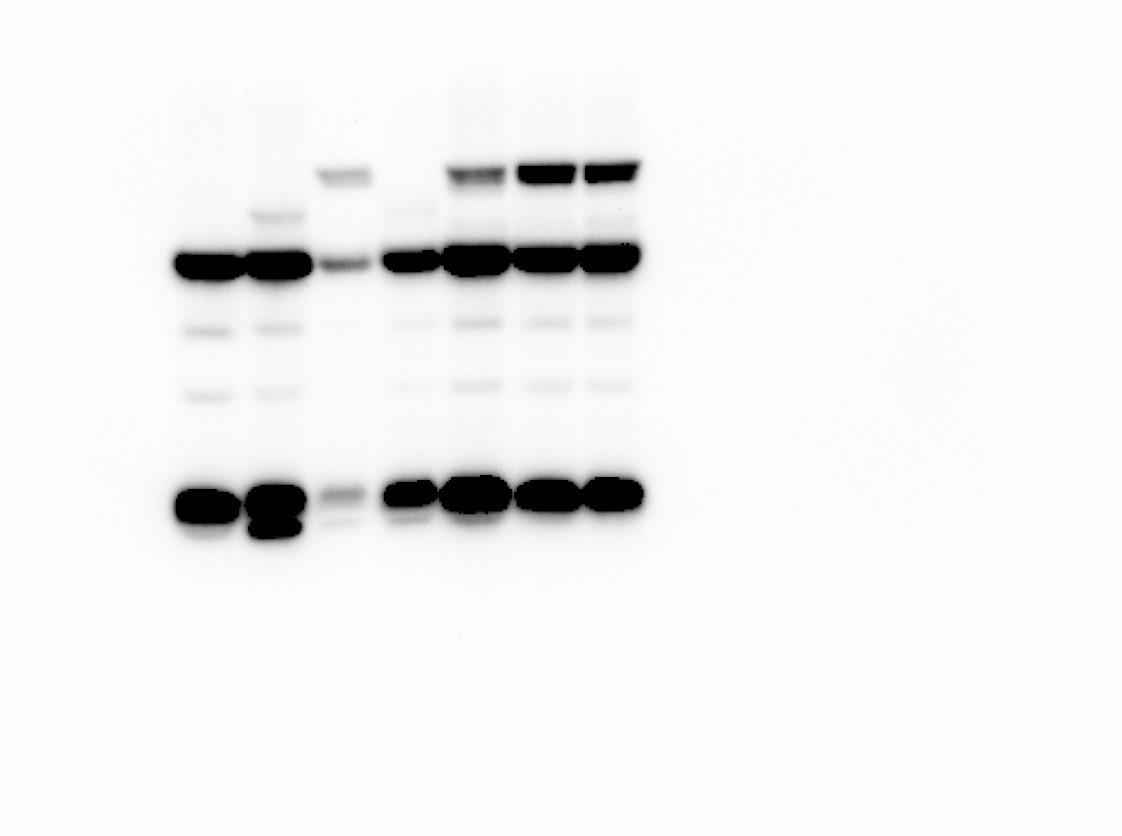

Supplement: Figure 1—figure supplement 3—source data 1. [file elife-74275-fig1-figsupp3-data1.zip › Figure 1-figure supplement 3-source data 1/fig sup 3 Set2 anti His long exposure.tif]

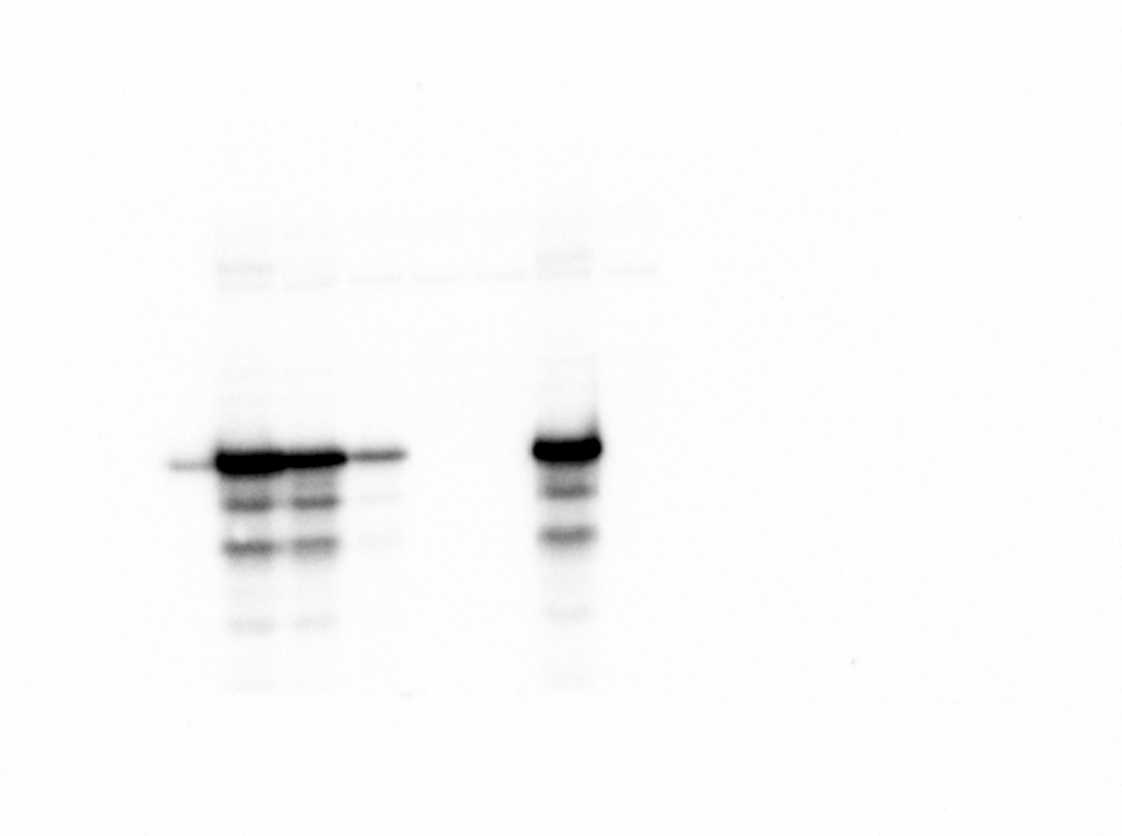

Supplement: Figure 1—figure supplement 3—source data 1. [file elife-74275-fig1-figsupp3-data1.zip › Figure 1-figure supplement 3-source data 1/fig sup 3 Set2 anti-GFP.tif]

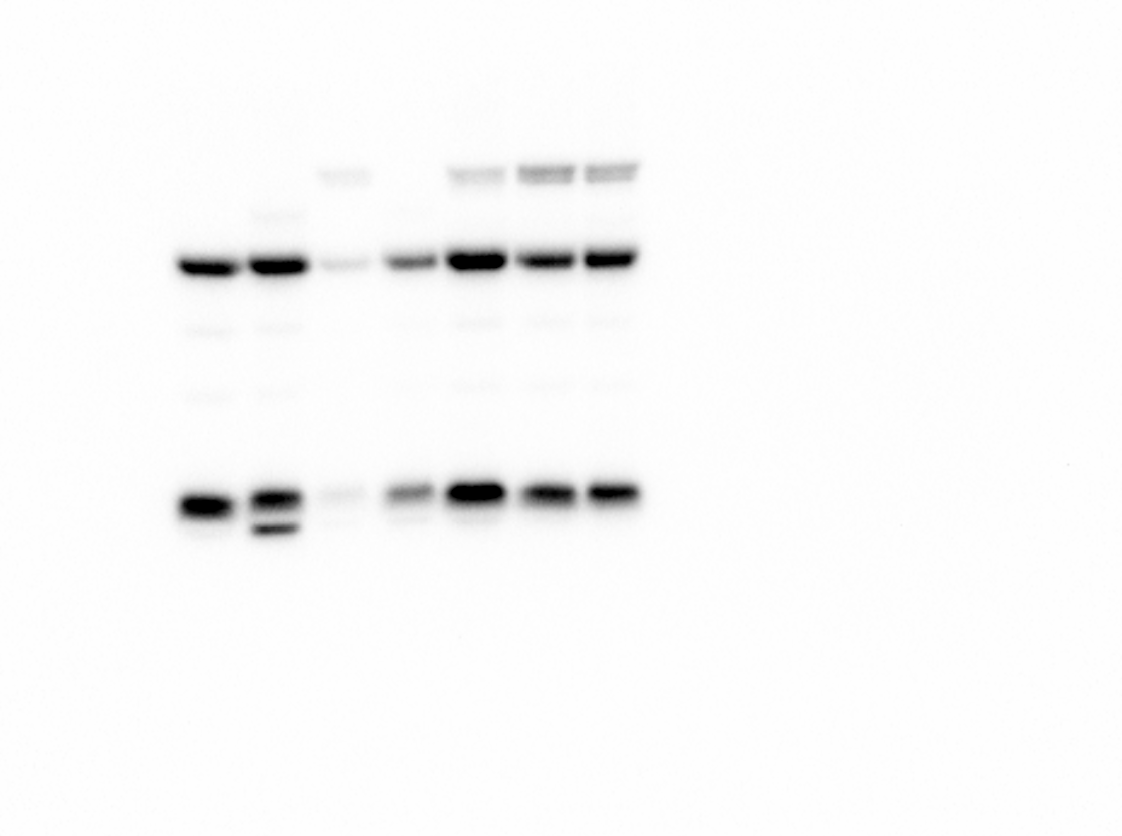

Supplement: Figure 1—figure supplement 3—source data 1. [file elife-74275-fig1-figsupp3-data1.zip › Figure 1-figure supplement 3-source data 1/fig sup 3 Set2 anti-His.tif]

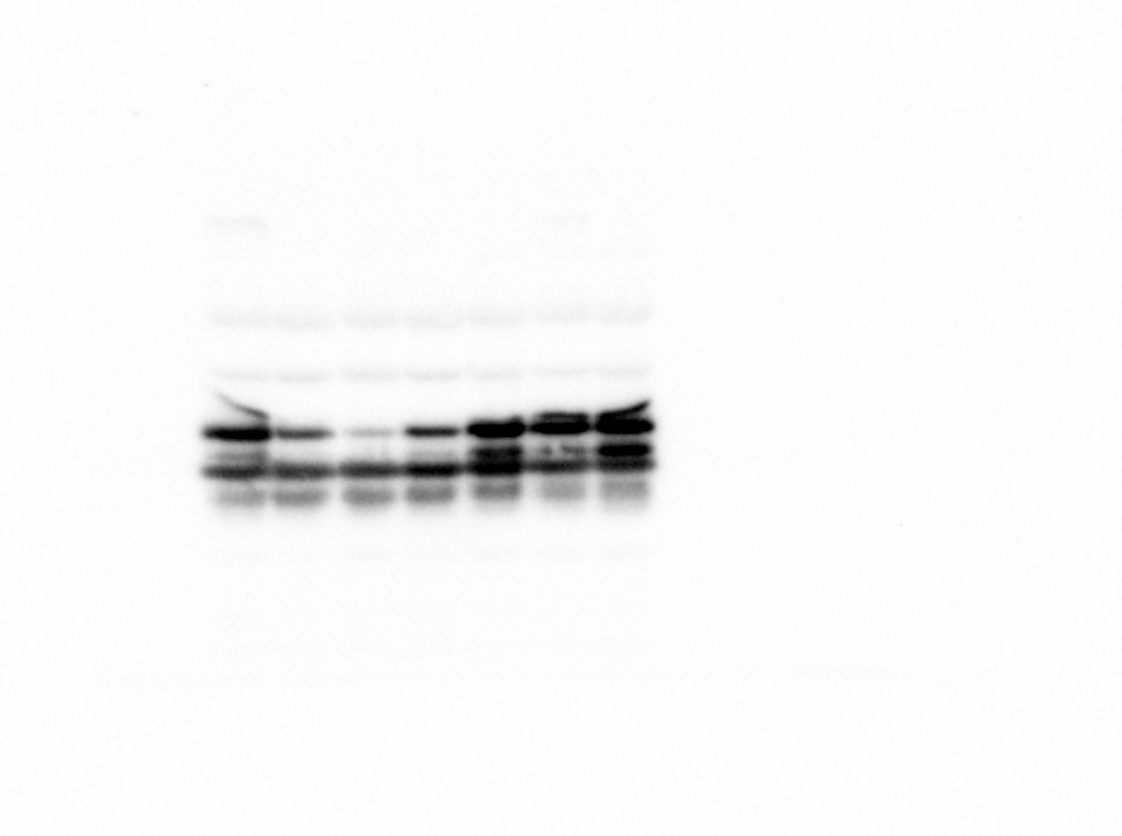

Supplement: Figure 1—figure supplement 3—source data 1. [file elife-74275-fig1-figsupp3-data1.zip › Figure 1-figure supplement 3-source data 1/fig sup 3 Set2 anti-IVFA.tif]

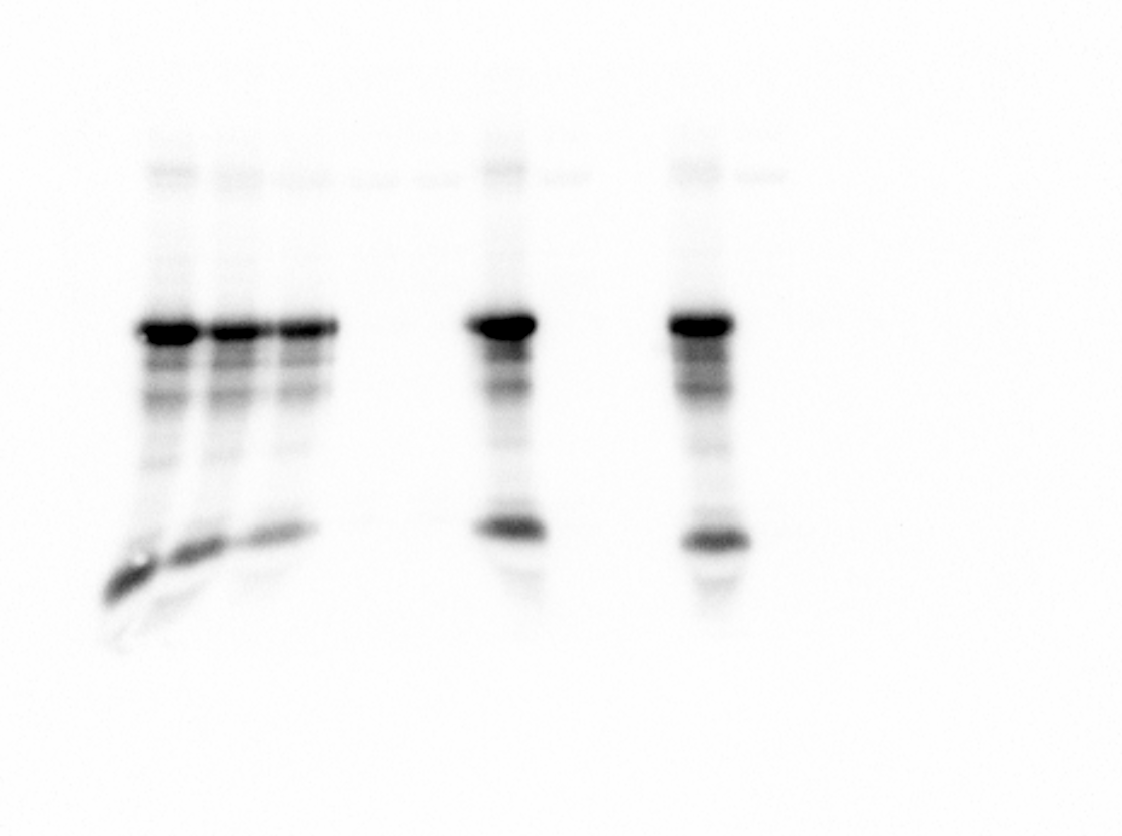

Supplement: Figure 1—figure supplement 3—source data 1. [file elife-74275-fig1-figsupp3-data1.zip › Figure 1-figure supplement 3-source data 1/fig sup 3 Set3 anti-GFP.tif]

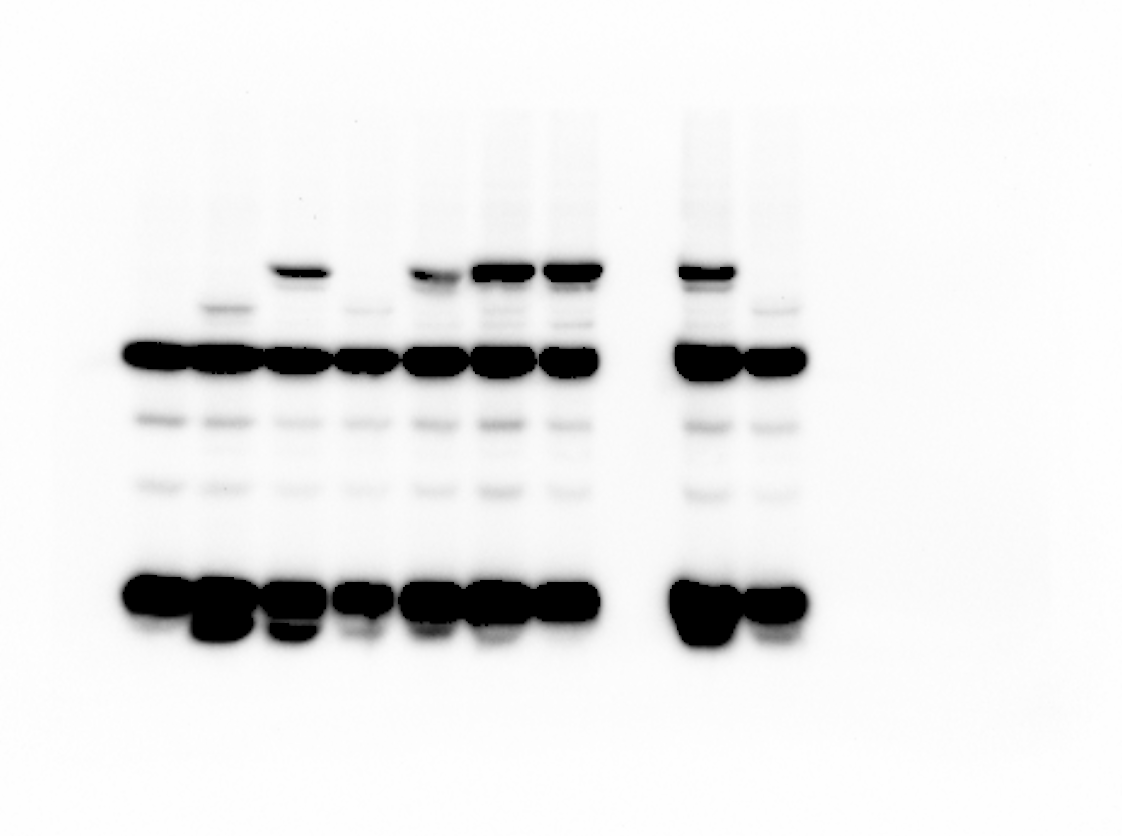

Supplement: Figure 1—figure supplement 3—source data 1. [file elife-74275-fig1-figsupp3-data1.zip › Figure 1-figure supplement 3-source data 1/fig sup 3 Set3 anti-His long exposure.tif]

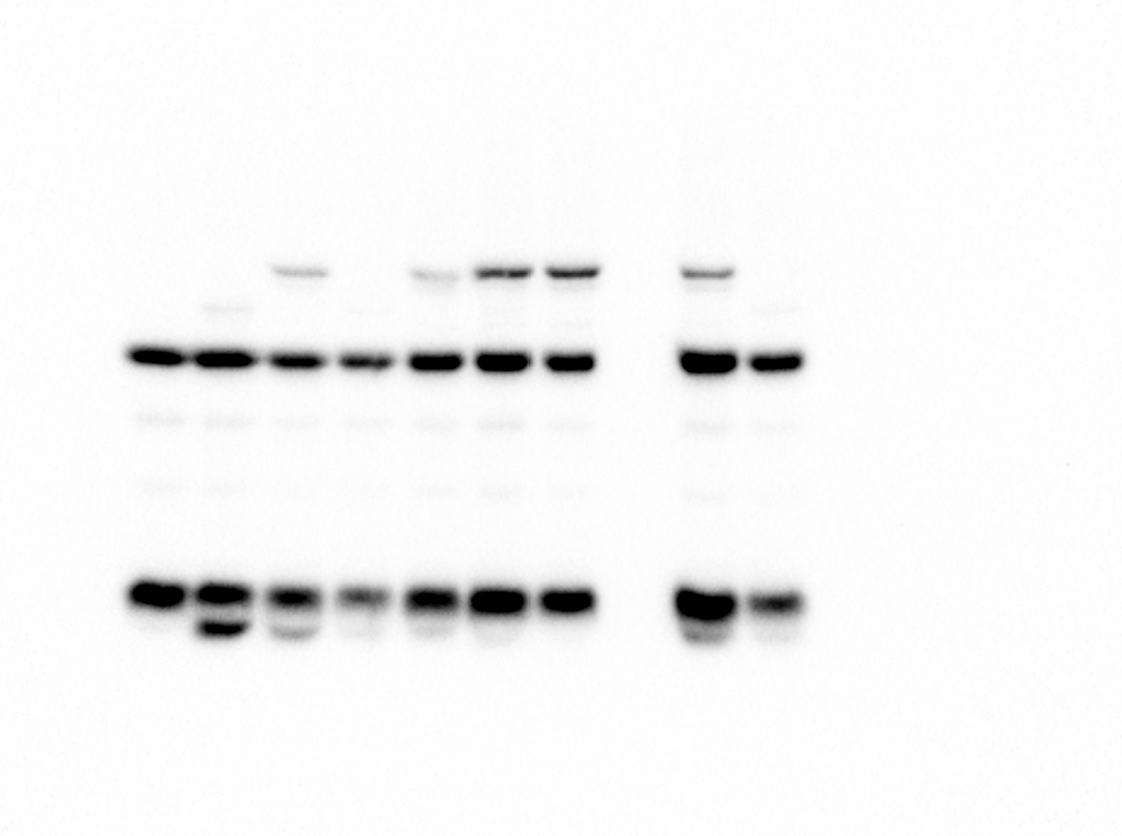

Supplement: Figure 1—figure supplement 3—source data 1. [file elife-74275-fig1-figsupp3-data1.zip › Figure 1-figure supplement 3-source data 1/fig sup 3 Set3 anti-His.tif]

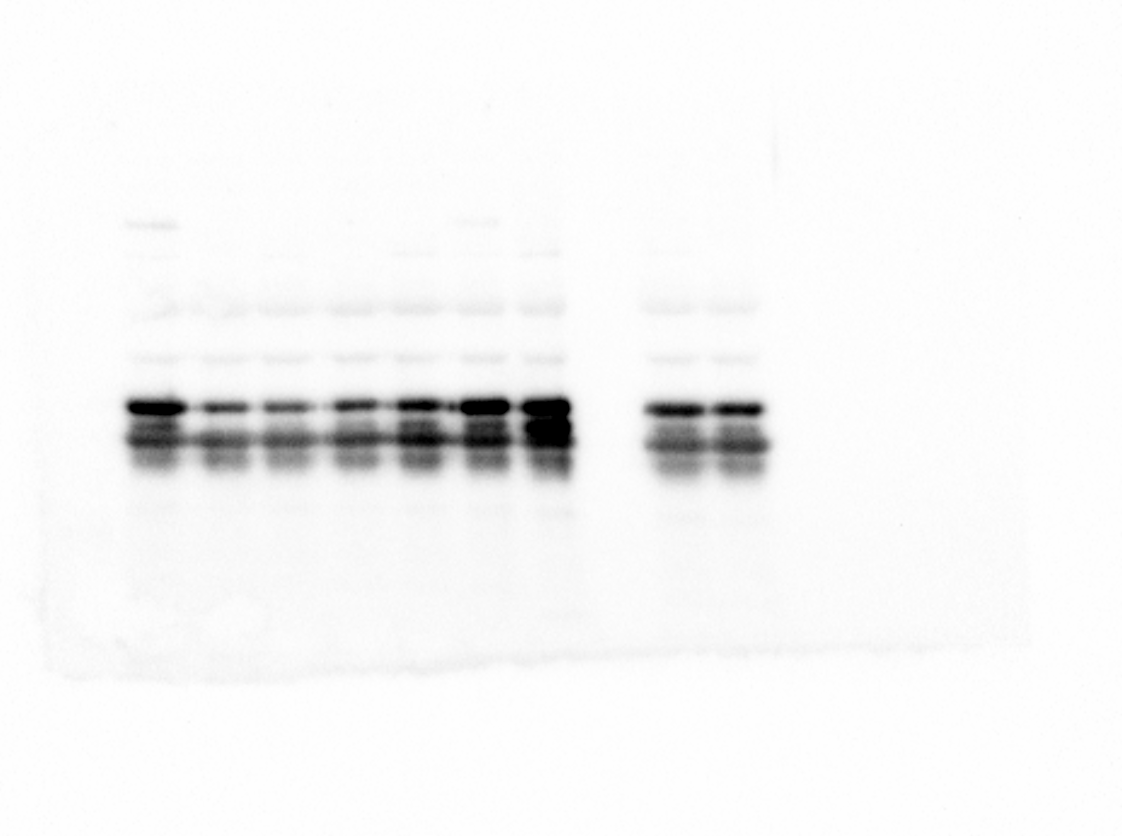

Supplement: Figure 1—figure supplement 3—source data 1. [file elife-74275-fig1-figsupp3-data1.zip › Figure 1-figure supplement 3-source data 1/fig sup 3 Set3 anti-IVFA.tif]

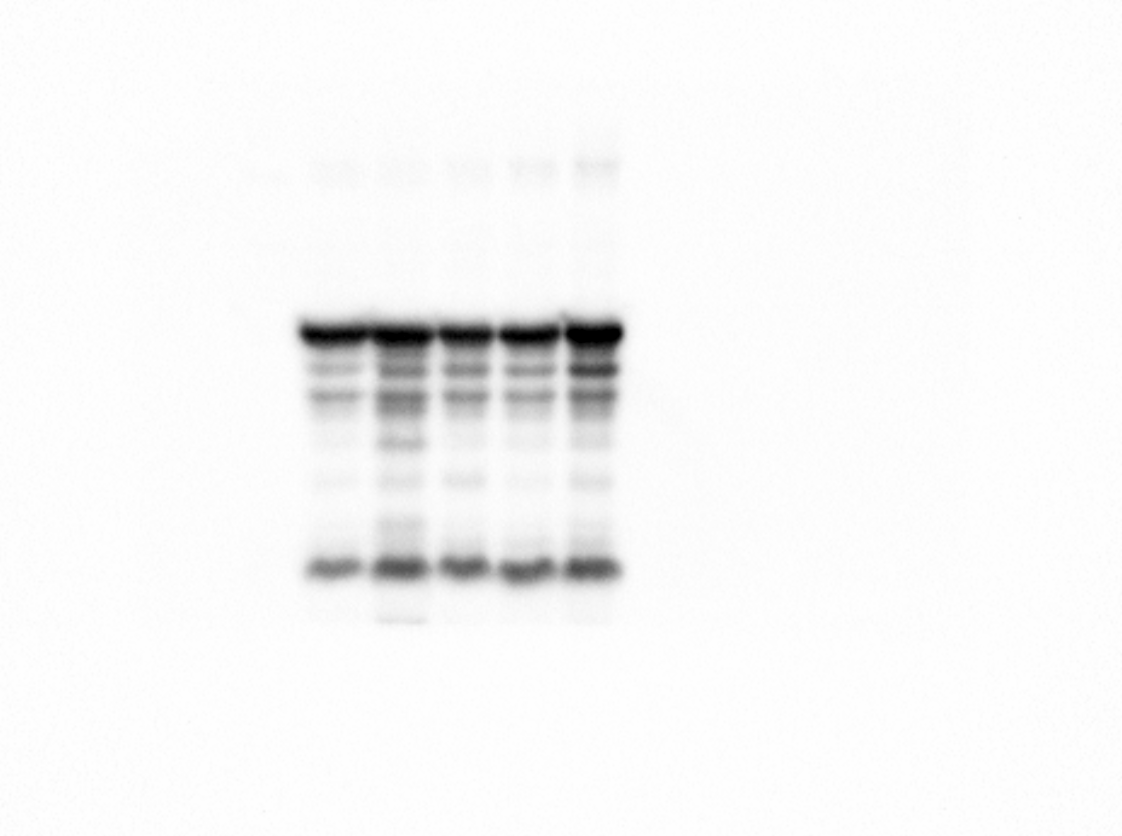

Supplement: Figure 1—figure supplement 4—source data 1. [file elife-74275-fig1-figsupp4-data1.zip › Figure 1-figure supplement 4-source data 1/fig sup 4 Set1 anti-GFP.tif]

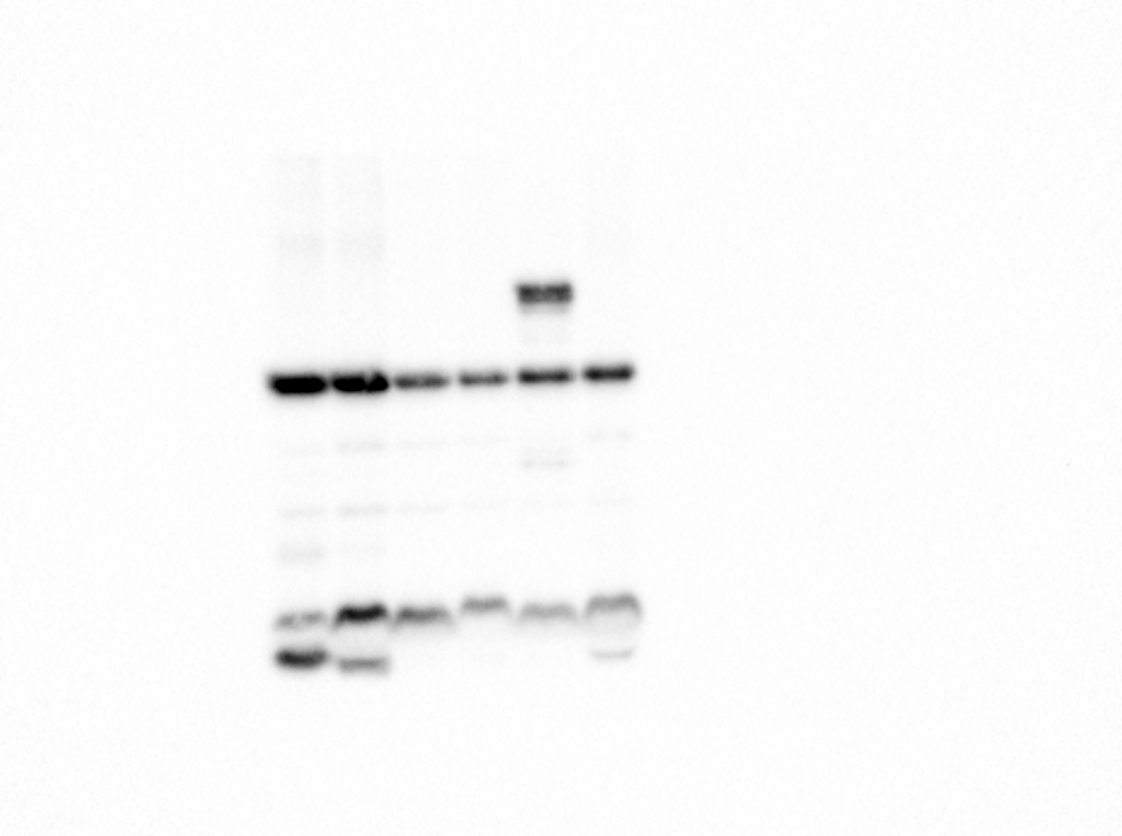

Supplement: Figure 1—figure supplement 4—source data 1. [file elife-74275-fig1-figsupp4-data1.zip › Figure 1-figure supplement 4-source data 1/fig sup 4 Set1 anti-His.tif]

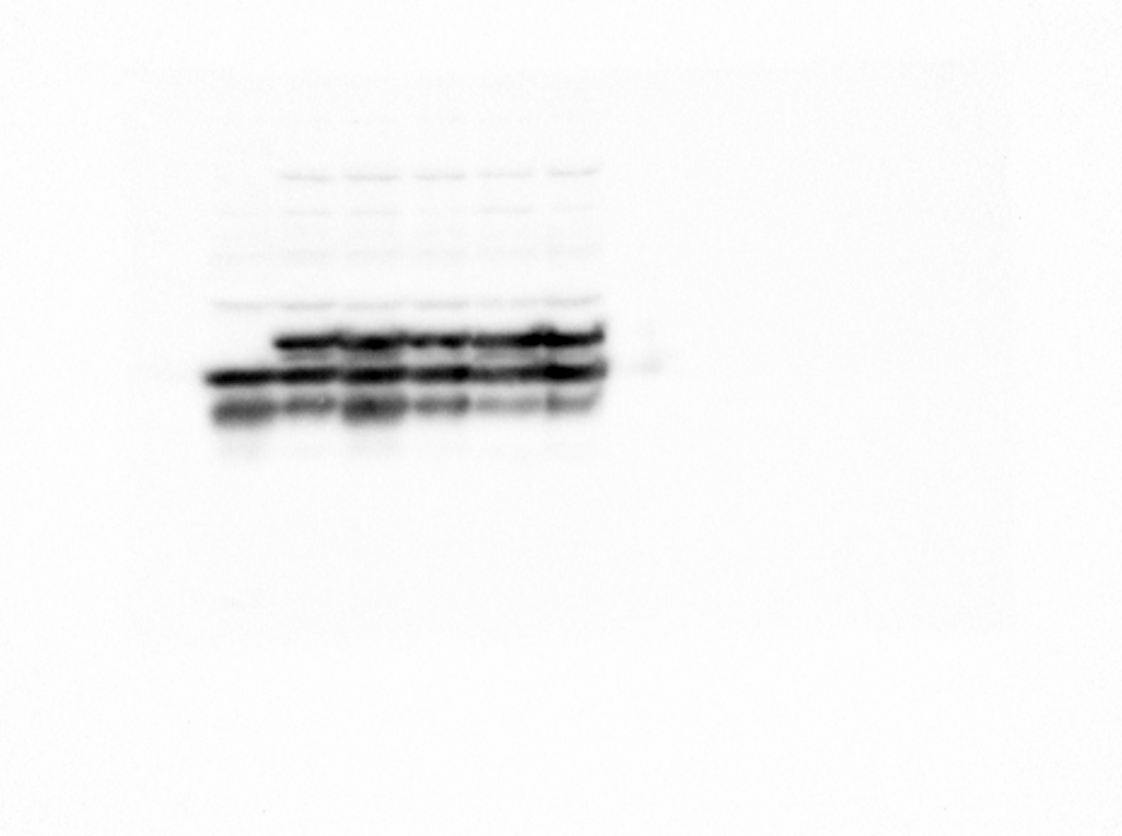

Supplement: Figure 1—figure supplement 4—source data 1. [file elife-74275-fig1-figsupp4-data1.zip › Figure 1-figure supplement 4-source data 1/fig sup 4 Set1 anti-IVFA.tif]

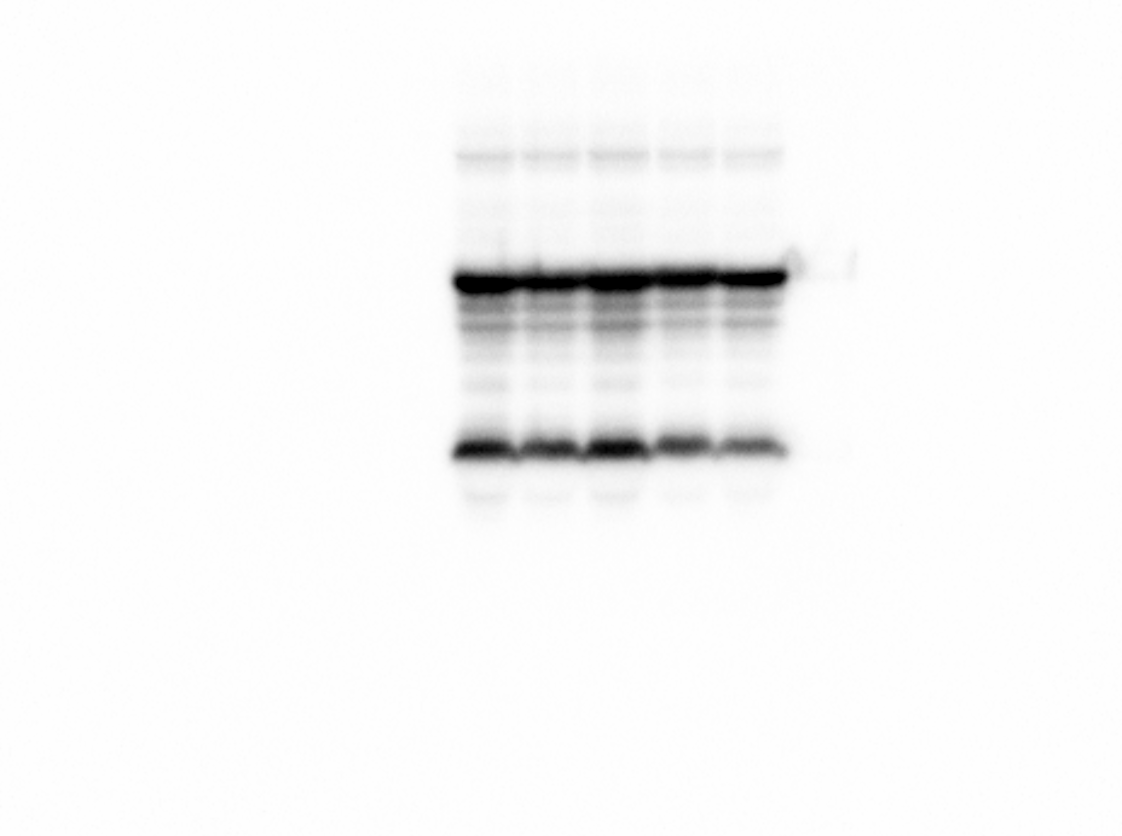

Supplement: Figure 1—figure supplement 4—source data 1. [file elife-74275-fig1-figsupp4-data1.zip › Figure 1-figure supplement 4-source data 1/fig sup 4 Sets 2 and 3 anti-GFP.tif]

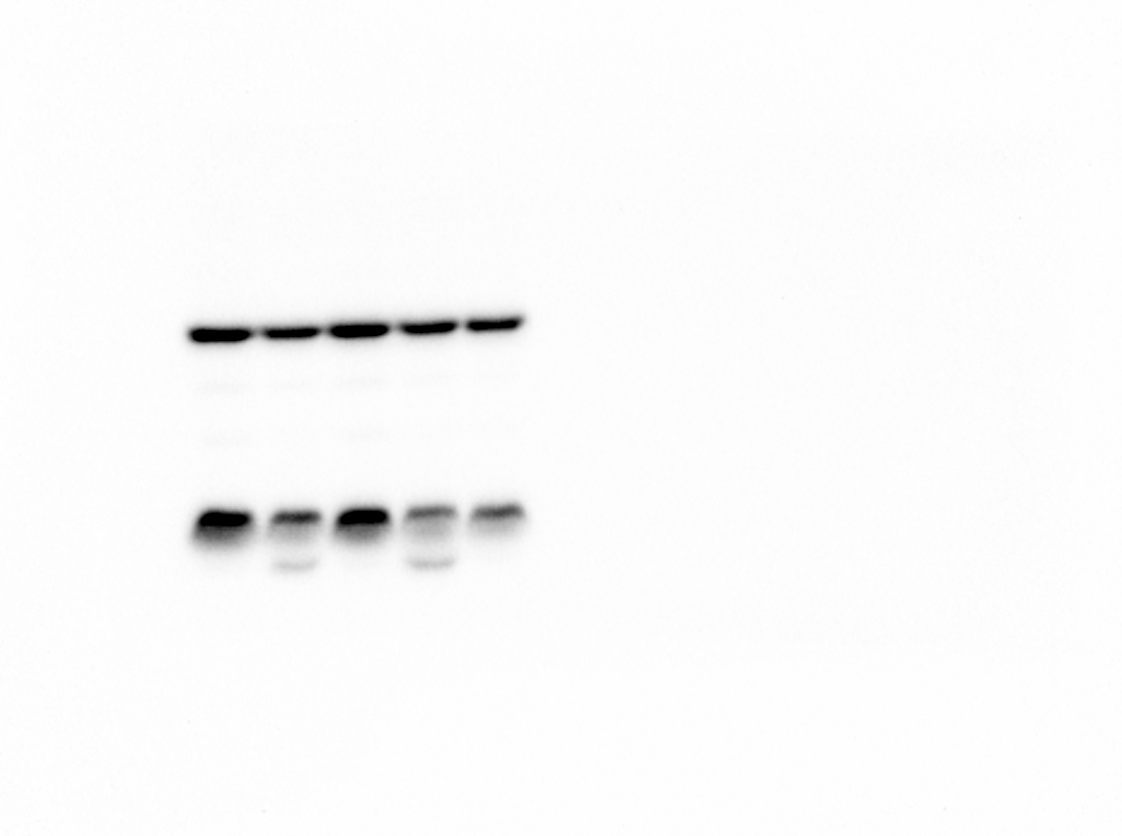

Supplement: Figure 1—figure supplement 4—source data 1. [file elife-74275-fig1-figsupp4-data1.zip › Figure 1-figure supplement 4-source data 1/fig sup 4 Sets 2 and 3 anti-His.tif]

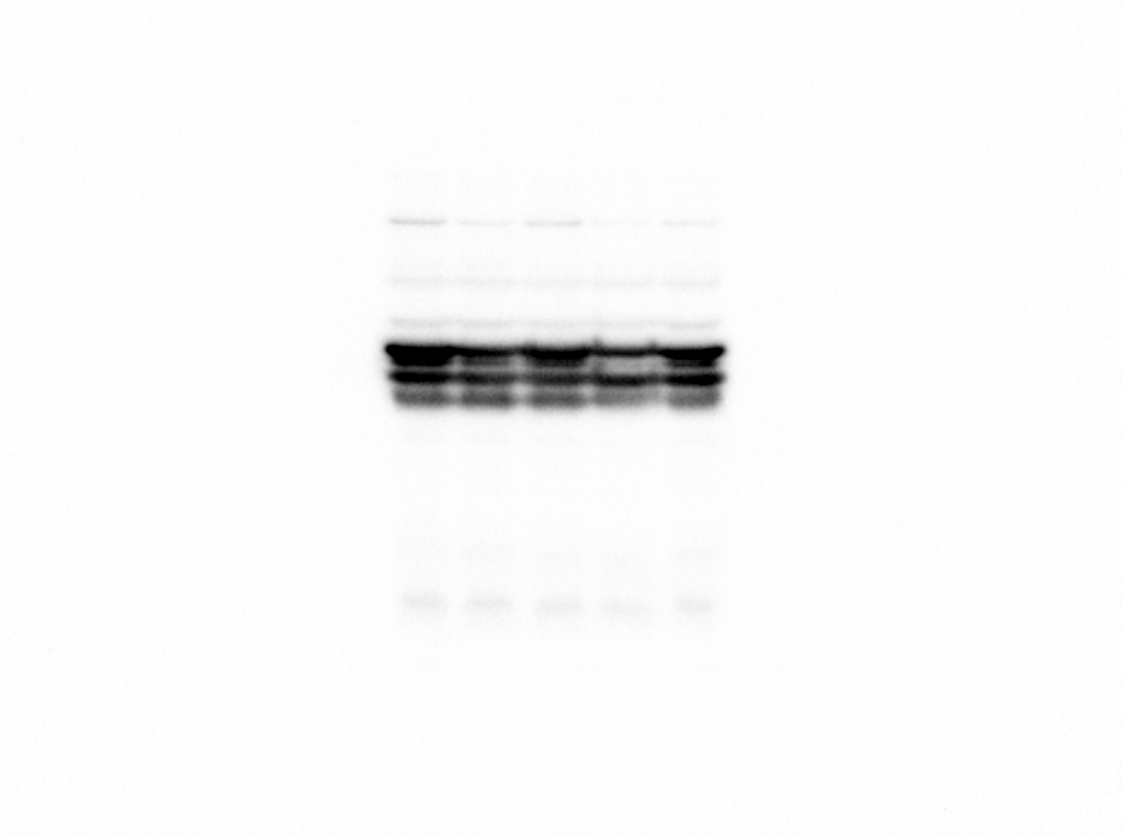

Supplement: Figure 1—figure supplement 4—source data 1. [file elife-74275-fig1-figsupp4-data1.zip › Figure 1-figure supplement 4-source data 1/fig sup 4 Sets 2 and 3 anti-IVFA.tif]

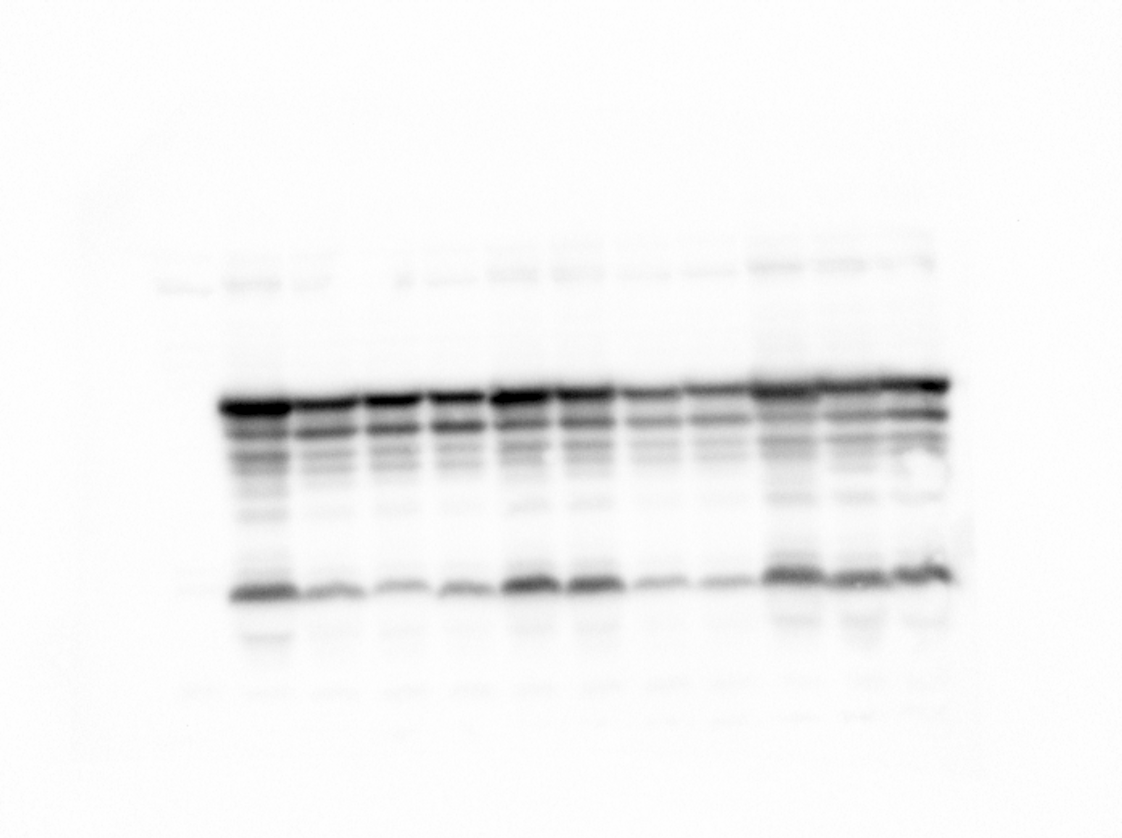

Supplement: Figure 2—source data 1. [file elife-74275-fig2-data1.zip › Figure 2-source data 1/Fig2 Blot1 Set1 anti-GFP.tif]

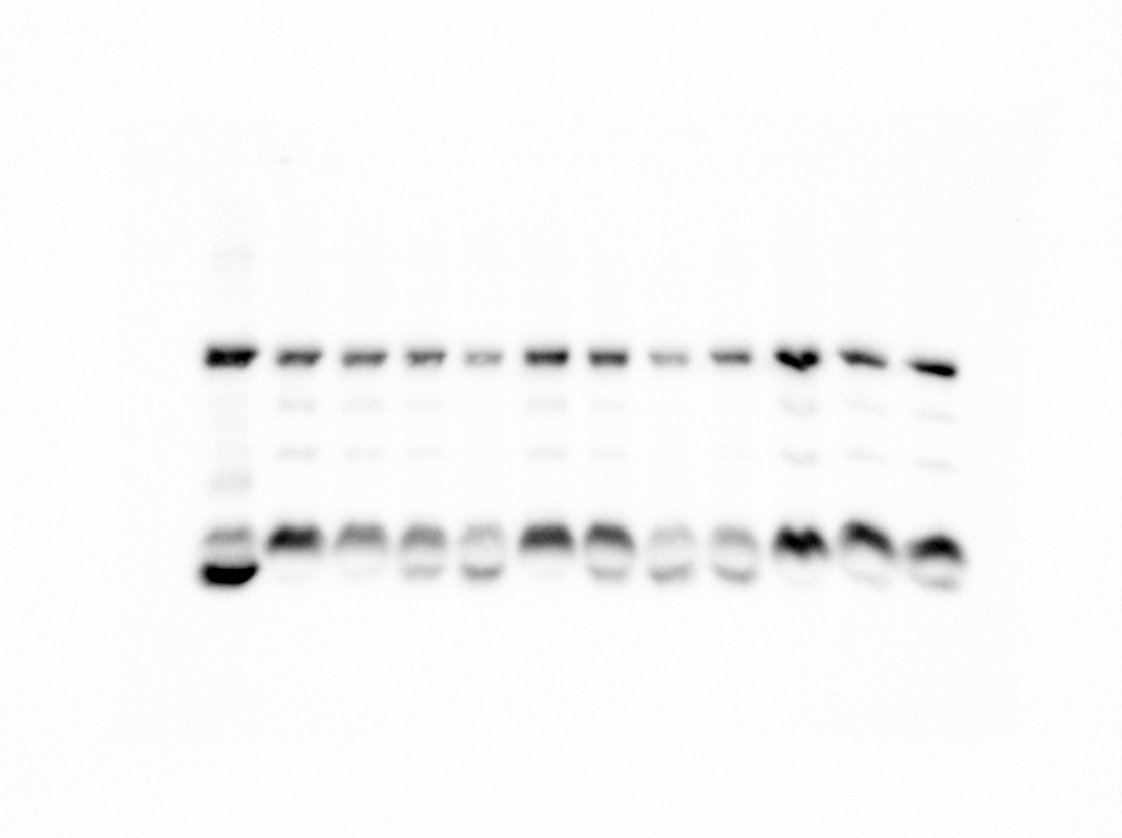

Supplement: Figure 2—source data 1. [file elife-74275-fig2-data1.zip › Figure 2-source data 1/Fig2 Blot1 Set1 anti-His.tif]

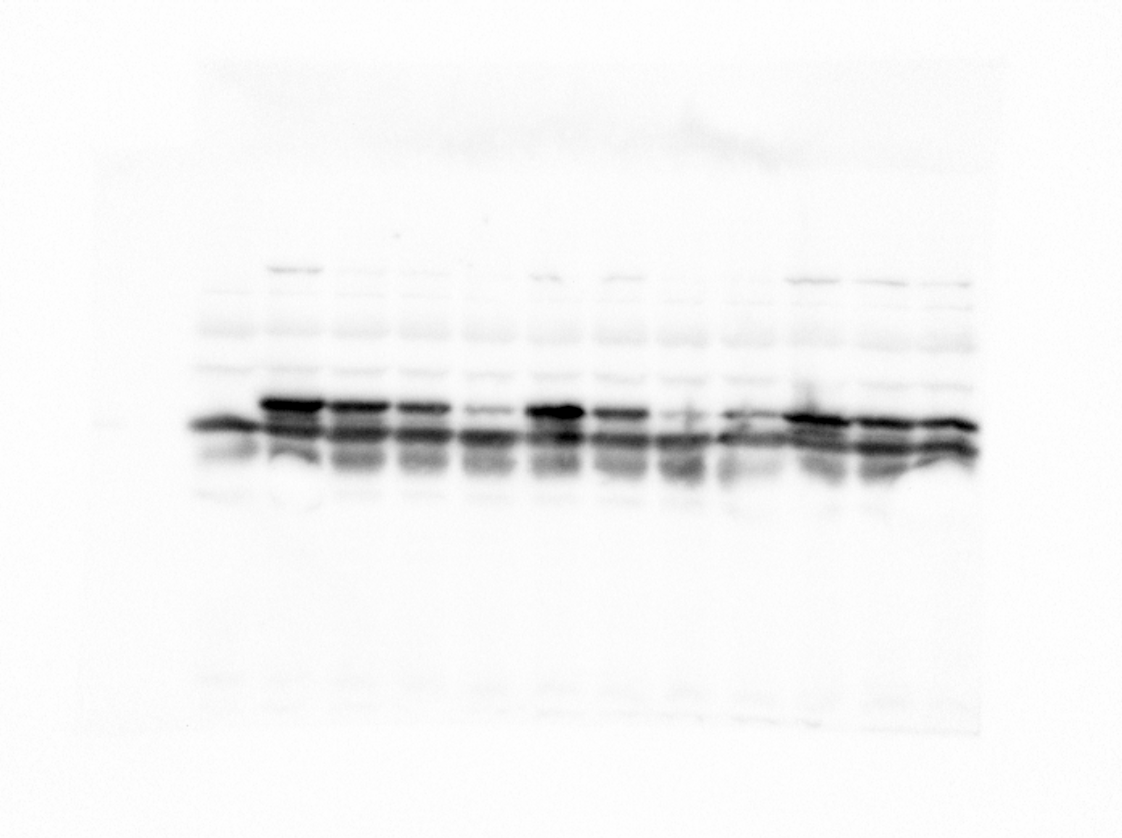

Supplement: Figure 2—source data 1. [file elife-74275-fig2-data1.zip › Figure 2-source data 1/Fig2 Blot1 Set1 anti-IVFA.tif]

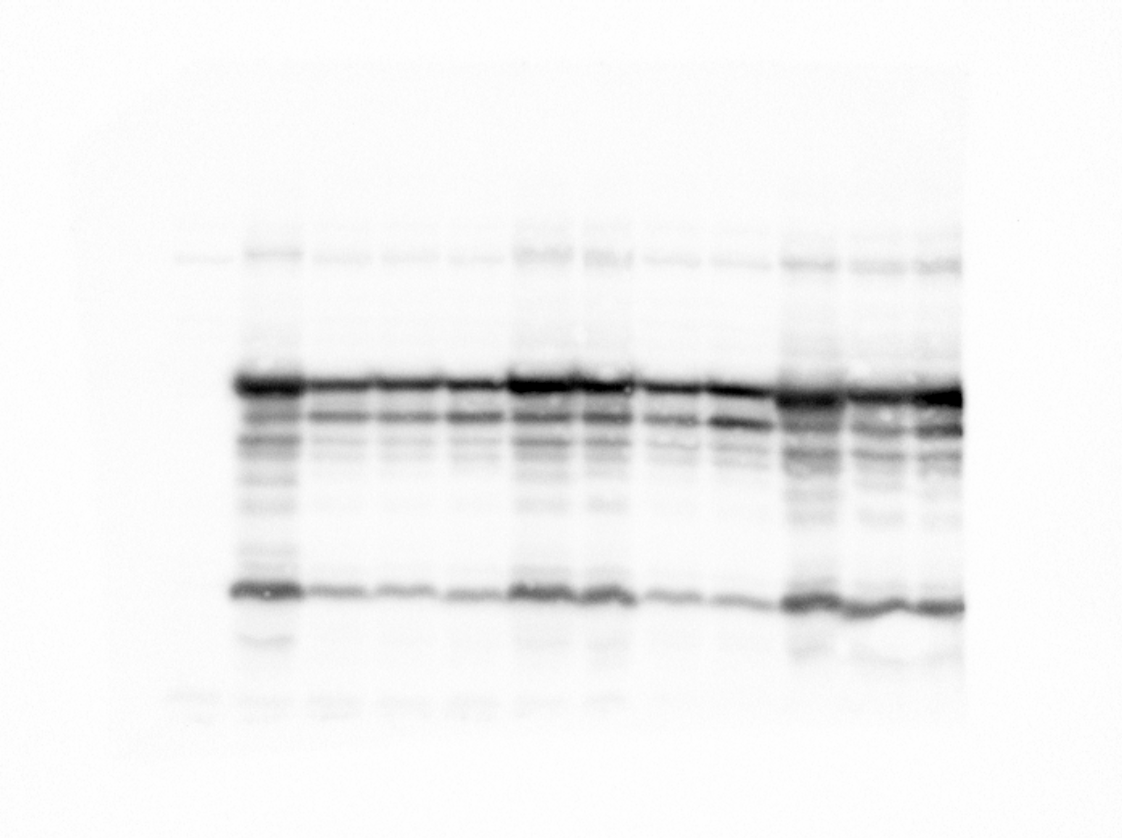

Supplement: Figure 2—source data 1. [file elife-74275-fig2-data1.zip › Figure 2-source data 1/Fig2 Blot1 Set2 anti-GFP.tif]

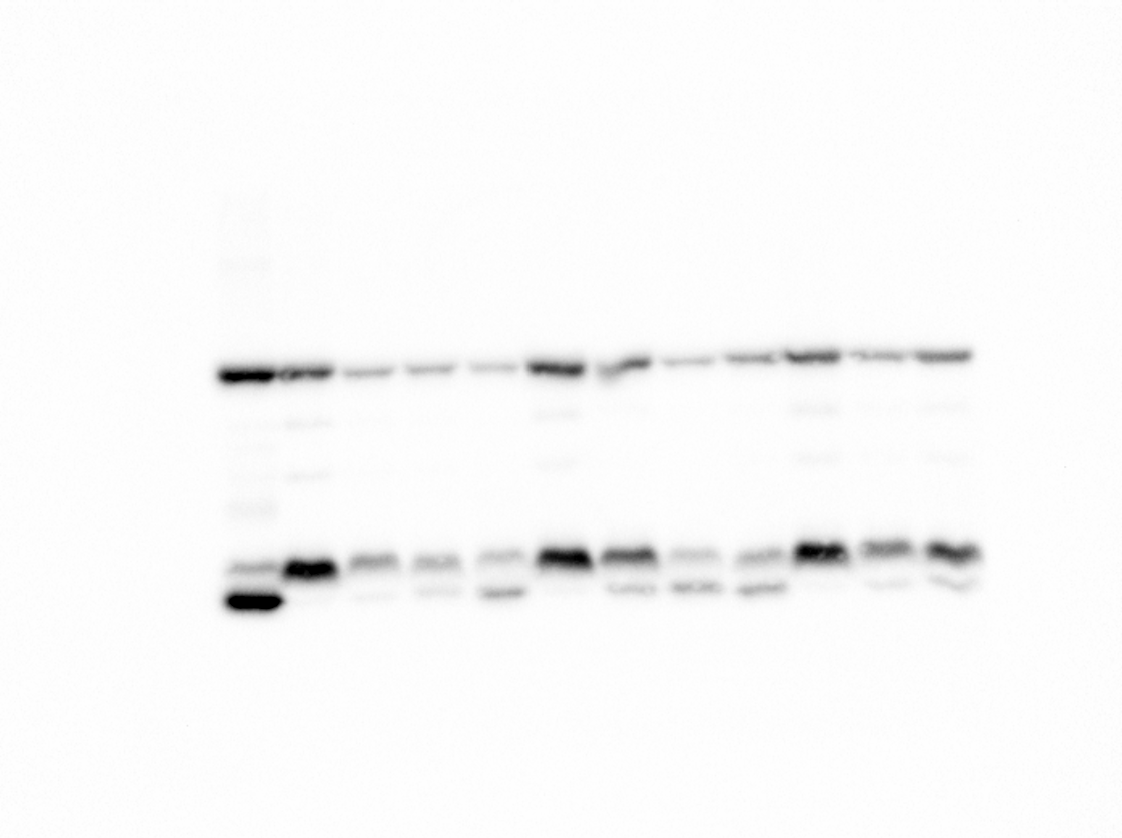

Supplement: Figure 2—source data 1. [file elife-74275-fig2-data1.zip › Figure 2-source data 1/Fig2 Blot1 Set2 anti-His.tif]

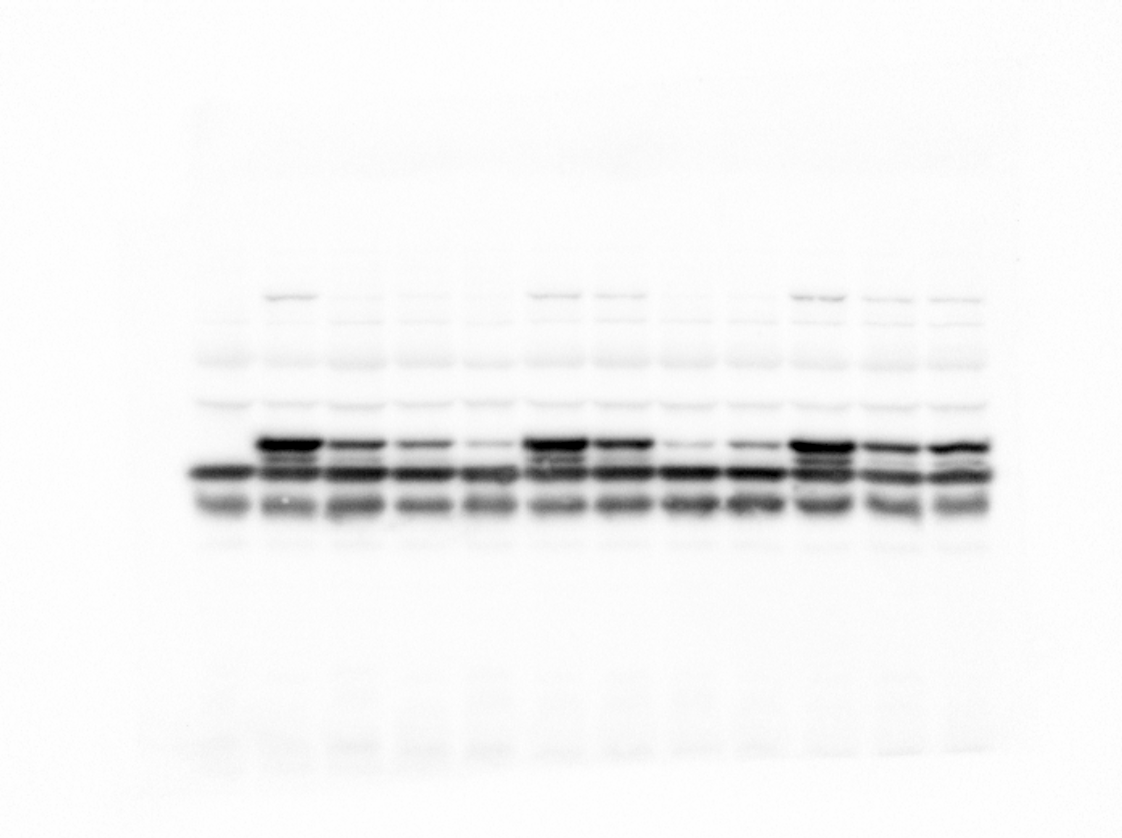

Supplement: Figure 2—source data 1. [file elife-74275-fig2-data1.zip › Figure 2-source data 1/Fig2 Blot1 Set2 anti-IVFA.tif]

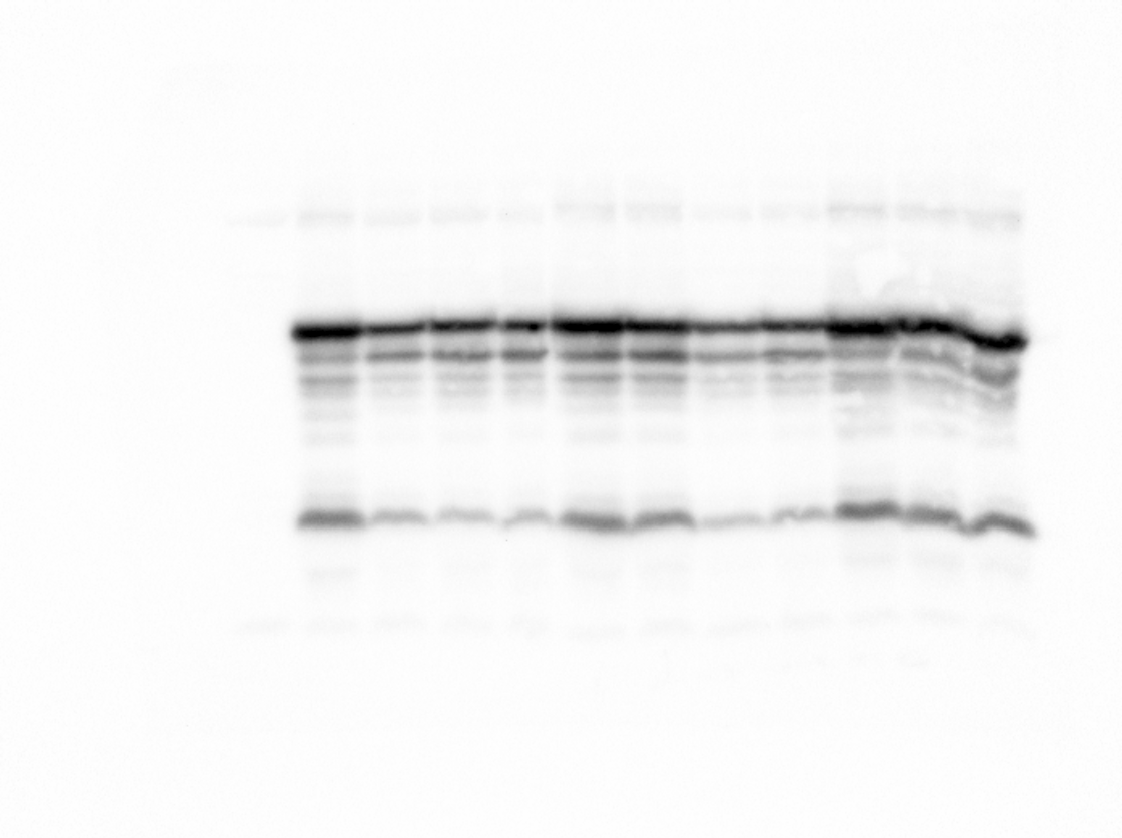

Supplement: Figure 2—source data 1. [file elife-74275-fig2-data1.zip › Figure 2-source data 1/Fig2 Blot1 Set3 anti-GFP.tif]

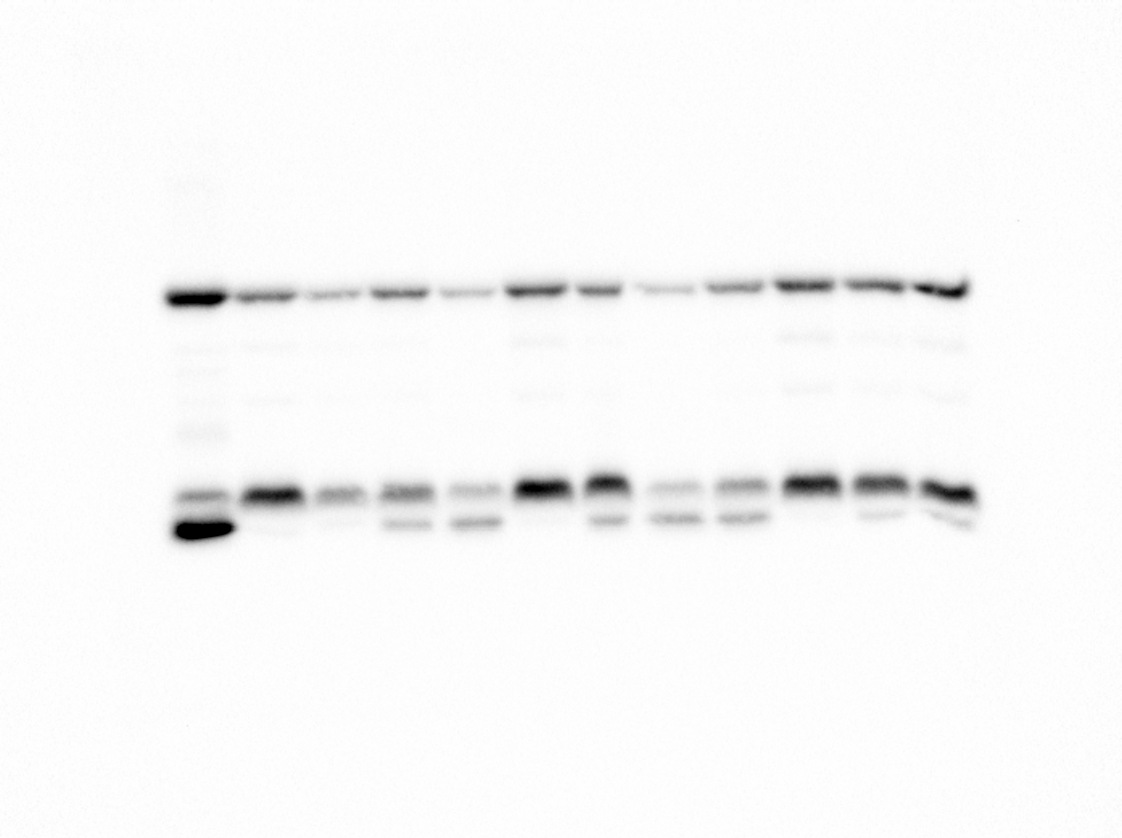

Supplement: Figure 2—source data 1. [file elife-74275-fig2-data1.zip › Figure 2-source data 1/Fig2 Blot1 Set3 anti-His.tif]

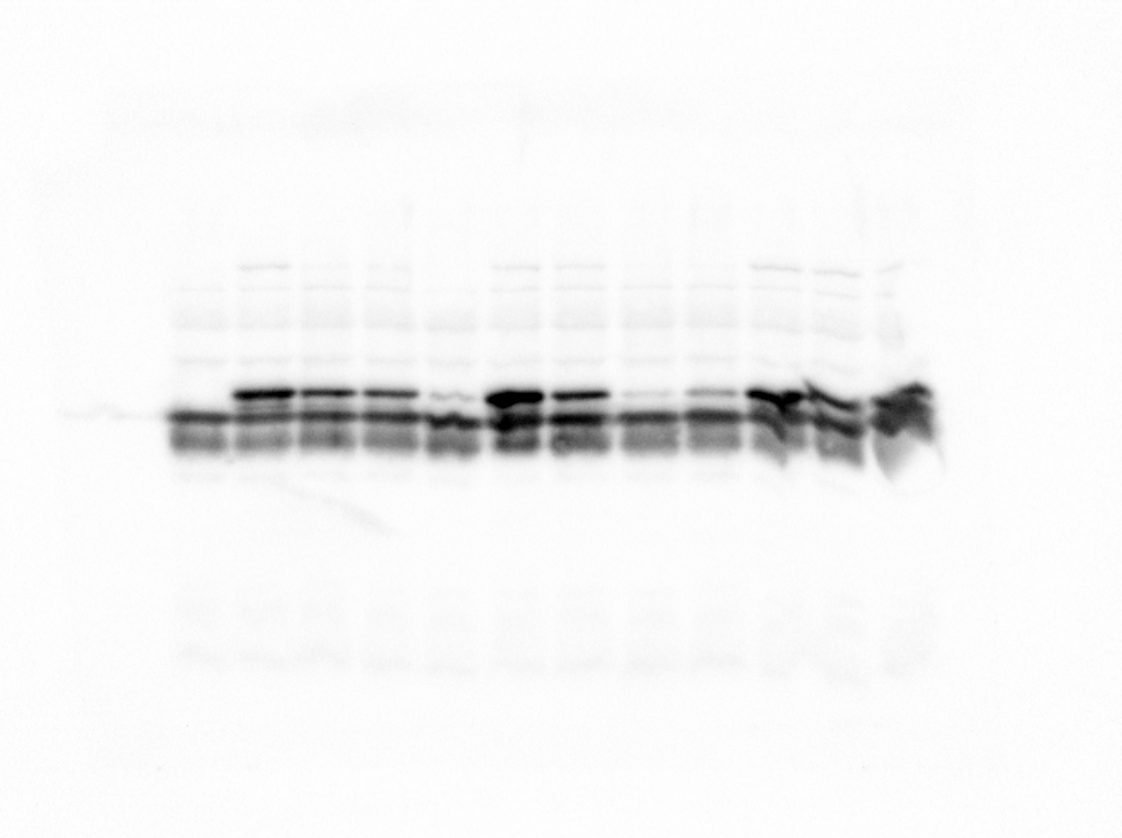

Supplement: Figure 2—source data 1. [file elife-74275-fig2-data1.zip › Figure 2-source data 1/Fig2 Blot1 Set3 anti-IVFA.tif]

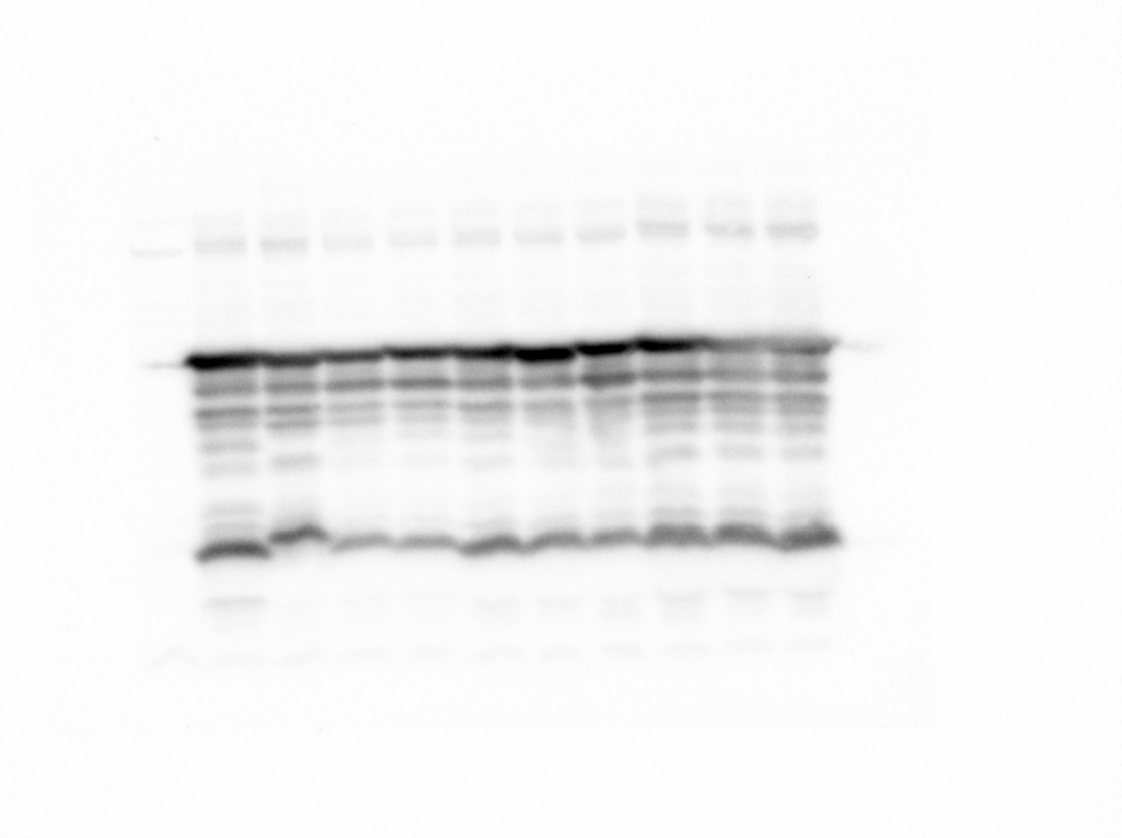

Supplement: Figure 2—source data 1. [file elife-74275-fig2-data1.zip › Figure 2-source data 1/Fig2 Blot2 Set1 anti-GFP.tif]

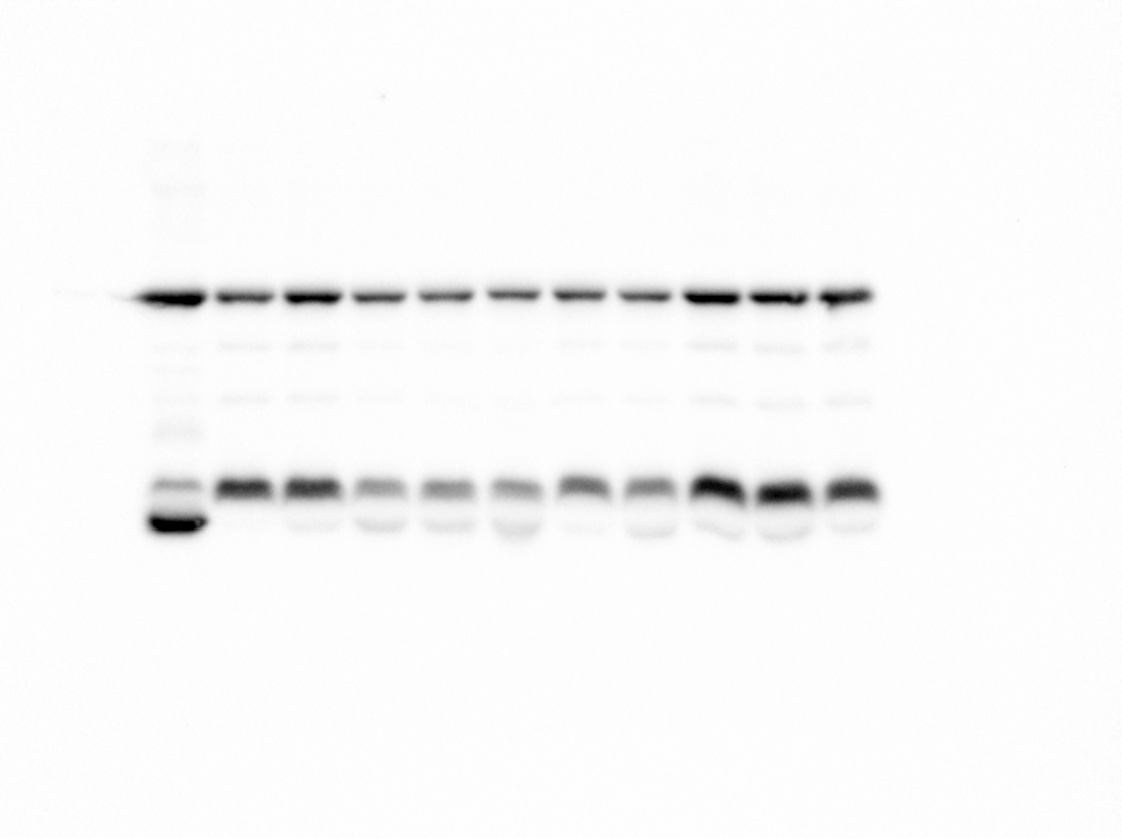

Supplement: Figure 2—source data 1. [file elife-74275-fig2-data1.zip › Figure 2-source data 1/Fig2 Blot2 Set1 anti-His.tif]

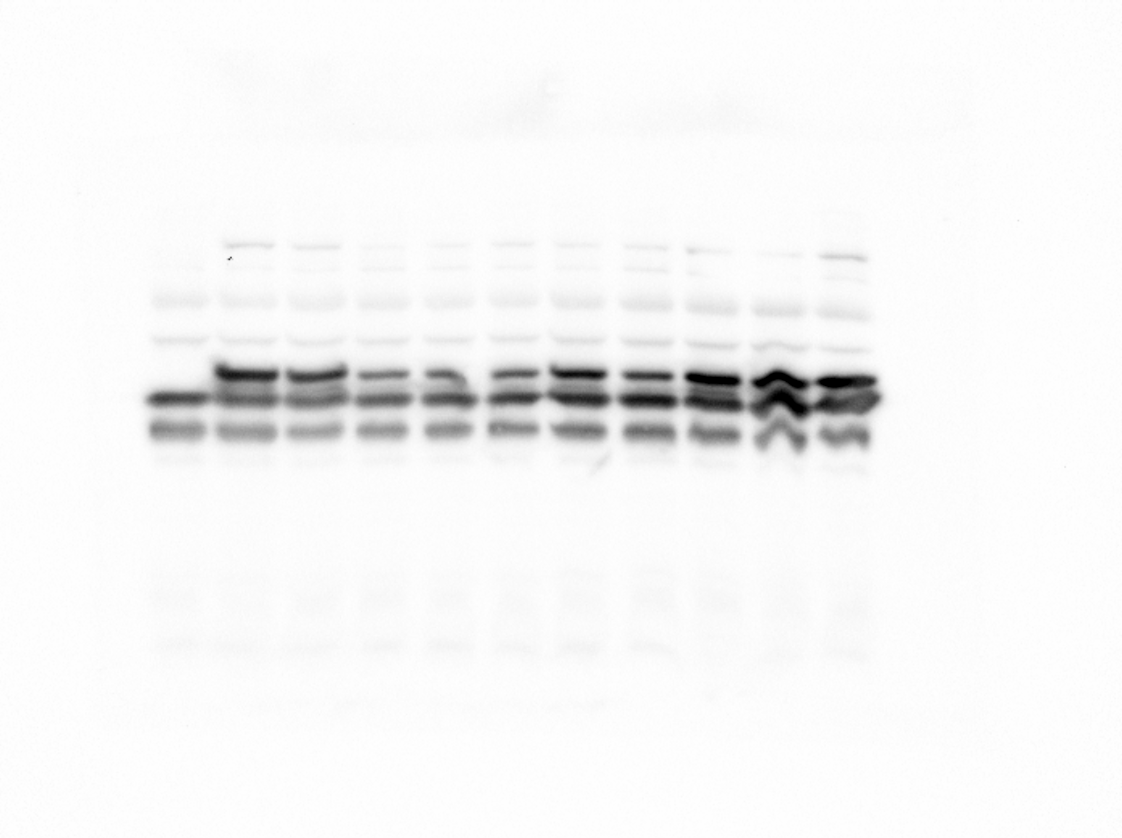

Supplement: Figure 2—source data 1. [file elife-74275-fig2-data1.zip › Figure 2-source data 1/Fig2 Blot2 Set1 anti-IVFA.tif]

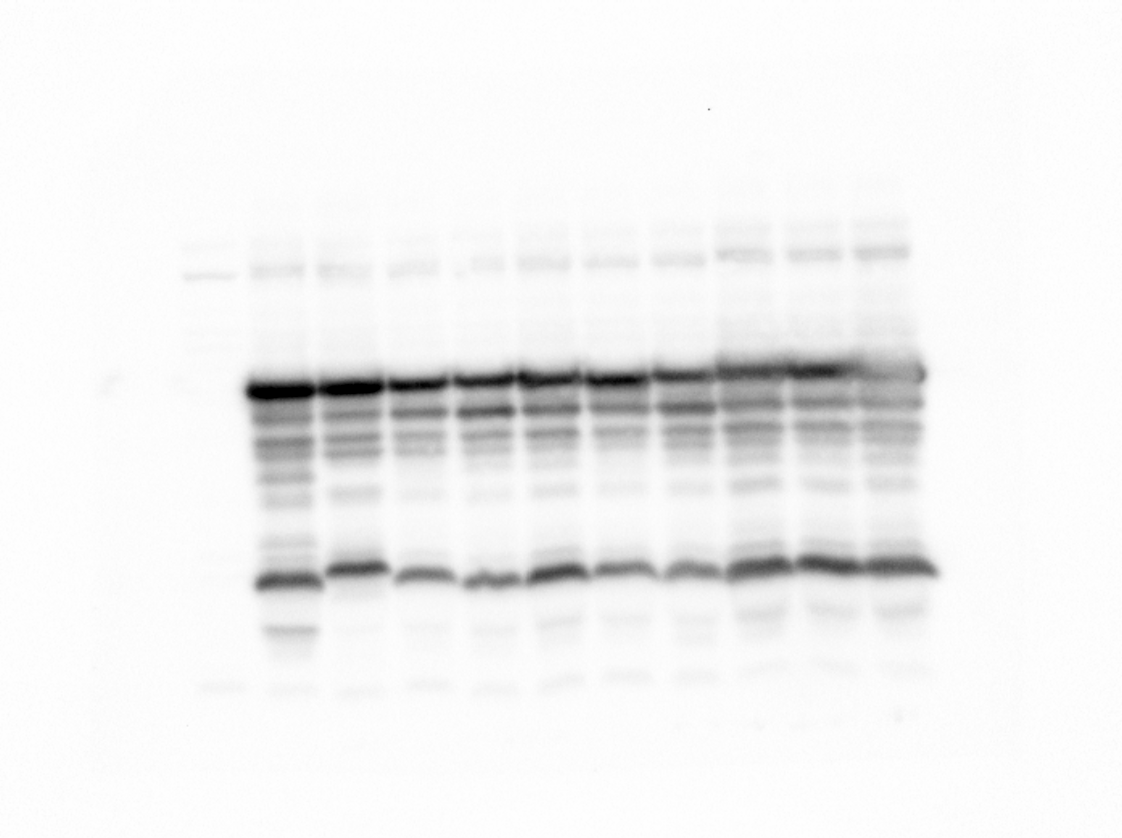

Supplement: Figure 2—source data 1. [file elife-74275-fig2-data1.zip › Figure 2-source data 1/Fig2 Blot2 Set2 anti-GFP.tif]

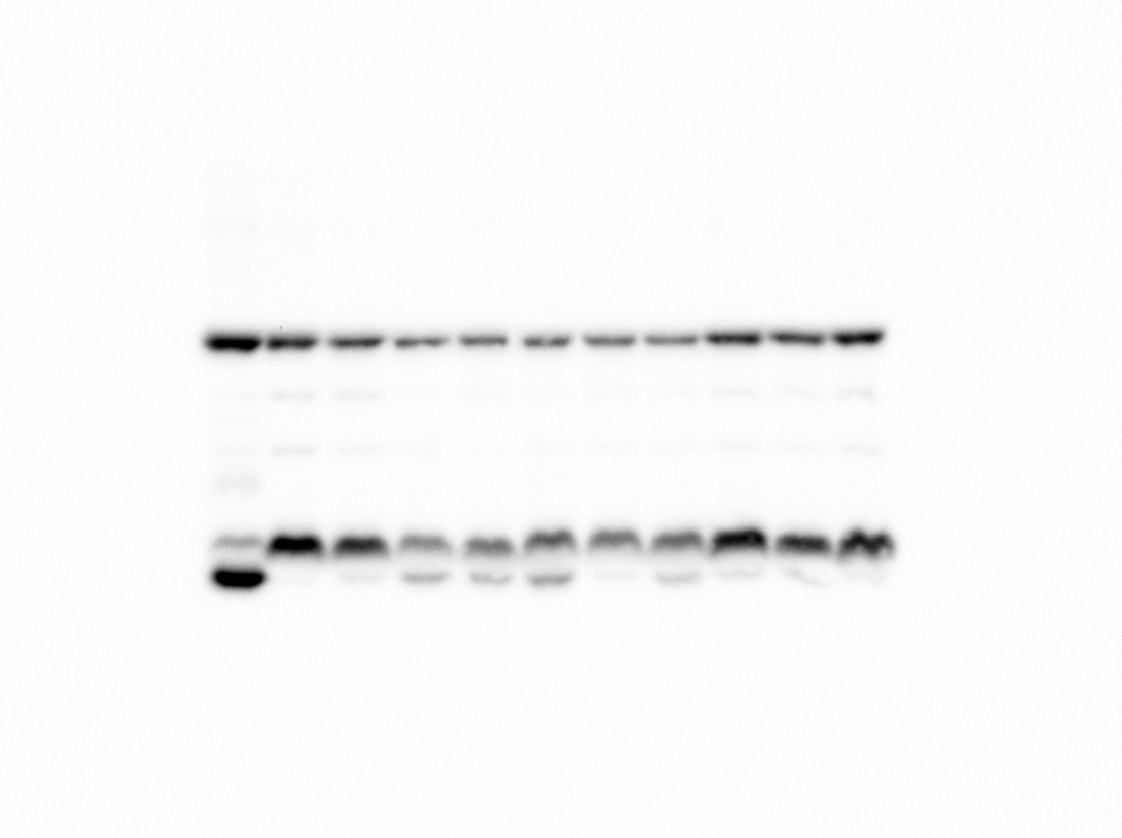

Supplement: Figure 2—source data 1. [file elife-74275-fig2-data1.zip › Figure 2-source data 1/Fig2 Blot2 Set2 anti-His.tif]

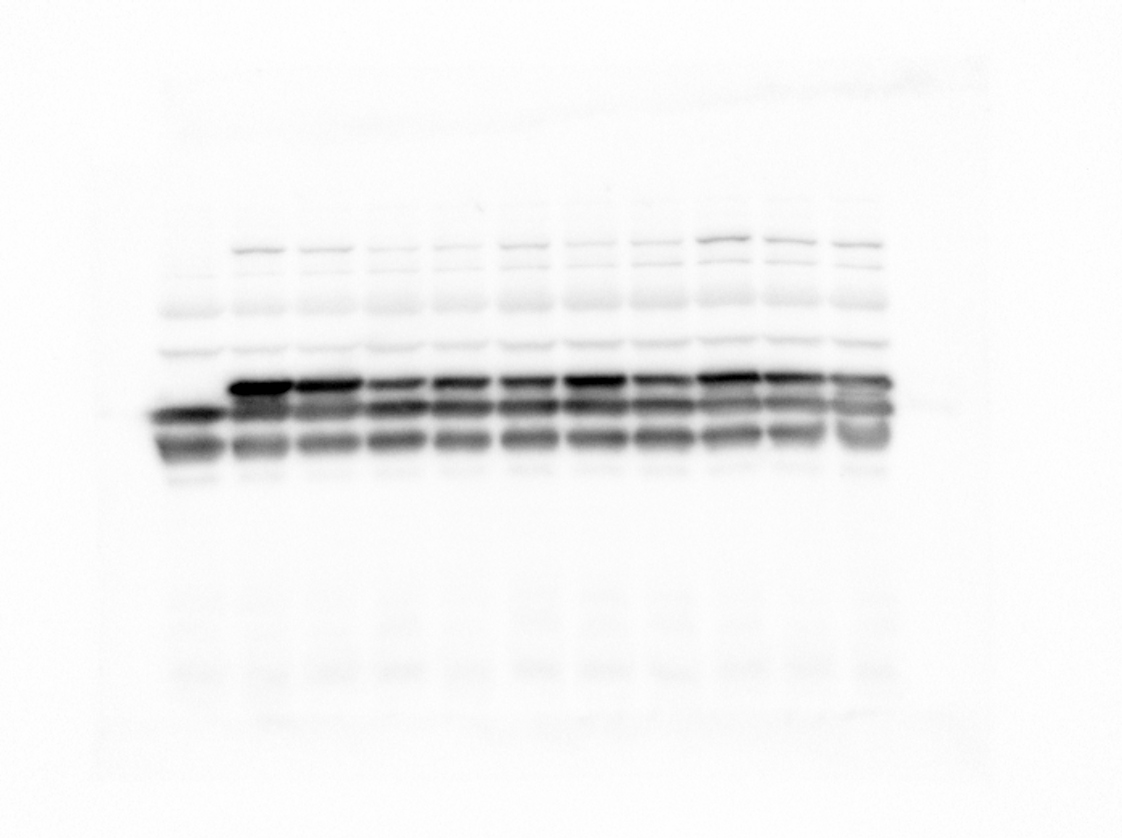

Supplement: Figure 2—source data 1. [file elife-74275-fig2-data1.zip › Figure 2-source data 1/Fig2 Blot2 Set2 anti-IVFA.tif]

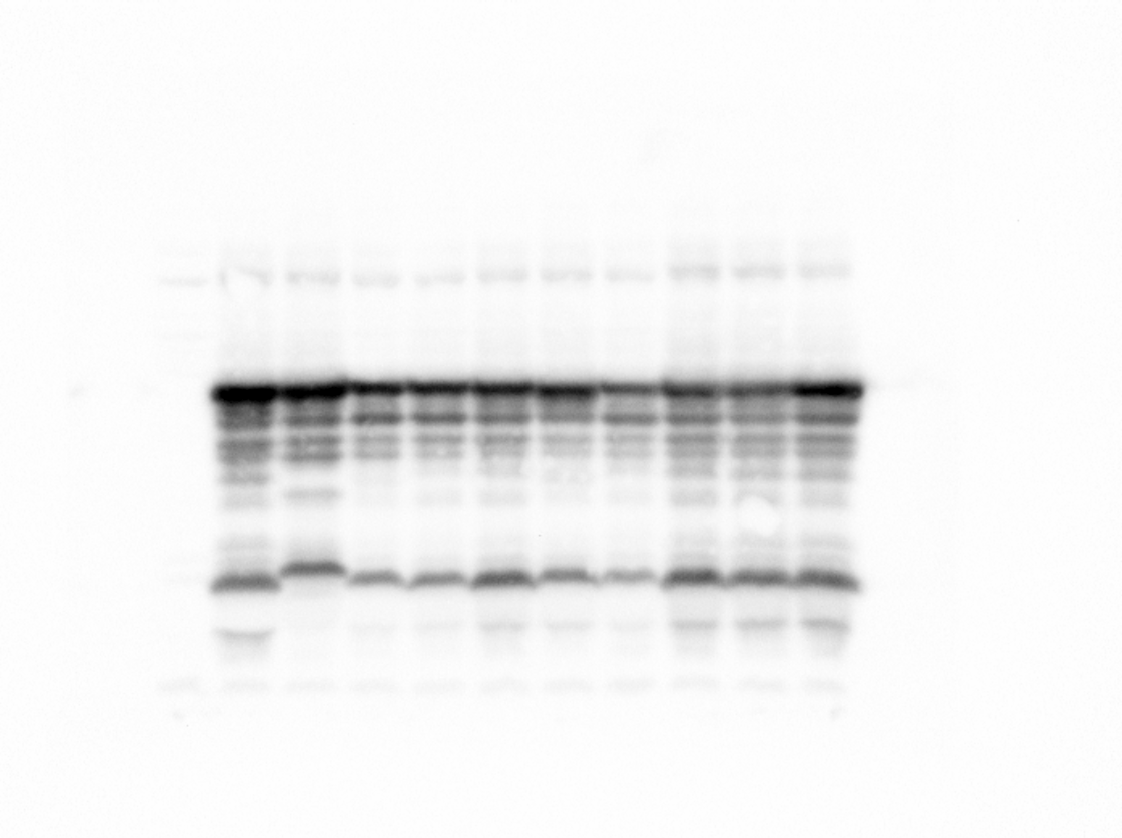

Supplement: Figure 2—source data 1. [file elife-74275-fig2-data1.zip › Figure 2-source data 1/Fig2 Blot2 Set3 anti-GFP.tif]

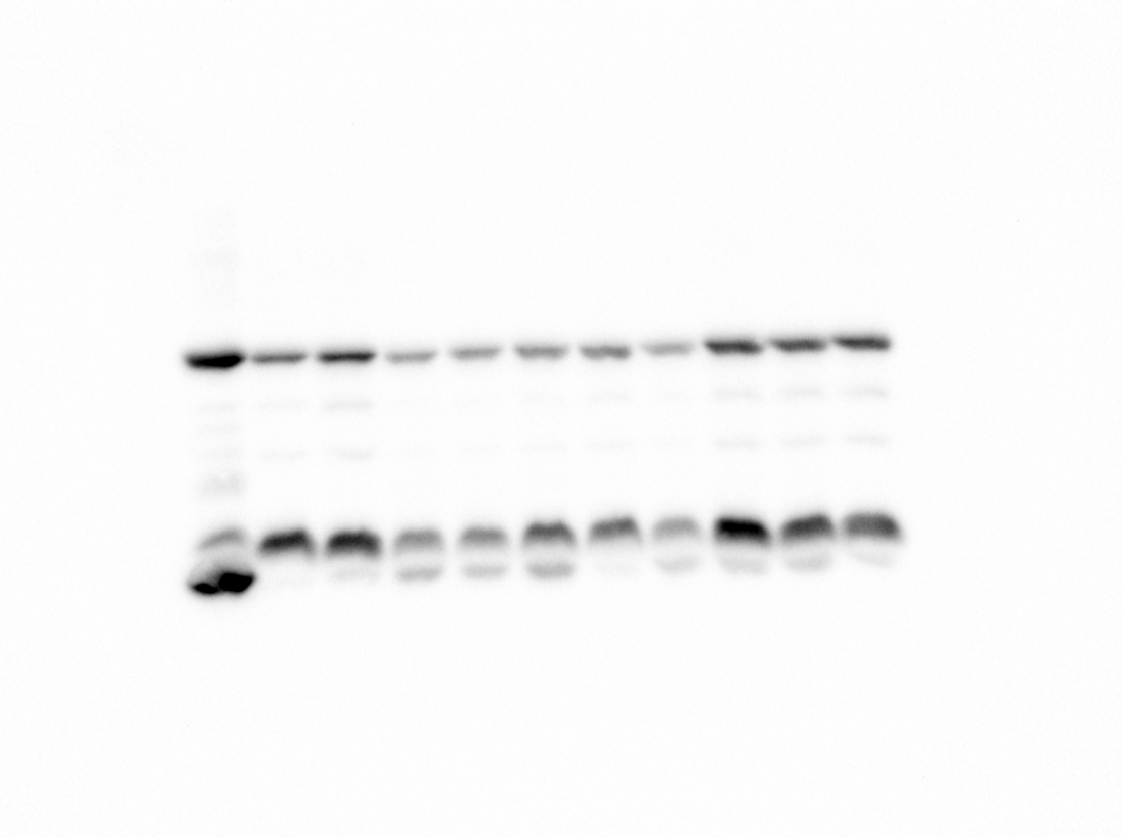

Supplement: Figure 2—source data 1. [file elife-74275-fig2-data1.zip › Figure 2-source data 1/Fig2 Blot2 Set3 anti-His.tif]

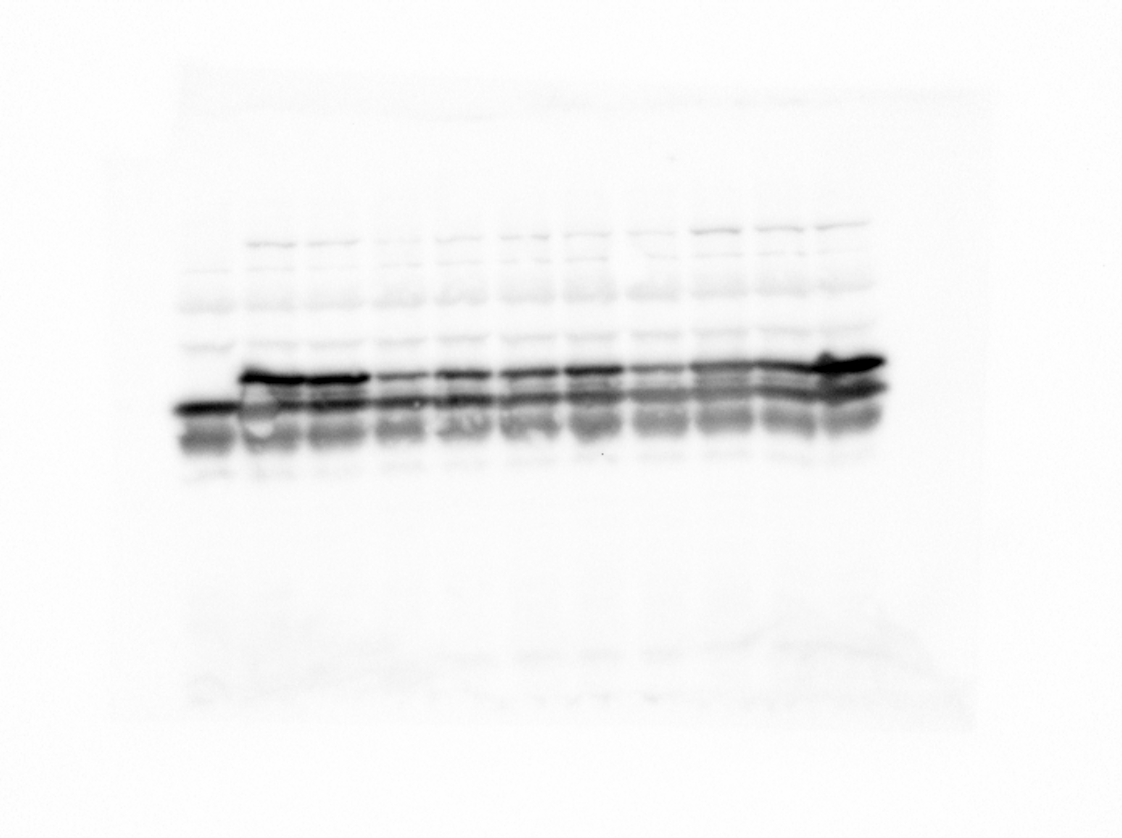

Supplement: Figure 2—source data 1. [file elife-74275-fig2-data1.zip › Figure 2-source data 1/Fig2 Blot2 Set3 anti-IVFA.tif]

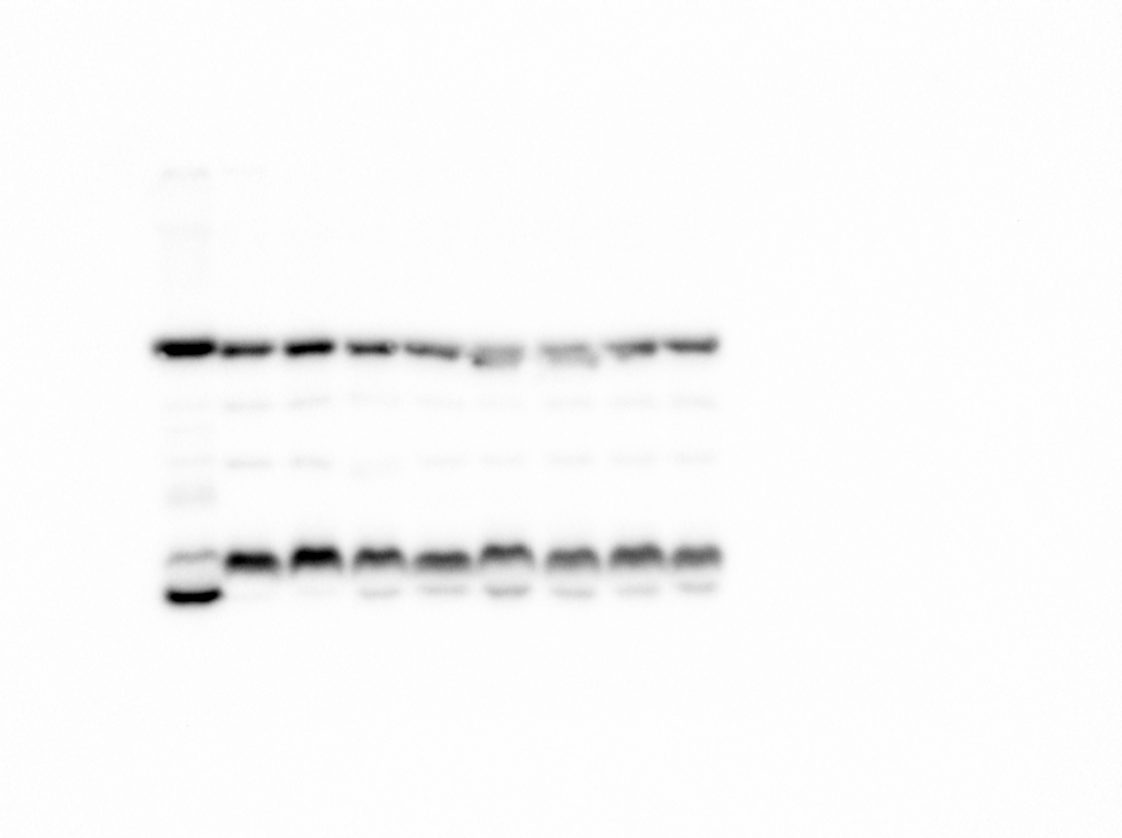

Supplement: Figure 2—source data 1. [file elife-74275-fig2-data1.zip › Figure 2-source data 1/Fig2 Redo1 anti-His.tif]

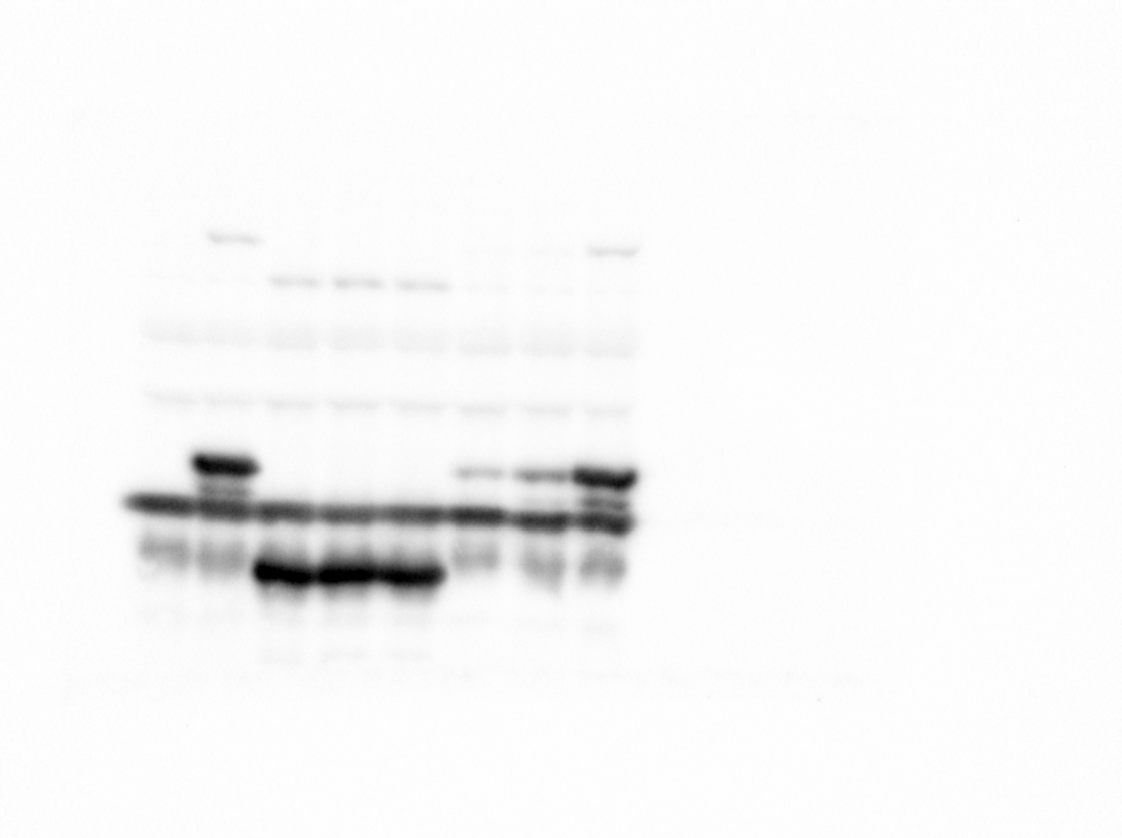

Supplement: Figure 2—source data 1. [file elife-74275-fig2-data1.zip › Figure 2-source data 1/Fig2 Redo2 anti-IVFA.tif]

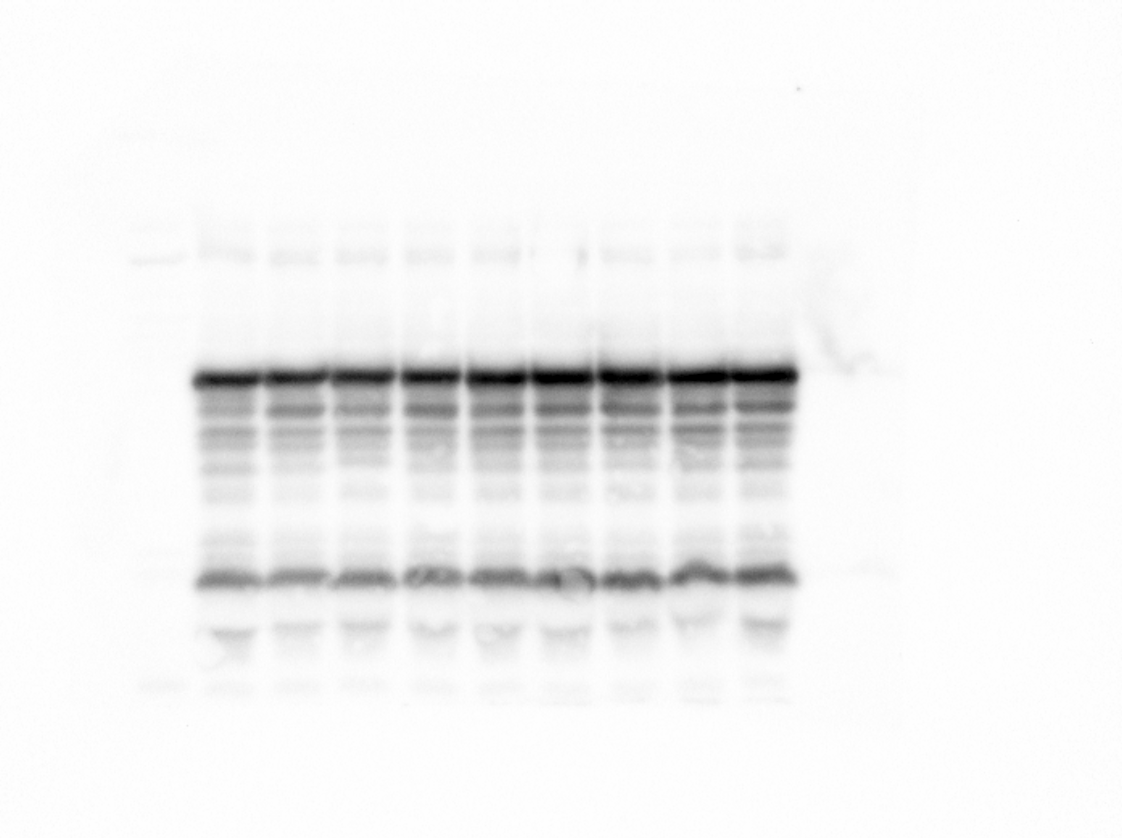

Supplement: Figure 2—source data 1. [file elife-74275-fig2-data1.zip › Figure 2-source data 1/Fig2 Redo3 anti-GFP.tif]

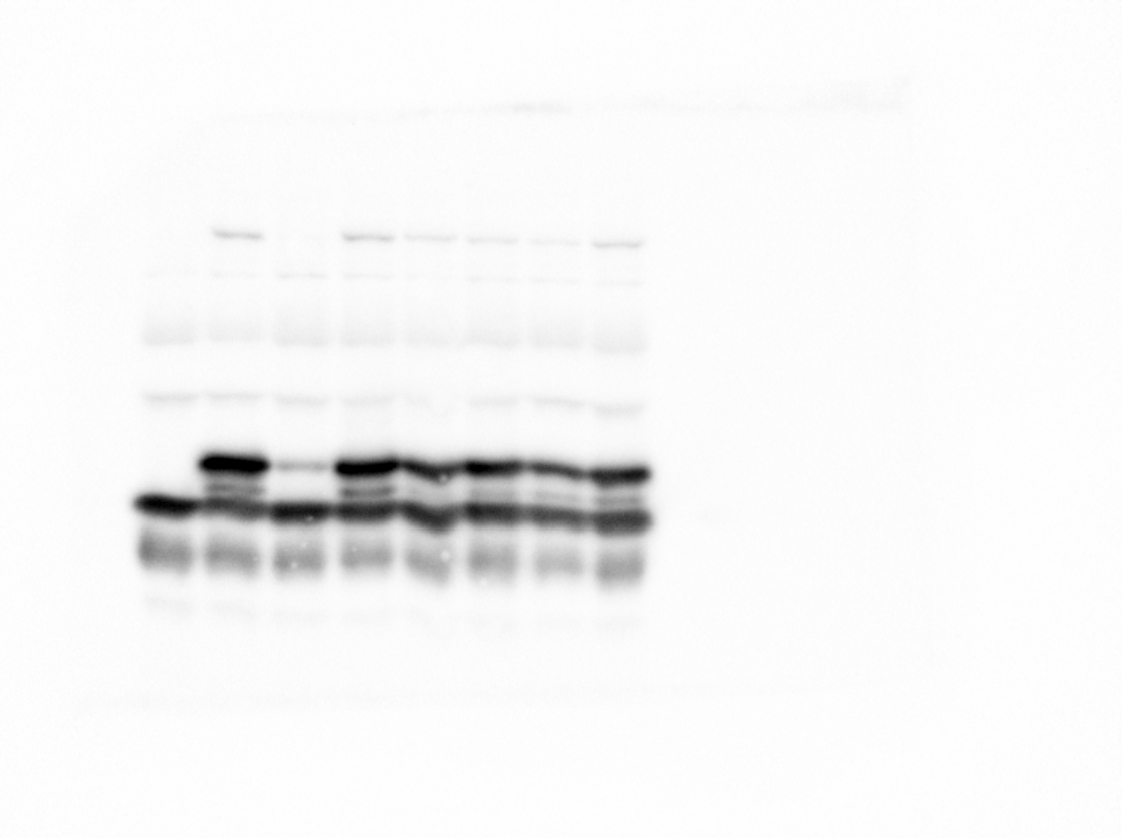

Supplement: Figure 2—source data 1. [file elife-74275-fig2-data1.zip › Figure 2-source data 1/Fig2 Redo4 anti-IVFA.tif]

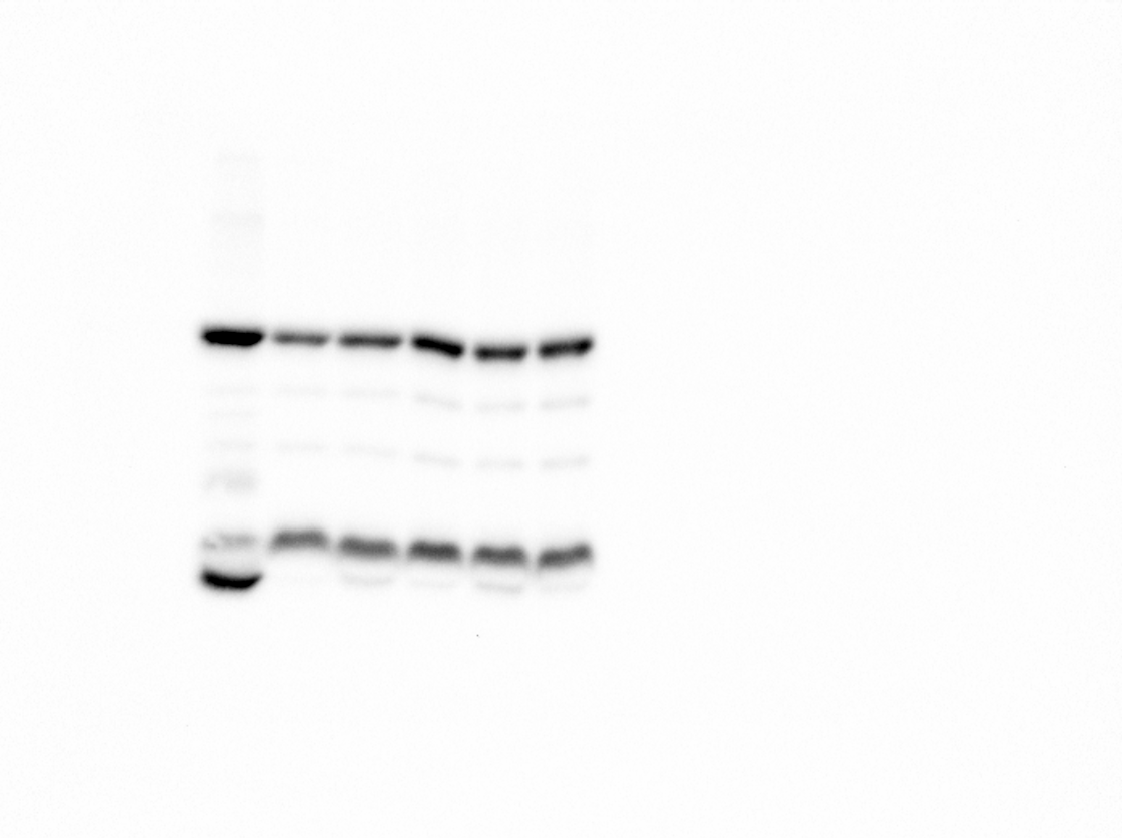

Supplement: Figure 2—source data 1. [file elife-74275-fig2-data1.zip › Figure 2-source data 1/Fig2 Redo5 anti-His.tif]

## Slide 1
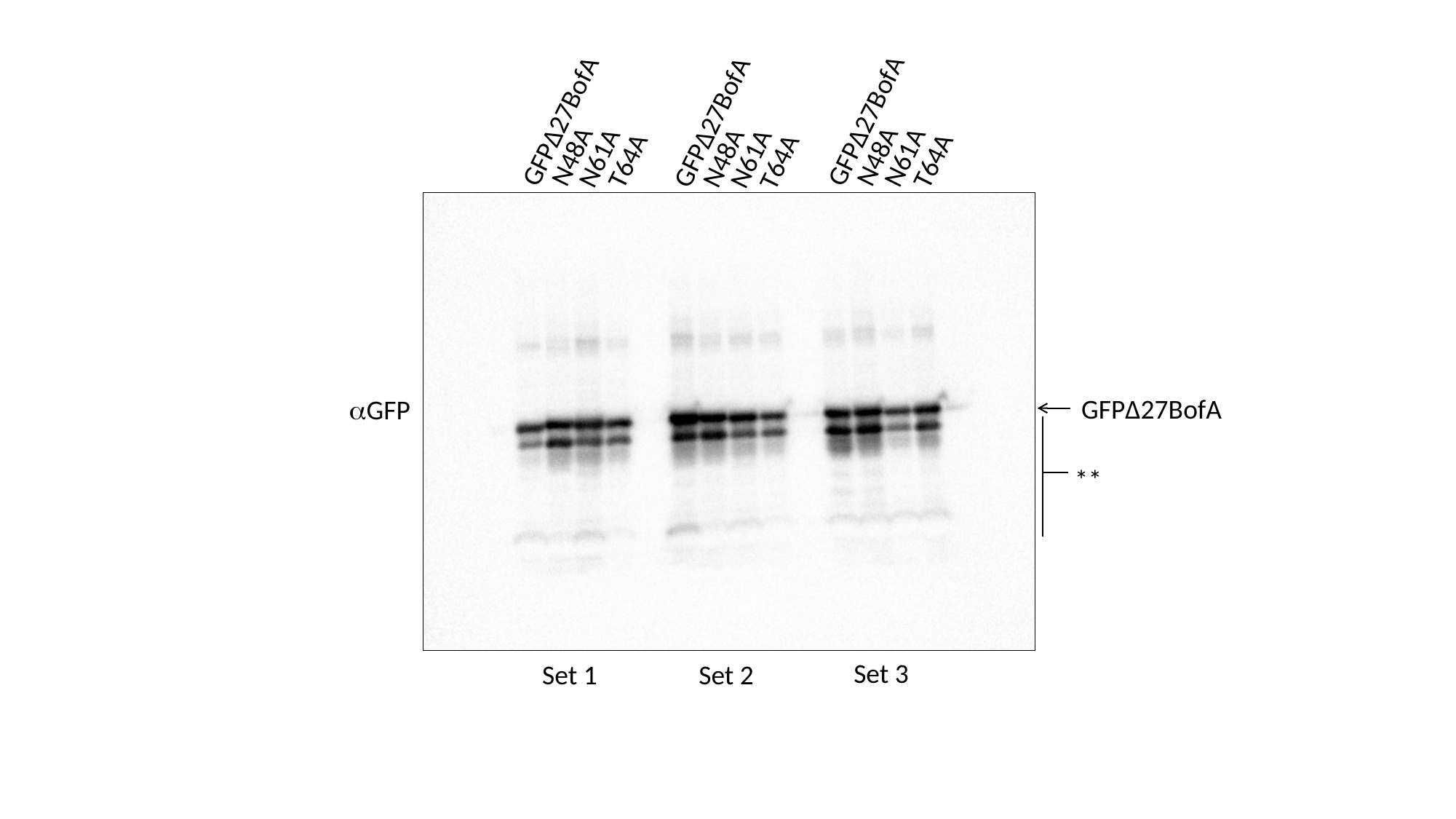

GFPΔ27BofA
GFPΔ27BofA
GFPΔ27BofA
N48A
N48A
N61A
N48A
N61A
N61A
T64A
T64A
T64A
GFPΔ27BofA
aGFP
**
Set 3
Set 1
Set 2

Supplement: Figure 2—figure supplement 2—source data 1. [file elife-74275-fig2-figsupp2-data1.zip › Figure 2-figure supplement 2-source data 1/fig sup 2 annotated blot.pptx]

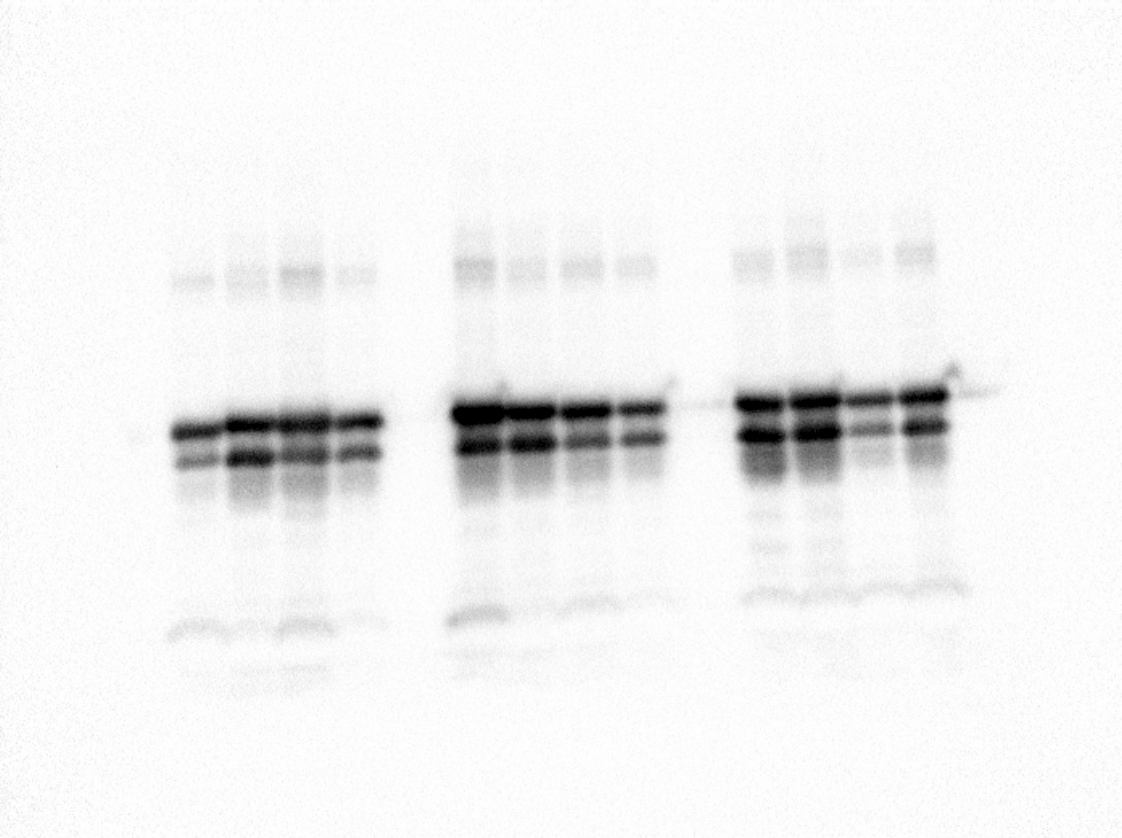

Supplement: Figure 2—figure supplement 2—source data 1. [file elife-74275-fig2-figsupp2-data1.zip › Figure 2-figure supplement 2-source data 1/fig sup 2 anti-GFP.tif]

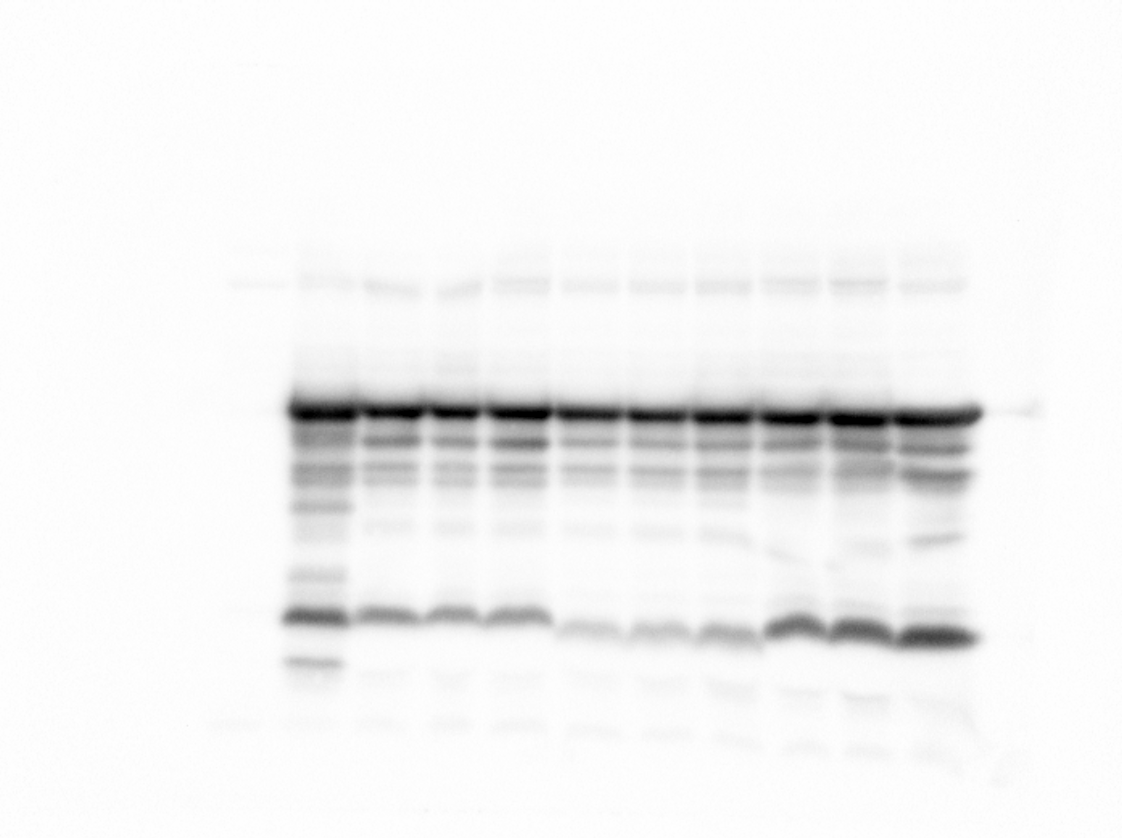

Supplement: Figure 2—figure supplement 3—source data 1. [file elife-74275-fig2-figsupp3-data1.zip › Figure 2-figure supplement 3-source data 1/fig sup 3 anti-GFP.tif]

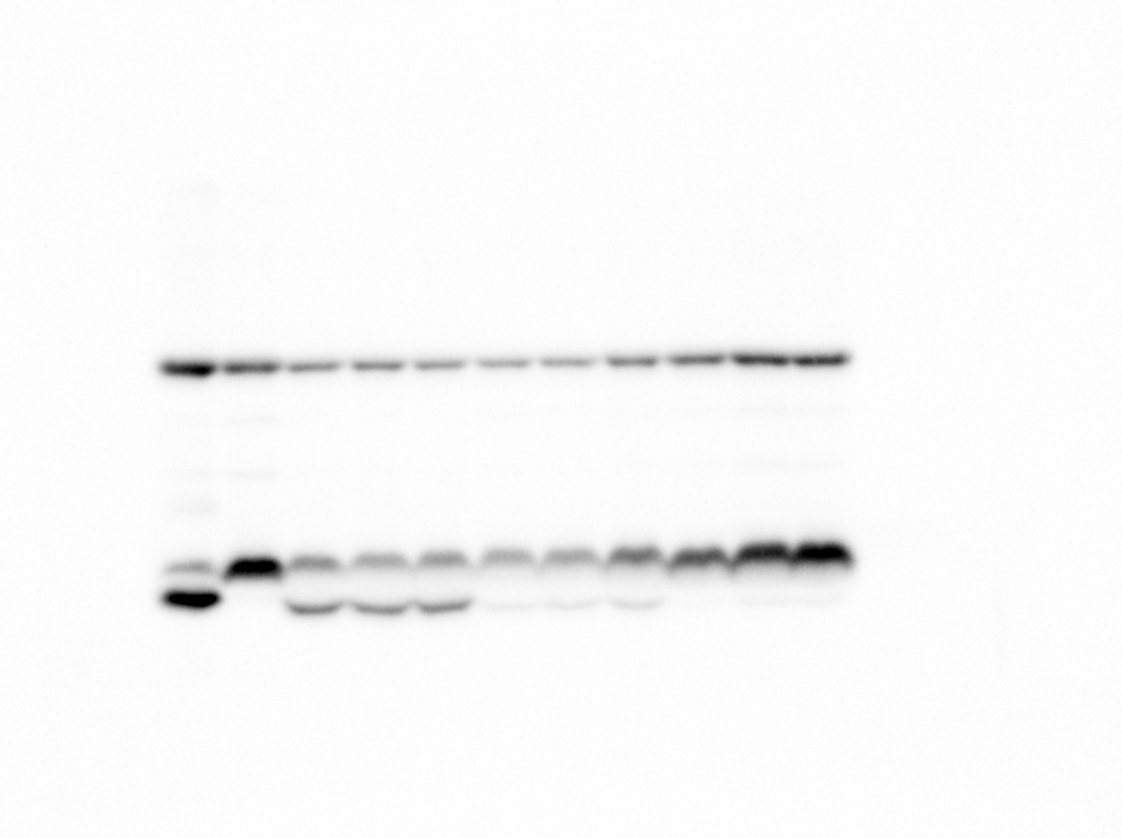

Supplement: Figure 2—figure supplement 3—source data 1. [file elife-74275-fig2-figsupp3-data1.zip › Figure 2-figure supplement 3-source data 1/fig sup 3 anti-His.tif]

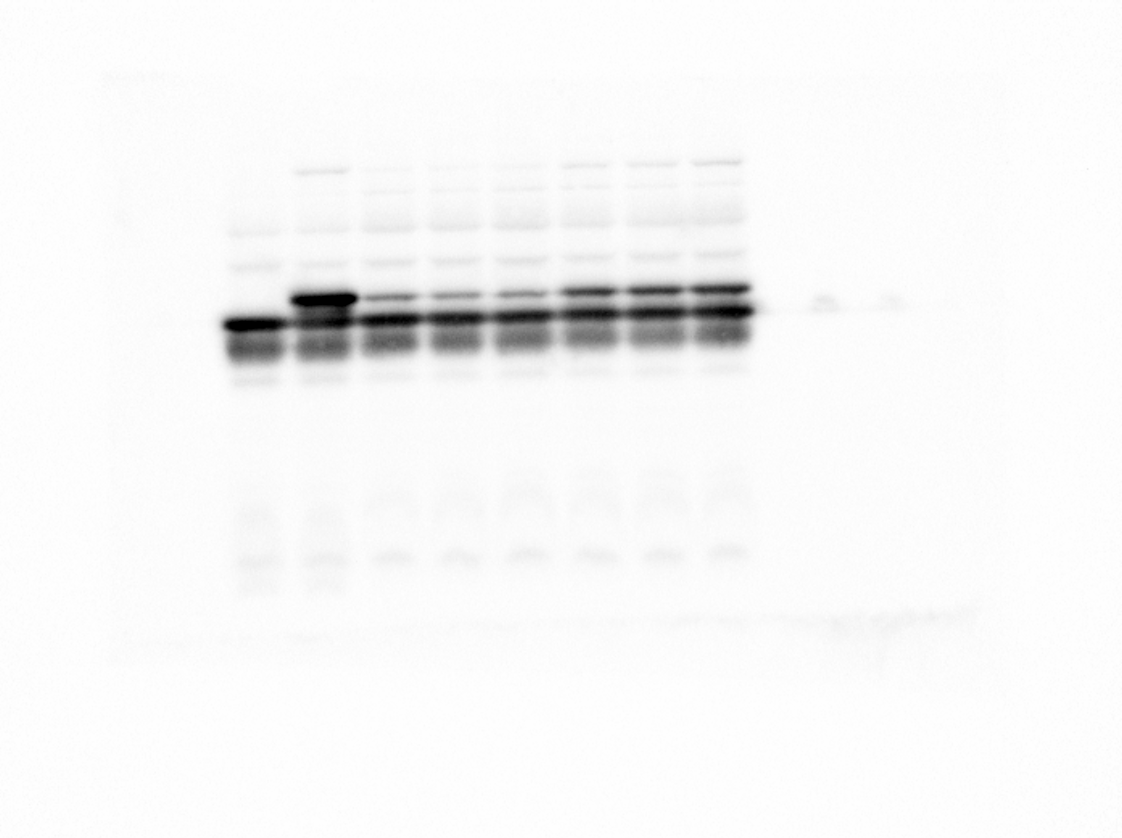

Supplement: Figure 2—figure supplement 3—source data 1. [file elife-74275-fig2-figsupp3-data1.zip › Figure 2-figure supplement 3-source data 1/fig sup 3 anti-IVFA.tif]

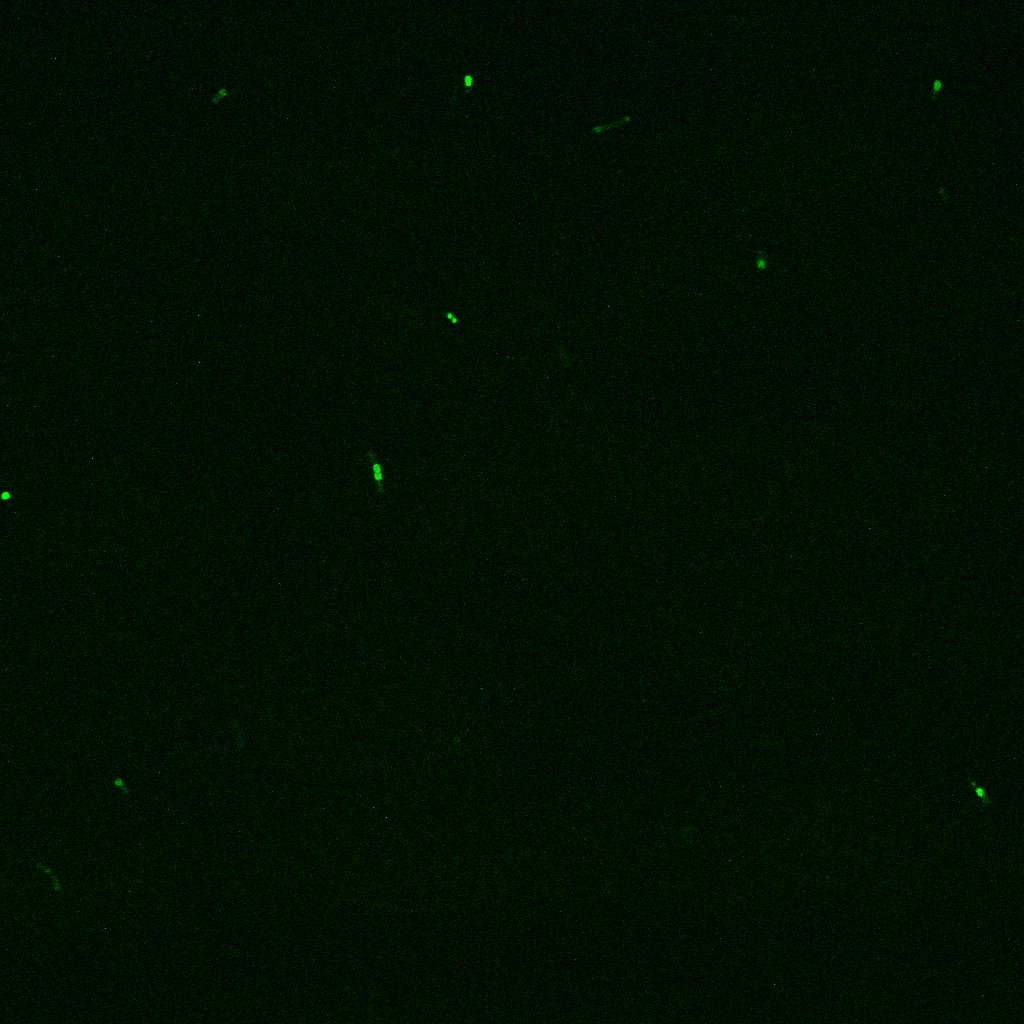

Supplement: Figure 3—source data 1. [file elife-74275-fig3-data1.zip › Figure 3-source data 1/Figure 3B images/GFPdelta27BofA 3 hrs 1 GFP.jpg]

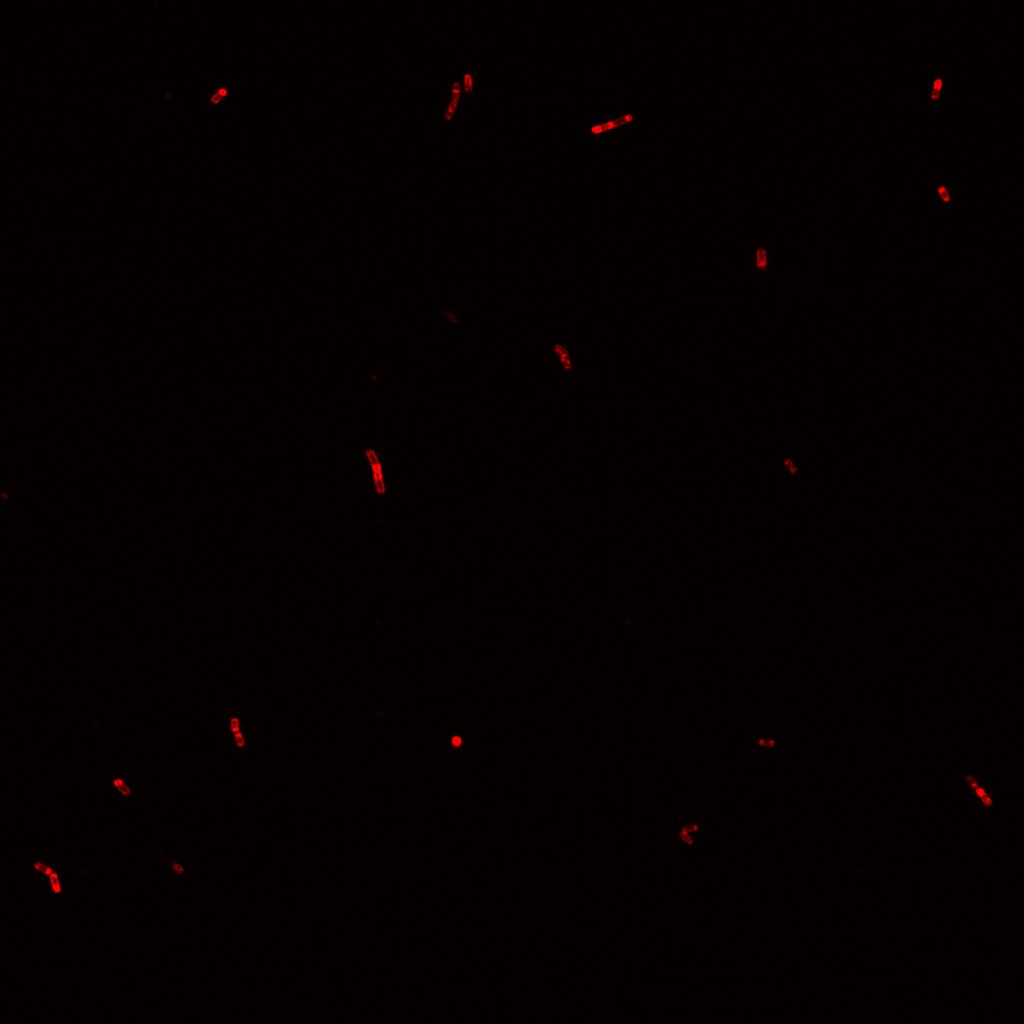

Supplement: Figure 3—source data 1. [file elife-74275-fig3-data1.zip › Figure 3-source data 1/Figure 3B images/GFPdelta27BofA 3 hrs 1 membrane.jpg]

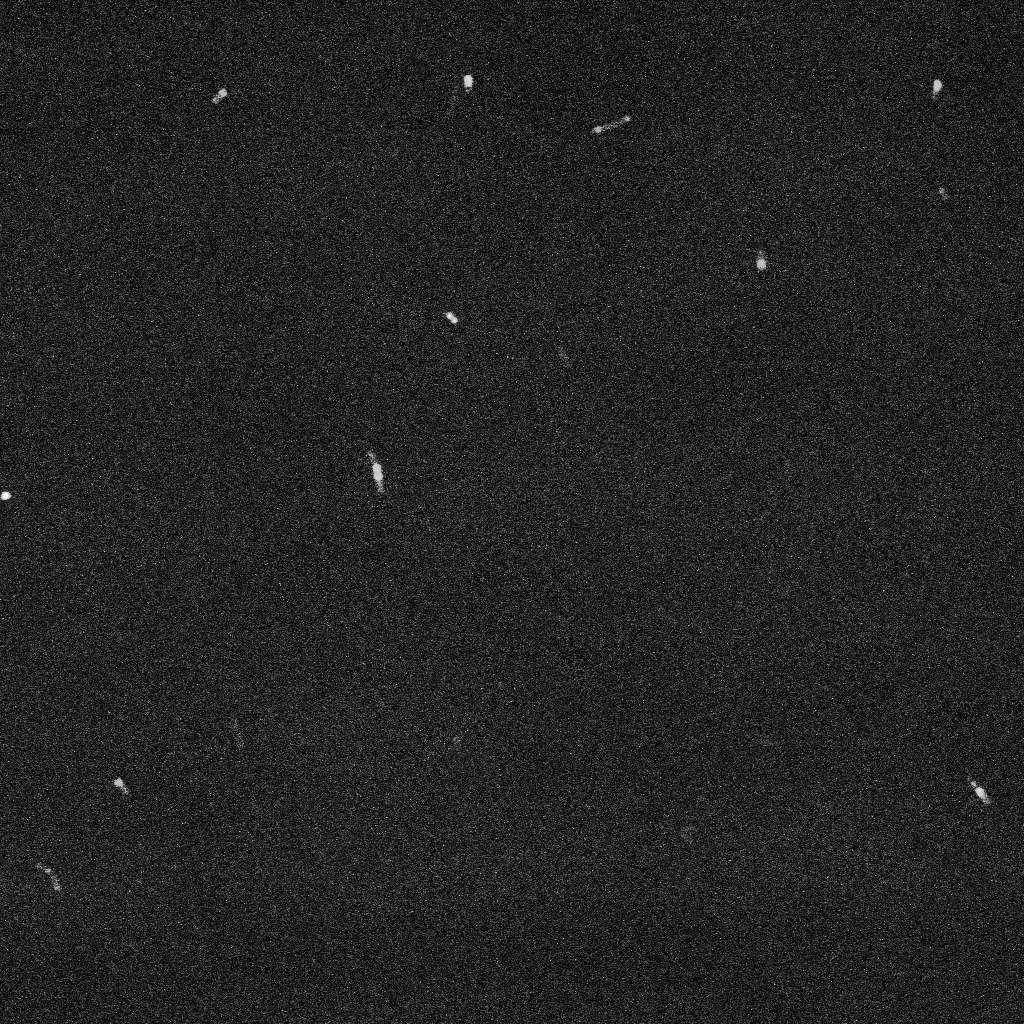

Supplement: Figure 3—source data 1. [file elife-74275-fig3-data1.zip › Figure 3-source data 1/Figure 3B images/GFPdelta27BofA 3 hrs 1.tif]

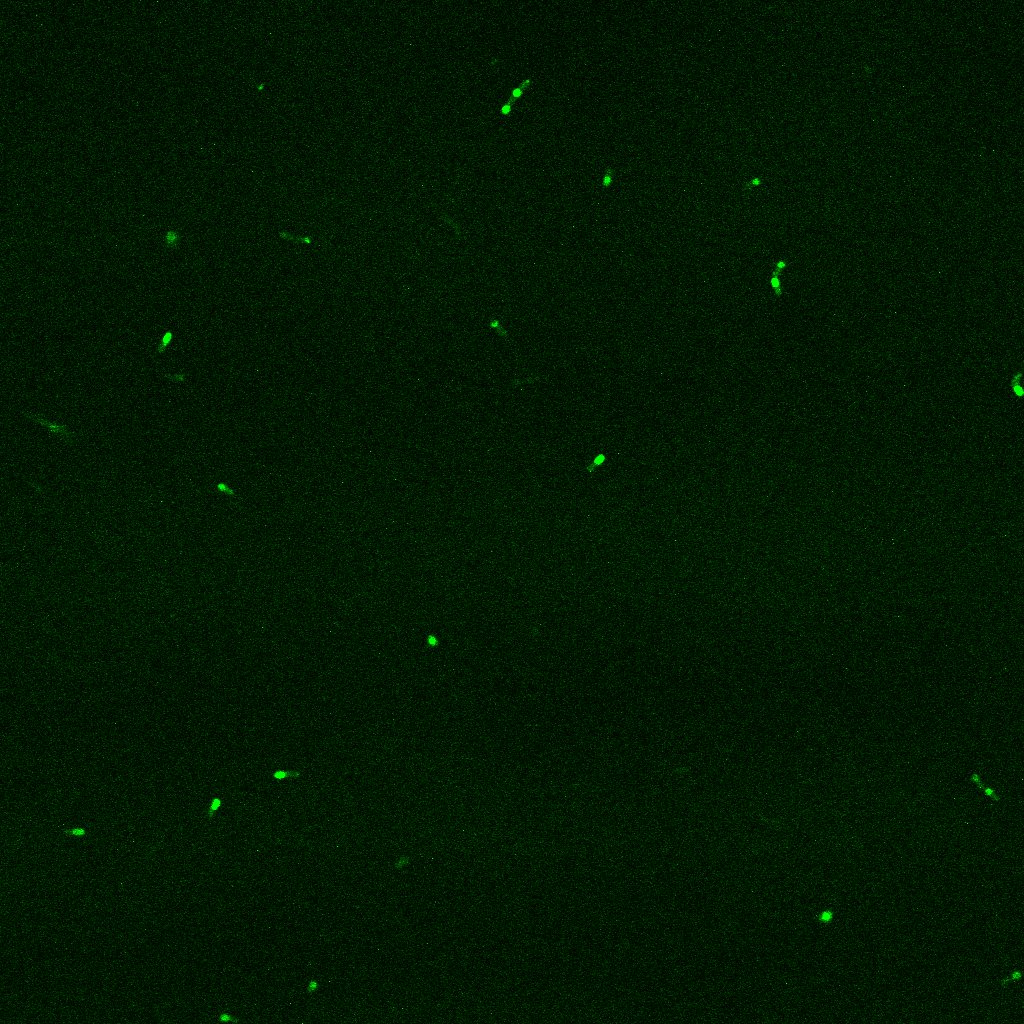

Supplement: Figure 3—source data 1. [file elife-74275-fig3-data1.zip › Figure 3-source data 1/Figure 3B images/GFPdelta27BofA 3 hrs 2 GFP.jpg]

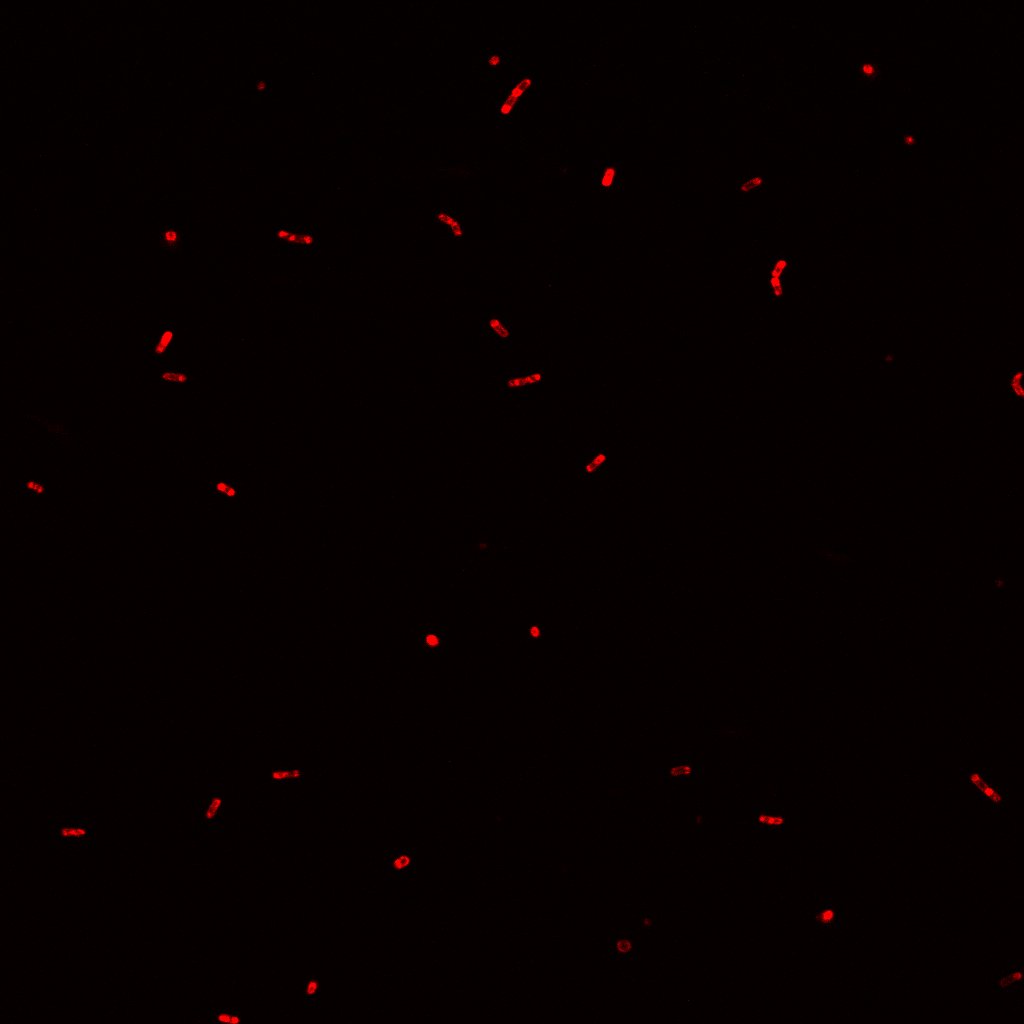

Supplement: Figure 3—source data 1. [file elife-74275-fig3-data1.zip › Figure 3-source data 1/Figure 3B images/GFPdelta27BofA 3 hrs 2 membrane.jpg]

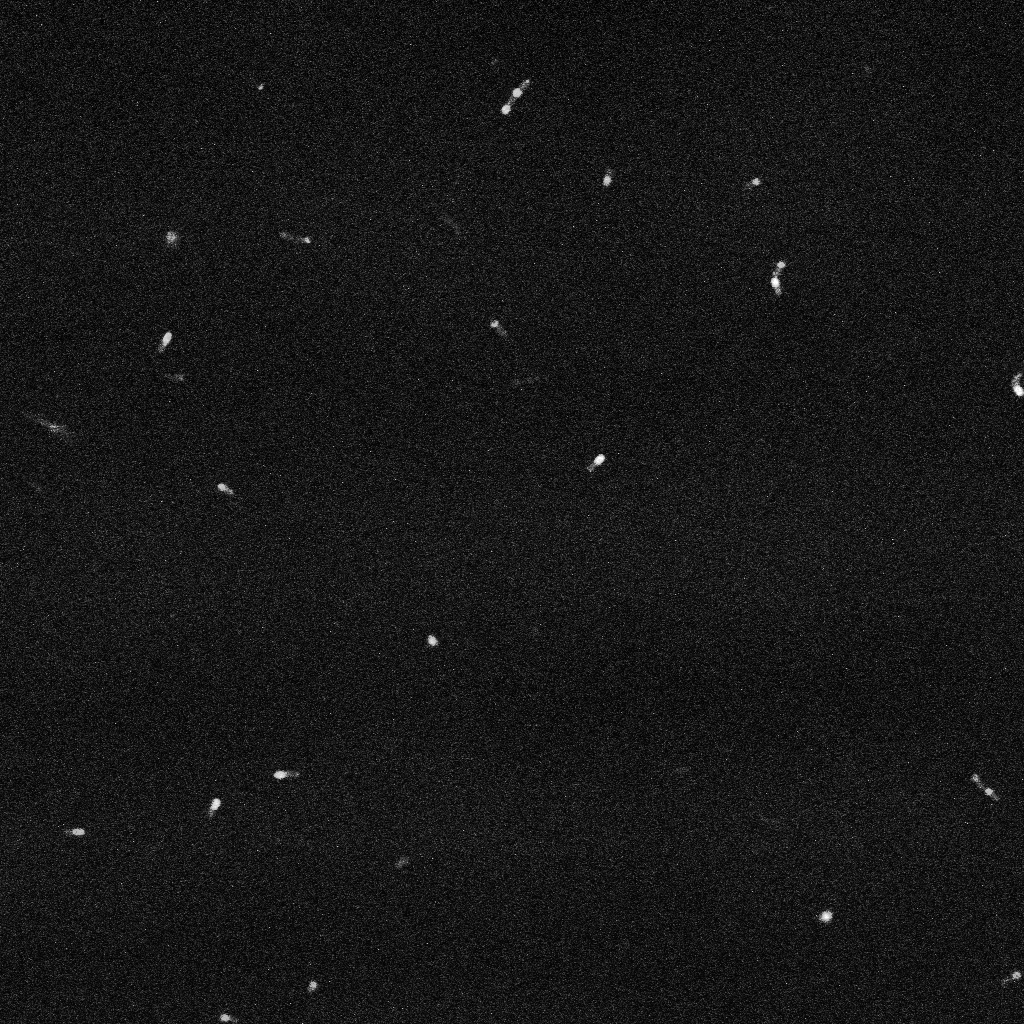

Supplement: Figure 3—source data 1. [file elife-74275-fig3-data1.zip › Figure 3-source data 1/Figure 3B images/GFPdelta27BofA 3 hrs 2.tif]

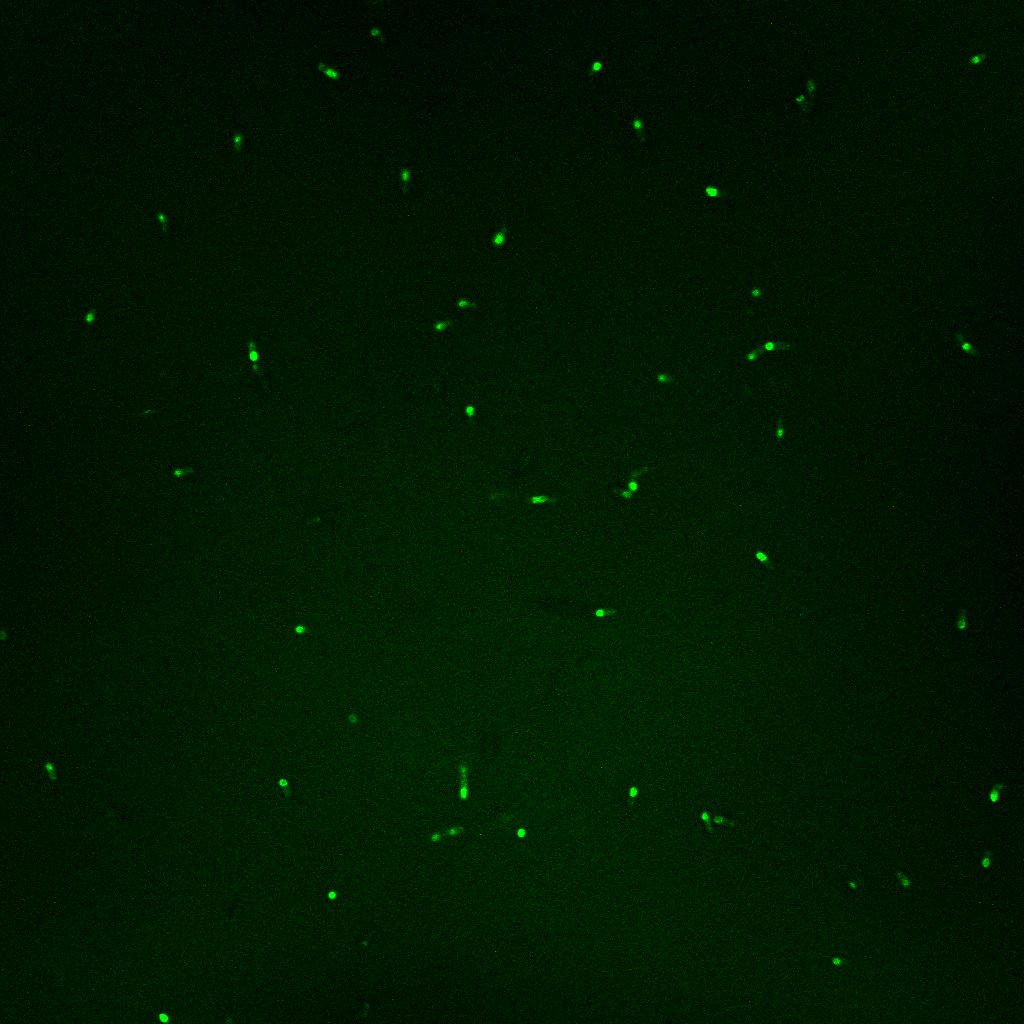

Supplement: Figure 3—source data 1. [file elife-74275-fig3-data1.zip › Figure 3-source data 1/Figure 3B images/GFPdelta27BofA 3 hrs 3 GFP.jpg]

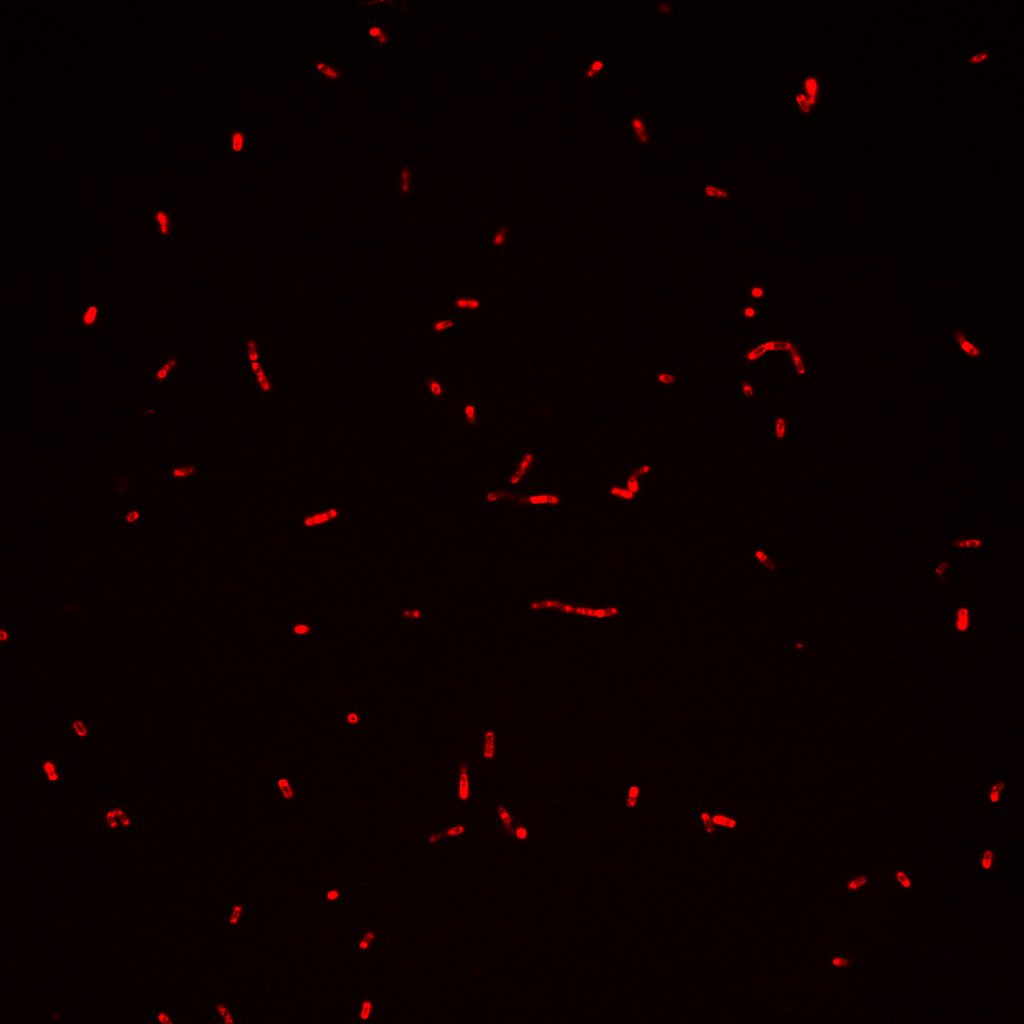

Supplement: Figure 3—source data 1. [file elife-74275-fig3-data1.zip › Figure 3-source data 1/Figure 3B images/GFPdelta27BofA 3 hrs 3 membrane.jpg]

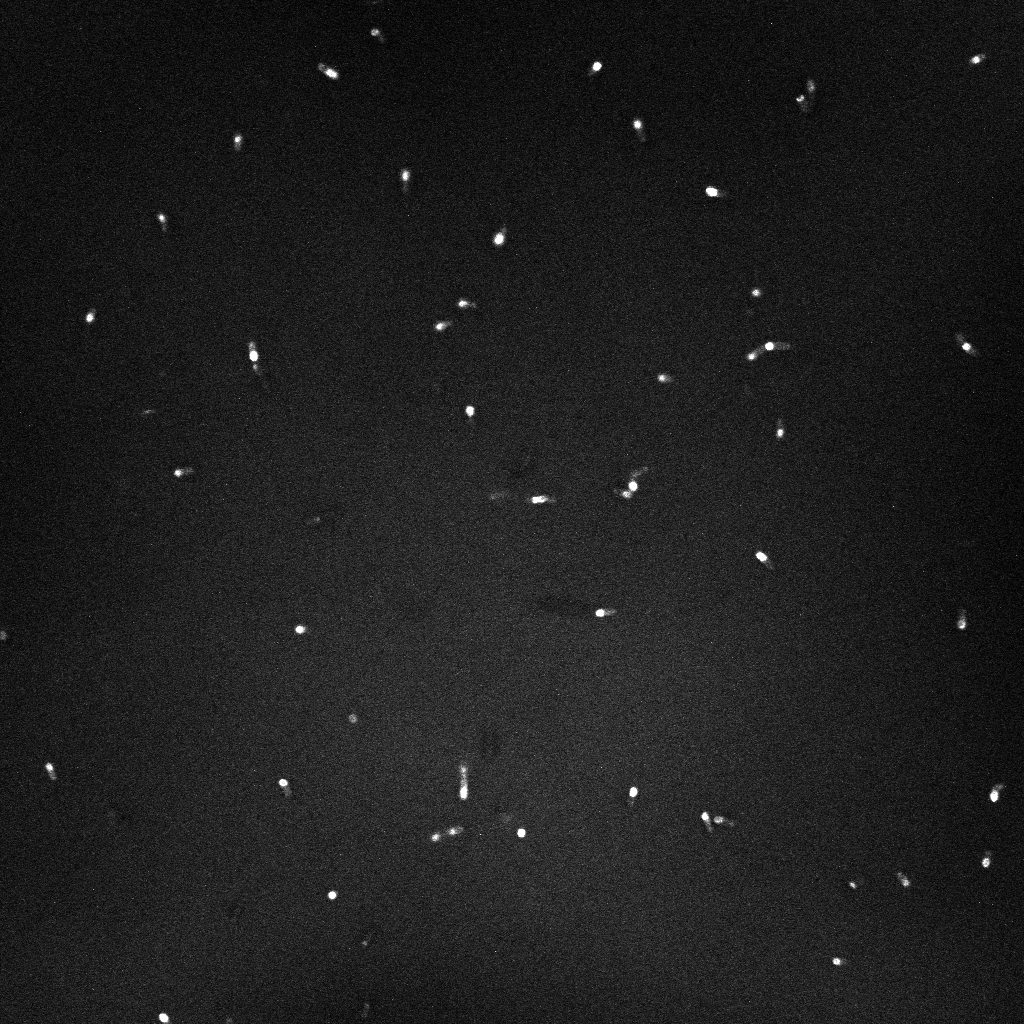

Supplement: Figure 3—source data 1. [file elife-74275-fig3-data1.zip › Figure 3-source data 1/Figure 3B images/GFPdelta27BofA 3 hrs 3.tif]

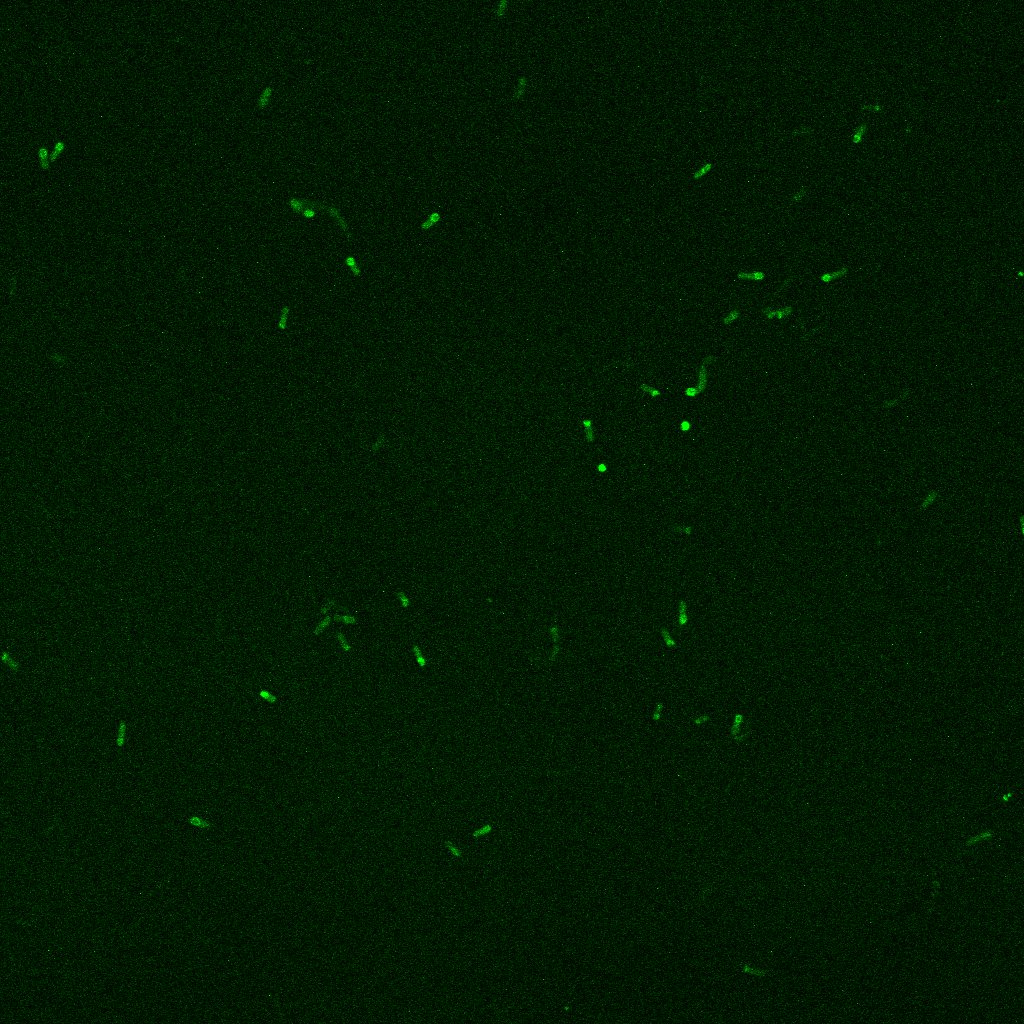

Supplement: Figure 3—source data 1. [file elife-74275-fig3-data1.zip › Figure 3-source data 1/Figure 3B images/N48A 3 hr 2 GFP.jpg]

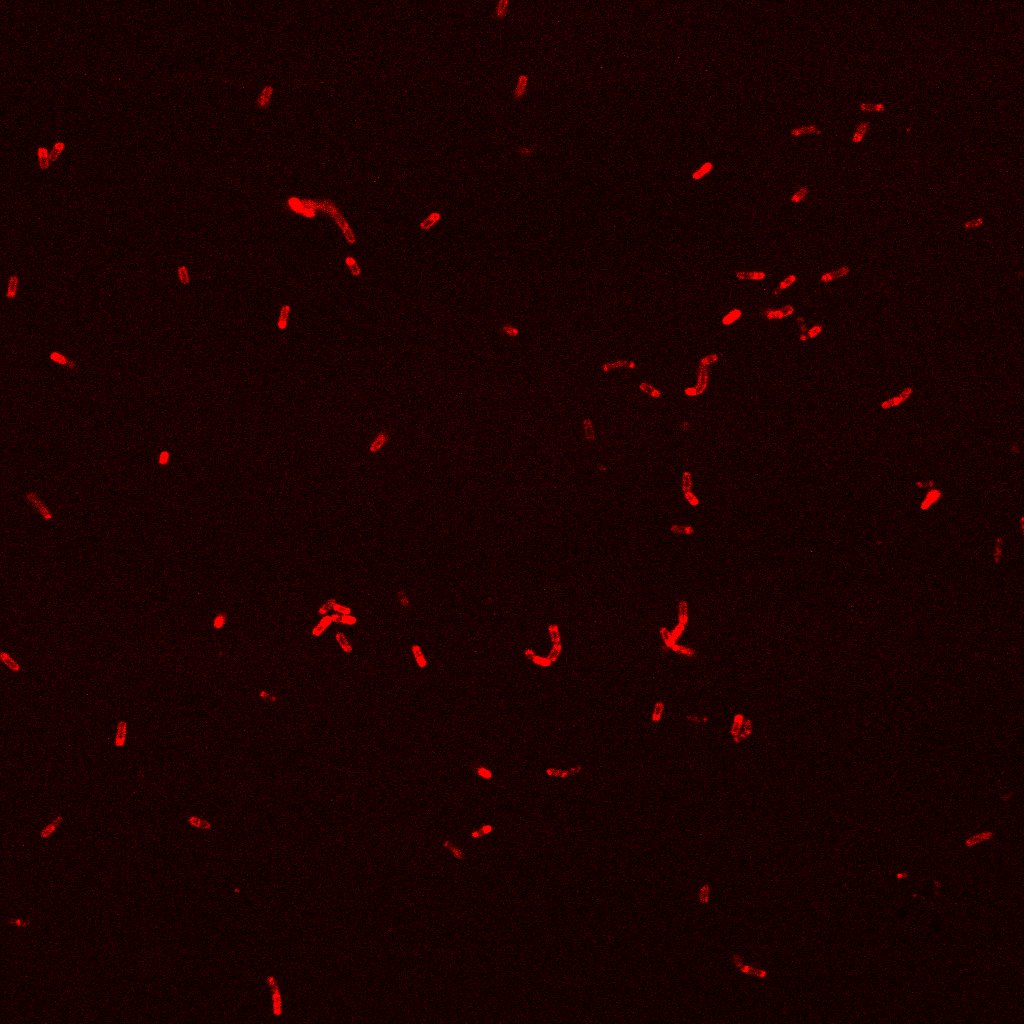

Supplement: Figure 3—source data 1. [file elife-74275-fig3-data1.zip › Figure 3-source data 1/Figure 3B images/N48A 3 hr 2 membrane.jpg]

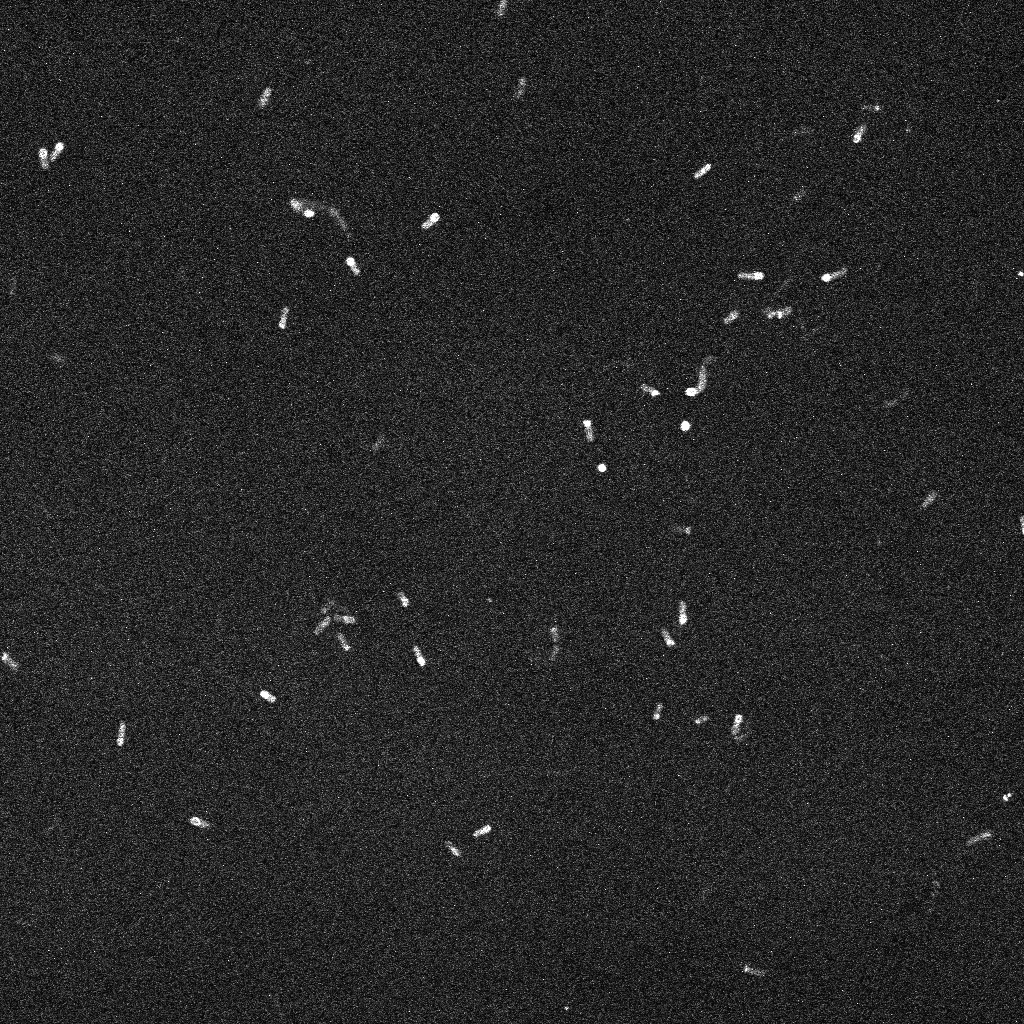

Supplement: Figure 3—source data 1. [file elife-74275-fig3-data1.zip › Figure 3-source data 1/Figure 3B images/N48A 3 hr 2.tif]

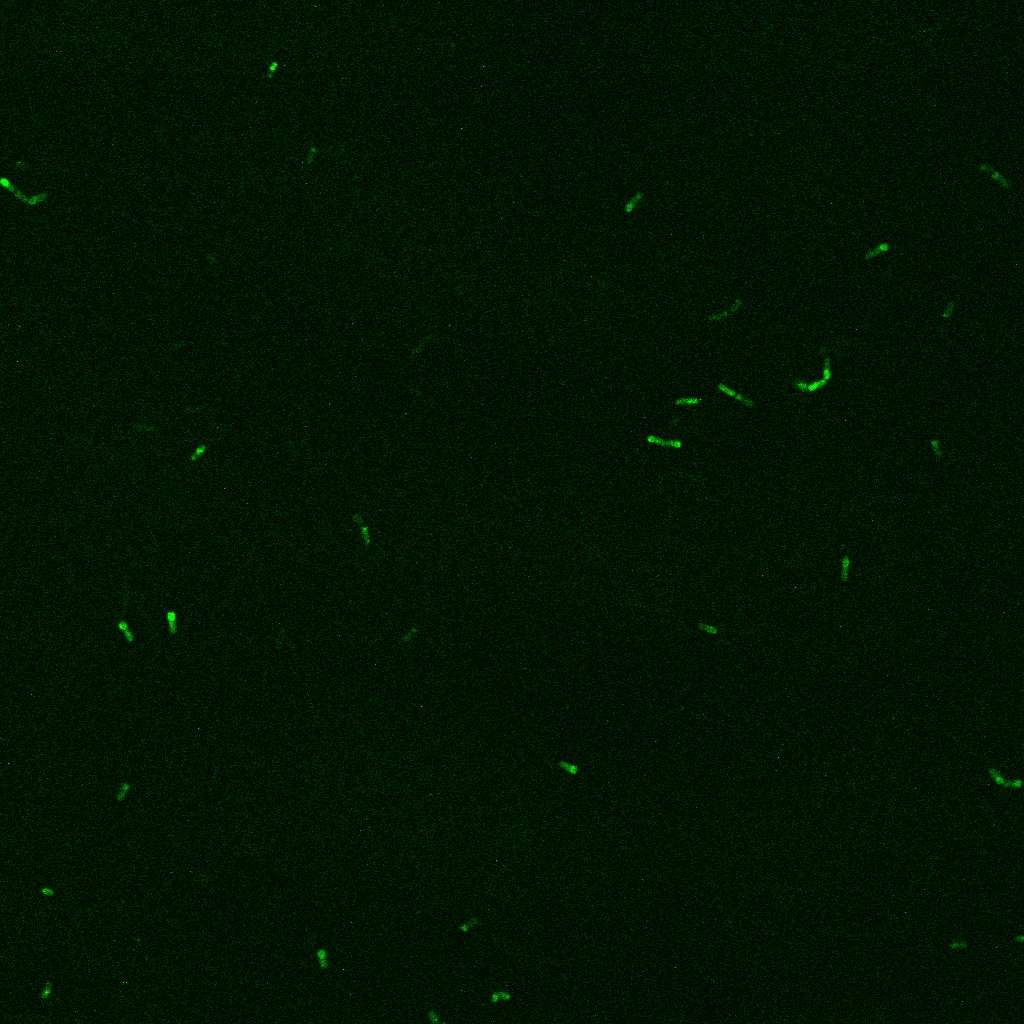

Supplement: Figure 3—source data 1. [file elife-74275-fig3-data1.zip › Figure 3-source data 1/Figure 3B images/N48A 3 hr 3 GFP.jpg]

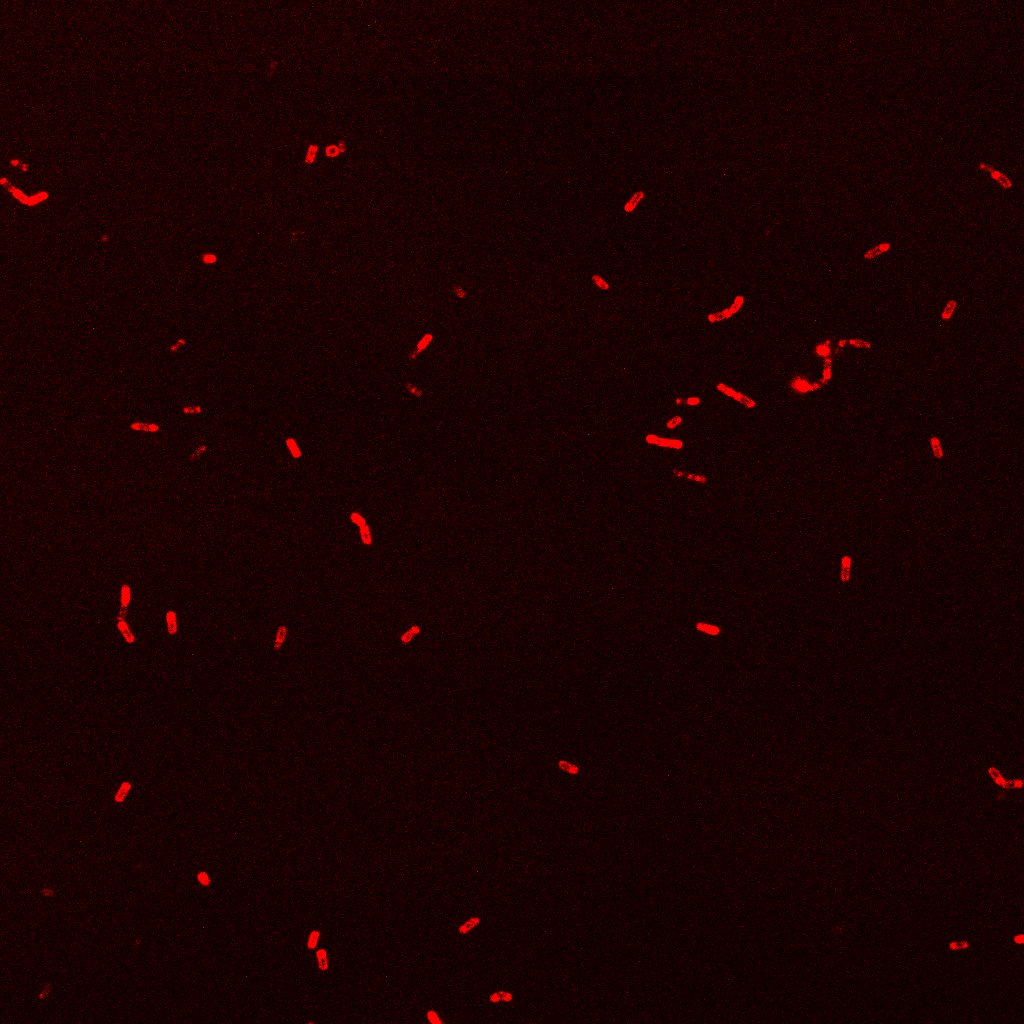

Supplement: Figure 3—source data 1. [file elife-74275-fig3-data1.zip › Figure 3-source data 1/Figure 3B images/N48A 3 hr 3 membrane.jpg]

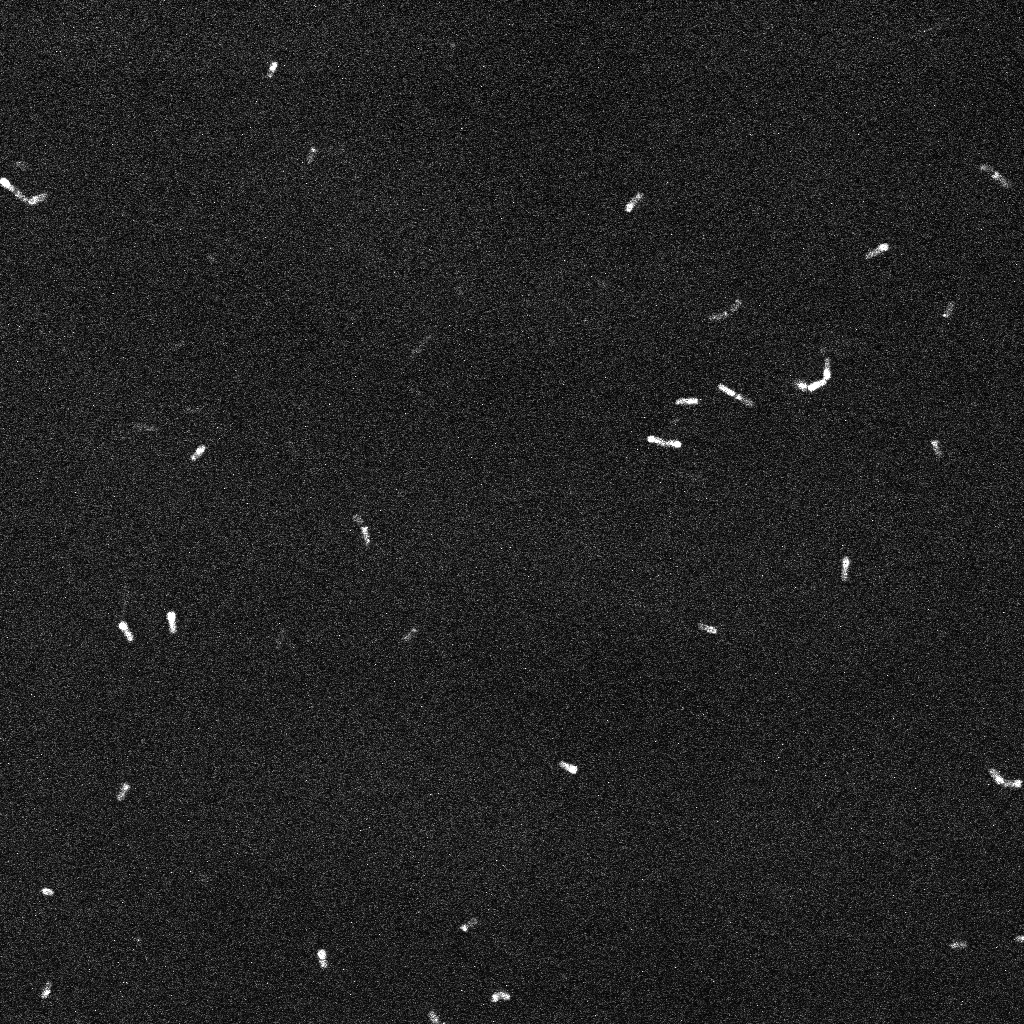

Supplement: Figure 3—source data 1. [file elife-74275-fig3-data1.zip › Figure 3-source data 1/Figure 3B images/N48A 3 hr 3.tif]

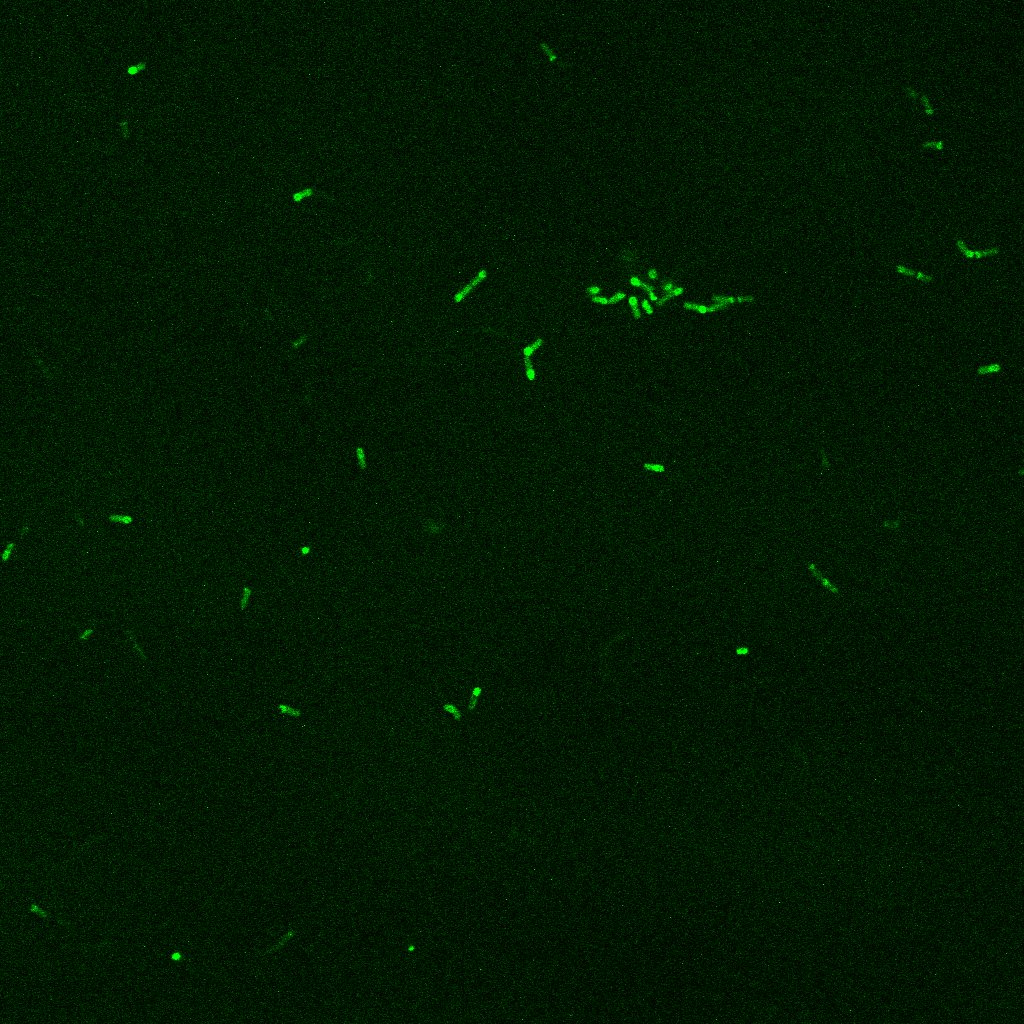

Supplement: Figure 3—source data 1. [file elife-74275-fig3-data1.zip › Figure 3-source data 1/Figure 3B images/N48A 3hr 1 GFP.jpg]

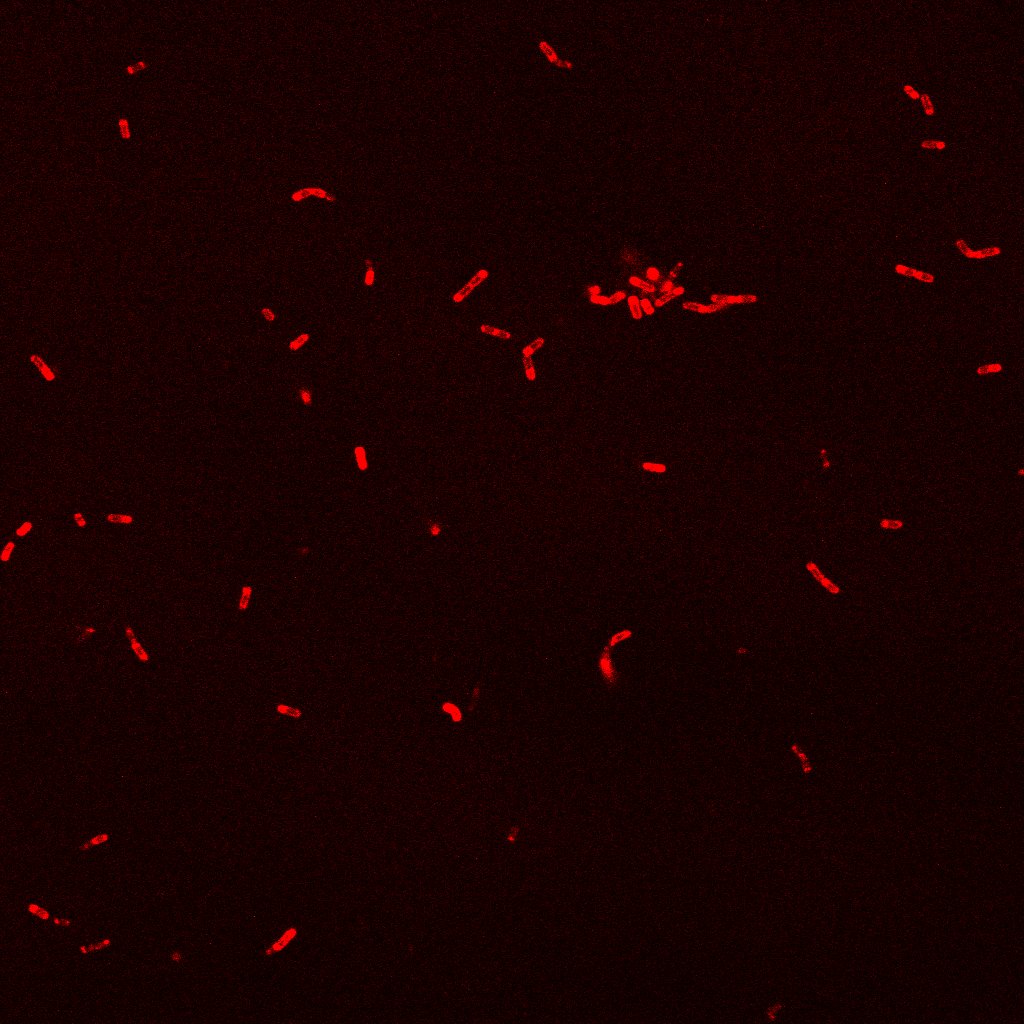

Supplement: Figure 3—source data 1. [file elife-74275-fig3-data1.zip › Figure 3-source data 1/Figure 3B images/N48A 3hr 1 membrane.jpg]

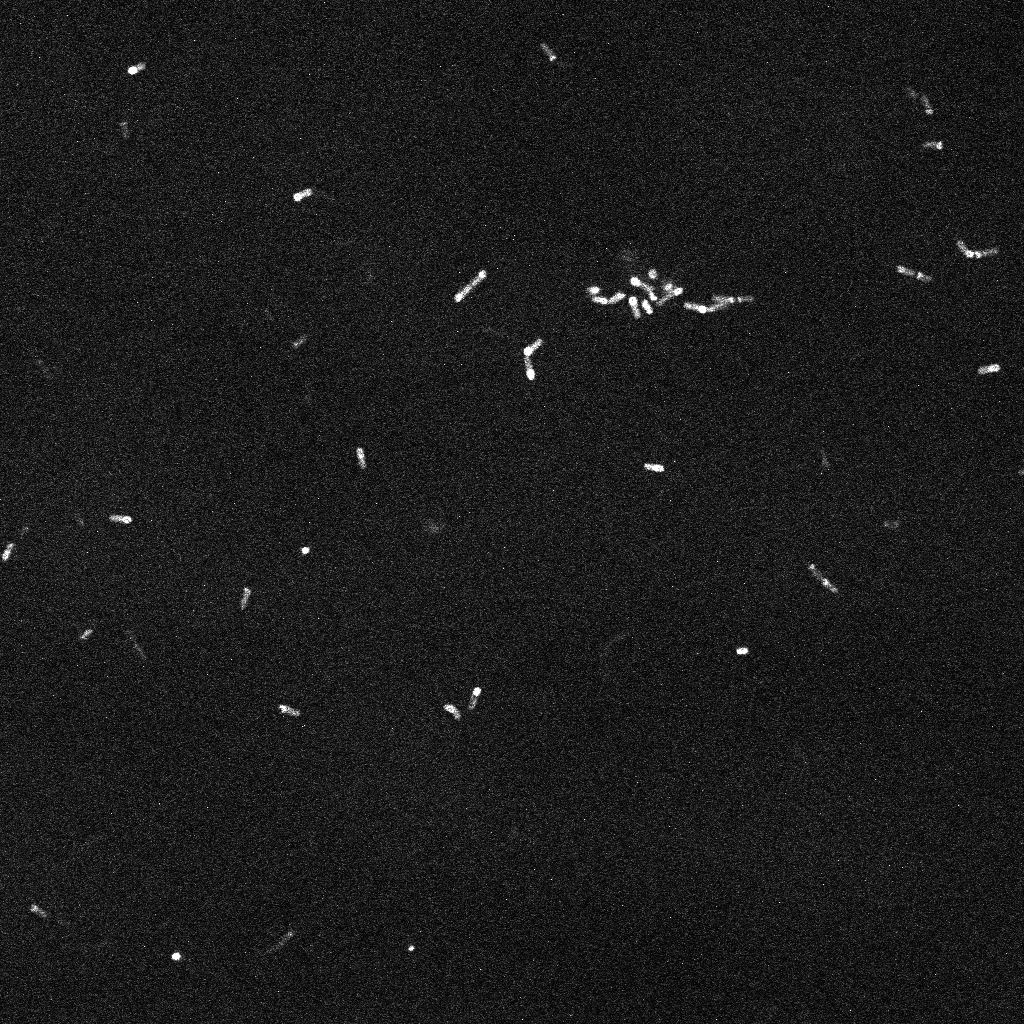

Supplement: Figure 3—source data 1. [file elife-74275-fig3-data1.zip › Figure 3-source data 1/Figure 3B images/N48A 3hr 1.tif]

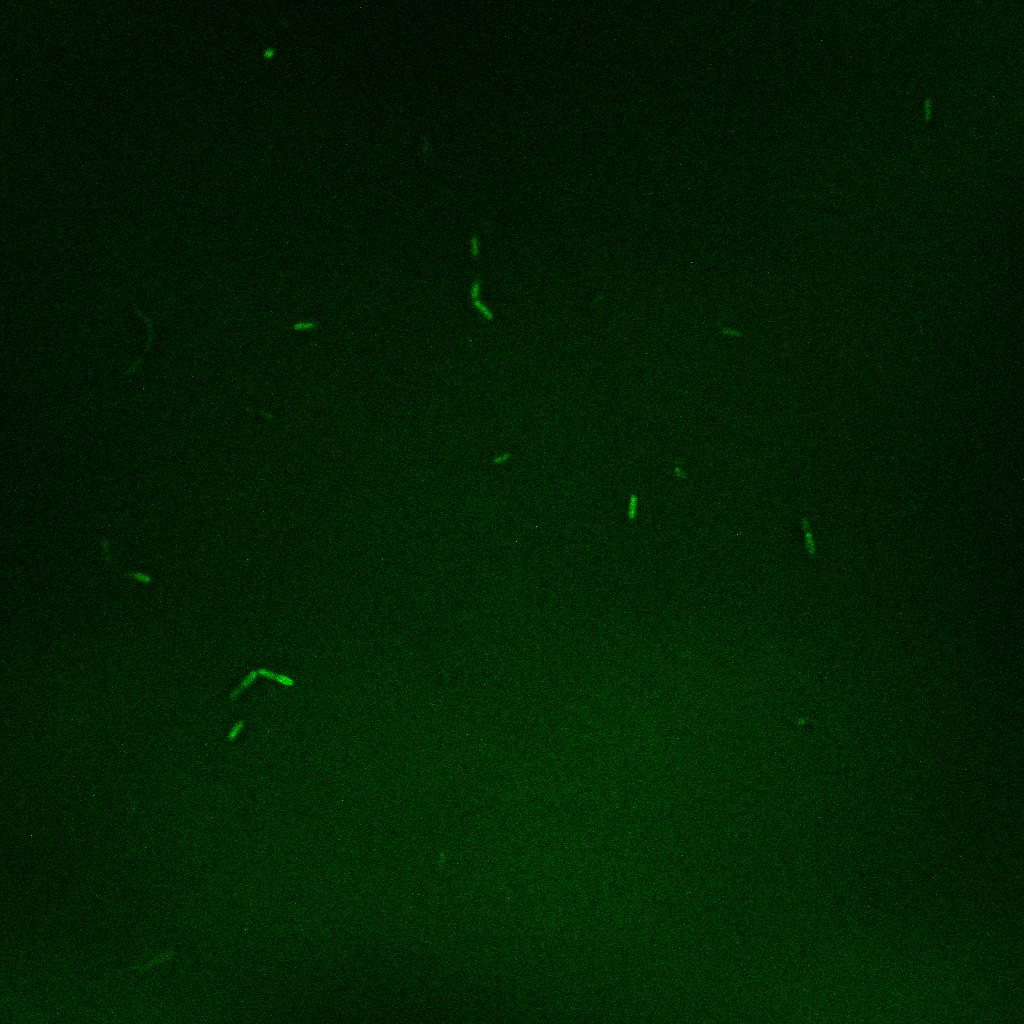

Supplement: Figure 3—source data 1. [file elife-74275-fig3-data1.zip › Figure 3-source data 1/Figure 3B images/N61A 3 hrs 1 GFP.jpg]

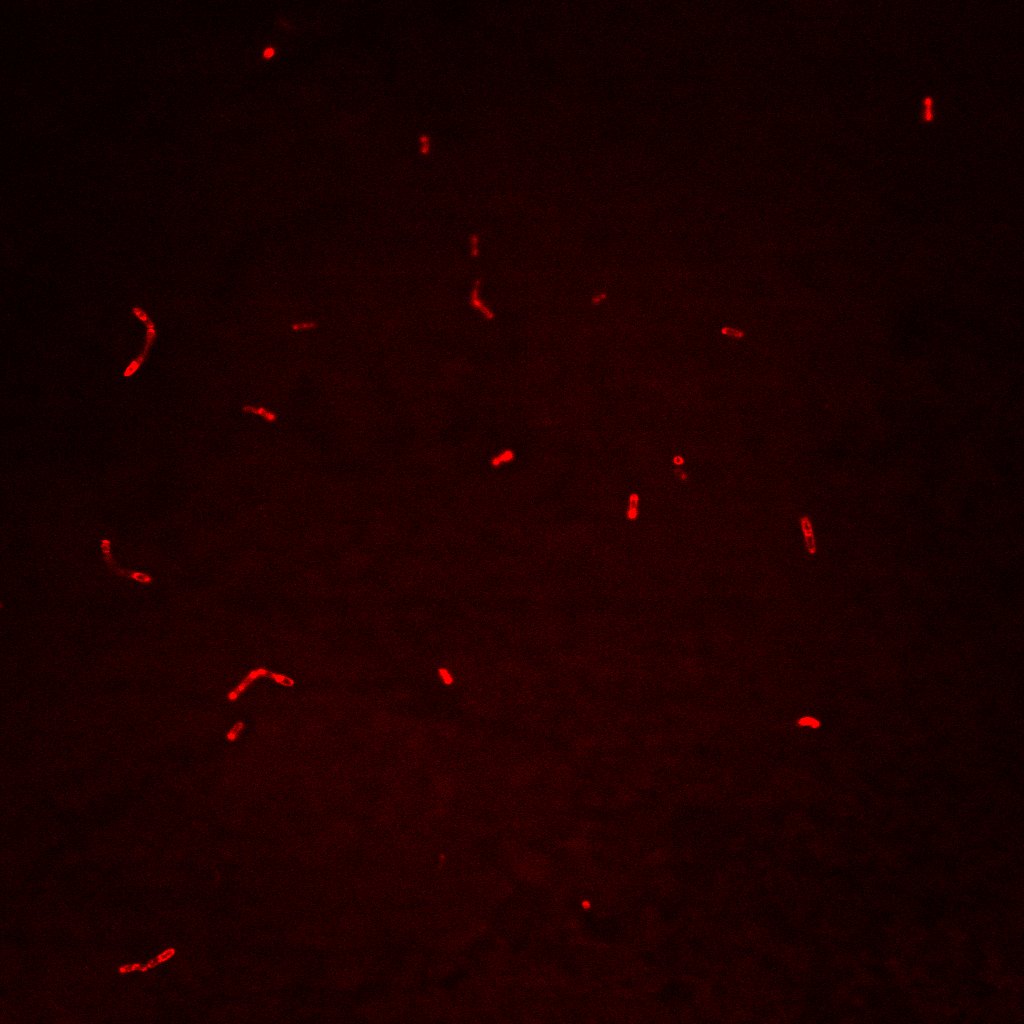

Supplement: Figure 3—source data 1. [file elife-74275-fig3-data1.zip › Figure 3-source data 1/Figure 3B images/N61A 3 hrs 1 membrane.jpg]

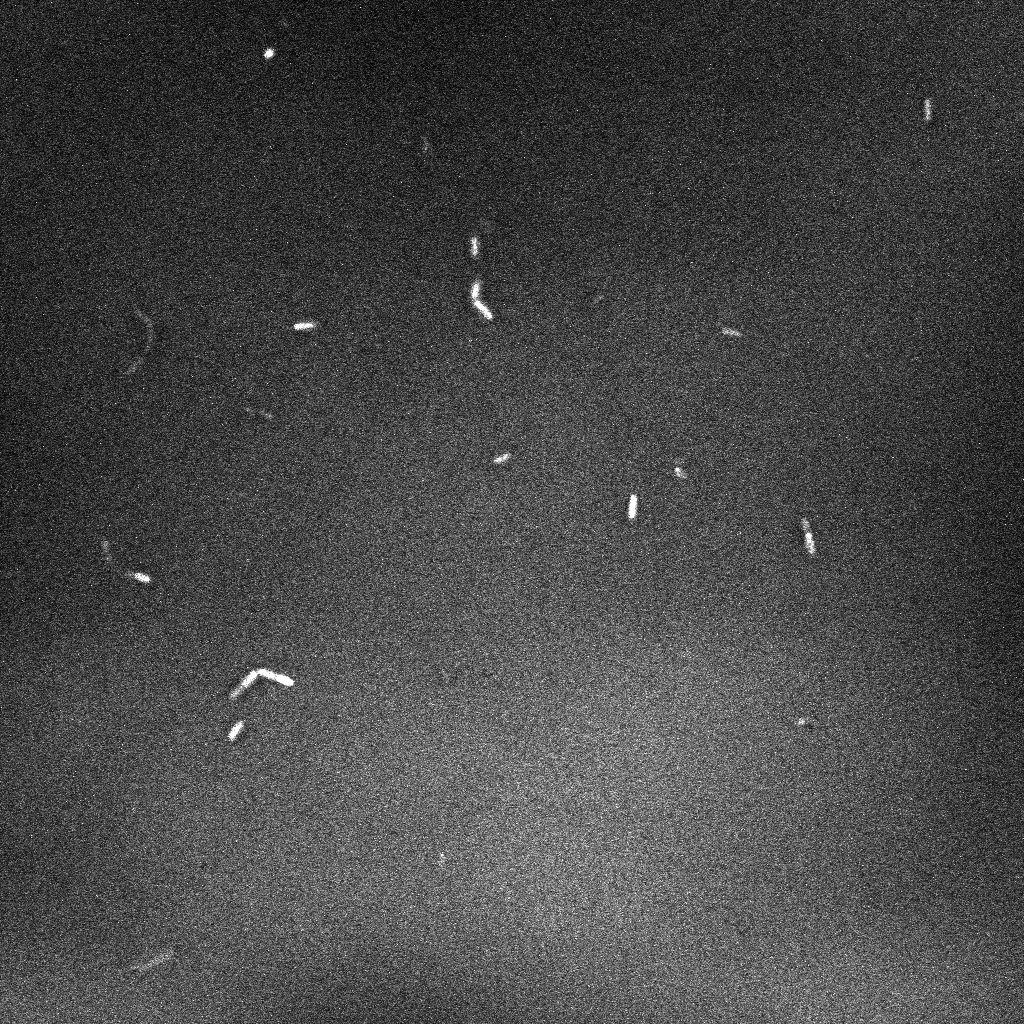

Supplement: Figure 3—source data 1. [file elife-74275-fig3-data1.zip › Figure 3-source data 1/Figure 3B images/N61A 3 hrs 1.tif]

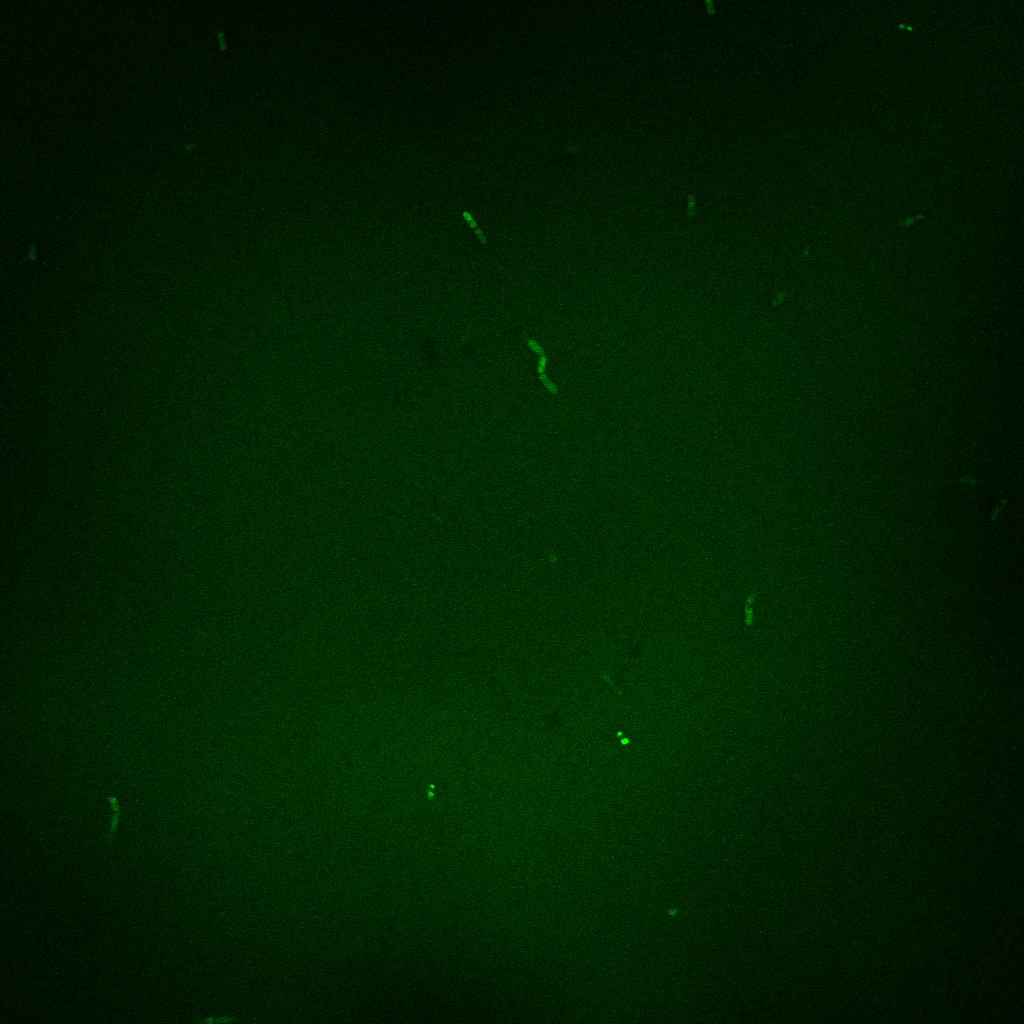

Supplement: Figure 3—source data 1. [file elife-74275-fig3-data1.zip › Figure 3-source data 1/Figure 3B images/N61A 3 hrs 2 GFP.jpg]

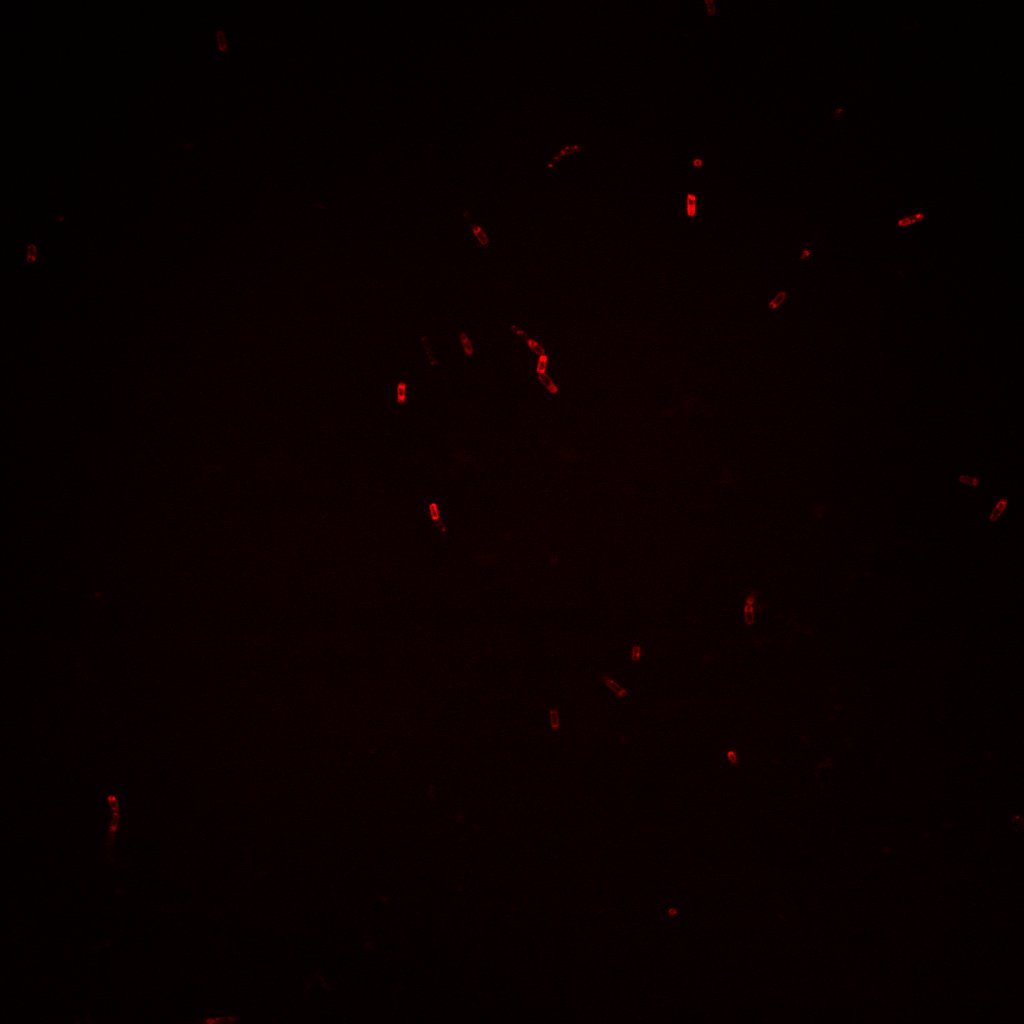

Supplement: Figure 3—source data 1. [file elife-74275-fig3-data1.zip › Figure 3-source data 1/Figure 3B images/N61A 3 hrs 2 membrane.jpg]

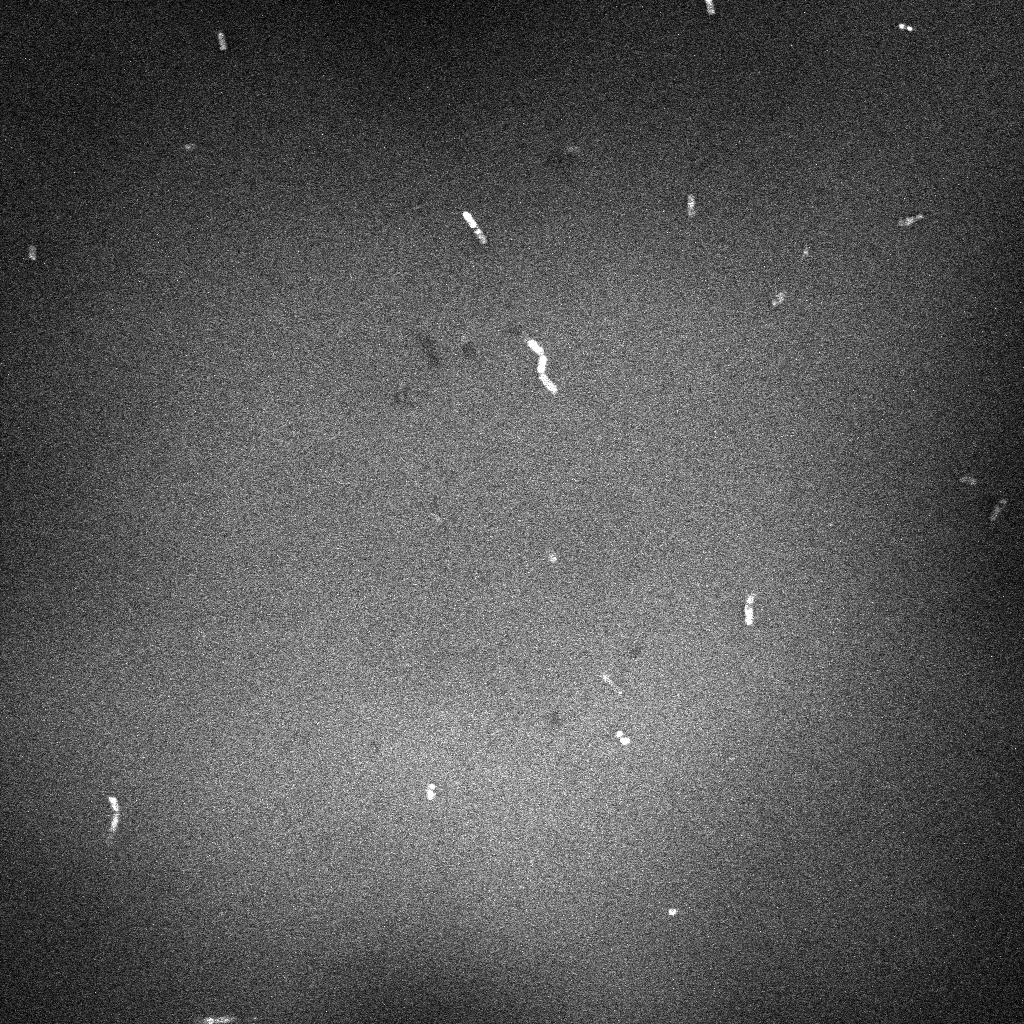

Supplement: Figure 3—source data 1. [file elife-74275-fig3-data1.zip › Figure 3-source data 1/Figure 3B images/N61A 3 hrs 2.tif]

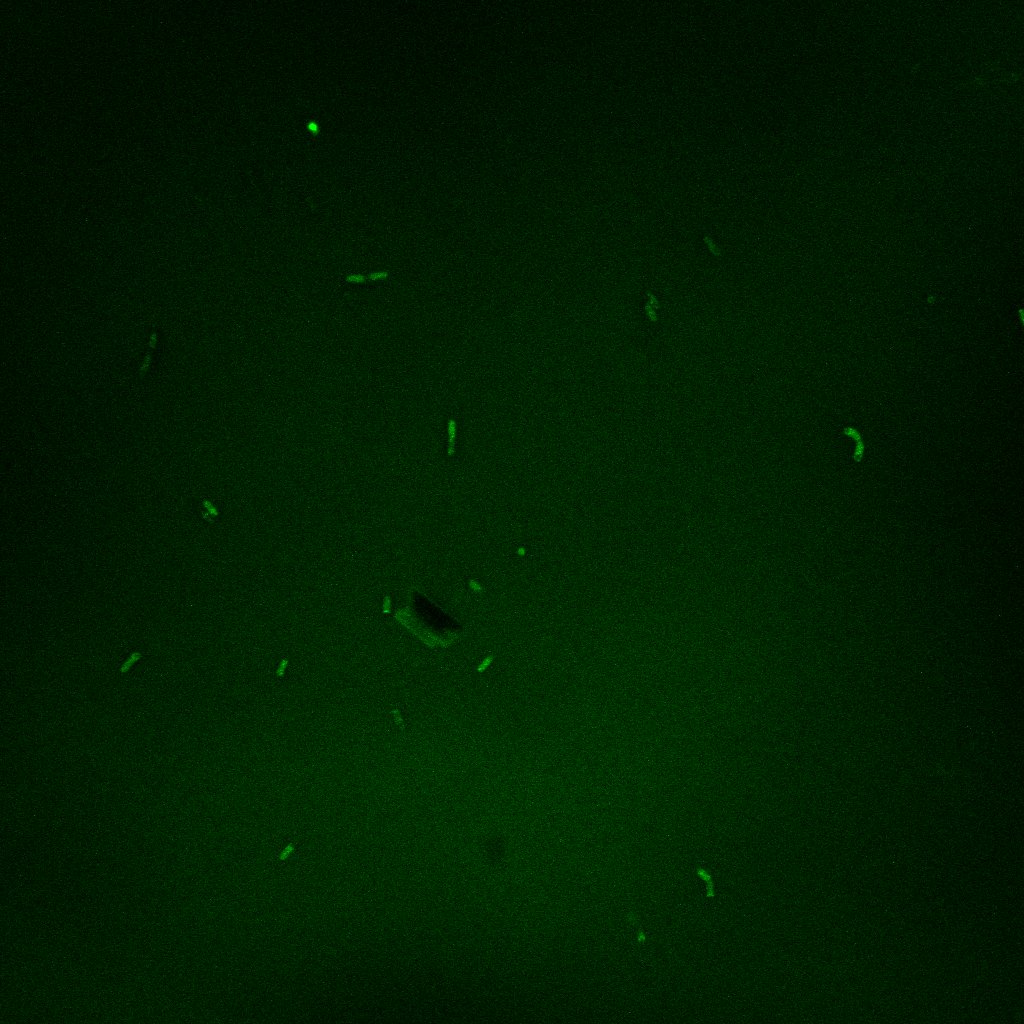

Supplement: Figure 3—source data 1. [file elife-74275-fig3-data1.zip › Figure 3-source data 1/Figure 3B images/N61A 3 hrs 3 GFP.jpg]

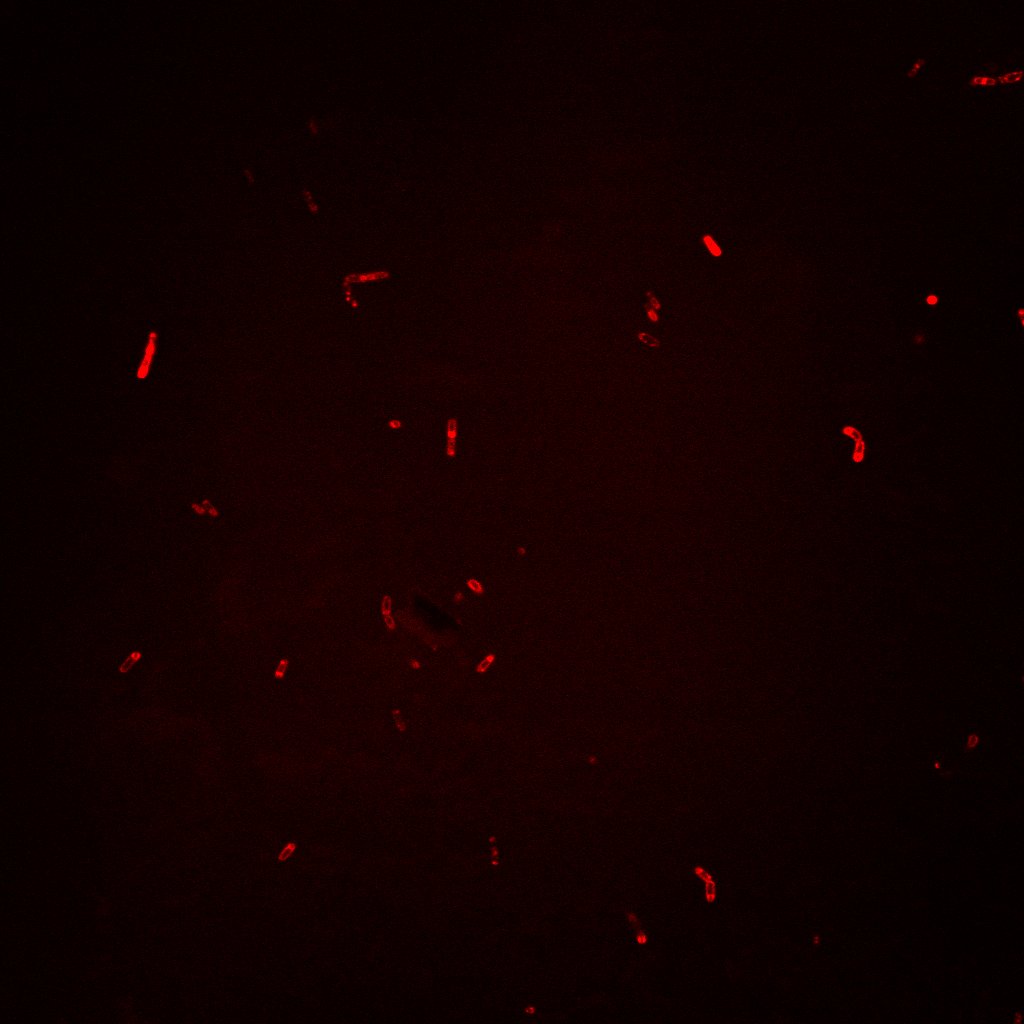

Supplement: Figure 3—source data 1. [file elife-74275-fig3-data1.zip › Figure 3-source data 1/Figure 3B images/N61A 3 hrs 3 membrane.jpg]

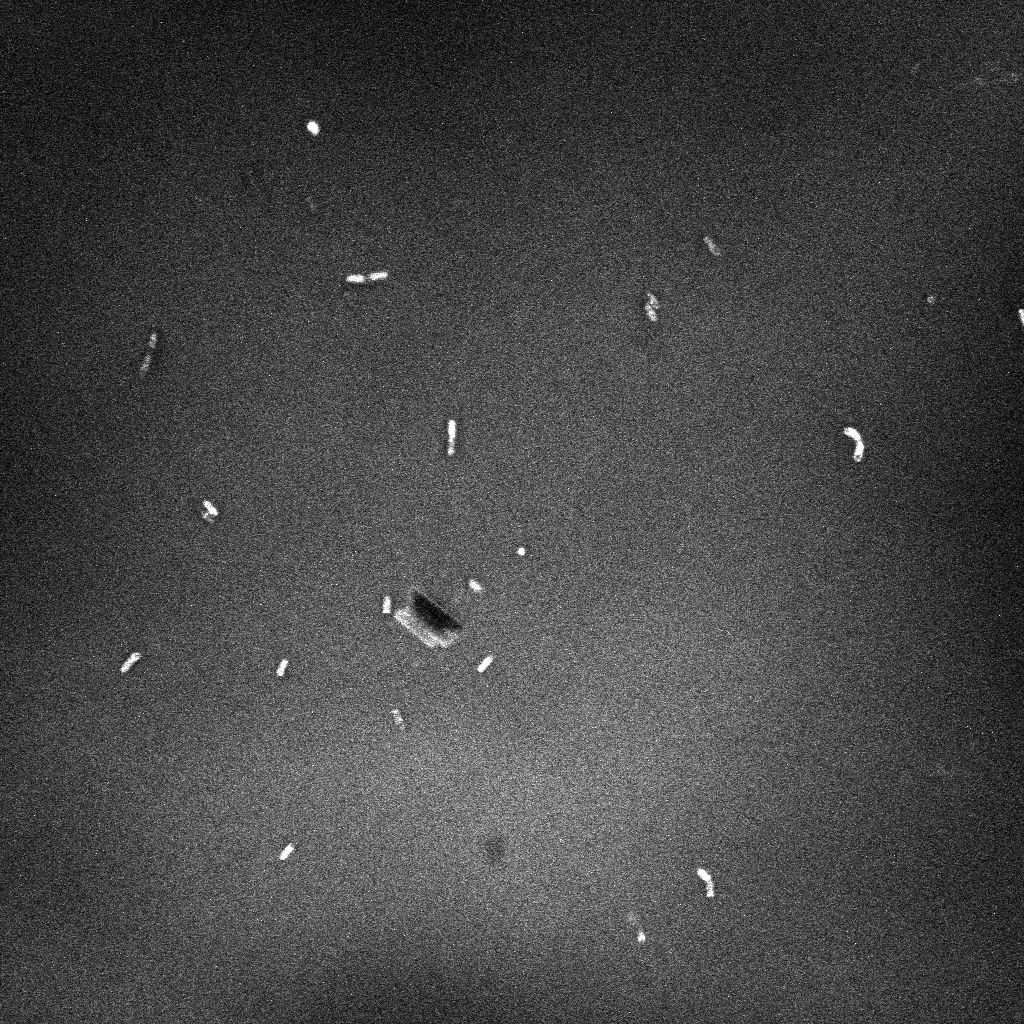

Supplement: Figure 3—source data 1. [file elife-74275-fig3-data1.zip › Figure 3-source data 1/Figure 3B images/N61A 3 hrs 3.tif]

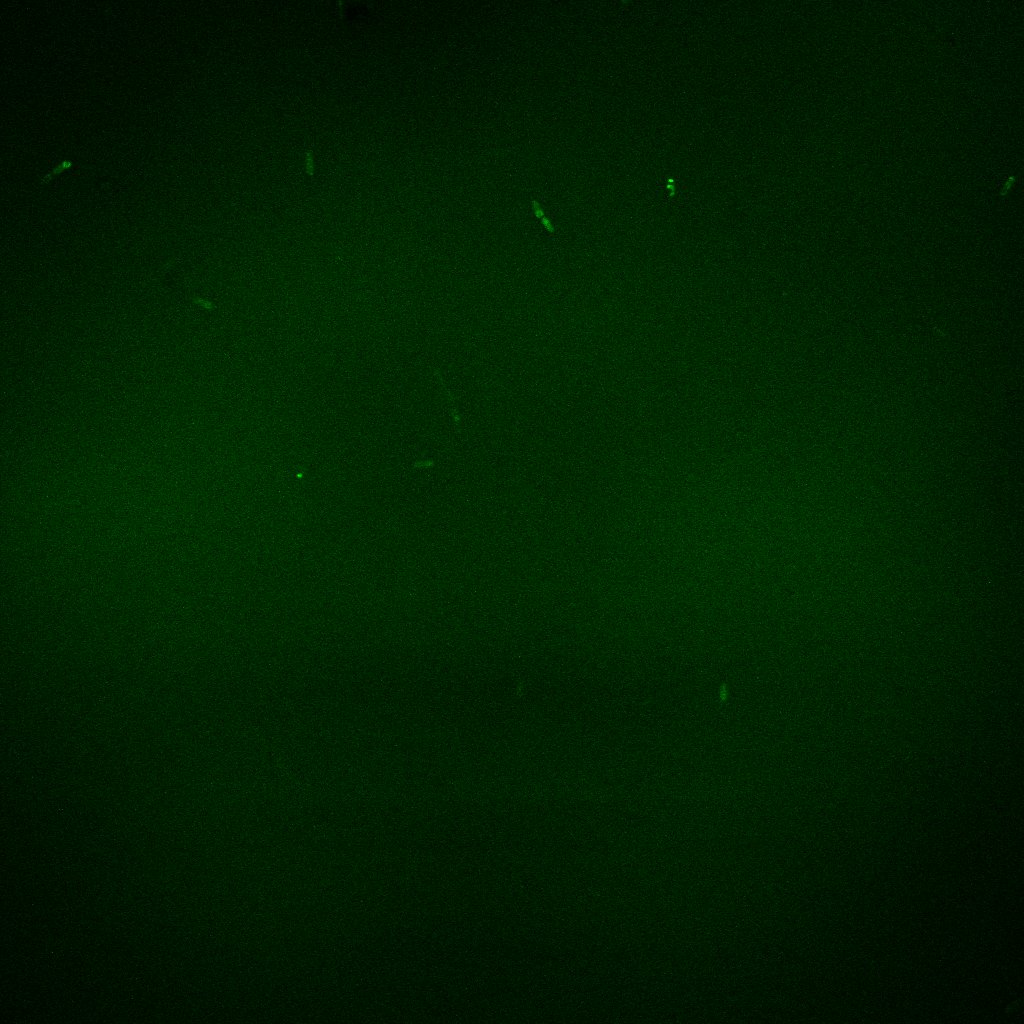

Supplement: Figure 3—source data 1. [file elife-74275-fig3-data1.zip › Figure 3-source data 1/Figure 3B images/T64A 3 hrs 1 GFP.jpg]

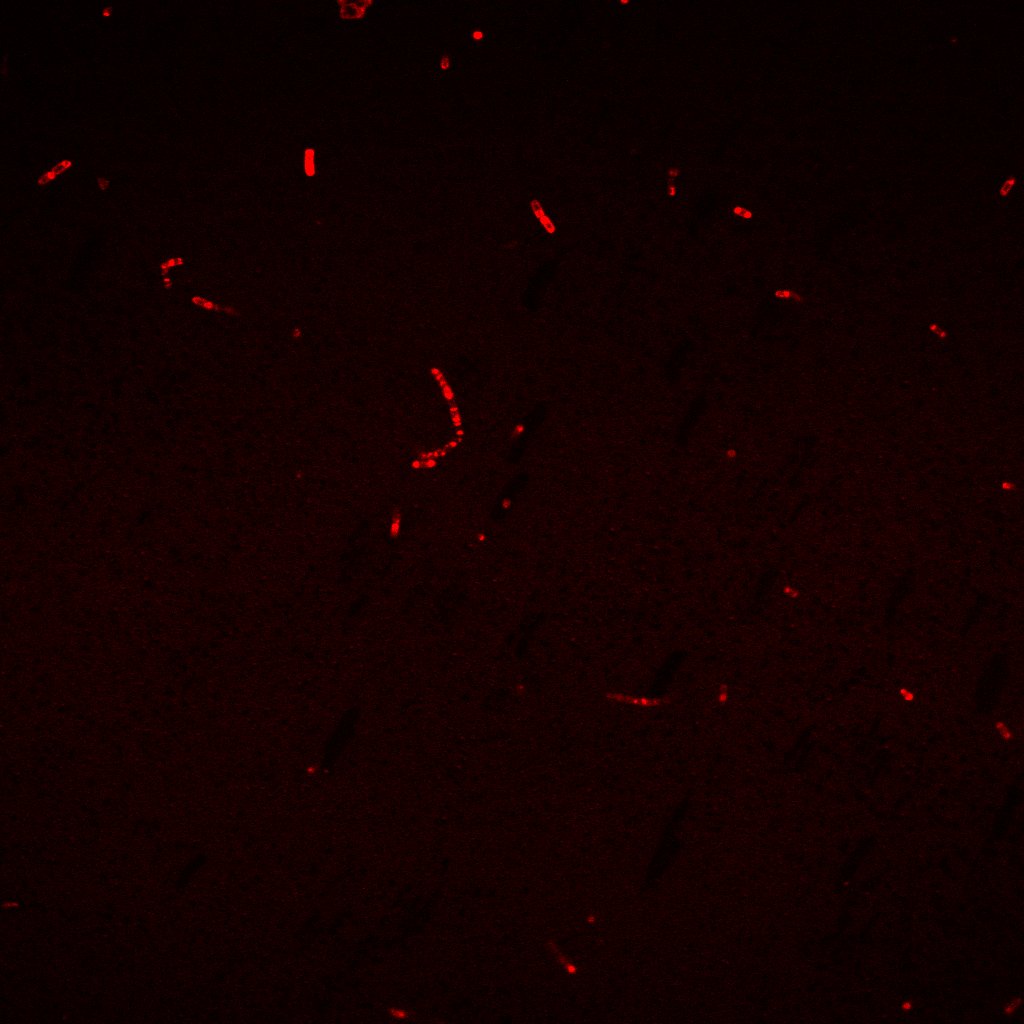

Supplement: Figure 3—source data 1. [file elife-74275-fig3-data1.zip › Figure 3-source data 1/Figure 3B images/T64A 3 hrs 1 membrane.jpg]
