# Supplementary material for: Inhibitory proteins block substrate access by occupying the active site cleft of Bacillus subtilis intramembrane protease SpoIVFB
Source: eLife. 2022 Apr 26;11:e74275. doi: 10.7554/eLife.74275 (PMC9042235; doi:10.7554/eLife.74275)
Supplement: Figure 1—figure supplement 2—source data 1. [file elife-74275-fig1-figsupp2-data1.zip › Figure 1-figure supplement 2-source data 1/figure supplement 2A/fig sup 2A annotated blots.pptx]

## Slide 1
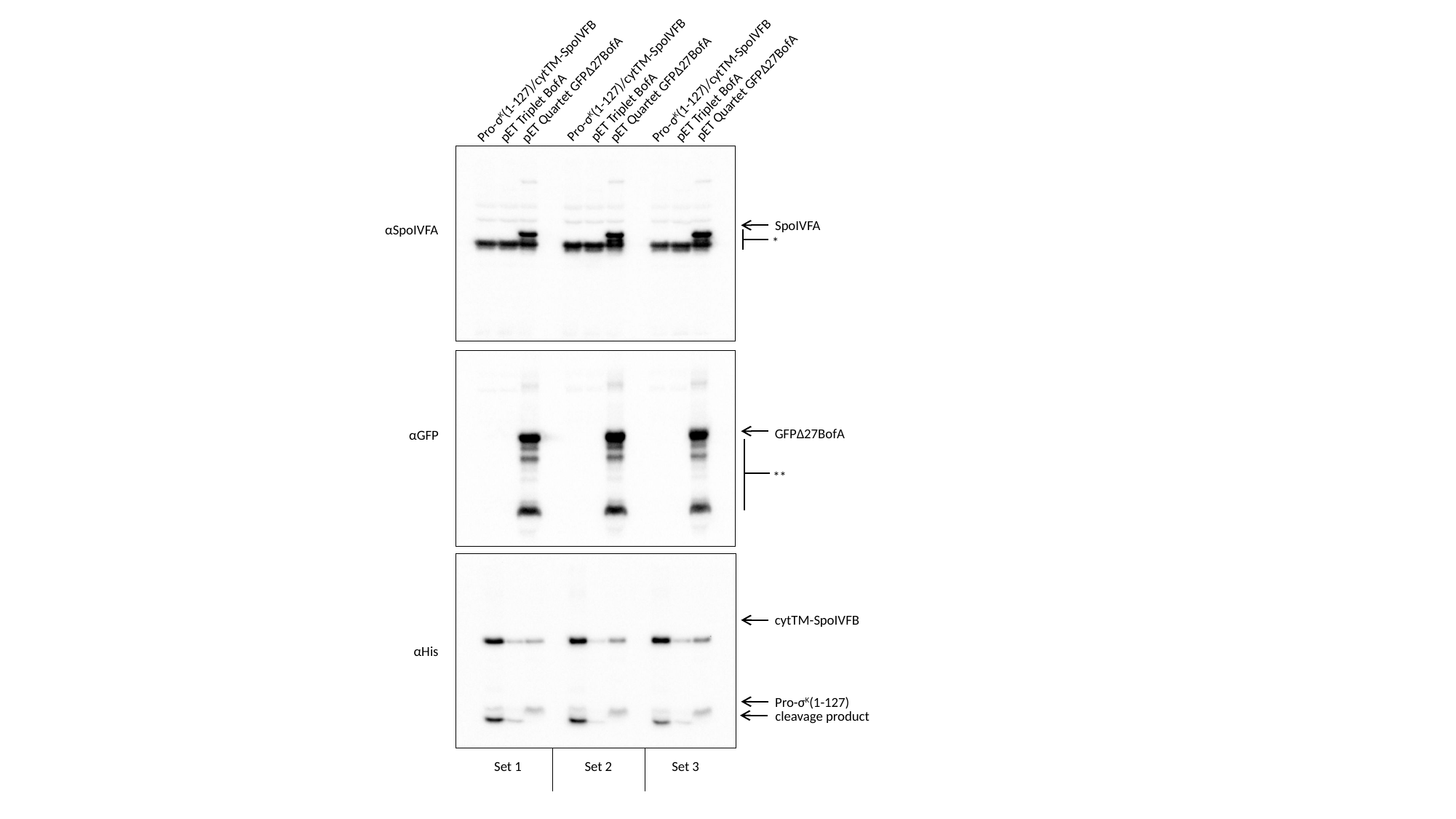

Pro-σK(1-127)/cytTM-SpoIVFB
Pro-σK(1-127)/cytTM-SpoIVFB
Pro-σK(1-127)/cytTM-SpoIVFB
pET Quartet GFPΔ27BofA
pET Quartet GFPΔ27BofA
pET Quartet GFPΔ27BofA
pET Triplet BofA
pET Triplet BofA
pET Triplet BofA
SpoIVFA
αSpoIVFA
*
GFPΔ27BofA
αGFP
**
cytTM-SpoIVFB
αHis
Pro-σK(1-127)
cleavage product
Set 1
Set 2
Set 3
