# Supplementary material for: Inhibitory proteins block substrate access by occupying the active site cleft of Bacillus subtilis intramembrane protease SpoIVFB
Source: eLife. 2022 Apr 26;11:e74275. doi: 10.7554/eLife.74275 (PMC9042235; doi:10.7554/eLife.74275)
Supplement: Figure 1—figure supplement 2—source data 1. [file elife-74275-fig1-figsupp2-data1.zip › Figure 1-figure supplement 2-source data 1/figure supplement 2B/fig sup 2B annotated blots.pptx]

## Slide 1
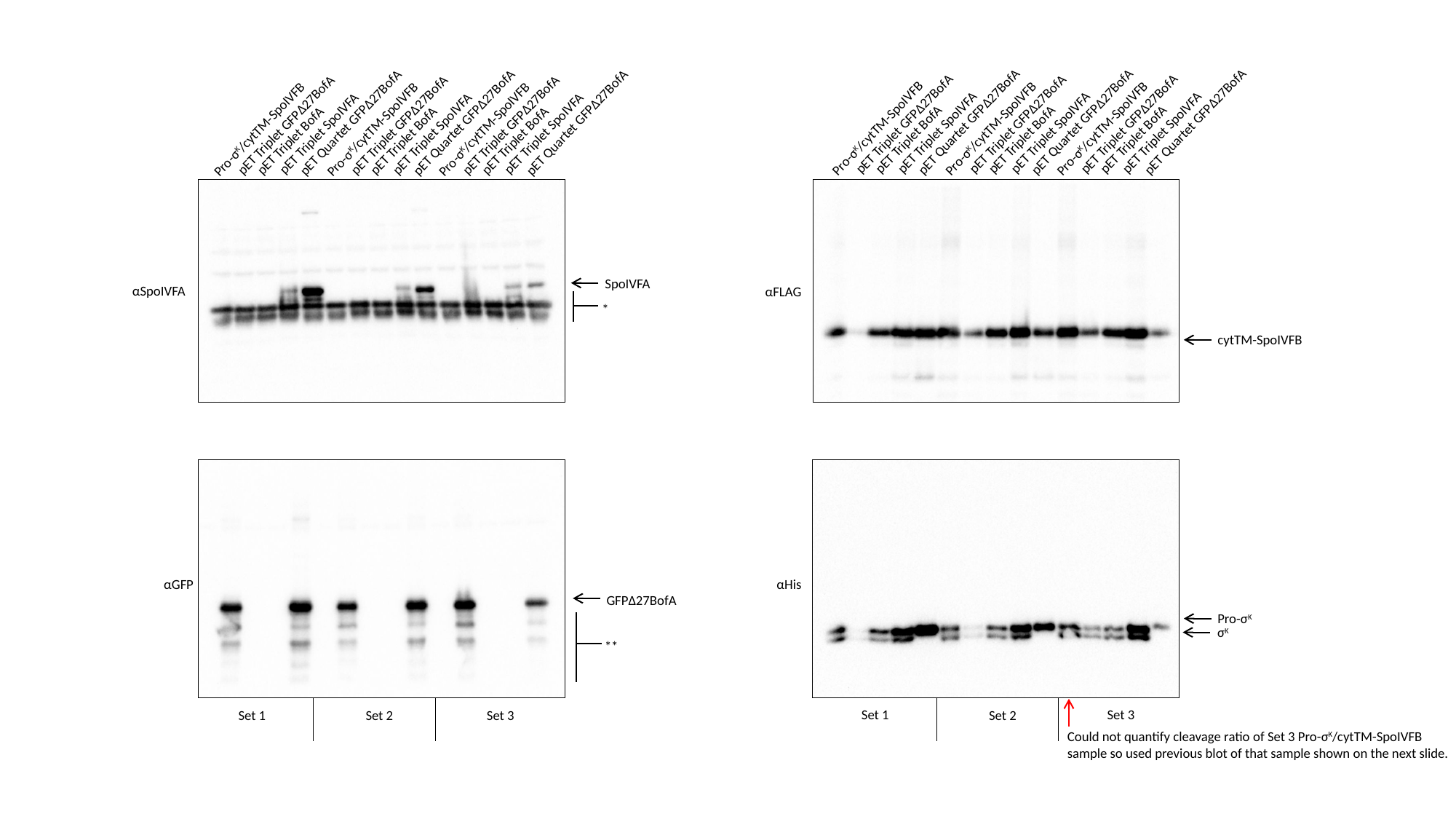

pET Quartet GFPΔ27BofA
pET Quartet GFPΔ27BofA
pET Quartet GFPΔ27BofA
pET Quartet GFPΔ27BofA
pET Quartet GFPΔ27BofA
pET Quartet GFPΔ27BofA
Pro-σK/cytTM-SpoIVFB
Pro-σK/cytTM-SpoIVFB
Pro-σK/cytTM-SpoIVFB
Pro-σK/cytTM-SpoIVFB
Pro-σK/cytTM-SpoIVFB
Pro-σK/cytTM-SpoIVFB
pET Triplet GFPΔ27BofA
pET Triplet GFPΔ27BofA
pET Triplet GFPΔ27BofA
pET Triplet GFPΔ27BofA
pET Triplet GFPΔ27BofA
pET Triplet GFPΔ27BofA
pET Triplet SpoIVFA
pET Triplet SpoIVFA
pET Triplet SpoIVFA
pET Triplet SpoIVFA
pET Triplet SpoIVFA
pET Triplet SpoIVFA
pET Triplet BofA
pET Triplet BofA
pET Triplet BofA
pET Triplet BofA
pET Triplet BofA
pET Triplet BofA
SpoIVFA
αSpoIVFA
αFLAG
*
cytTM-SpoIVFB
αGFP
αHis
GFPΔ27BofA
Pro-σK
σK
**
Set 1
Set 3
Set 2
Set 1
Set 3
Set 2
Could not quantify cleavage ratio of Set 3 Pro-σK/cytTM-SpoIVFB sample so used previous blot of that sample shown on the next slide.

## Slide 2
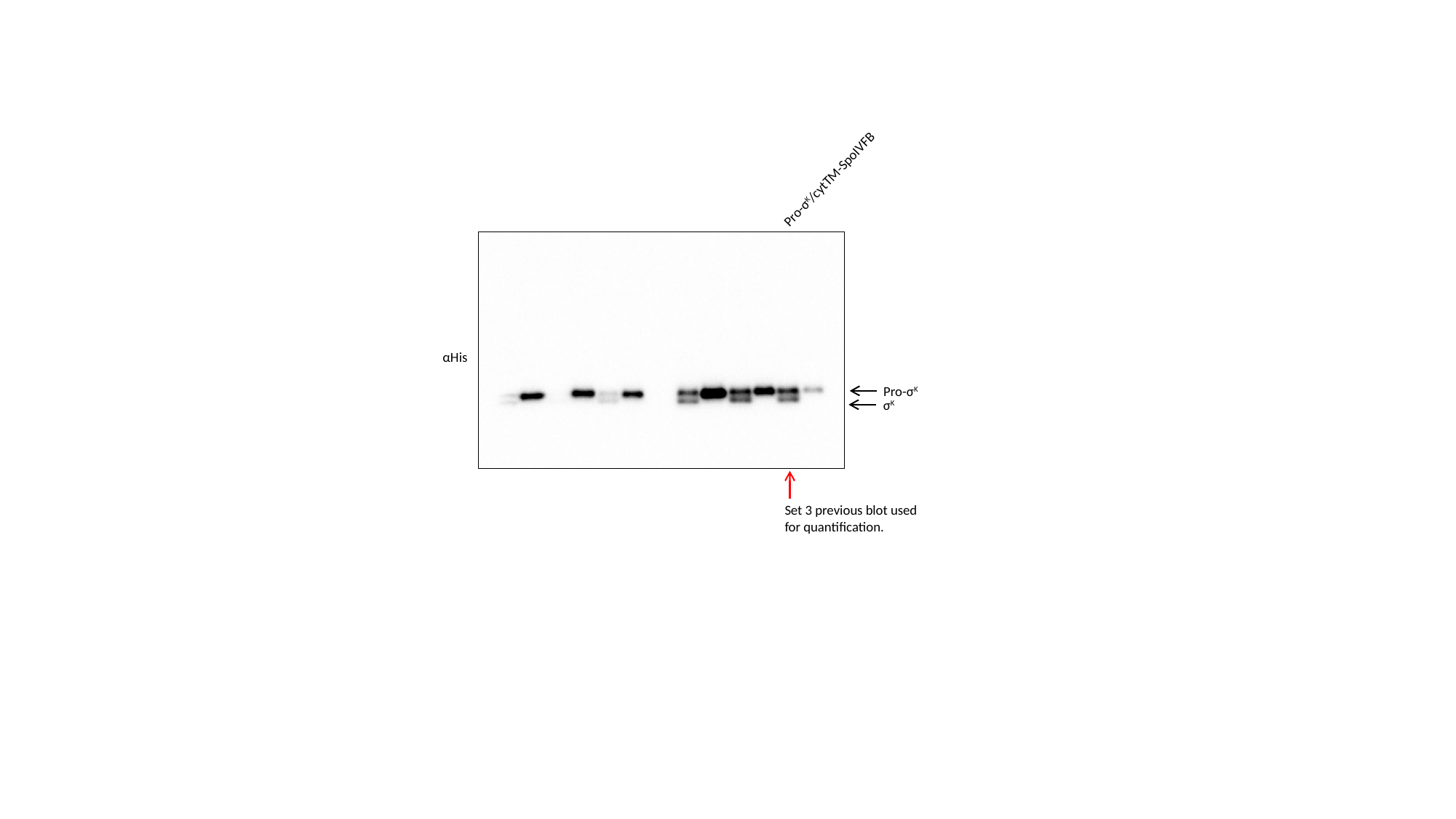

Pro-σK/cytTM-SpoIVFB
αHis
Pro-σK
σK
Set 3 previous blot used for quantification.
