# Supplementary material for: Inhibitory proteins block substrate access by occupying the active site cleft of Bacillus subtilis intramembrane protease SpoIVFB
Source: eLife. 2022 Apr 26;11:e74275. doi: 10.7554/eLife.74275 (PMC9042235; doi:10.7554/eLife.74275)
Supplement: Figure 1—figure supplement 3—source data 1. [file elife-74275-fig1-figsupp3-data1.zip › Figure 1-figure supplement 3-source data 1/fig sup 3 annotated blots.pptx]

## Slide 1
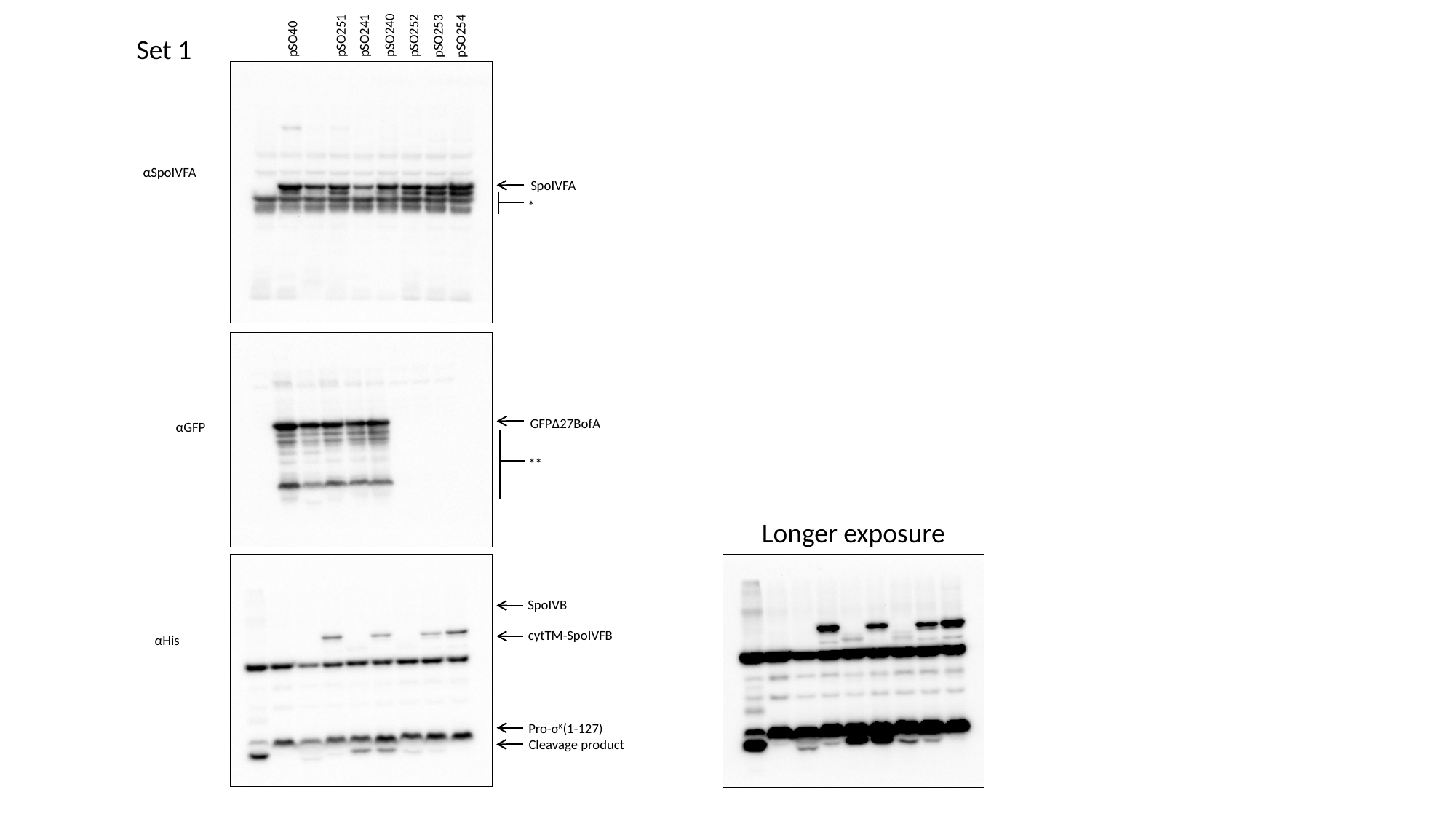

pSO240
pSO251
pSO241
pSO252
pSO253
pSO254
pSO40
Set 1
αSpoIVFA
SpoIVFA
*
GFPΔ27BofA
αGFP
**
Longer exposure
SpoIVB
cytTM-SpoIVFB
αHis
Pro-σK(1-127)
Cleavage product

## Slide 2
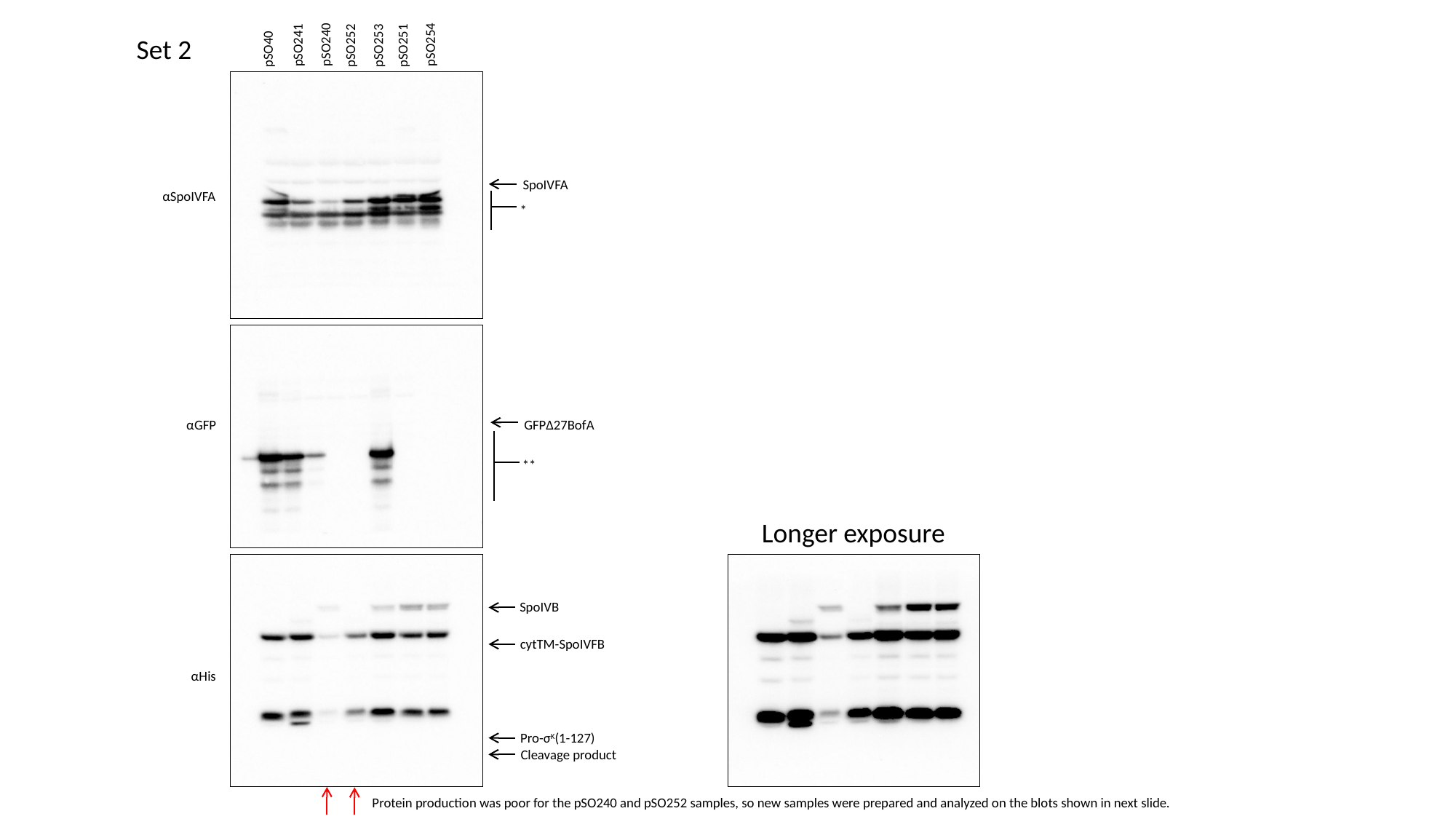

Set 2
pSO240
pSO254
pSO241
pSO252
pSO253
pSO251
pSO40
SpoIVFA
αSpoIVFA
*
αGFP
GFPΔ27BofA
**
Longer exposure
SpoIVB
cytTM-SpoIVFB
αHis
Pro-σK(1-127)
Cleavage product
Protein production was poor for the pSO240 and pSO252 samples, so new samples were prepared and analyzed on the blots shown in next slide.

## Slide 3
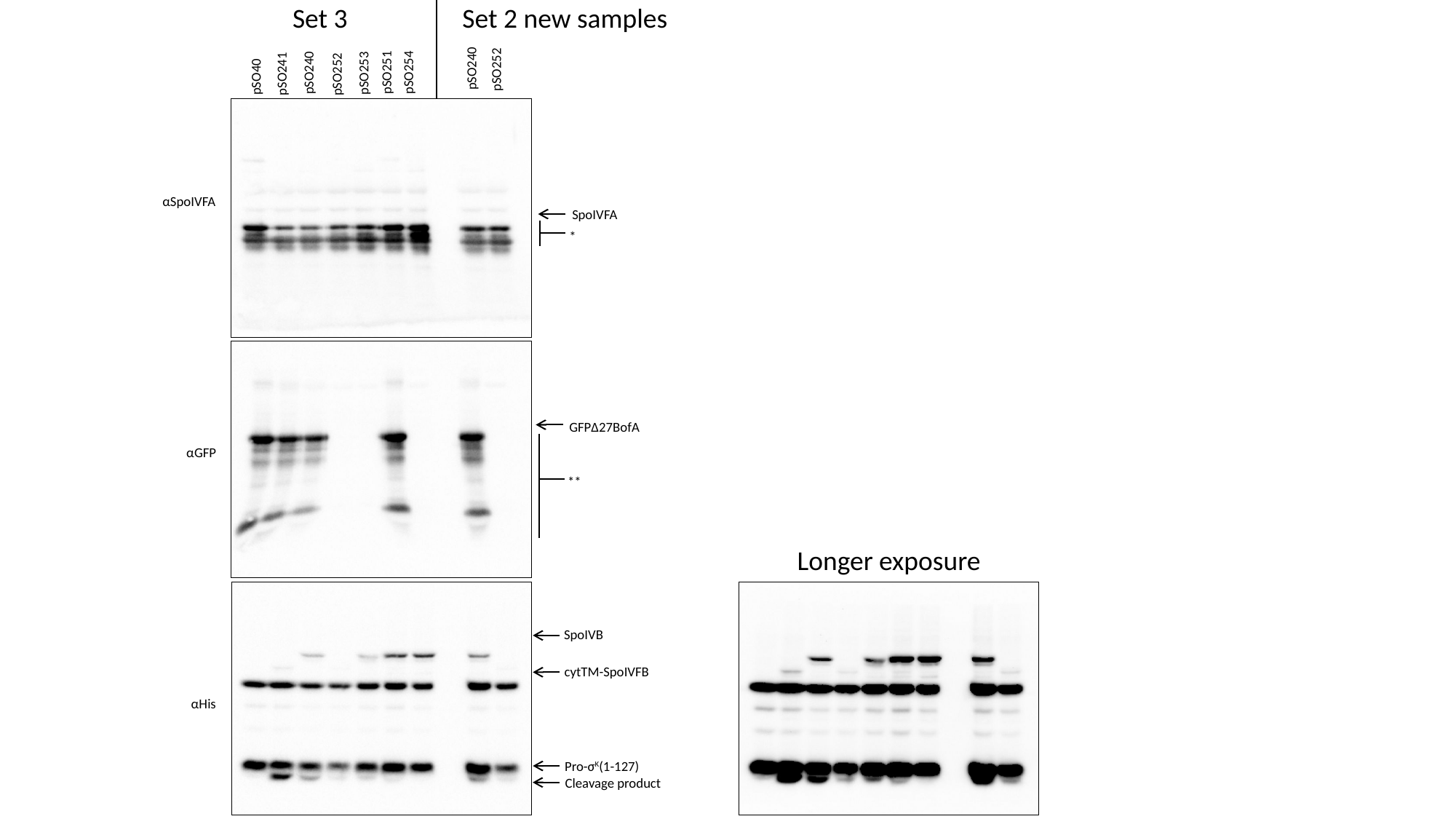

Set 3
Set 2 new samples
pSO240
pSO252
pSO251
pSO254
pSO240
pSO253
pSO241
pSO252
pSO40
αSpoIVFA
SpoIVFA
*
GFPΔ27BofA
αGFP
**
Longer exposure
SpoIVB
cytTM-SpoIVFB
αHis
Pro-σK(1-127)
Cleavage product
