# Supplementary material for: Inhibitory proteins block substrate access by occupying the active site cleft of Bacillus subtilis intramembrane protease SpoIVFB
Source: eLife. 2022 Apr 26;11:e74275. doi: 10.7554/eLife.74275 (PMC9042235; doi:10.7554/eLife.74275)
Supplement: Figure 1—figure supplement 4—source data 1. [file elife-74275-fig1-figsupp4-data1.zip › Figure 1-figure supplement 4-source data 1/fig sup 4 annotated blots.pptx]

## Slide 1
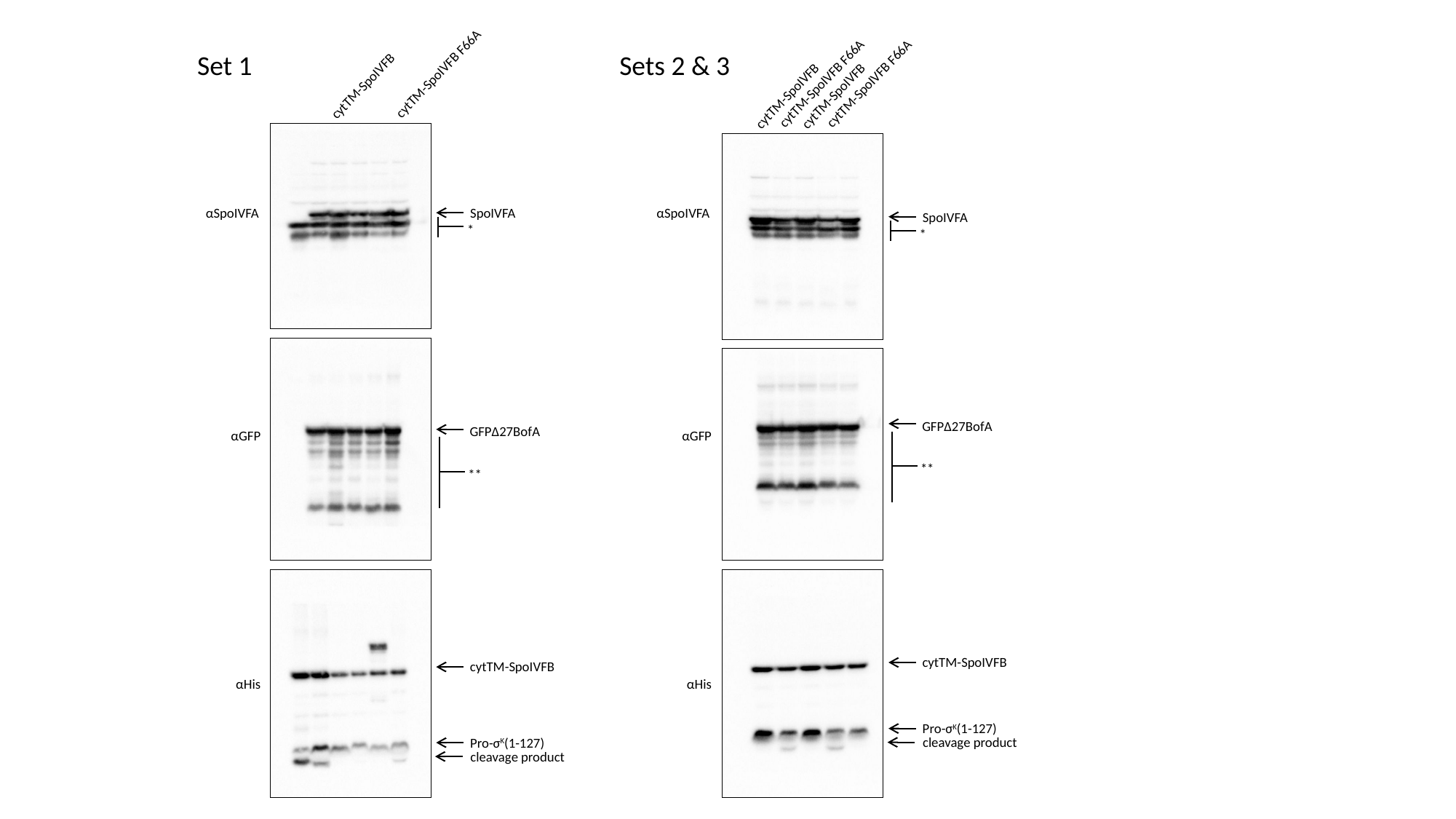

Set 1
Sets 2 & 3
cytTM-SpoIVFB F66A
cytTM-SpoIVFB F66A
cytTM-SpoIVFB F66A
cytTM-SpoIVFB
cytTM-SpoIVFB
cytTM-SpoIVFB
αSpoIVFA
SpoIVFA
αSpoIVFA
SpoIVFA
*
*
GFPΔ27BofA
GFPΔ27BofA
αGFP
αGFP
**
**
cytTM-SpoIVFB
cytTM-SpoIVFB
αHis
αHis
Pro-σK(1-127)
cleavage product
Pro-σK(1-127)
cleavage product
