# Supplementary material for: Inhibitory proteins block substrate access by occupying the active site cleft of Bacillus subtilis intramembrane protease SpoIVFB
Source: eLife. 2022 Apr 26;11:e74275. doi: 10.7554/eLife.74275 (PMC9042235; doi:10.7554/eLife.74275)
Supplement: Figure 2—source data 1. [file elife-74275-fig2-data1.zip › Figure 2-source data 1/Fig2 annotated blots.pptx]

## Slide 1
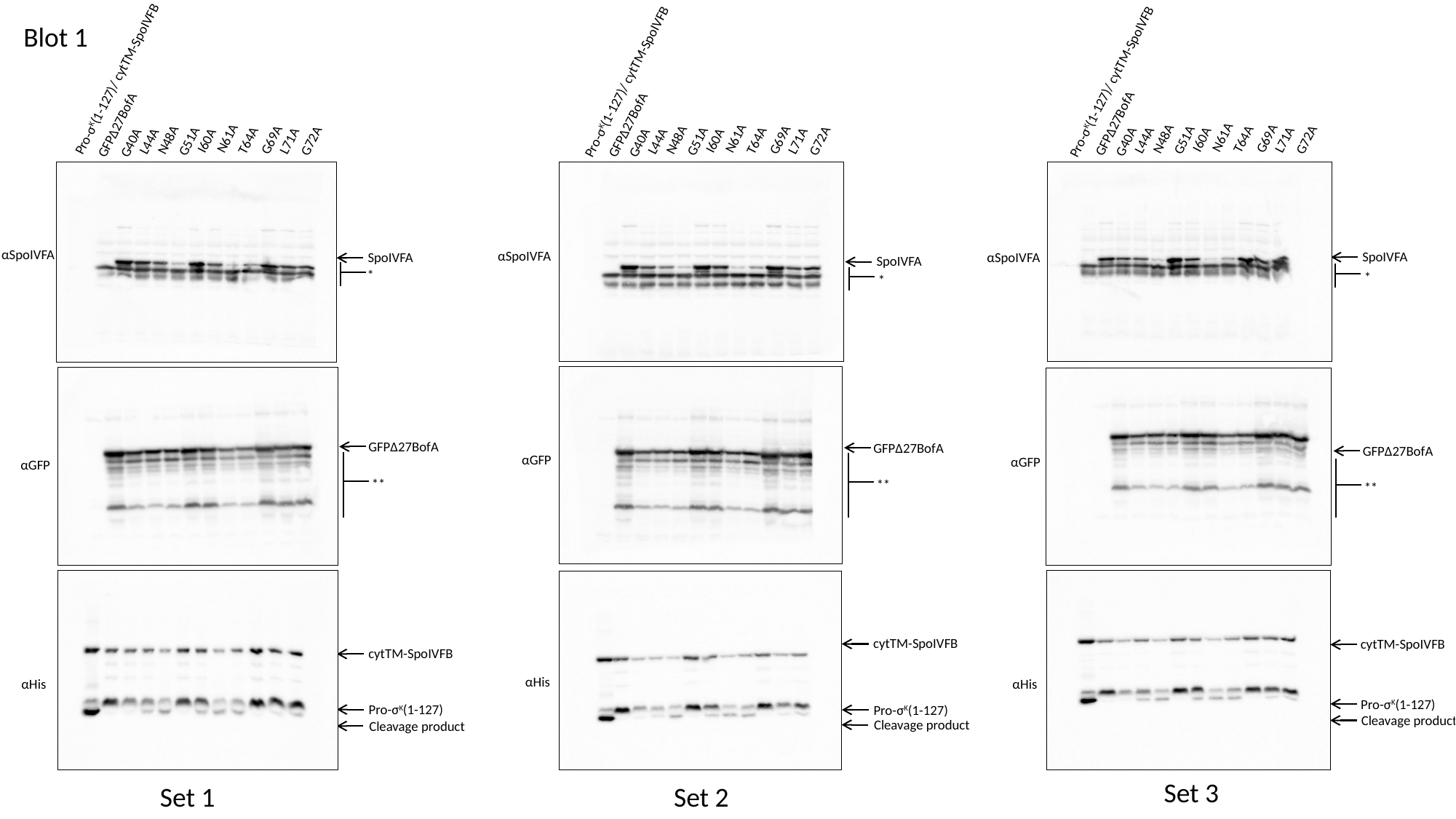

Blot 1
Pro-σK(1-127)/ cytTM-SpoIVFB
Pro-σK(1-127)/ cytTM-SpoIVFB
Pro-σK(1-127)/ cytTM-SpoIVFB
GFPΔ27BofA
GFPΔ27BofA
GFPΔ27BofA
I60A
N61A
T64A
I60A
N61A
G69A
L71A
I60A
N61A
L44A
N48A
T64A
G72A
T64A
G69A
L71A
G69A
L71A
L44A
N48A
L44A
N48A
G72A
G72A
G51A
G51A
G51A
G40A
G40A
G40A
αSpoIVFA
αSpoIVFA
SpoIVFA
SpoIVFA
αSpoIVFA
SpoIVFA
*
*
*
GFPΔ27BofA
GFPΔ27BofA
GFPΔ27BofA
αGFP
αGFP
αGFP
**
**
**
cytTM-SpoIVFB
cytTM-SpoIVFB
cytTM-SpoIVFB
αHis
αHis
αHis
Pro-σK(1-127)
Pro-σK(1-127)
Pro-σK(1-127)
Cleavage product
Cleavage product
Cleavage product
Set 3
Set 1
Set 2

## Slide 2
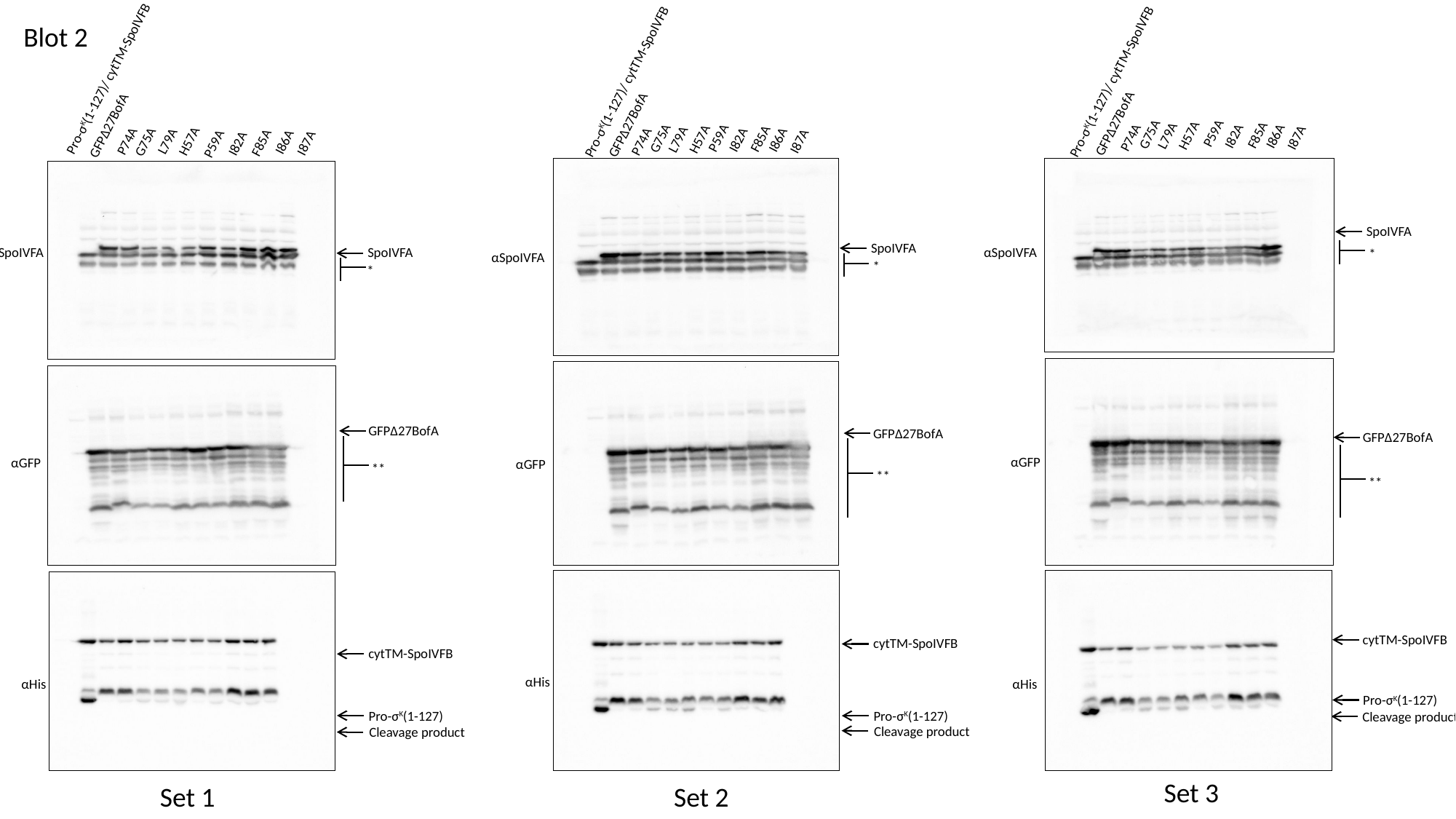

Blot 2
Pro-σK(1-127)/ cytTM-SpoIVFB
Pro-σK(1-127)/ cytTM-SpoIVFB
Pro-σK(1-127)/ cytTM-SpoIVFB
GFPΔ27BofA
GFPΔ27BofA
GFPΔ27BofA
P59A
I82A
F85A
I86A
G75A
L79A
I87A
P59A
I82A
F85A
I86A
H57A
G75A
L79A
I87A
P74A
I86A
L79A
I87A
I82A
F85A
H57A
G75A
P74A
P59A
P74A
H57A
SpoIVFA
SpoIVFA
αSpoIVFA
αSpoIVFA
SpoIVFA
*
αSpoIVFA
*
*
GFPΔ27BofA
GFPΔ27BofA
GFPΔ27BofA
αGFP
αGFP
αGFP
**
**
**
cytTM-SpoIVFB
cytTM-SpoIVFB
cytTM-SpoIVFB
αHis
αHis
αHis
Pro-σK(1-127)
Pro-σK(1-127)
Pro-σK(1-127)
Cleavage product
Cleavage product
Cleavage product
Set 3
Set 1
Set 2

## Slide 3
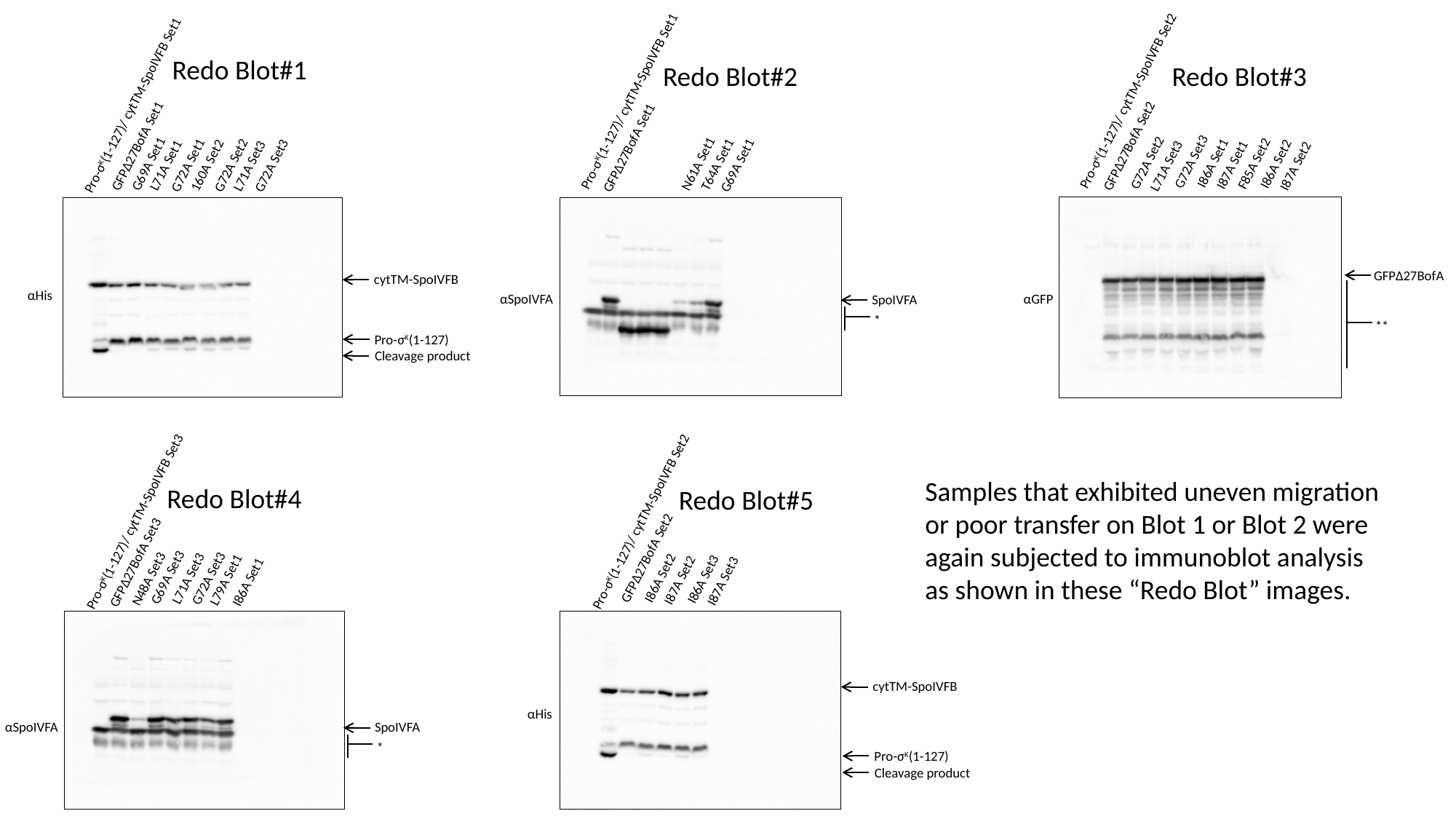

Redo Blot#1
Redo Blot#2
Redo Blot#3
Pro-σK(1-127)/ cytTM-SpoIVFB Set2
Pro-σK(1-127)/ cytTM-SpoIVFB Set1
Pro-σK(1-127)/ cytTM-SpoIVFB Set1
GFPΔ27BofA Set2
GFPΔ27BofA Set1
GFPΔ27BofA Set1
160A Set2
G72A Set2
G72A Set1
G72A Set3
N61A Set1
L71A Set1
L71A Set3
I86A Set1
G72A Set3
I86A Set2
I87A Set1
F85A Set2
G72A Set2
I87A Set2
G69A Set1
T64A Set1
L71A Set3
G69A Set1
GFPΔ27BofA
cytTM-SpoIVFB
αHis
αSpoIVFA
αGFP
SpoIVFA
*
**
Pro-σK(1-127)
Cleavage product
Samples that exhibited uneven migration or poor transfer on Blot 1 or Blot 2 were again subjected to immunoblot analysis as shown in these “Redo Blot” images.
Redo Blot#4
Redo Blot#5
Pro-σK(1-127)/ cytTM-SpoIVFB Set3
Pro-σK(1-127)/ cytTM-SpoIVFB Set2
GFPΔ27BofA Set2
GFPΔ27BofA Set3
G72A Set3
L71A Set3
N48A Set3
I86A Set2
I86A Set1
L79A Set1
I86A Set3
I87A Set2
I87A Set3
G69A Set3
cytTM-SpoIVFB
αHis
αSpoIVFA
SpoIVFA
*
Pro-σK(1-127)
Cleavage product
