# Supplementary material for: Inhibitory proteins block substrate access by occupying the active site cleft of Bacillus subtilis intramembrane protease SpoIVFB
Source: eLife. 2022 Apr 26;11:e74275. doi: 10.7554/eLife.74275 (PMC9042235; doi:10.7554/eLife.74275)
Supplement: Figure 2—figure supplement 3—source data 1. [file elife-74275-fig2-figsupp3-data1.zip › Figure 2-figure supplement 3-source data 1/fig sup 3 annotated blots.pptx]

## Slide 1
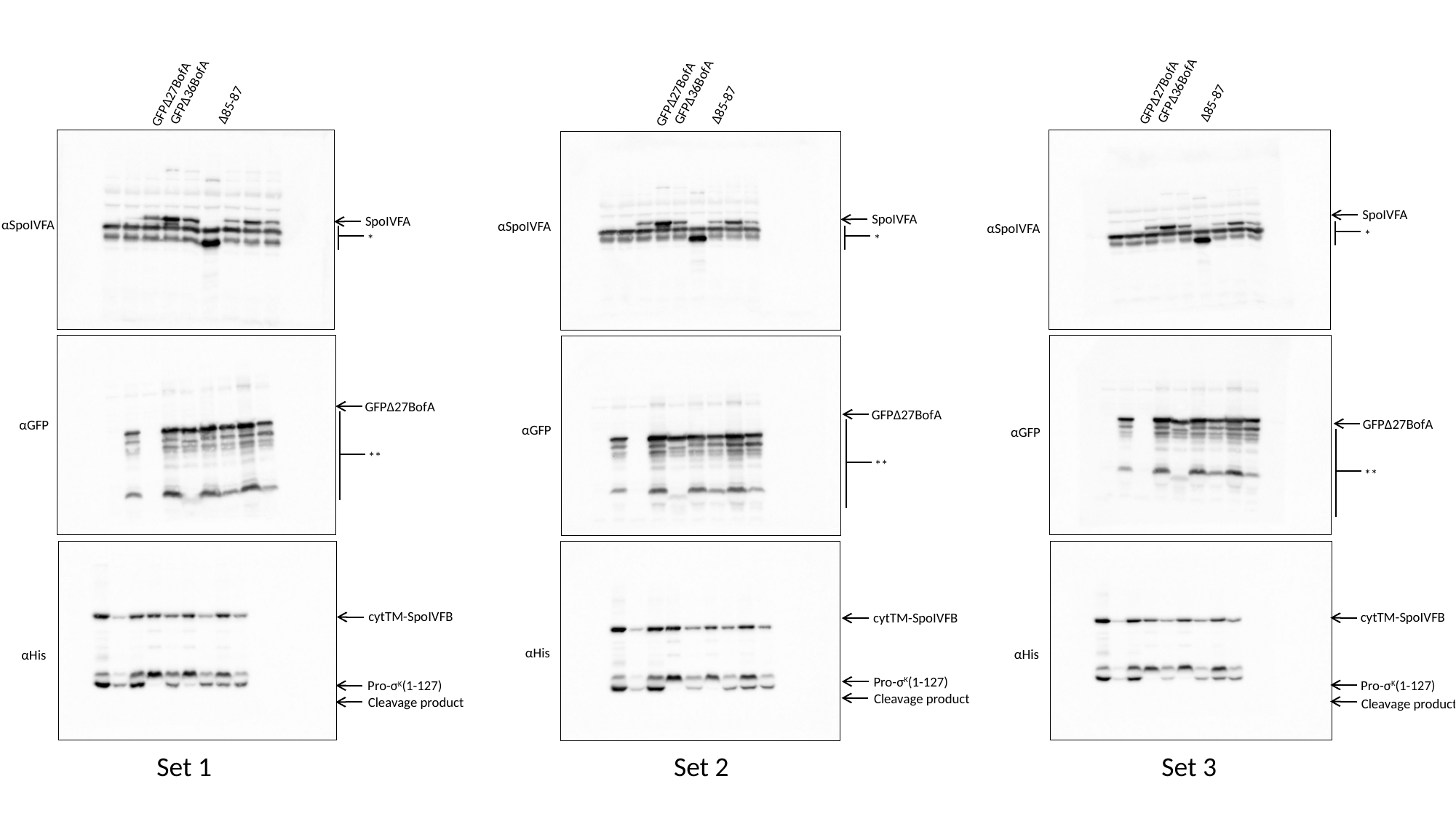

Δ85-87
GFPΔ36BofA
Δ85-87
Δ85-87
GFPΔ36BofA
GFPΔ36BofA
GFPΔ27BofA
GFPΔ27BofA
GFPΔ27BofA
SpoIVFA
SpoIVFA
SpoIVFA
αSpoIVFA
αSpoIVFA
αSpoIVFA
*
*
*
GFPΔ27BofA
GFPΔ27BofA
GFPΔ27BofA
αGFP
αGFP
αGFP
**
**
**
cytTM-SpoIVFB
cytTM-SpoIVFB
cytTM-SpoIVFB
αHis
αHis
αHis
Pro-σK(1-127)
Pro-σK(1-127)
Pro-σK(1-127)
Cleavage product
Cleavage product
Cleavage product
Set 2
Set 3
Set 1

## Slide 2
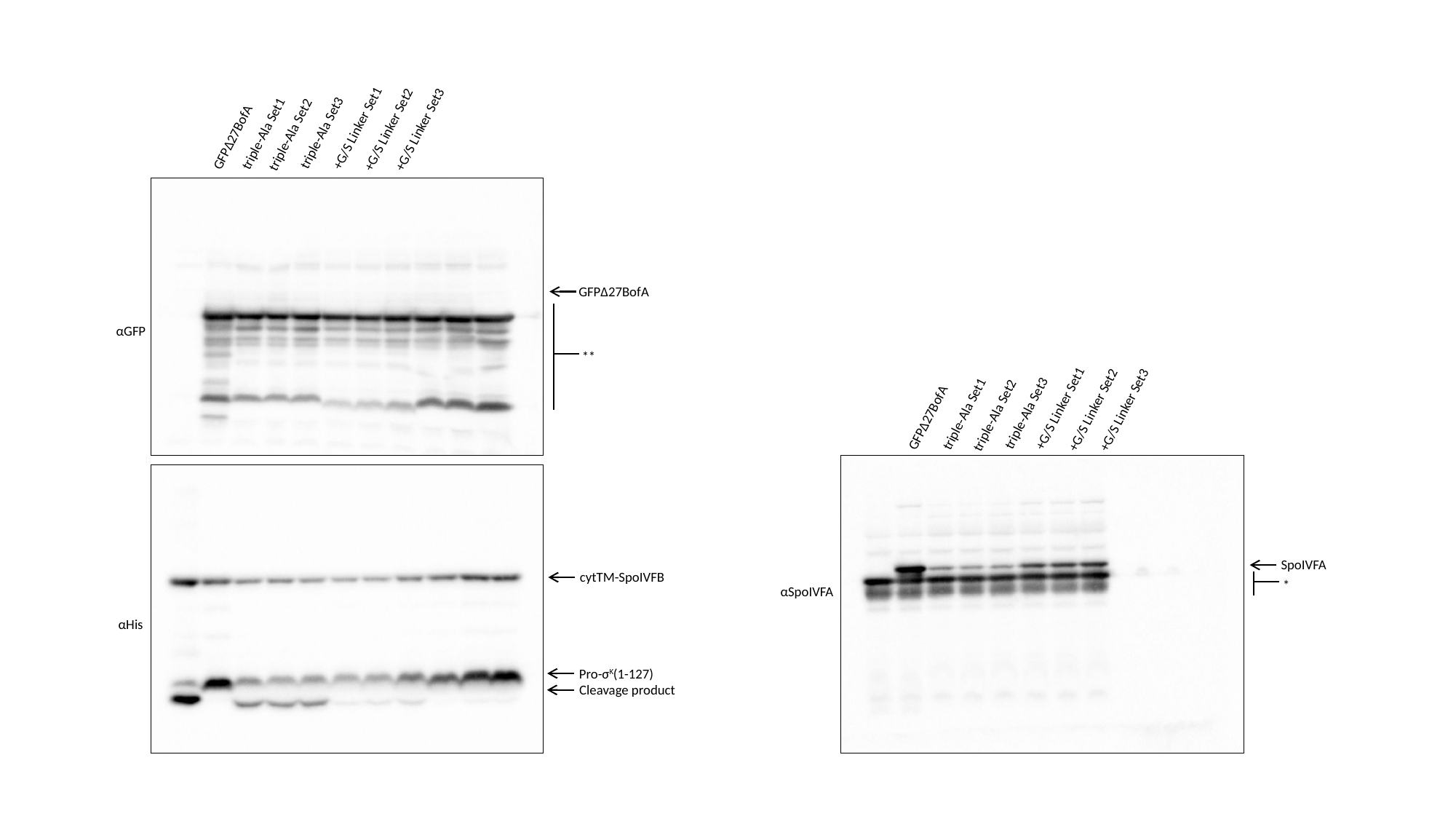

triple-Ala Set3
GFPΔ27BofA
triple-Ala Set1
+G/S Linker Set1
+G/S Linker Set2
+G/S Linker Set3
triple-Ala Set2
GFPΔ27BofA
αGFP
**
triple-Ala Set3
GFPΔ27BofA
triple-Ala Set1
+G/S Linker Set1
+G/S Linker Set2
+G/S Linker Set3
triple-Ala Set2
SpoIVFA
cytTM-SpoIVFB
*
αSpoIVFA
αHis
Pro-σK(1-127)
Cleavage product
