# Supplementary material for: Inhibitory proteins block substrate access by occupying the active site cleft of Bacillus subtilis intramembrane protease SpoIVFB
Source: eLife. 2022 Apr 26;11:e74275. doi: 10.7554/eLife.74275 (PMC9042235; doi:10.7554/eLife.74275)
Supplement: Figure 3—source data 1. [file elife-74275-fig3-data1.zip › Figure 3-source data 1/Figure 3A/Fig3A annotated blots.pptx]

## Slide 1
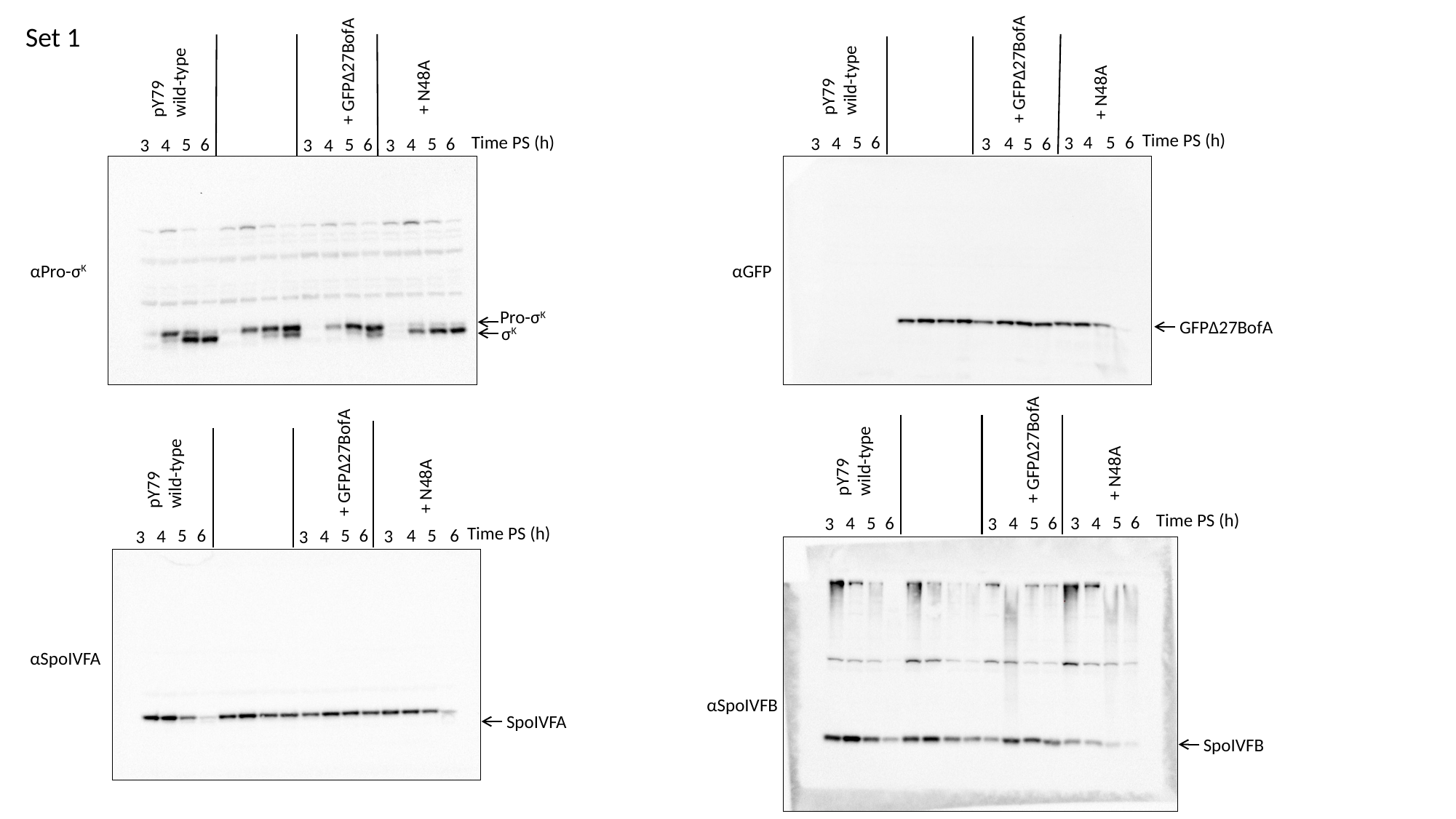

Set 1
+ GFPΔ27BofA
pY79
wild-type
+ GFPΔ27BofA
pY79
wild-type
+ N48A
+ N48A
Time PS (h)
Time PS (h)
6
5
4
6
5
3
4
4
3
3
6
5
6
5
4
6
5
6
5
3
4
4
3
3
αPro-σK
αGFP
Pro-σK
GFPΔ27BofA
σK
+ GFPΔ27BofA
pY79
wild-type
+ GFPΔ27BofA
pY79
wild-type
+ N48A
+ N48A
Time PS (h)
6
5
4
6
5
6
5
3
4
4
3
3
Time PS (h)
6
5
4
6
5
6
5
3
4
4
3
3
αSpoIVFA
αSpoIVFB
SpoIVFA
SpoIVFB

## Slide 2
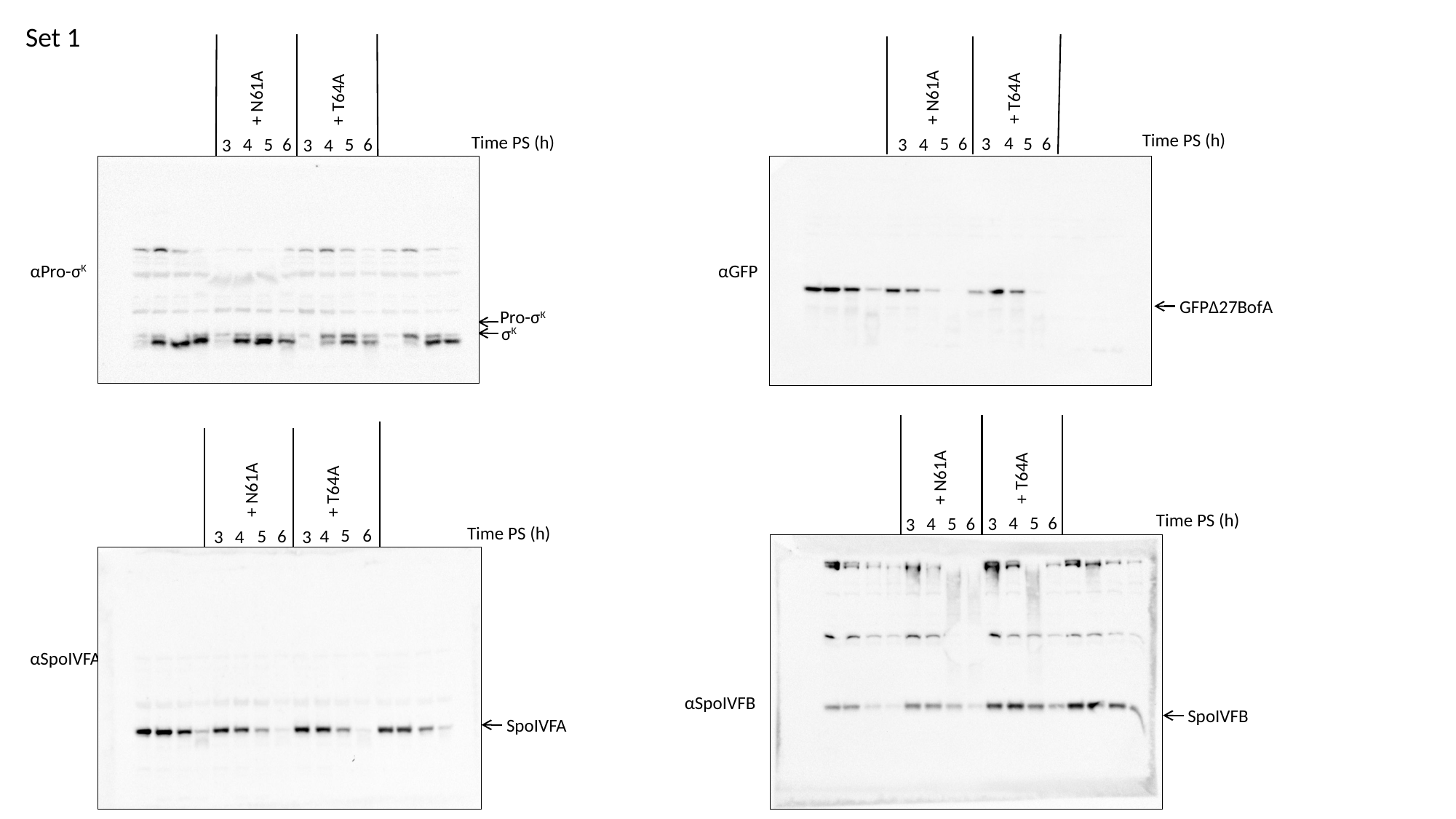

Set 1
+ N61A
+ T64A
+ N61A
+ T64A
Time PS (h)
Time PS (h)
4
3
6
5
6
5
6
5
4
6
5
4
3
4
3
3
αPro-σK
αGFP
GFPΔ27BofA
Pro-σK
σK
+ N61A
+ T64A
+ N61A
+ T64A
Time PS (h)
6
5
4
6
5
3
4
3
Time PS (h)
6
5
6
5
4
4
3
3
αSpoIVFA
αSpoIVFB
SpoIVFB
SpoIVFA

## Slide 3
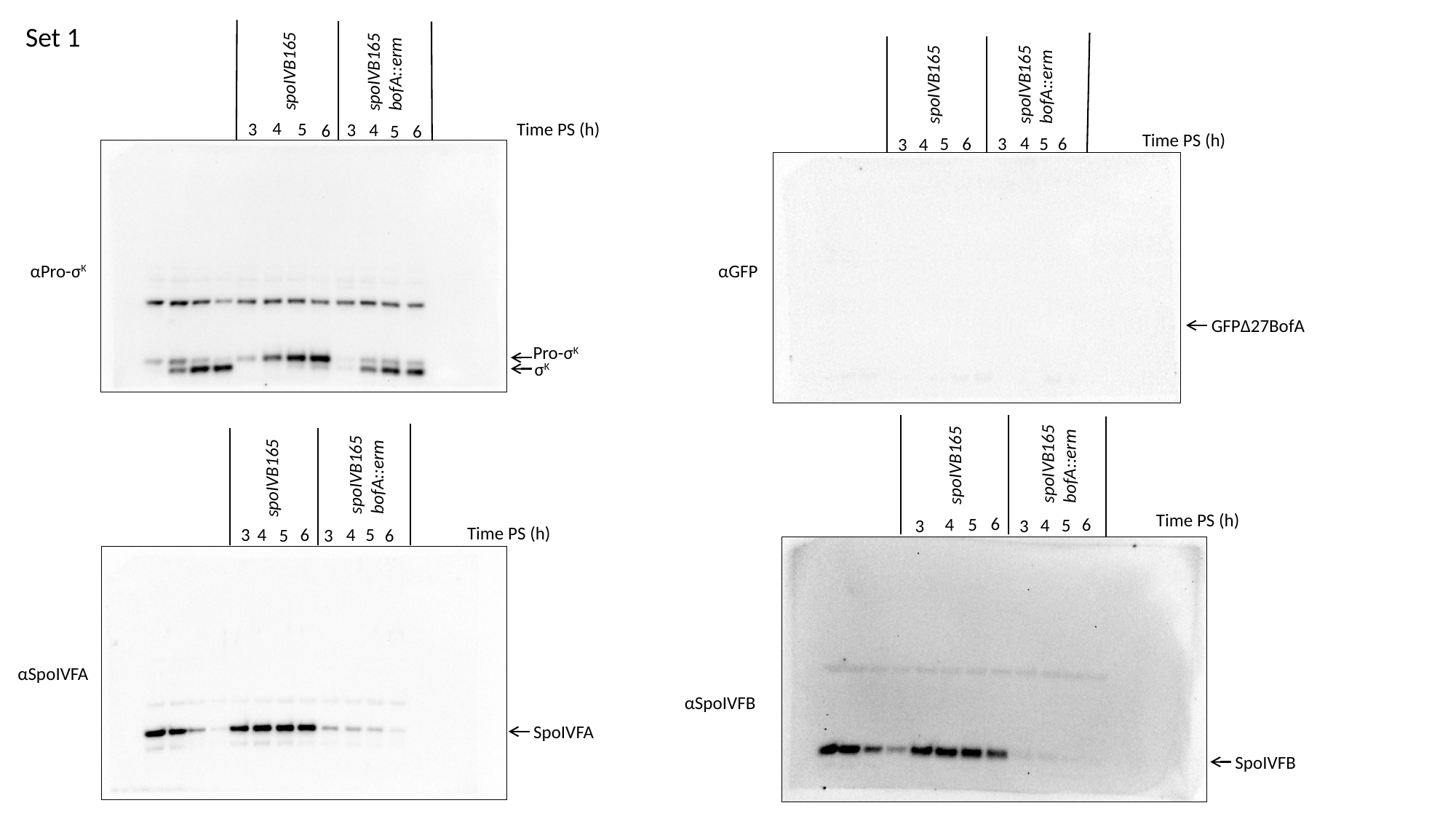

Set 1
spoIVB165
bofA::erm
spoIVB165
spoIVB165
bofA::erm
spoIVB165
4
3
Time PS (h)
5
4
3
6
6
5
Time PS (h)
4
3
6
5
6
5
4
3
αPro-σK
αGFP
GFPΔ27BofA
Pro-σK
σK
spoIVB165
bofA::erm
spoIVB165
bofA::erm
spoIVB165
spoIVB165
Time PS (h)
6
5
6
4
4
5
3
3
Time PS (h)
3
4
6
4
5
3
5
6
αSpoIVFA
αSpoIVFB
SpoIVFA
SpoIVFB

## Slide 4
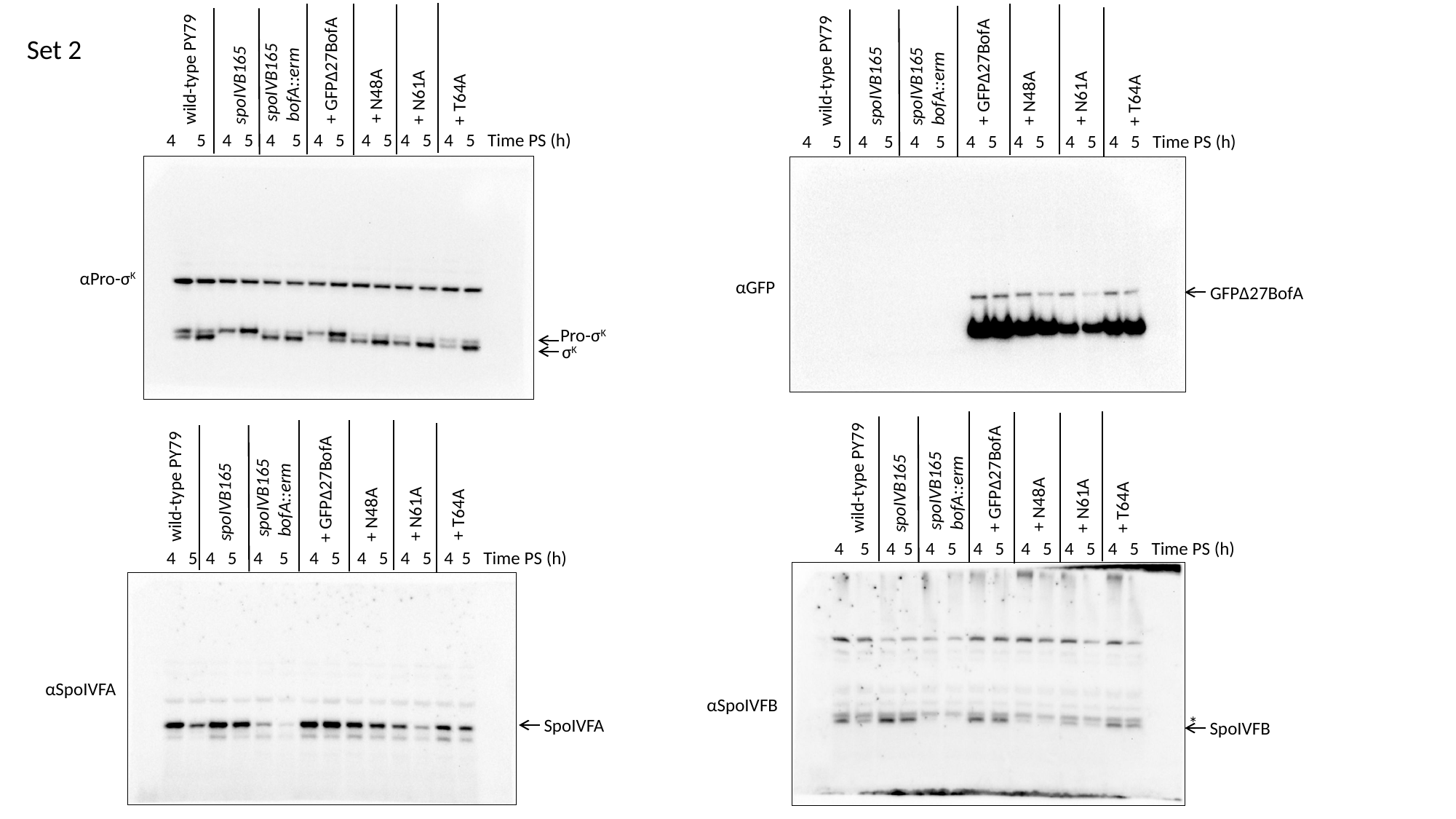

wild-type PY79
wild-type PY79
+ GFPΔ27BofA
Set 2
+ GFPΔ27BofA
spoIVB165
bofA::erm
spoIVB165
bofA::erm
spoIVB165
spoIVB165
+ N48A
+ N61A
+ N61A
+ N48A
+ T64A
+ T64A
 4 5 4 5 4 5 4 5 4 5 4 5 4 5 Time PS (h)
 4 5 4 5 4 5 4 5 4 5 4 5 4 5 Time PS (h)
αPro-σK
αGFP
GFPΔ27BofA
Pro-σK
σK
wild-type PY79
+ GFPΔ27BofA
wild-type PY79
+ GFPΔ27BofA
spoIVB165
bofA::erm
spoIVB165
bofA::erm
spoIVB165
spoIVB165
+ N48A
+ N61A
+ T64A
+ N61A
+ T64A
+ N48A
 4 5 4 5 4 5 4 5 4 5 4 5 4 5 Time PS (h)
 4 5 4 5 4 5 4 5 4 5 4 5 4 5 Time PS (h)
αSpoIVFA
αSpoIVFB
*
SpoIVFA
SpoIVFB

## Slide 5
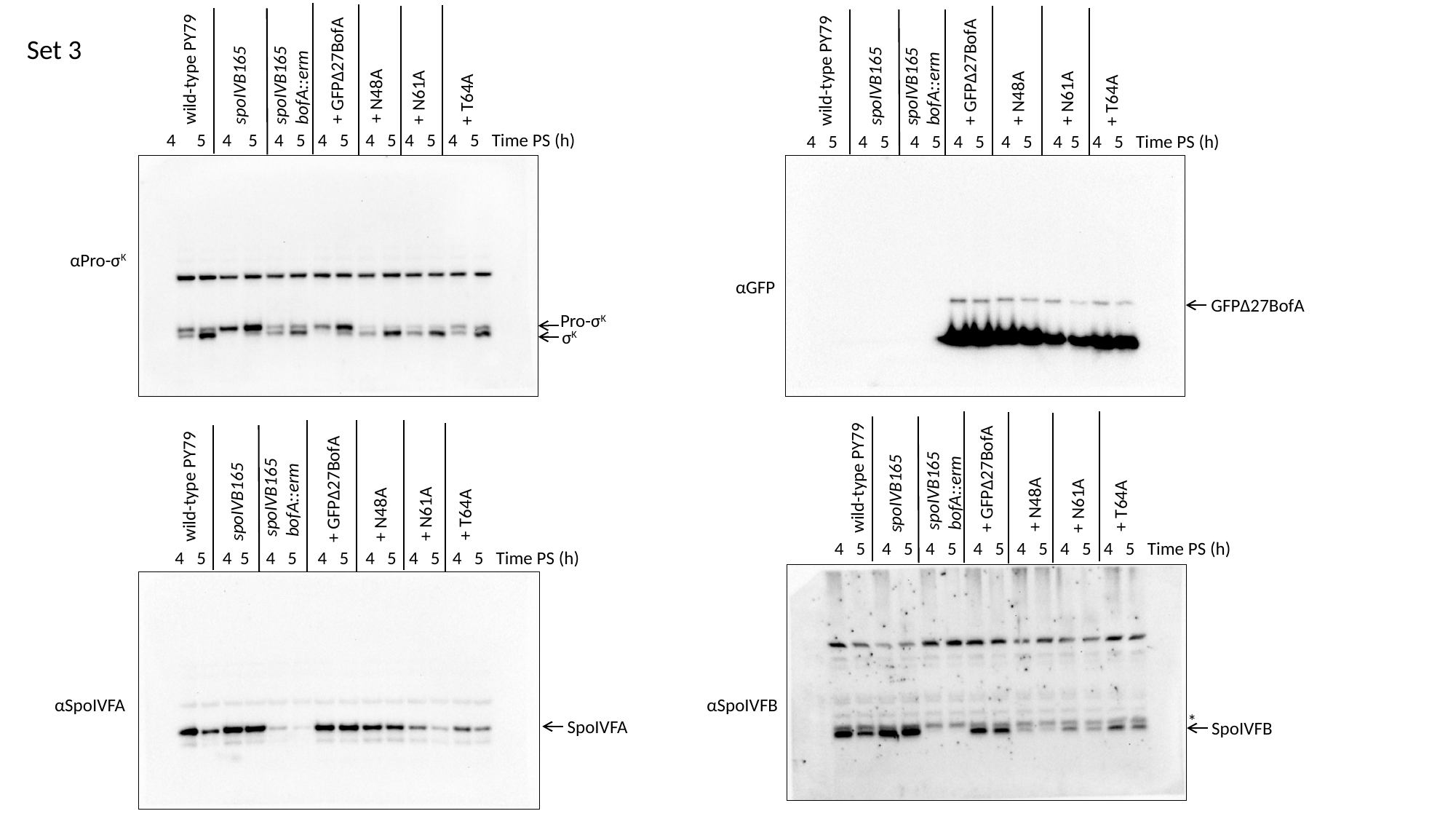

wild-type PY79
wild-type PY79
+ GFPΔ27BofA
Set 3
+ GFPΔ27BofA
spoIVB165
bofA::erm
spoIVB165
bofA::erm
spoIVB165
spoIVB165
+ N48A
+ N61A
+ N61A
+ N48A
+ T64A
+ T64A
 4 5 4 5 4 5 4 5 4 5 4 5 4 5 Time PS (h)
 4 5 4 5 4 5 4 5 4 5 4 5 4 5 Time PS (h)
αPro-σK
αGFP
GFPΔ27BofA
Pro-σK
σK
wild-type PY79
+ GFPΔ27BofA
wild-type PY79
+ GFPΔ27BofA
spoIVB165
bofA::erm
spoIVB165
bofA::erm
spoIVB165
spoIVB165
+ N48A
+ N61A
+ T64A
+ N61A
+ T64A
+ N48A
 4 5 4 5 4 5 4 5 4 5 4 5 4 5 Time PS (h)
 4 5 4 5 4 5 4 5 4 5 4 5 4 5 Time PS (h)
αSpoIVFA
αSpoIVFB
*
SpoIVFA
SpoIVFB
