# Supplementary material for: Inhibitory proteins block substrate access by occupying the active site cleft of Bacillus subtilis intramembrane protease SpoIVFB
Source: eLife. 2022 Apr 26;11:e74275. doi: 10.7554/eLife.74275 (PMC9042235; doi:10.7554/eLife.74275)
Supplement: Figure 3—source data 1. [file elife-74275-fig3-data1.zip › Figure 3-source data 1/Figure 3B images/readme.docx]

Three files are associated with each confocal microscopy image – a TIF file that is a stack of the green (GFP) and red (membrane) fluorescence, which can be viewed and manipulated in ImageJ (1), and two JPG files with optimized green (GFP) and red (membrane) fluorescence for easy viewing.

Reference

1. Rasband WS (1997-2018) ImageJ. *U. S. National Institutes of Health, Bethesda, Maryland, USA* <http://imagej.nih.gov/ij/>.
