# Supplementary figures and images for: Inhibitory proteins block substrate access by occupying the active site cleft of Bacillus subtilis intramembrane protease SpoIVFB (part 2 of 3)
Source: eLife. 2022 Apr 26;11:e74275. doi: 10.7554/eLife.74275 (PMC9042235; doi:10.7554/eLife.74275)

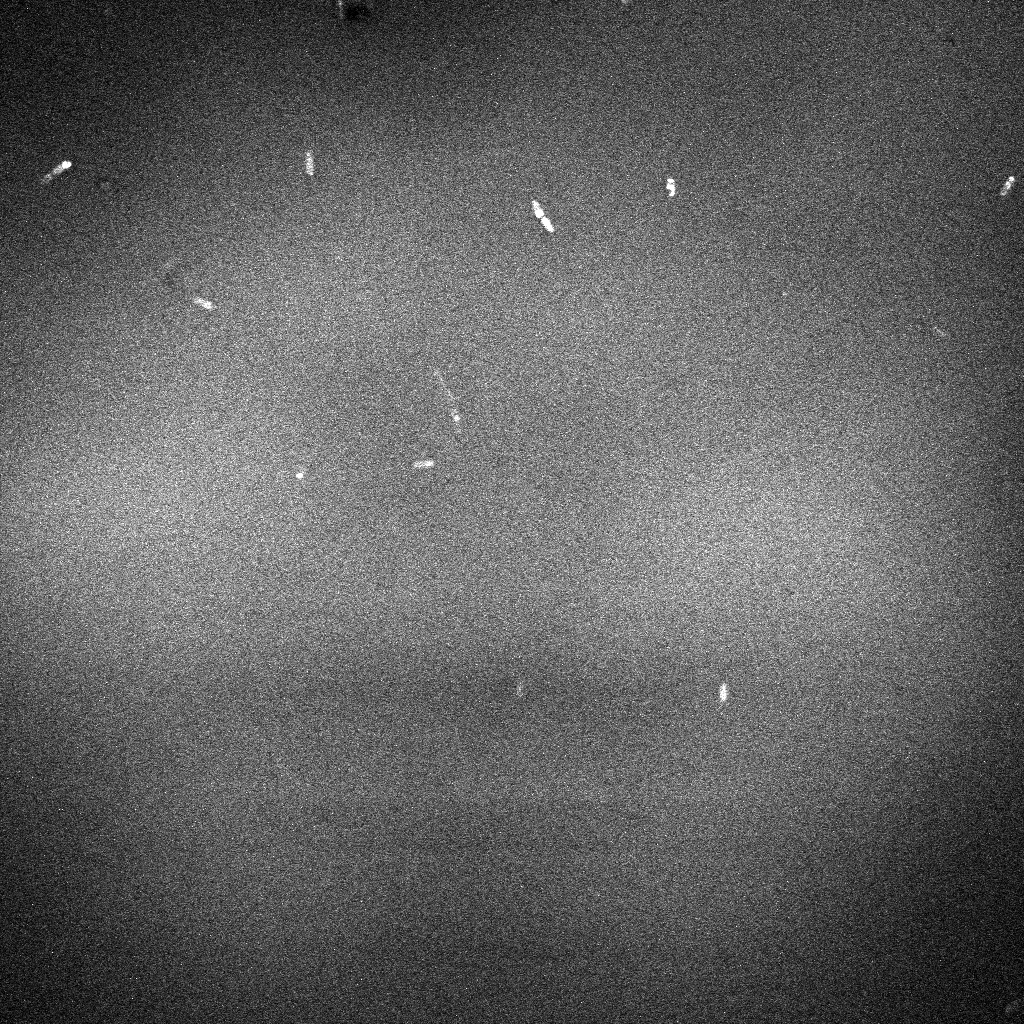

Supplement: Figure 3—source data 1. [file elife-74275-fig3-data1.zip › Figure 3-source data 1/Figure 3B images/T64A 3 hrs 1.tif]

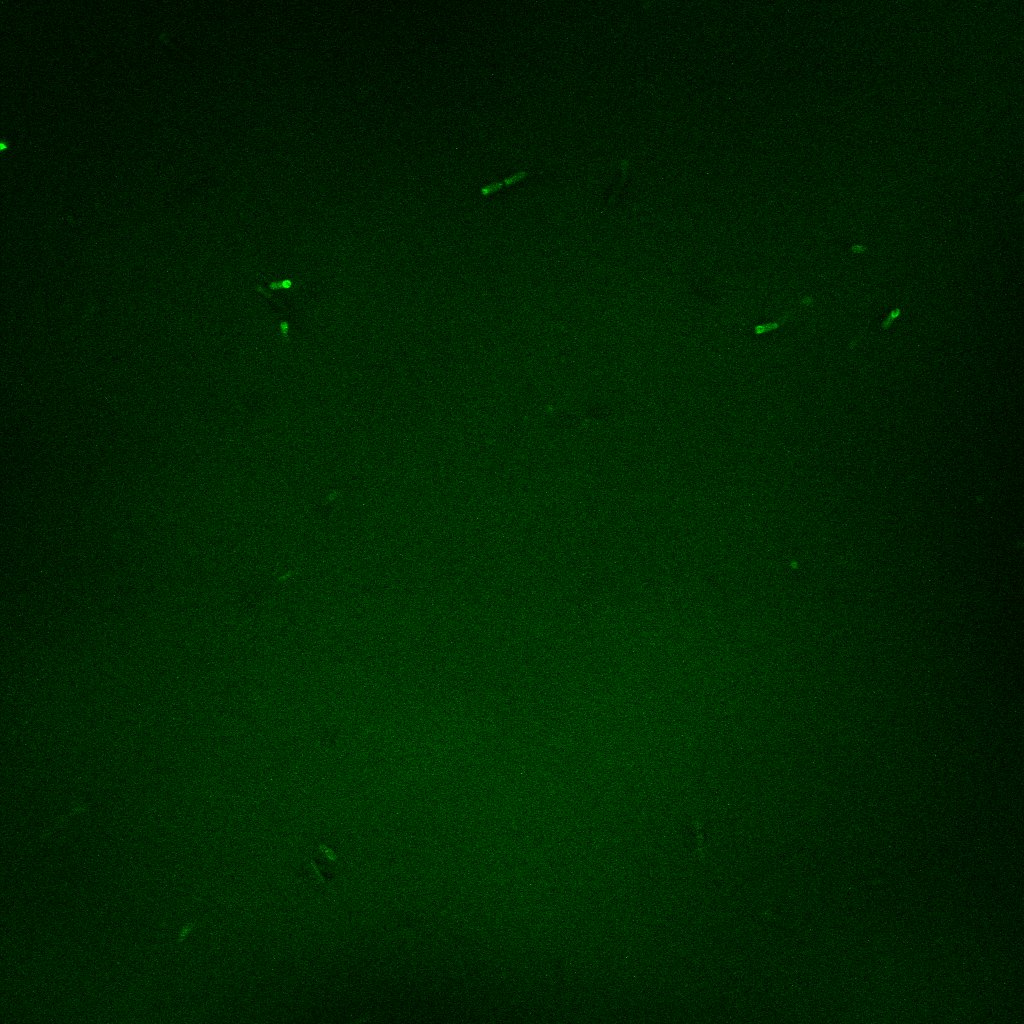

Supplement: Figure 3—source data 1. [file elife-74275-fig3-data1.zip › Figure 3-source data 1/Figure 3B images/T64A 3 hrs 2 GFP.jpg]

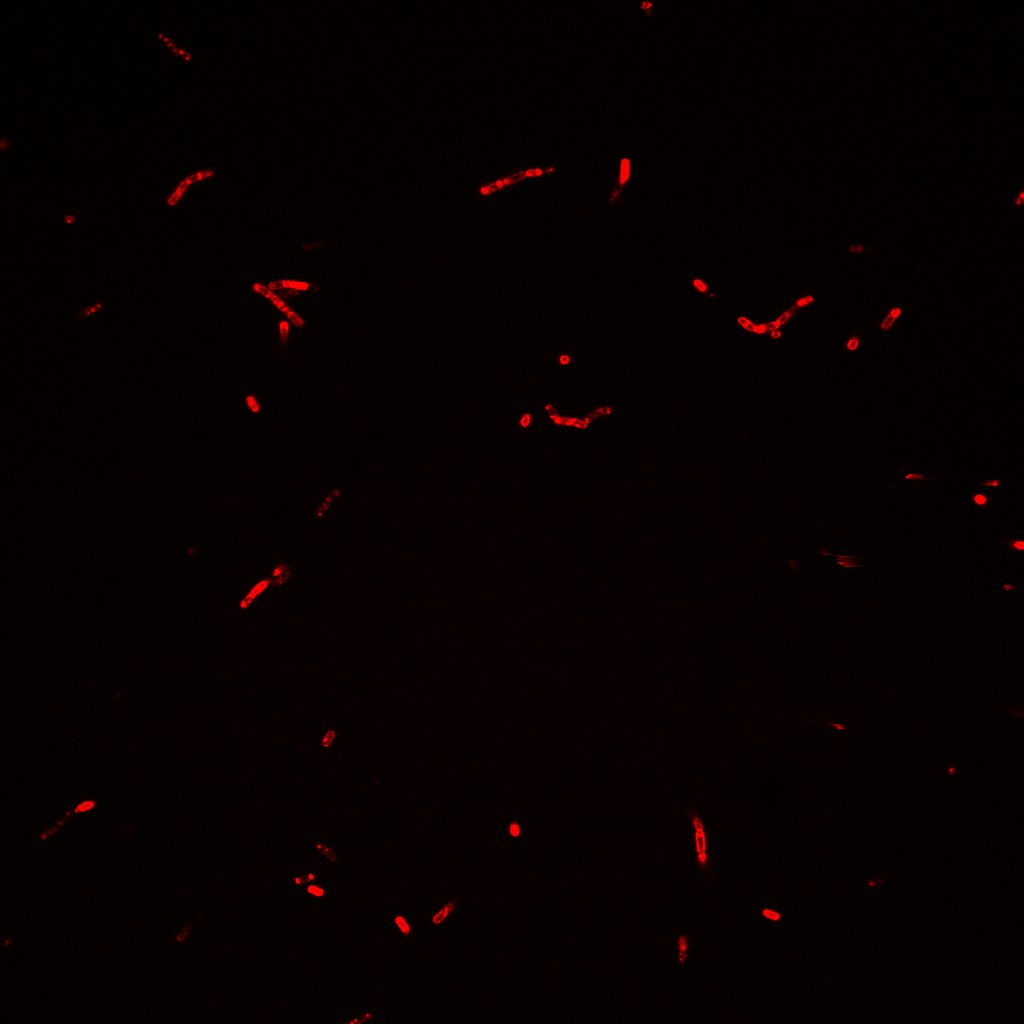

Supplement: Figure 3—source data 1. [file elife-74275-fig3-data1.zip › Figure 3-source data 1/Figure 3B images/T64A 3 hrs 2 membrane.jpg]

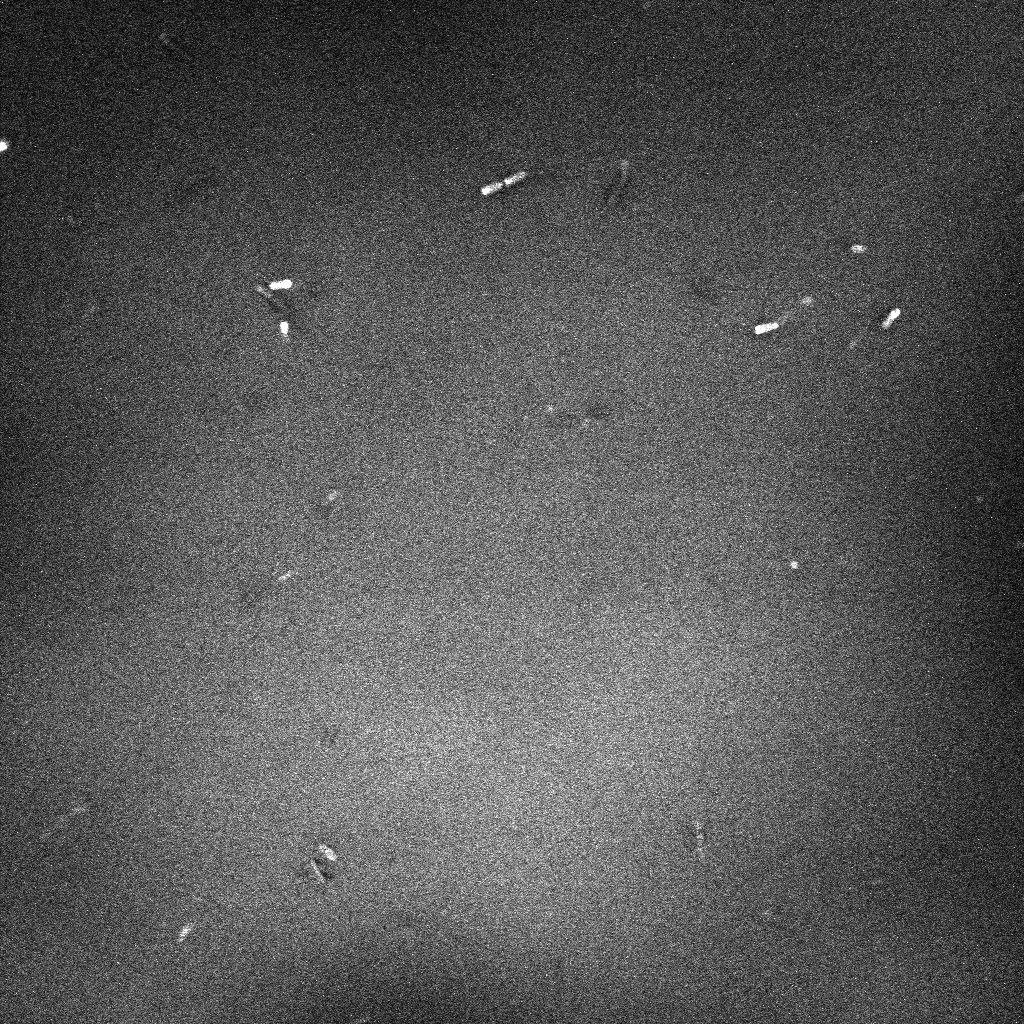

Supplement: Figure 3—source data 1. [file elife-74275-fig3-data1.zip › Figure 3-source data 1/Figure 3B images/T64A 3 hrs 2.tif]

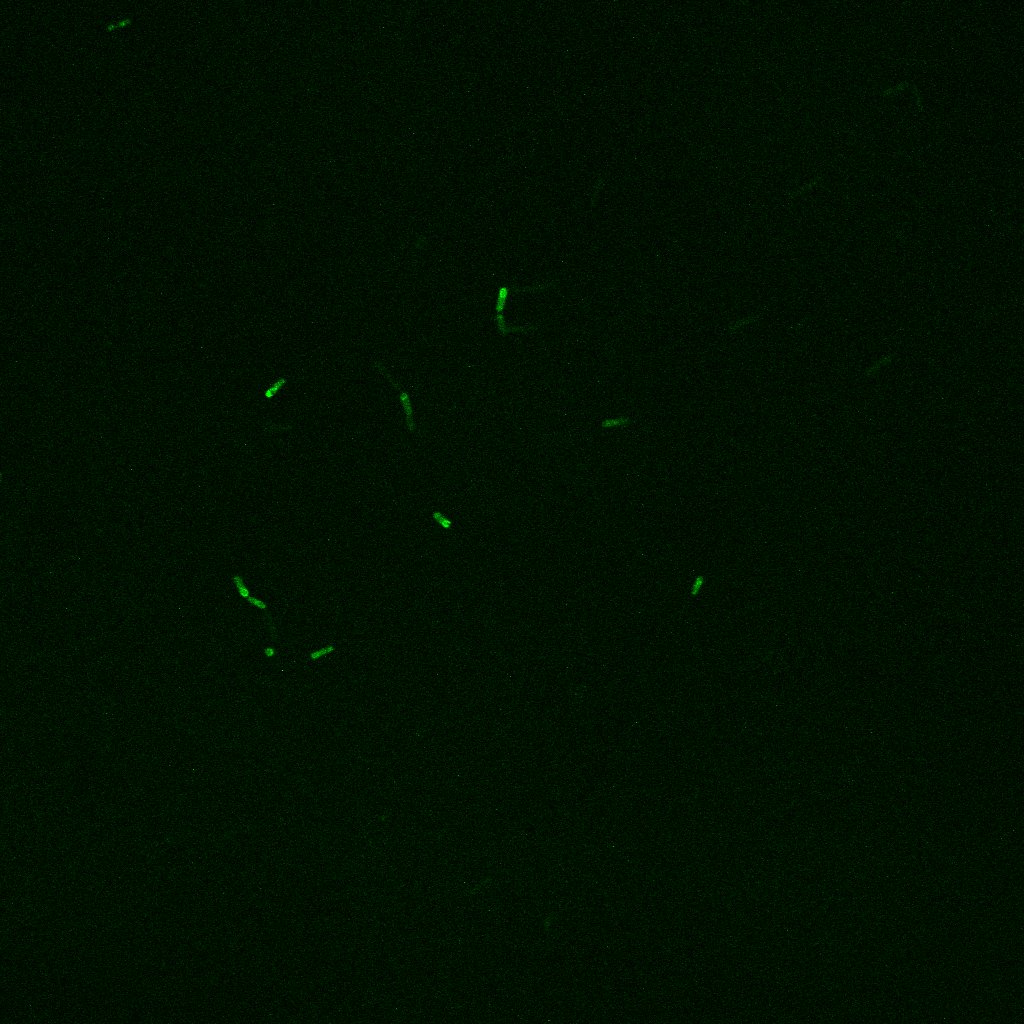

Supplement: Figure 3—source data 1. [file elife-74275-fig3-data1.zip › Figure 3-source data 1/Figure 3B images/T64A 3 hrs 3 GFP.jpg]

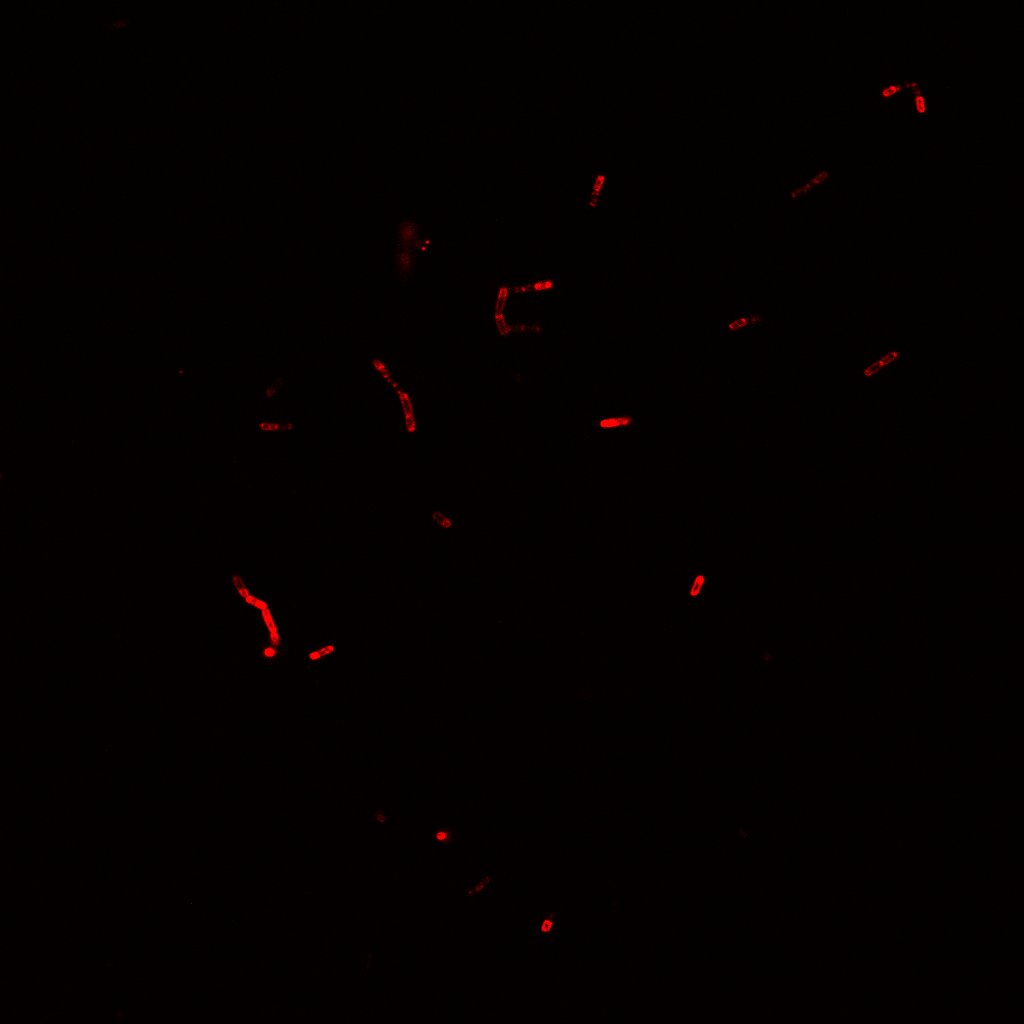

Supplement: Figure 3—source data 1. [file elife-74275-fig3-data1.zip › Figure 3-source data 1/Figure 3B images/T64A 3 hrs 3 membrane.jpg]

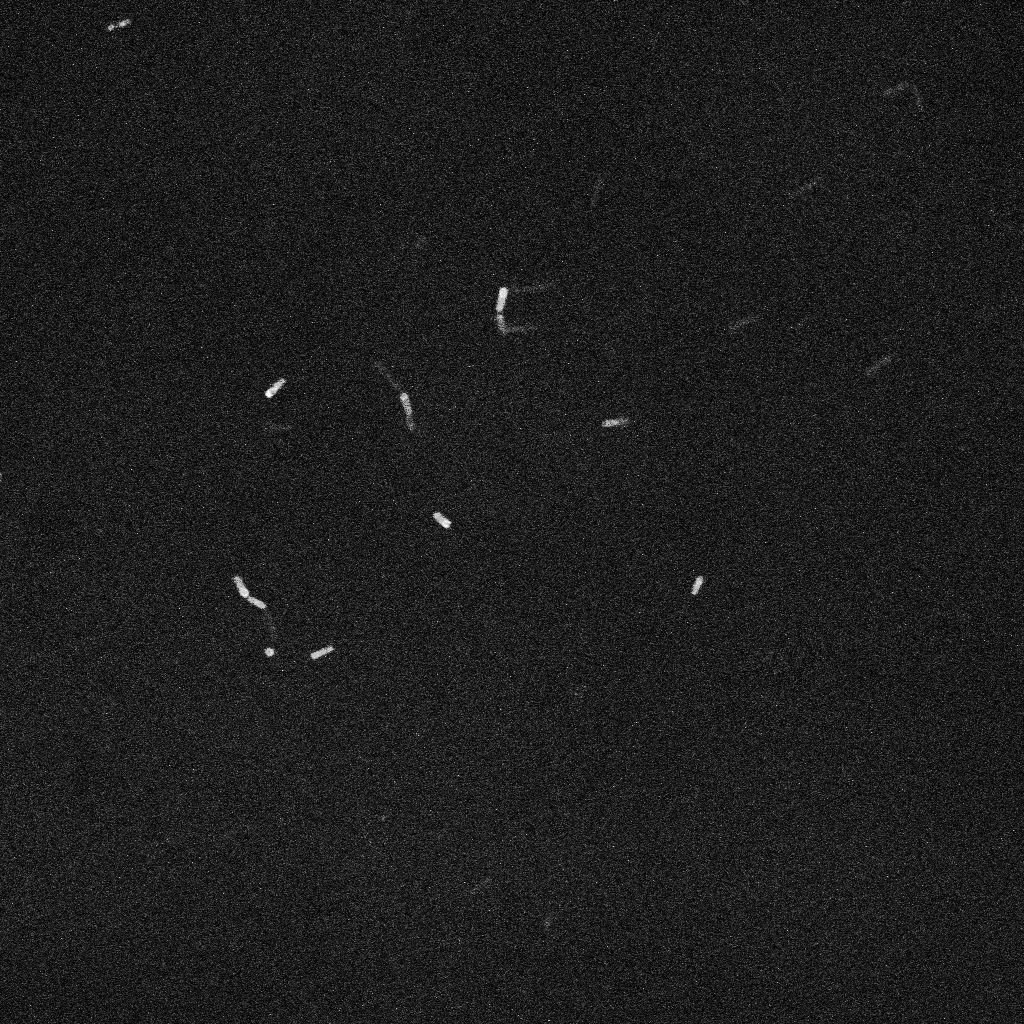

Supplement: Figure 3—source data 1. [file elife-74275-fig3-data1.zip › Figure 3-source data 1/Figure 3B images/T64A 3 hrs 3.tif]

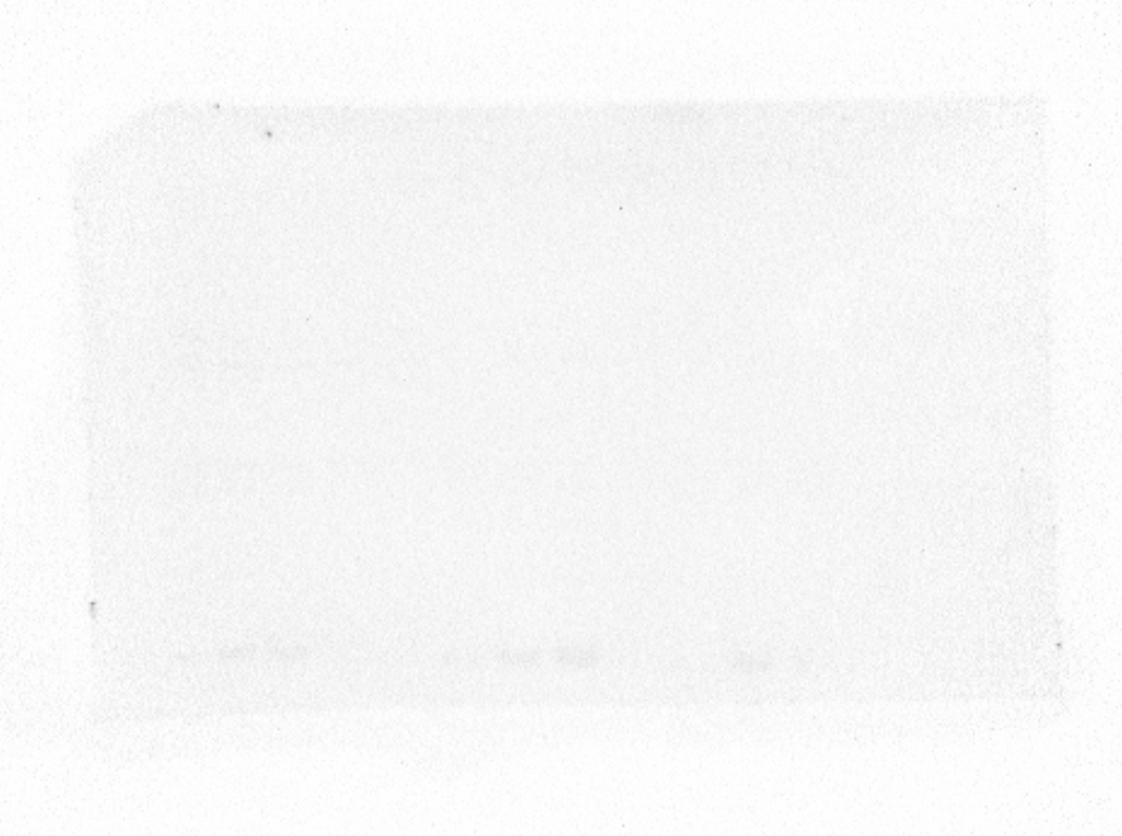

Supplement: Figure 3—source data 2. [file elife-74275-fig3-data2.zip › Figure 3-source data 2/Fig3A Set1 anti-GFP (BK754 and ZR264).tif]

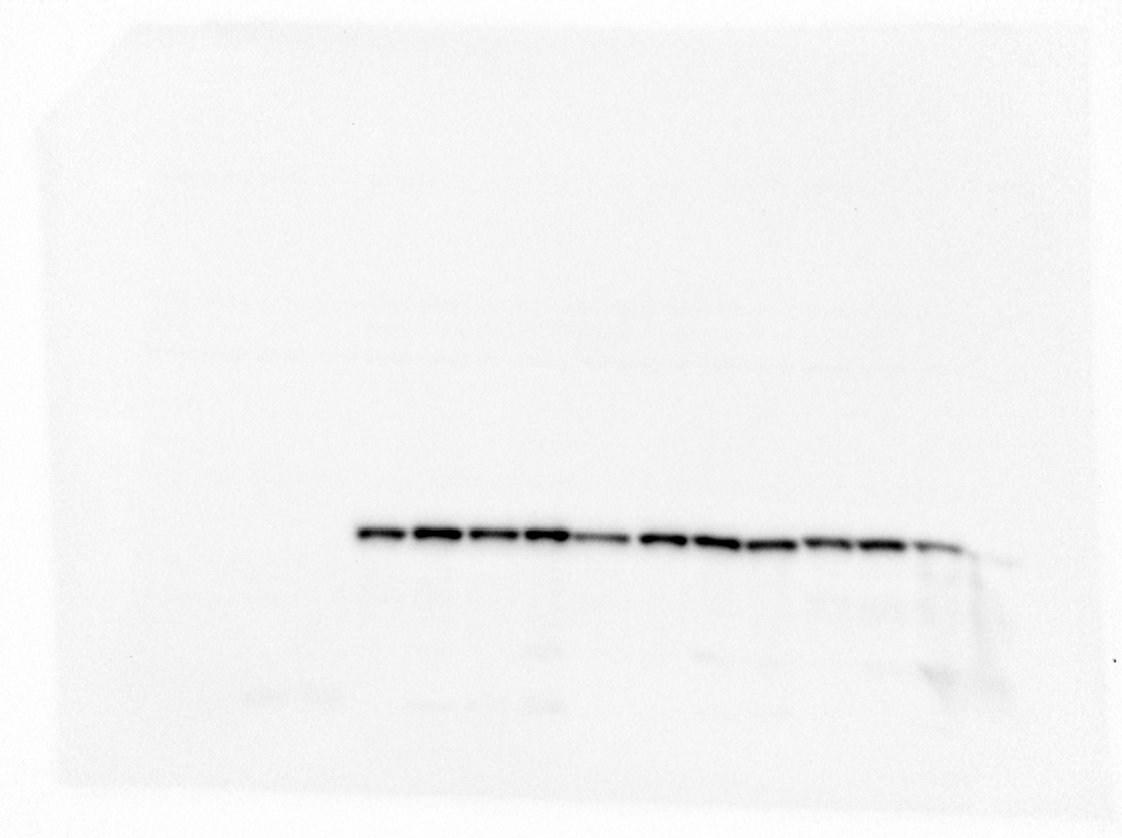

Supplement: Figure 3—source data 2. [file elife-74275-fig3-data2.zip › Figure 3-source data 2/Fig3A Set1 anti-GFP (PY79_ SO3_ SO6).tif]

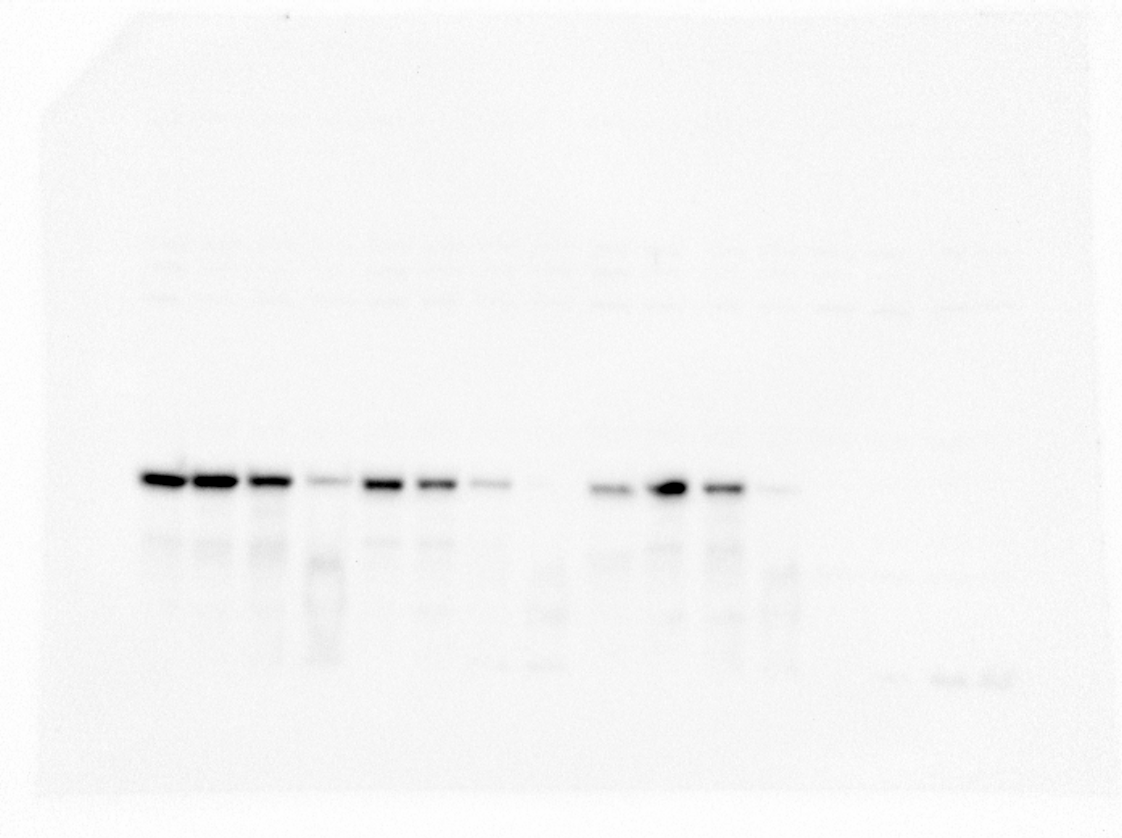

Supplement: Figure 3—source data 2. [file elife-74275-fig3-data2.zip › Figure 3-source data 2/Fig3A Set1 anti-GFP (SO8 and SO10).tif]

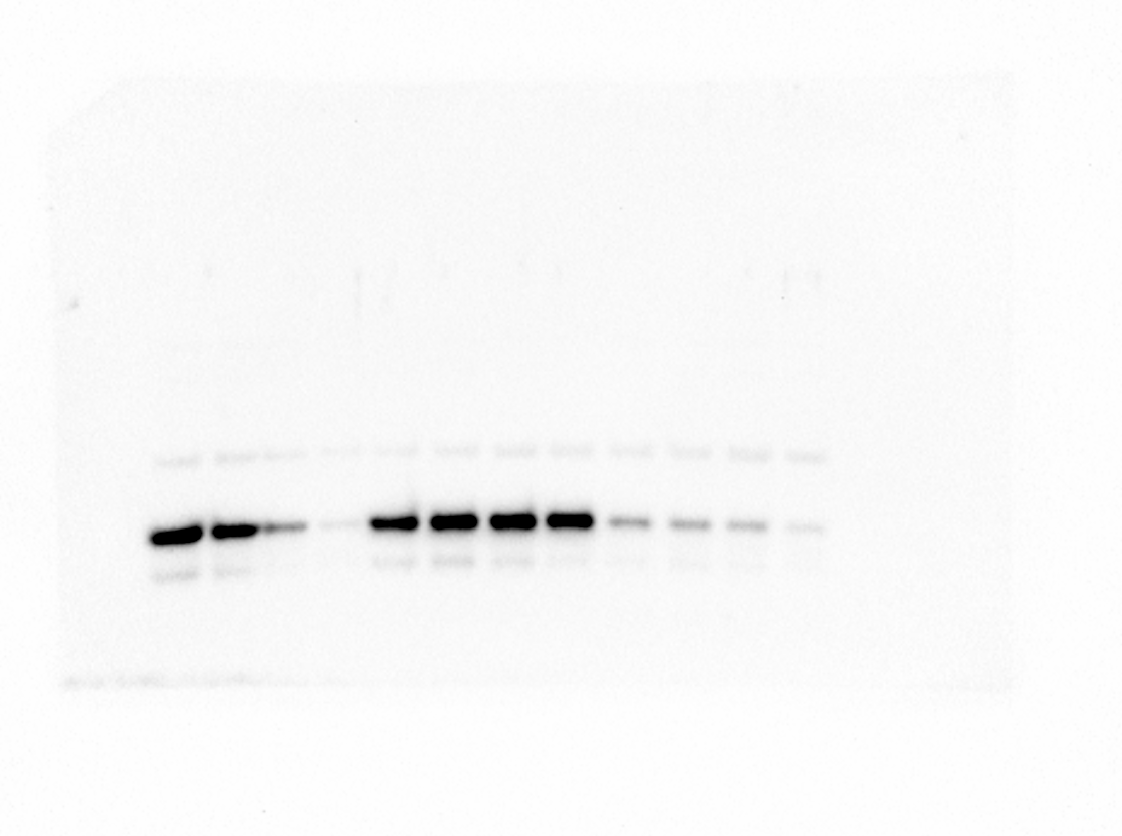

Supplement: Figure 3—source data 2. [file elife-74275-fig3-data2.zip › Figure 3-source data 2/Fig3A Set1 anti-IVFA (BK754 and ZR264).tif]

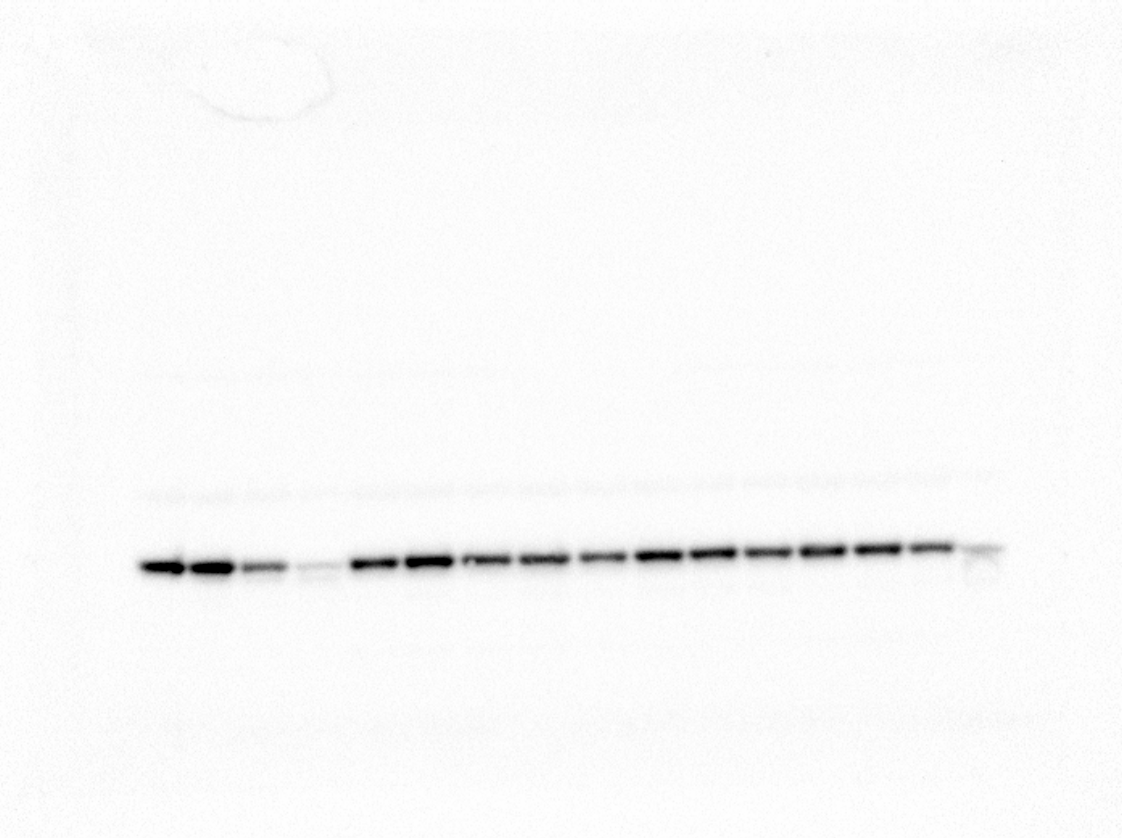

Supplement: Figure 3—source data 2. [file elife-74275-fig3-data2.zip › Figure 3-source data 2/Fig3A Set1 anti-IVFA (PY79_ SO3_ SO6).tif]

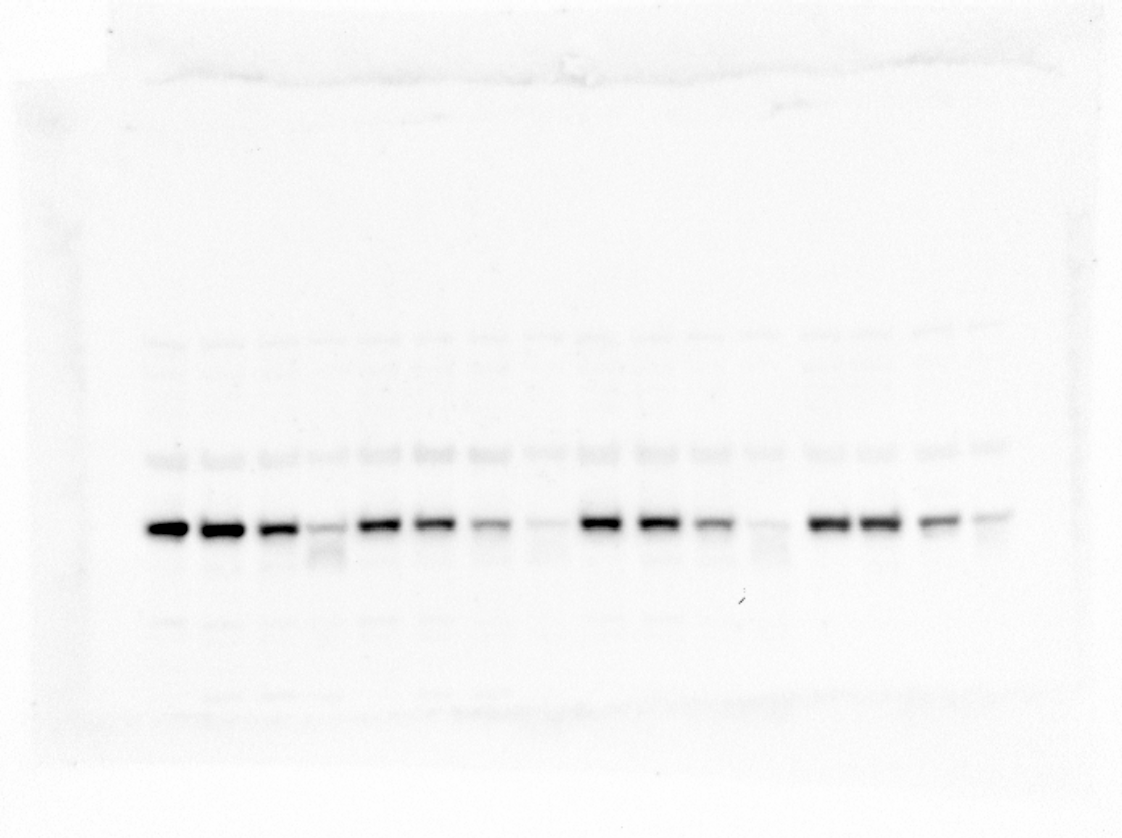

Supplement: Figure 3—source data 2. [file elife-74275-fig3-data2.zip › Figure 3-source data 2/Fig3A Set1 anti-IVFA (SO8 and SO10).tif]

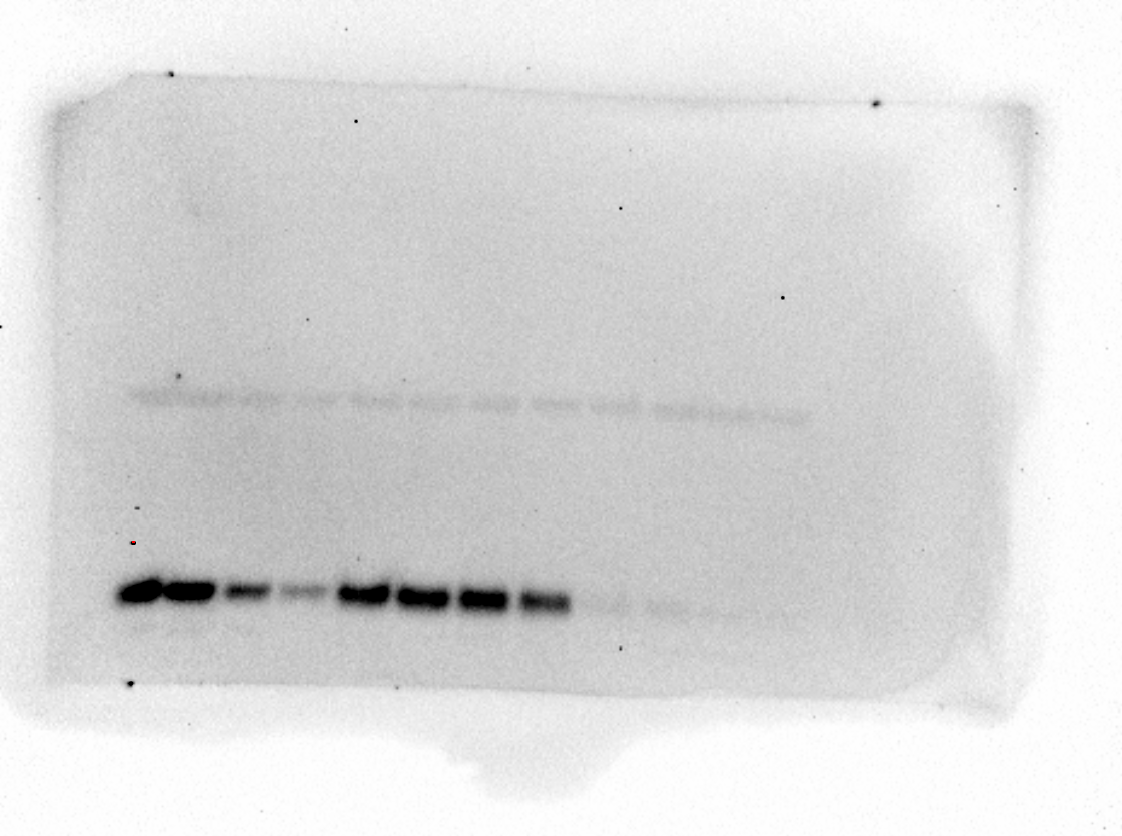

Supplement: Figure 3—source data 2. [file elife-74275-fig3-data2.zip › Figure 3-source data 2/Fig3A Set1 anti-IVFB (BK754 and ZR264).tif]

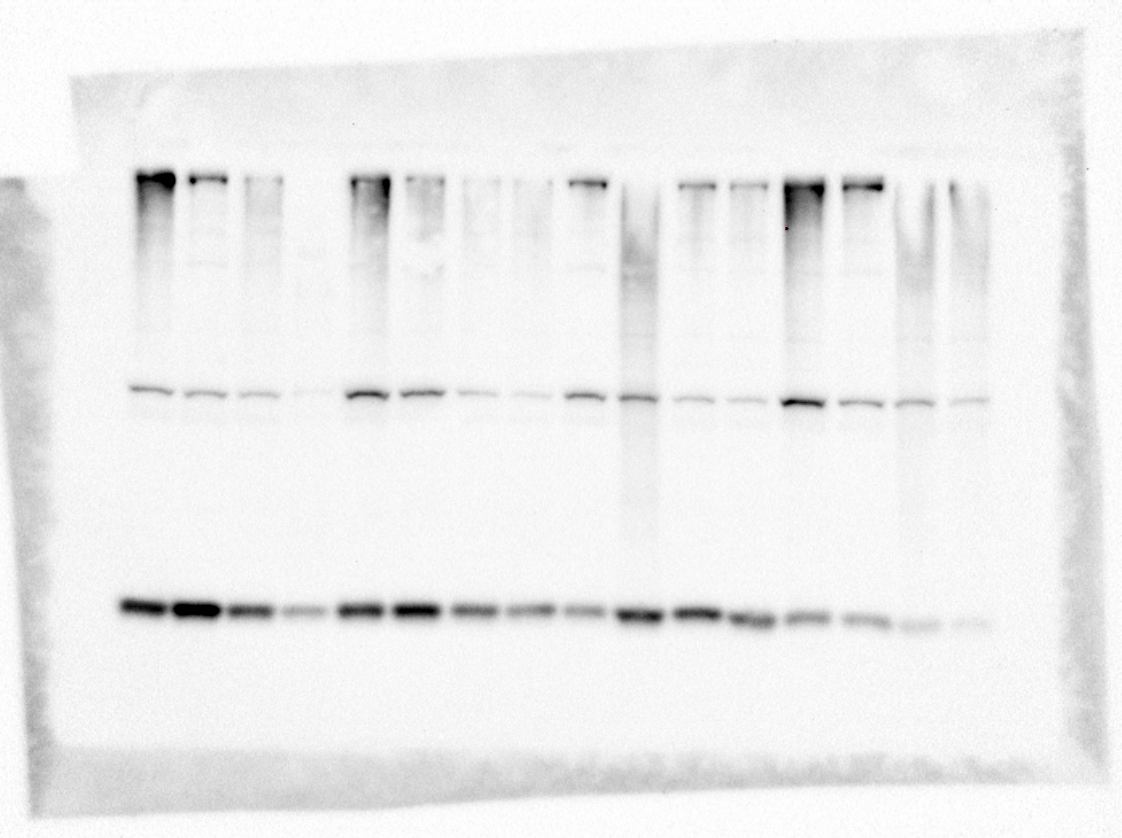

Supplement: Figure 3—source data 2. [file elife-74275-fig3-data2.zip › Figure 3-source data 2/Fig3A Set1 anti-IVFB (PY79_ SO3_ SO6).tif]

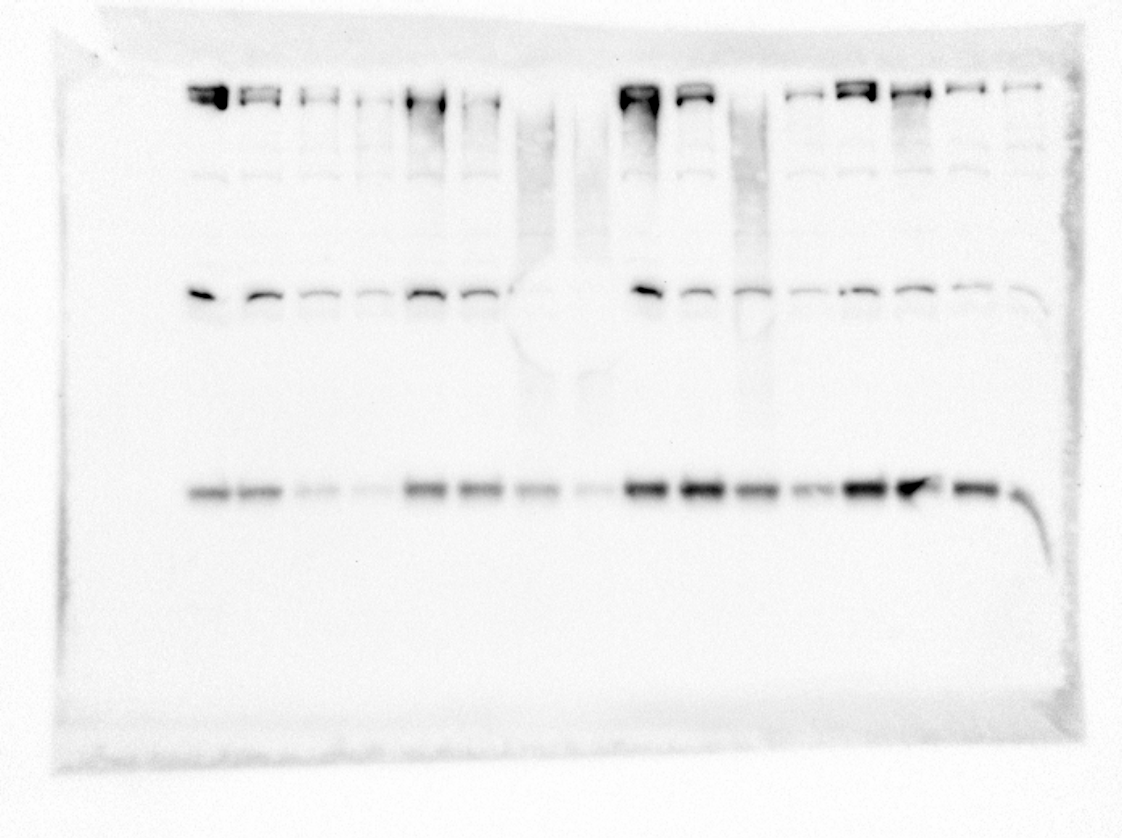

Supplement: Figure 3—source data 2. [file elife-74275-fig3-data2.zip › Figure 3-source data 2/Fig3A Set1 anti-IVFB (SO8 and SO10).tif]

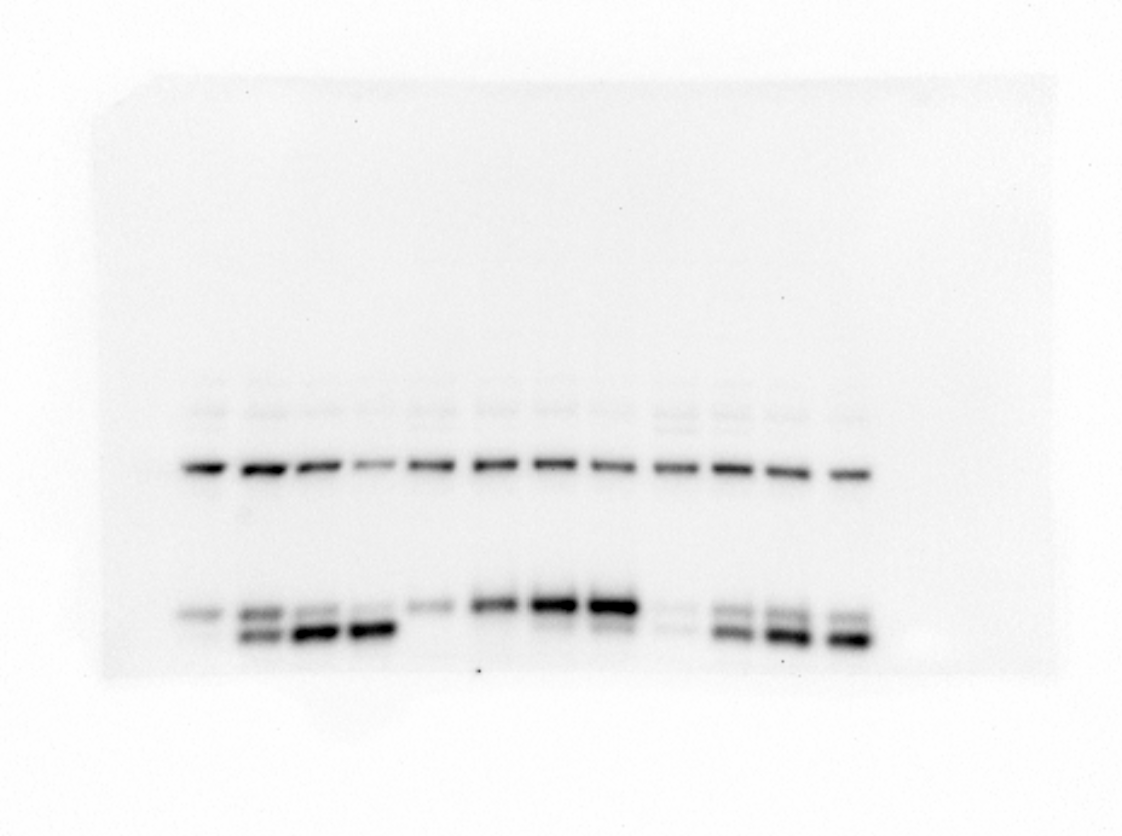

Supplement: Figure 3—source data 2. [file elife-74275-fig3-data2.zip › Figure 3-source data 2/Fig3A Set1 anti-sigK (BK754 and ZR264).tif]

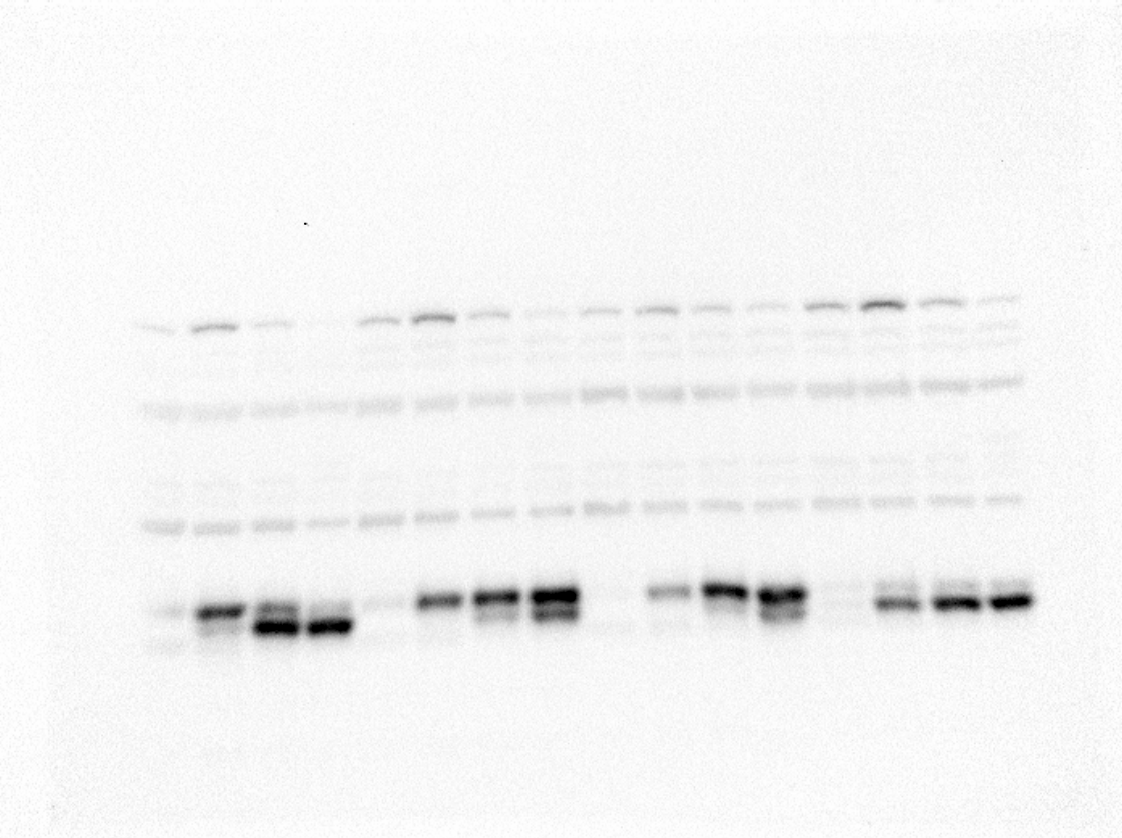

Supplement: Figure 3—source data 2. [file elife-74275-fig3-data2.zip › Figure 3-source data 2/Fig3A Set1 anti-sigK (PY79_ SO3_ SO6).tif]

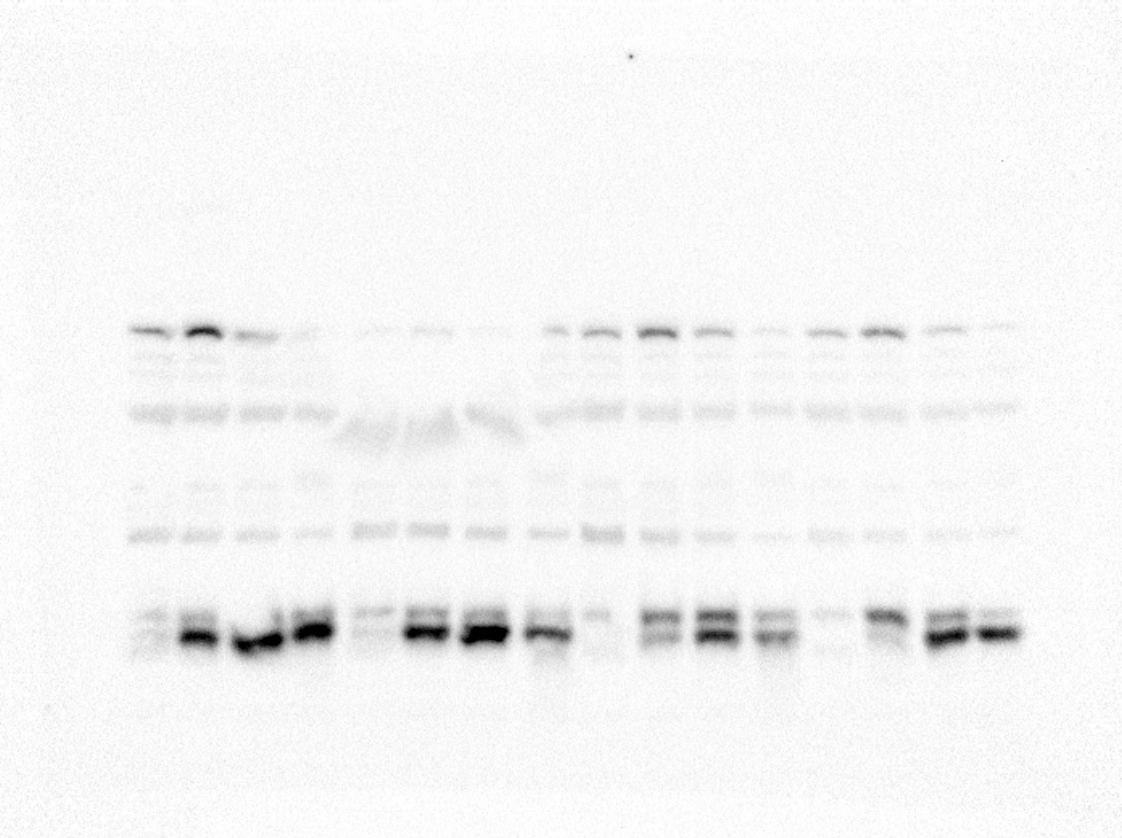

Supplement: Figure 3—source data 2. [file elife-74275-fig3-data2.zip › Figure 3-source data 2/Fig3A Set1 anti-sigK (SO8 and SO10).tif]

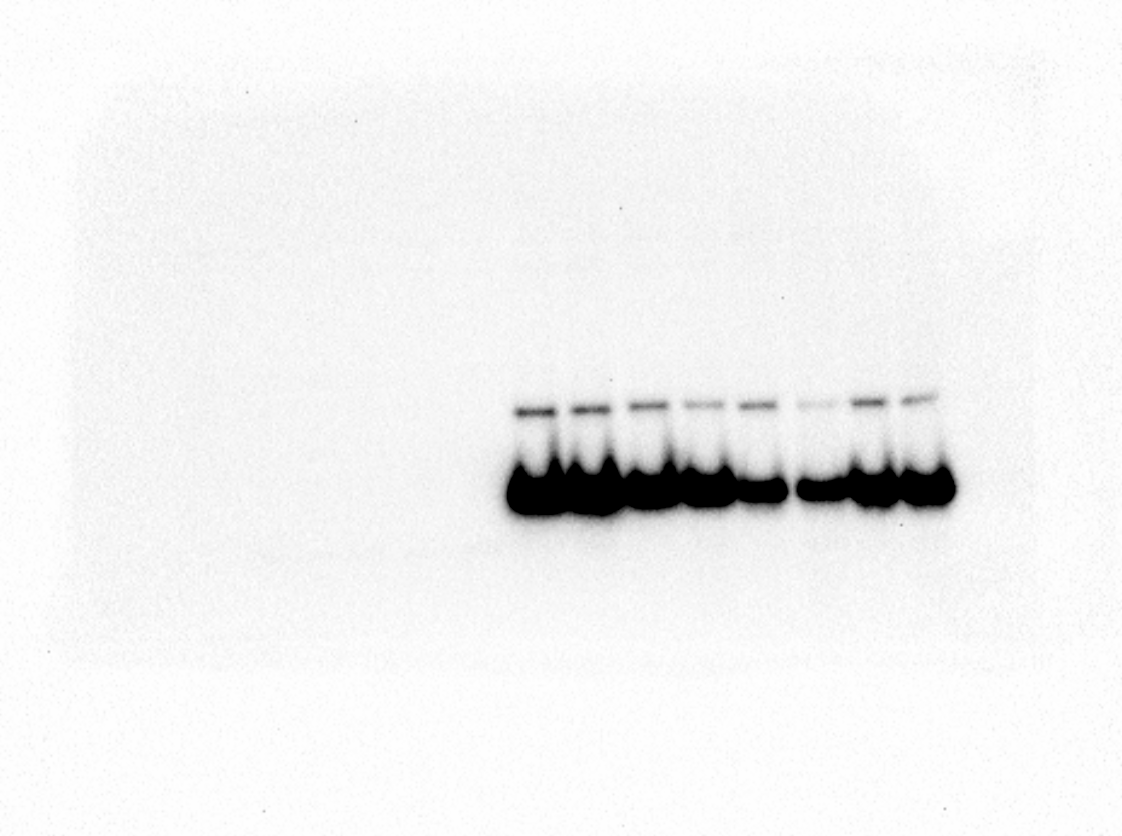

Supplement: Figure 3—source data 2. [file elife-74275-fig3-data2.zip › Figure 3-source data 2/Fig3A Set2 anti-GFP.tif]

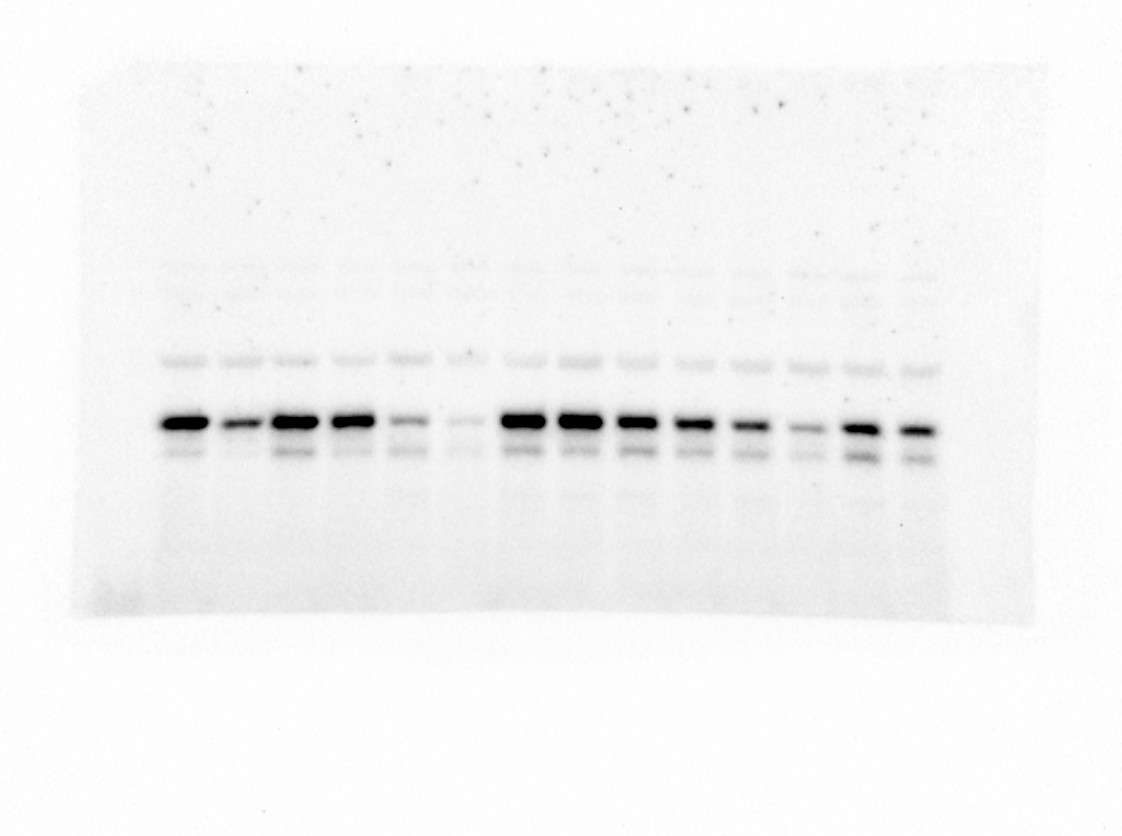

Supplement: Figure 3—source data 2. [file elife-74275-fig3-data2.zip › Figure 3-source data 2/Fig3A Set2 anti-IVFA.tif]

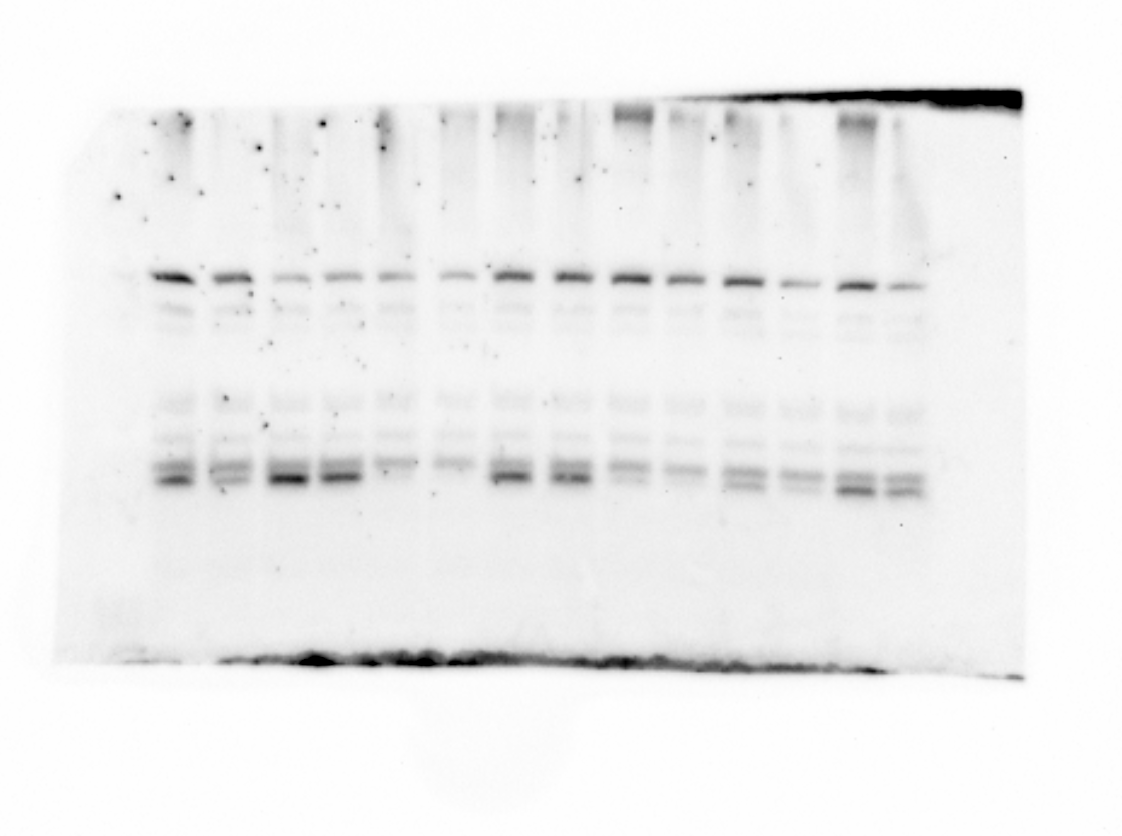

Supplement: Figure 3—source data 2. [file elife-74275-fig3-data2.zip › Figure 3-source data 2/Fig3A Set2 anti-IVFB 60.tif]

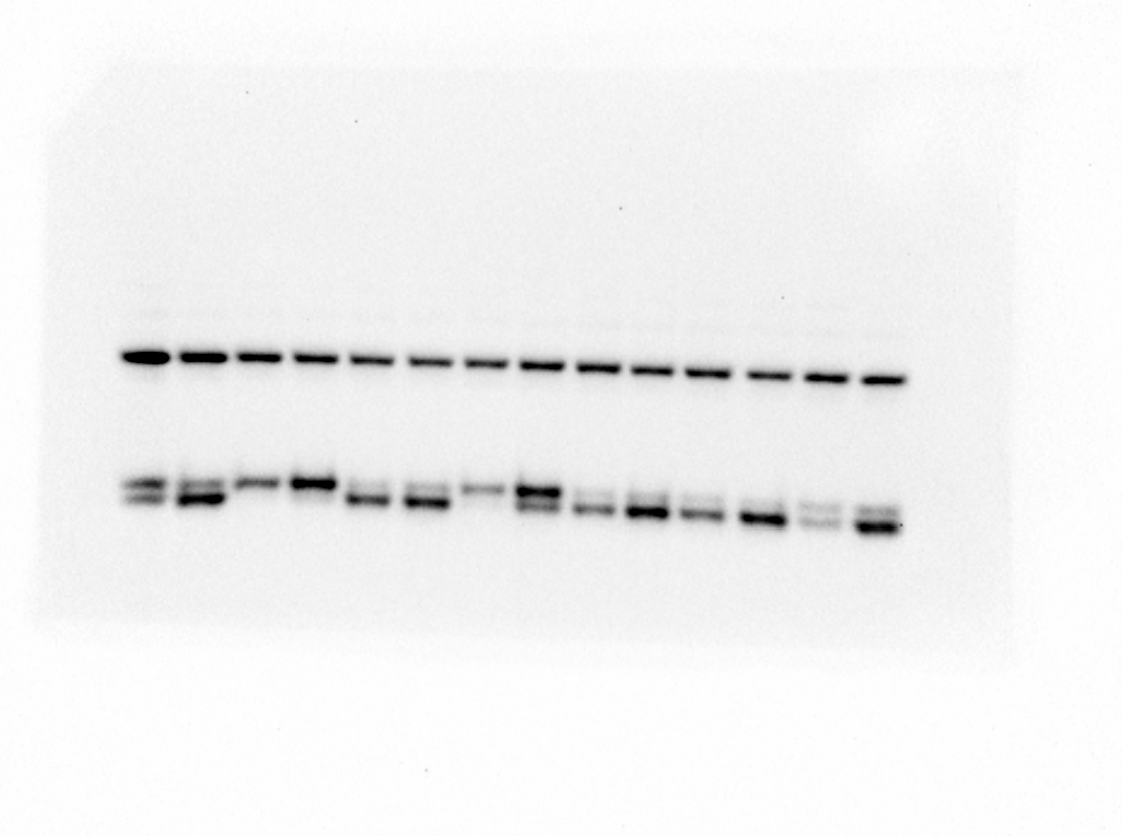

Supplement: Figure 3—source data 2. [file elife-74275-fig3-data2.zip › Figure 3-source data 2/Fig3A Set2 anti-sigK.tif]

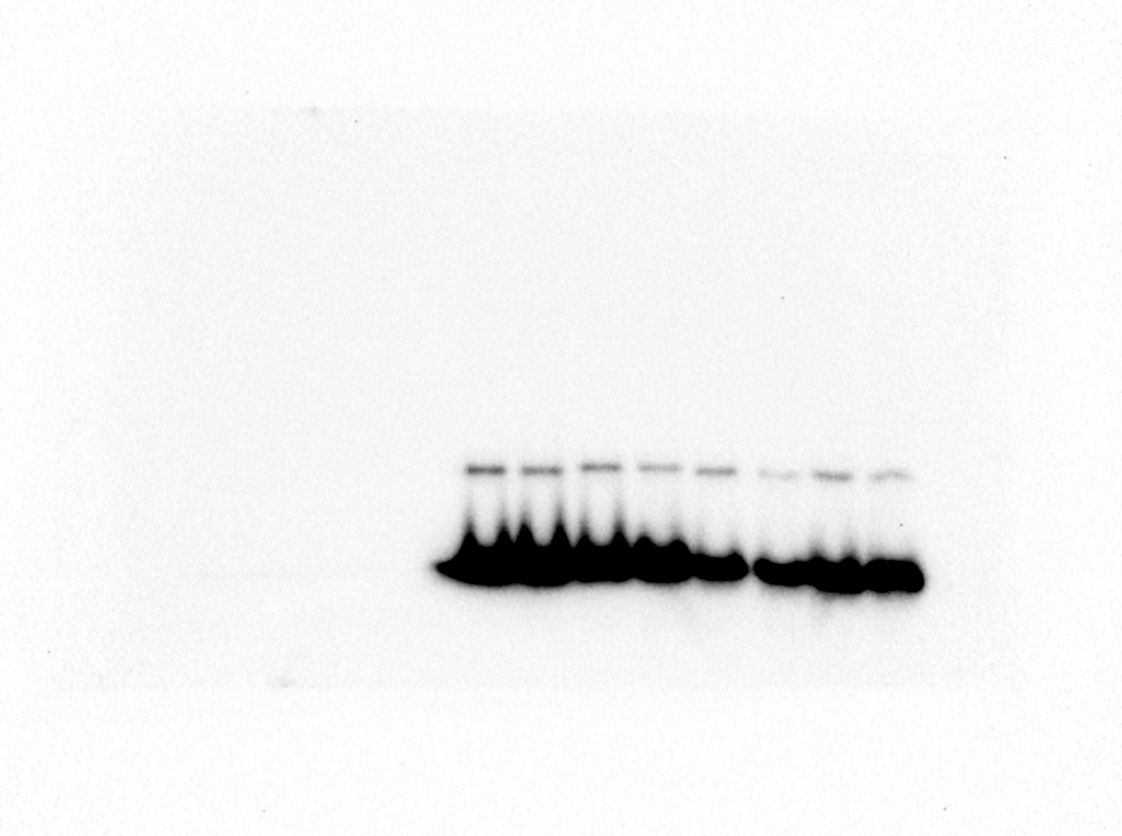

Supplement: Figure 3—source data 2. [file elife-74275-fig3-data2.zip › Figure 3-source data 2/Fig3A Set3 anti-GFP.tif]

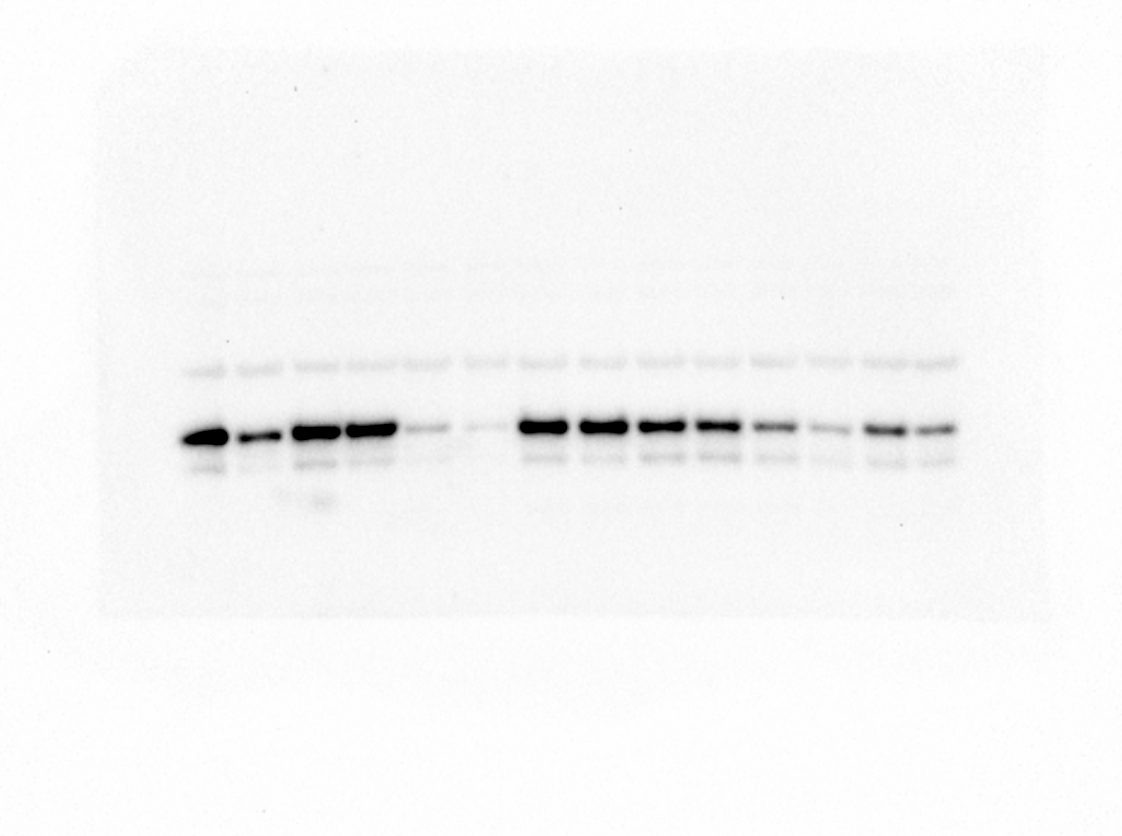

Supplement: Figure 3—source data 2. [file elife-74275-fig3-data2.zip › Figure 3-source data 2/Fig3A Set3 anti-IVFA.tif]

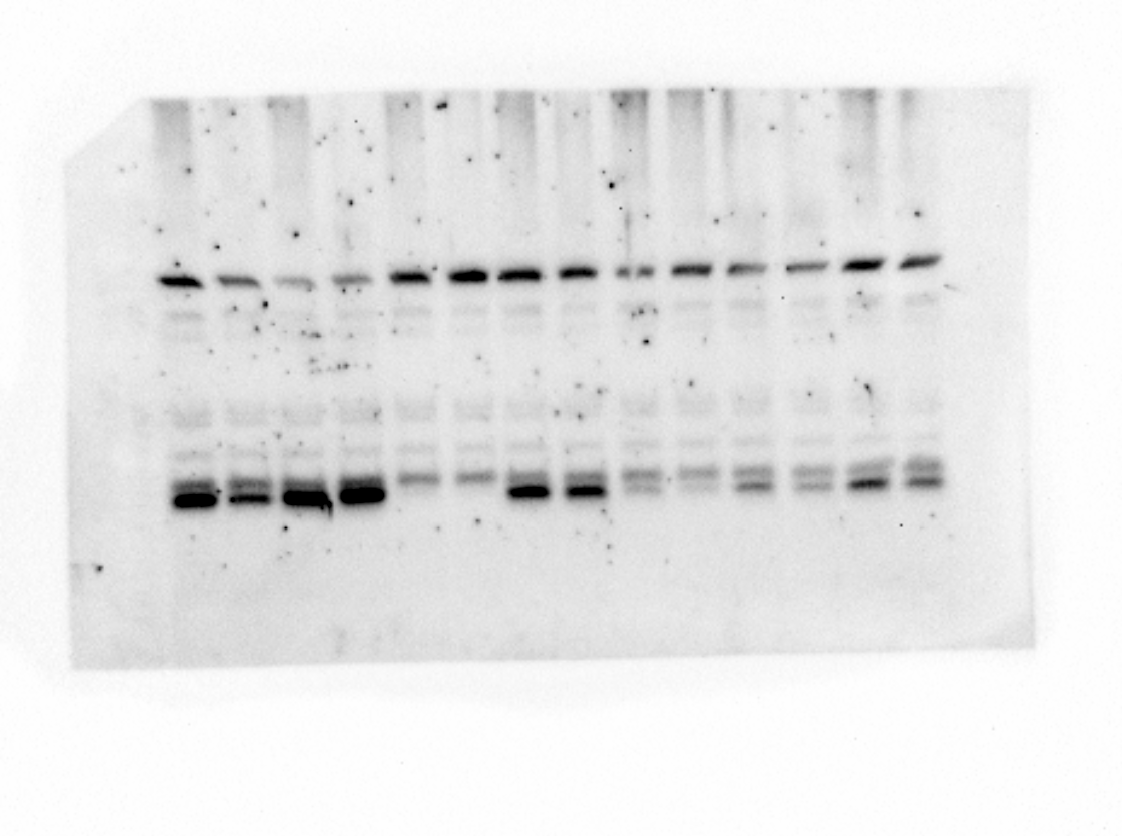

Supplement: Figure 3—source data 2. [file elife-74275-fig3-data2.zip › Figure 3-source data 2/Fig3A Set3 anti-IVFB.tif]

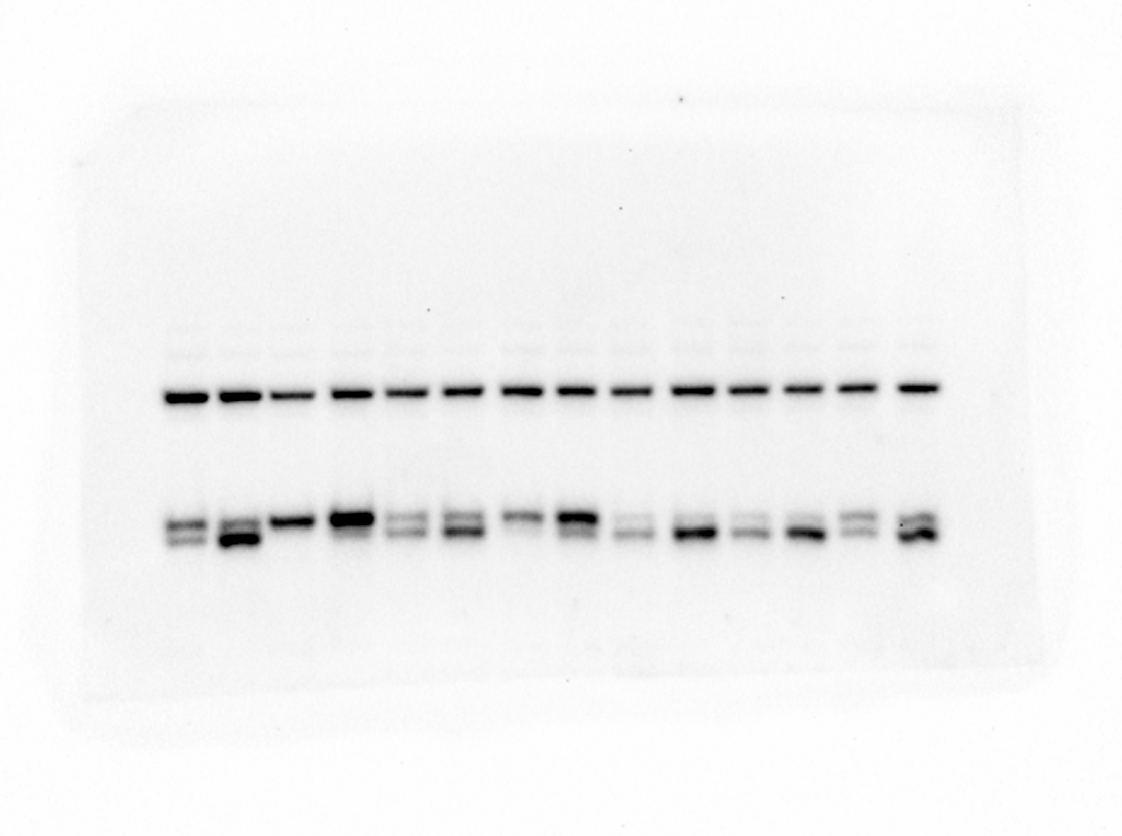

Supplement: Figure 3—source data 2. [file elife-74275-fig3-data2.zip › Figure 3-source data 2/Fig3A Set3 anti-sigK.tif]

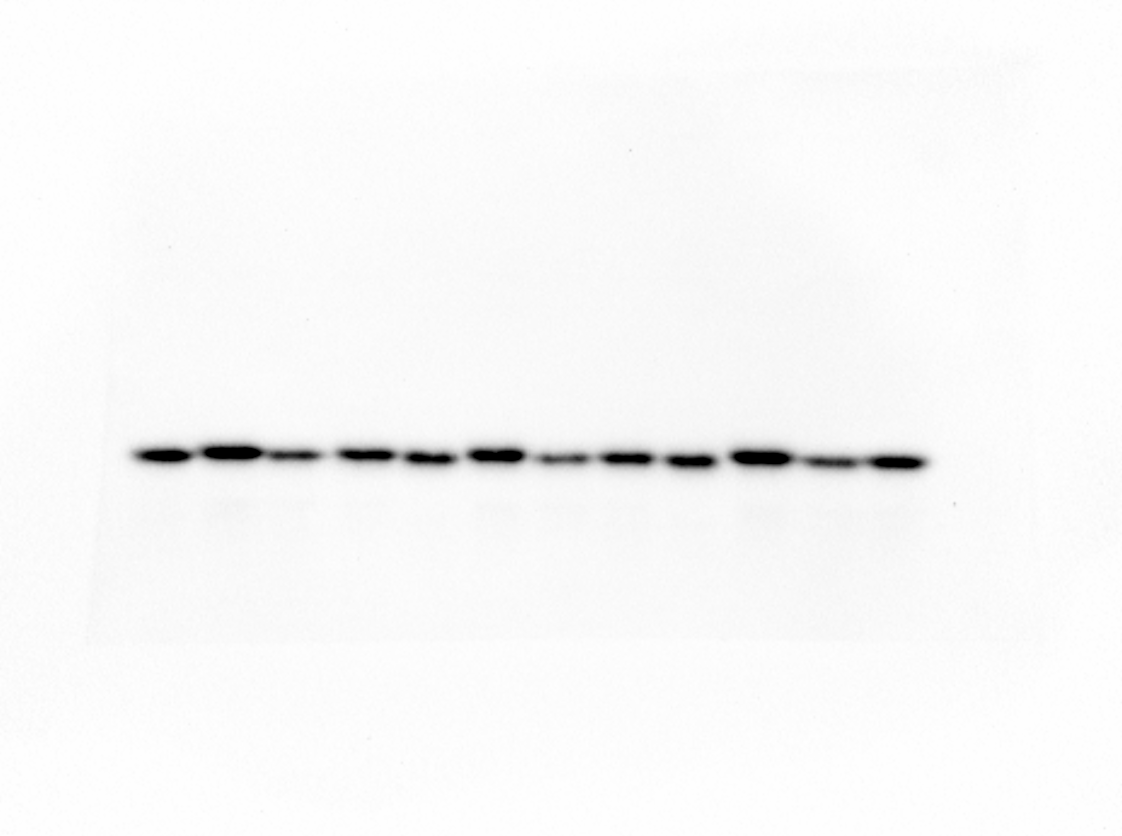

Supplement: Figure 3—figure supplement 1—source data 1. [file elife-74275-fig3-figsupp1-data1.zip › Figure 3-figure supplement 1-source data 1/fig sup 1 1min exposure anti-GFP.tif]

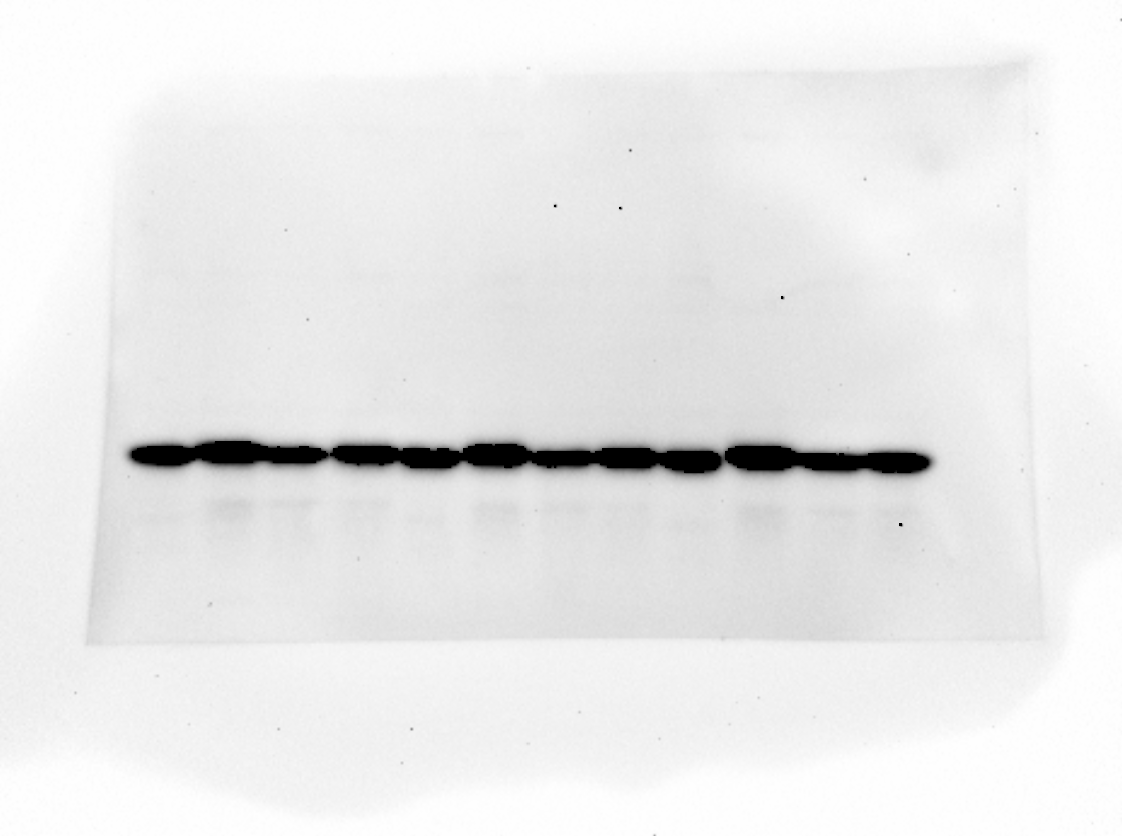

Supplement: Figure 3—figure supplement 1—source data 1. [file elife-74275-fig3-figsupp1-data1.zip › Figure 3-figure supplement 1-source data 1/fig sup 1 5min exposure anti-GFP.tif]

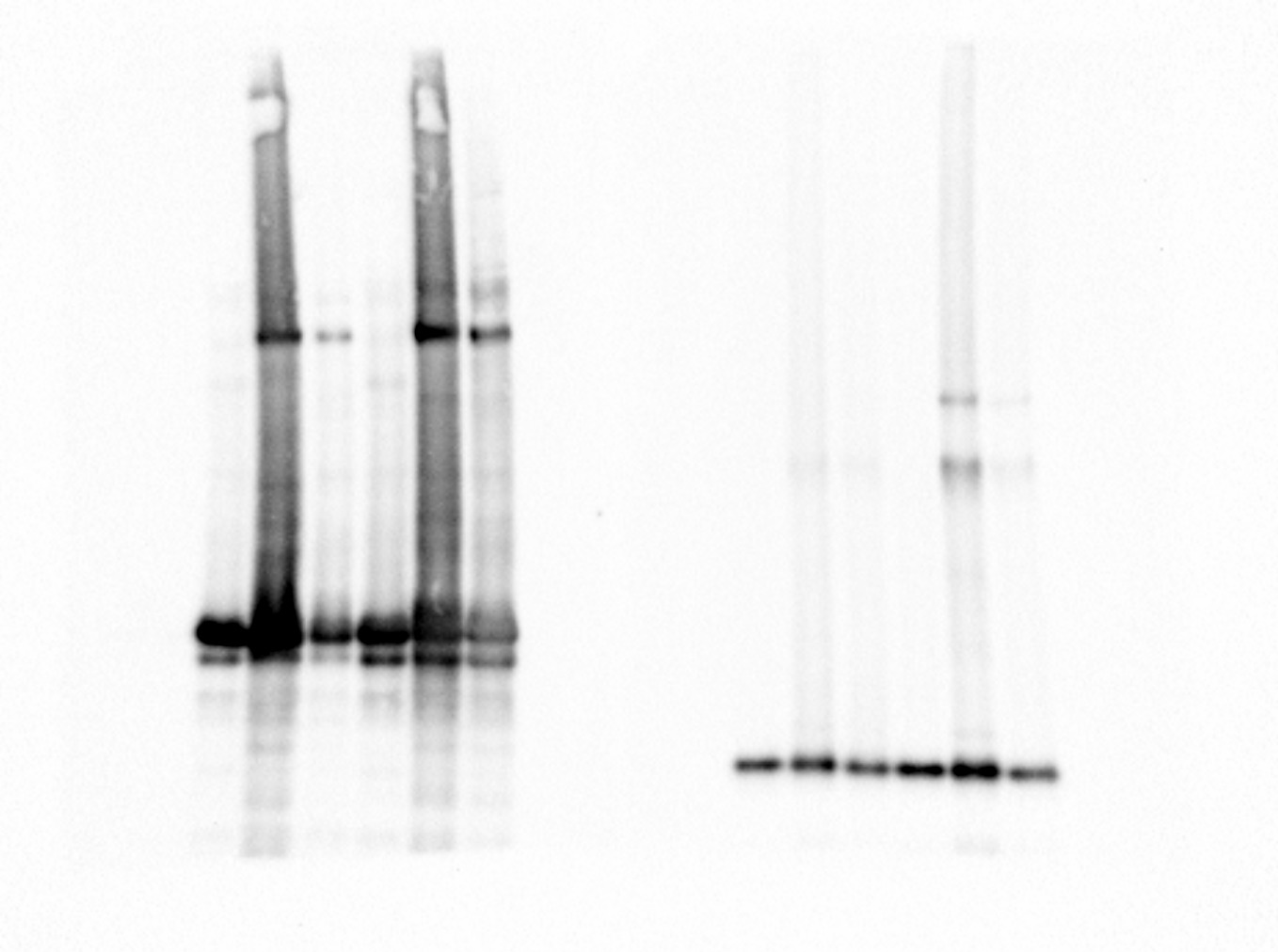

Supplement: Figure 4—source data 1. [file elife-74275-fig4-data1.zip › Figure 4-source data 1/Fig4A anti-FLAG.tif]

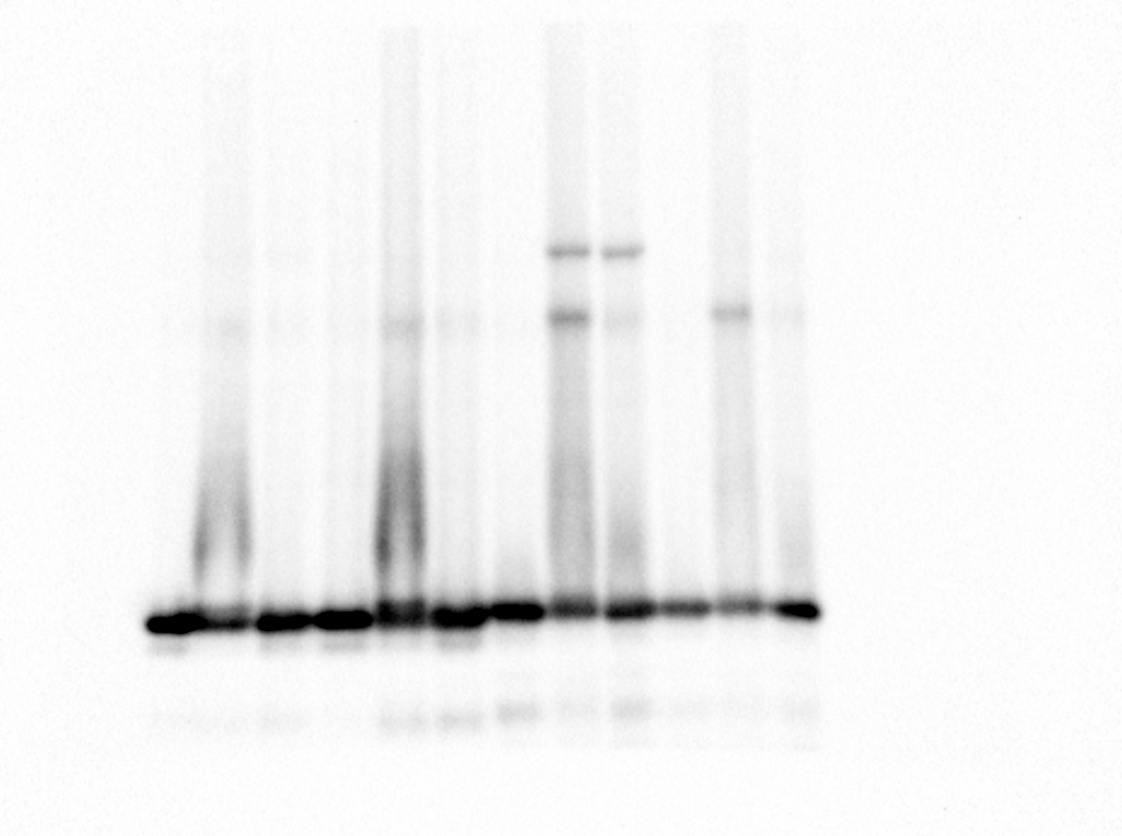

Supplement: Figure 4—source data 1. [file elife-74275-fig4-data1.zip › Figure 4-source data 1/Fig4B anti-FLAG.tif]

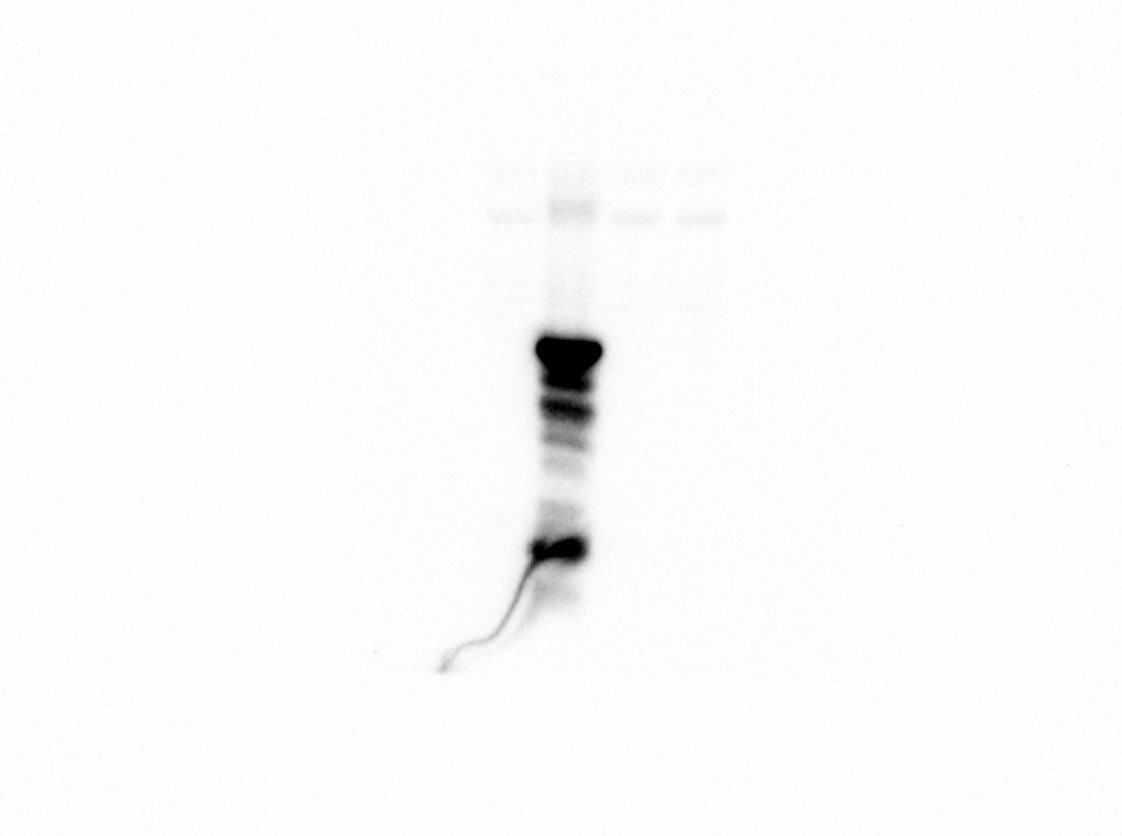

Supplement: Figure 4—figure supplement 2—source data 1. [file elife-74275-fig4-figsupp2-data1.zip › Figure 4-figure supplement 2-source data 1/fig sup 2 Set1 anti-GFP.tif]

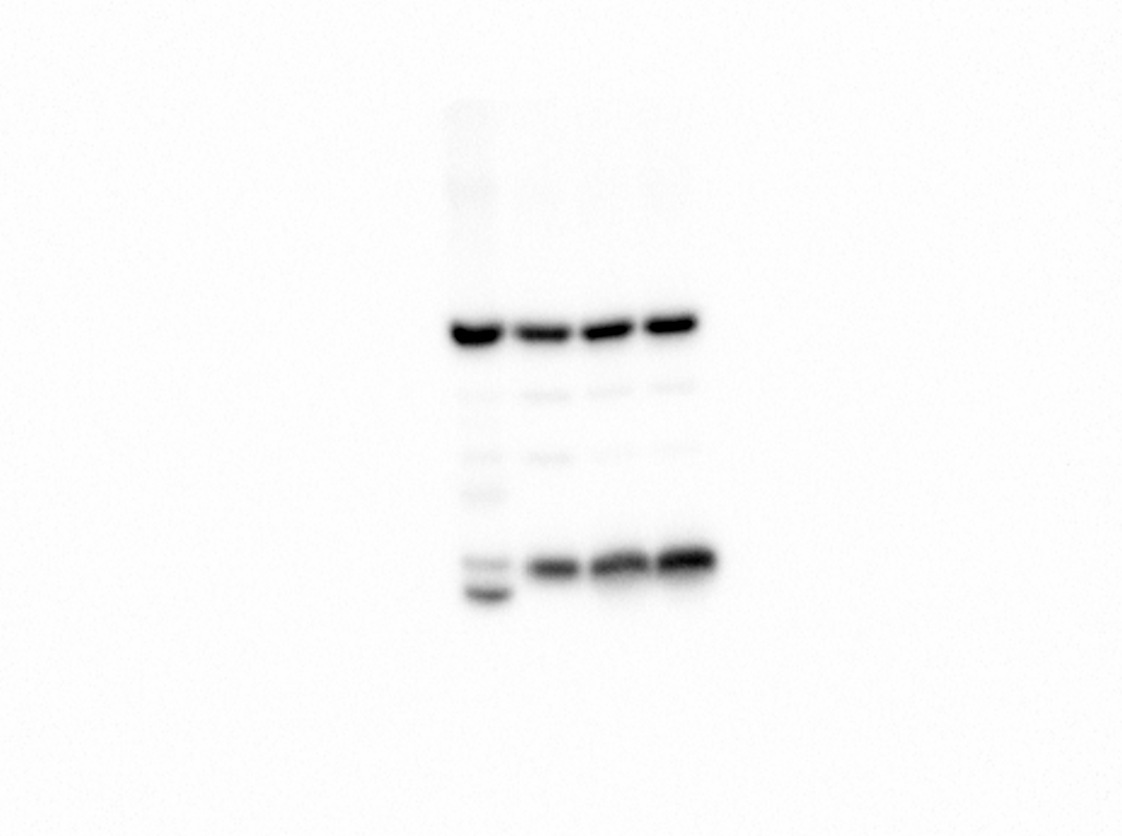

Supplement: Figure 4—figure supplement 2—source data 1. [file elife-74275-fig4-figsupp2-data1.zip › Figure 4-figure supplement 2-source data 1/fig sup 2 Set1 anti-His.tif]

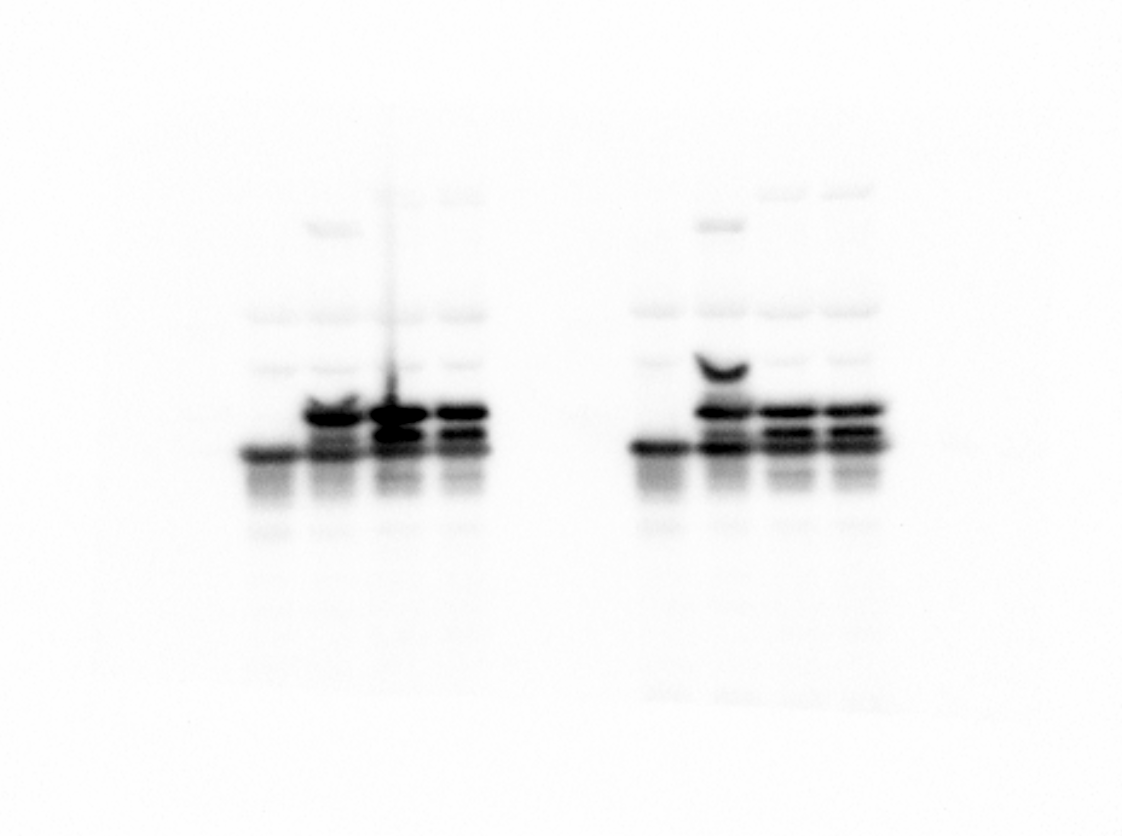

Supplement: Figure 4—figure supplement 2—source data 1. [file elife-74275-fig4-figsupp2-data1.zip › Figure 4-figure supplement 2-source data 1/fig sup 2 Set1 anti-IVFA.tif]

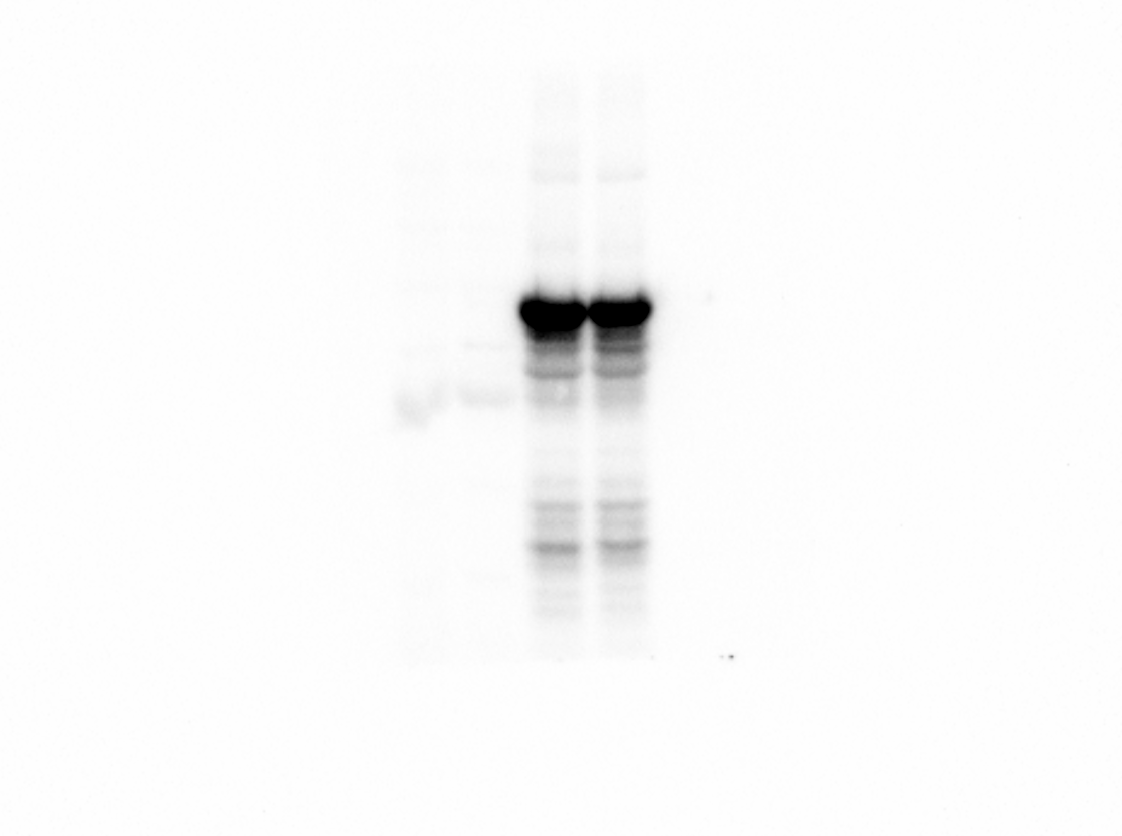

Supplement: Figure 4—figure supplement 2—source data 1. [file elife-74275-fig4-figsupp2-data1.zip › Figure 4-figure supplement 2-source data 1/fig sup 2 Set1 anti-MBP.tif]

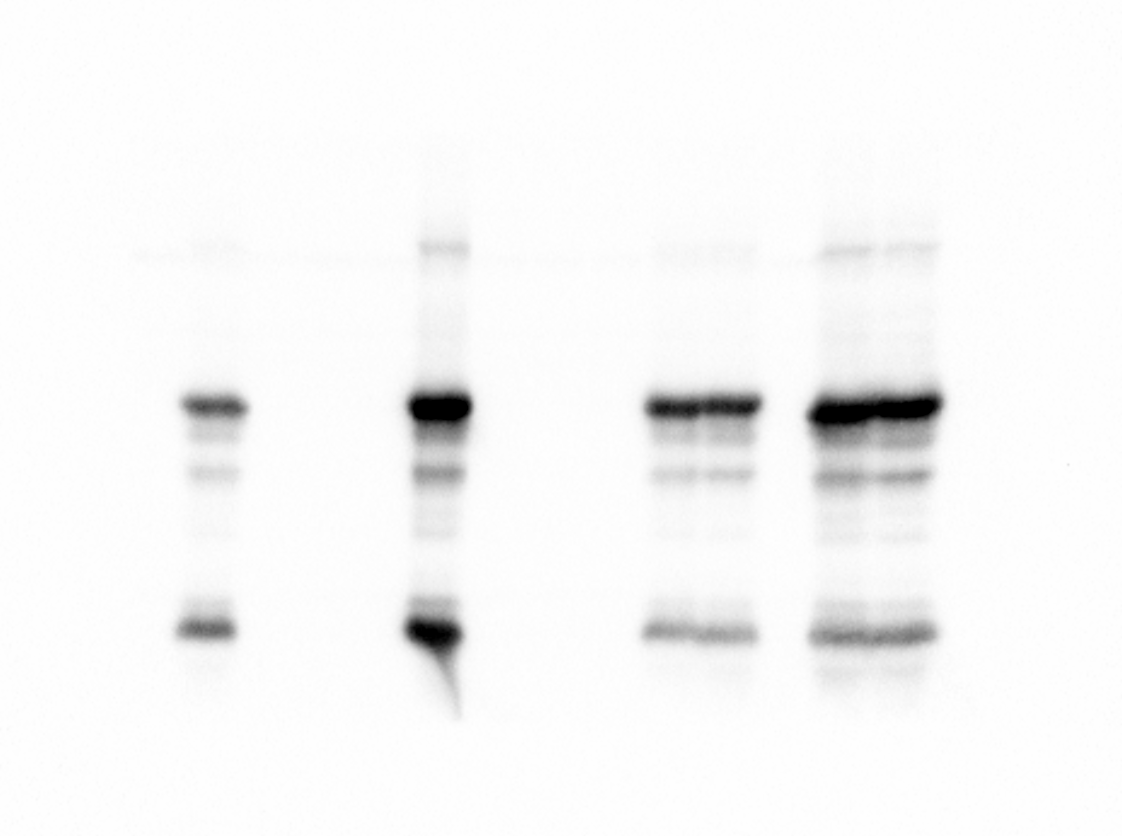

Supplement: Figure 4—figure supplement 2—source data 1. [file elife-74275-fig4-figsupp2-data1.zip › Figure 4-figure supplement 2-source data 1/fig sup 2 Set2 anti-GFP.tif]

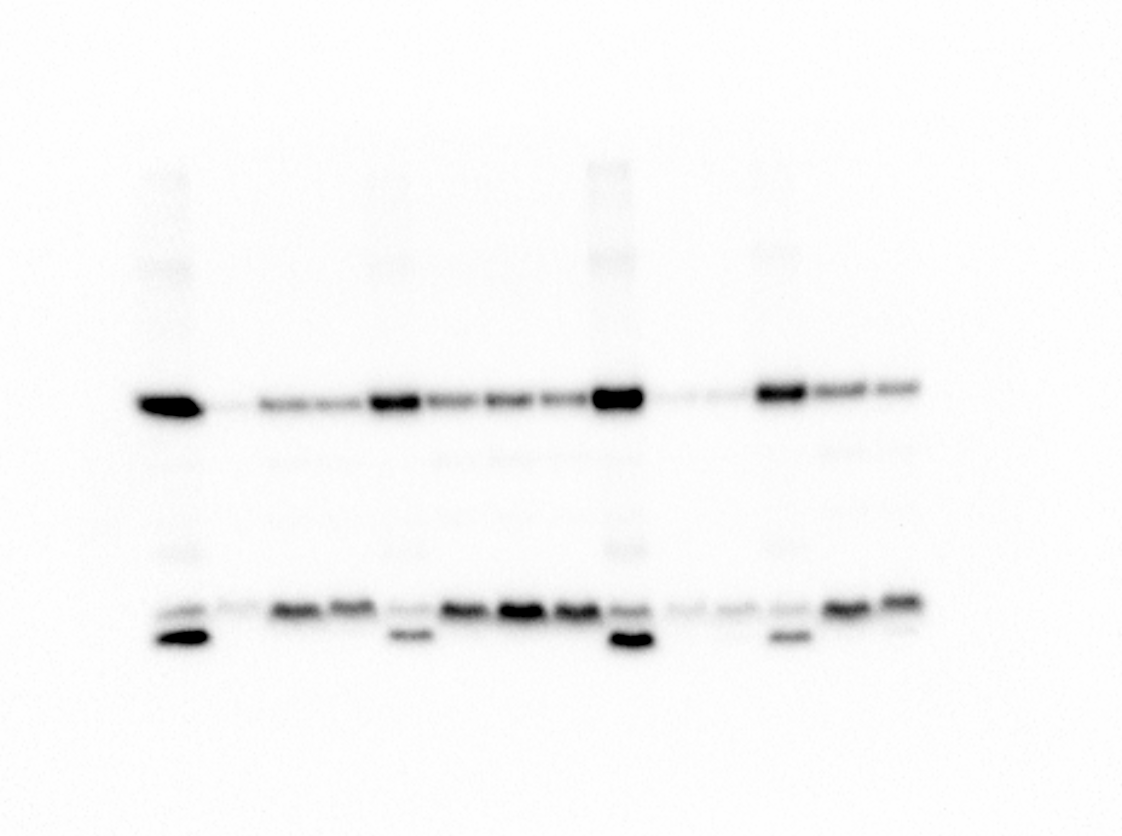

Supplement: Figure 4—figure supplement 2—source data 1. [file elife-74275-fig4-figsupp2-data1.zip › Figure 4-figure supplement 2-source data 1/fig sup 2 Set2 anti-His.tif]

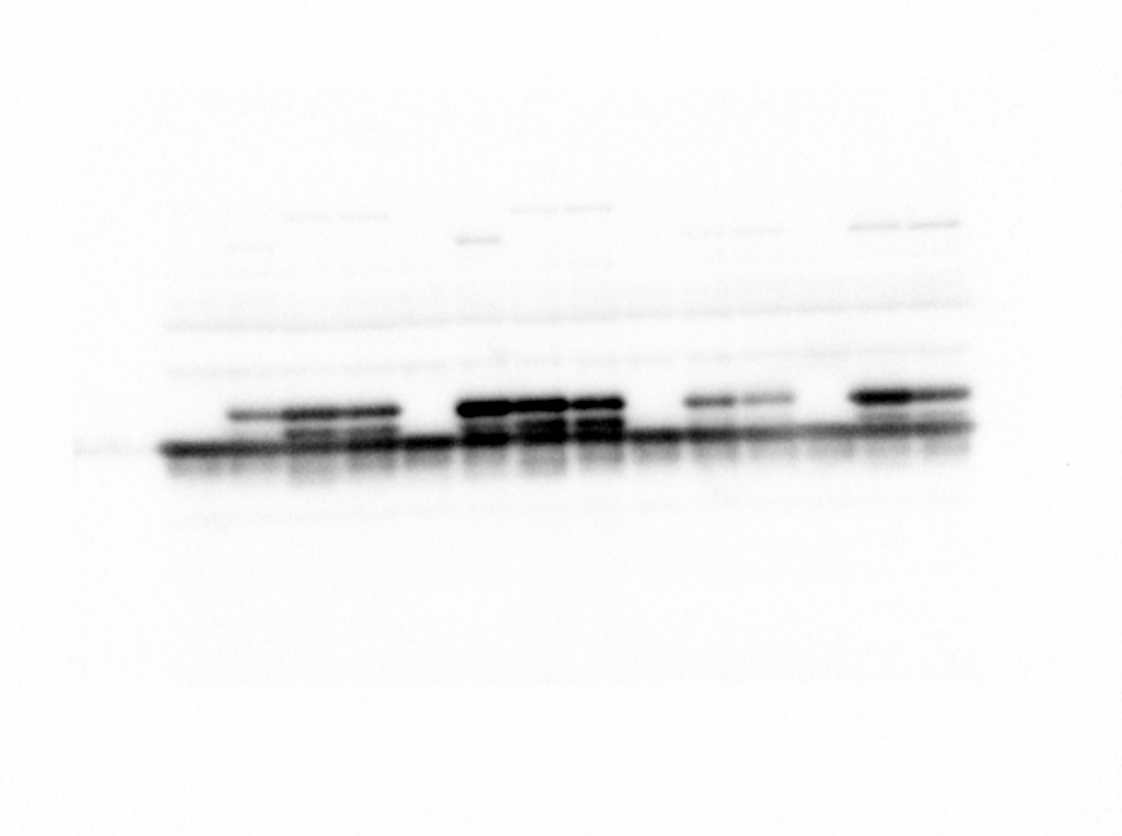

Supplement: Figure 4—figure supplement 2—source data 1. [file elife-74275-fig4-figsupp2-data1.zip › Figure 4-figure supplement 2-source data 1/fig sup 2 Set2 anti-IVFA.tif]

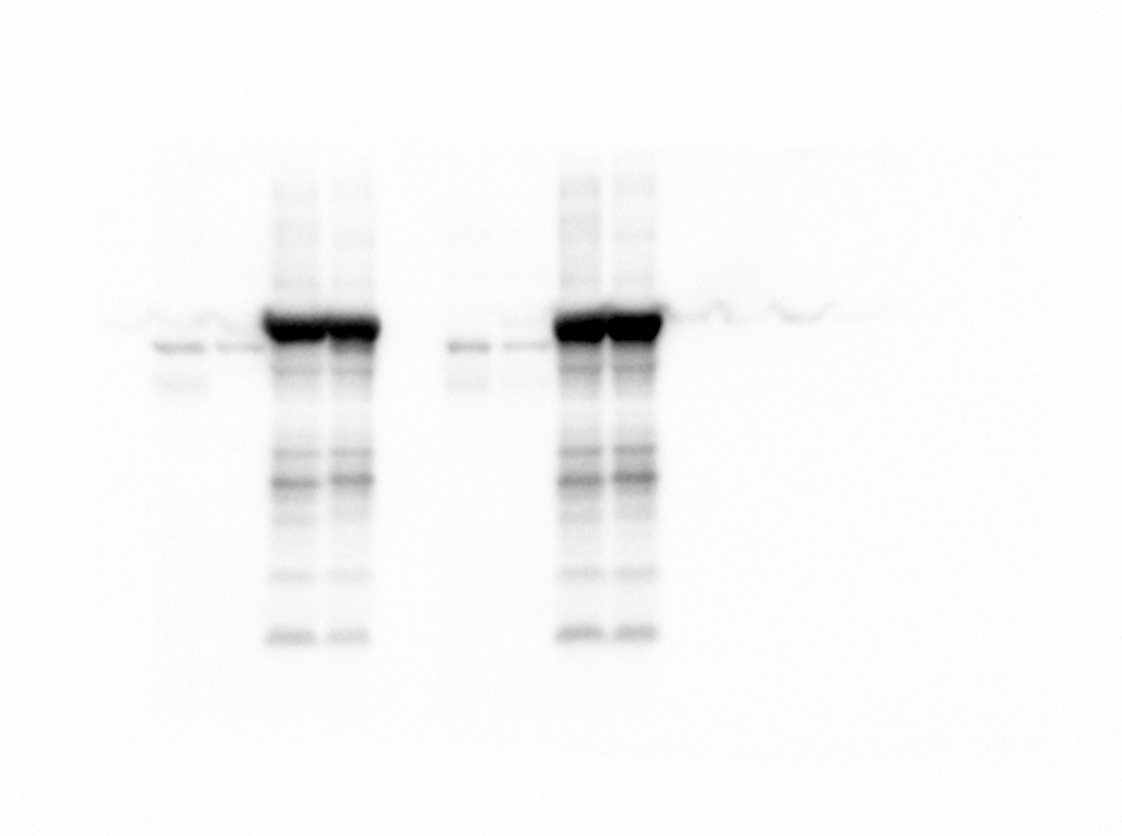

Supplement: Figure 4—figure supplement 2—source data 1. [file elife-74275-fig4-figsupp2-data1.zip › Figure 4-figure supplement 2-source data 1/fig sup 2 Set2 anti-MBP.tif]

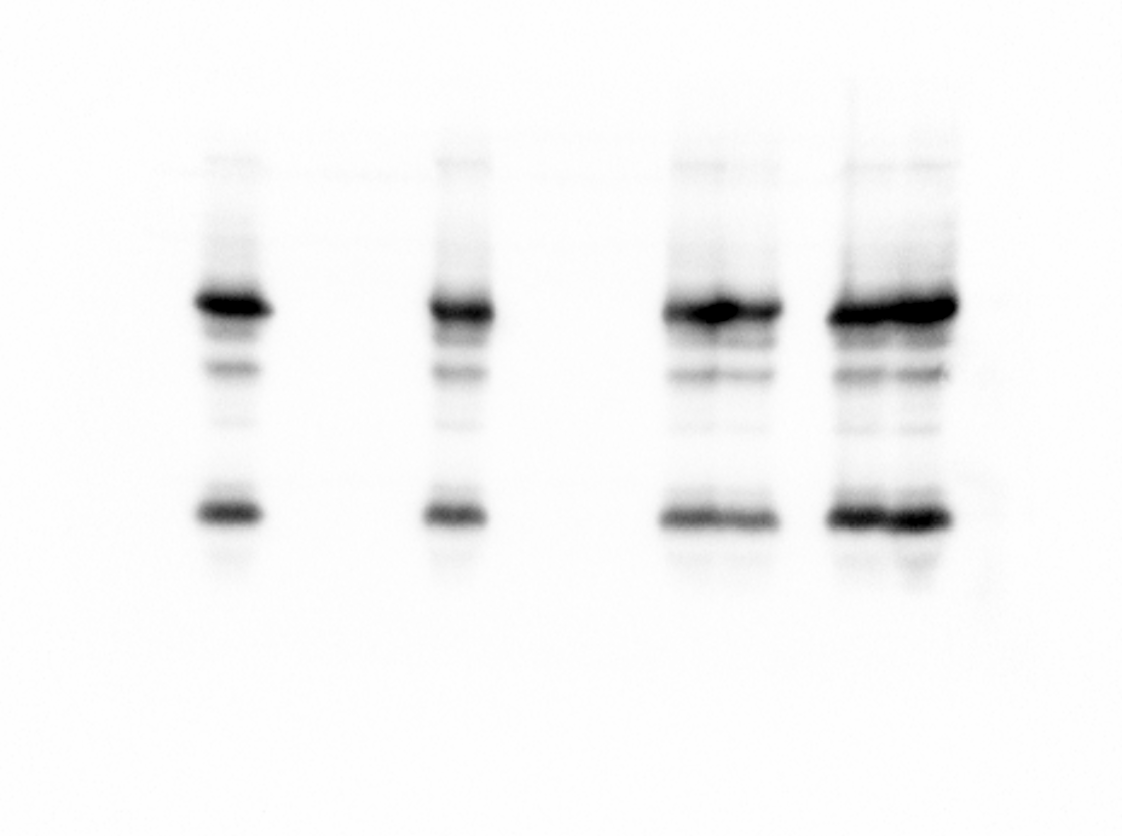

Supplement: Figure 4—figure supplement 2—source data 1. [file elife-74275-fig4-figsupp2-data1.zip › Figure 4-figure supplement 2-source data 1/fig sup 2 Set3 anti-GFP.tif]

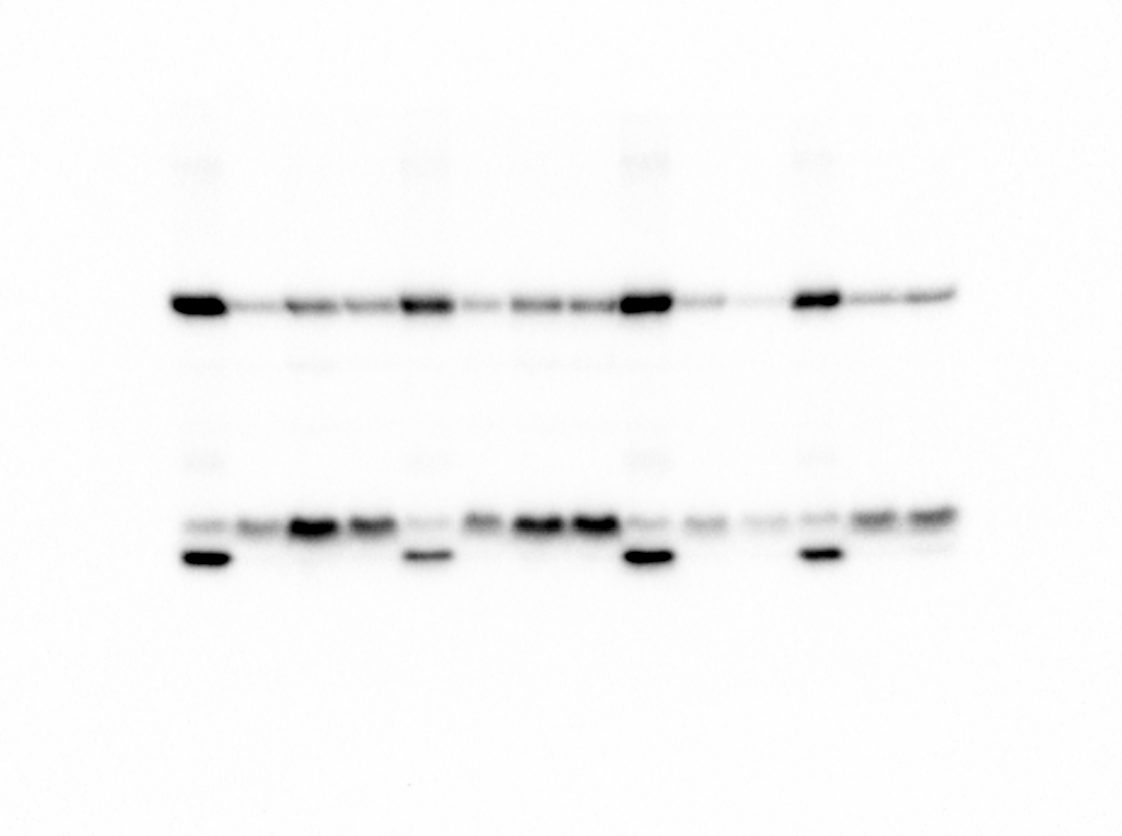

Supplement: Figure 4—figure supplement 2—source data 1. [file elife-74275-fig4-figsupp2-data1.zip › Figure 4-figure supplement 2-source data 1/fig sup 2 Set3 anti-His.tif]

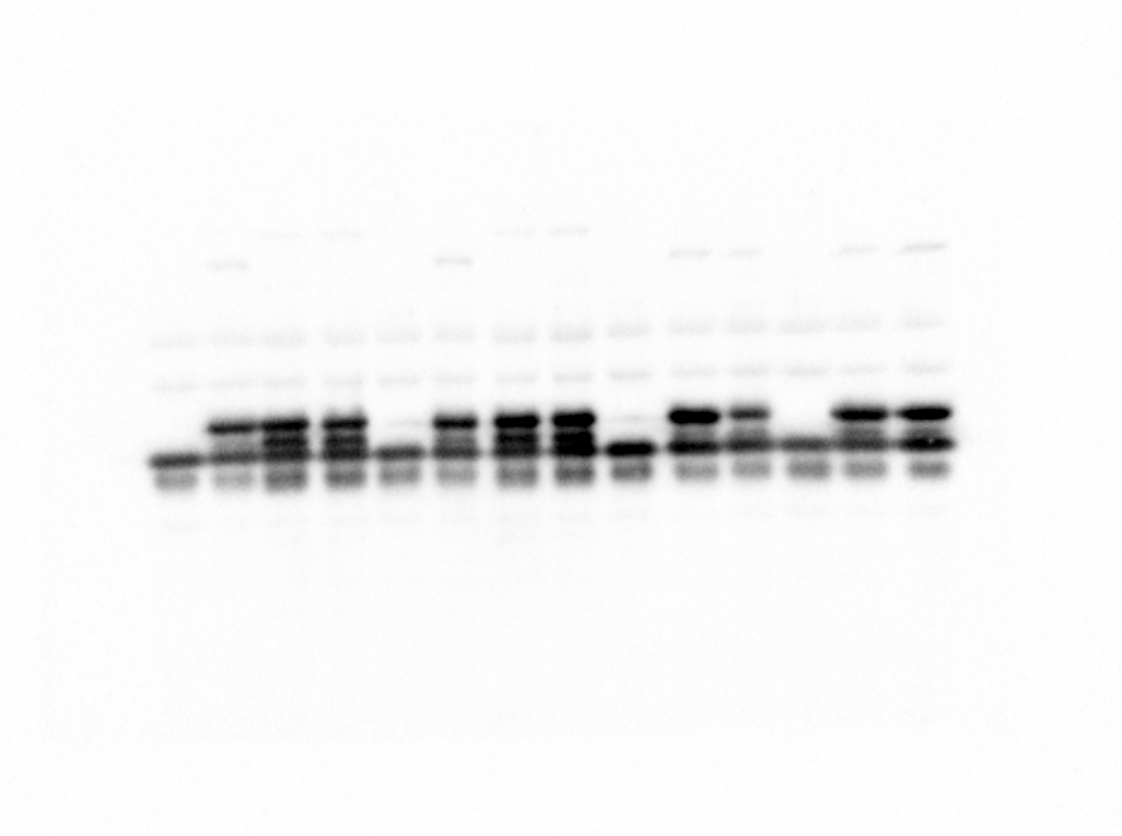

Supplement: Figure 4—figure supplement 2—source data 1. [file elife-74275-fig4-figsupp2-data1.zip › Figure 4-figure supplement 2-source data 1/fig sup 2 Set3 anti-IVFA.tif]

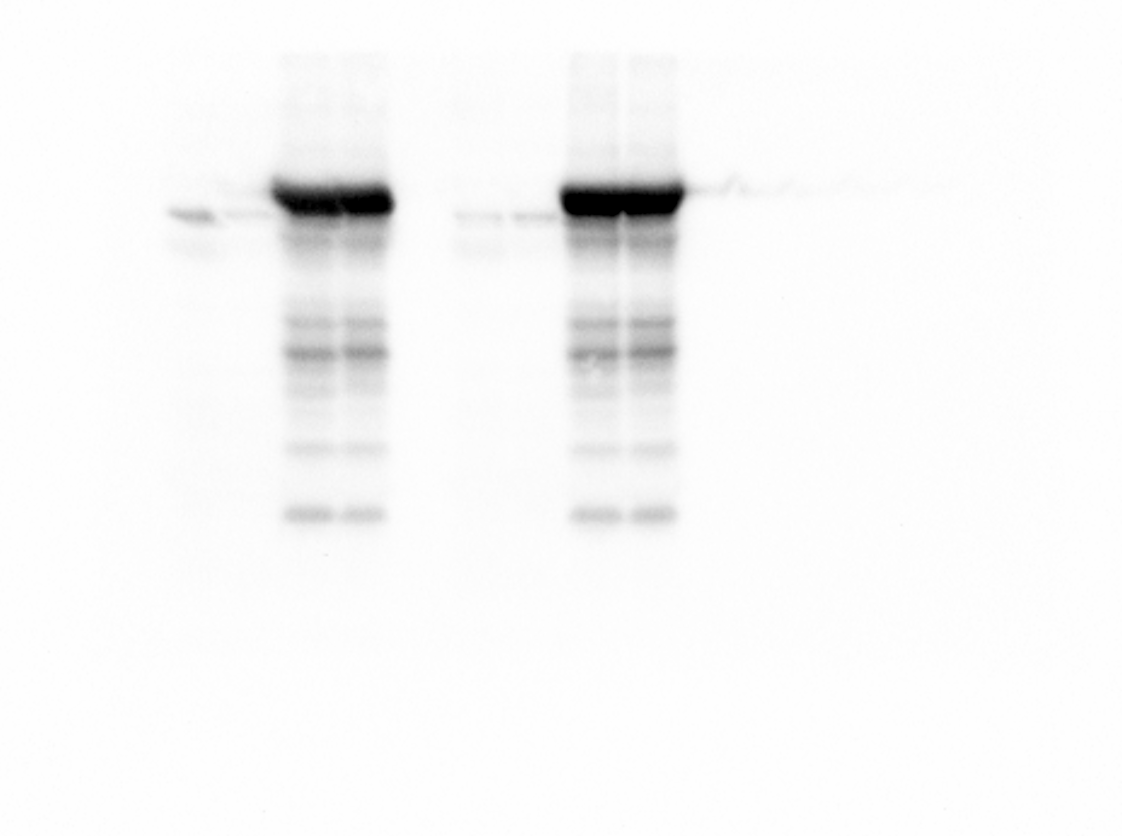

Supplement: Figure 4—figure supplement 2—source data 1. [file elife-74275-fig4-figsupp2-data1.zip › Figure 4-figure supplement 2-source data 1/fig sup 2 Set3 anti-MBP.tif]

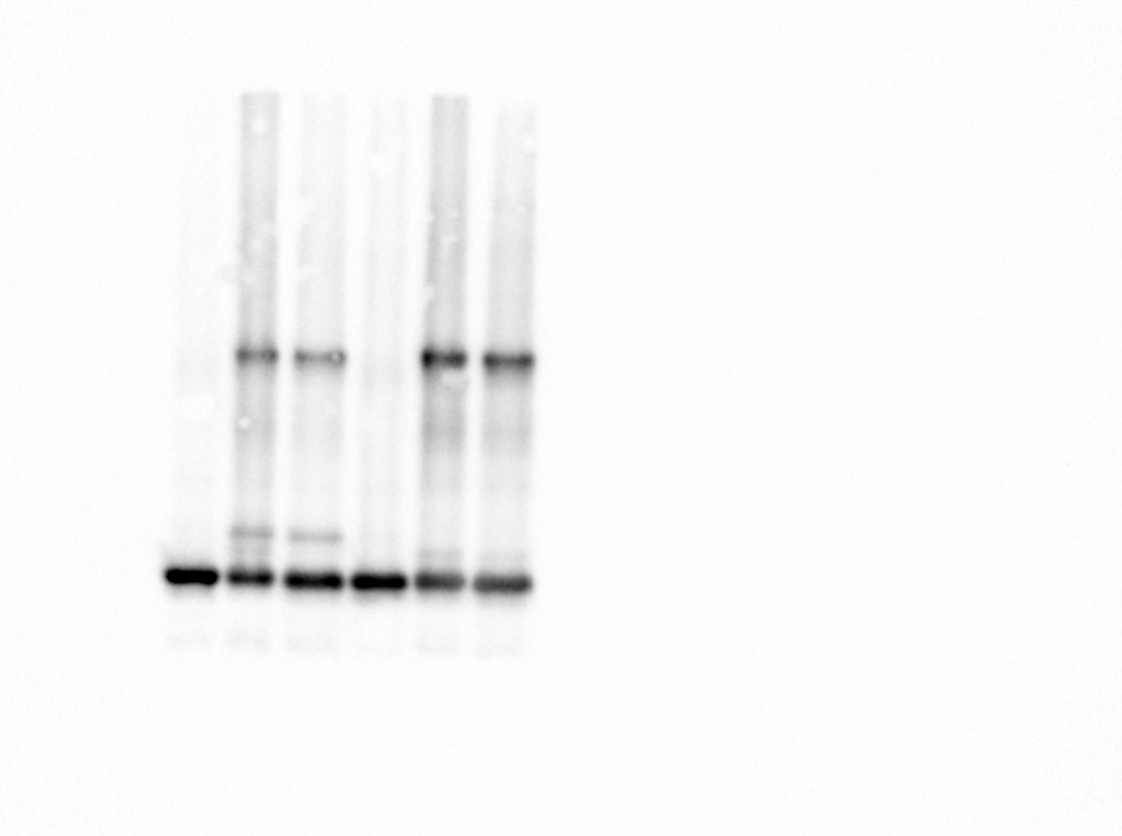

Supplement: Figure 4—figure supplement 4—source data 1. [file elife-74275-fig4-figsupp4-data1.zip › Figure 4-figure supplement 4-source data 1/fig sup 4 E44C anti-FLAG.tif]

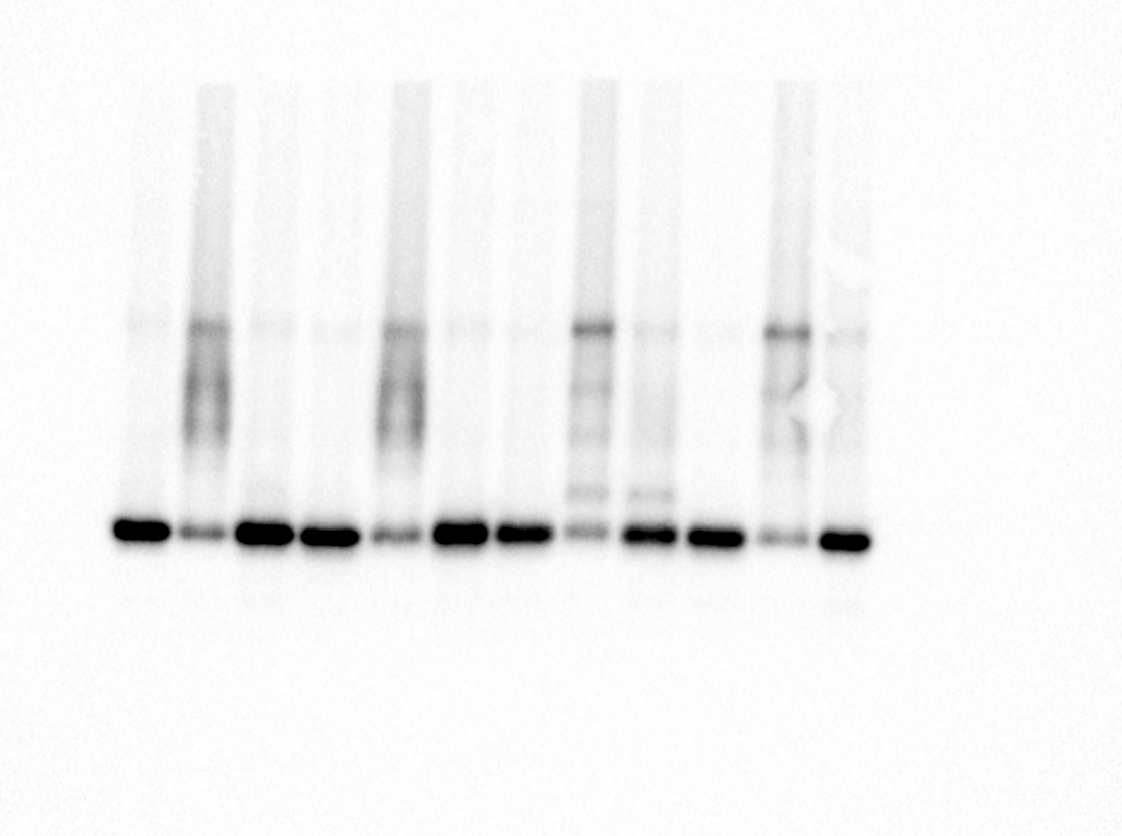

Supplement: Figure 4—figure supplement 4—source data 1. [file elife-74275-fig4-figsupp4-data1.zip › Figure 4-figure supplement 4-source data 1/fig sup 4 V70C and P135C anti-FLAG.tif]

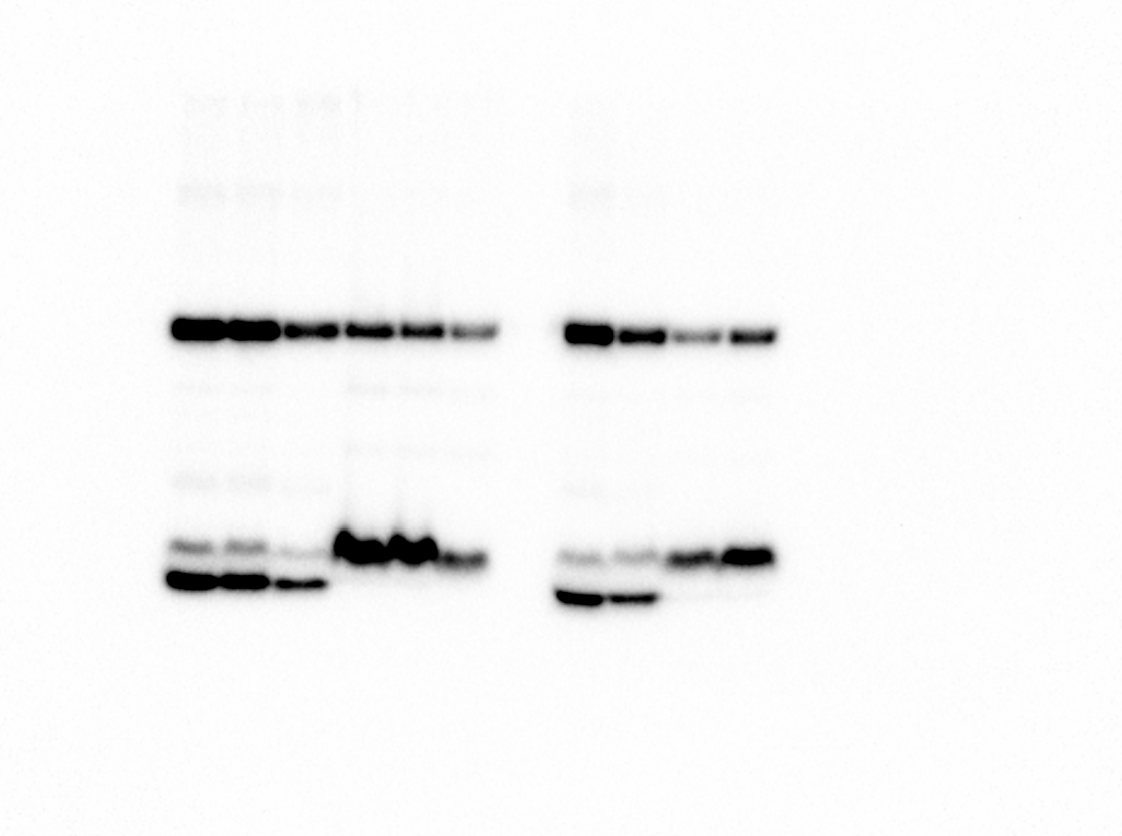

Supplement: Figure 4—figure supplement 5—source data 1. [file elife-74275-fig4-figsupp5-data1.zip › Figure 4-figure supplement 5-source data 1/figure supplement 5B/fig sup 5B Set 3 anti-His blot 3.tif]

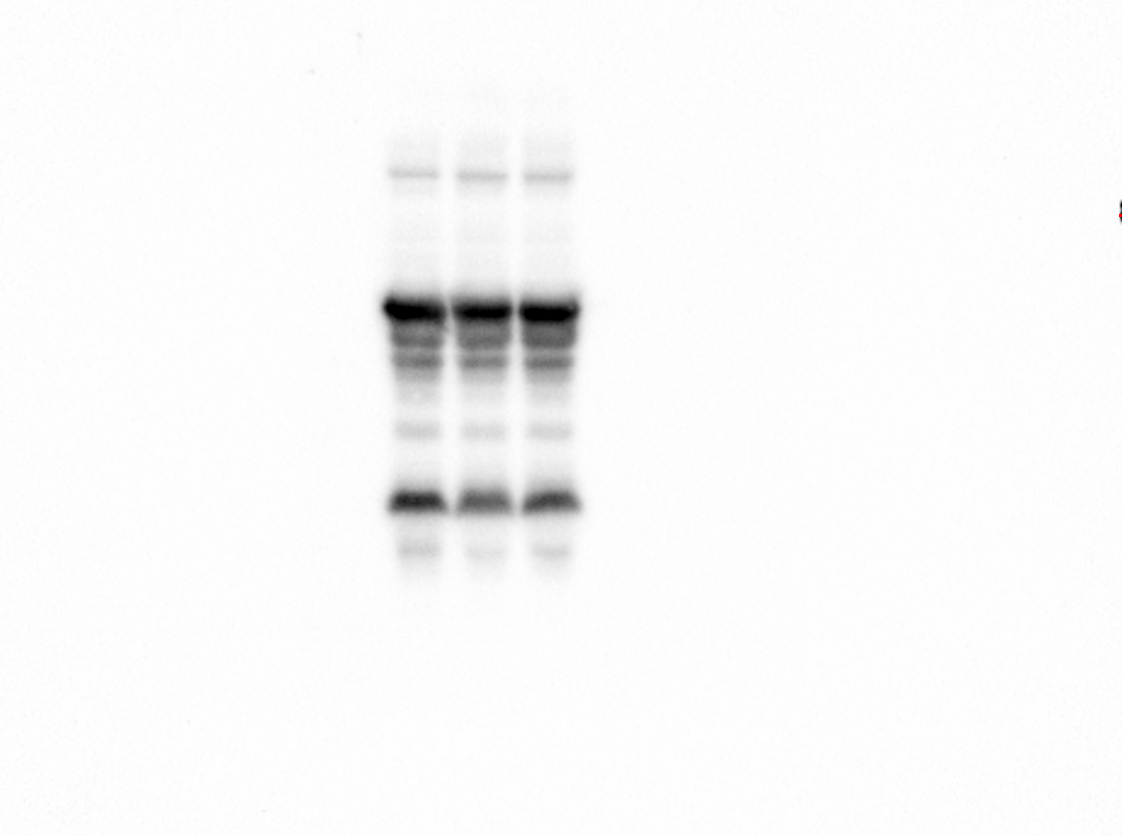

Supplement: Figure 4—figure supplement 5—source data 1. [file elife-74275-fig4-figsupp5-data1.zip › Figure 4-figure supplement 5-source data 1/figure supplement 5B/fig sup 5B Set1 anti-GFP blot 1.tif]

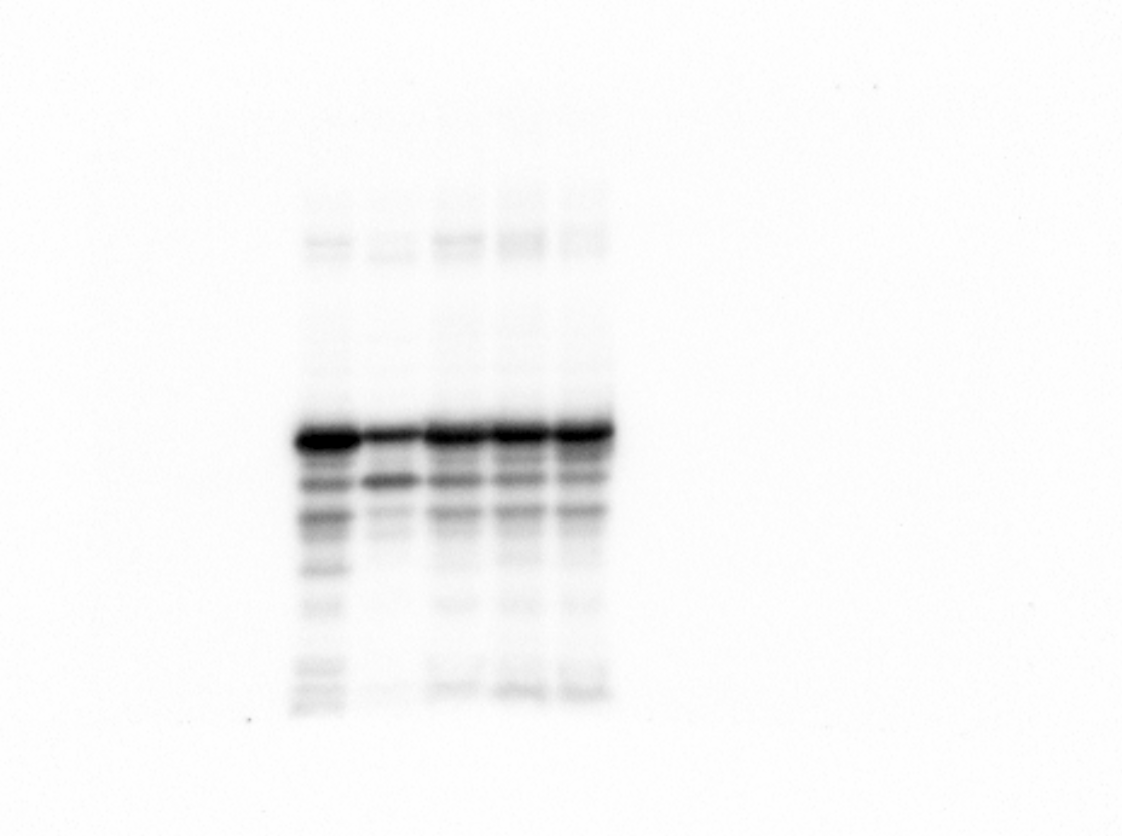

Supplement: Figure 4—figure supplement 5—source data 1. [file elife-74275-fig4-figsupp5-data1.zip › Figure 4-figure supplement 5-source data 1/figure supplement 5B/fig sup 5B Set1 anti-GFP blot 2.tif]

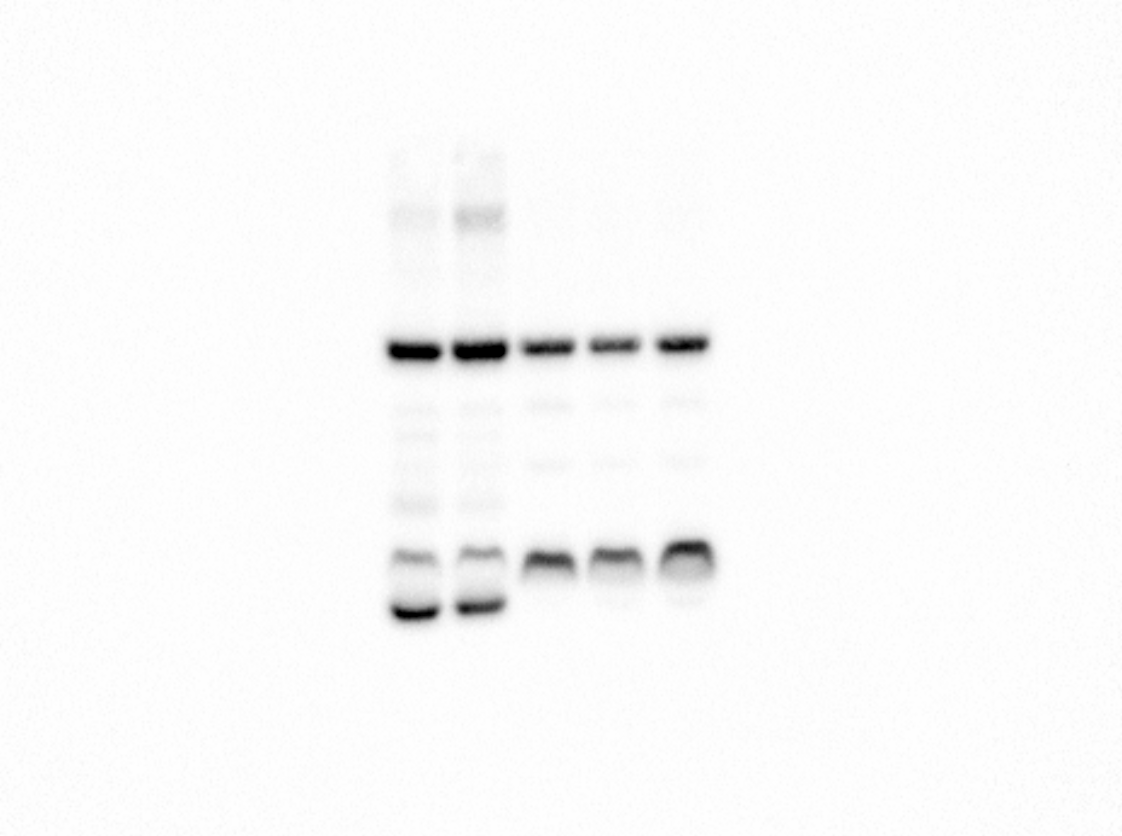

Supplement: Figure 4—figure supplement 5—source data 1. [file elife-74275-fig4-figsupp5-data1.zip › Figure 4-figure supplement 5-source data 1/figure supplement 5B/fig sup 5B Set1 anti-His blot 1.tif]

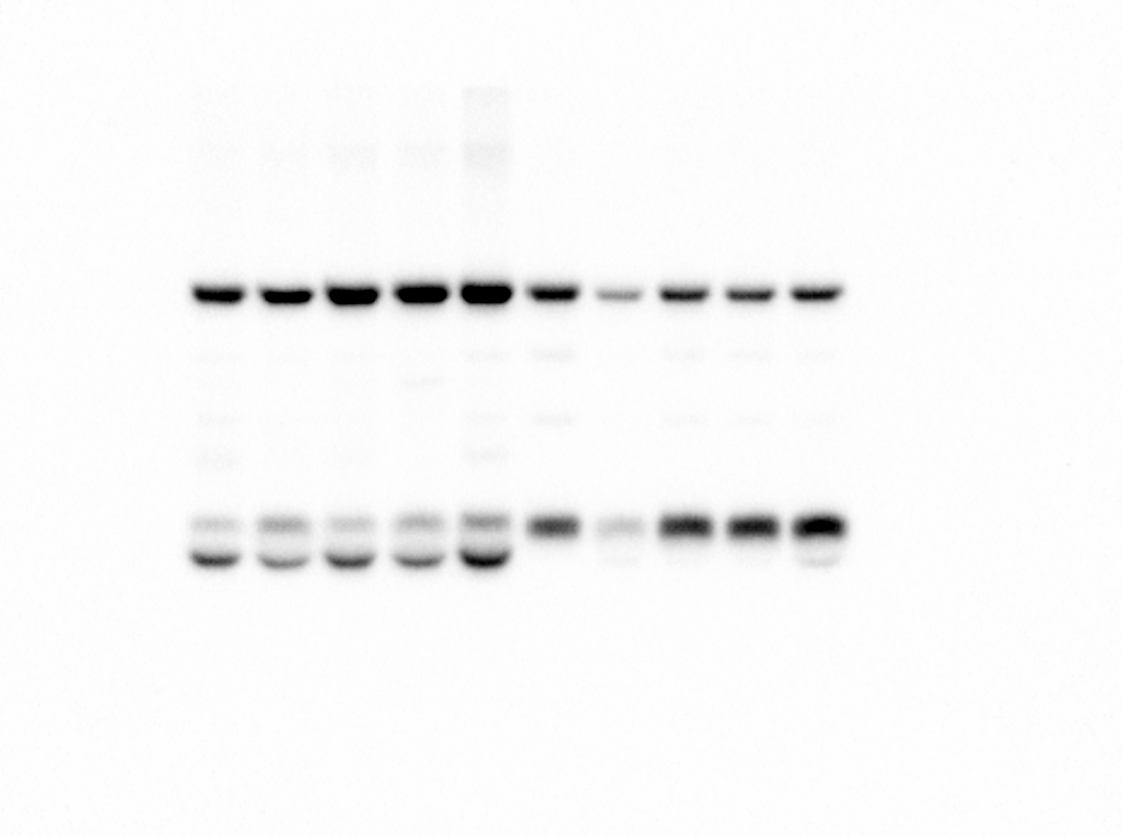

Supplement: Figure 4—figure supplement 5—source data 1. [file elife-74275-fig4-figsupp5-data1.zip › Figure 4-figure supplement 5-source data 1/figure supplement 5B/fig sup 5B Set1 anti-His blot 2.tif]

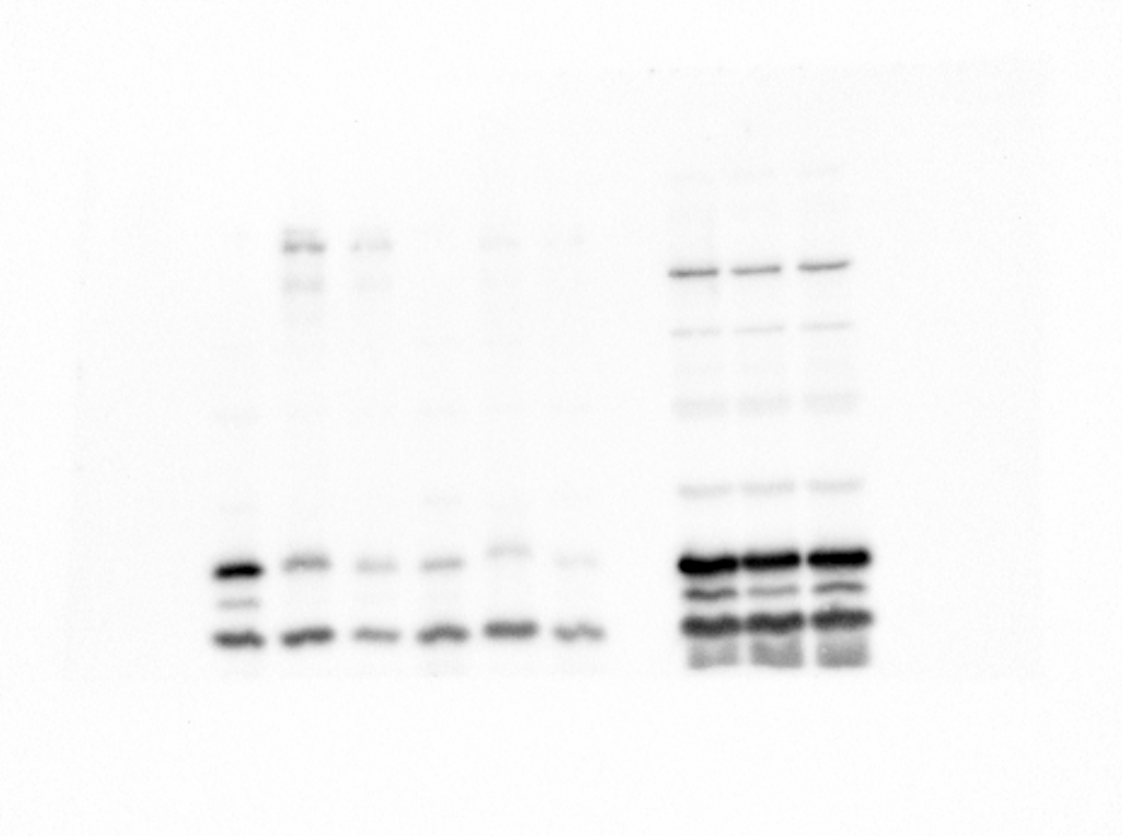

Supplement: Figure 4—figure supplement 5—source data 1. [file elife-74275-fig4-figsupp5-data1.zip › Figure 4-figure supplement 5-source data 1/figure supplement 5B/fig sup 5B Set1 anti-IVFA blot 1.tif]

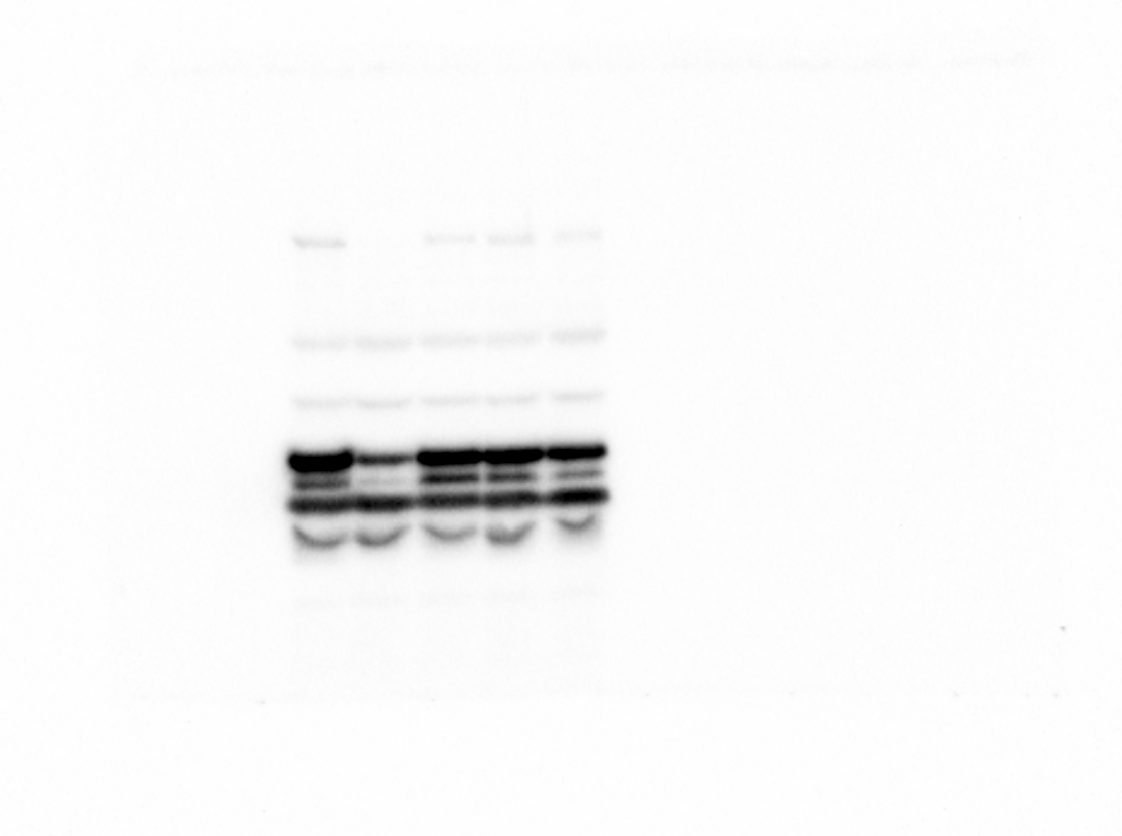

Supplement: Figure 4—figure supplement 5—source data 1. [file elife-74275-fig4-figsupp5-data1.zip › Figure 4-figure supplement 5-source data 1/figure supplement 5B/fig sup 5B Set1 anti-IVFA blot 2.tif]

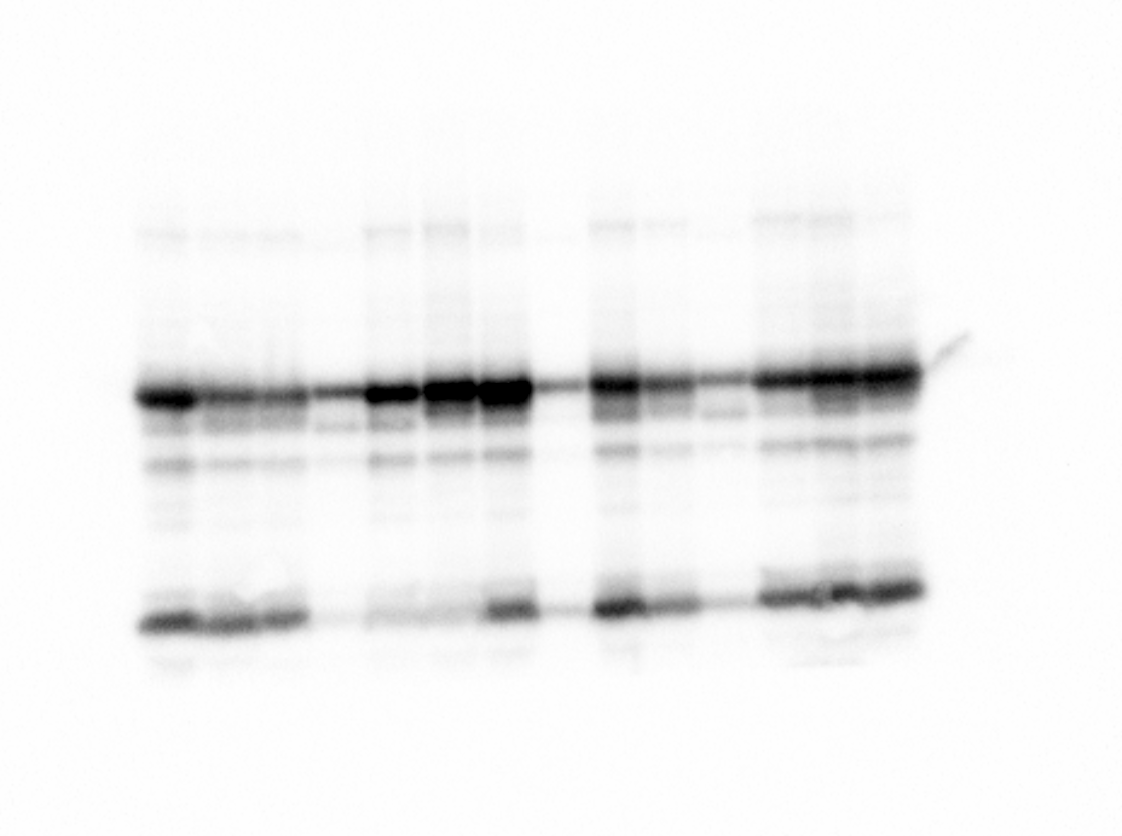

Supplement: Figure 4—figure supplement 5—source data 1. [file elife-74275-fig4-figsupp5-data1.zip › Figure 4-figure supplement 5-source data 1/figure supplement 5B/fig sup 5B Sets 2 and 3 anti-GFP.tif]

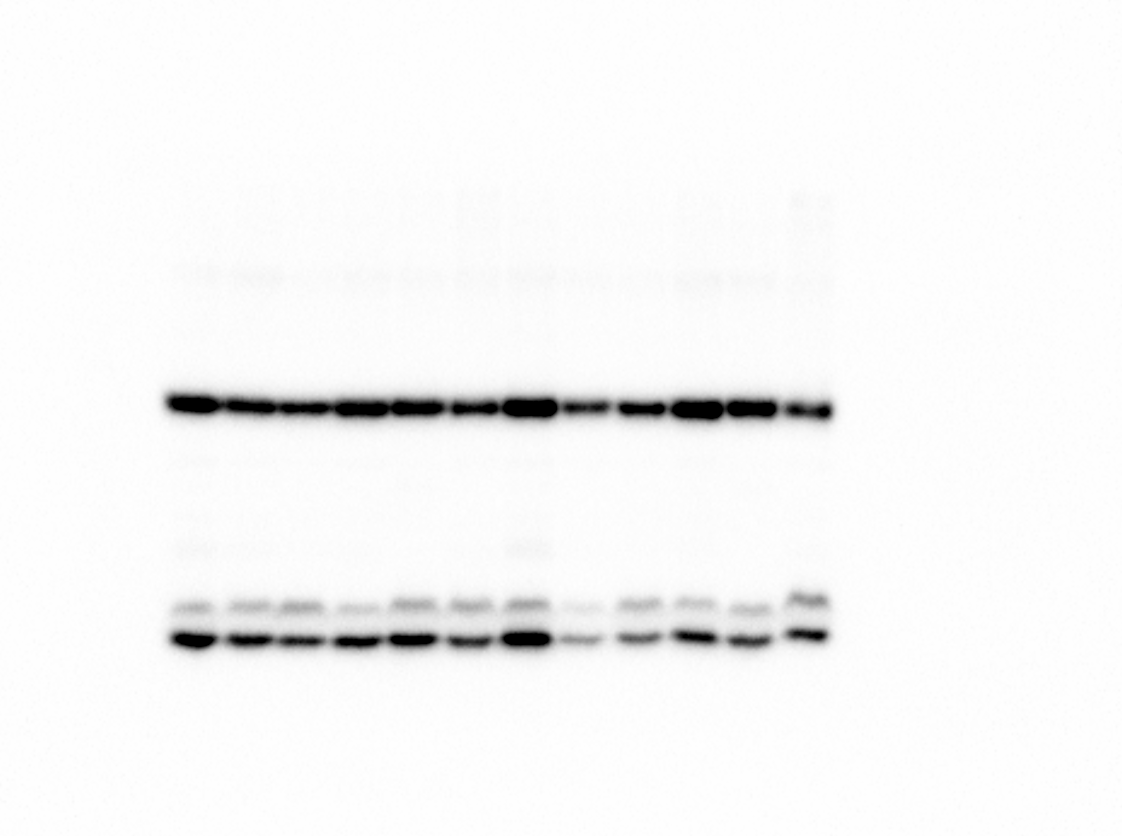

Supplement: Figure 4—figure supplement 5—source data 1. [file elife-74275-fig4-figsupp5-data1.zip › Figure 4-figure supplement 5-source data 1/figure supplement 5B/fig sup 5B Sets 2 and 3 anti-His blot 1.tif]

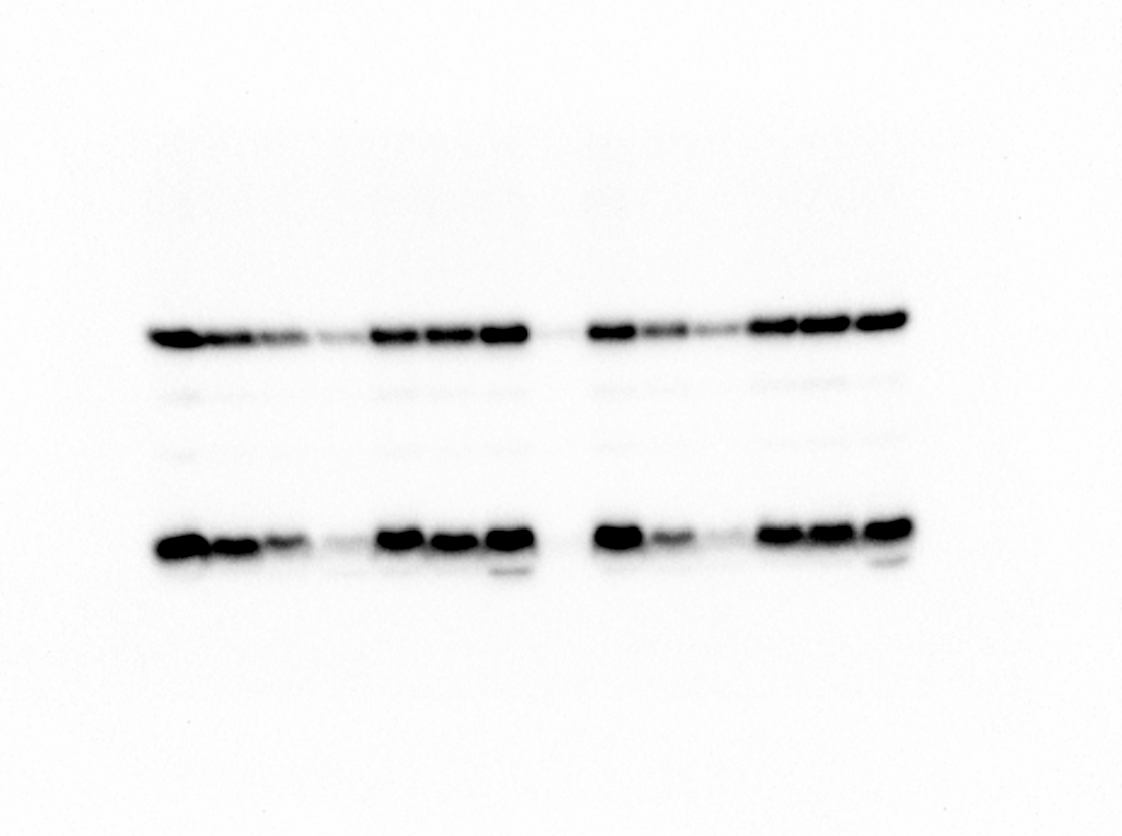

Supplement: Figure 4—figure supplement 5—source data 1. [file elife-74275-fig4-figsupp5-data1.zip › Figure 4-figure supplement 5-source data 1/figure supplement 5B/fig sup 5B Sets 2 and 3 anti-His blot 2.tif]

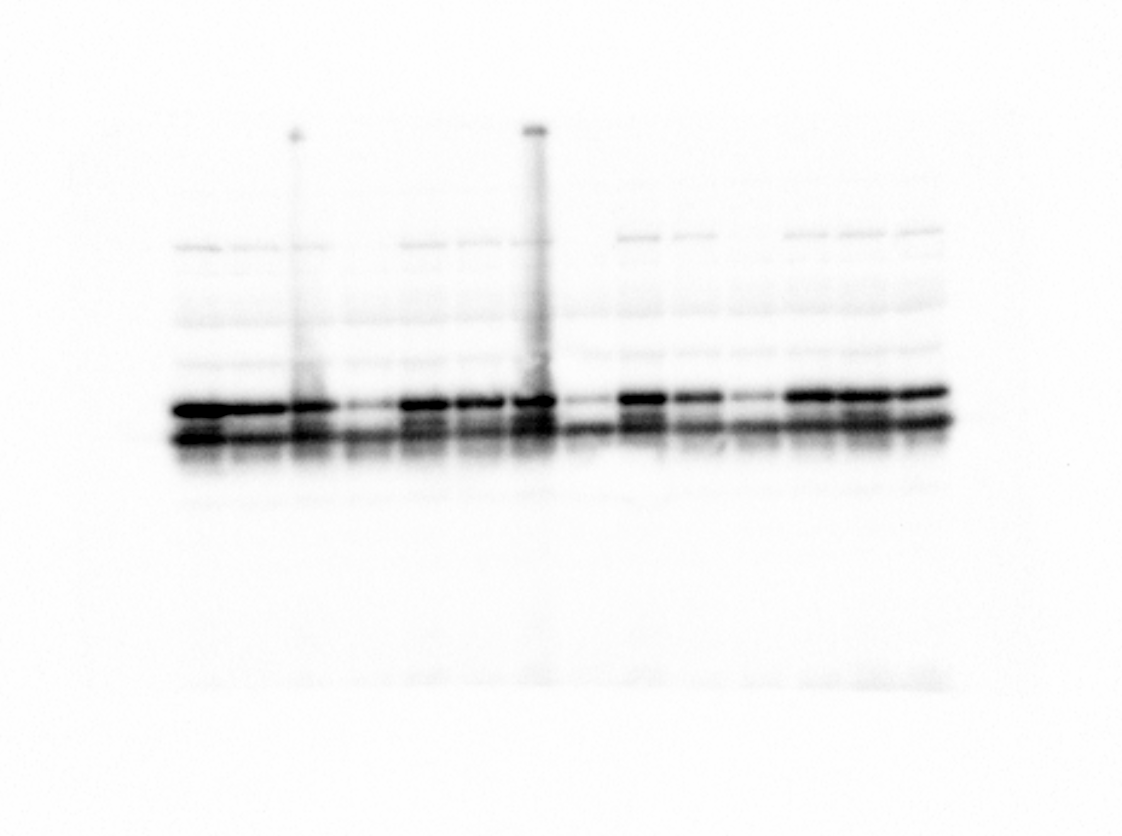

Supplement: Figure 4—figure supplement 5—source data 1. [file elife-74275-fig4-figsupp5-data1.zip › Figure 4-figure supplement 5-source data 1/figure supplement 5B/fig sup 5B Sets 2 and 3 anti-IVFA.tif]

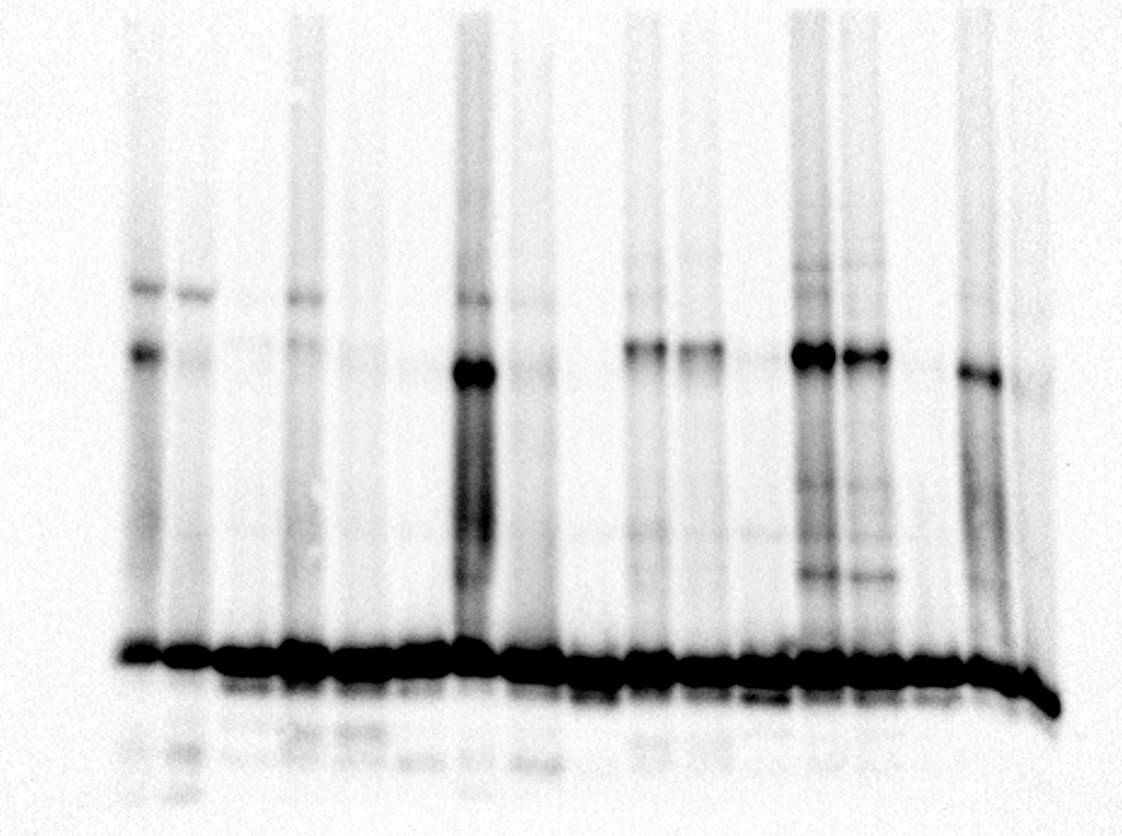

Supplement: Figure 4—figure supplement 5—source data 1. [file elife-74275-fig4-figsupp5-data1.zip › Figure 4-figure supplement 5-source data 1/figure supplement 5C/fig sup 5C anti-FLAG blot 1.tif]

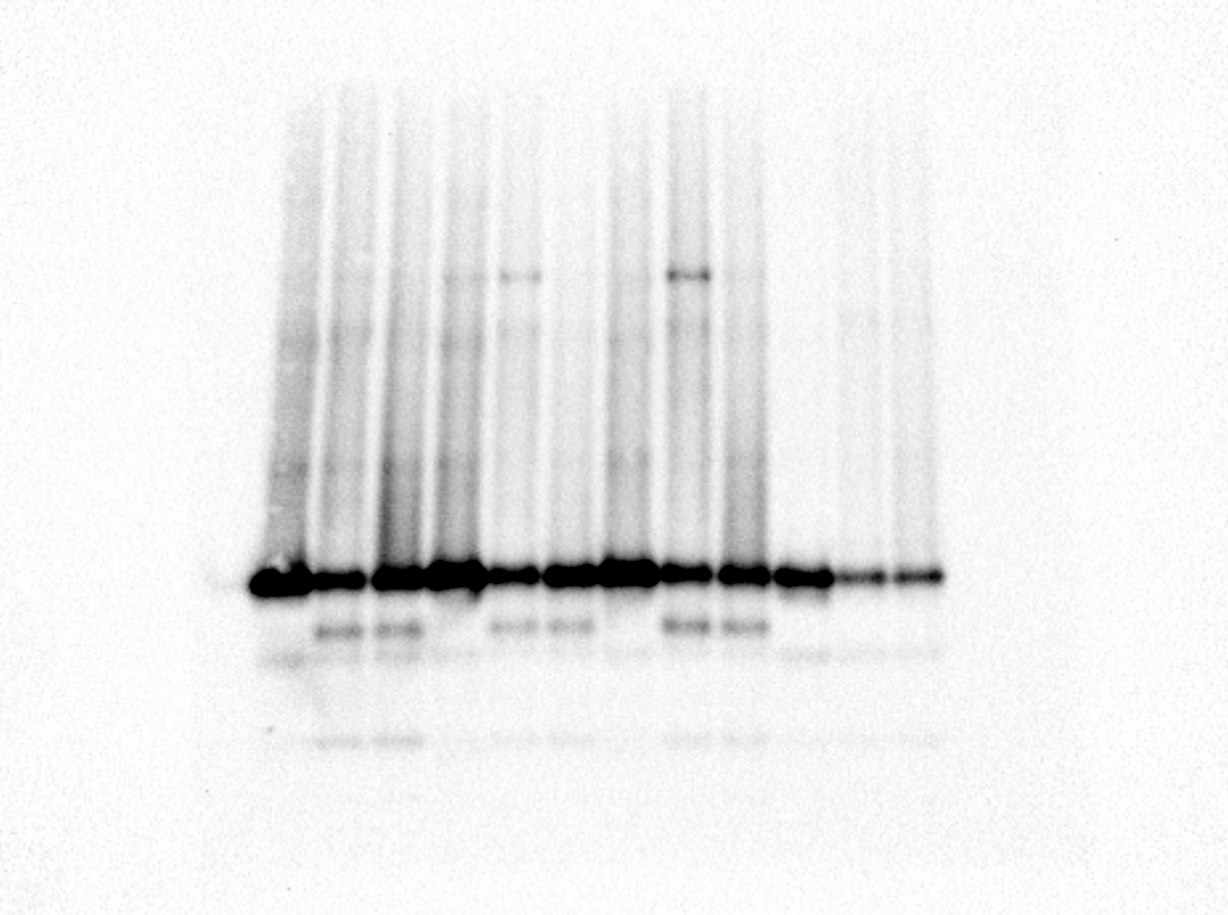

Supplement: Figure 4—figure supplement 5—source data 1. [file elife-74275-fig4-figsupp5-data1.zip › Figure 4-figure supplement 5-source data 1/figure supplement 5C/fig sup 5C anti-FLAG blot 2.tif]

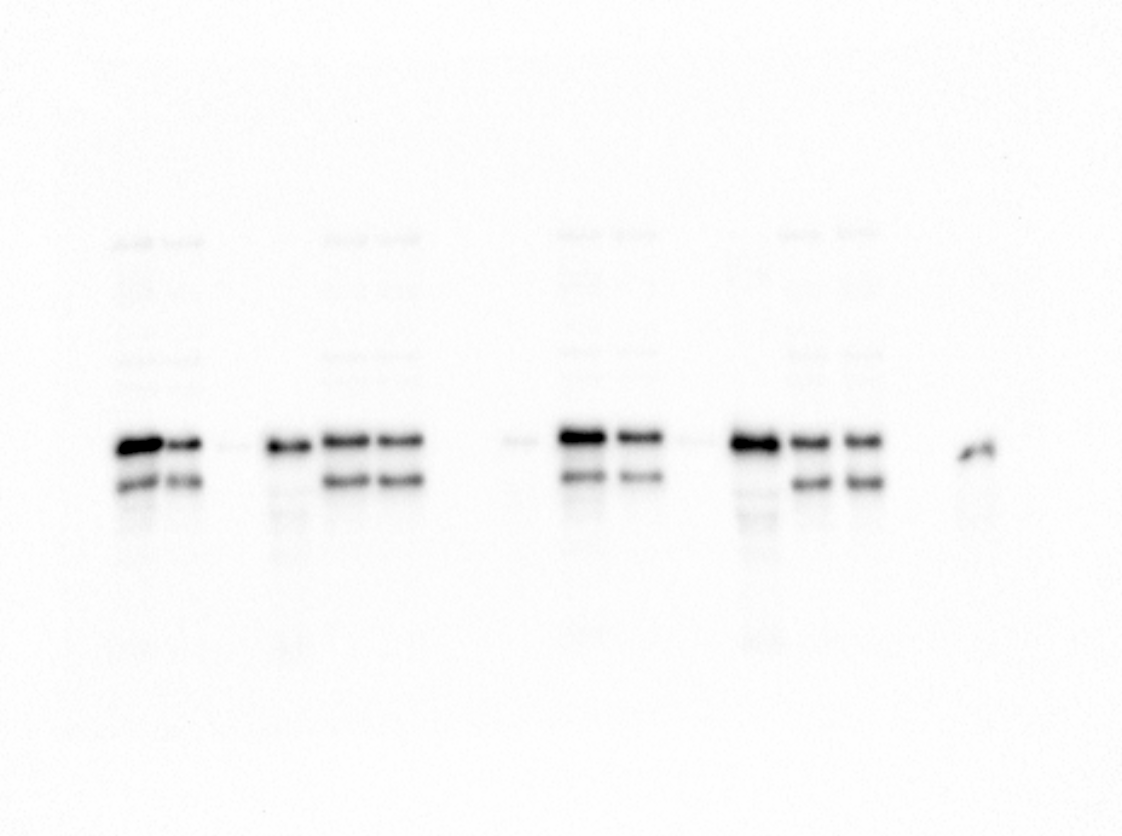

Supplement: Figure 5—source data 1. [file elife-74275-fig5-data1.zip › Figure 5-source data 1/Figure 5A/Fig5A anti GFP.tif]

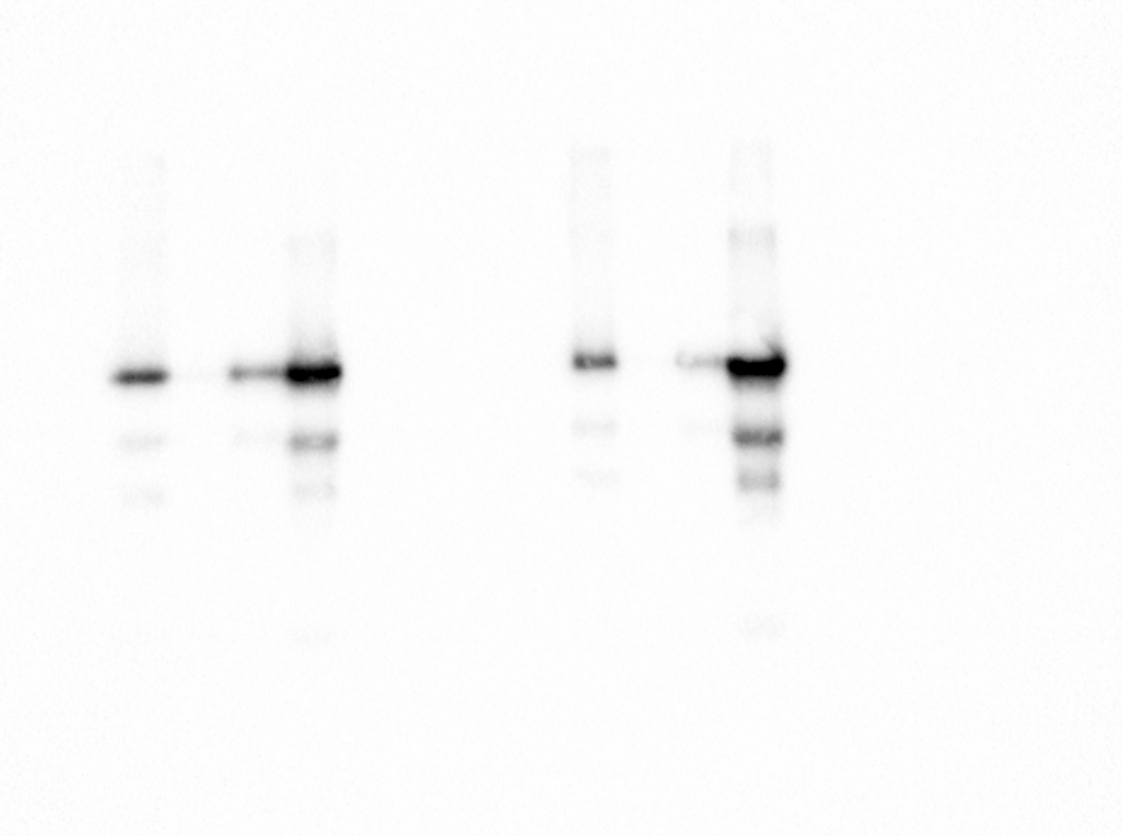

Supplement: Figure 5—source data 1. [file elife-74275-fig5-data1.zip › Figure 5-source data 1/Figure 5A/Fig5A anti-FLAG.tif]

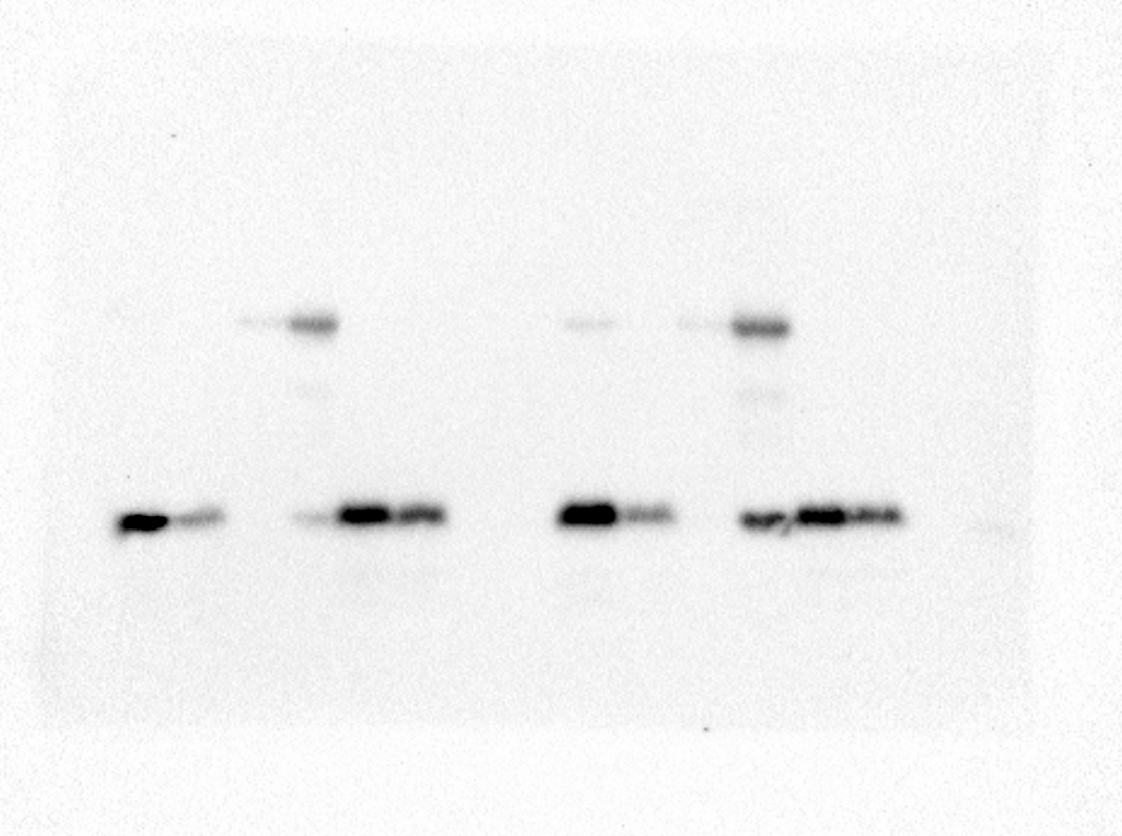

Supplement: Figure 5—source data 1. [file elife-74275-fig5-data1.zip › Figure 5-source data 1/Figure 5A/Fig5A anti-His.tif]

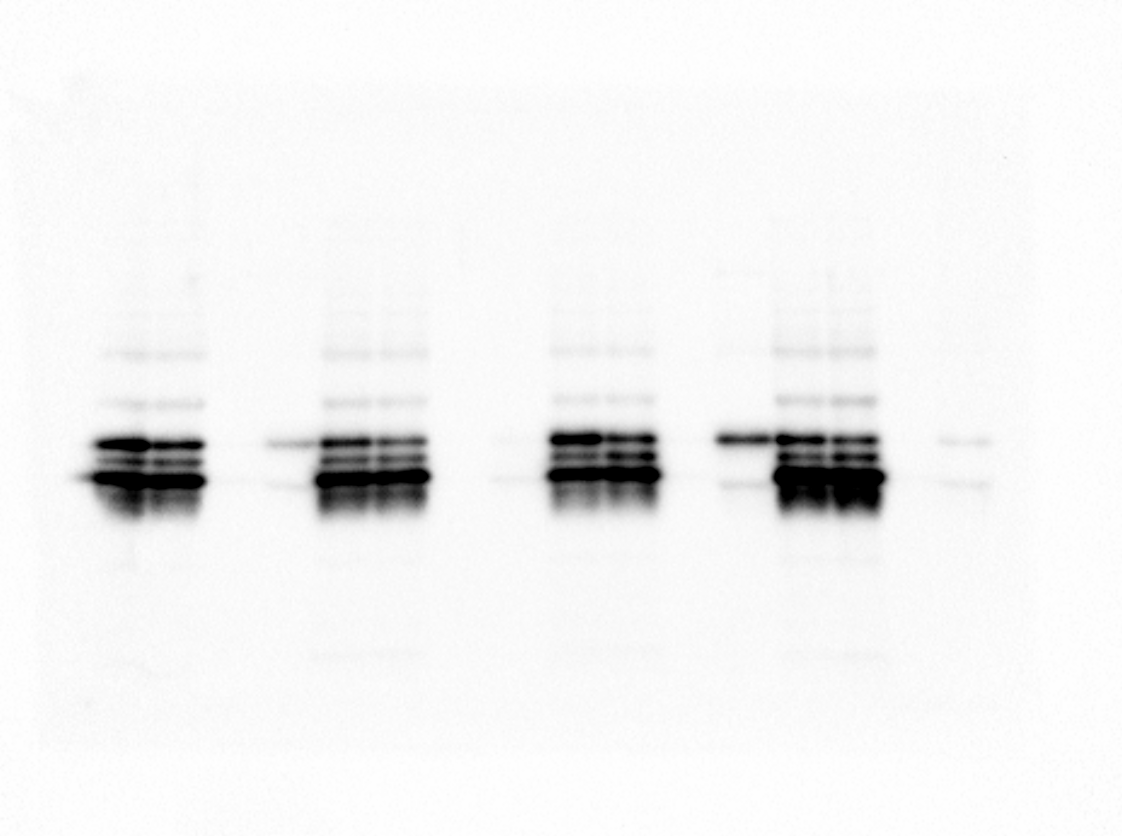

Supplement: Figure 5—source data 1. [file elife-74275-fig5-data1.zip › Figure 5-source data 1/Figure 5A/Fig5A anti-IVFA.tif]

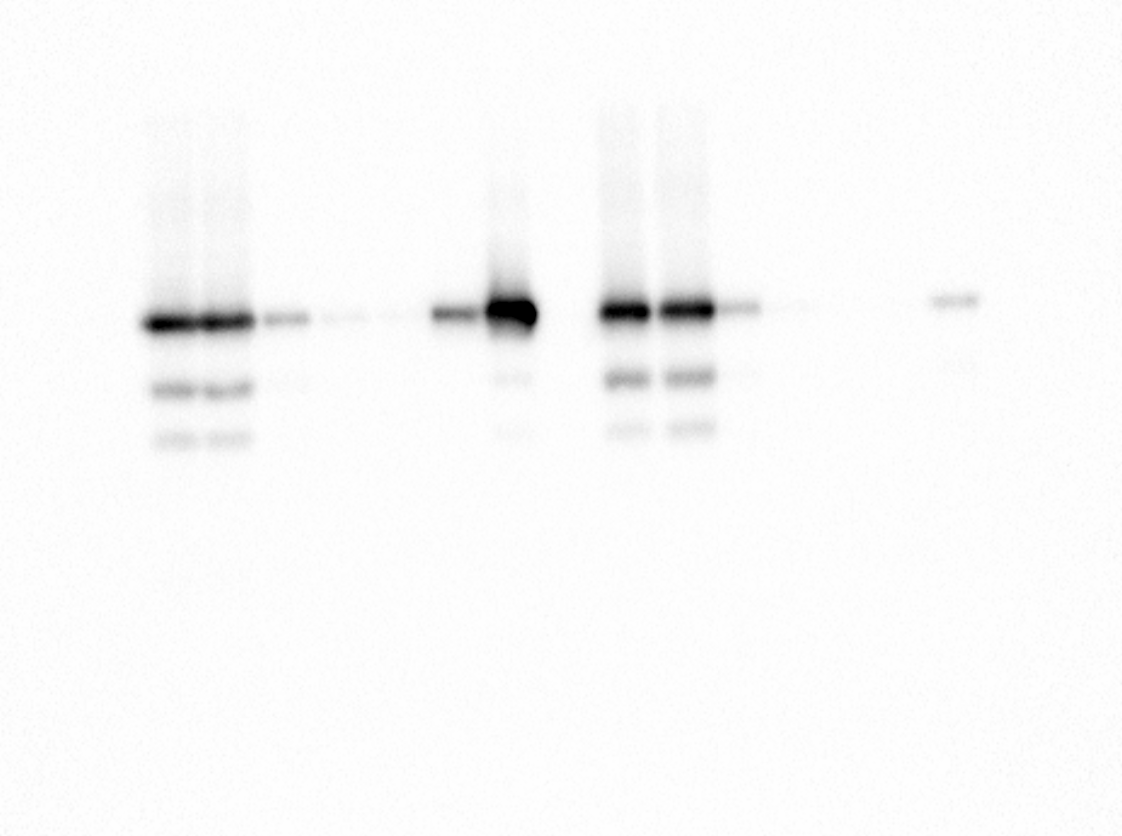

Supplement: Figure 5—figure supplement 1—source data 1. [file elife-74275-fig5-figsupp1-data1.zip › Figure 5-figure supplement 1-source data 1/fig sup 1A anti-FLAG.tif]

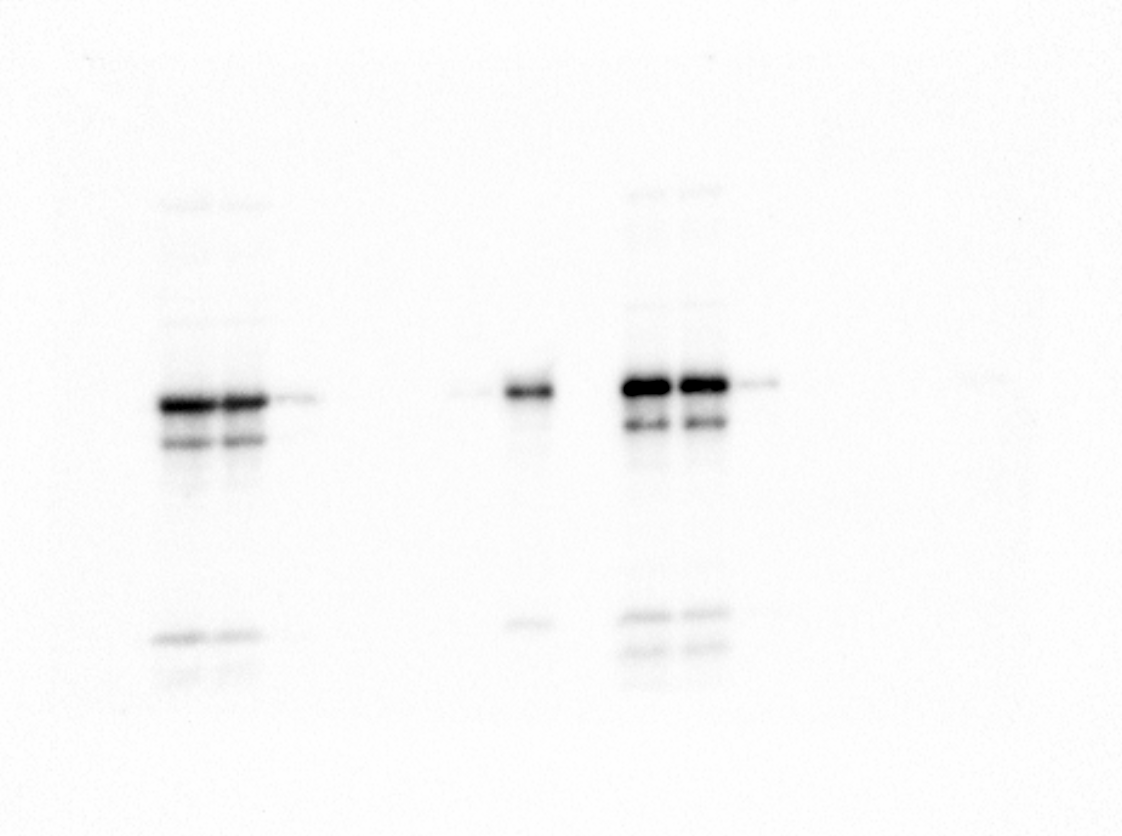

Supplement: Figure 5—figure supplement 1—source data 1. [file elife-74275-fig5-figsupp1-data1.zip › Figure 5-figure supplement 1-source data 1/fig sup 1A anti-GFP.tif]

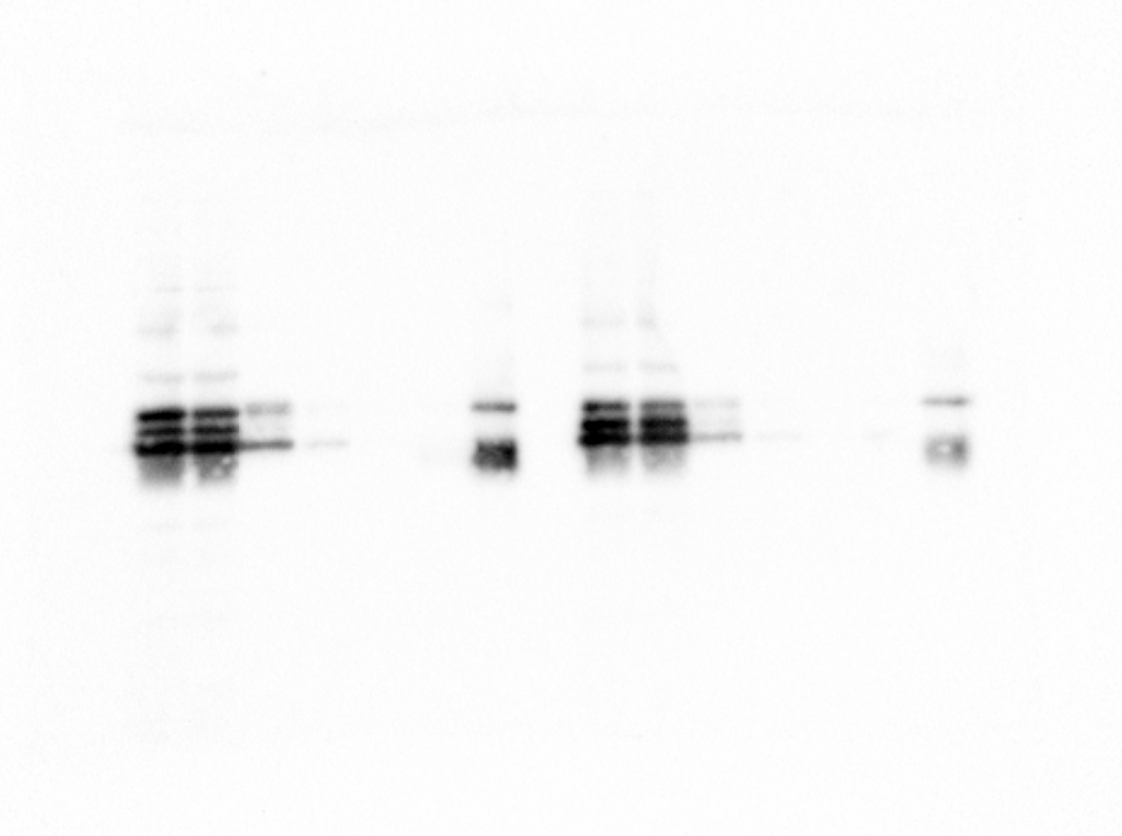

Supplement: Figure 5—figure supplement 1—source data 1. [file elife-74275-fig5-figsupp1-data1.zip › Figure 5-figure supplement 1-source data 1/fig sup 1A anti-IVFA.tif]

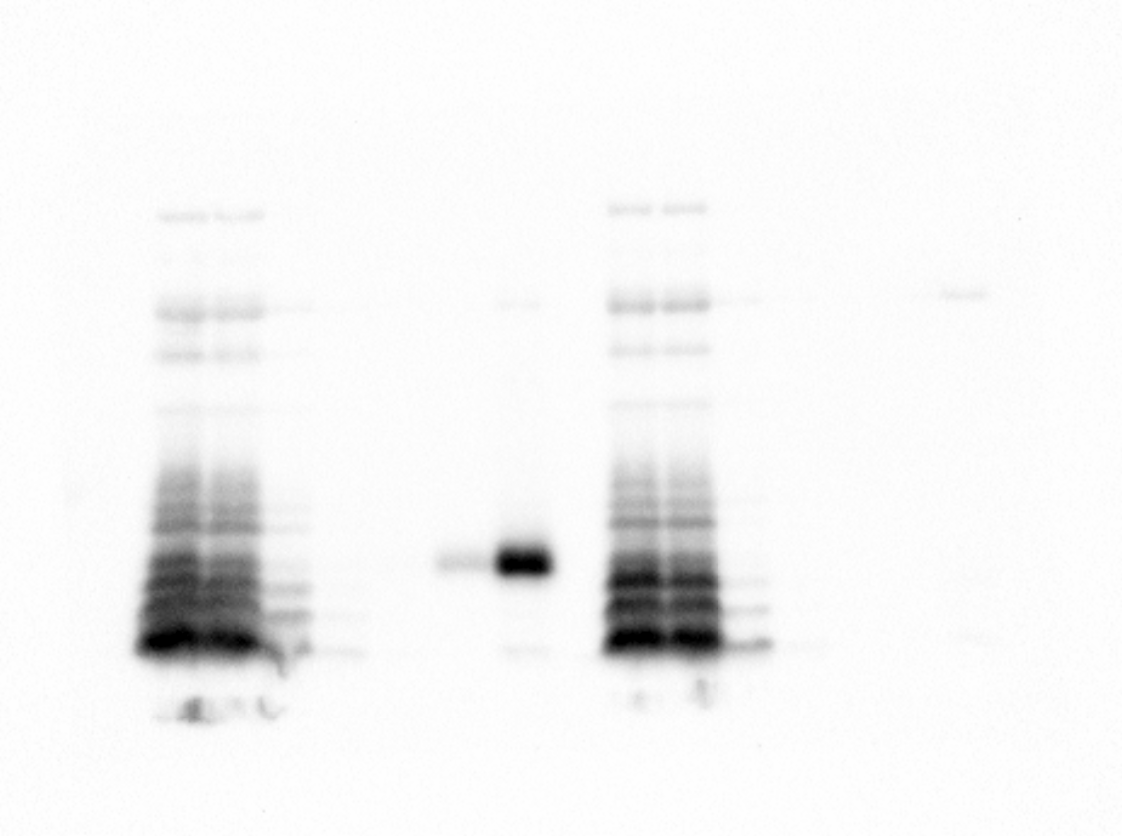

Supplement: Figure 5—figure supplement 1—source data 1. [file elife-74275-fig5-figsupp1-data1.zip › Figure 5-figure supplement 1-source data 1/fig sup 1A anti-ProsigK.tif]

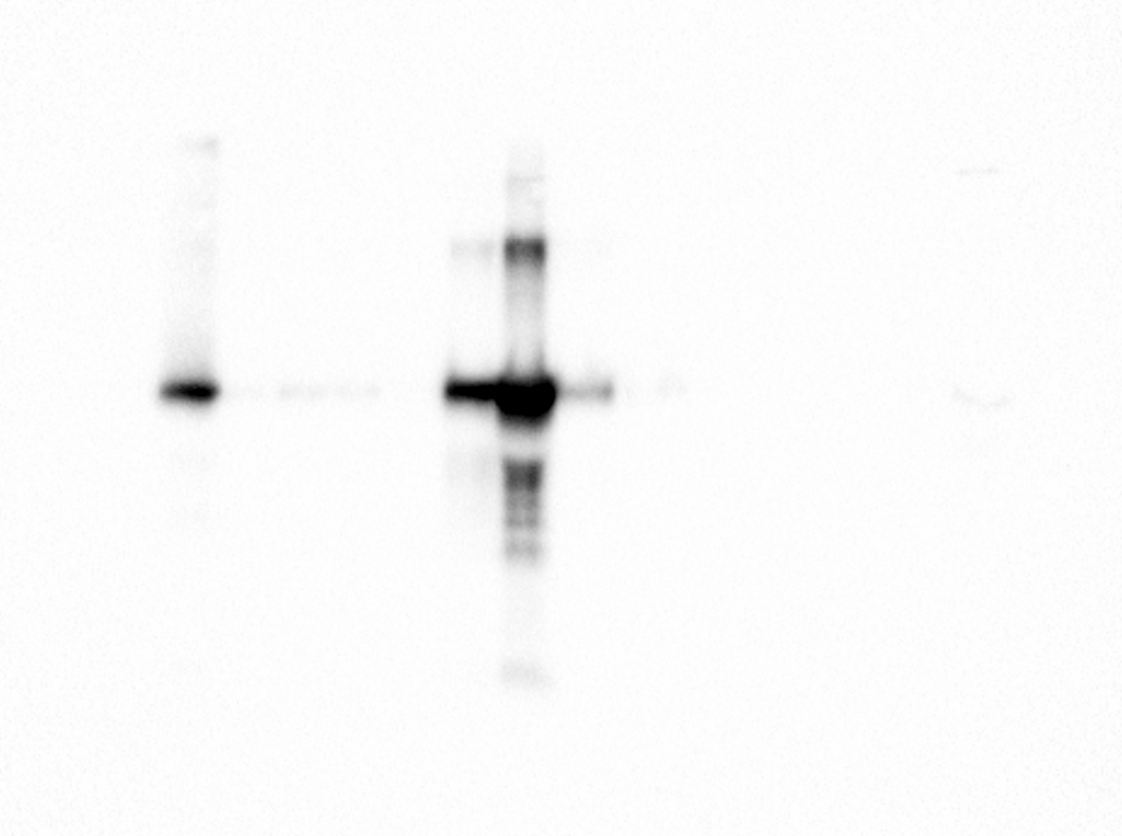

Supplement: Figure 5—figure supplement 1—source data 1. [file elife-74275-fig5-figsupp1-data1.zip › Figure 5-figure supplement 1-source data 1/fig sup 1B anti-FLAG.tif]

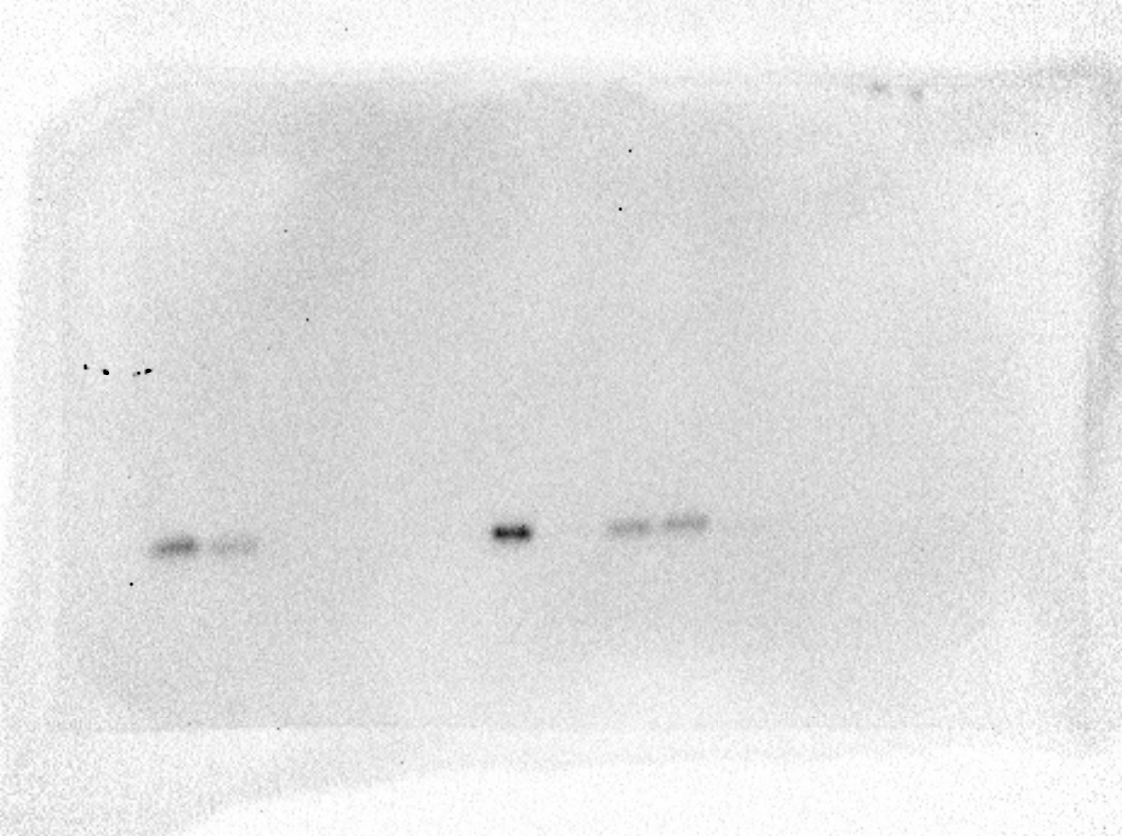

Supplement: Figure 5—figure supplement 1—source data 1. [file elife-74275-fig5-figsupp1-data1.zip › Figure 5-figure supplement 1-source data 1/fig sup 1B anti-His.tif]

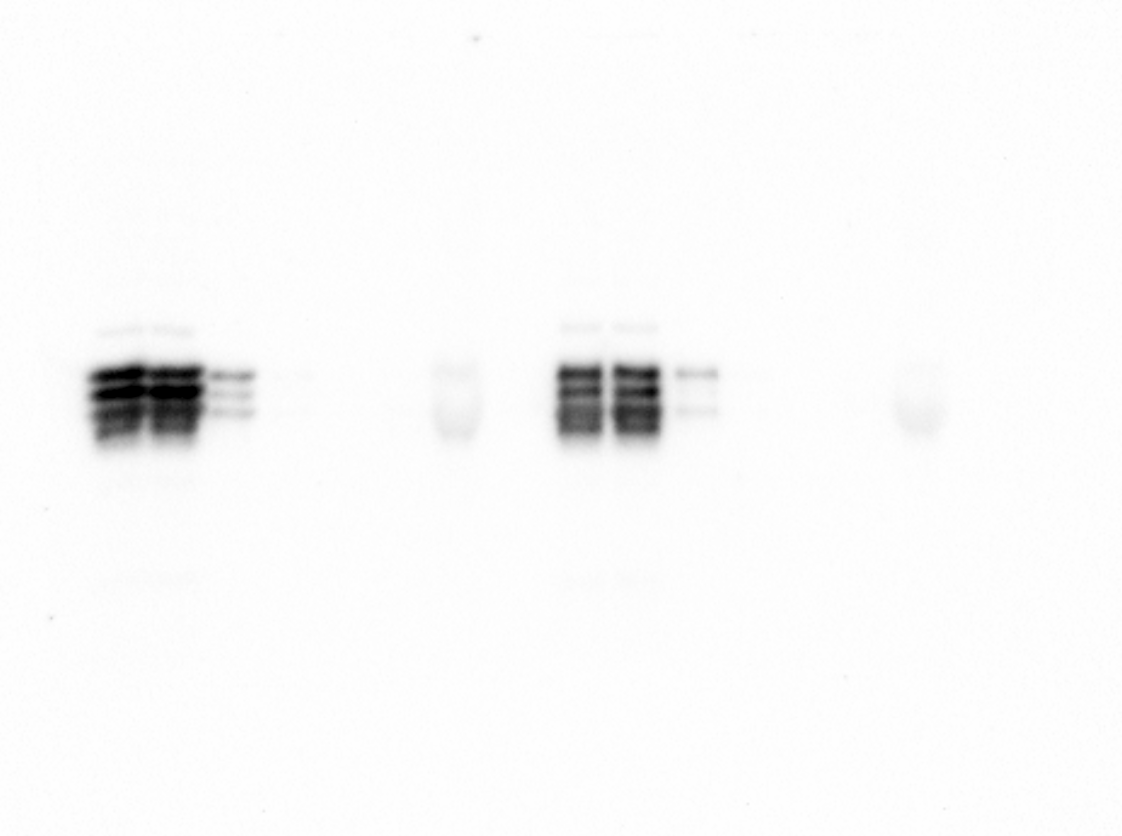

Supplement: Figure 5—figure supplement 1—source data 1. [file elife-74275-fig5-figsupp1-data1.zip › Figure 5-figure supplement 1-source data 1/fig sup 1B anti-IVFA.tif]

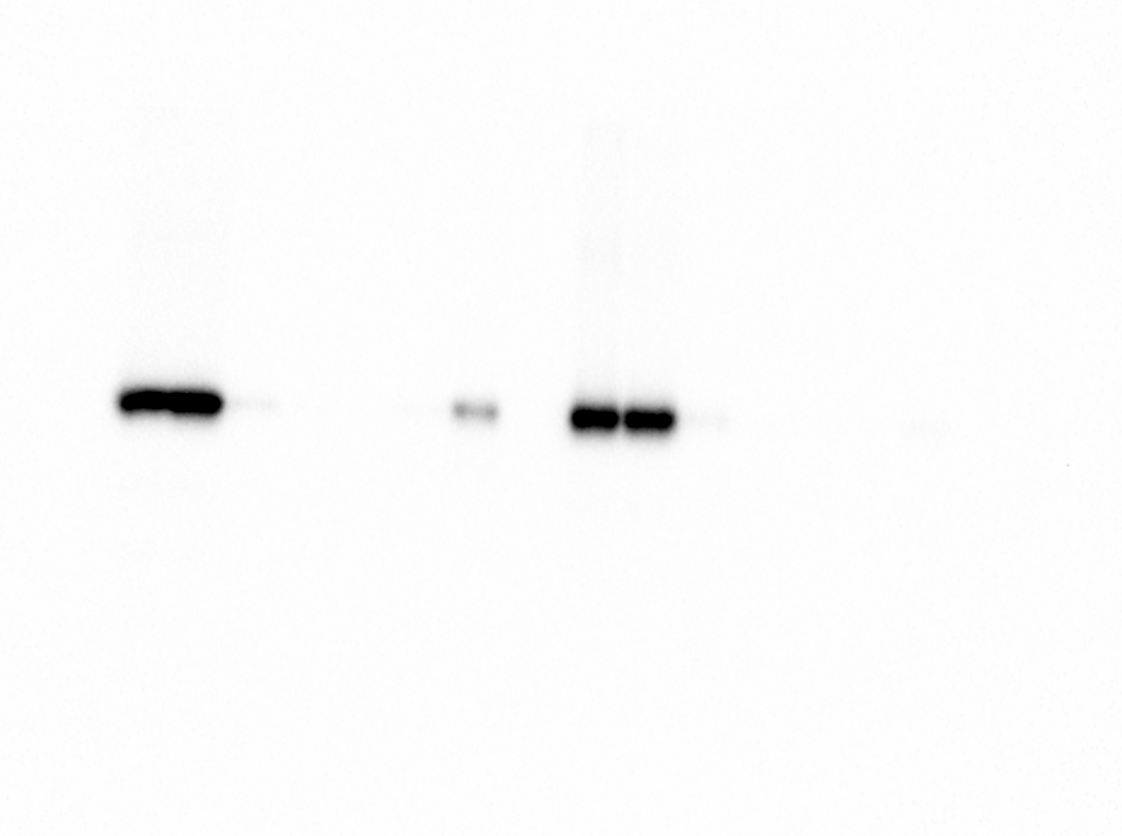

Supplement: Figure 5—figure supplement 1—source data 1. [file elife-74275-fig5-figsupp1-data1.zip › Figure 5-figure supplement 1-source data 1/fig sup 1C anti-FLAG.tif]

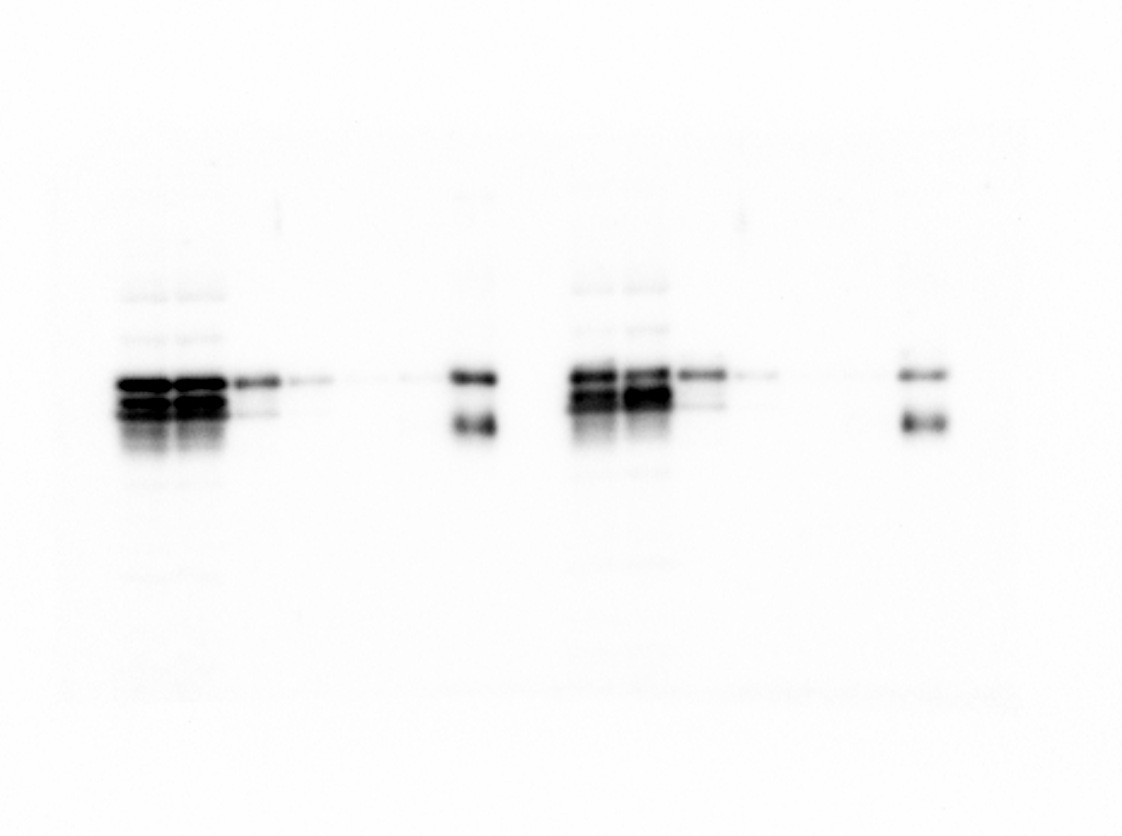

Supplement: Figure 5—figure supplement 1—source data 1. [file elife-74275-fig5-figsupp1-data1.zip › Figure 5-figure supplement 1-source data 1/fig sup 1C anti-IVFA.tif]

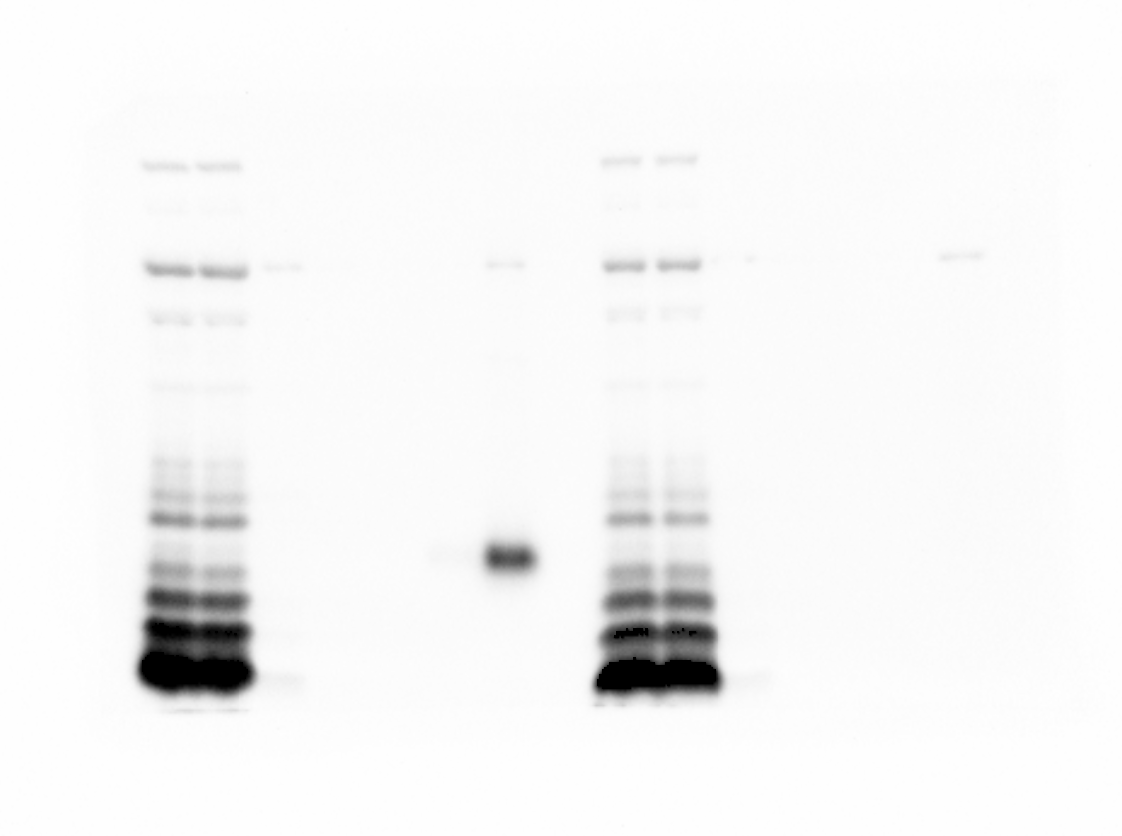

Supplement: Figure 5—figure supplement 1—source data 1. [file elife-74275-fig5-figsupp1-data1.zip › Figure 5-figure supplement 1-source data 1/fig sup 1C anti-ProsigK.tif]

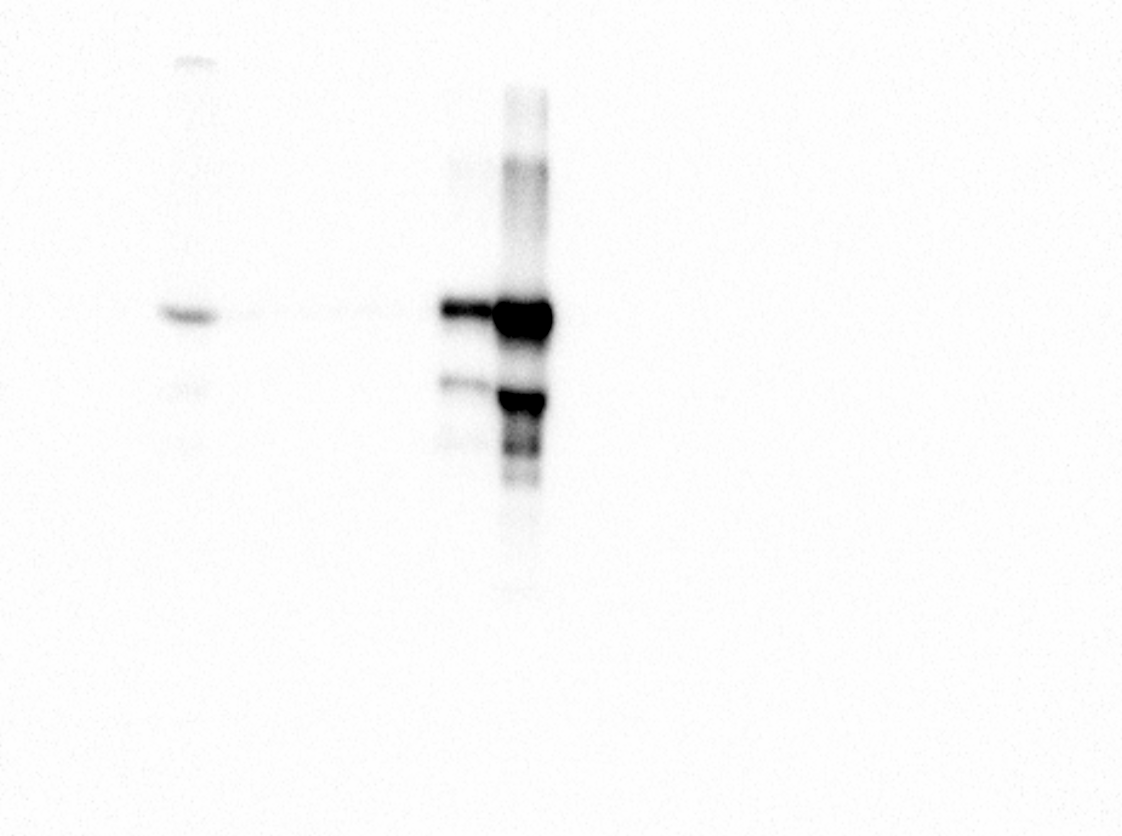

Supplement: Figure 5—figure supplement 2—source data 1. [file elife-74275-fig5-figsupp2-data1.zip › Figure 5-figure supplement 2-source data 1/fig sup 2A anti-FLAG.tif]

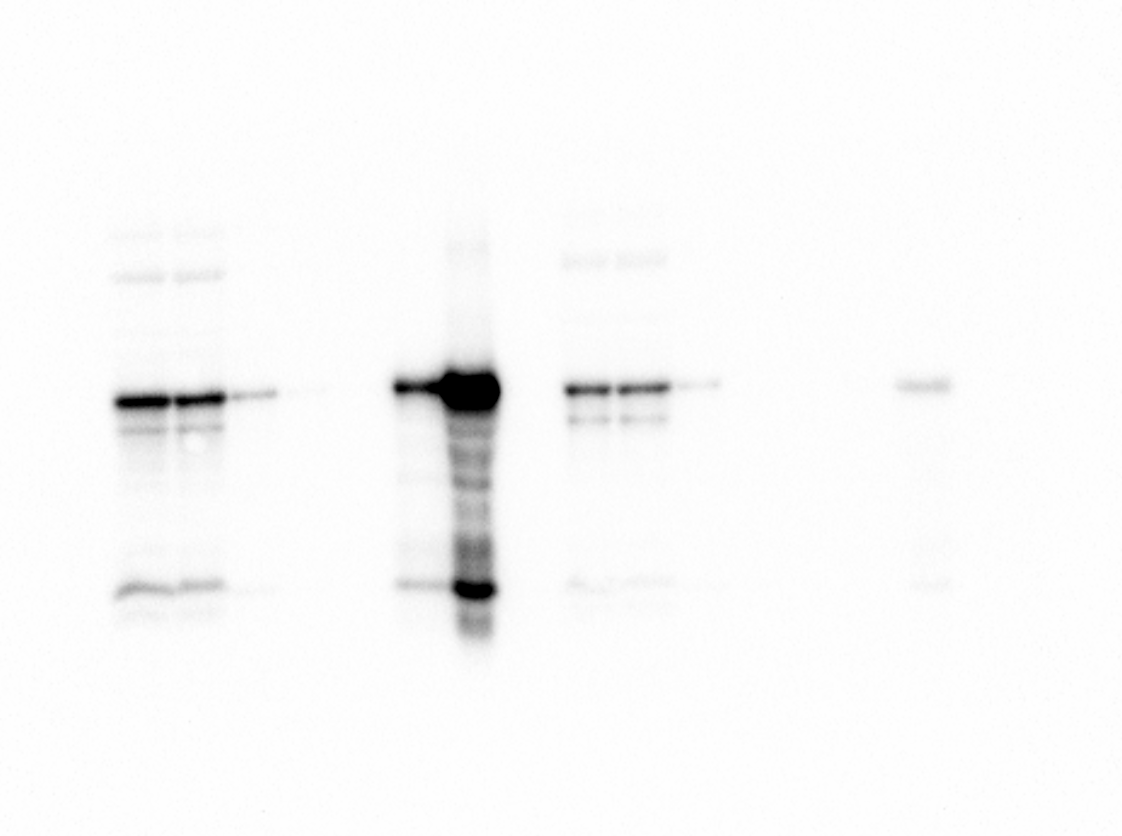

Supplement: Figure 5—figure supplement 2—source data 1. [file elife-74275-fig5-figsupp2-data1.zip › Figure 5-figure supplement 2-source data 1/fig sup 2A anti-GFP.tif]

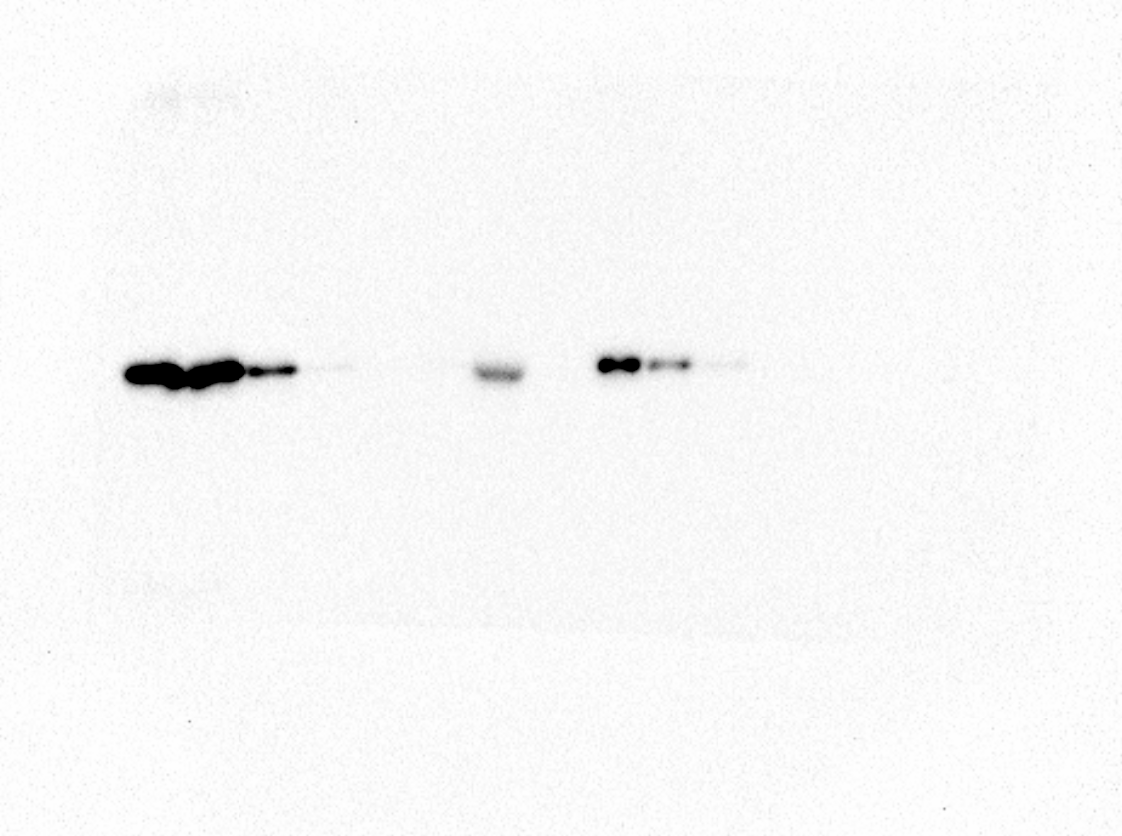

Supplement: Figure 5—figure supplement 2—source data 1. [file elife-74275-fig5-figsupp2-data1.zip › Figure 5-figure supplement 2-source data 1/fig sup 2A anti-His.tif]

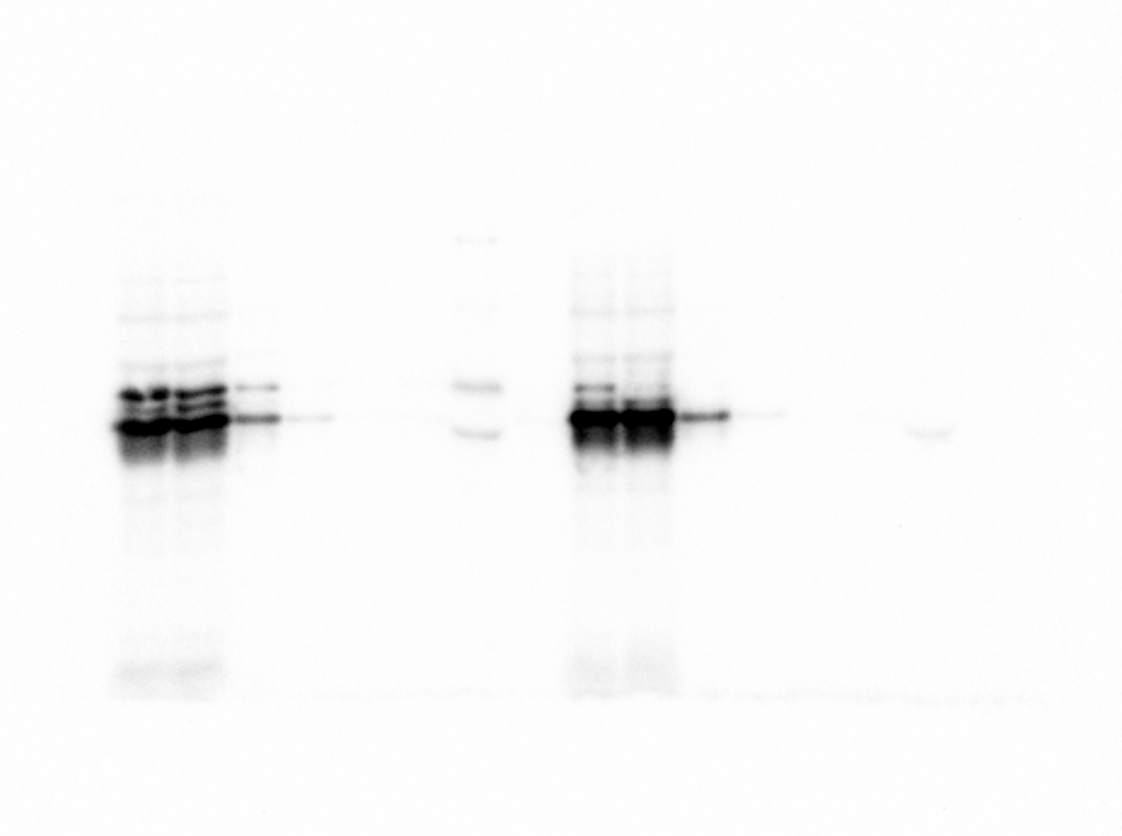

Supplement: Figure 5—figure supplement 2—source data 1. [file elife-74275-fig5-figsupp2-data1.zip › Figure 5-figure supplement 2-source data 1/fig sup 2A anti-IVFA.tif]

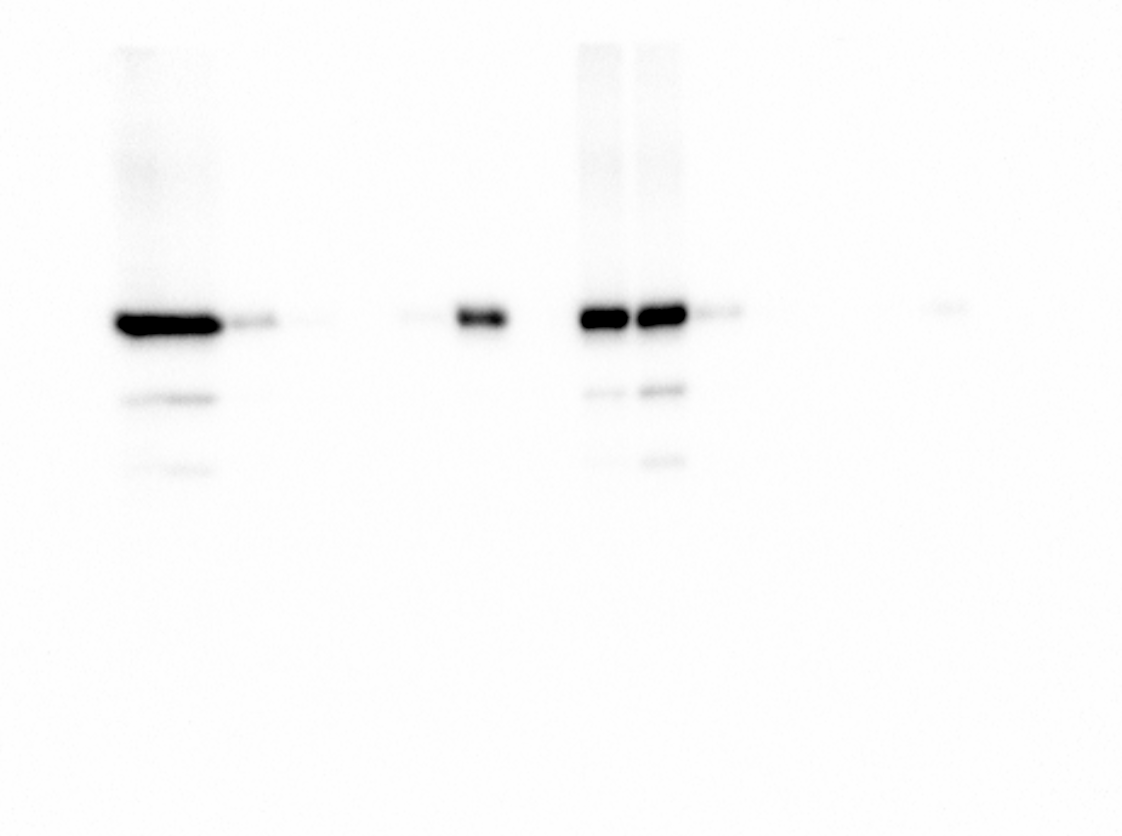

Supplement: Figure 5—figure supplement 2—source data 1. [file elife-74275-fig5-figsupp2-data1.zip › Figure 5-figure supplement 2-source data 1/fig sup 2B anti-FLAG.tif]

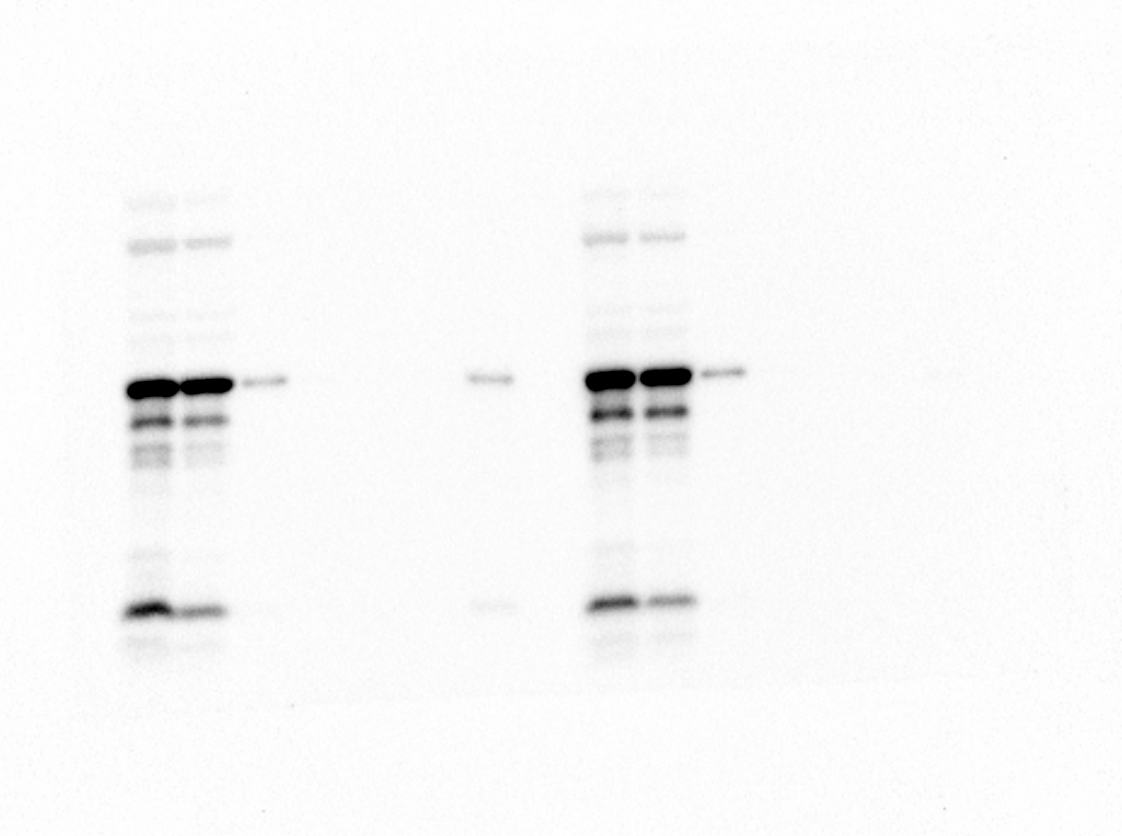

Supplement: Figure 5—figure supplement 2—source data 1. [file elife-74275-fig5-figsupp2-data1.zip › Figure 5-figure supplement 2-source data 1/fig sup 2B anti-GFP.tif]

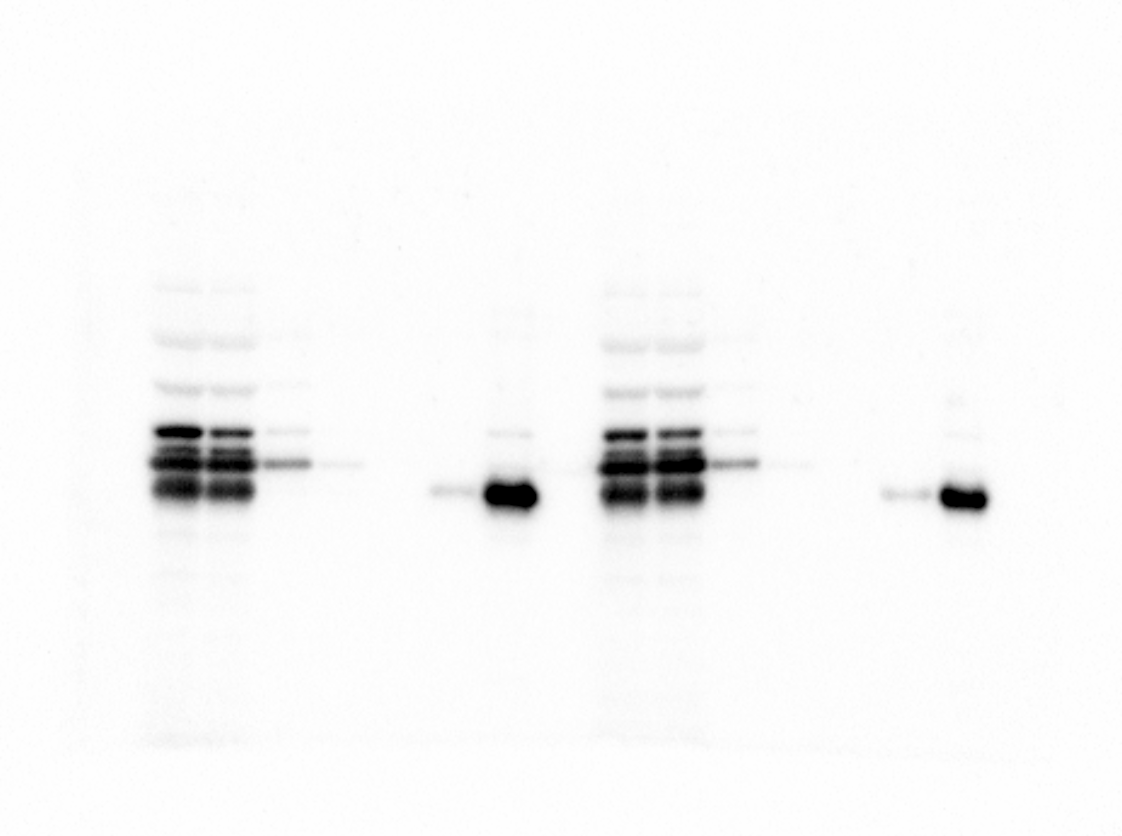

Supplement: Figure 5—figure supplement 2—source data 1. [file elife-74275-fig5-figsupp2-data1.zip › Figure 5-figure supplement 2-source data 1/fig sup 2B anti-IVFA.tif]

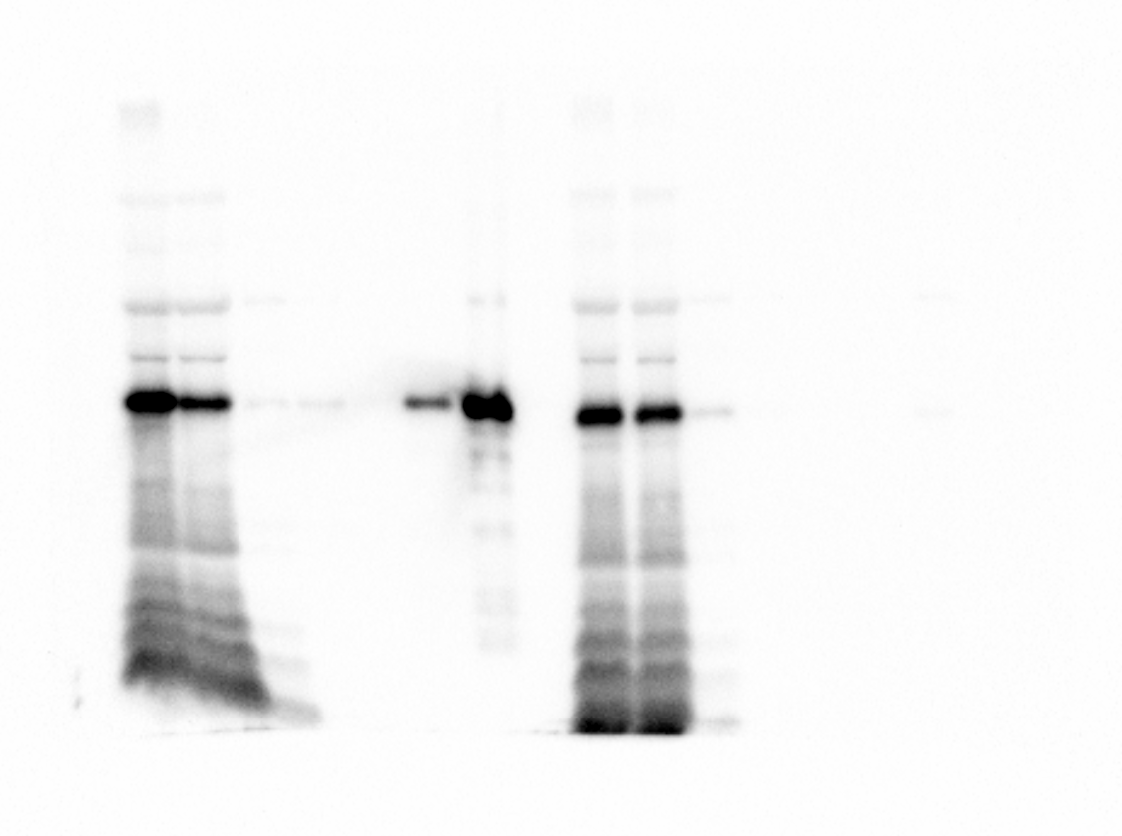

Supplement: Figure 5—figure supplement 2—source data 1. [file elife-74275-fig5-figsupp2-data1.zip › Figure 5-figure supplement 2-source data 1/fig sup 2B anti-ProsigK.tif]

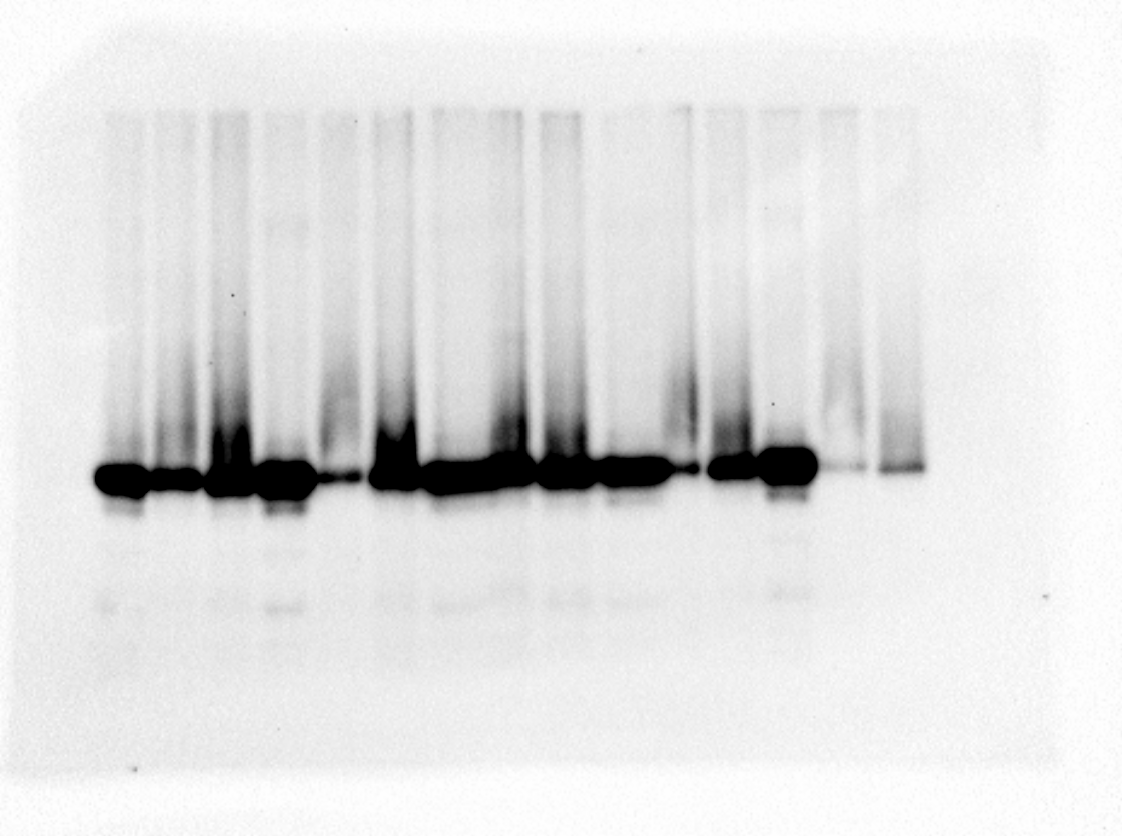

Supplement: Figure 5—figure supplement 3—source data 1. [file elife-74275-fig5-figsupp3-data1.zip › Figure 5-figure supplement 3-source data 1/figure supplement 3A/fig sup 3A anti-FLAG blot 1.tif]

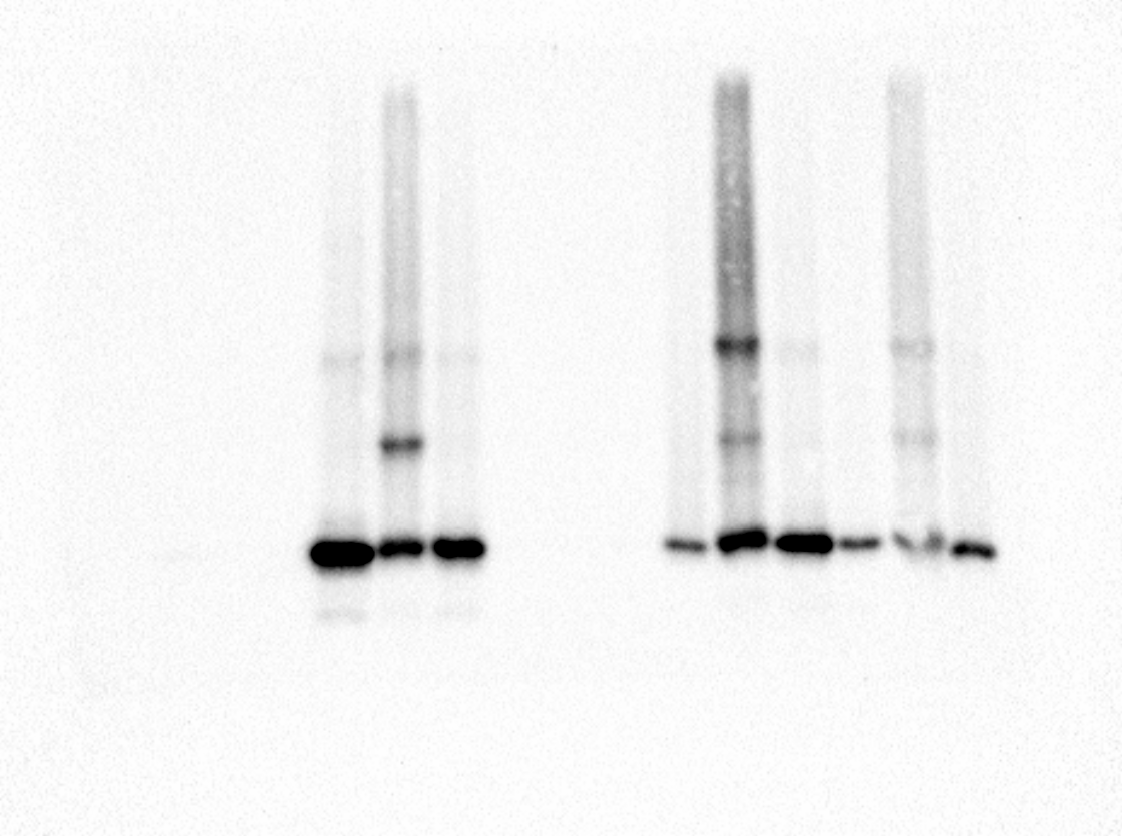

Supplement: Figure 5—figure supplement 3—source data 1. [file elife-74275-fig5-figsupp3-data1.zip › Figure 5-figure supplement 3-source data 1/figure supplement 3A/fig sup 3A anti-FLAG blot 2.tif]

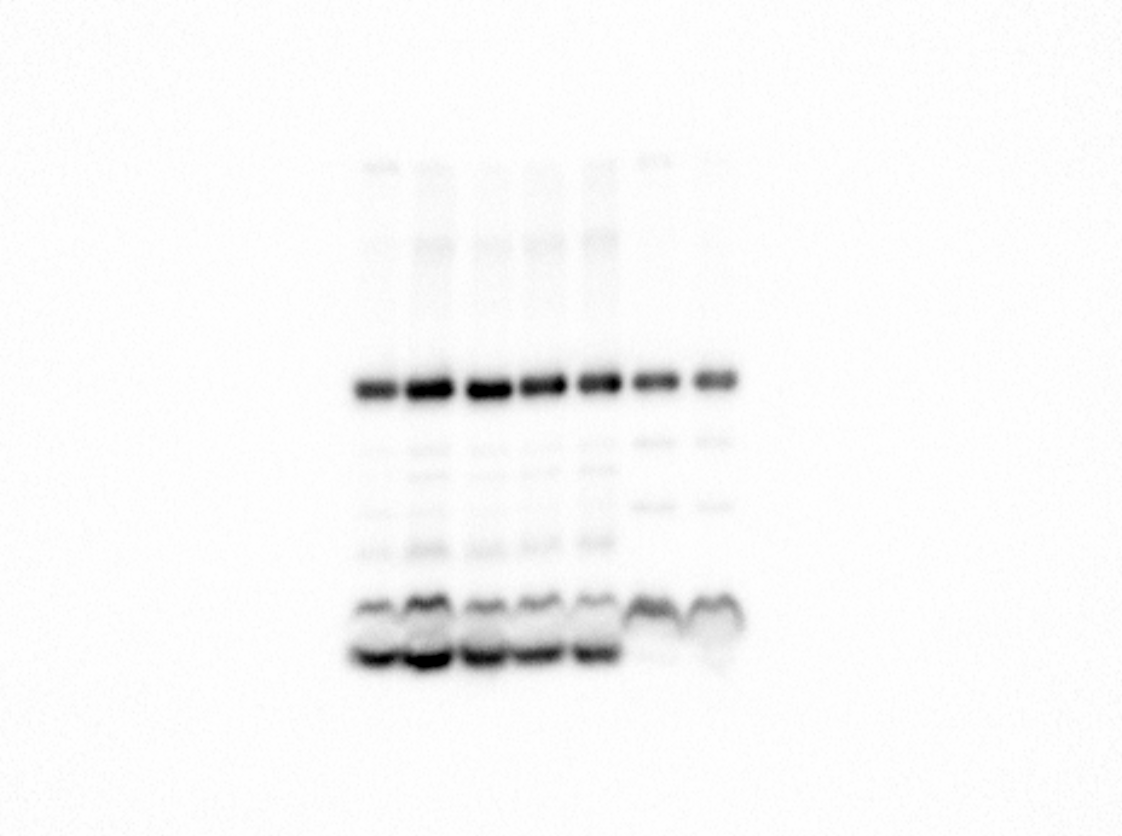

Supplement: Figure 5—figure supplement 3—source data 1. [file elife-74275-fig5-figsupp3-data1.zip › Figure 5-figure supplement 3-source data 1/figure supplement 3B/fig sup 3B Set 1 anti-His.tif]

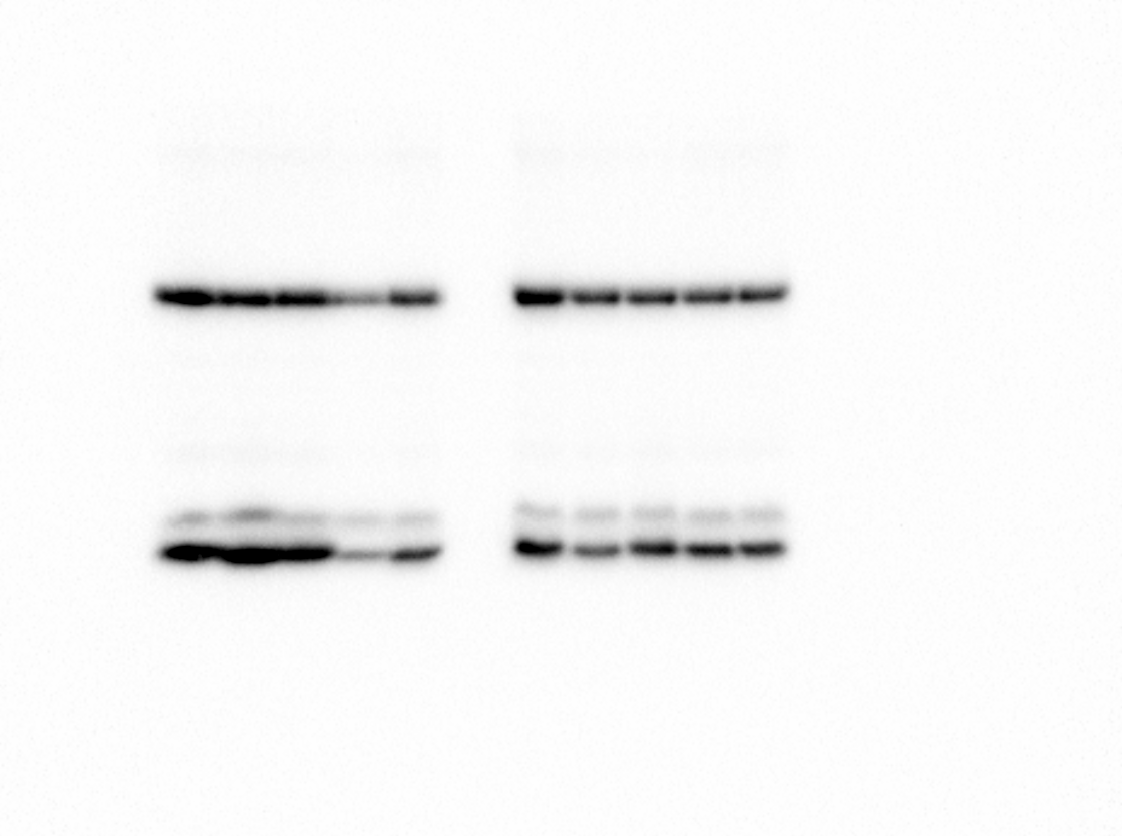

Supplement: Figure 5—figure supplement 3—source data 1. [file elife-74275-fig5-figsupp3-data1.zip › Figure 5-figure supplement 3-source data 1/figure supplement 3B/fig sup 3B Sets 2 and 3 anti-His.tif]

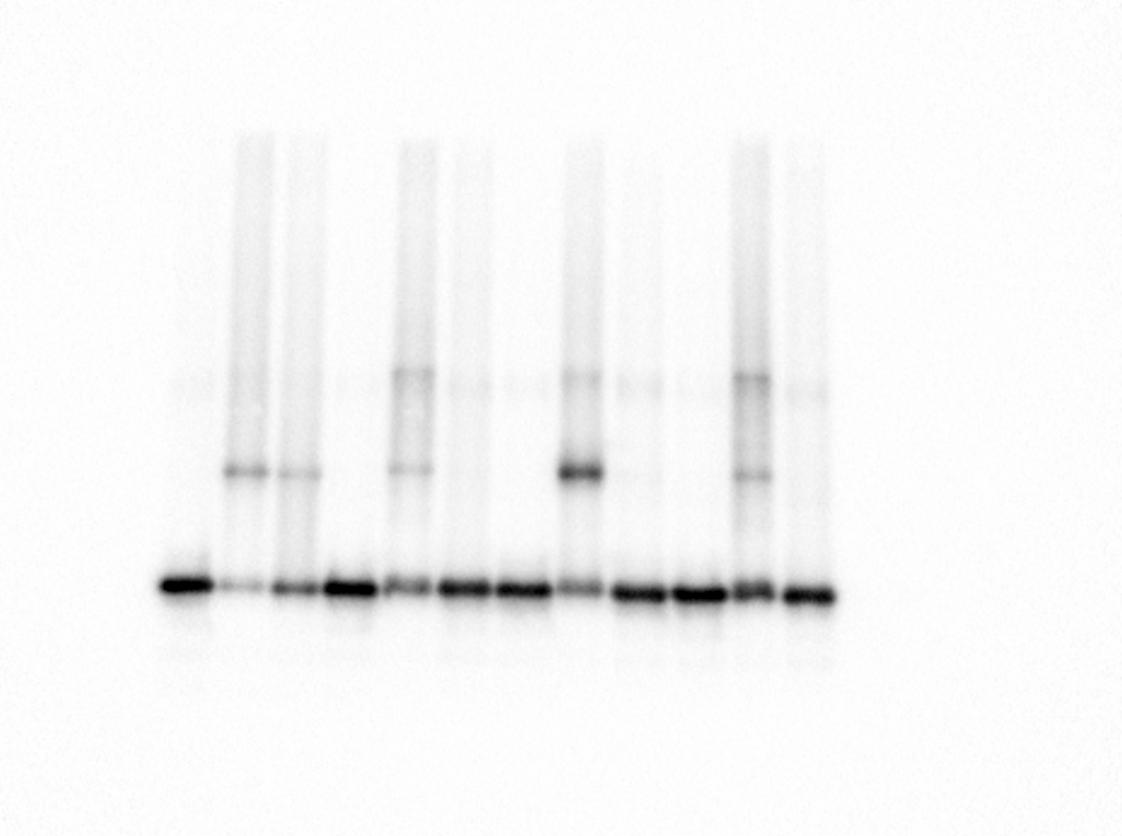

Supplement: Figure 5—figure supplement 3—source data 1. [file elife-74275-fig5-figsupp3-data1.zip › Figure 5-figure supplement 3-source data 1/figure supplement 3C/pSO120 and 130 anti-FLAG 15-30min Set1.tif]

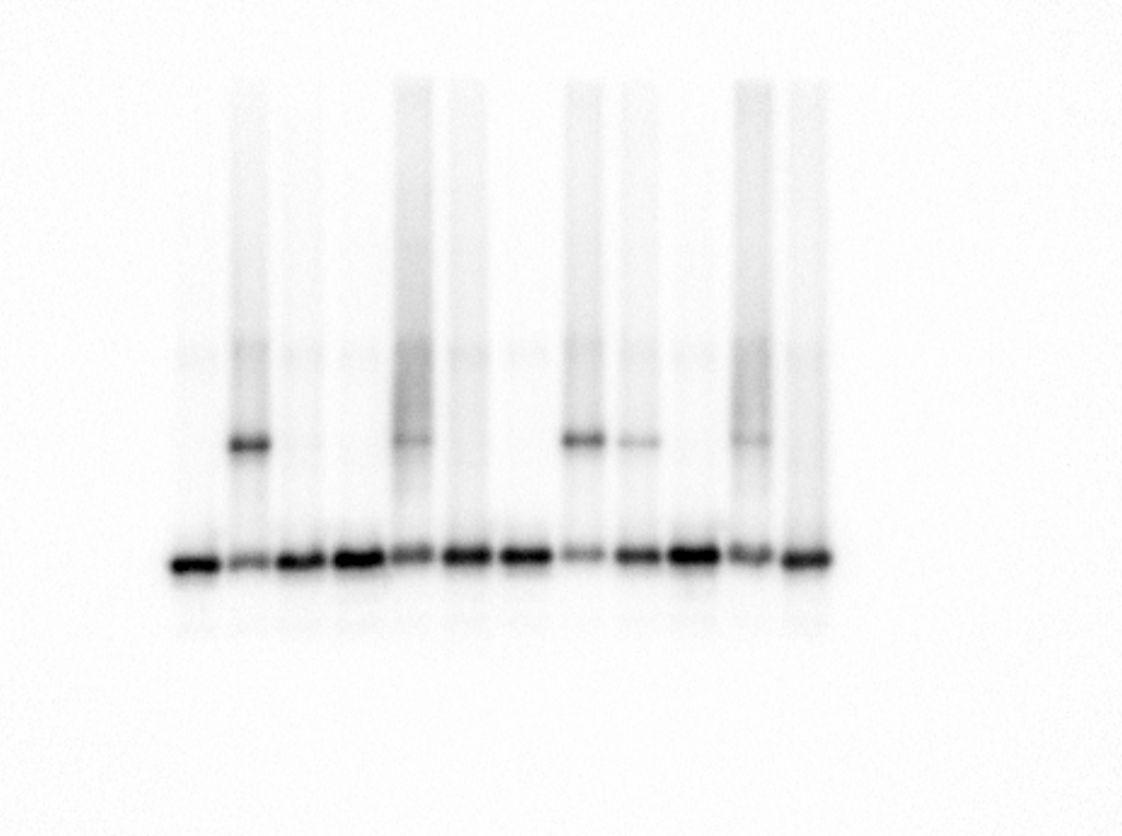

Supplement: Figure 5—figure supplement 3—source data 1. [file elife-74275-fig5-figsupp3-data1.zip › Figure 5-figure supplement 3-source data 1/figure supplement 3C/pSO120 and 130 anti-FLAG 15-30min Set2.tif]

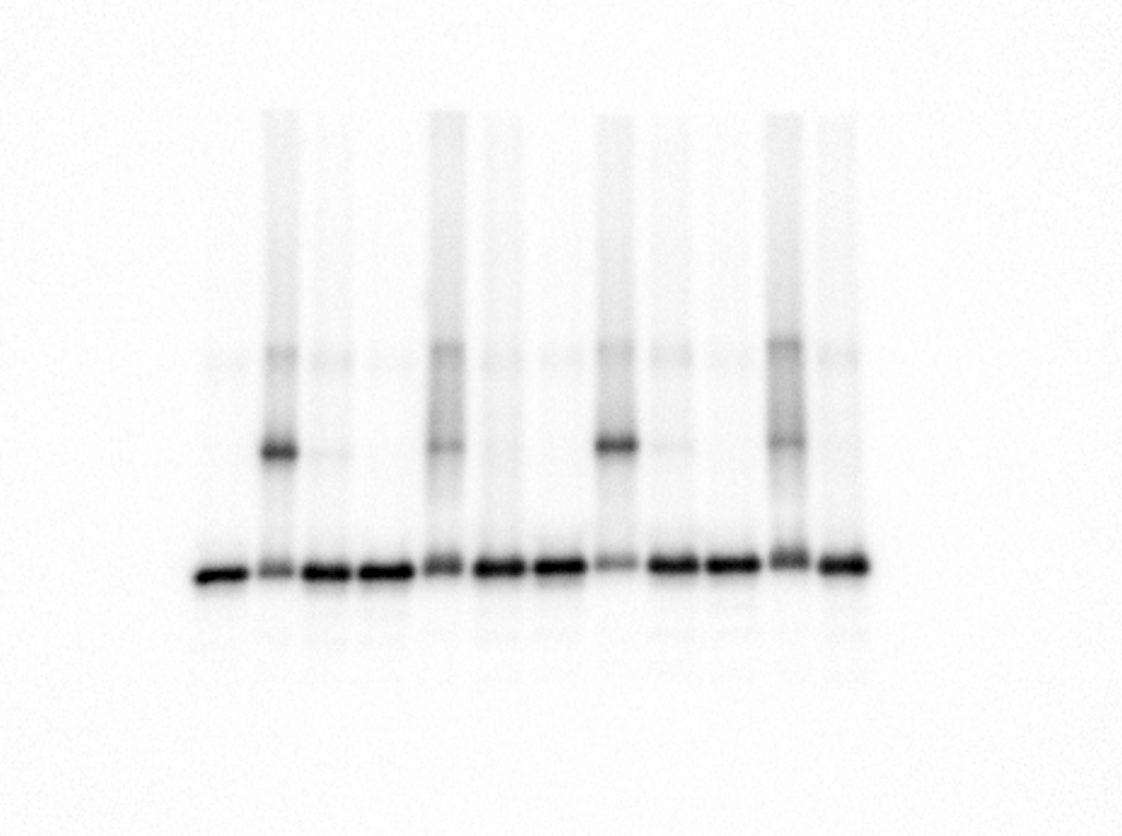

Supplement: Figure 5—figure supplement 3—source data 1. [file elife-74275-fig5-figsupp3-data1.zip › Figure 5-figure supplement 3-source data 1/figure supplement 3C/pSO120 and 130 anti-FLAG 45-60min Set1.tif]

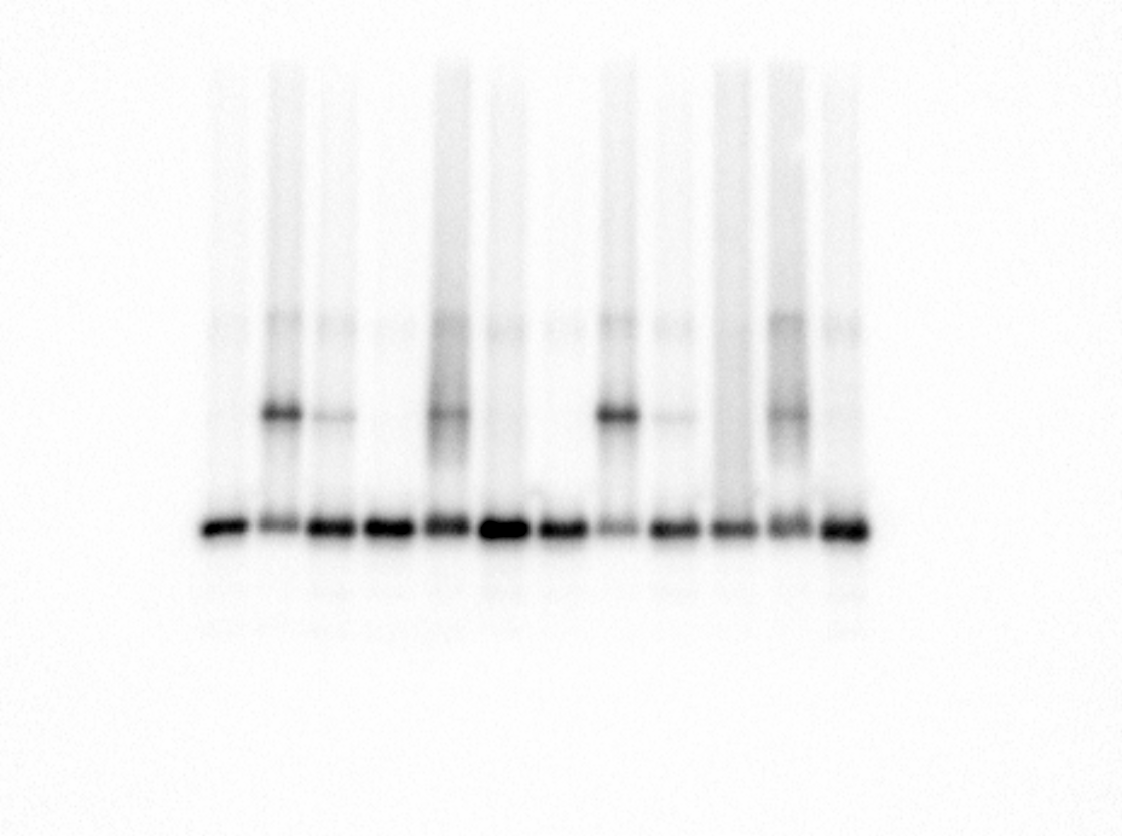

Supplement: Figure 5—figure supplement 3—source data 1. [file elife-74275-fig5-figsupp3-data1.zip › Figure 5-figure supplement 3-source data 1/figure supplement 3C/pSO120 and 130 anti-FLAG 45-60min Set2.tif]

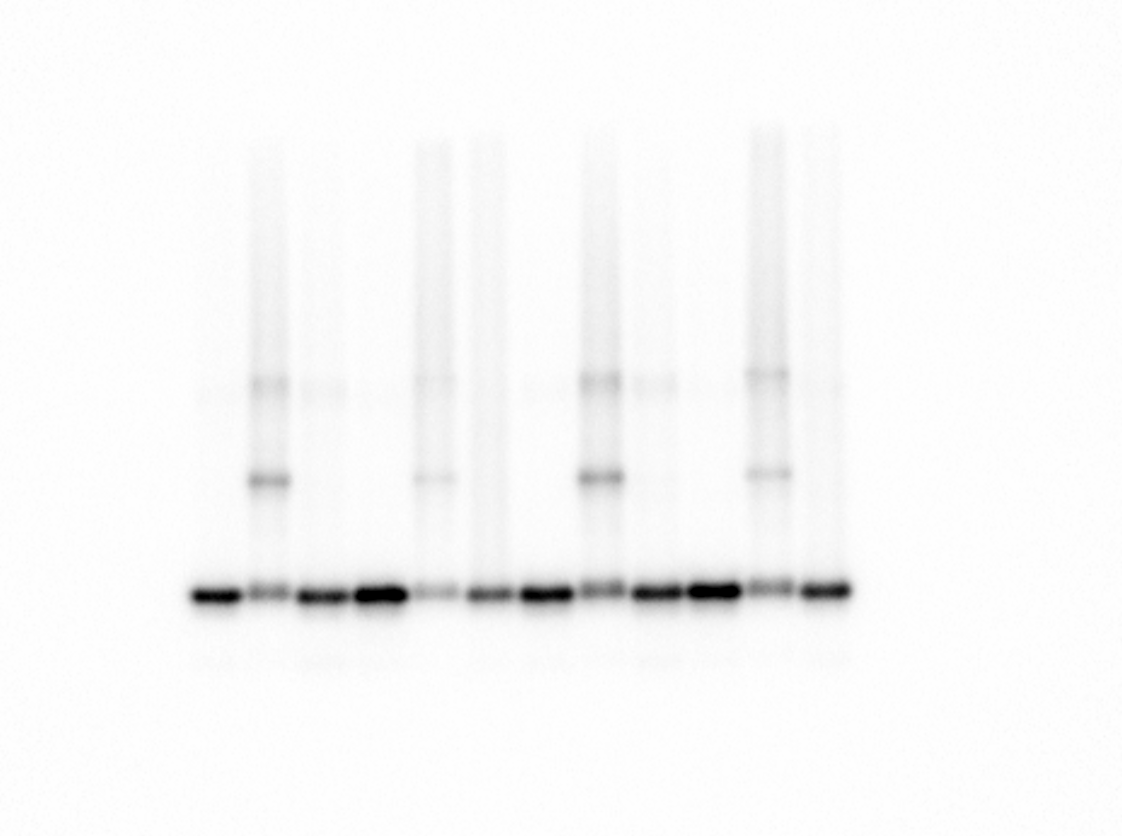

Supplement: Figure 5—figure supplement 3—source data 1. [file elife-74275-fig5-figsupp3-data1.zip › Figure 5-figure supplement 3-source data 1/figure supplement 3C/pSO127 and 133 anti-FLAG 15-30min Set1.tif]

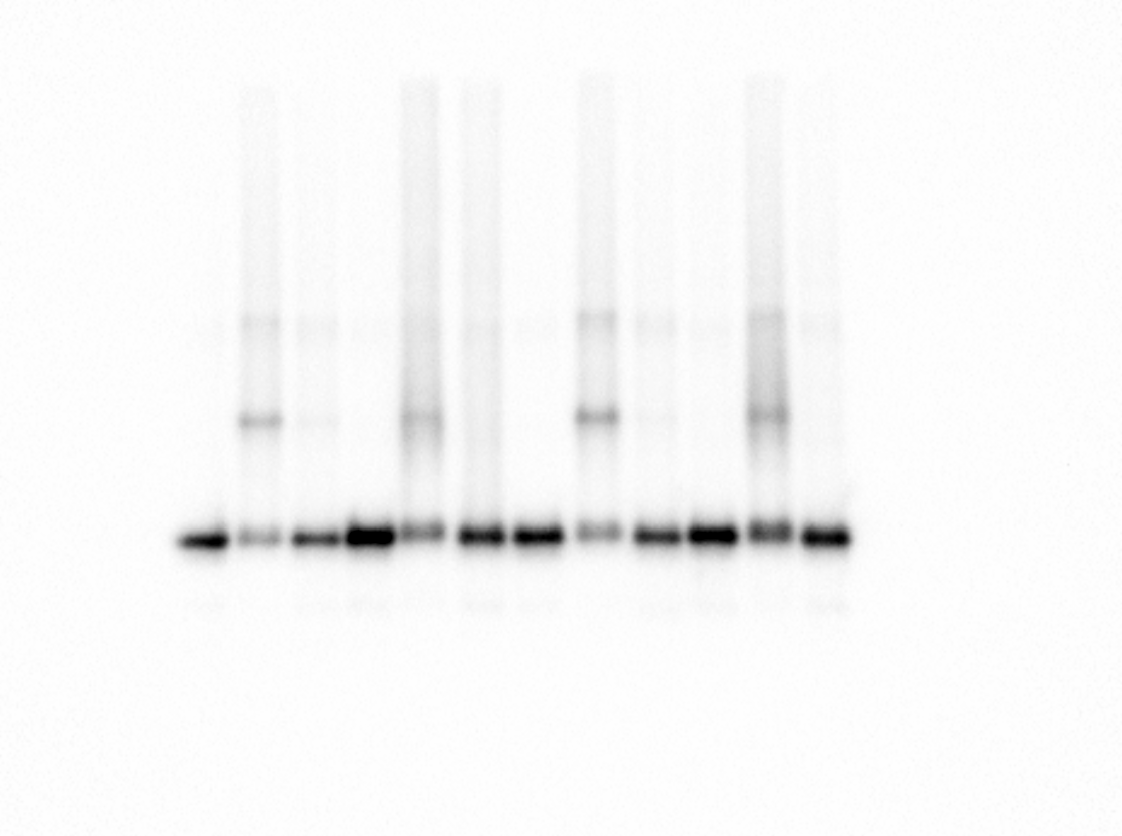

Supplement: Figure 5—figure supplement 3—source data 1. [file elife-74275-fig5-figsupp3-data1.zip › Figure 5-figure supplement 3-source data 1/figure supplement 3C/pSO127 and 133 anti-FLAG 15-30min Set2.tif]

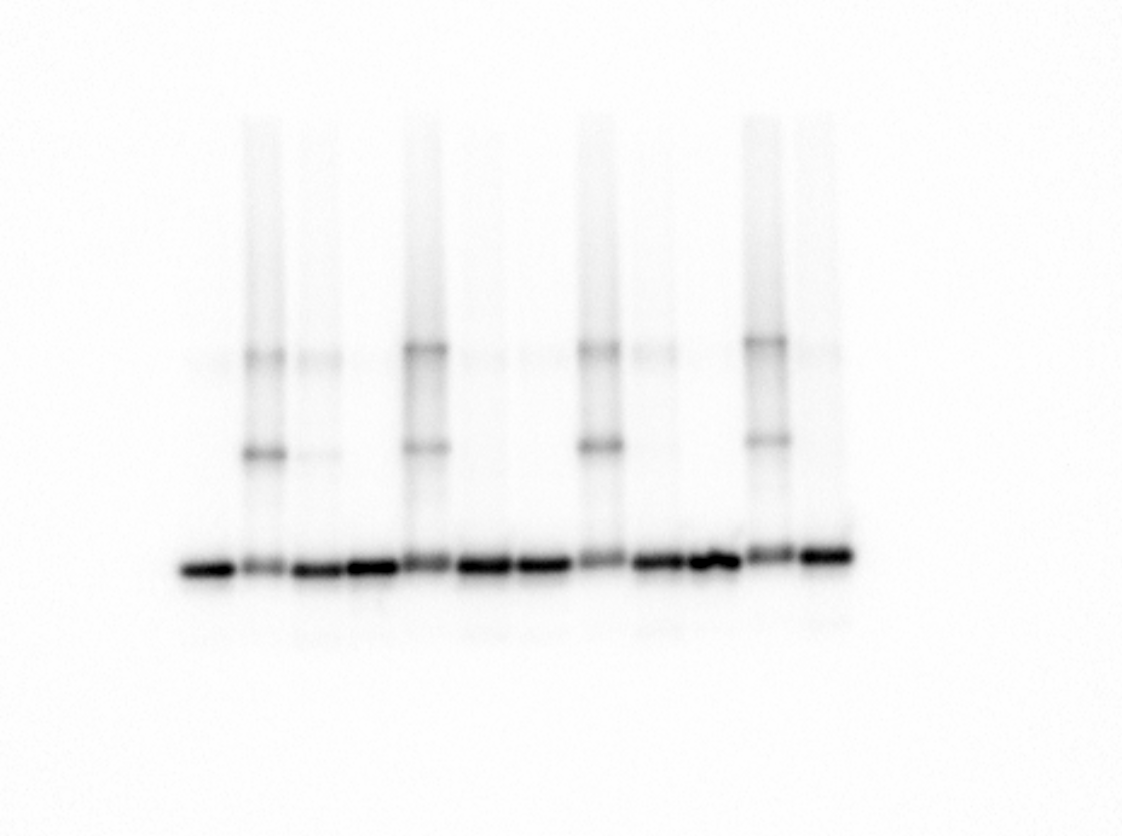

Supplement: Figure 5—figure supplement 3—source data 1. [file elife-74275-fig5-figsupp3-data1.zip › Figure 5-figure supplement 3-source data 1/figure supplement 3C/pSO127 and 133 anti-FLAG 45-60min Set1.tif]

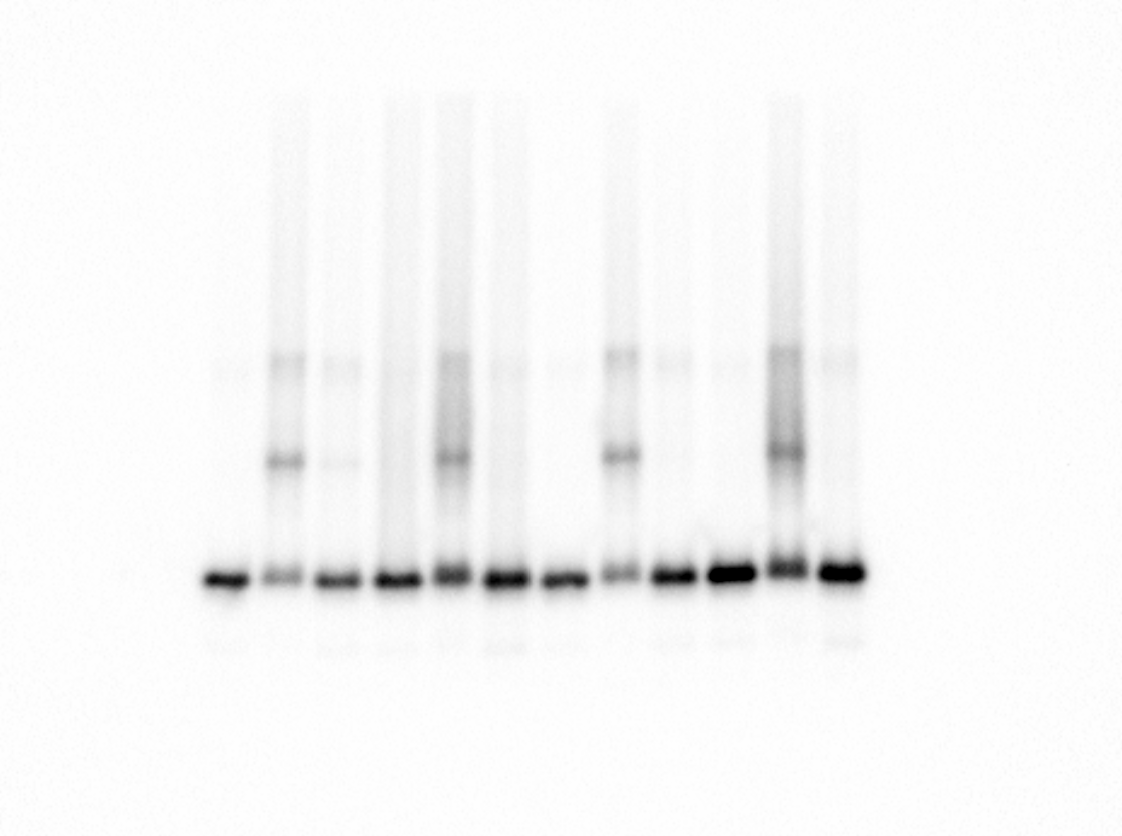

Supplement: Figure 5—figure supplement 3—source data 1. [file elife-74275-fig5-figsupp3-data1.zip › Figure 5-figure supplement 3-source data 1/figure supplement 3C/pSO127 and 133 anti-FLAG 45-60min Set2.tif]

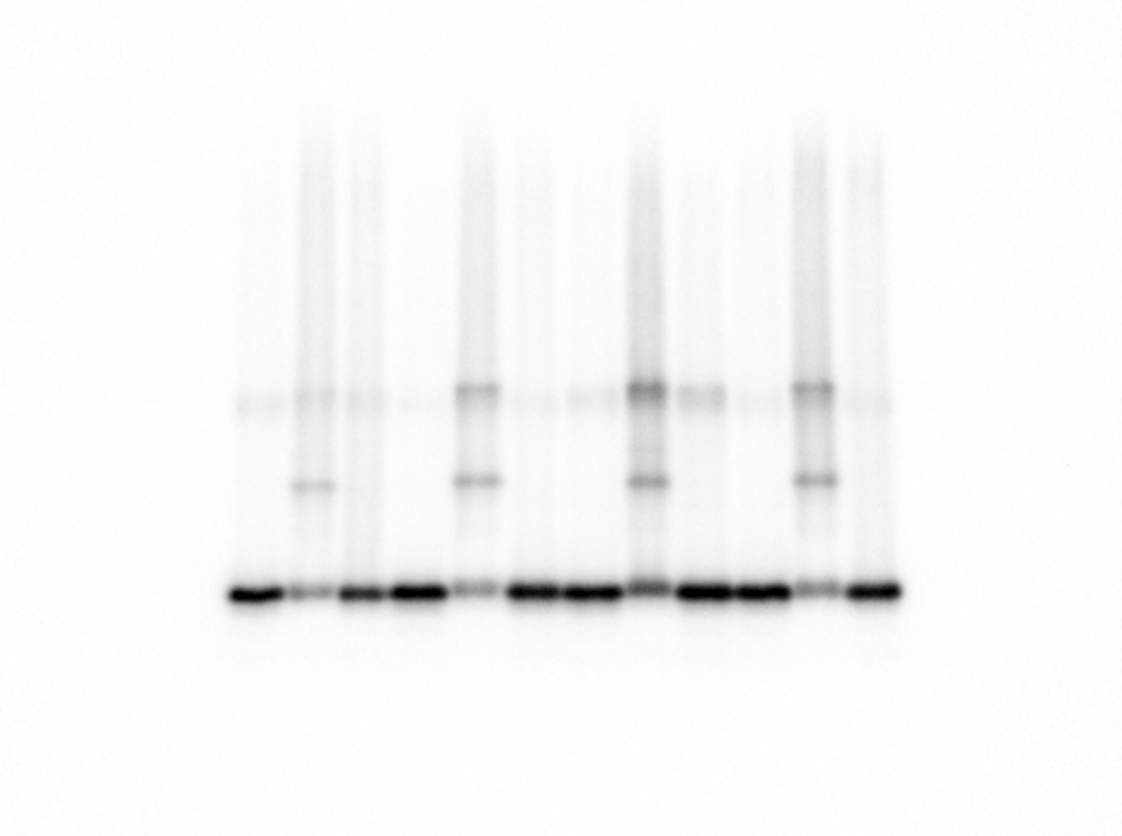

Supplement: Figure 5—figure supplement 3—source data 1. [file elife-74275-fig5-figsupp3-data1.zip › Figure 5-figure supplement 3-source data 1/figure supplement 3C/pSO245 and 246 anti-FLAG 15-30min Set1.tif]

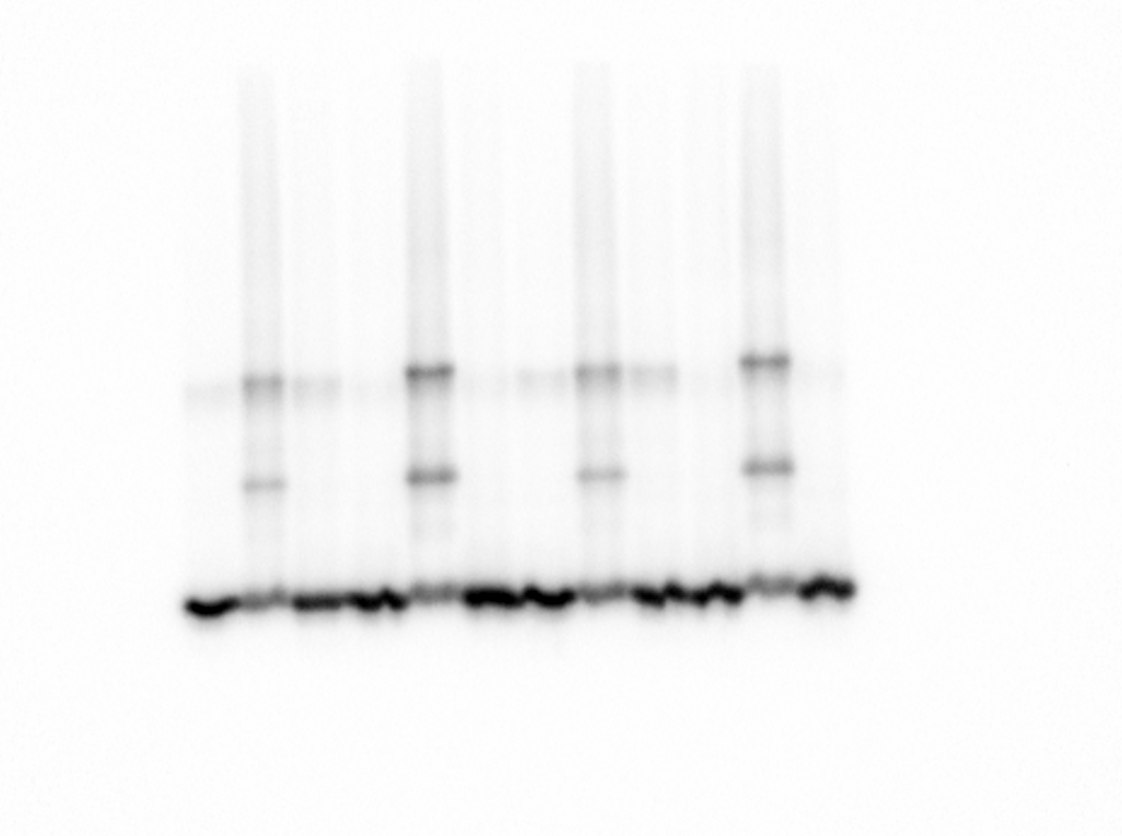

Supplement: Figure 5—figure supplement 3—source data 1. [file elife-74275-fig5-figsupp3-data1.zip › Figure 5-figure supplement 3-source data 1/figure supplement 3C/pSO245 and 246 anti-FLAG 15-30min Set2.tif]

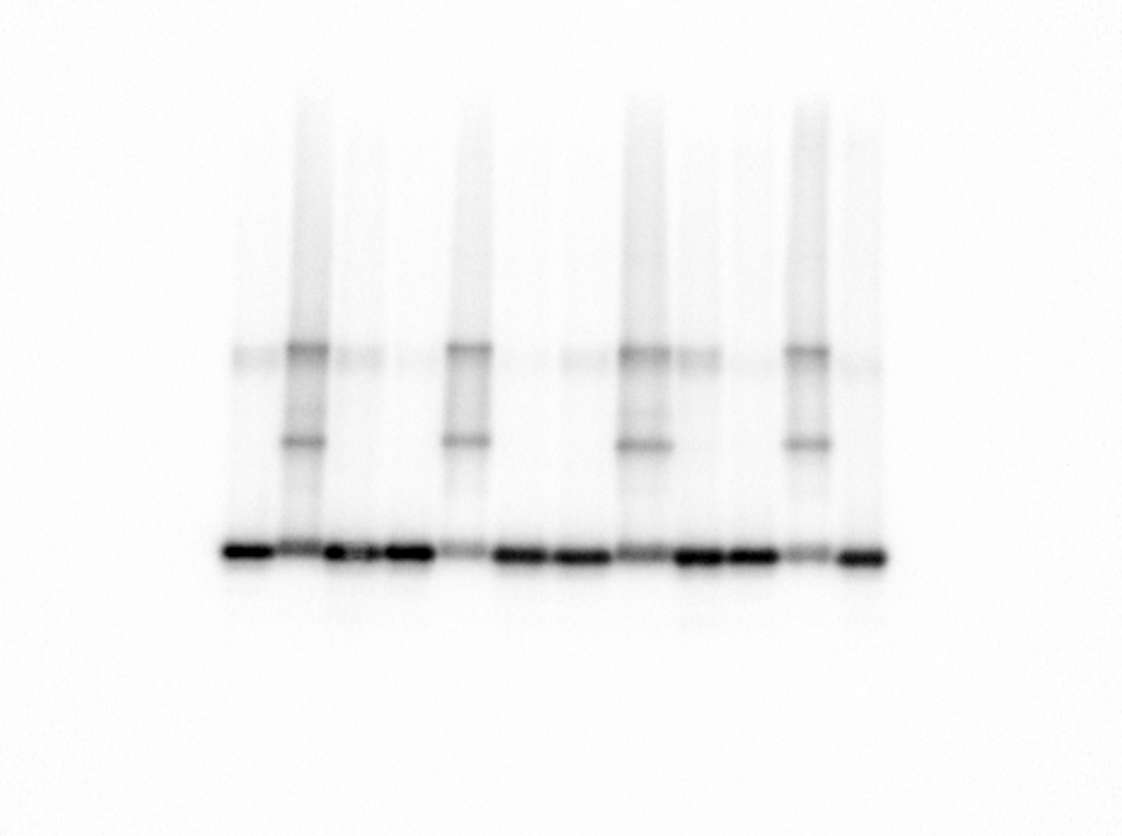

Supplement: Figure 5—figure supplement 3—source data 1. [file elife-74275-fig5-figsupp3-data1.zip › Figure 5-figure supplement 3-source data 1/figure supplement 3C/pSO245 and 246 anti-FLAG 45-60min Set1.tif]

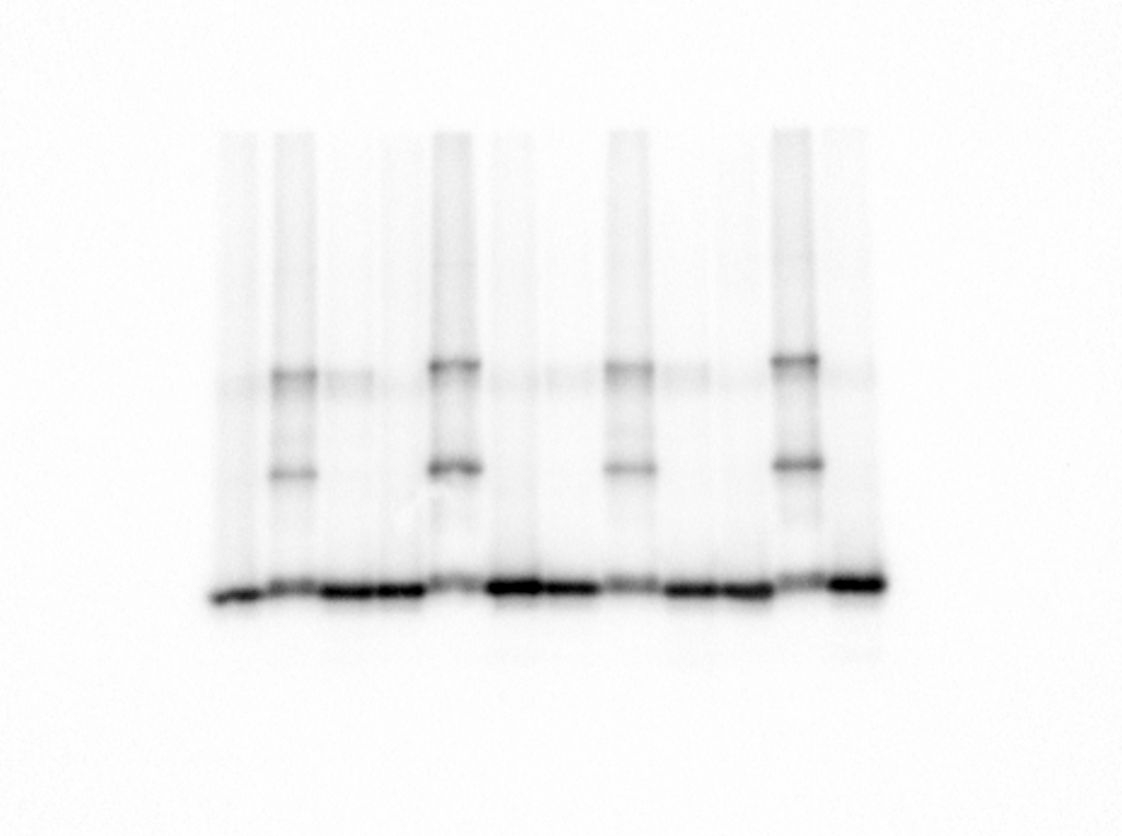

Supplement: Figure 5—figure supplement 3—source data 1. [file elife-74275-fig5-figsupp3-data1.zip › Figure 5-figure supplement 3-source data 1/figure supplement 3C/pSO245 and 246 anti-FLAG 45-60min Set2.tif]

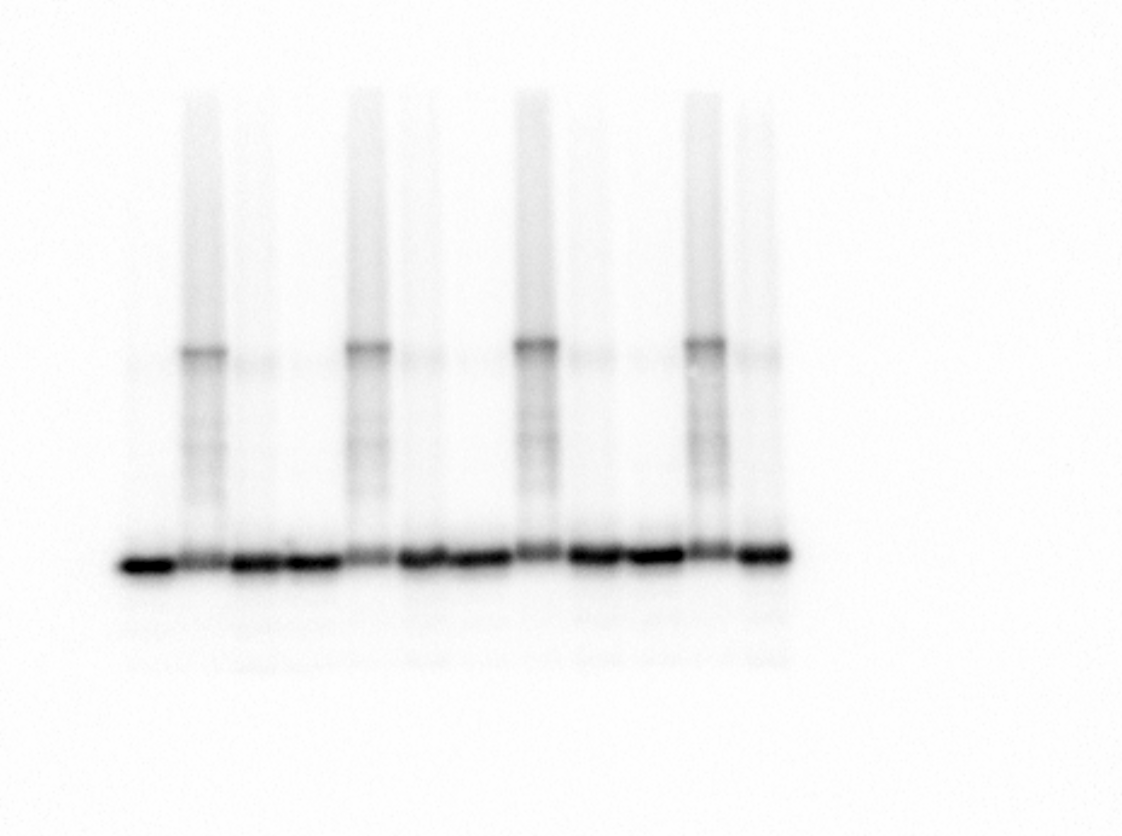

Supplement: Figure 5—figure supplement 3—source data 1. [file elife-74275-fig5-figsupp3-data1.zip › Figure 5-figure supplement 3-source data 1/figure supplement 3C/pSO255 anti-FLAG 15-60min Set1.tif]

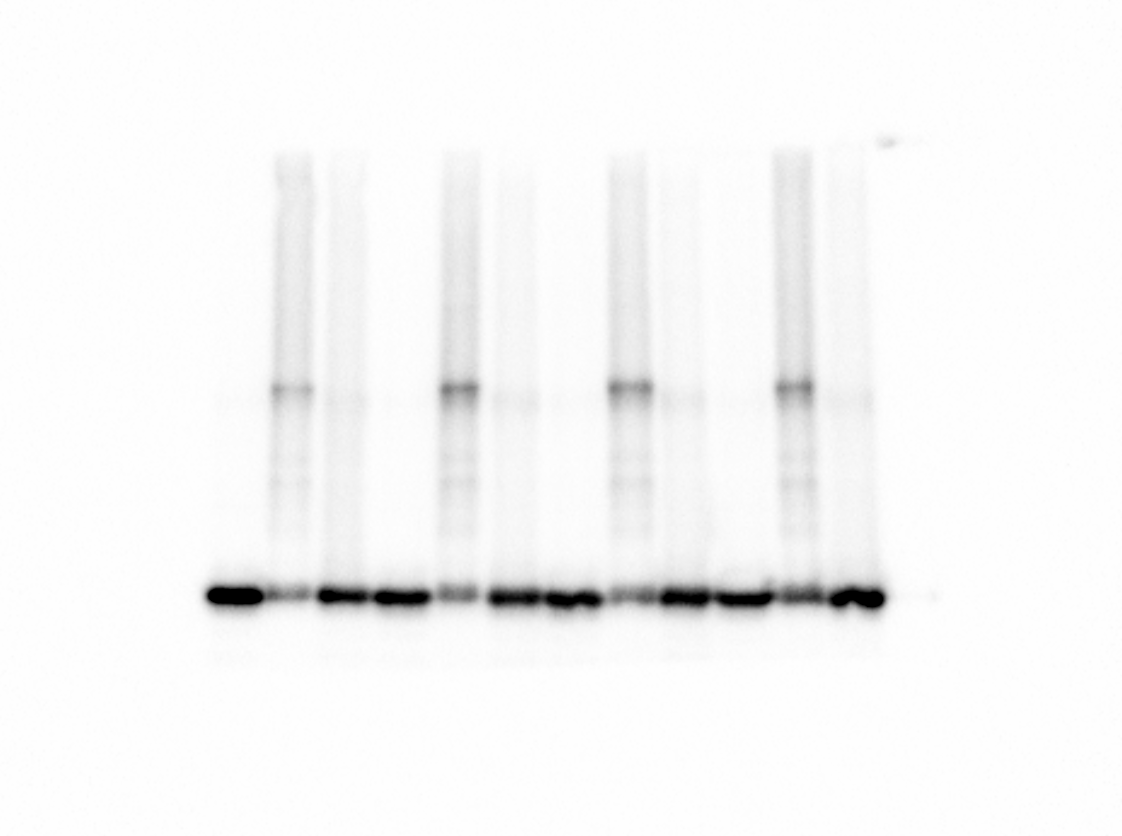

Supplement: Figure 5—figure supplement 3—source data 1. [file elife-74275-fig5-figsupp3-data1.zip › Figure 5-figure supplement 3-source data 1/figure supplement 3C/pSO255 anti-FLAG 15-60min Set2.tif]

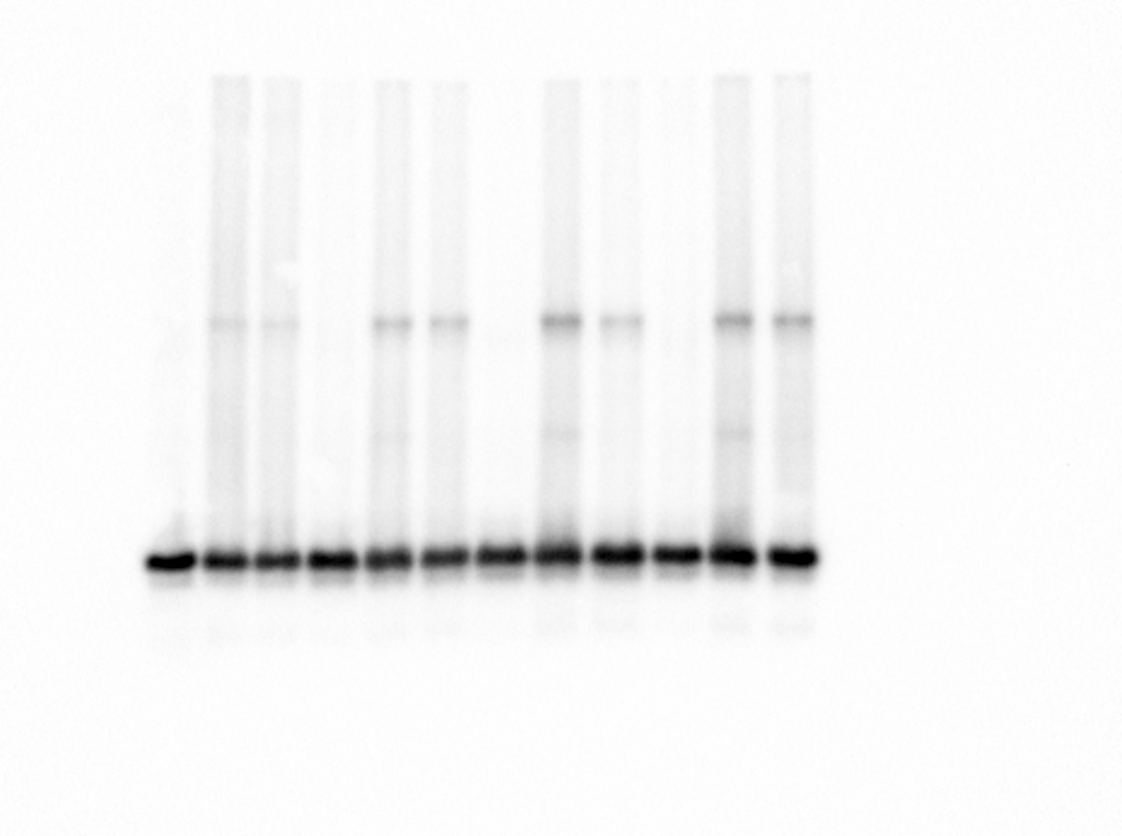

Supplement: Figure 6—figure supplement 1—source data 1. [file elife-74275-fig6-figsupp1-data1.zip › Figure 6-figure supplement 1-source data 1/figure supplement 1A/pSO167 anti-FLAG 15-60min Set1.tif]

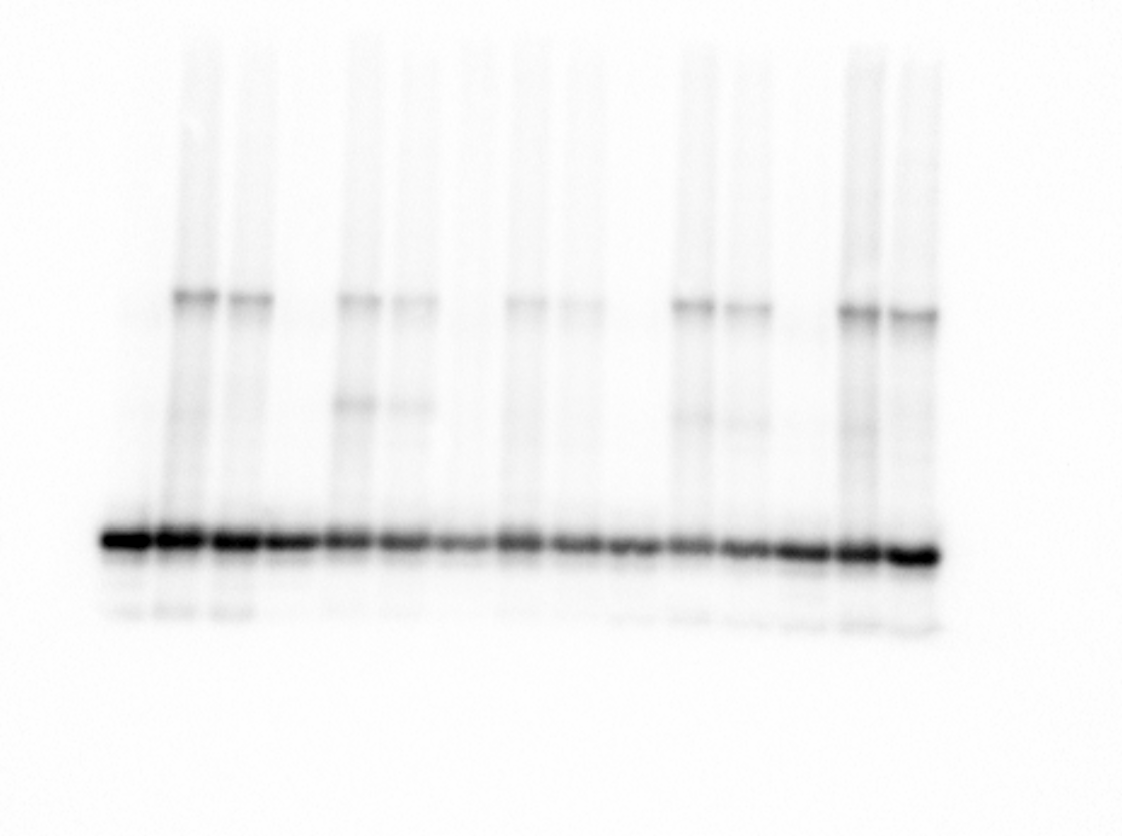

Supplement: Figure 6—figure supplement 1—source data 1. [file elife-74275-fig6-figsupp1-data1.zip › Figure 6-figure supplement 1-source data 1/figure supplement 1A/pSO167, 169, 170 and 128 15min and 167 30min anti-FLAG Set2.tif]
