# Supplementary material for: Inhibitory proteins block substrate access by occupying the active site cleft of Bacillus subtilis intramembrane protease SpoIVFB
Source: eLife. 2022 Apr 26;11:e74275. doi: 10.7554/eLife.74275 (PMC9042235; doi:10.7554/eLife.74275)
Supplement: Figure 3—figure supplement 1—source data 1. [file elife-74275-fig3-figsupp1-data1.zip › Figure 3-figure supplement 1-source data 1/fig sup 1 annotated blots.pptx]

## Slide 1
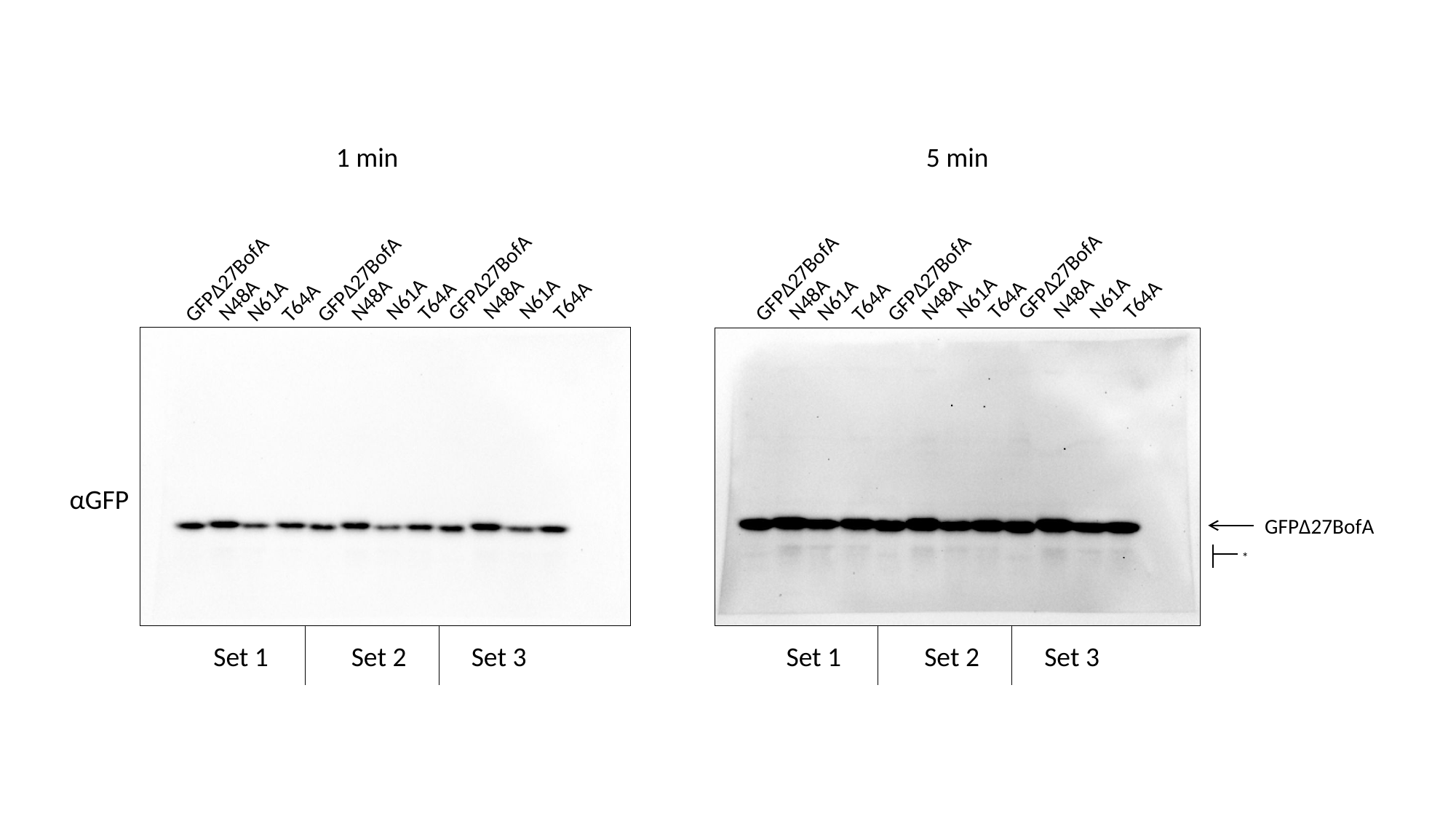

1 min
5 min
GFPΔ27BofA
GFPΔ27BofA
GFPΔ27BofA
GFPΔ27BofA
GFPΔ27BofA
GFPΔ27BofA
N61A
N48A
N61A
N61A
N48A
N61A
N48A
N48A
T64A
T64A
N61A
N48A
N48A
T64A
T64A
N61A
T64A
T64A
αGFP
GFPΔ27BofA
*
Set 1
Set 2
Set 3
Set 1
Set 2
Set 3
