# Supplementary material for: Inhibitory proteins block substrate access by occupying the active site cleft of Bacillus subtilis intramembrane protease SpoIVFB
Source: eLife. 2022 Apr 26;11:e74275. doi: 10.7554/eLife.74275 (PMC9042235; doi:10.7554/eLife.74275)
Supplement: Figure 4—source data 1. [file elife-74275-fig4-data1.zip › Figure 4-source data 1/Fig 4 annotated blots.pptx]

## Slide 1
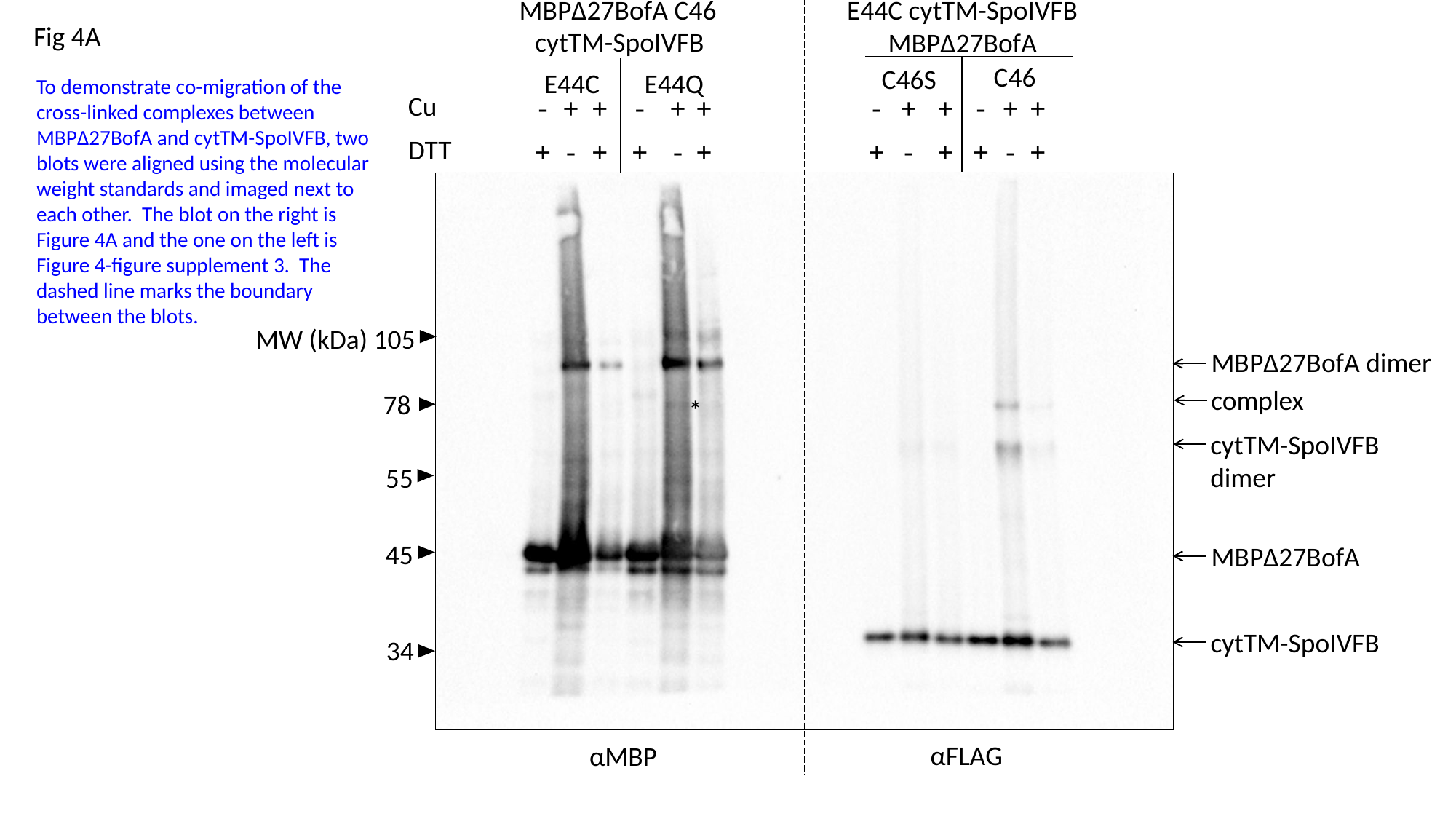

E44C cytTM-SpoIVFB
MBPΔ27BofA
MBPΔ27BofA C46
Fig 4A
cytTM-SpoIVFB
C46
C46S
E44C
E44Q
To demonstrate co-migration of the cross-linked complexes between MBPΔ27BofA and cytTM-SpoIVFB, two blots were aligned using the molecular weight standards and imaged next to each other. The blot on the right is Figure 4A and the one on the left is Figure 4-figure supplement 3. The dashed line marks the boundary between the blots.
| Cu | - | + | + | - | + | + | - | + | + | - | + | + | |
| --- | --- | --- | --- | --- | --- | --- | --- | --- | --- | --- | --- | --- | --- |
| DTT | + | - | + | + | - | + | + | - | + | + | - | + | |
MW (kDa) 105
MBPΔ27BofA dimer
complex
78
*
cytTM-SpoIVFB
dimer
55
45
MBPΔ27BofA
cytTM-SpoIVFB
34
αFLAG
αMBP

## Slide 2
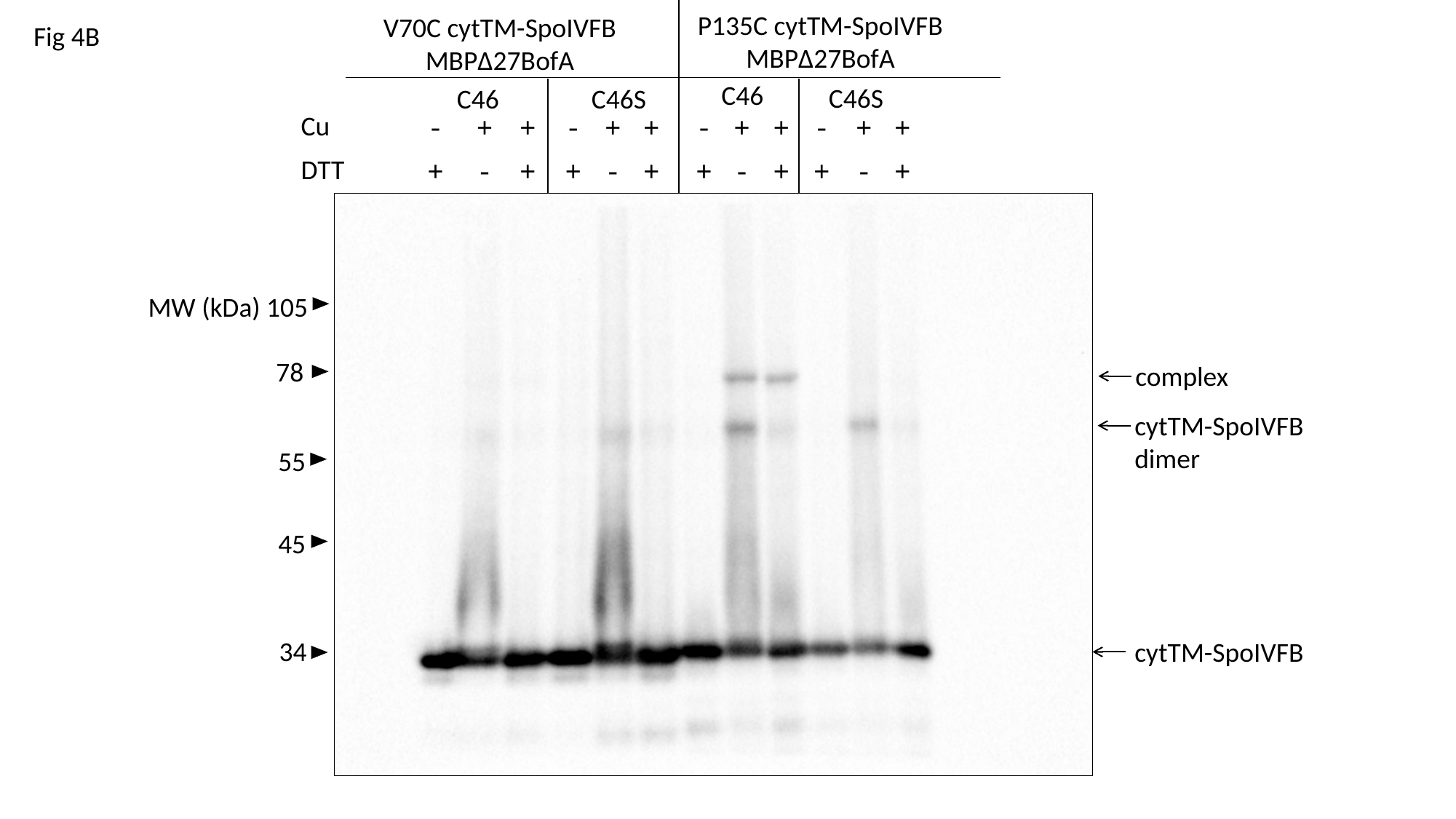

P135C cytTM-SpoIVFB
MBPΔ27BofA
V70C cytTM-SpoIVFB
MBPΔ27BofA
Fig 4B
C46
C46S
C46S
C46
| Cu | - | + | + | - | + | + | - | + | + | - | + | + | |
| --- | --- | --- | --- | --- | --- | --- | --- | --- | --- | --- | --- | --- | --- |
| DTT | + | - | + | + | - | + | + | - | + | + | - | + | |
MW (kDa) 105
78
complex
cytTM-SpoIVFB
dimer
55
45
34
cytTM-SpoIVFB
