# Supplementary material for: Inhibitory proteins block substrate access by occupying the active site cleft of Bacillus subtilis intramembrane protease SpoIVFB
Source: eLife. 2022 Apr 26;11:e74275. doi: 10.7554/eLife.74275 (PMC9042235; doi:10.7554/eLife.74275)
Supplement: Figure 4—figure supplement 1—source data 1. [file elife-74275-fig4-figsupp1-data1.zip › Figure 4-figure supplement 1-source data 1/readme.docx]

The PyMOL session file (fig sup 1) was derived from a model of a SpoIVFB tetramer in complex with two Pro-σ^K^ monomers reported previously (1). Only SpoIVFB chain A is shown in Figure 4-figure supplement 1A. In Figure 4-figure supplement 1B, predicted TMS2 of BofA is also shown, in an orientation to satisfy experimentally-observed cross-links (Figure 4, Figure 4-figure supplement 5, and Supplementary File 1).

Reference

1. Halder S, Parrell D, Whitten D, Feig M, & Kroos L (2017) Interaction of intramembrane metalloprotease SpoIVFB with substrate Pro-σ^K^. *Proc. Natl. Acad. Sci. USA* 114:E10677-E10686.
