# Supplementary material for: Inhibitory proteins block substrate access by occupying the active site cleft of Bacillus subtilis intramembrane protease SpoIVFB
Source: eLife. 2022 Apr 26;11:e74275. doi: 10.7554/eLife.74275 (PMC9042235; doi:10.7554/eLife.74275)
Supplement: Figure 4—figure supplement 2—source data 1. [file elife-74275-fig4-figsupp2-data1.zip › Figure 4-figure supplement 2-source data 1/fig sup 2 annotated blots.pptx]

## Slide 1
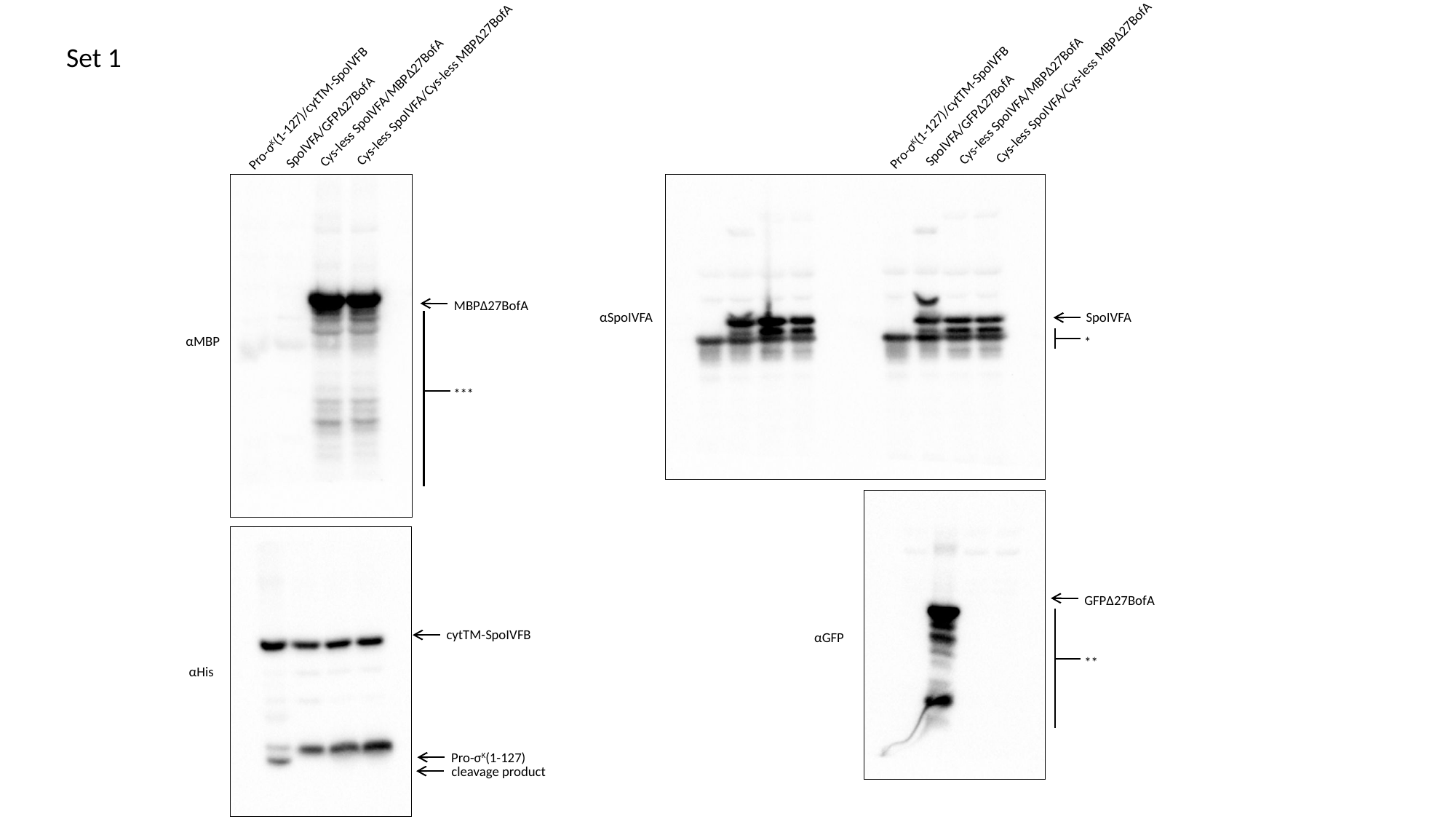

Set 1
Cys-less SpoIVFA/Cys-less MBPΔ27BofA
Cys-less SpoIVFA/Cys-less MBPΔ27BofA
Cys-less SpoIVFA/MBPΔ27BofA
Cys-less SpoIVFA/MBPΔ27BofA
Pro-σK(1-127)/cytTM-SpoIVFB
Pro-σK(1-127)/cytTM-SpoIVFB
SpoIVFA/GFPΔ27BofA
SpoIVFA/GFPΔ27BofA
MBPΔ27BofA
αSpoIVFA
SpoIVFA
αMBP
*
***
GFPΔ27BofA
cytTM-SpoIVFB
αGFP
**
αHis
Pro-σK(1-127)
cleavage product

## Slide 2
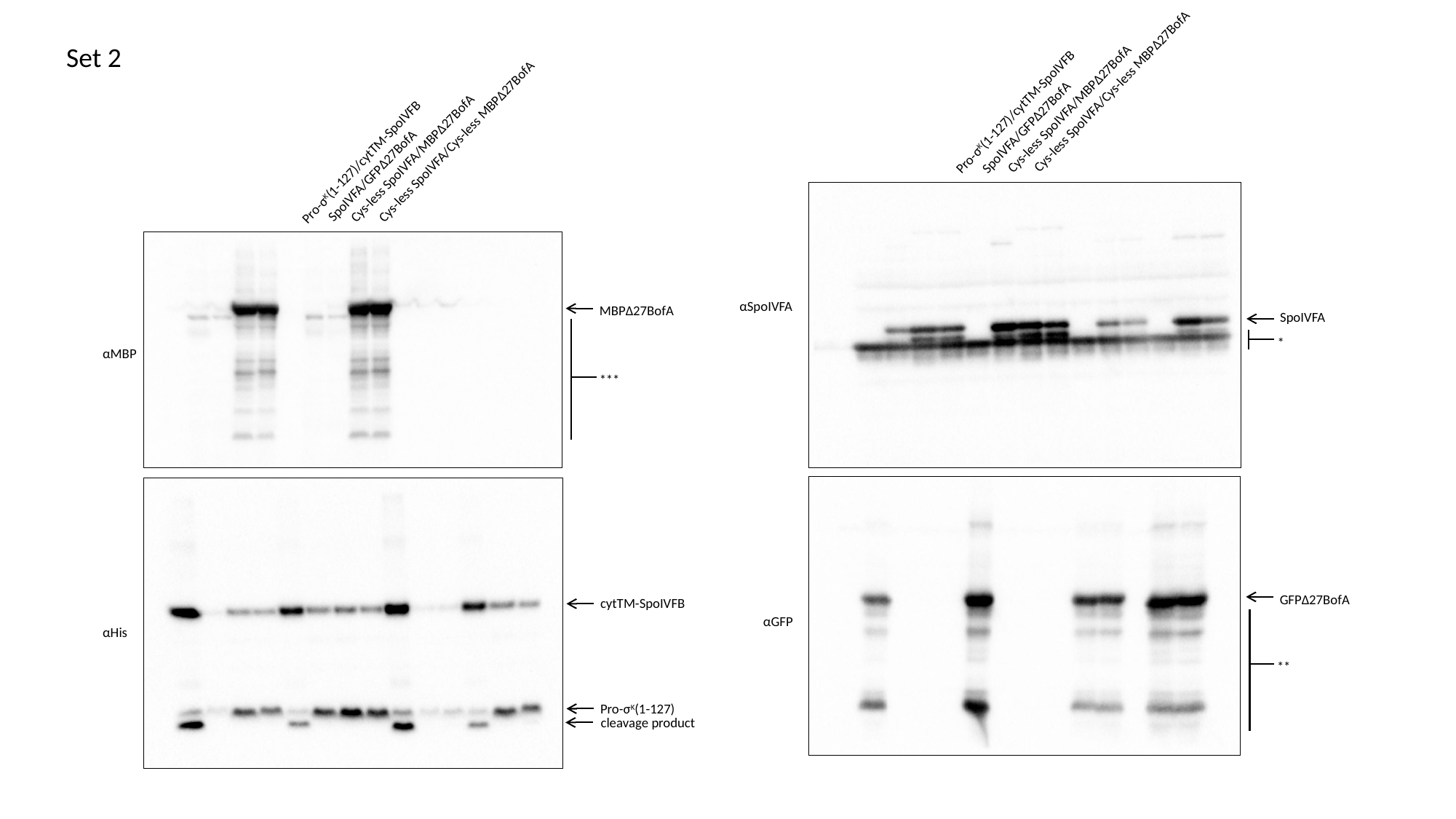

Set 2
Cys-less SpoIVFA/Cys-less MBPΔ27BofA
Cys-less SpoIVFA/MBPΔ27BofA
Pro-σK(1-127)/cytTM-SpoIVFB
SpoIVFA/GFPΔ27BofA
Cys-less SpoIVFA/Cys-less MBPΔ27BofA
Cys-less SpoIVFA/MBPΔ27BofA
Pro-σK(1-127)/cytTM-SpoIVFB
SpoIVFA/GFPΔ27BofA
αSpoIVFA
MBPΔ27BofA
SpoIVFA
*
αMBP
***
GFPΔ27BofA
cytTM-SpoIVFB
αGFP
αHis
**
Pro-σK(1-127)
cleavage product

## Slide 3
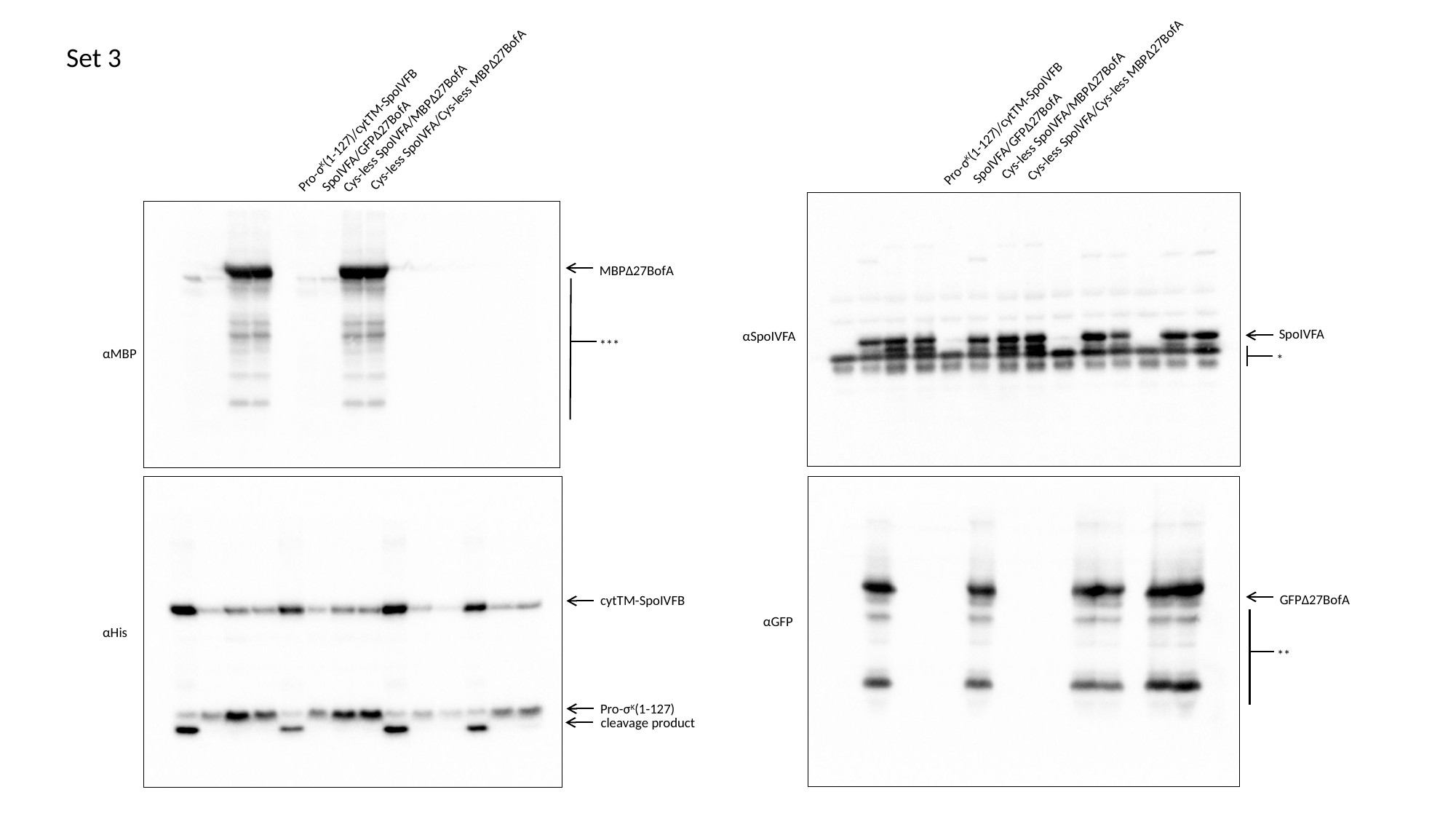

Set 3
Cys-less SpoIVFA/Cys-less MBPΔ27BofA
Cys-less SpoIVFA/Cys-less MBPΔ27BofA
Cys-less SpoIVFA/MBPΔ27BofA
Pro-σK(1-127)/cytTM-SpoIVFB
Cys-less SpoIVFA/MBPΔ27BofA
Pro-σK(1-127)/cytTM-SpoIVFB
SpoIVFA/GFPΔ27BofA
SpoIVFA/GFPΔ27BofA
MBPΔ27BofA
SpoIVFA
αSpoIVFA
***
αMBP
*
GFPΔ27BofA
cytTM-SpoIVFB
αGFP
αHis
**
Pro-σK(1-127)
cleavage product
