# Supplementary material for: Inhibitory proteins block substrate access by occupying the active site cleft of Bacillus subtilis intramembrane protease SpoIVFB
Source: eLife. 2022 Apr 26;11:e74275. doi: 10.7554/eLife.74275 (PMC9042235; doi:10.7554/eLife.74275)
Supplement: Figure 4—figure supplement 4—source data 1. [file elife-74275-fig4-figsupp4-data1.zip › Figure 4-figure supplement 4-source data 1/fig sup 4 annotated blots.pptx]

## Slide 1
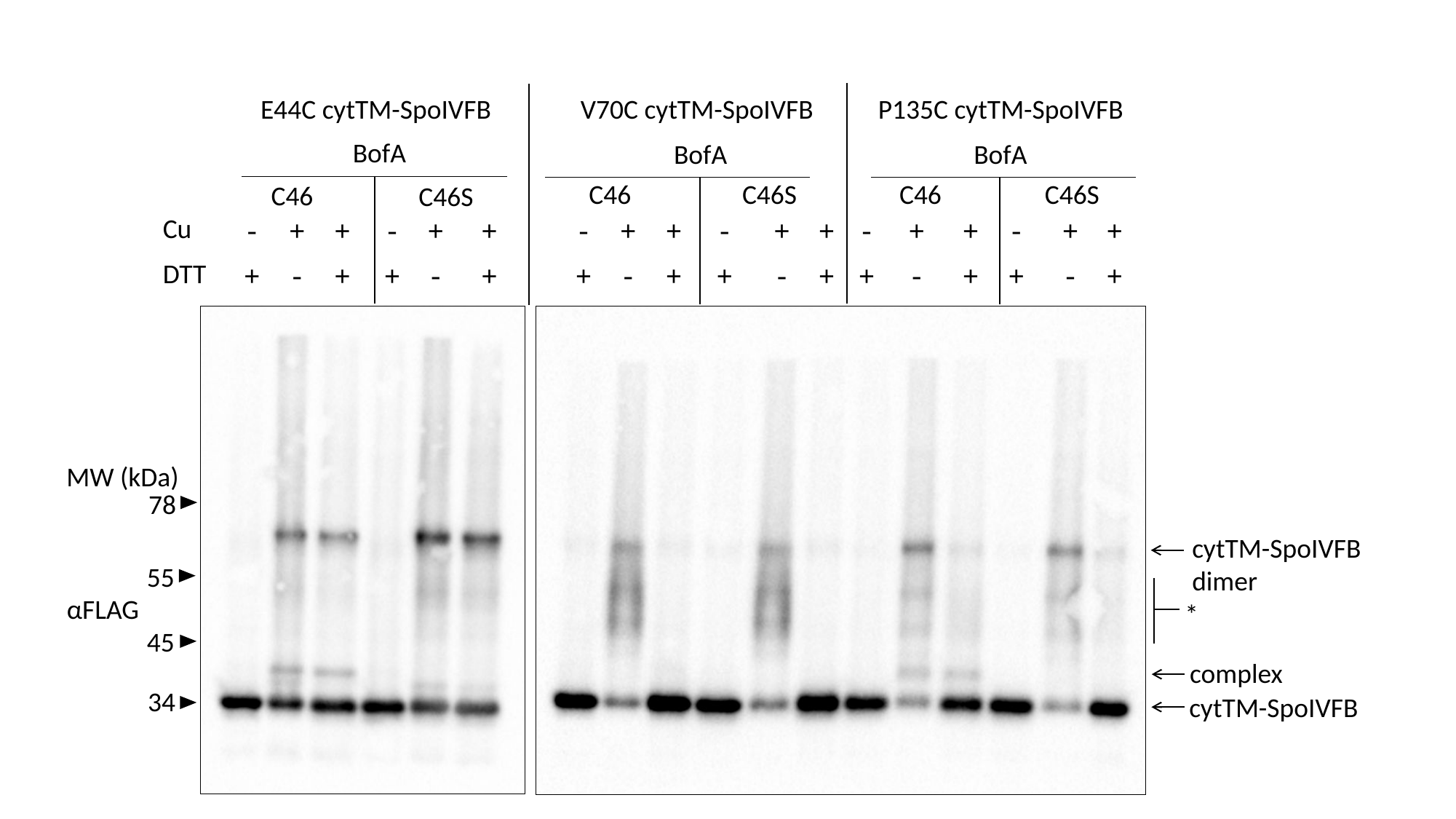

E44C cytTM-SpoIVFB
V70C cytTM-SpoIVFB
P135C cytTM-SpoIVFB
BofA
BofA
 BofA
C46
C46
C46S
C46S
C46
C46S
| Cu | - | + | + | - | + | + | - | + | + | - | + | + | - | + | + | - | + | + |
| --- | --- | --- | --- | --- | --- | --- | --- | --- | --- | --- | --- | --- | --- | --- | --- | --- | --- | --- |
| DTT | + | - | + | + | - | + | + | - | + | + | - | + | + | - | + | + | - | + |
MW (kDa)
78
cytTM-SpoIVFB
dimer
55
αFLAG
*
45
complex
34
 cytTM-SpoIVFB
