# Supplementary material for: Inhibitory proteins block substrate access by occupying the active site cleft of Bacillus subtilis intramembrane protease SpoIVFB
Source: eLife. 2022 Apr 26;11:e74275. doi: 10.7554/eLife.74275 (PMC9042235; doi:10.7554/eLife.74275)
Supplement: Figure 4—figure supplement 5—source data 1. [file elife-74275-fig4-figsupp5-data1.zip › Figure 4-figure supplement 5-source data 1/figure supplement 5B/fig sup 5B annotated blots.pptx]

## Slide 1
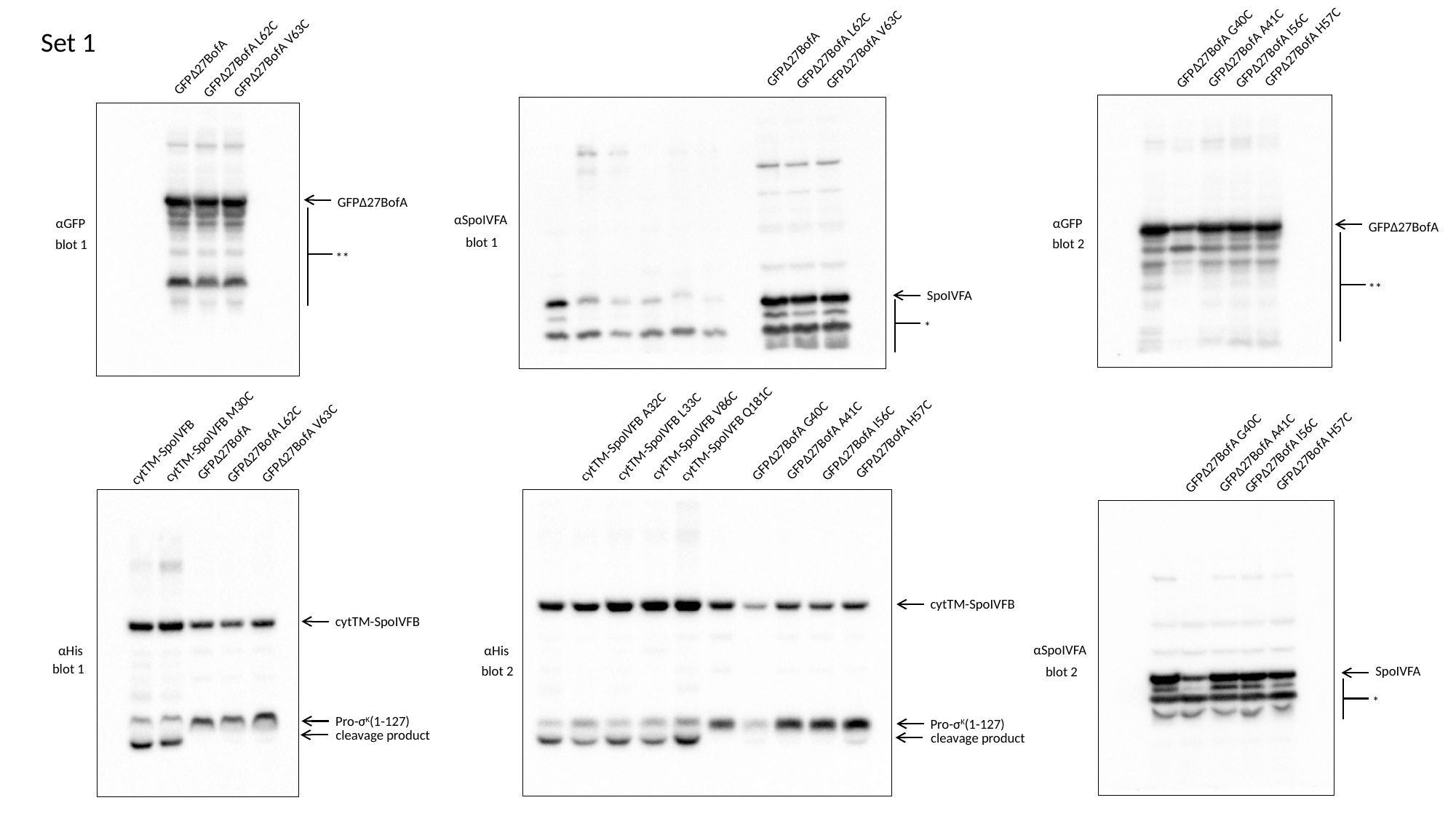

Set 1
GFPΔ27BofA H57C
GFPΔ27BofA A41C
GFPΔ27BofA G40C
GFPΔ27BofA V63C
GFPΔ27BofA L62C
GFPΔ27BofA I56C
GFPΔ27BofA V63C
GFPΔ27BofA
GFPΔ27BofA L62C
GFPΔ27BofA
GFPΔ27BofA
αSpoIVFA
αGFP
αGFP
GFPΔ27BofA
blot 1
blot 2
blot 1
**
**
SpoIVFA
*
cytTM-SpoIVFB Q181C
cytTM-SpoIVFB V86C
cytTM-SpoIVFB M30C
cytTM-SpoIVFB A32C
cytTM-SpoIVFB L33C
GFPΔ27BofA H57C
GFPΔ27BofA A41C
GFPΔ27BofA G40C
GFPΔ27BofA I56C
GFPΔ27BofA V63C
GFPΔ27BofA L62C
GFPΔ27BofA H57C
cytTM-SpoIVFB
GFPΔ27BofA
GFPΔ27BofA A41C
GFPΔ27BofA G40C
GFPΔ27BofA I56C
cytTM-SpoIVFB
cytTM-SpoIVFB
αSpoIVFA
αHis
αHis
blot 1
blot 2
SpoIVFA
blot 2
*
Pro-σK(1-127)
Pro-σK(1-127)
cleavage product
cleavage product

## Slide 2
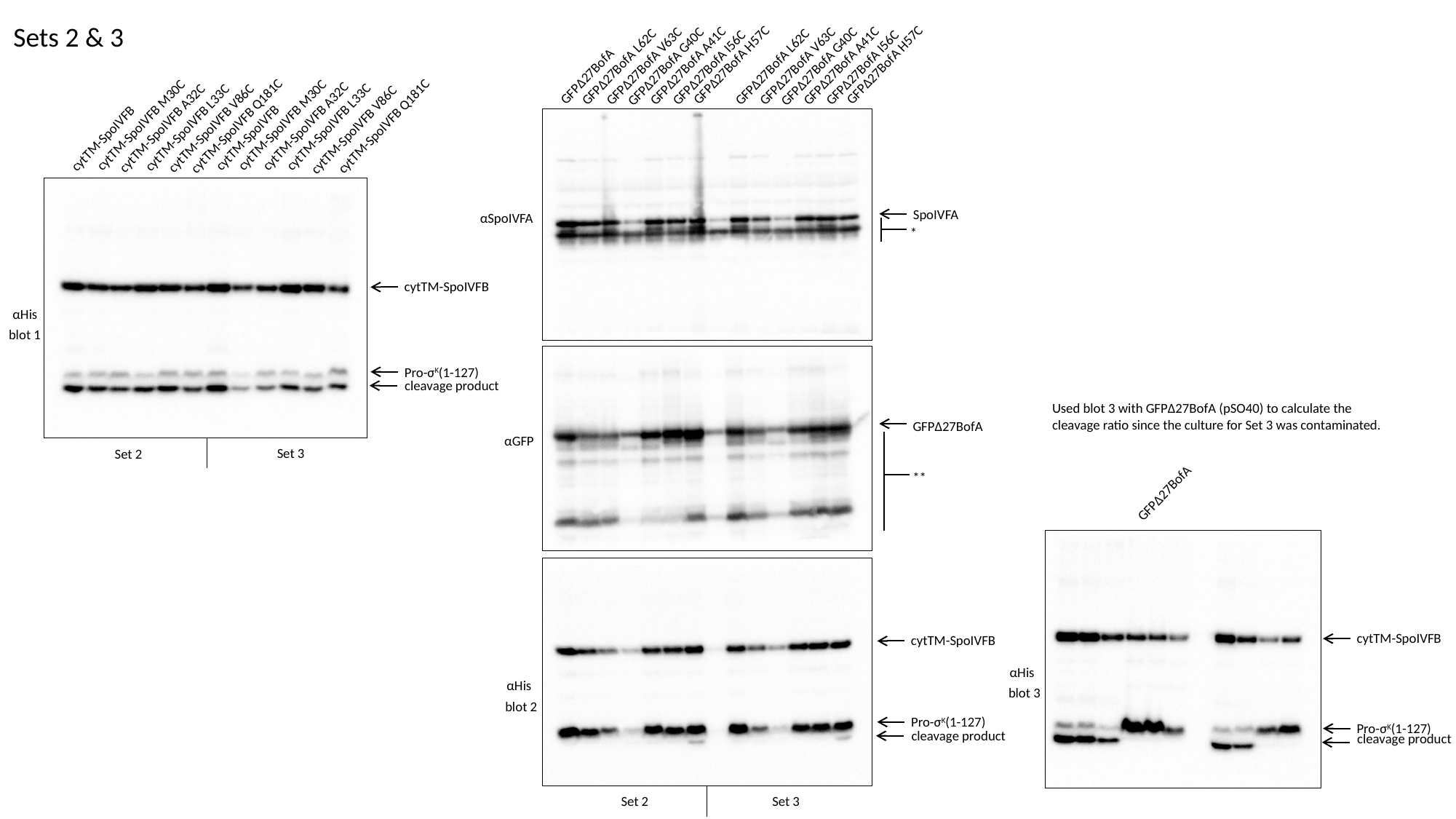

Sets 2 & 3
GFPΔ27BofA H57C
GFPΔ27BofA H57C
GFPΔ27BofA A41C
GFPΔ27BofA A41C
GFPΔ27BofA V63C
GFPΔ27BofA V63C
GFPΔ27BofA G40C
GFPΔ27BofA G40C
GFPΔ27BofA L62C
GFPΔ27BofA L62C
GFPΔ27BofA I56C
GFPΔ27BofA I56C
GFPΔ27BofA
cytTM-SpoIVFB M30C
cytTM-SpoIVFB M30C
cytTM-SpoIVFB Q181C
cytTM-SpoIVFB Q181C
cytTM-SpoIVFB A32C
cytTM-SpoIVFB L33C
cytTM-SpoIVFB L33C
cytTM-SpoIVFB V86C
cytTM-SpoIVFB A32C
cytTM-SpoIVFB V86C
cytTM-SpoIVFB
cytTM-SpoIVFB
SpoIVFA
αSpoIVFA
*
cytTM-SpoIVFB
αHis
blot 1
Pro-σK(1-127)
cleavage product
Used blot 3 with GFPΔ27BofA (pSO40) to calculate the cleavage ratio since the culture for Set 3 was contaminated.
GFPΔ27BofA
αGFP
Set 3
Set 2
**
GFPΔ27BofA
cytTM-SpoIVFB
cytTM-SpoIVFB
αHis
αHis
blot 3
blot 2
Pro-σK(1-127)
Pro-σK(1-127)
cleavage product
cleavage product
Set 3
Set 2
