# Supplementary material for: Inhibitory proteins block substrate access by occupying the active site cleft of Bacillus subtilis intramembrane protease SpoIVFB
Source: eLife. 2022 Apr 26;11:e74275. doi: 10.7554/eLife.74275 (PMC9042235; doi:10.7554/eLife.74275)
Supplement: Figure 4—figure supplement 5—source data 1. [file elife-74275-fig4-figsupp5-data1.zip › Figure 4-figure supplement 5-source data 1/figure supplement 5C/fig sup 5C annotated blots.pptx]

## Slide 1
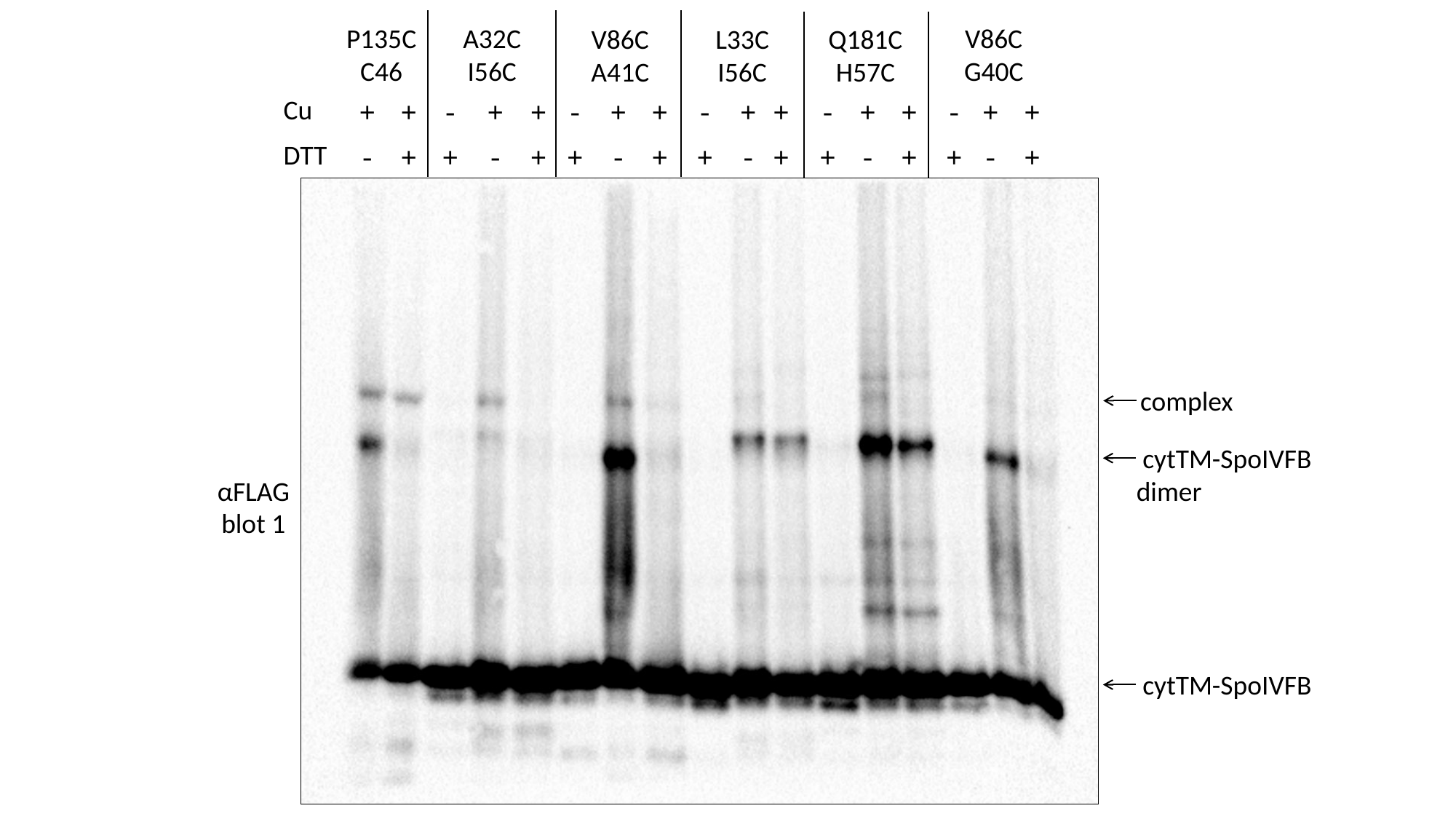

V86C
G40C
A32C
I56C
P135C
C46
V86C
A41C
L33C
I56C
Q181C
H57C
| Cu | + | + | - | + | + | - | + | + | - | + | + | - | + | + | - | + | + | |
| --- | --- | --- | --- | --- | --- | --- | --- | --- | --- | --- | --- | --- | --- | --- | --- | --- | --- | --- |
| DTT | - | + | + | - | + | + | - | + | + | - | + | + | - | + | + | - | + | |
complex
 cytTM-SpoIVFB
dimer
αFLAG
blot 1
 cytTM-SpoIVFB

## Slide 2
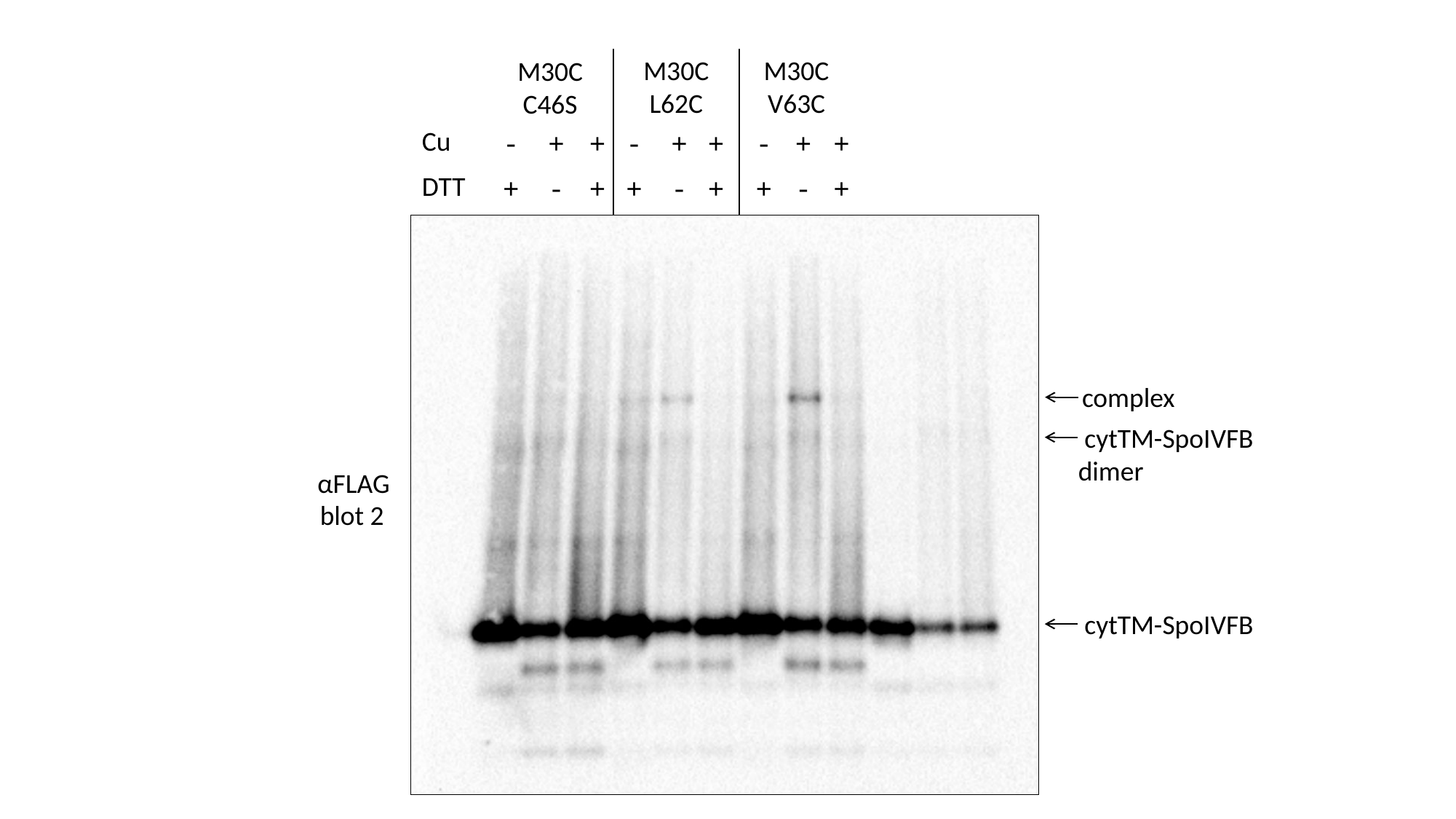

M30C
L62C
M30C
V63C
M30C
C46S
| Cu | - | + | + | - | + | + | - | + | + | | | | | | | | | |
| --- | --- | --- | --- | --- | --- | --- | --- | --- | --- | --- | --- | --- | --- | --- | --- | --- | --- | --- |
| DTT | + | - | + | + | - | + | + | - | + | | | | | | | | | |
complex
 cytTM-SpoIVFB
dimer
αFLAG
blot 2
 cytTM-SpoIVFB
