# Supplementary material for: Inhibitory proteins block substrate access by occupying the active site cleft of Bacillus subtilis intramembrane protease SpoIVFB
Source: eLife. 2022 Apr 26;11:e74275. doi: 10.7554/eLife.74275 (PMC9042235; doi:10.7554/eLife.74275)
Supplement: Figure 5—source data 1. [file elife-74275-fig5-data1.zip › Figure 5-source data 1/Figure 5A/Fig5A annotated blots.pptx]

## Slide 1
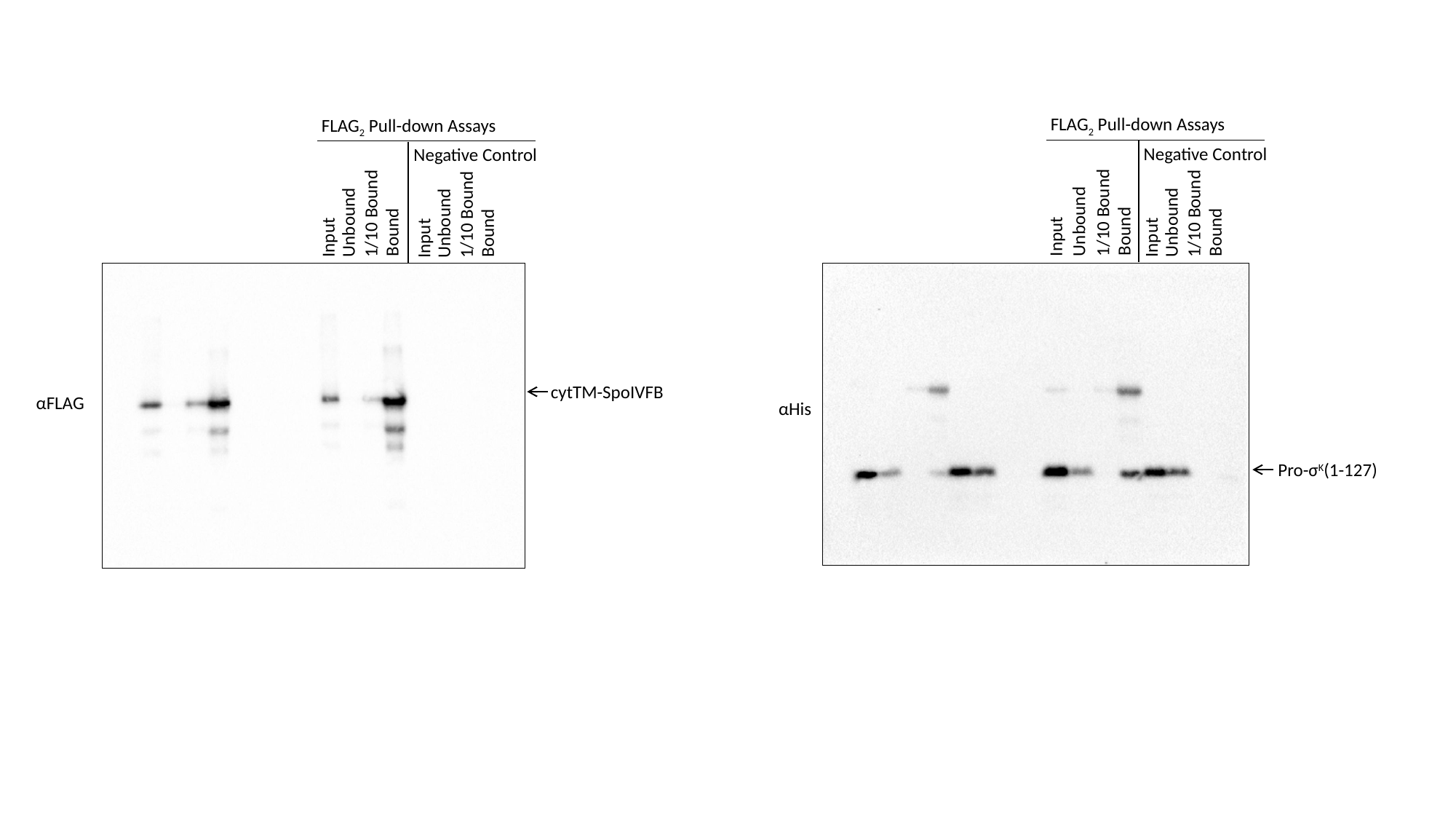

FLAG2 Pull-down Assays
FLAG2 Pull-down Assays
Negative Control
Negative Control
1/10 Bound
1/10 Bound
1/10 Bound
1/10 Bound
Unbound
Unbound
Unbound
Unbound
Bound
Bound
Bound
Bound
Input
Input
Input
Input
cytTM-SpoIVFB
αFLAG
αHis
Pro-σK(1-127)

## Slide 2
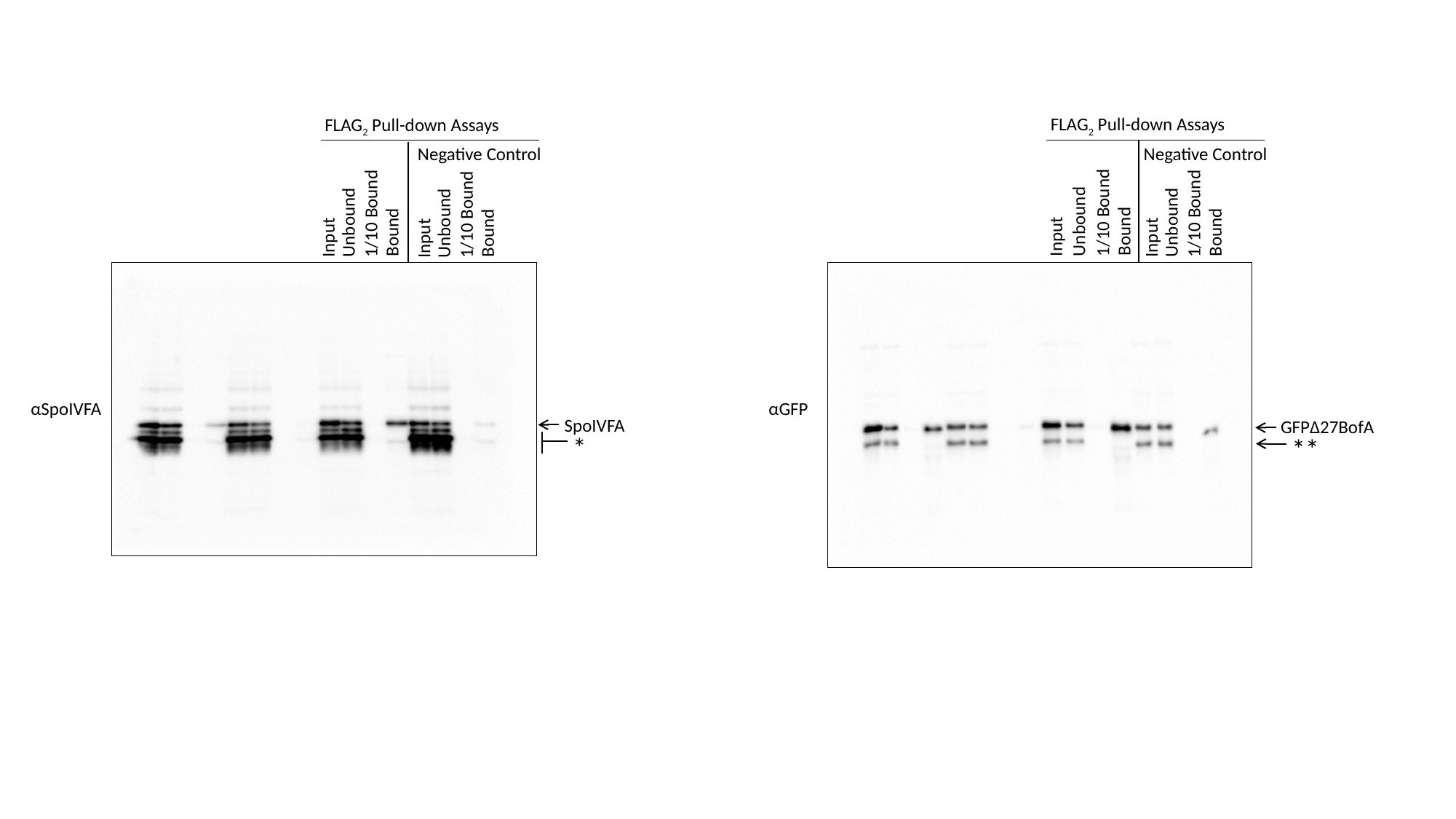

FLAG2 Pull-down Assays
FLAG2 Pull-down Assays
Negative Control
Negative Control
1/10 Bound
1/10 Bound
1/10 Bound
1/10 Bound
Unbound
Unbound
Unbound
Unbound
Bound
Bound
Bound
Bound
Input
Input
Input
Input
αSpoIVFA
αGFP
SpoIVFA
GFPΔ27BofA
*
**
