# Supplementary material for: Inhibitory proteins block substrate access by occupying the active site cleft of Bacillus subtilis intramembrane protease SpoIVFB
Source: eLife. 2022 Apr 26;11:e74275. doi: 10.7554/eLife.74275 (PMC9042235; doi:10.7554/eLife.74275)
Supplement: Figure 5—figure supplement 1—source data 1. [file elife-74275-fig5-figsupp1-data1.zip › Figure 5-figure supplement 1-source data 1/fig sup 1 annotated blots.pptx]

## Slide 1
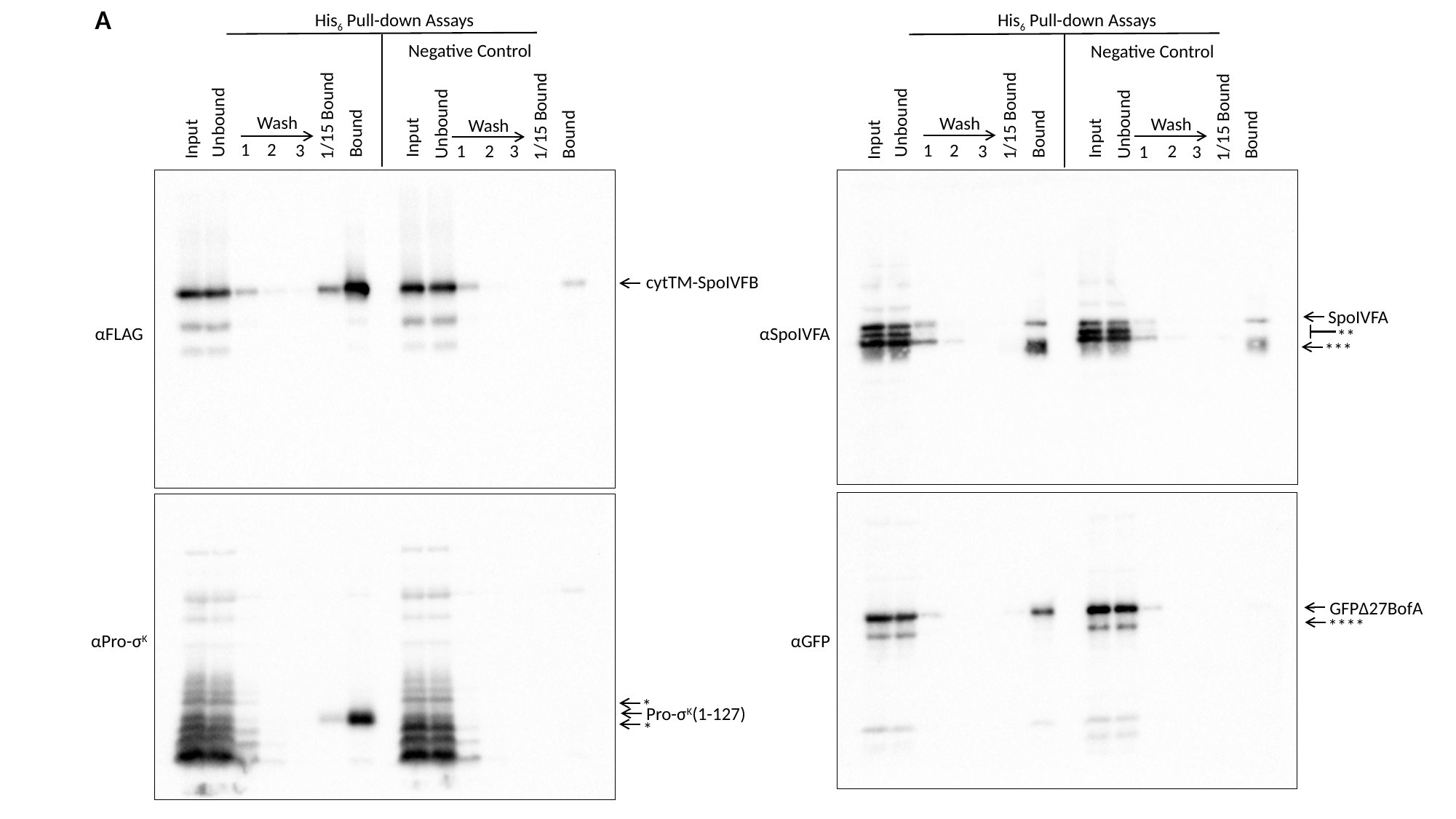

A
His6 Pull-down Assays
His6 Pull-down Assays
Negative Control
Negative Control
1/15 Bound
1/15 Bound
1/15 Bound
1/15 Bound
Unbound
Wash
Unbound
Wash
Wash
Unbound
Unbound
Wash
Bound
Bound
Bound
Bound
Input
Input
Input
Input
1
2
1
2
3
2
3
1
2
3
1
3
cytTM-SpoIVFB
SpoIVFA
αSpoIVFA
αFLAG
**
***
GFPΔ27BofA
****
αPro-σK
αGFP
*
Pro-σK(1-127)
*

## Slide 2
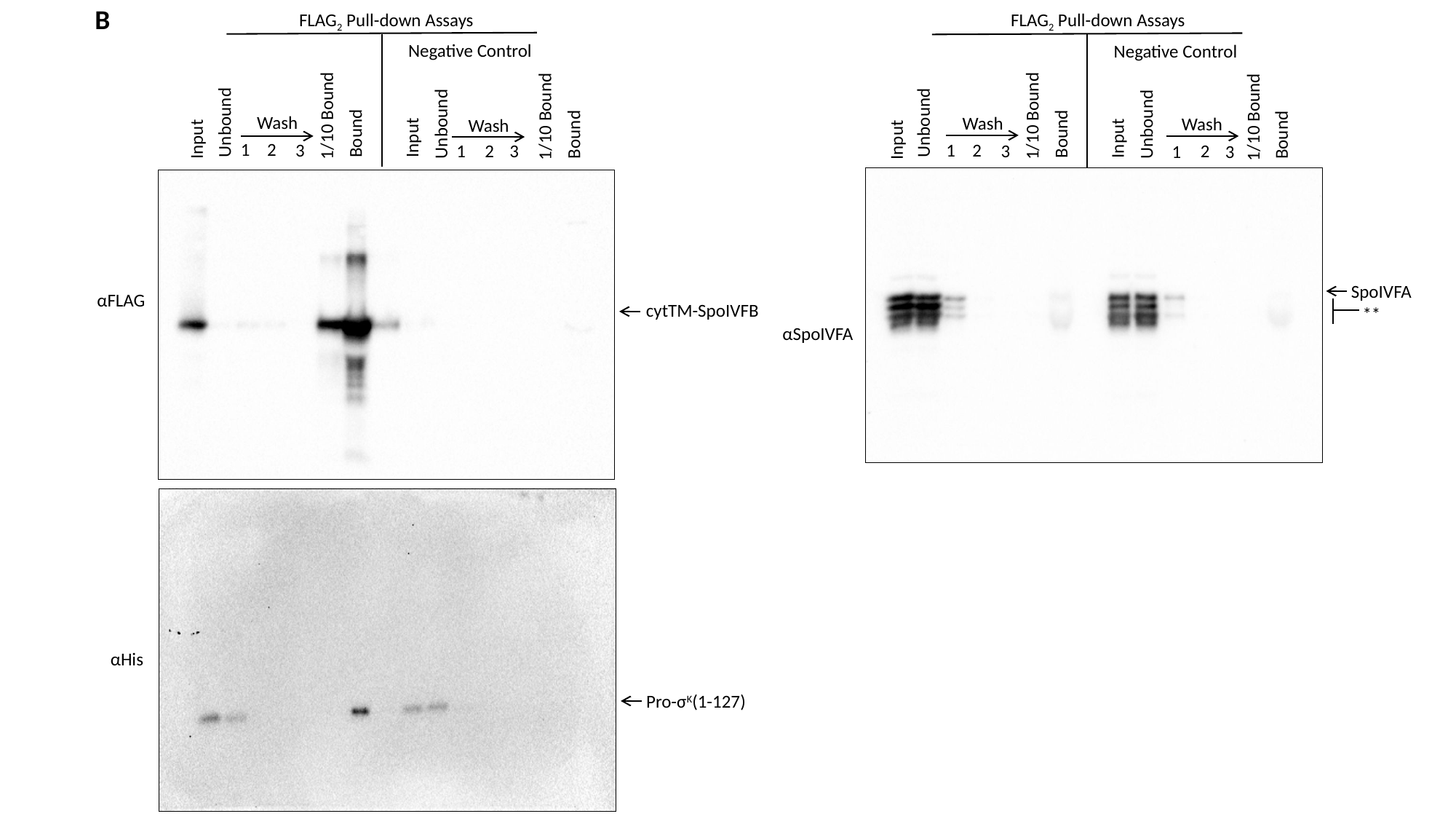

B
FLAG2 Pull-down Assays
FLAG2 Pull-down Assays
Negative Control
Negative Control
1/10 Bound
1/10 Bound
1/10 Bound
1/10 Bound
Unbound
Wash
Unbound
Wash
Wash
Unbound
Unbound
Wash
Bound
Bound
Bound
Bound
Input
Input
Input
Input
1
2
1
2
3
2
3
1
2
3
1
3
SpoIVFA
αFLAG
cytTM-SpoIVFB
**
αSpoIVFA
αHis
Pro-σK(1-127)

## Slide 3
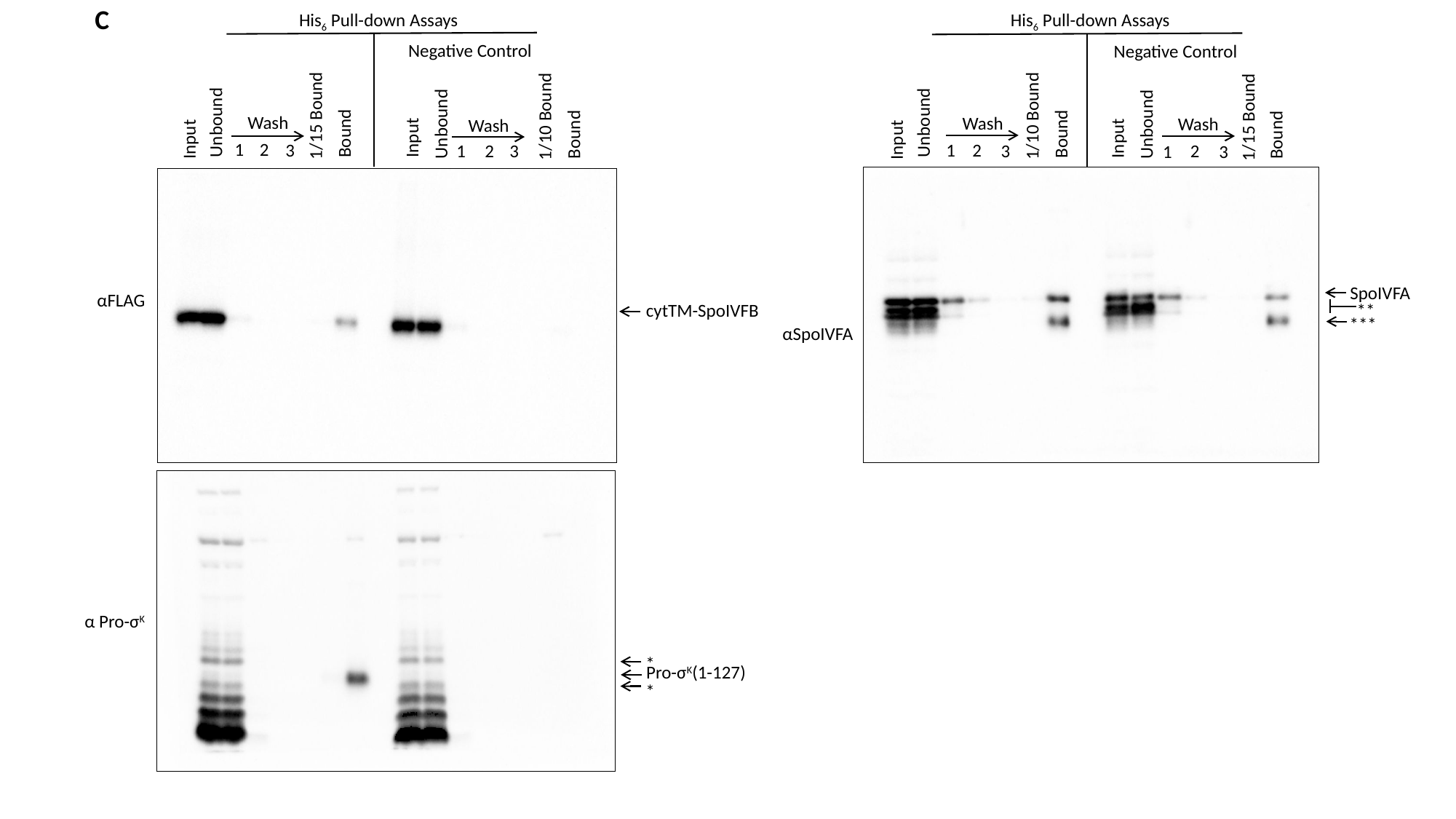

C
His6 Pull-down Assays
His6 Pull-down Assays
Negative Control
Negative Control
1/15 Bound
1/10 Bound
1/10 Bound
1/15 Bound
Unbound
Wash
Unbound
Wash
Wash
Unbound
Unbound
Wash
Bound
Bound
Bound
Bound
Input
Input
Input
Input
1
2
1
2
3
2
3
1
2
3
1
3
SpoIVFA
αFLAG
cytTM-SpoIVFB
**
***
αSpoIVFA
α Pro-σK
*
Pro-σK(1-127)
*
