# Supplementary material for: Inhibitory proteins block substrate access by occupying the active site cleft of Bacillus subtilis intramembrane protease SpoIVFB
Source: eLife. 2022 Apr 26;11:e74275. doi: 10.7554/eLife.74275 (PMC9042235; doi:10.7554/eLife.74275)
Supplement: Figure 5—figure supplement 2—source data 1. [file elife-74275-fig5-figsupp2-data1.zip › Figure 5-figure supplement 2-source data 1/fig sup 2 annotated blots.pptx]

## Slide 1
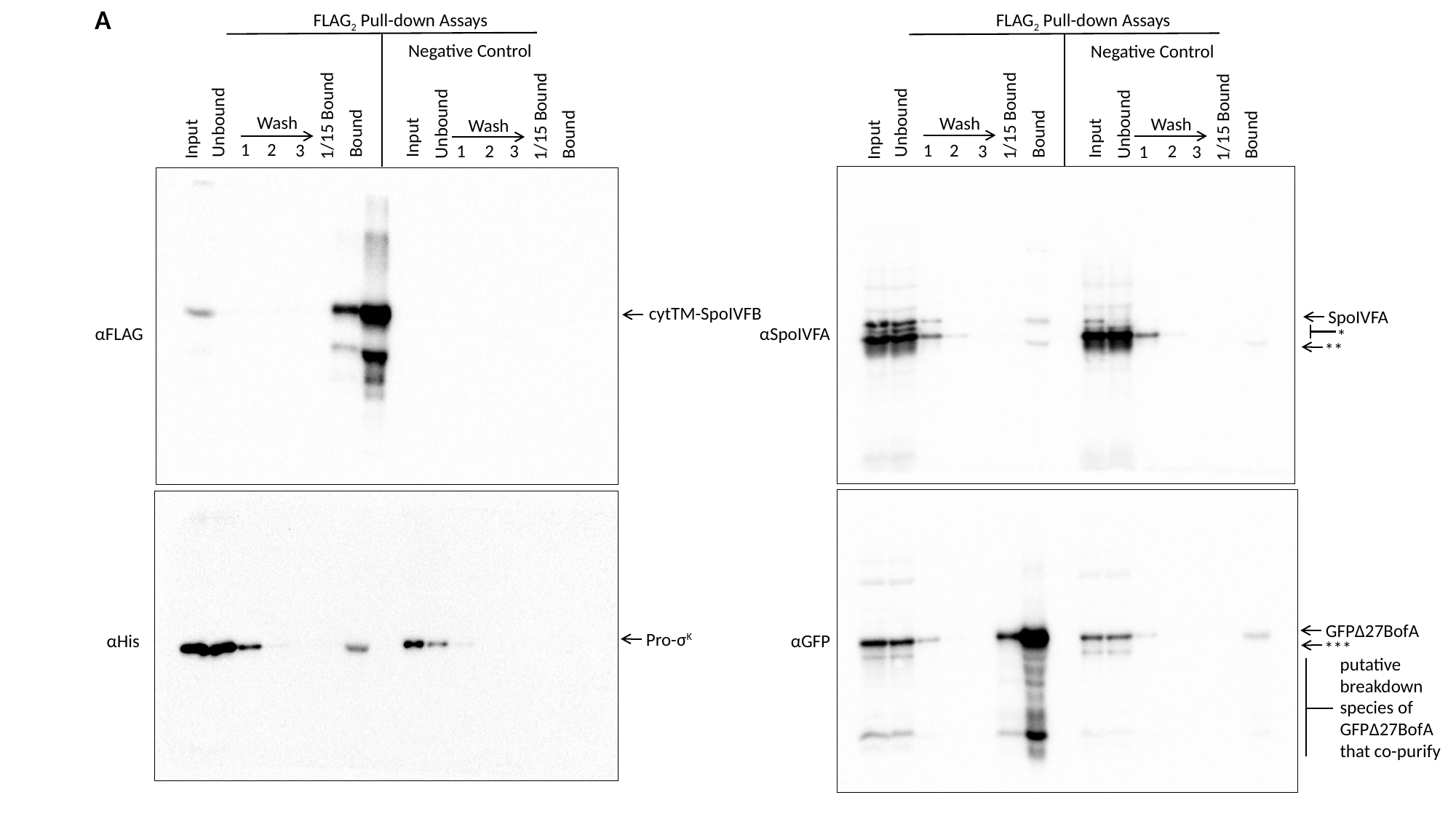

A
FLAG2 Pull-down Assays
FLAG2 Pull-down Assays
Negative Control
Negative Control
1/15 Bound
1/15 Bound
1/15 Bound
1/15 Bound
Unbound
Wash
Unbound
Wash
Wash
Unbound
Unbound
Wash
Bound
Bound
Bound
Bound
Input
Input
Input
Input
1
2
1
2
3
2
3
1
2
3
1
3
cytTM-SpoIVFB
SpoIVFA
αSpoIVFA
αFLAG
*
**
GFPΔ27BofA
Pro-σK
αHis
αGFP
***
putative breakdown species of GFPΔ27BofA that co-purify

## Slide 2
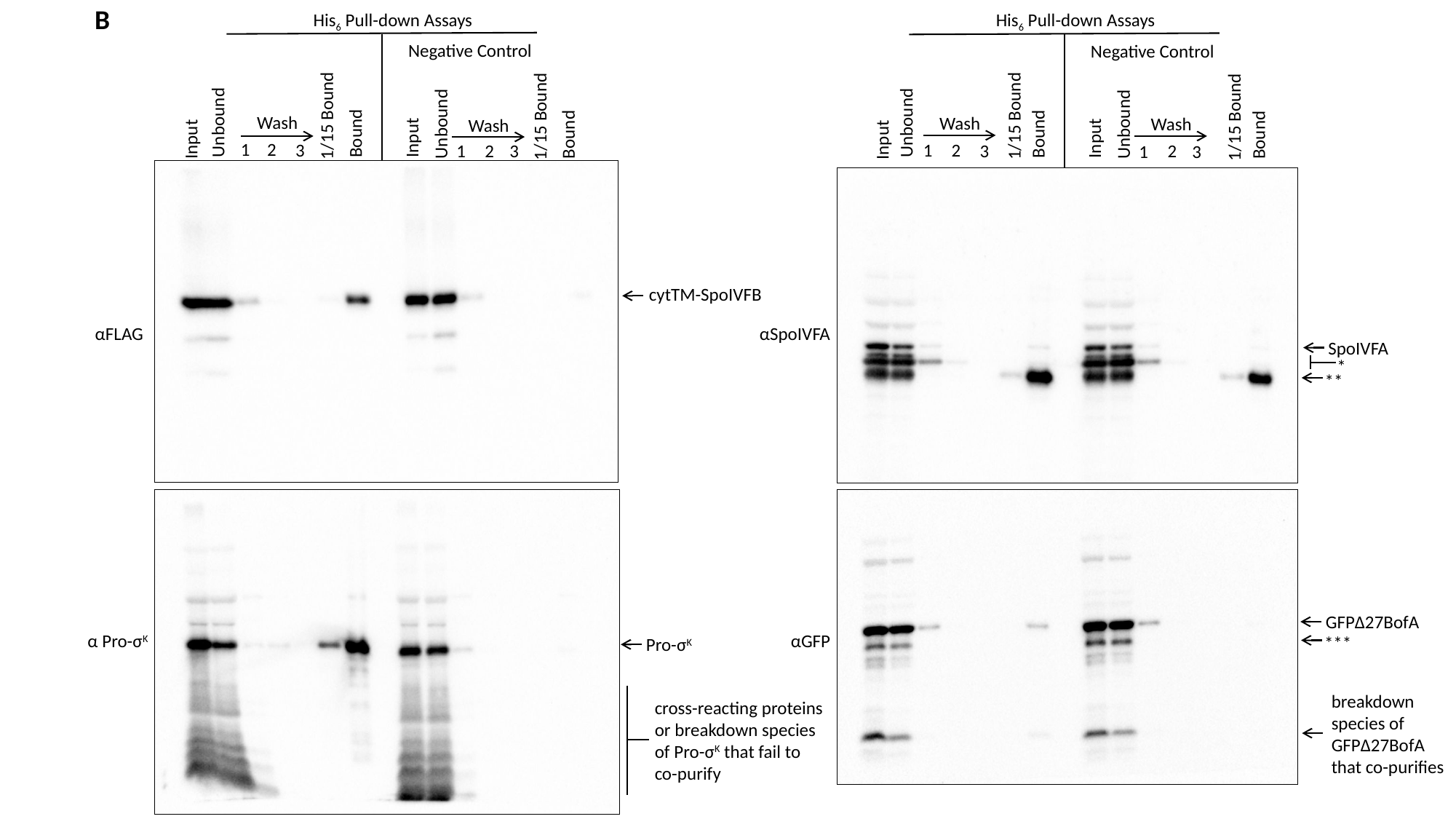

B
His6 Pull-down Assays
His6 Pull-down Assays
Negative Control
Negative Control
1/15 Bound
1/15 Bound
1/15 Bound
1/15 Bound
Unbound
Wash
Unbound
Wash
Wash
Unbound
Unbound
Wash
Bound
Bound
Bound
Bound
Input
Input
Input
Input
1
2
1
2
3
2
3
1
2
3
1
3
cytTM-SpoIVFB
αSpoIVFA
αFLAG
SpoIVFA
*
**
GFPΔ27BofA
α Pro-σK
αGFP
***
Pro-σK
breakdown species of GFPΔ27BofA that co-purifies
cross-reacting proteins or breakdown species of Pro-σK that fail to co-purify
