# Supplementary material for: Inhibitory proteins block substrate access by occupying the active site cleft of Bacillus subtilis intramembrane protease SpoIVFB
Source: eLife. 2022 Apr 26;11:e74275. doi: 10.7554/eLife.74275 (PMC9042235; doi:10.7554/eLife.74275)
Supplement: Figure 5—figure supplement 3—source data 1. [file elife-74275-fig5-figsupp3-data1.zip › Figure 5-figure supplement 3-source data 1/figure supplement 3A/fig sup 3A annotated blots.pptx]

## Slide 1
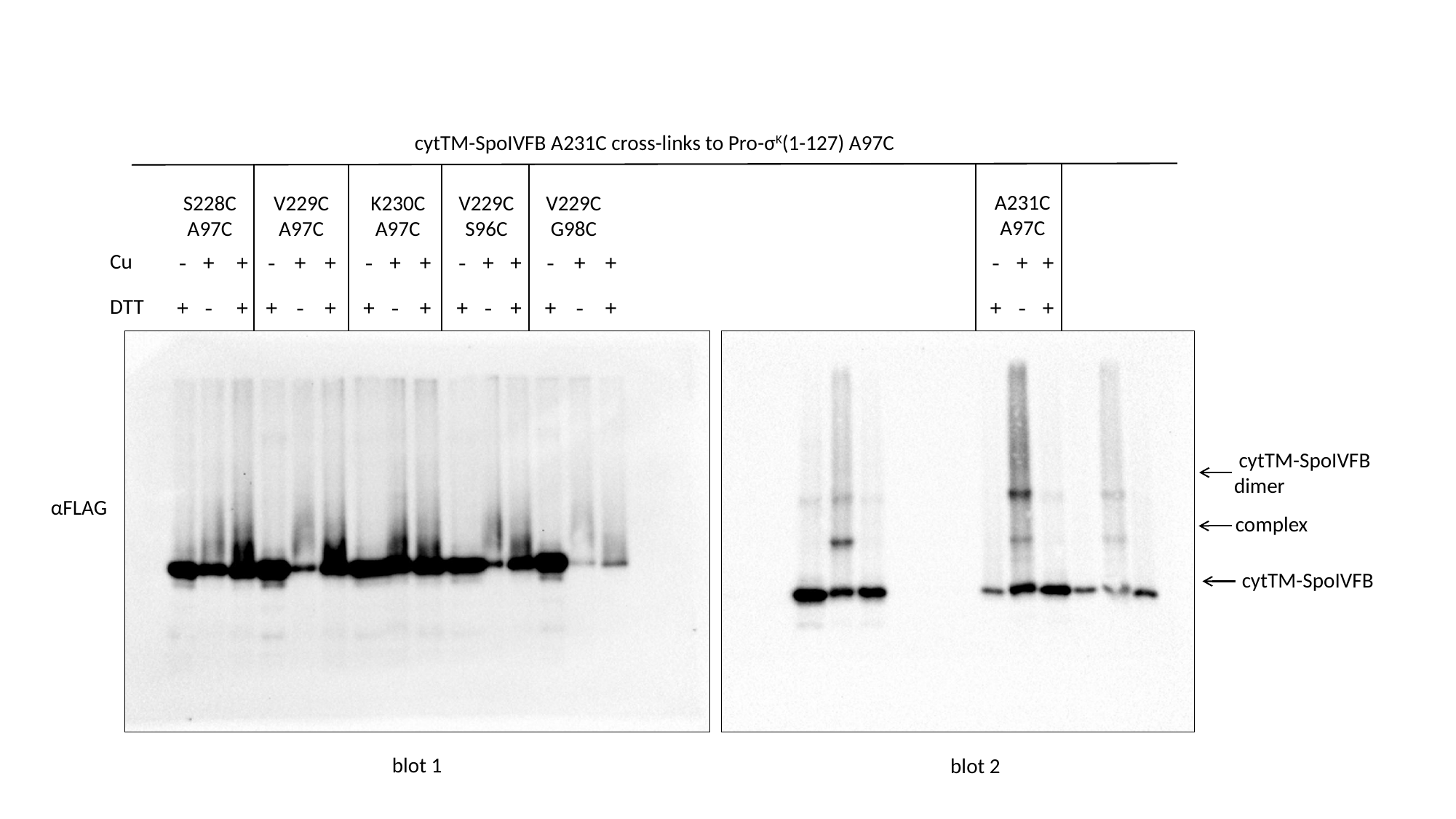

cytTM-SpoIVFB A231C cross-links to Pro-σK(1-127) A97C
A231C
A97C
S228C
A97C
V229C
A97C
K230C
A97C
V229C
S96C
V229C
G98C
| Cu | - | + | + | - | + | + | - | + | + | - | + | + | - | + | + | - | + | + |
| --- | --- | --- | --- | --- | --- | --- | --- | --- | --- | --- | --- | --- | --- | --- | --- | --- | --- | --- |
| DTT | + | - | + | + | - | + | + | - | + | + | - | + | + | - | + | + | - | + |
 cytTM-SpoIVFB
dimer
αFLAG
complex
 cytTM-SpoIVFB
blot 1
blot 2
