# Supplementary material for: Inhibitory proteins block substrate access by occupying the active site cleft of Bacillus subtilis intramembrane protease SpoIVFB
Source: eLife. 2022 Apr 26;11:e74275. doi: 10.7554/eLife.74275 (PMC9042235; doi:10.7554/eLife.74275)
Supplement: Figure 5—figure supplement 3—source data 1. [file elife-74275-fig5-figsupp3-data1.zip › Figure 5-figure supplement 3-source data 1/figure supplement 3B/fig sup 3B annotated blots.pptx]

## Slide 1
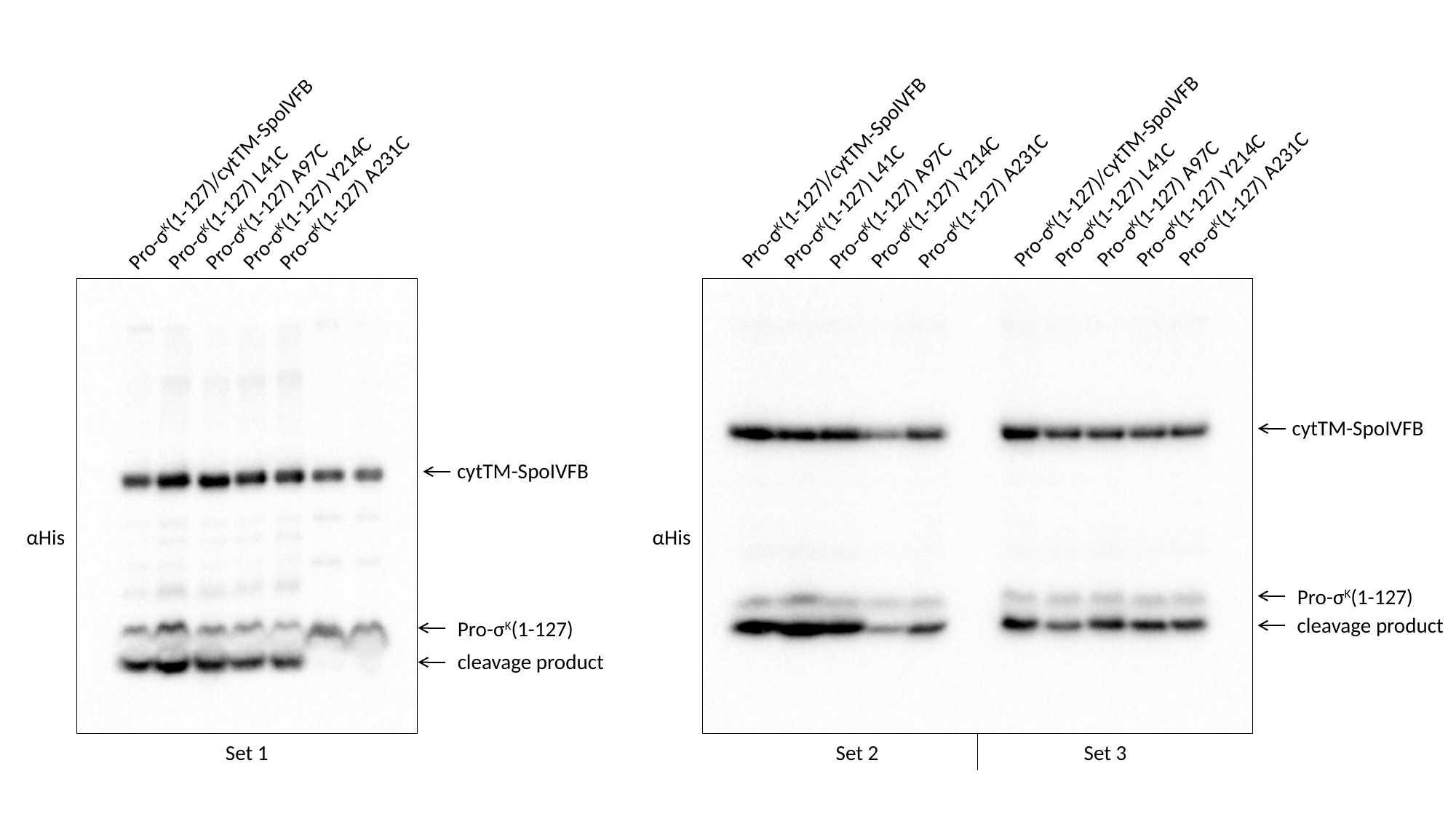

Pro-σK(1-127)/cytTM-SpoIVFB
Pro-σK(1-127)/cytTM-SpoIVFB
Pro-σK(1-127)/cytTM-SpoIVFB
Pro-σK(1-127) A231C
Pro-σK(1-127) Y214C
Pro-σK(1-127) A231C
Pro-σK(1-127) Y214C
Pro-σK(1-127) A231C
Pro-σK(1-127) Y214C
Pro-σK(1-127) A97C
Pro-σK(1-127) L41C
Pro-σK(1-127) A97C
Pro-σK(1-127) A97C
Pro-σK(1-127) L41C
Pro-σK(1-127) L41C
cytTM-SpoIVFB
cytTM-SpoIVFB
αHis
αHis
Pro-σK(1-127)
cleavage product
Pro-σK(1-127)
cleavage product
Set 3
Set 1
Set 2
