# Supplementary figures and images for: Inhibitory proteins block substrate access by occupying the active site cleft of Bacillus subtilis intramembrane protease SpoIVFB (part 3 of 3)
Source: eLife. 2022 Apr 26;11:e74275. doi: 10.7554/eLife.74275 (PMC9042235; doi:10.7554/eLife.74275)

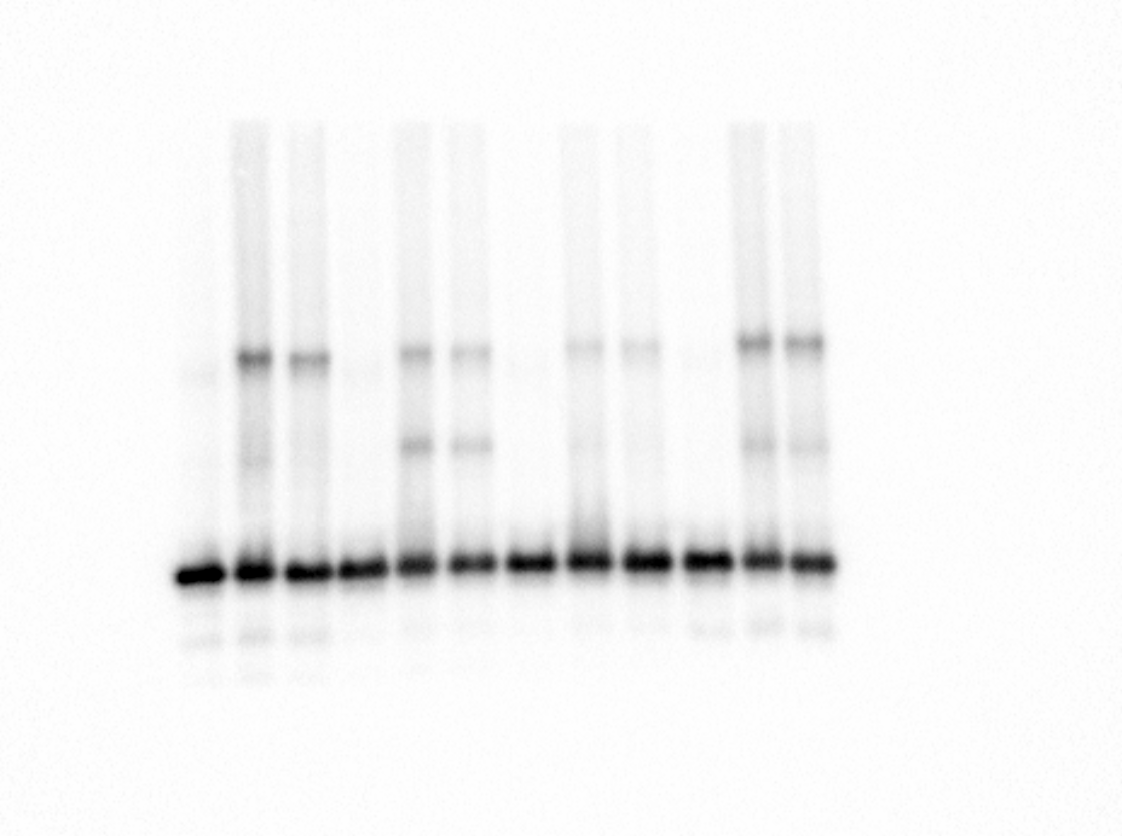

Supplement: Figure 6—figure supplement 1—source data 1. [file elife-74275-fig6-figsupp1-data1.zip › Figure 6-figure supplement 1-source data 1/figure supplement 1A/pSO167, 169, 170 and 128 anti-FLAG 45min Set2.tif]

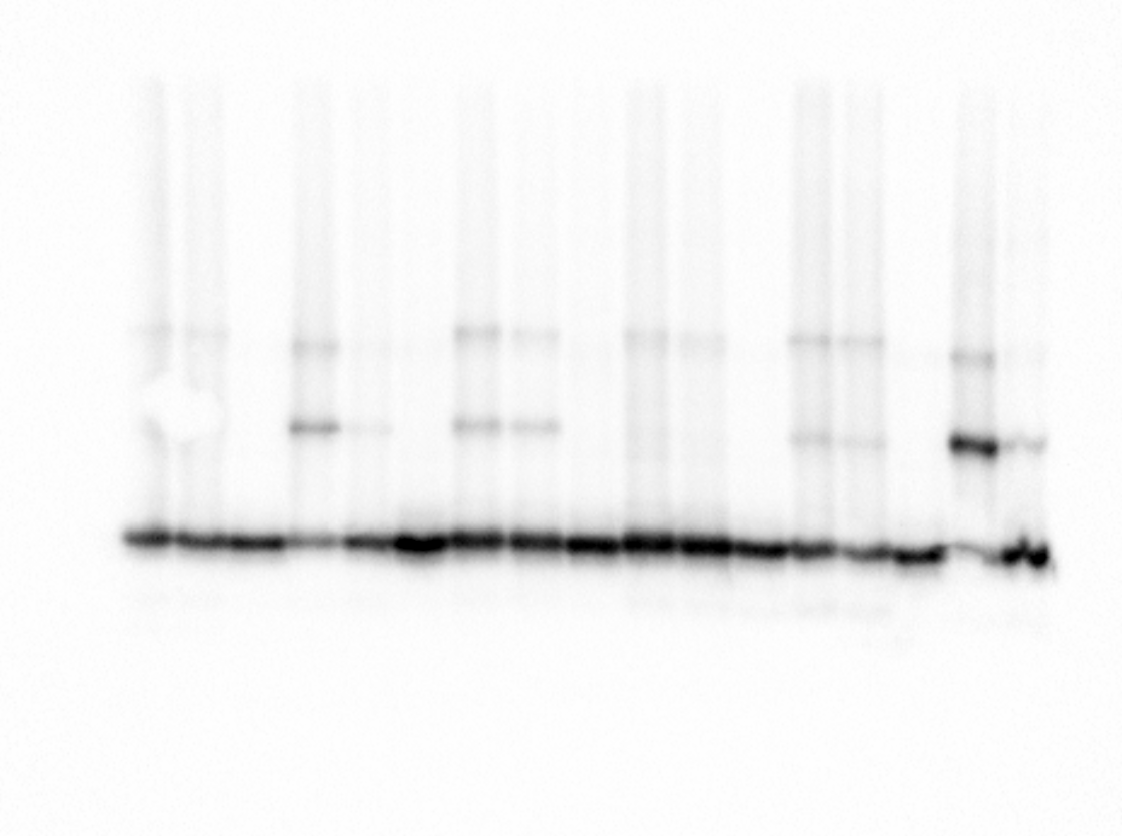

Supplement: Figure 6—figure supplement 1—source data 1. [file elife-74275-fig6-figsupp1-data1.zip › Figure 6-figure supplement 1-source data 1/figure supplement 1A/pSO168, 169, 170 and 128 anti-FLAG 15min Set1.tif]

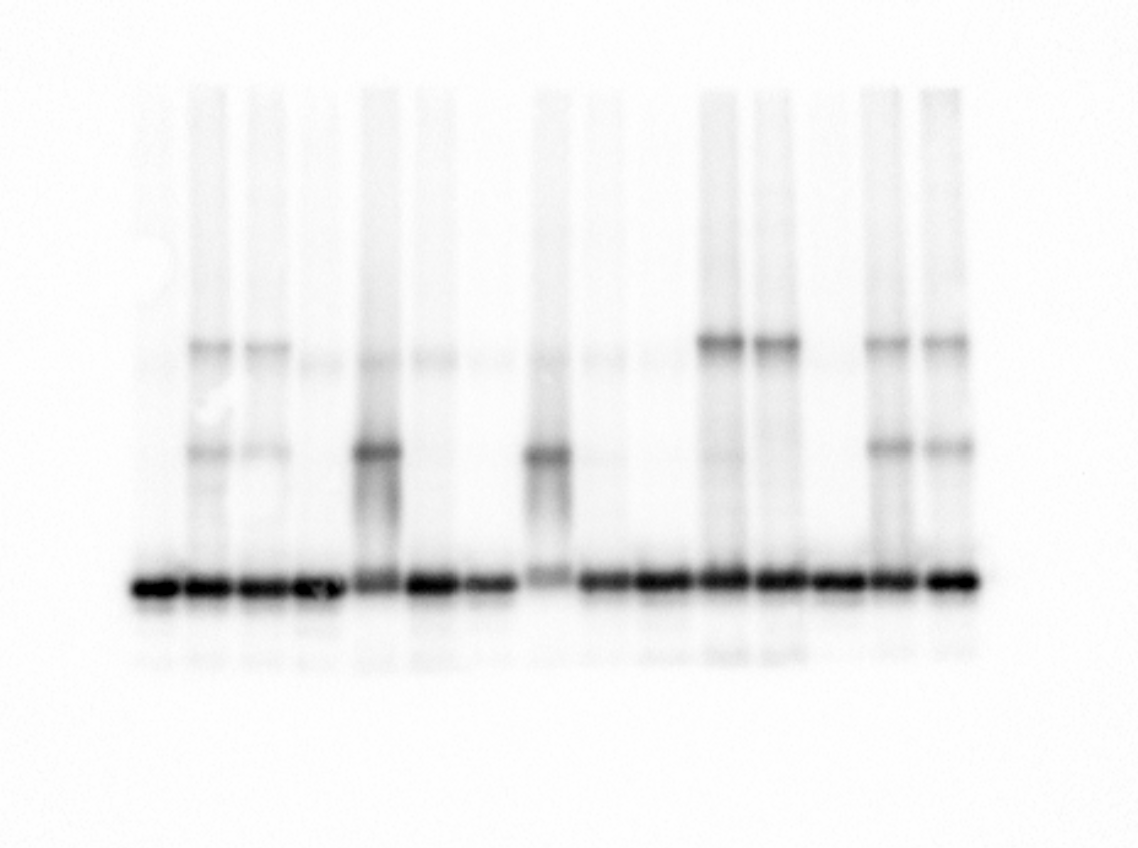

Supplement: Figure 6—figure supplement 1—source data 1. [file elife-74275-fig6-figsupp1-data1.zip › Figure 6-figure supplement 1-source data 1/figure supplement 1A/pSO169 30min, 168 and 134 45min, 167 and 169 60min anti-FLAG Set2.tif]

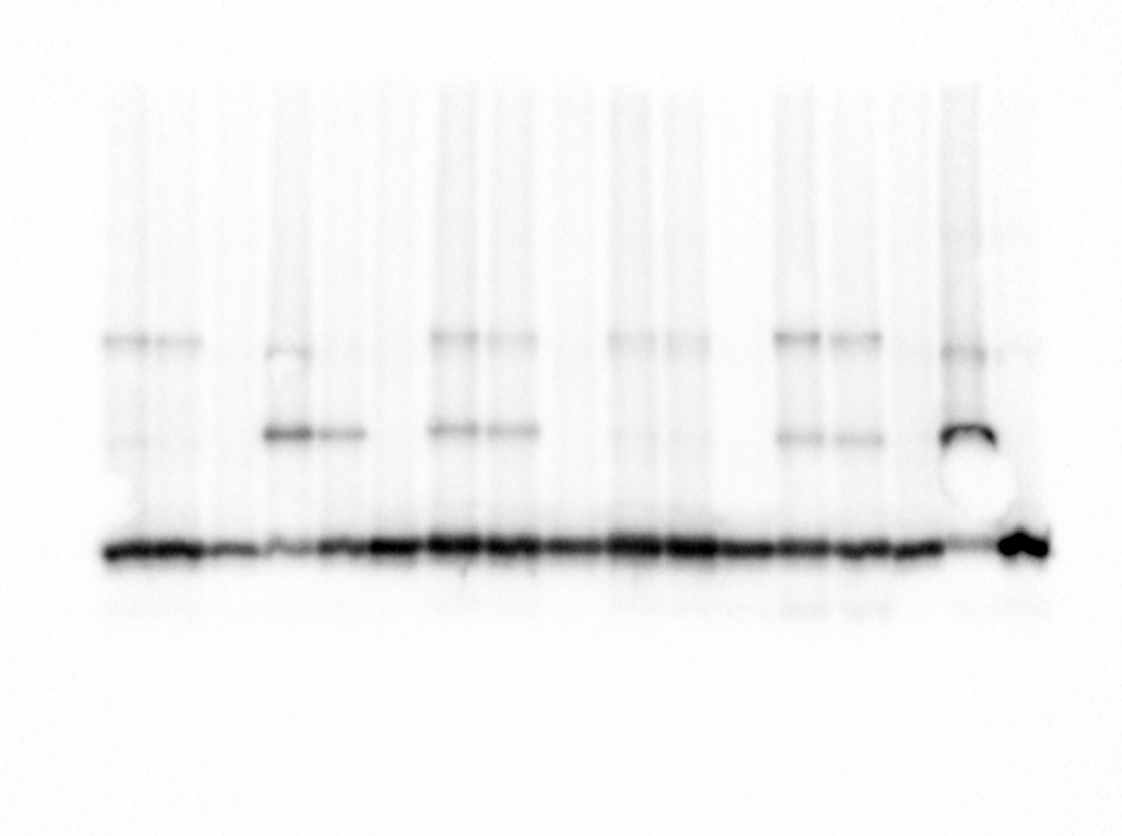

Supplement: Figure 6—figure supplement 1—source data 1. [file elife-74275-fig6-figsupp1-data1.zip › Figure 6-figure supplement 1-source data 1/figure supplement 1A/pSO169, 170, 128 anti-FLAG 30min Set1.tif]

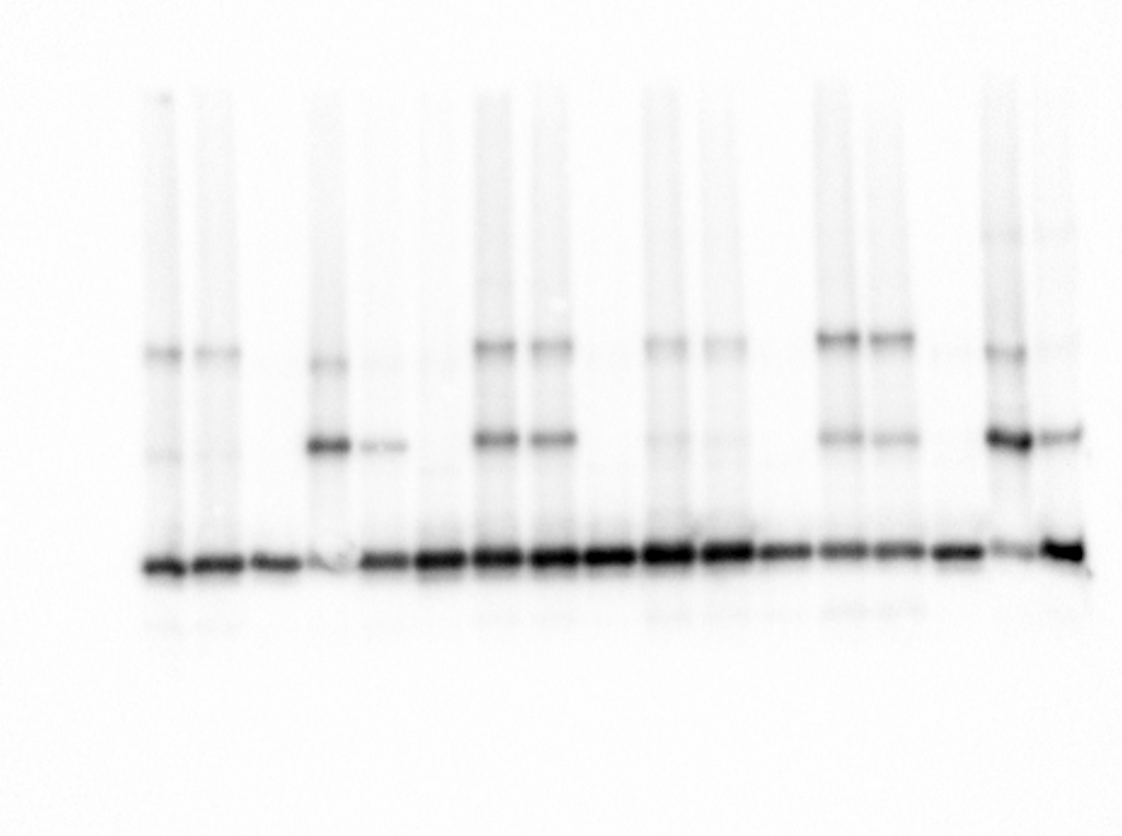

Supplement: Figure 6—figure supplement 1—source data 1. [file elife-74275-fig6-figsupp1-data1.zip › Figure 6-figure supplement 1-source data 1/figure supplement 1A/pSO169, 170, 128 anti-FLAG 60min Set1.tif]

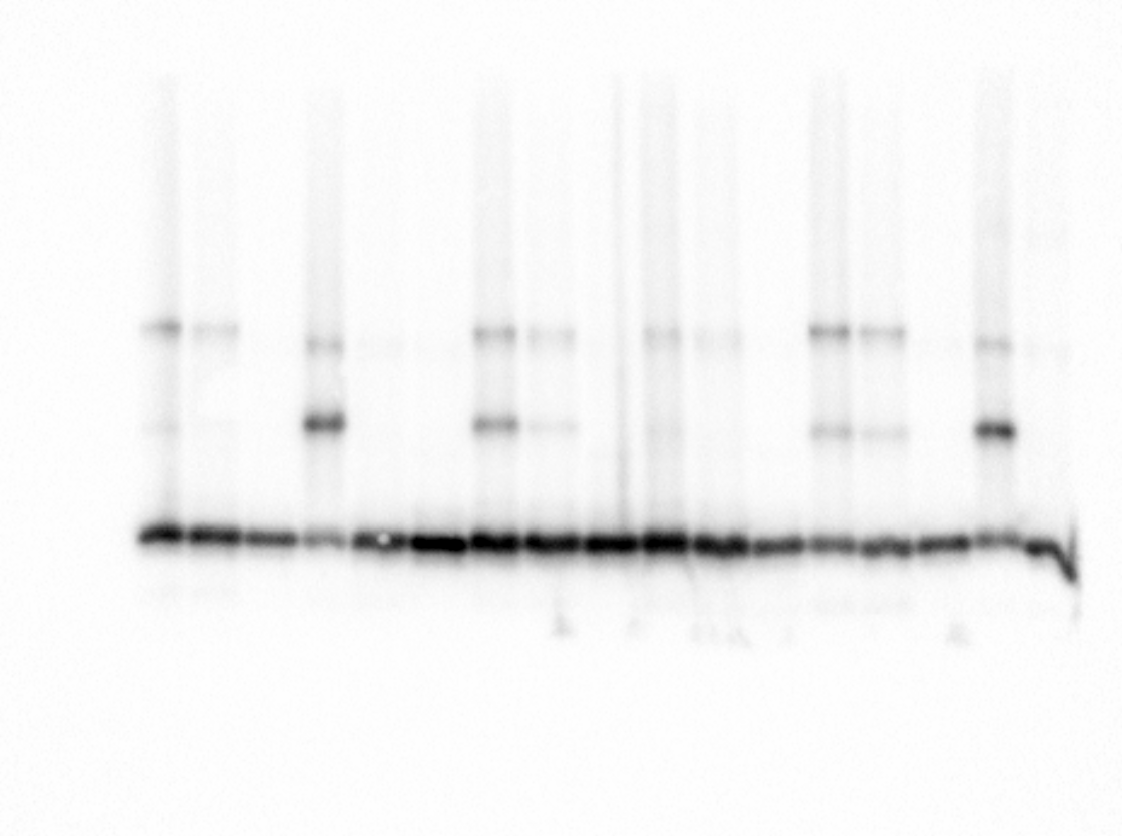

Supplement: Figure 6—figure supplement 1—source data 1. [file elife-74275-fig6-figsupp1-data1.zip › Figure 6-figure supplement 1-source data 1/figure supplement 1A/pSO169. 170, 128 anti-FLAG 45min Set1.tif]

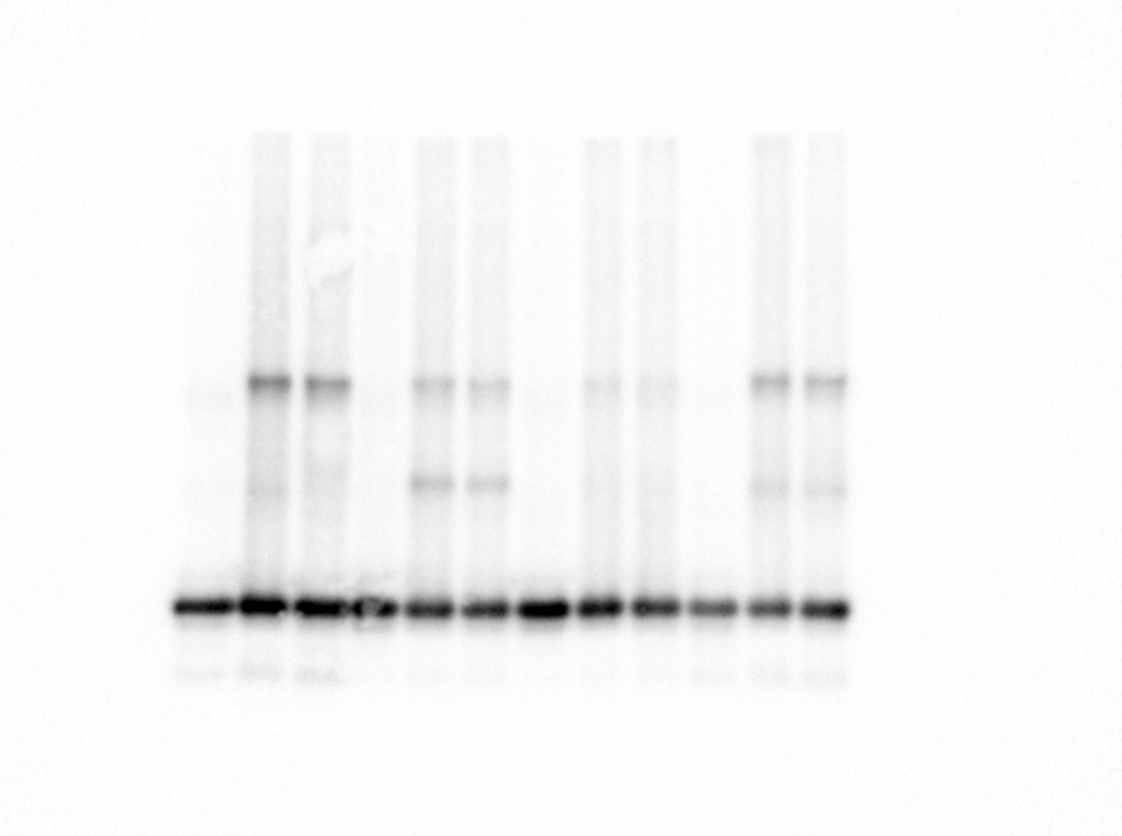

Supplement: Figure 6—figure supplement 1—source data 1. [file elife-74275-fig6-figsupp1-data1.zip › Figure 6-figure supplement 1-source data 1/figure supplement 1A/pSO170 and 128 anti-FLAG 30min Set2.tif]

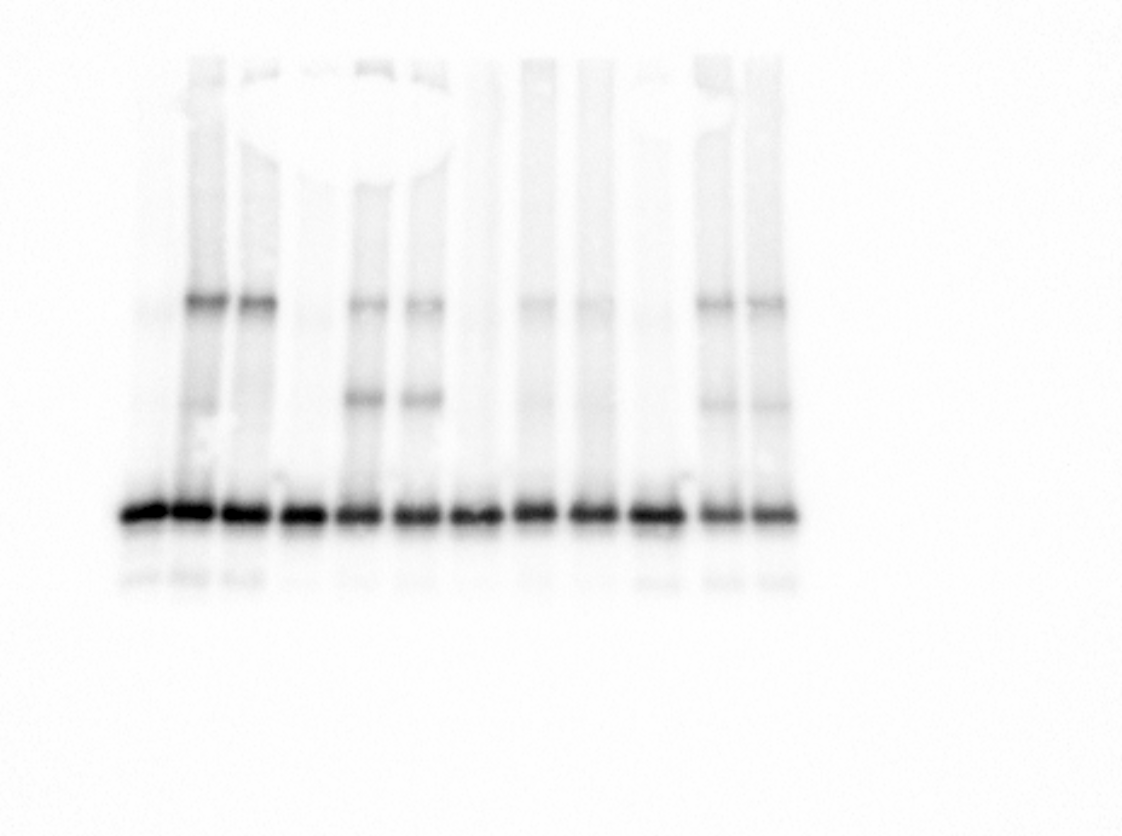

Supplement: Figure 6—figure supplement 1—source data 1. [file elife-74275-fig6-figsupp1-data1.zip › Figure 6-figure supplement 1-source data 1/figure supplement 1A/pSO170 and 128 anti-FLAG 60min Set2.tif]

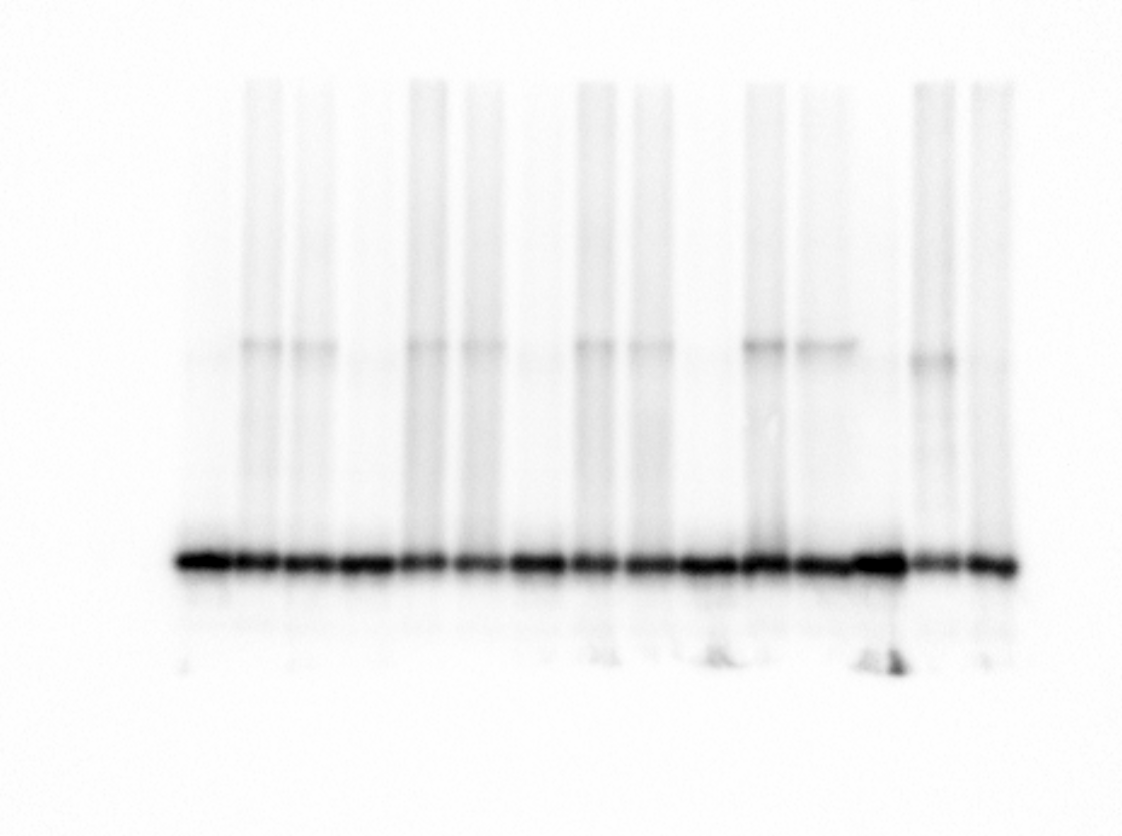

Supplement: Figure 6—figure supplement 1—source data 1. [file elife-74275-fig6-figsupp1-data1.zip › Figure 6-figure supplement 1-source data 1/figure supplement 1A/pSO79 15-45min Set2 and pSO136 45min Set1 anti-FLAG.tif]

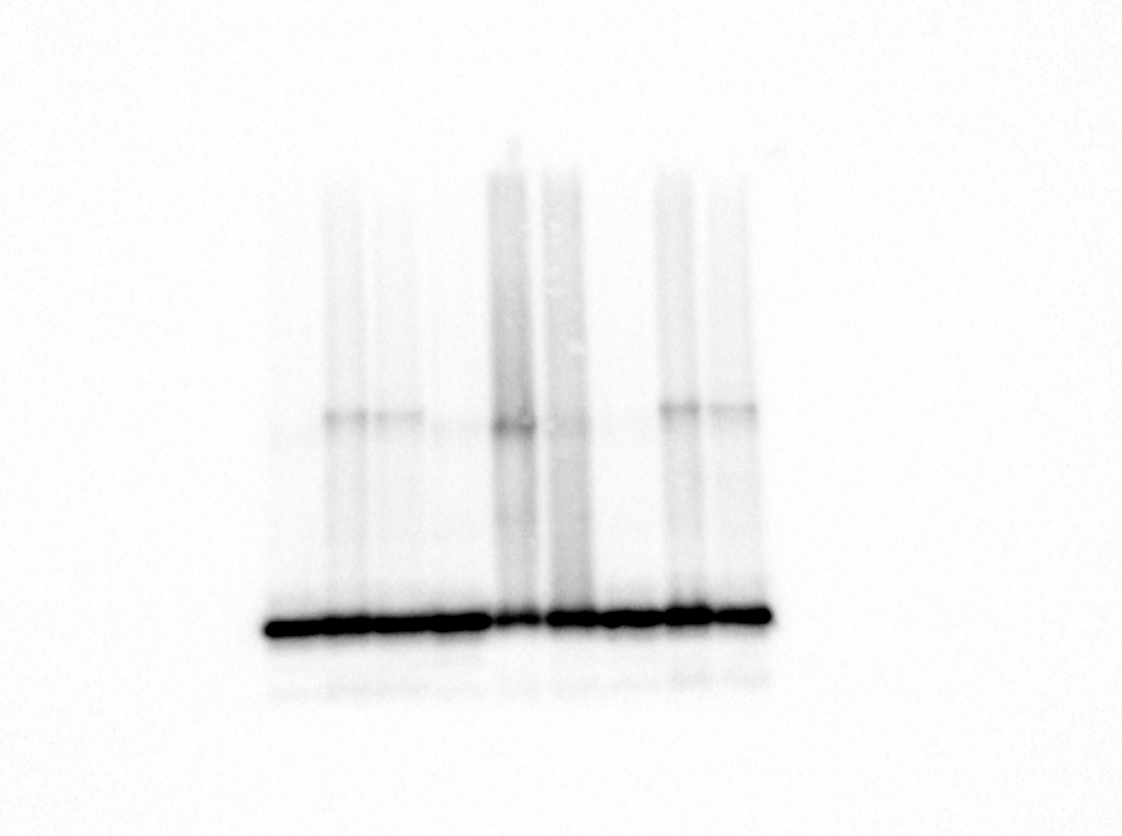

Supplement: Figure 6—figure supplement 1—source data 1. [file elife-74275-fig6-figsupp1-data1.zip › Figure 6-figure supplement 1-source data 1/figure supplement 1A/pSO79 30 and 60min anti-FLAG Set1.tif]

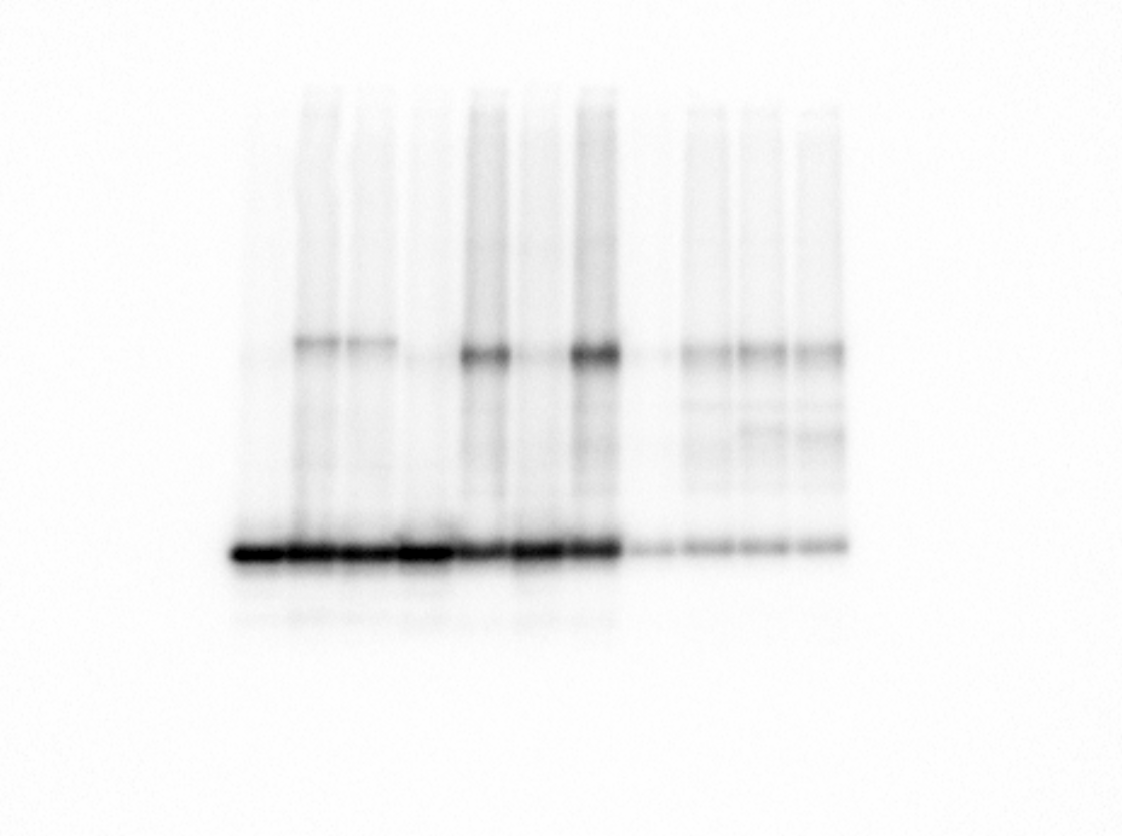

Supplement: Figure 6—figure supplement 1—source data 1. [file elife-74275-fig6-figsupp1-data1.zip › Figure 6-figure supplement 1-source data 1/figure supplement 1A/pSO79 60min and pSO136 30min anti-FLAG Set2.tif]

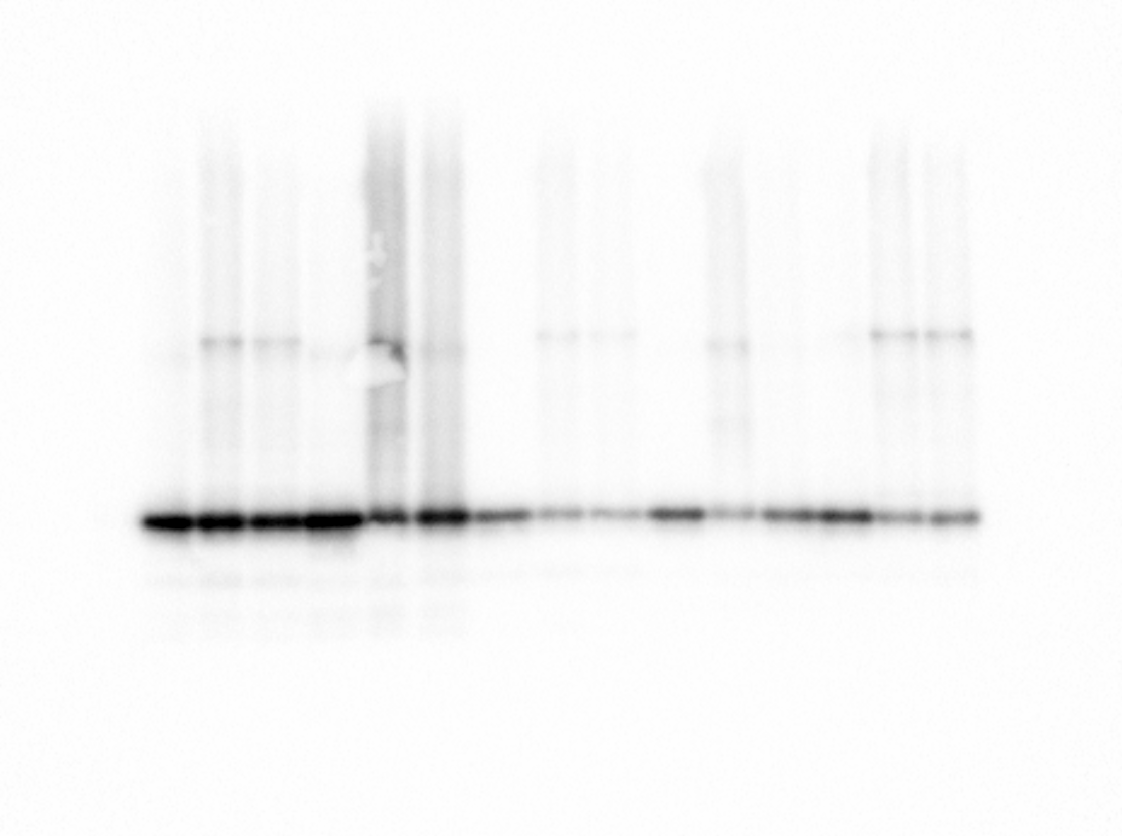

Supplement: Figure 6—figure supplement 1—source data 1. [file elife-74275-fig6-figsupp1-data1.zip › Figure 6-figure supplement 1-source data 1/figure supplement 1A/pSO79, 110 and 111 45min Set1 and pSO229 45min Set2 anti-FLAG.tif]

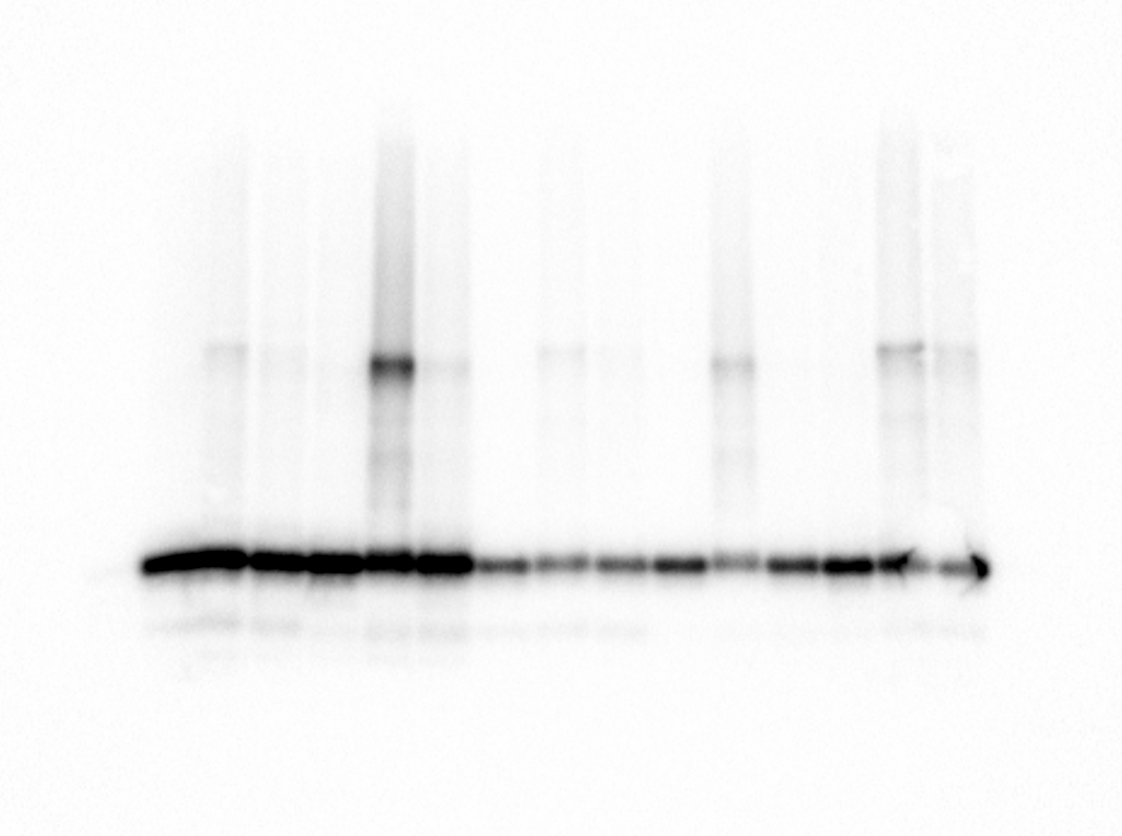

Supplement: Figure 6—figure supplement 1—source data 1. [file elife-74275-fig6-figsupp1-data1.zip › Figure 6-figure supplement 1-source data 1/figure supplement 1A/pSO79, 136, 110 and 111 15min anti-FLAG Set1.tif]

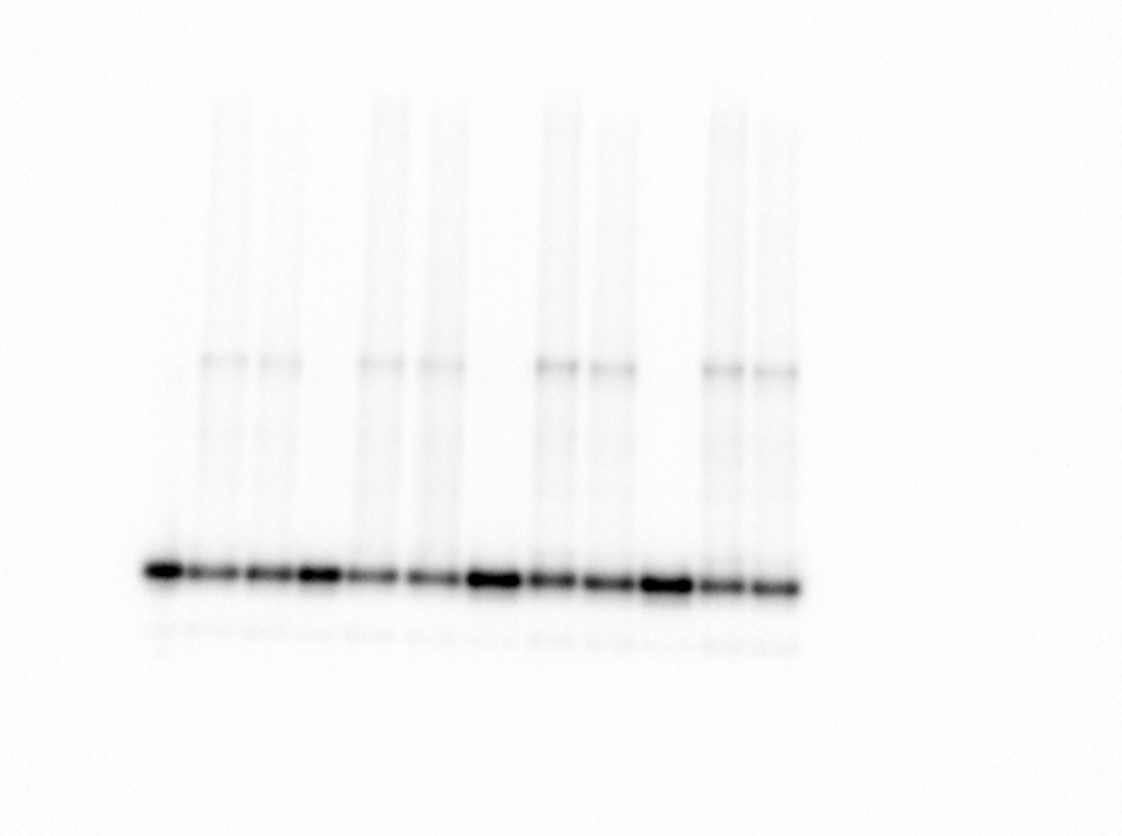

Supplement: Figure 6—figure supplement 1—source data 2. [file elife-74275-fig6-figsupp1-data2.zip › Figure 6-figure supplement 1-source data 2/figure supplement 1B/pSO110 15-60min anti-FLAG Set2.tif]

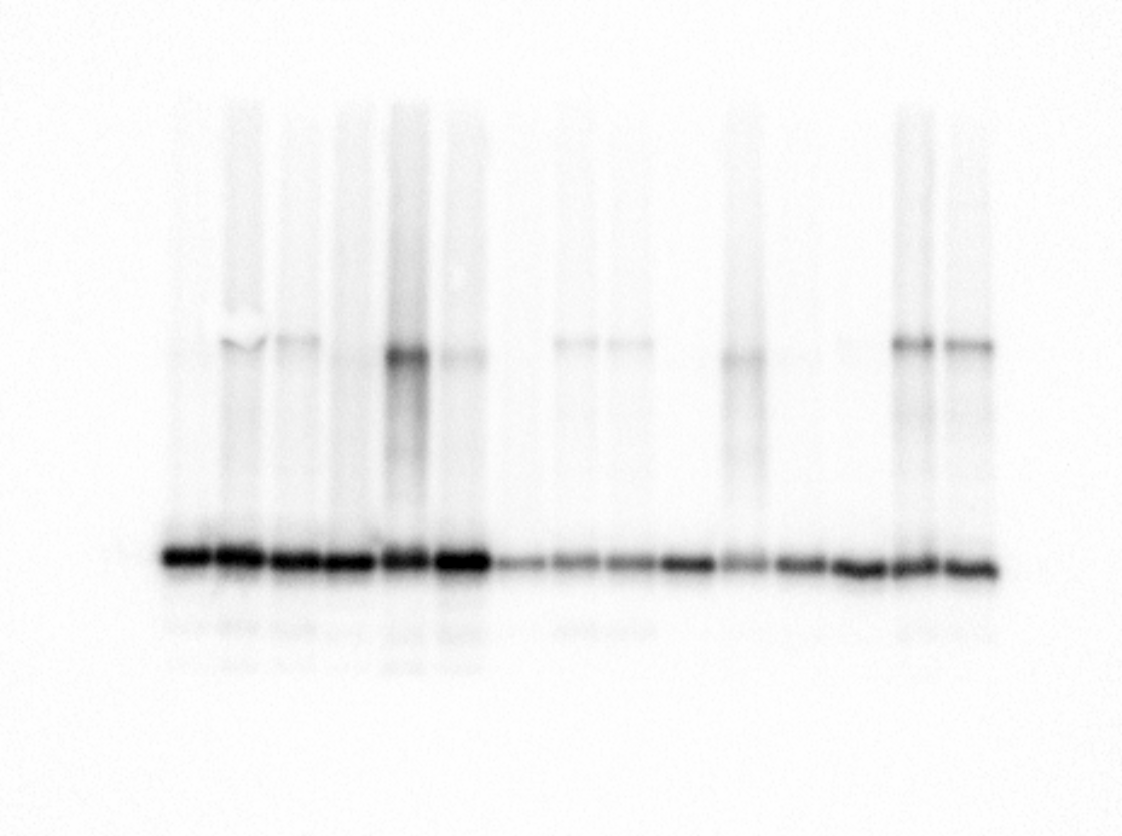

Supplement: Figure 6—figure supplement 1—source data 2. [file elife-74275-fig6-figsupp1-data2.zip › Figure 6-figure supplement 1-source data 2/figure supplement 1B/pSO110, 111 and 136 30min Set1 and pSO229 30min Set2 anti-FLAG.tif]

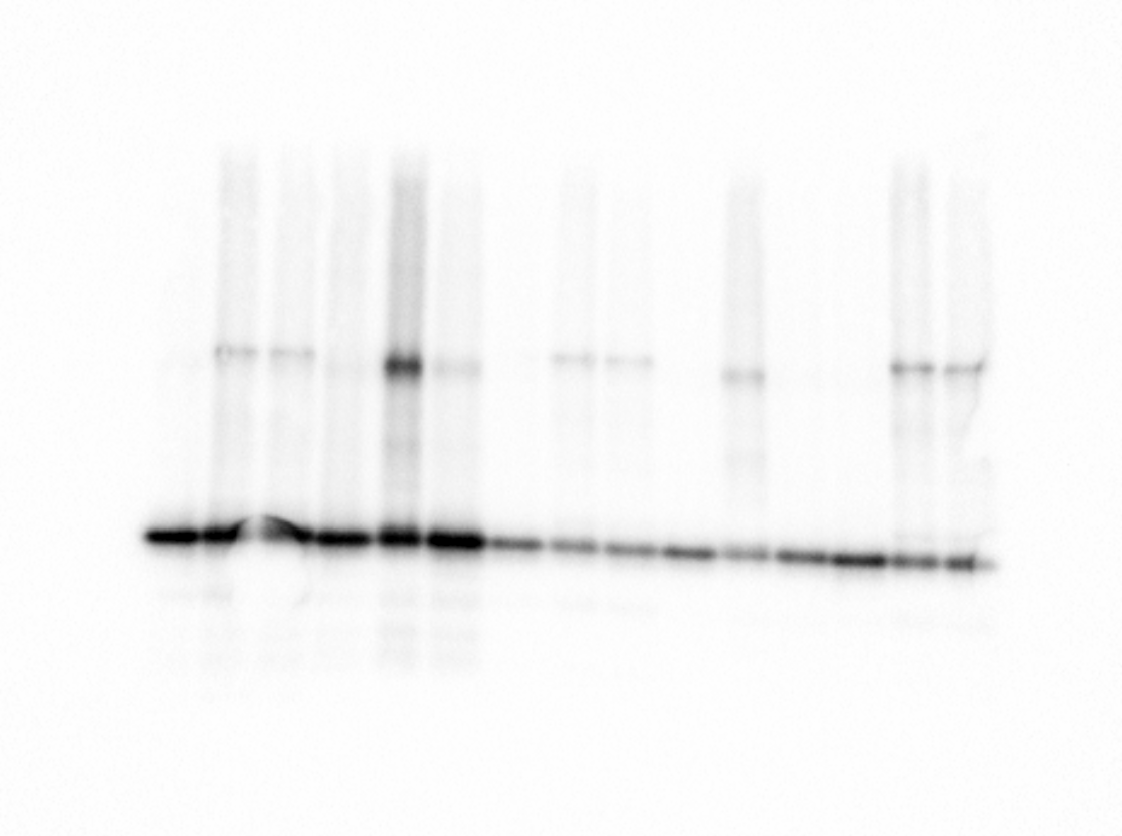

Supplement: Figure 6—figure supplement 1—source data 2. [file elife-74275-fig6-figsupp1-data2.zip › Figure 6-figure supplement 1-source data 2/figure supplement 1B/pSO110, 111 and 136 60min anti-FLAG Set1.tif]

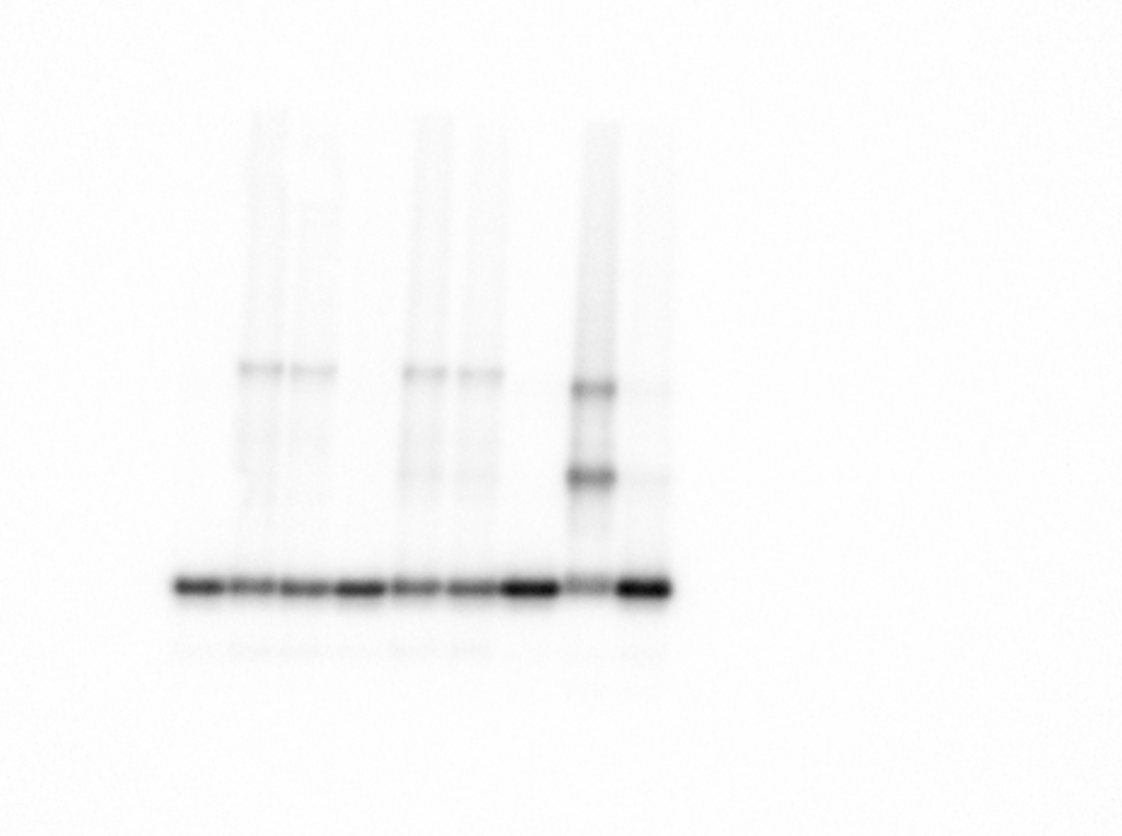

Supplement: Figure 6—figure supplement 1—source data 2. [file elife-74275-fig6-figsupp1-data2.zip › Figure 6-figure supplement 1-source data 2/figure supplement 1B/pSO131 and 132 15min anti-FLAG Set2.tif]

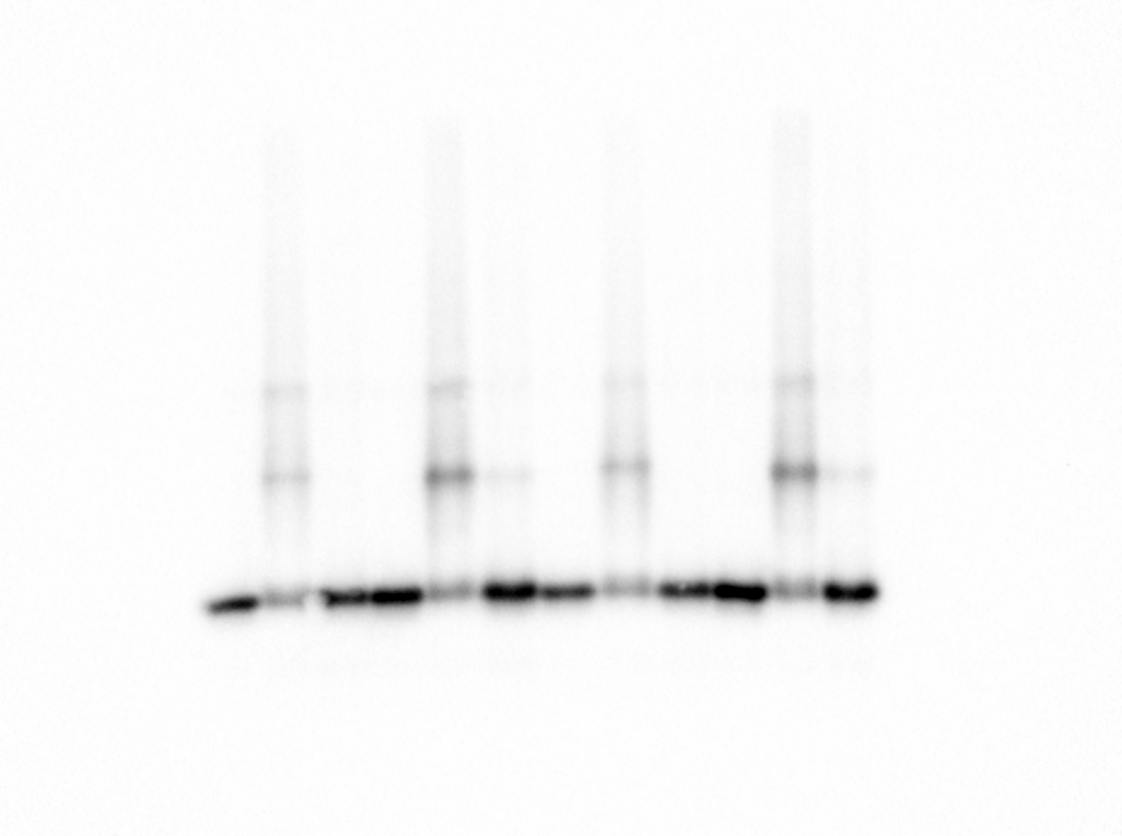

Supplement: Figure 6—figure supplement 1—source data 2. [file elife-74275-fig6-figsupp1-data2.zip › Figure 6-figure supplement 1-source data 2/figure supplement 1B/pSO132 30min anti-FLAG Set2.tif]

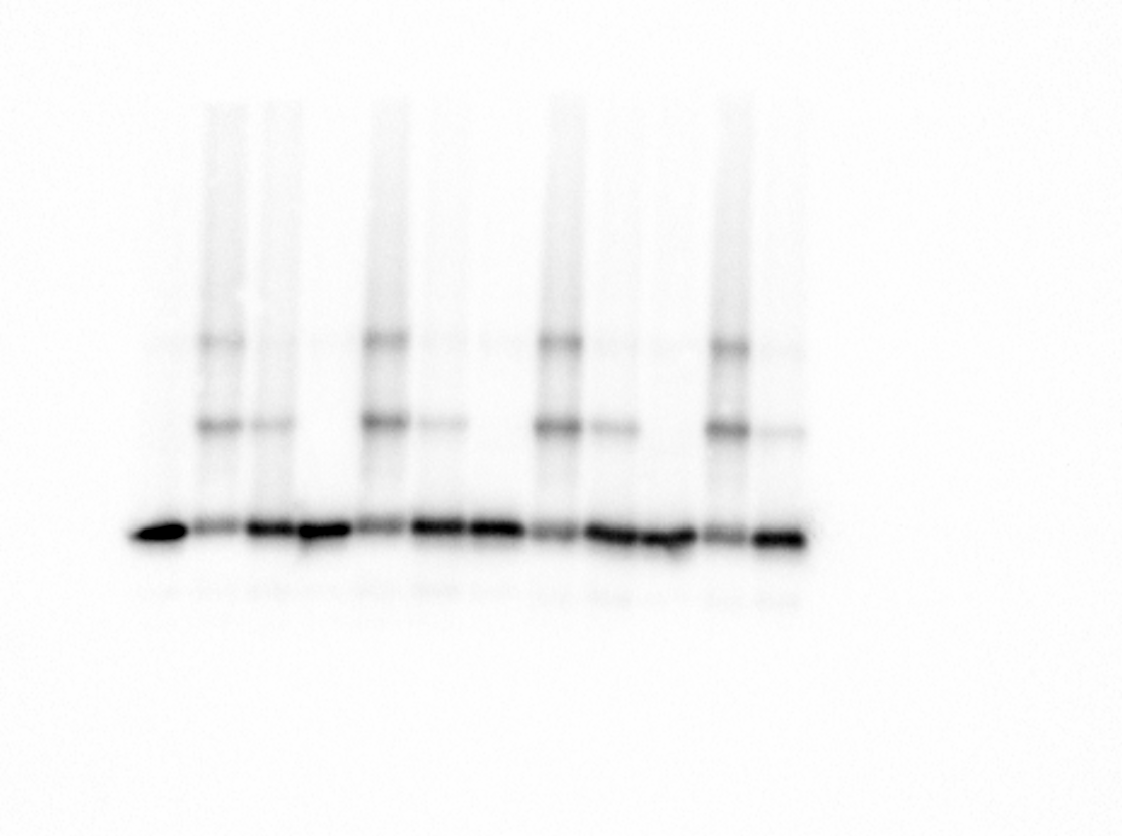

Supplement: Figure 6—figure supplement 1—source data 2. [file elife-74275-fig6-figsupp1-data2.zip › Figure 6-figure supplement 1-source data 2/figure supplement 1B/pSO132 anti-FLAG 15-60min Set1.tif]

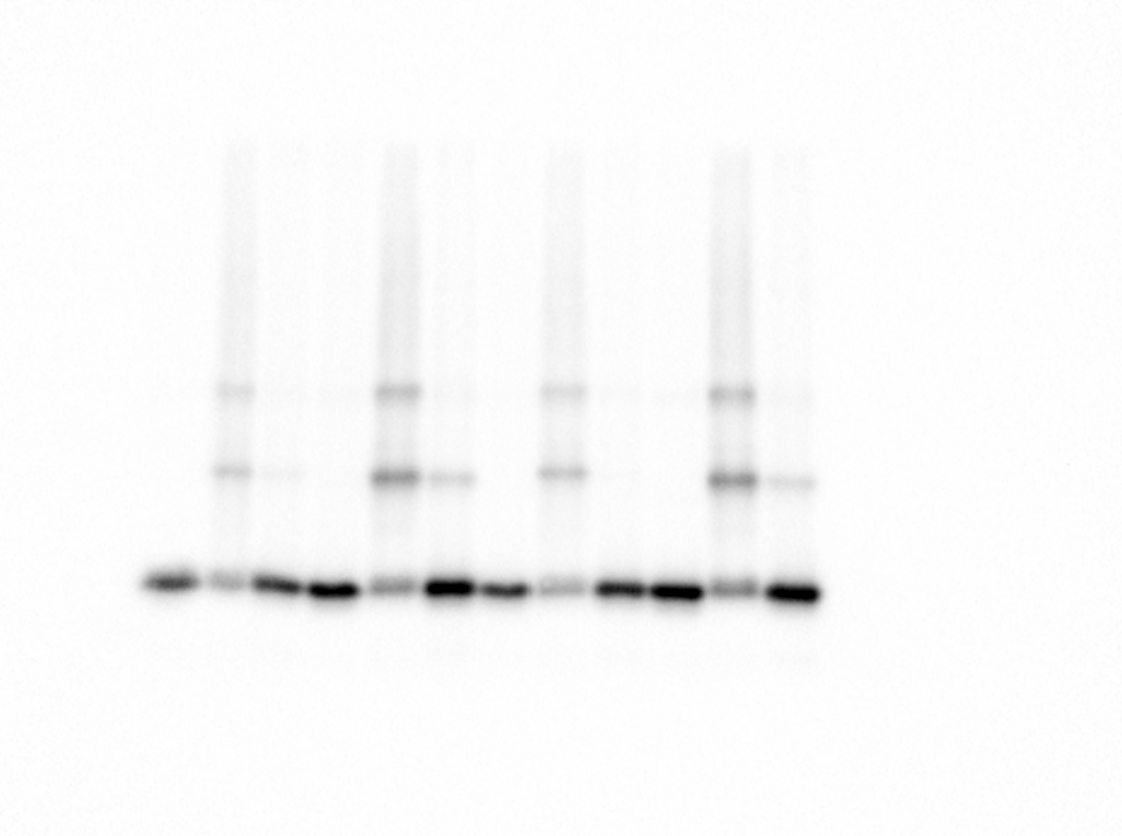

Supplement: Figure 6—figure supplement 1—source data 2. [file elife-74275-fig6-figsupp1-data2.zip › Figure 6-figure supplement 1-source data 2/figure supplement 1B/pSO132 anti-FLAG 45-60min Set2.tif]

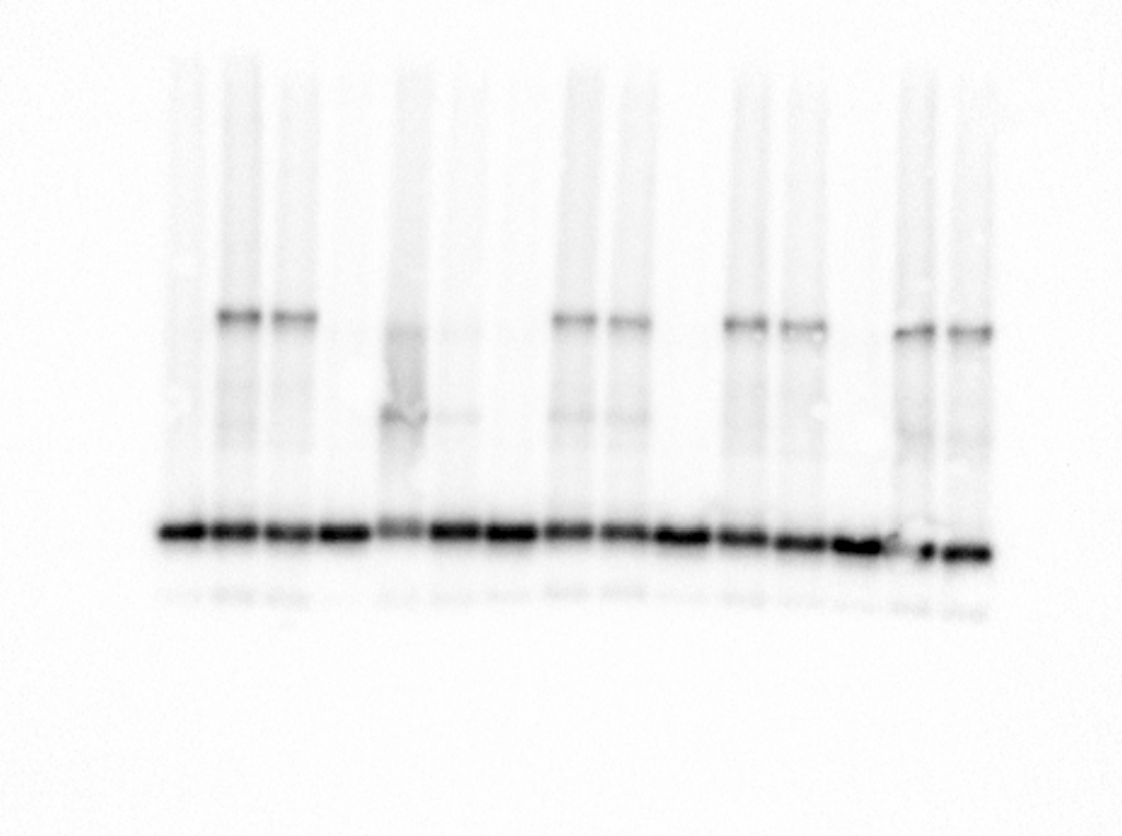

Supplement: Figure 6—figure supplement 1—source data 2. [file elife-74275-fig6-figsupp1-data2.zip › Figure 6-figure supplement 1-source data 2/figure supplement 1B/pSO163 and 165 anti-FLAG 30min Set1.tif]

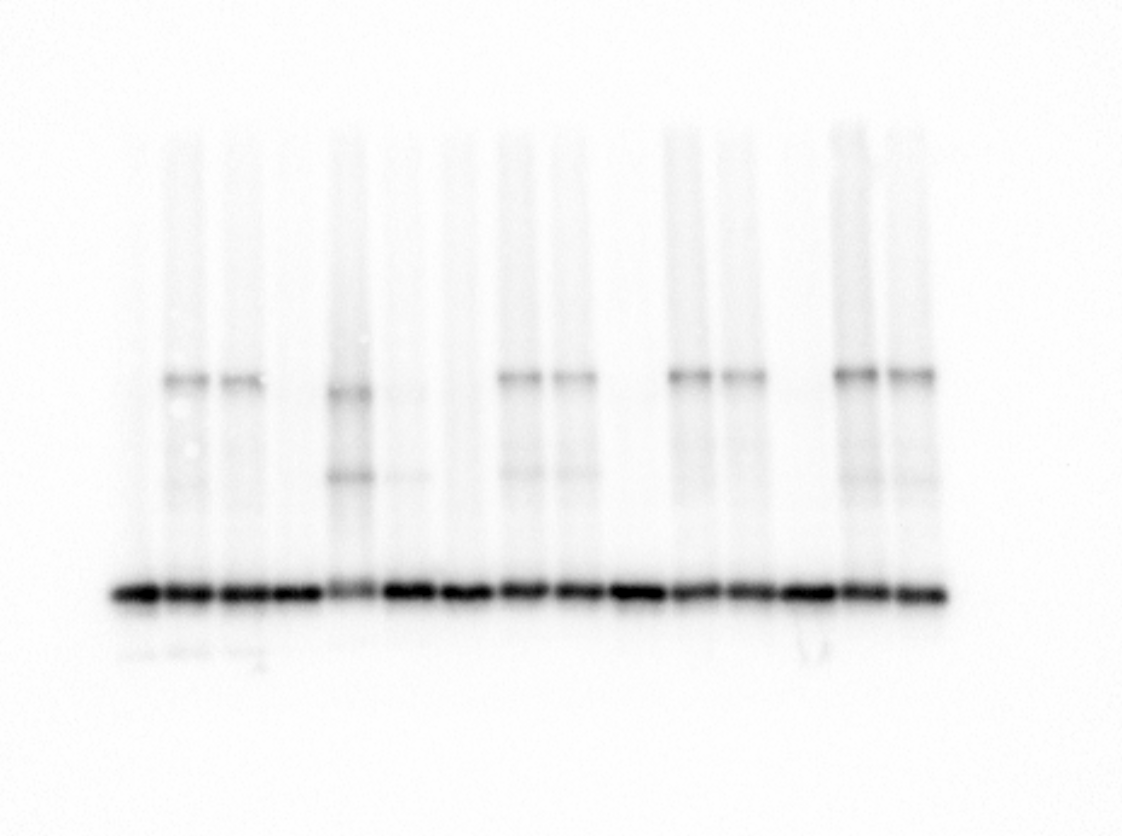

Supplement: Figure 6—figure supplement 1—source data 2. [file elife-74275-fig6-figsupp1-data2.zip › Figure 6-figure supplement 1-source data 2/figure supplement 1B/pSO163_ 164_ 165_ 166 and 131 anti-FLAG 15min Set1.tif]

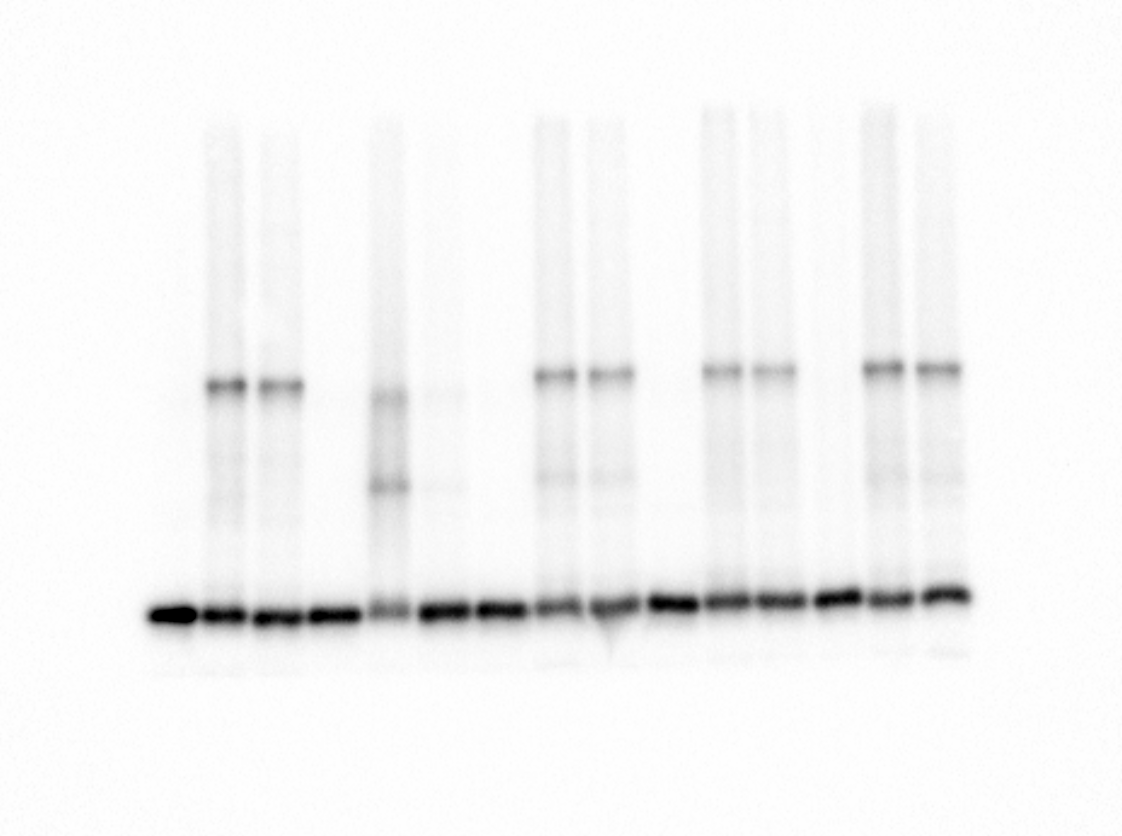

Supplement: Figure 6—figure supplement 1—source data 2. [file elife-74275-fig6-figsupp1-data2.zip › Figure 6-figure supplement 1-source data 2/figure supplement 1B/pSO163_ 164_ 165_ 166 and 131 anti-FLAG 45min Set1.tif]

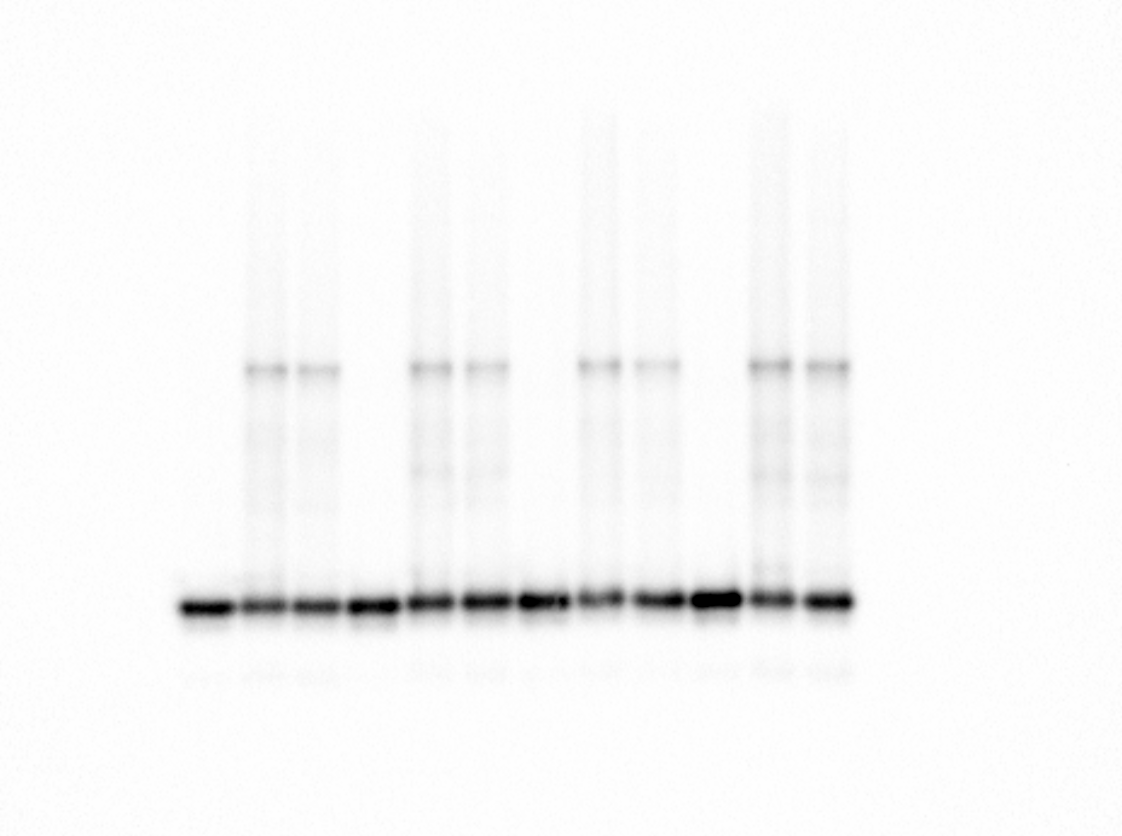

Supplement: Figure 6—figure supplement 1—source data 2. [file elife-74275-fig6-figsupp1-data2.zip › Figure 6-figure supplement 1-source data 2/figure supplement 1B/pSO163_ 165_ 166_ 131 anti-FLAG 30min Set2.tif]

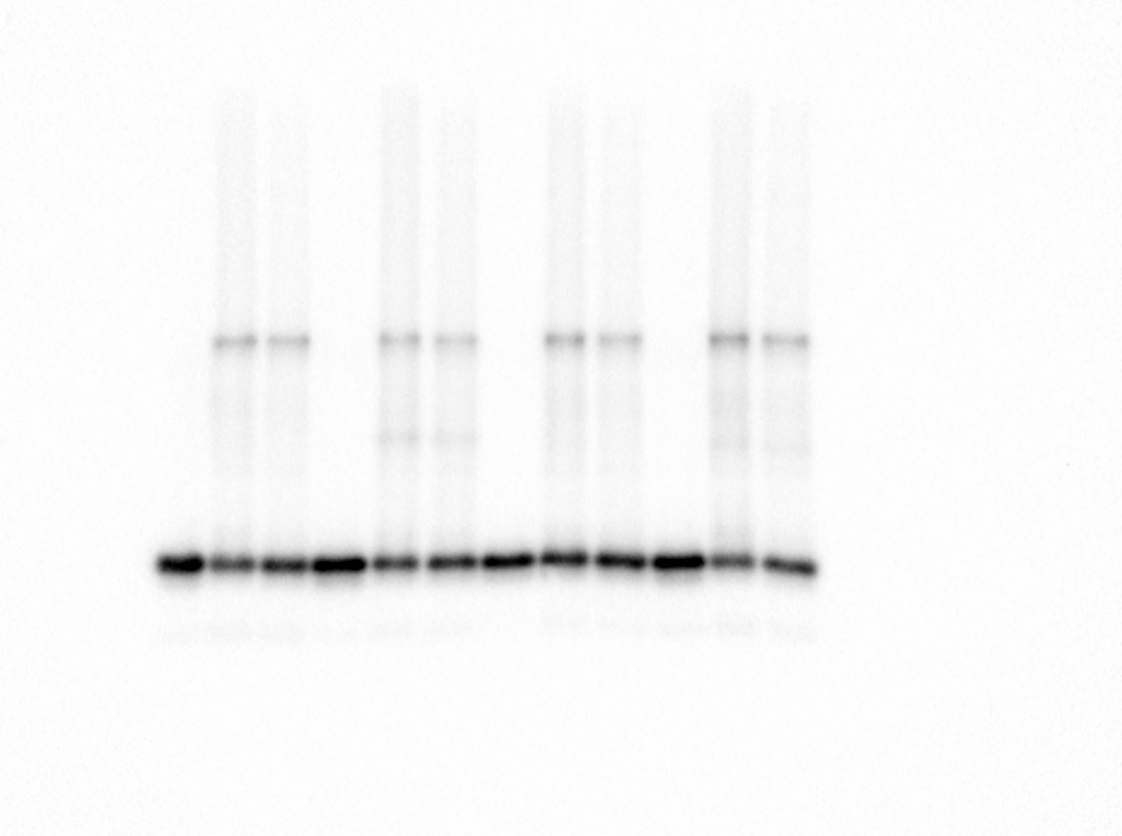

Supplement: Figure 6—figure supplement 1—source data 2. [file elife-74275-fig6-figsupp1-data2.zip › Figure 6-figure supplement 1-source data 2/figure supplement 1B/pSO163_ 165_ 166_ 131 anti-FLAG 45min Set2.tif]

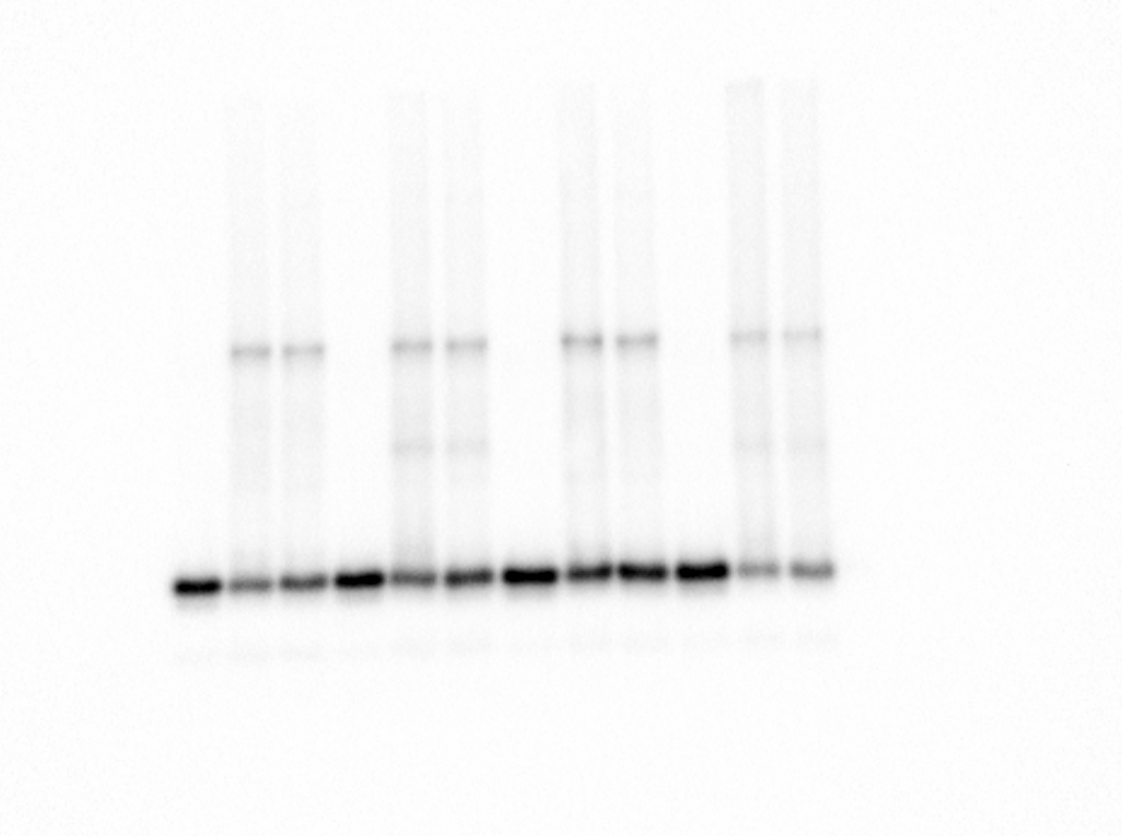

Supplement: Figure 6—figure supplement 1—source data 2. [file elife-74275-fig6-figsupp1-data2.zip › Figure 6-figure supplement 1-source data 2/figure supplement 1B/pSO163_ 165_ 166_ 131 anti-FLAG 60min Set2.tif]

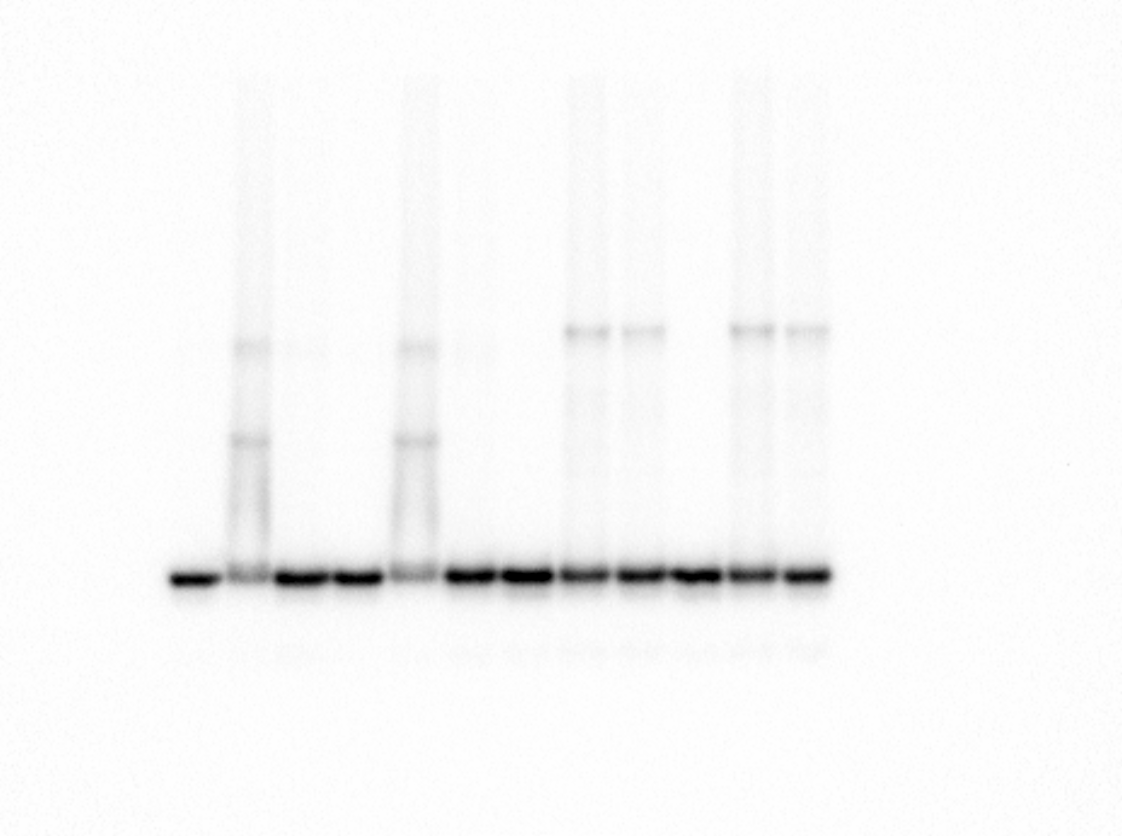

Supplement: Figure 6—figure supplement 1—source data 2. [file elife-74275-fig6-figsupp1-data2.zip › Figure 6-figure supplement 1-source data 2/figure supplement 1B/pSO164 and 163 15min anti-FLAG Set2.tif]

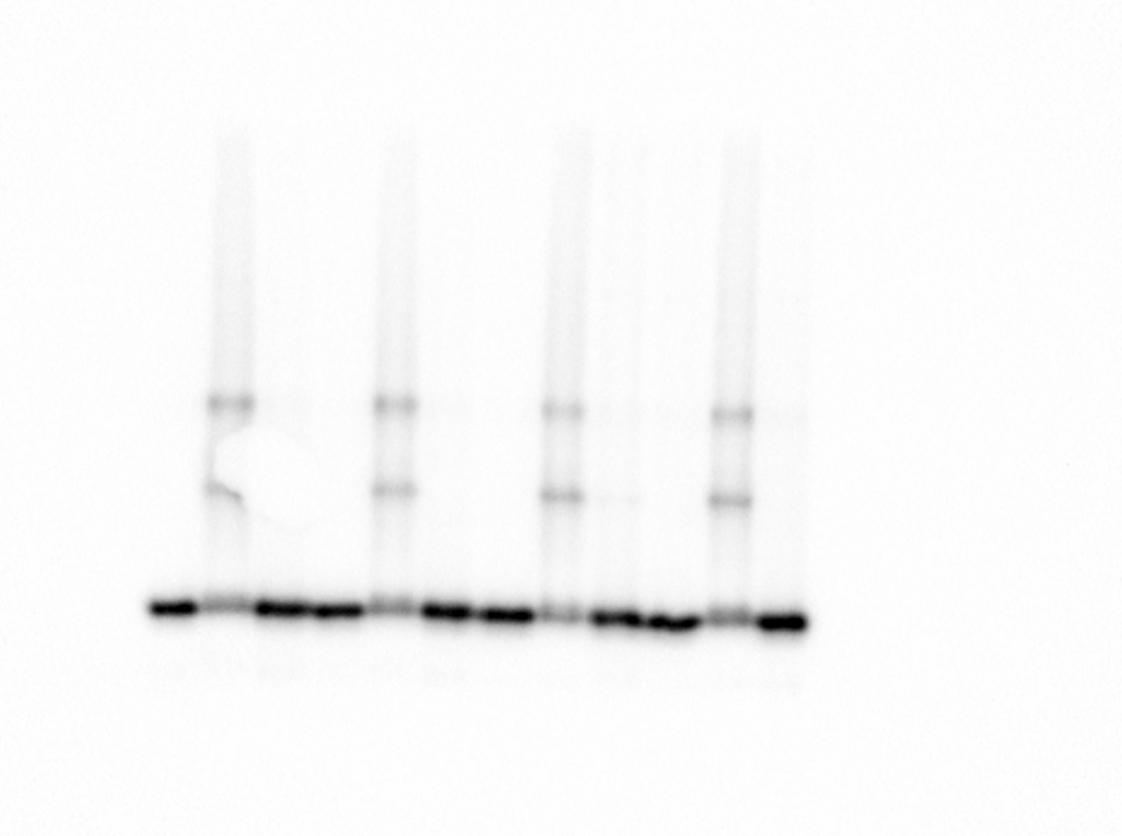

Supplement: Figure 6—figure supplement 1—source data 2. [file elife-74275-fig6-figsupp1-data2.zip › Figure 6-figure supplement 1-source data 2/figure supplement 1B/pSO164 anti-FLAG 30-60min Set2.tif]

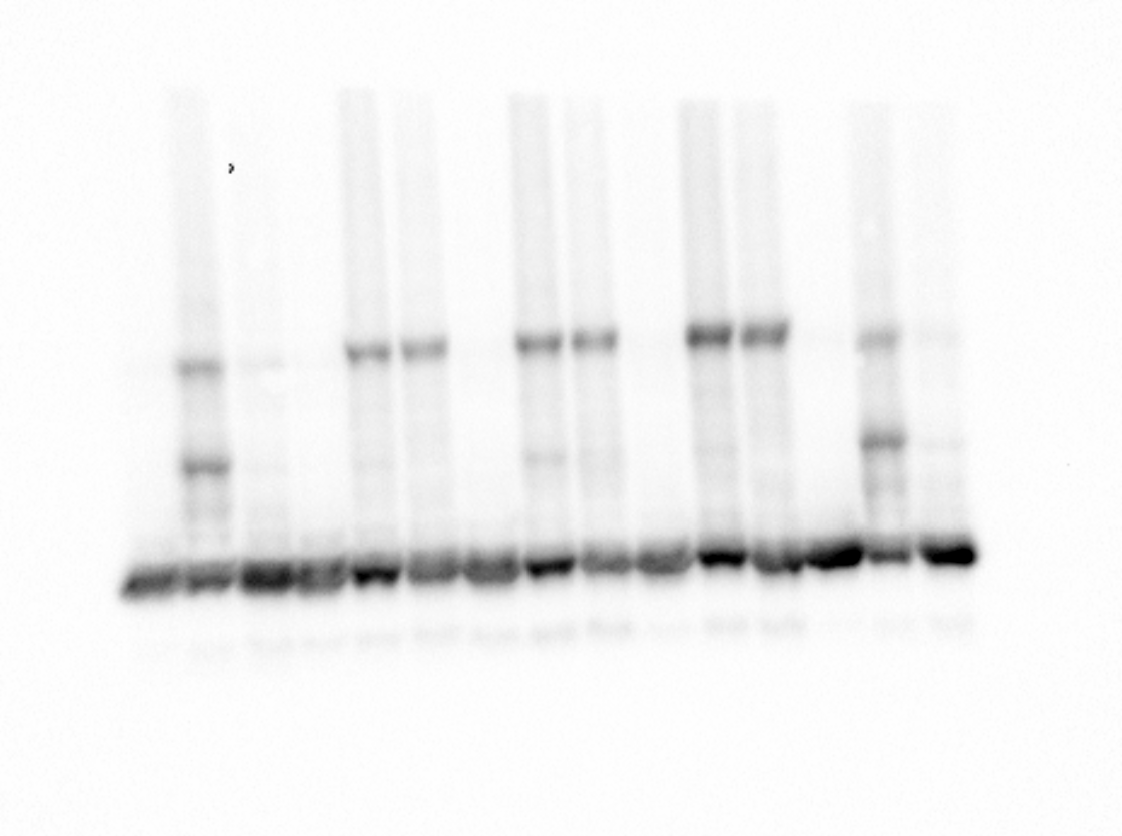

Supplement: Figure 6—figure supplement 1—source data 2. [file elife-74275-fig6-figsupp1-data2.zip › Figure 6-figure supplement 1-source data 2/figure supplement 1B/pSO164_ 166_ 131 30min and 163_ 164 60 min anti-FLAG Set1.tif]

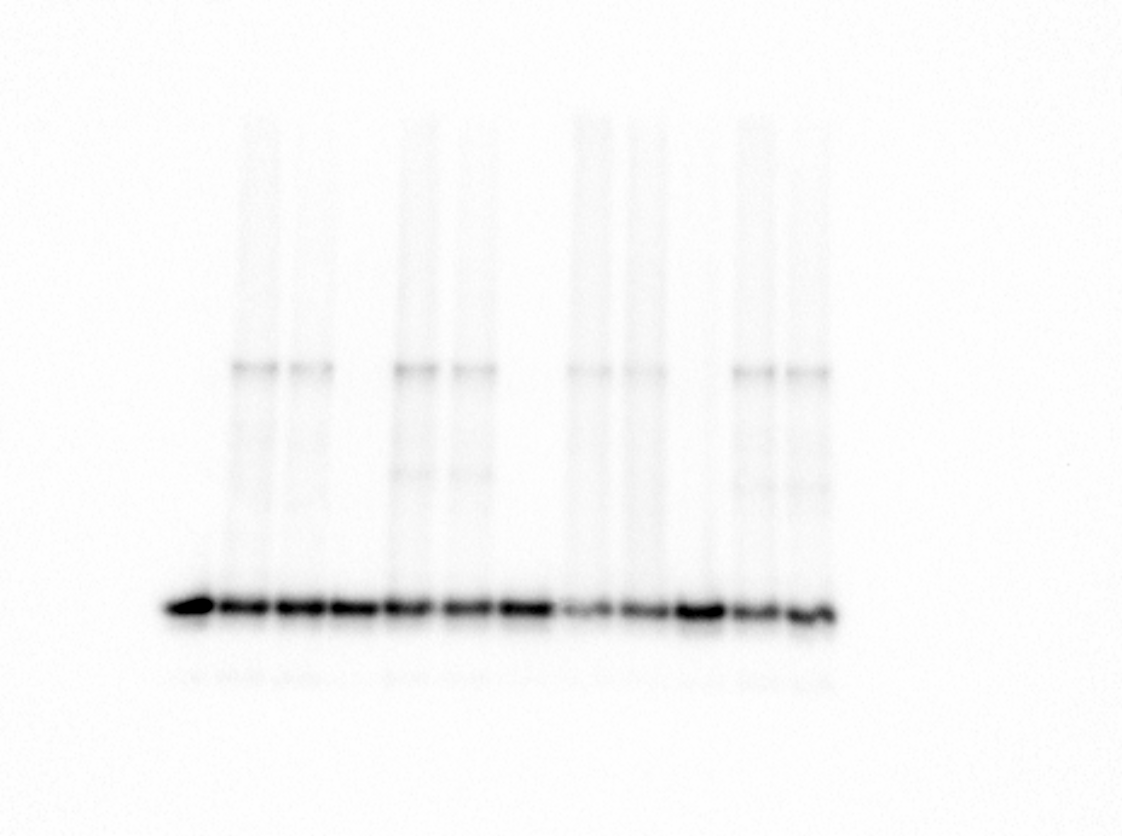

Supplement: Figure 6—figure supplement 1—source data 2. [file elife-74275-fig6-figsupp1-data2.zip › Figure 6-figure supplement 1-source data 2/figure supplement 1B/pSO165 and 166 anti-FLAG 15min Set2.tif]

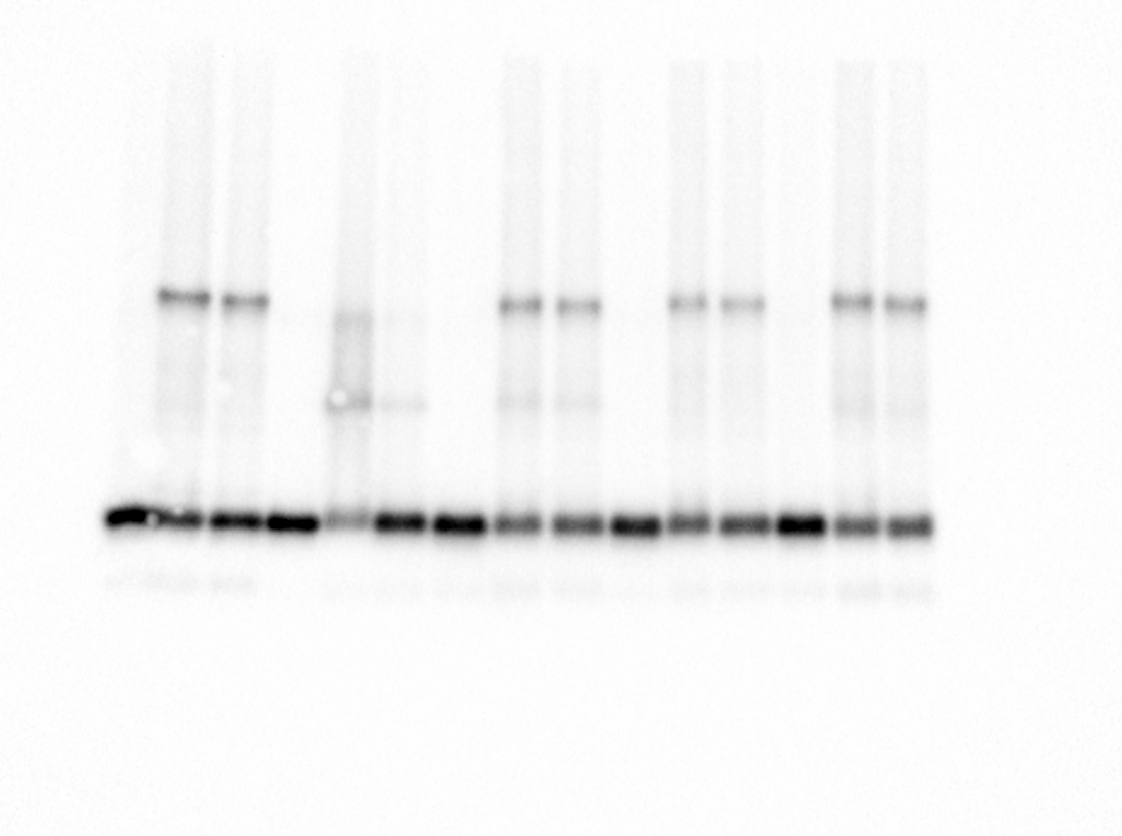

Supplement: Figure 6—figure supplement 1—source data 2. [file elife-74275-fig6-figsupp1-data2.zip › Figure 6-figure supplement 1-source data 2/figure supplement 1B/pSO165_ 166_ 131 anti-FLAG 60min Set1.tif]

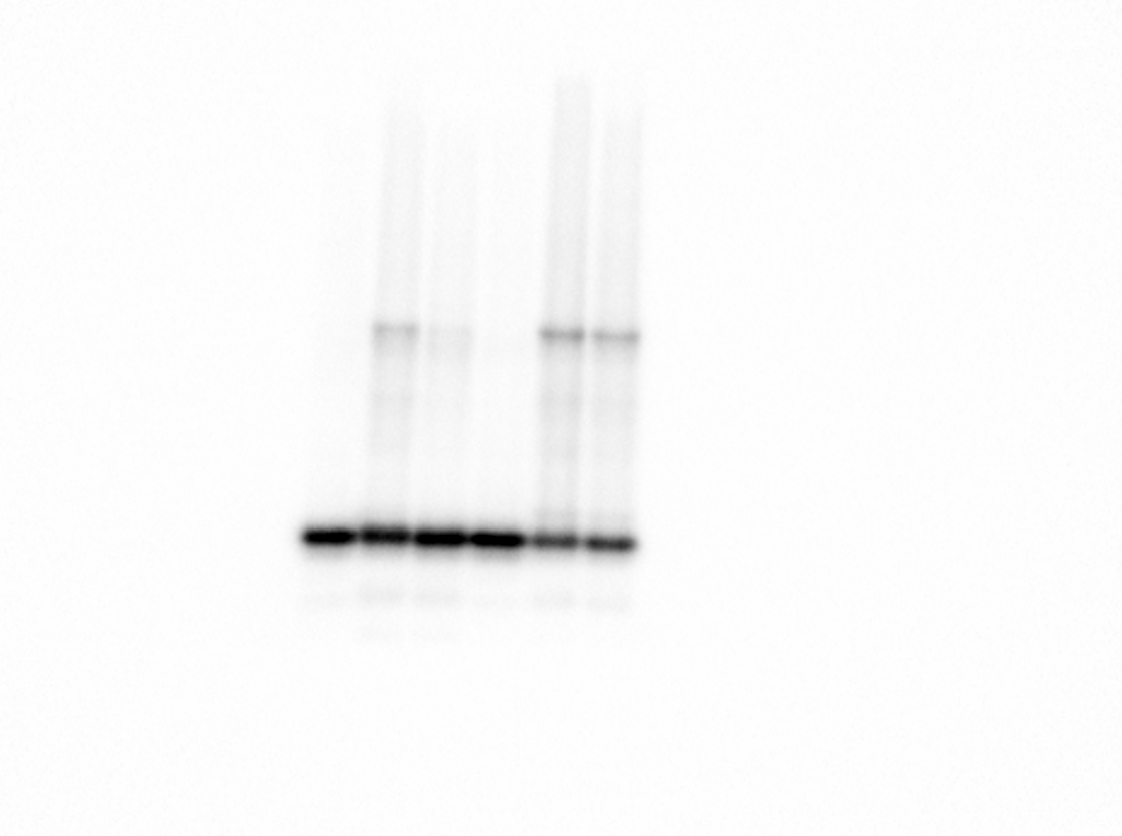

Supplement: Figure 6—figure supplement 2—source data 1. [file elife-74275-fig6-figsupp2-data1.zip › Figure 6-figure supplement 2-source data 1/figure supplement 2A/pSO229 15 and 60min anti-FLAG Set2.tif]

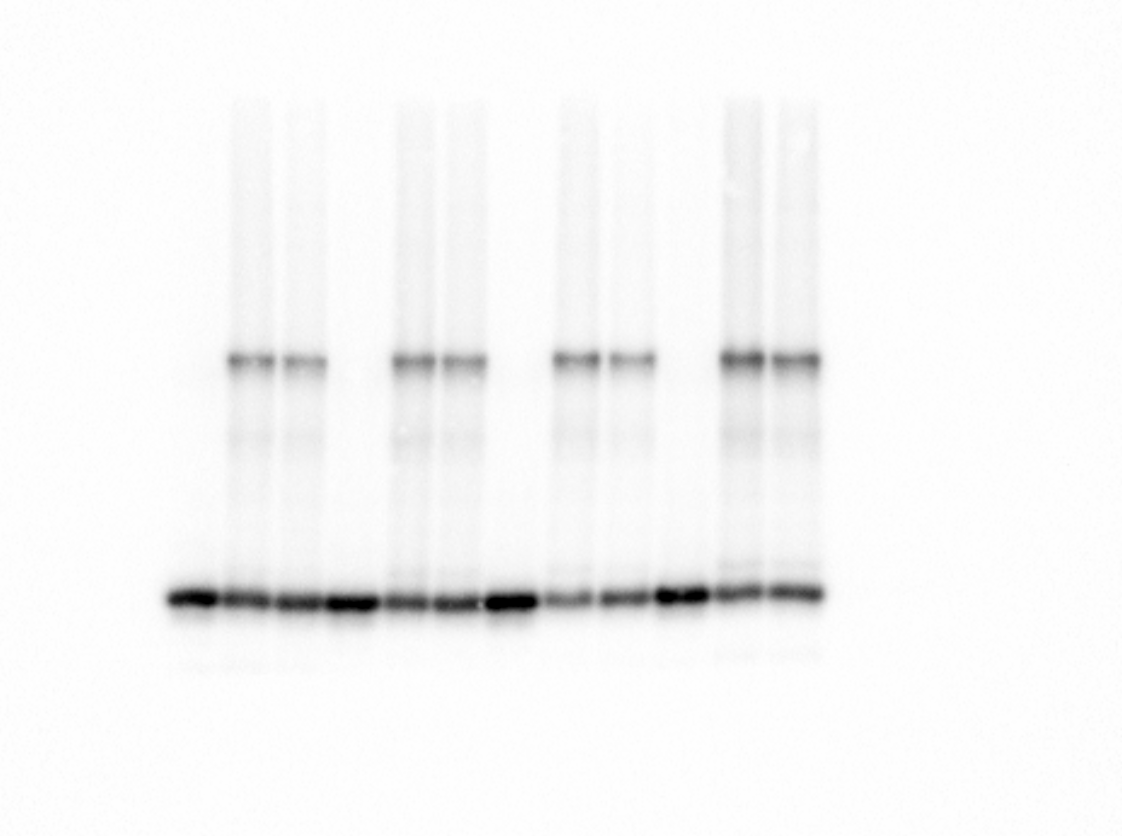

Supplement: Figure 6—figure supplement 2—source data 1. [file elife-74275-fig6-figsupp2-data1.zip › Figure 6-figure supplement 2-source data 1/figure supplement 2A/pSO229 15-60min anti-FLAG Set1.tif]

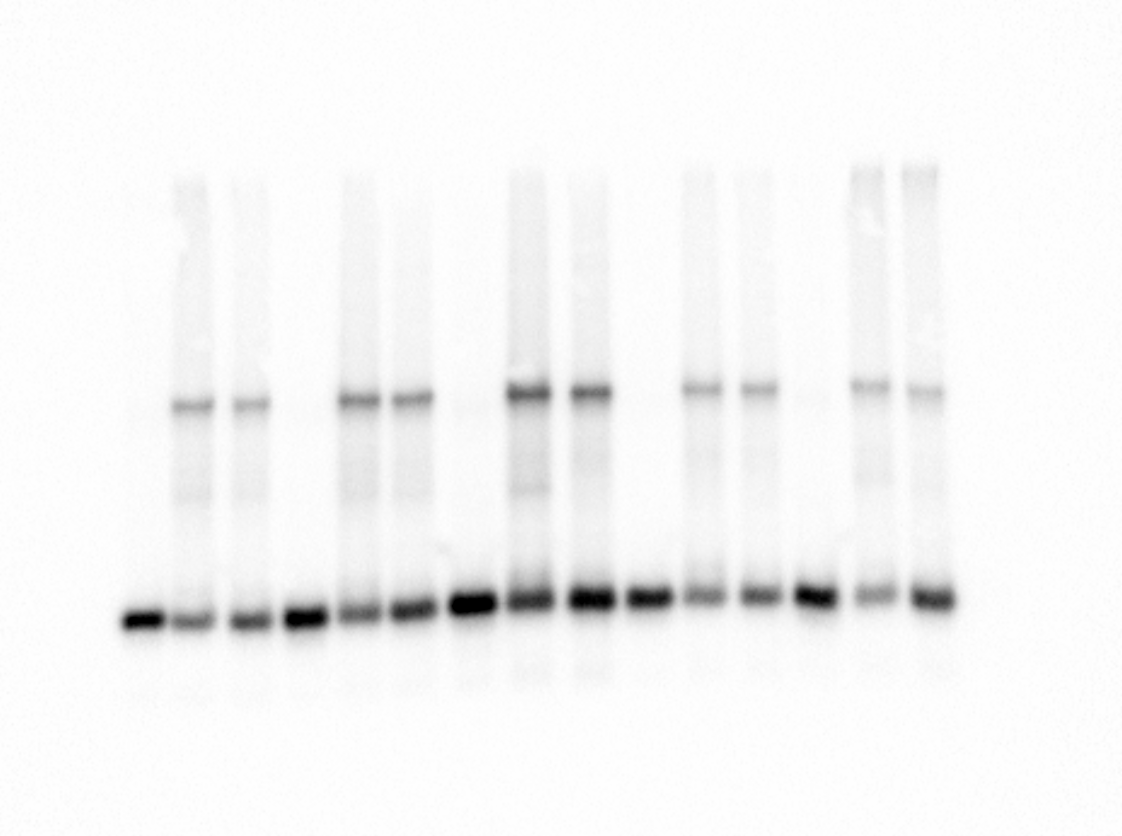

Supplement: Figure 6—figure supplement 2—source data 1. [file elife-74275-fig6-figsupp2-data1.zip › Figure 6-figure supplement 2-source data 1/figure supplement 2A/pSO234 and 239 45min and 238_ 235 and 239 60min anti-FLAG Set1.tif]

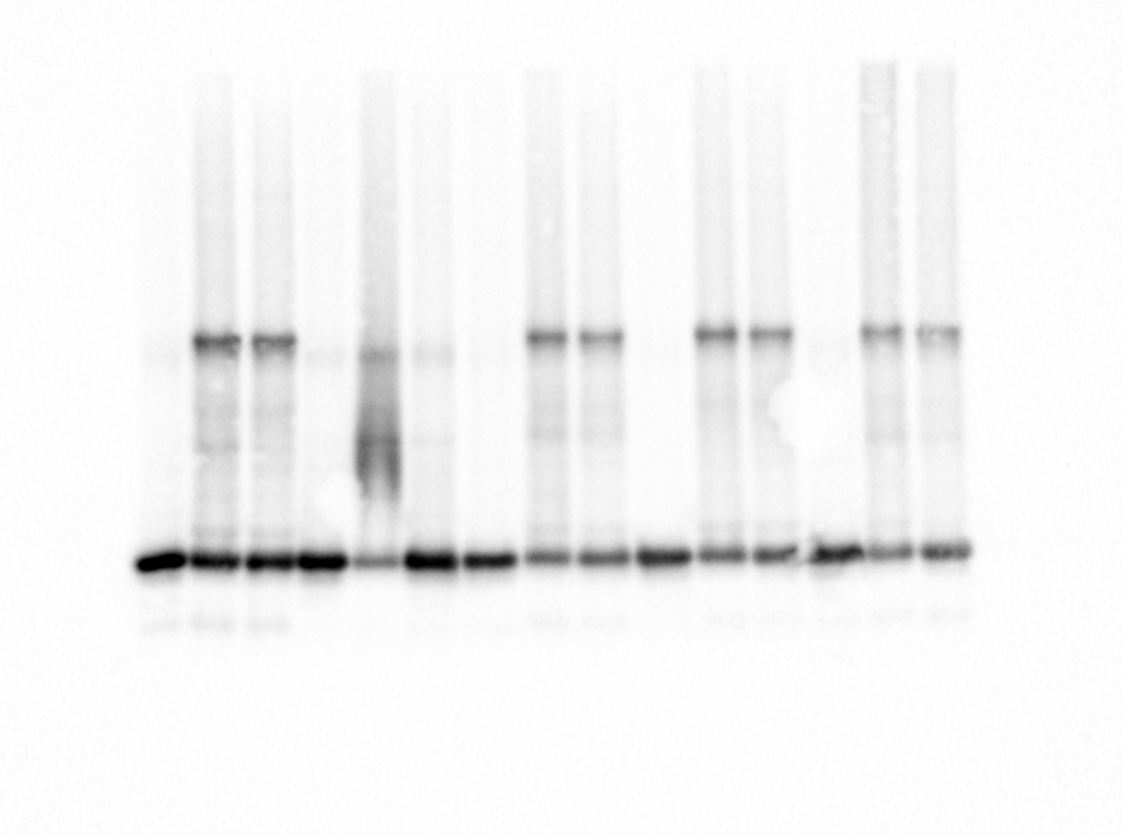

Supplement: Figure 6—figure supplement 2—source data 1. [file elife-74275-fig6-figsupp2-data1.zip › Figure 6-figure supplement 2-source data 1/figure supplement 2A/pSO234 anti-FLAG 60min Set1.tif]

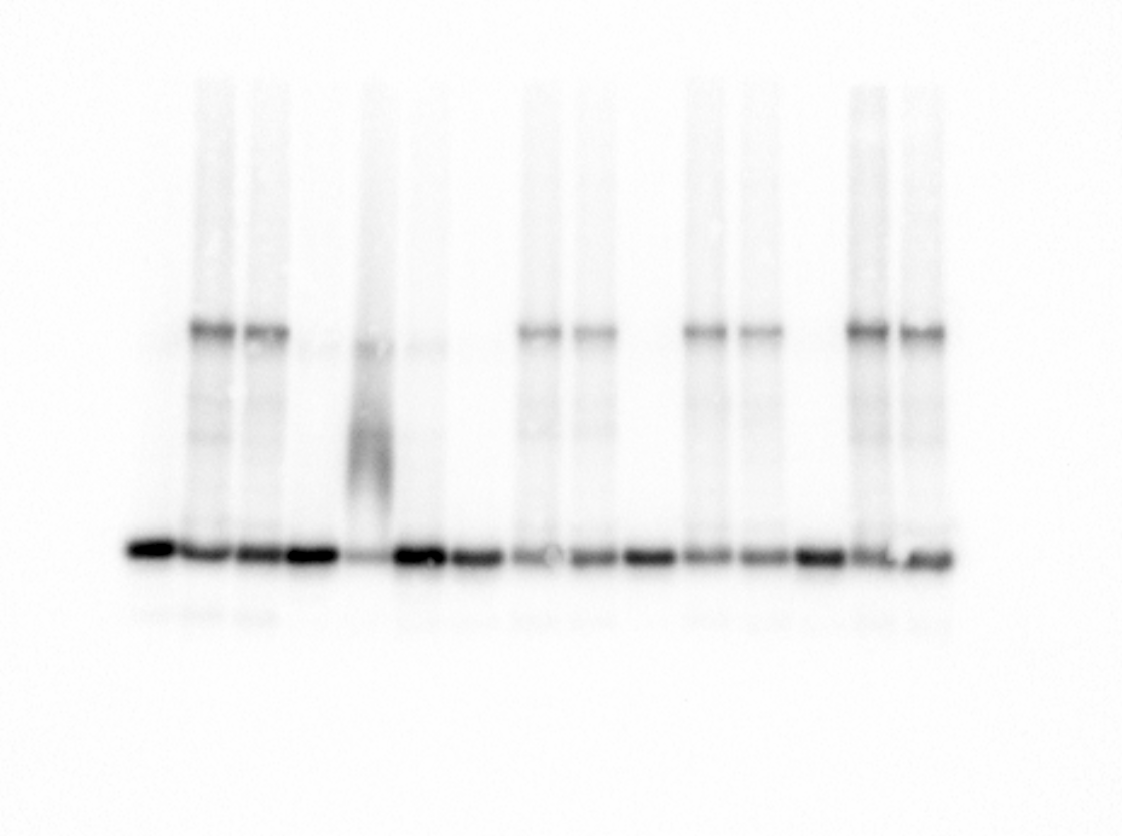

Supplement: Figure 6—figure supplement 2—source data 1. [file elife-74275-fig6-figsupp2-data1.zip › Figure 6-figure supplement 2-source data 1/figure supplement 2A/pSO238 and 235 anti-FLAG 45min Set1.tif]

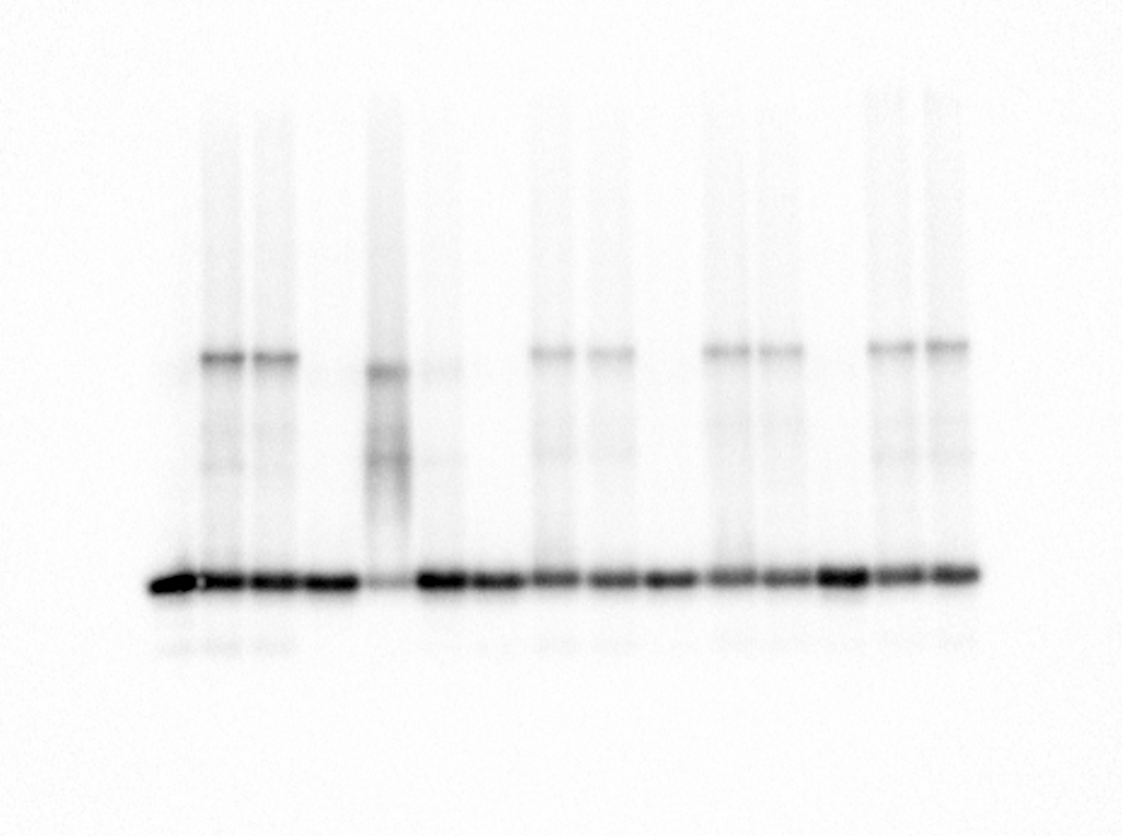

Supplement: Figure 6—figure supplement 2—source data 1. [file elife-74275-fig6-figsupp2-data1.zip › Figure 6-figure supplement 2-source data 1/figure supplement 2A/pSO238_ 234_ 235_ 239 anti-FLAG 15min Set1.tif]

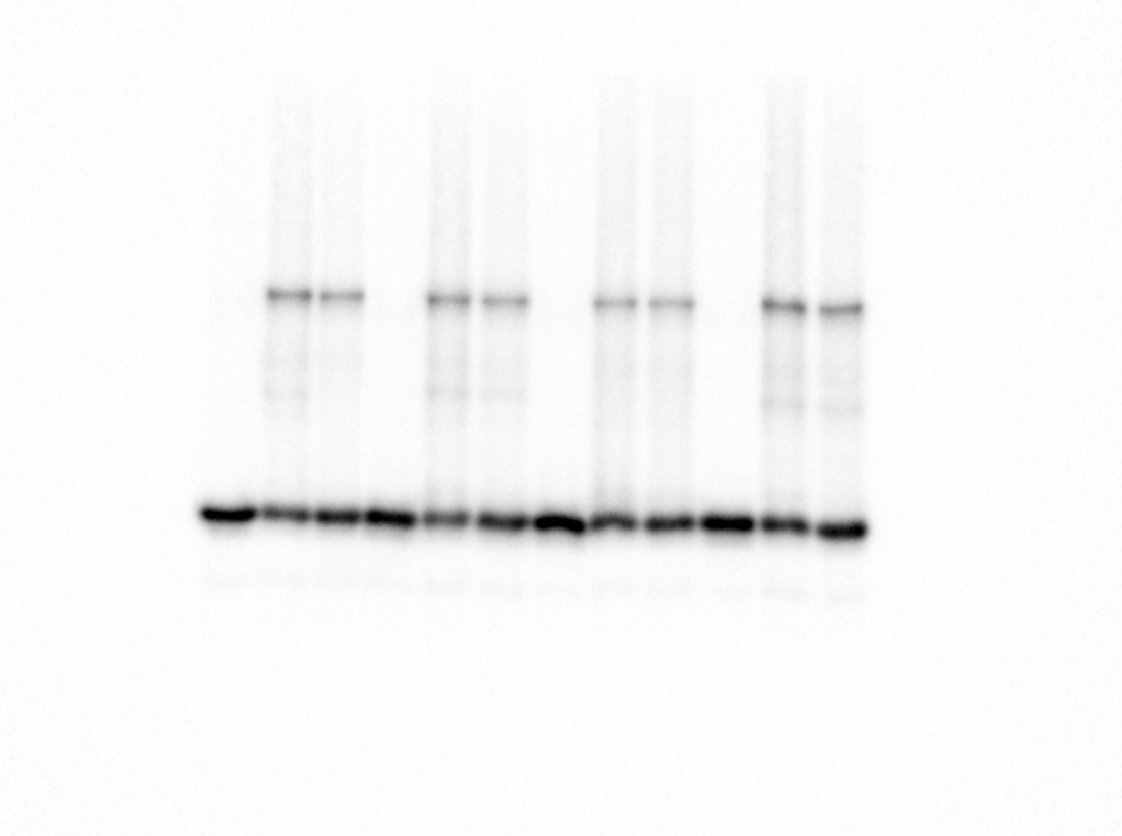

Supplement: Figure 6—figure supplement 2—source data 1. [file elife-74275-fig6-figsupp2-data1.zip › Figure 6-figure supplement 2-source data 1/figure supplement 2A/pSO238_ 234_ 235_ 239 anti-FLAG 15min Set2.tif]

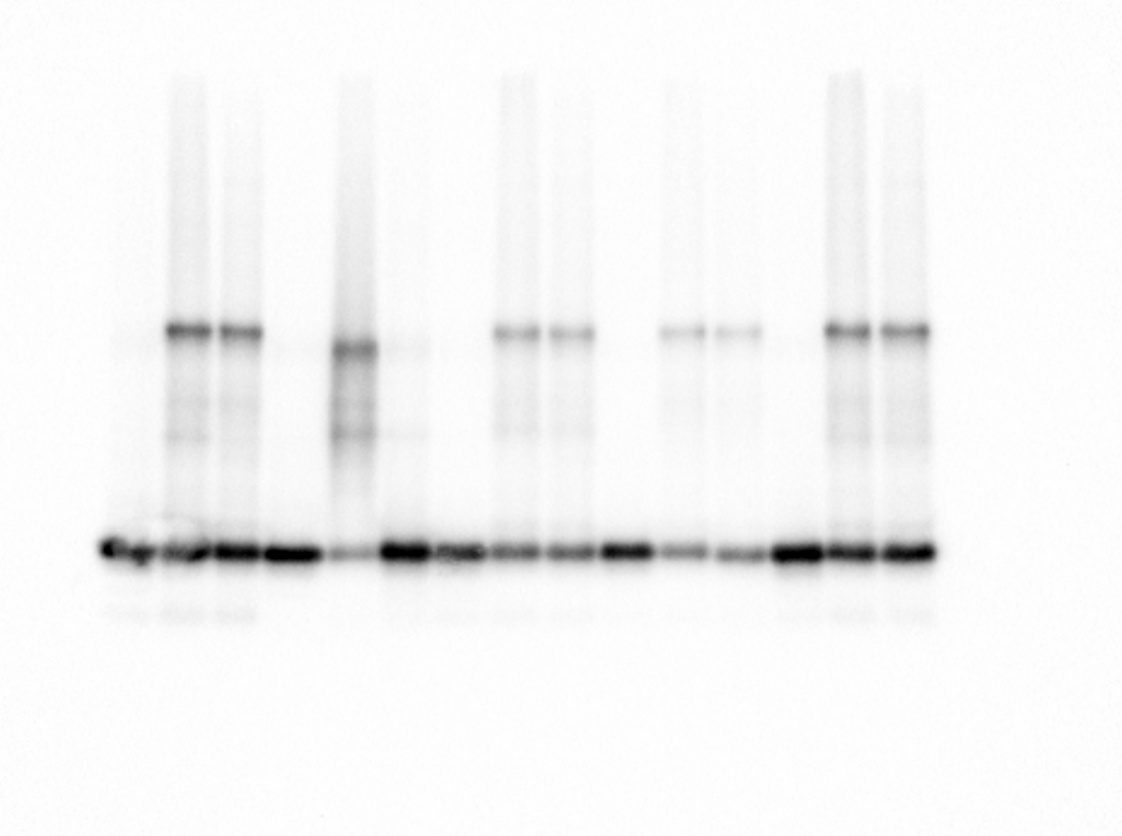

Supplement: Figure 6—figure supplement 2—source data 1. [file elife-74275-fig6-figsupp2-data1.zip › Figure 6-figure supplement 2-source data 1/figure supplement 2A/pSO238_ 234_ 235_ 239 anti-FLAG 30min Set1.tif]

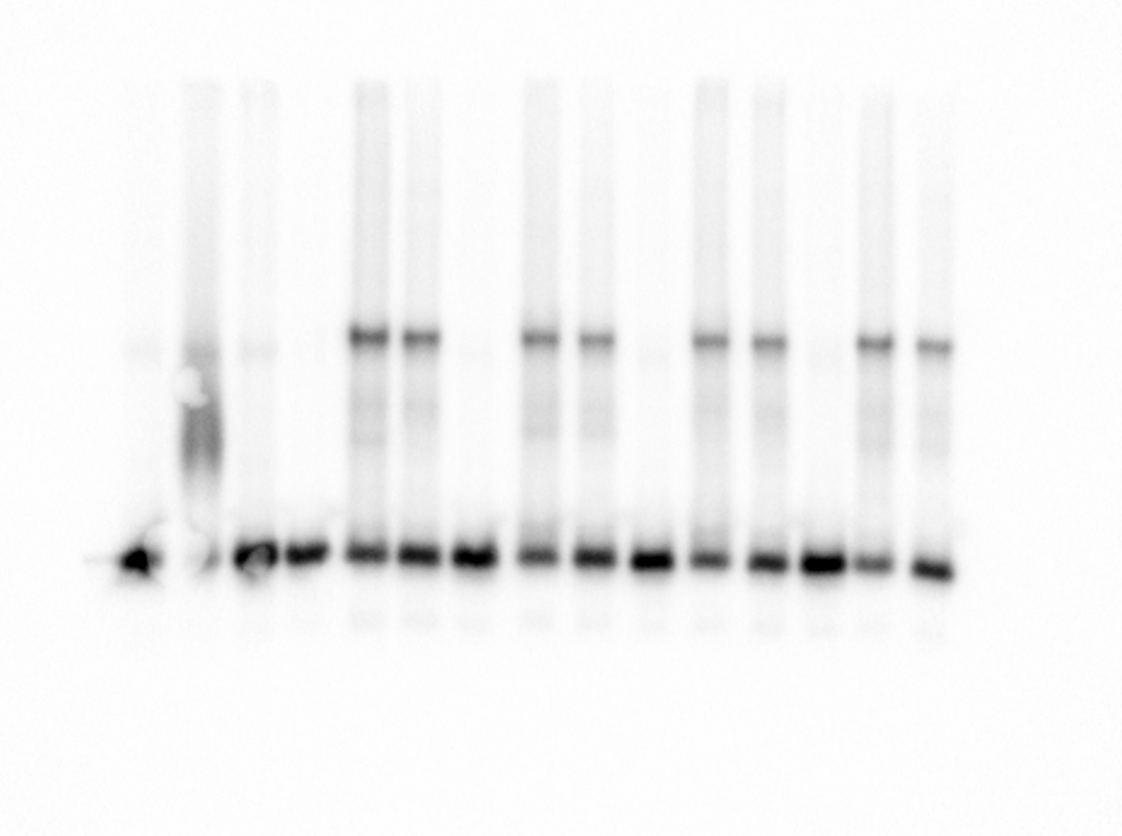

Supplement: Figure 6—figure supplement 2—source data 1. [file elife-74275-fig6-figsupp2-data1.zip › Figure 6-figure supplement 2-source data 1/figure supplement 2A/pSO238_ 234_ 235_ 239 anti-FLAG 30min Set2.tif]

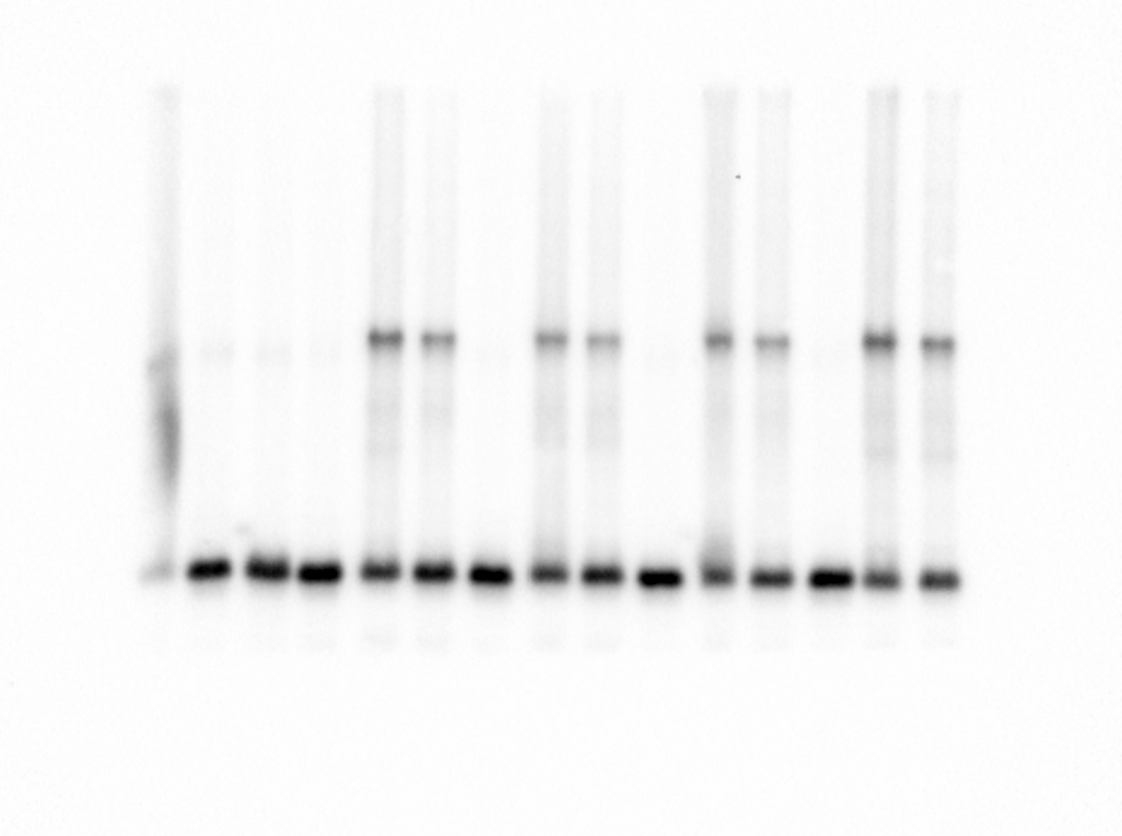

Supplement: Figure 6—figure supplement 2—source data 1. [file elife-74275-fig6-figsupp2-data1.zip › Figure 6-figure supplement 2-source data 1/figure supplement 2A/pSO238_ 234_ 235_ 239 anti-FLAG 45min Set2.tif]

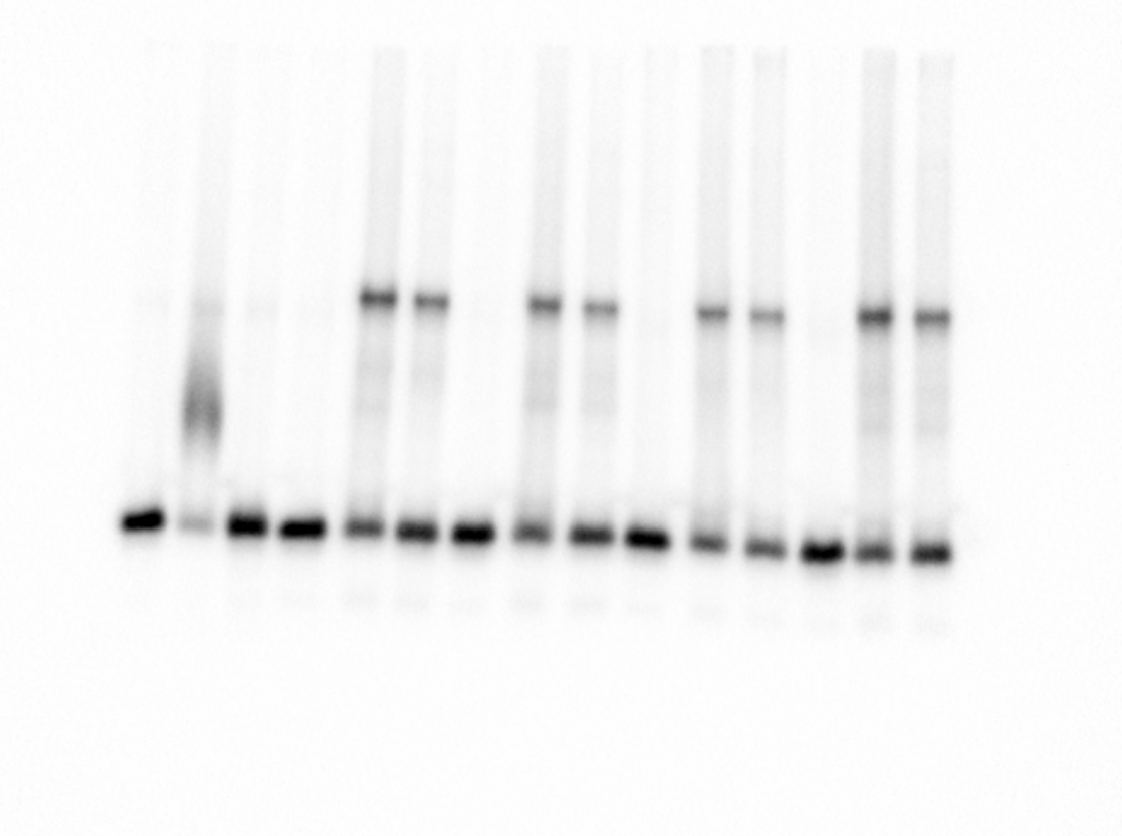

Supplement: Figure 6—figure supplement 2—source data 1. [file elife-74275-fig6-figsupp2-data1.zip › Figure 6-figure supplement 2-source data 1/figure supplement 2A/pSO238_ 234_ 235_ 239 anti-FLAG 60min Set2.tif]

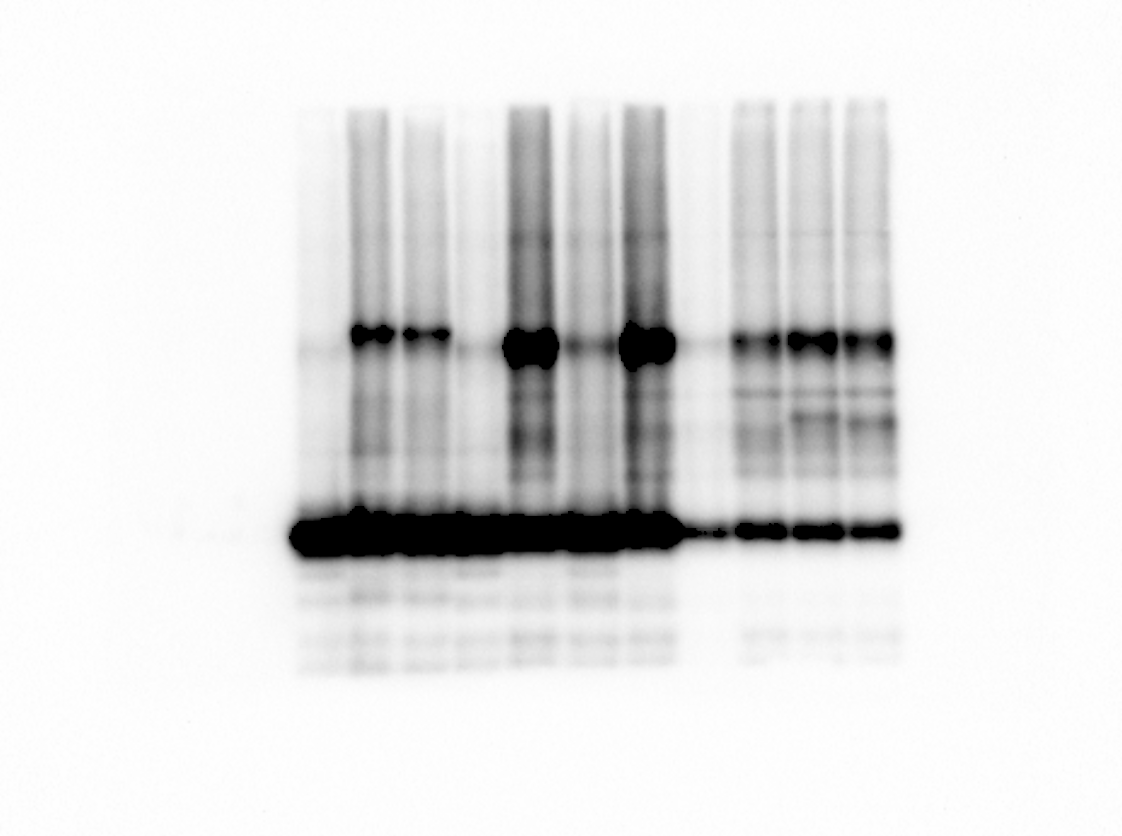

Supplement: Figure 6—figure supplement 3—source data 1. [file elife-74275-fig6-figsupp3-data1.zip › Figure 6-figure supplement 3-source data 1/fig sup 3G anti-FLAG long exposure.tif]

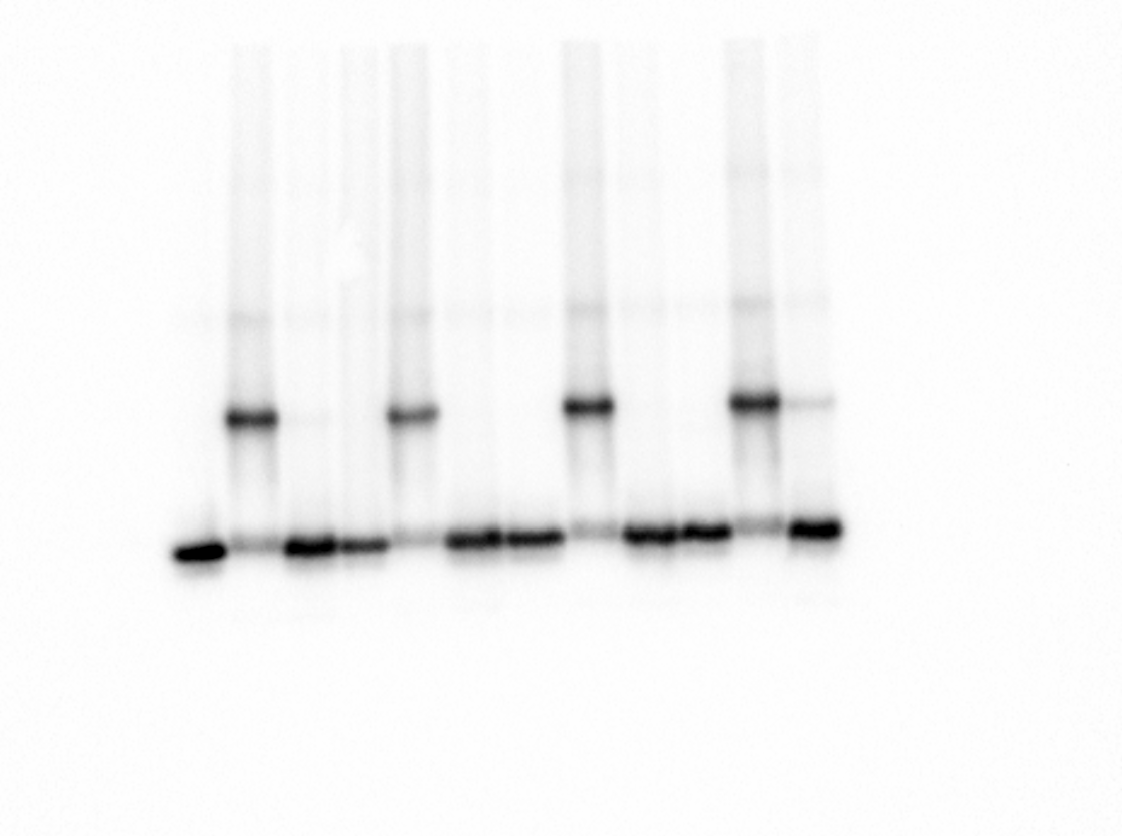

Supplement: Figure 6—figure supplement 3—source data 1. [file elife-74275-fig6-figsupp3-data1.zip › Figure 6-figure supplement 3-source data 1/figure supplement 3A/pSO134 anti-FLAG 15-60min Set1.tif]

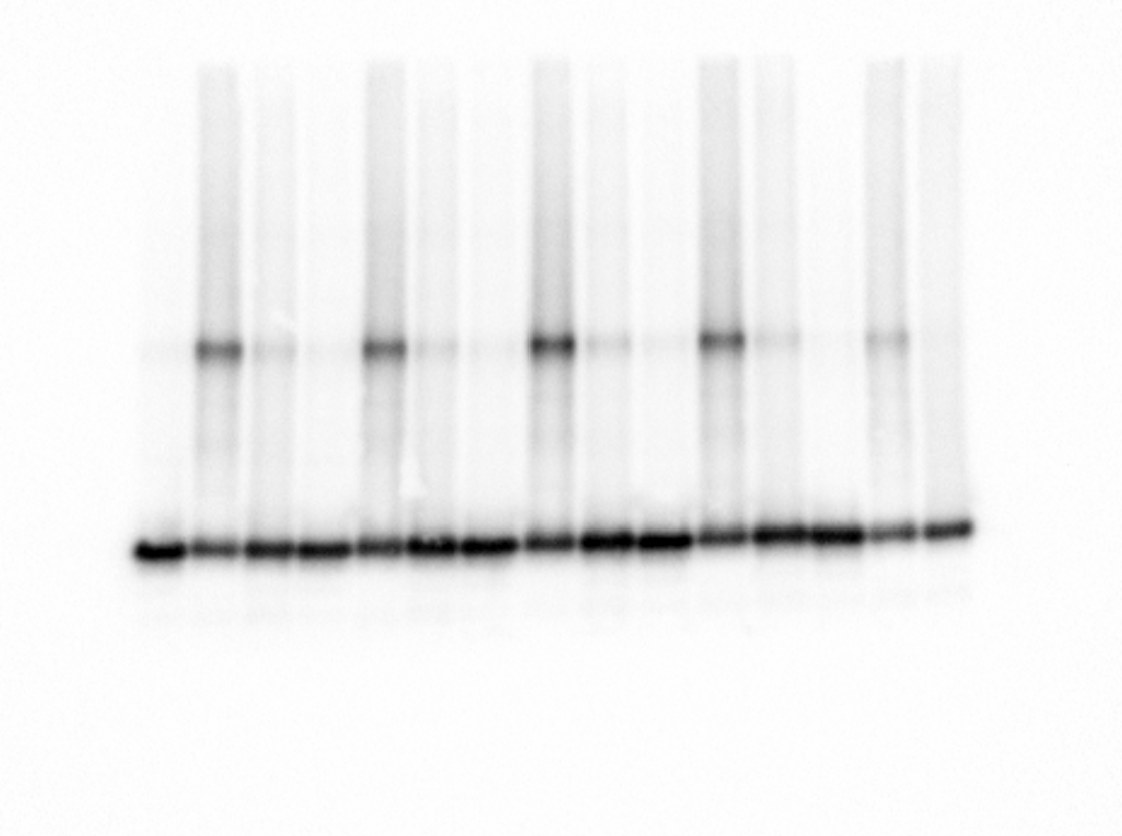

Supplement: Figure 6—figure supplement 3—source data 1. [file elife-74275-fig6-figsupp3-data1.zip › Figure 6-figure supplement 3-source data 1/figure supplement 3A/pSO136 15, 45 and 60min anti-FLAG Set2.tif]

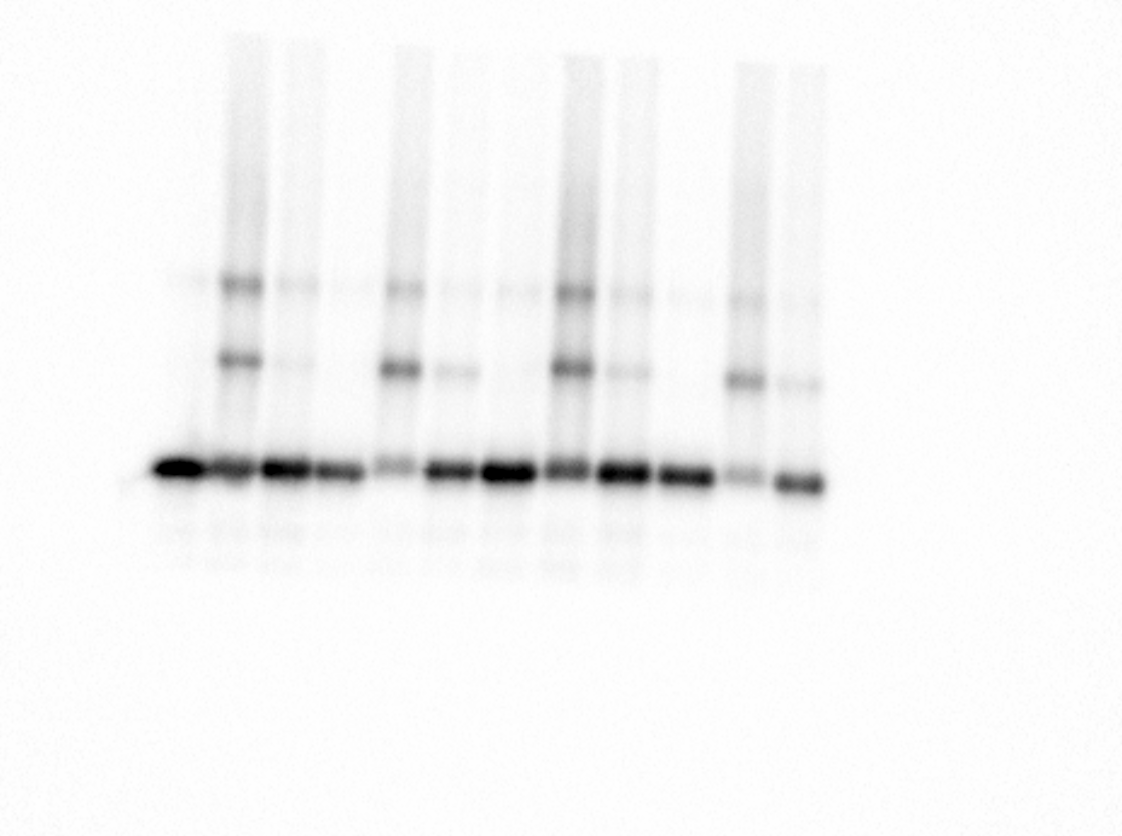

Supplement: Figure 6—figure supplement 3—source data 1. [file elife-74275-fig6-figsupp3-data1.zip › Figure 6-figure supplement 3-source data 1/figure supplement 3A/pSO168 and 134 15-30 min anti-FLAG Set2.tif]

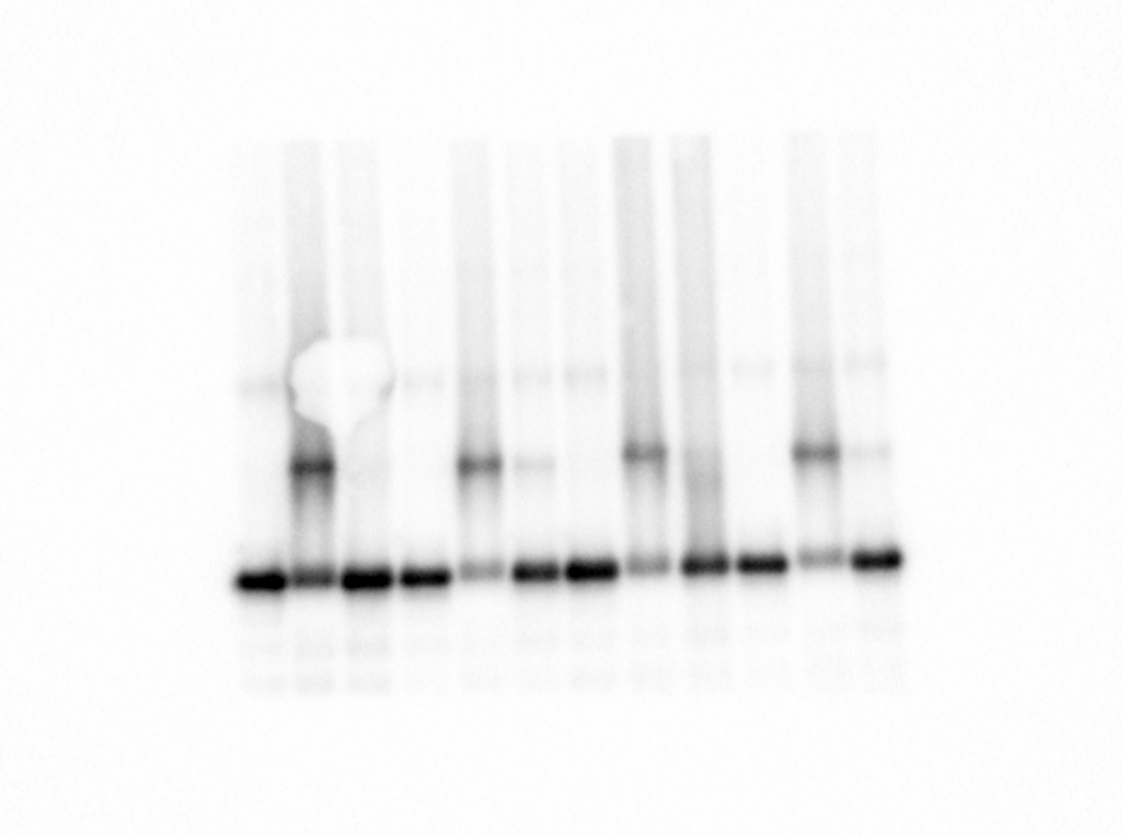

Supplement: Figure 6—figure supplement 3—source data 1. [file elife-74275-fig6-figsupp3-data1.zip › Figure 6-figure supplement 3-source data 1/figure supplement 3A/pSO168 and 134 anti-FLAG 60min Set2.tif]

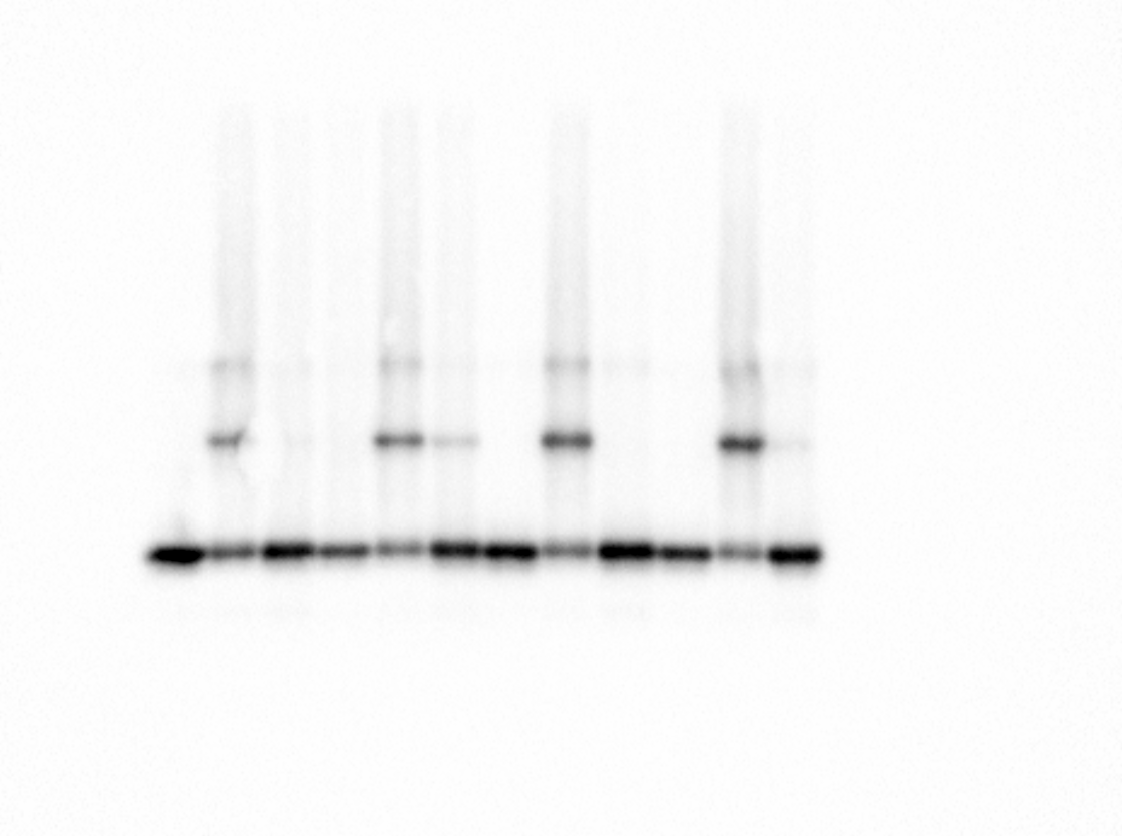

Supplement: Figure 6—figure supplement 3—source data 1. [file elife-74275-fig6-figsupp3-data1.zip › Figure 6-figure supplement 3-source data 1/figure supplement 3A/pSO168 anti-FLAG 30-60min Set1.tif]

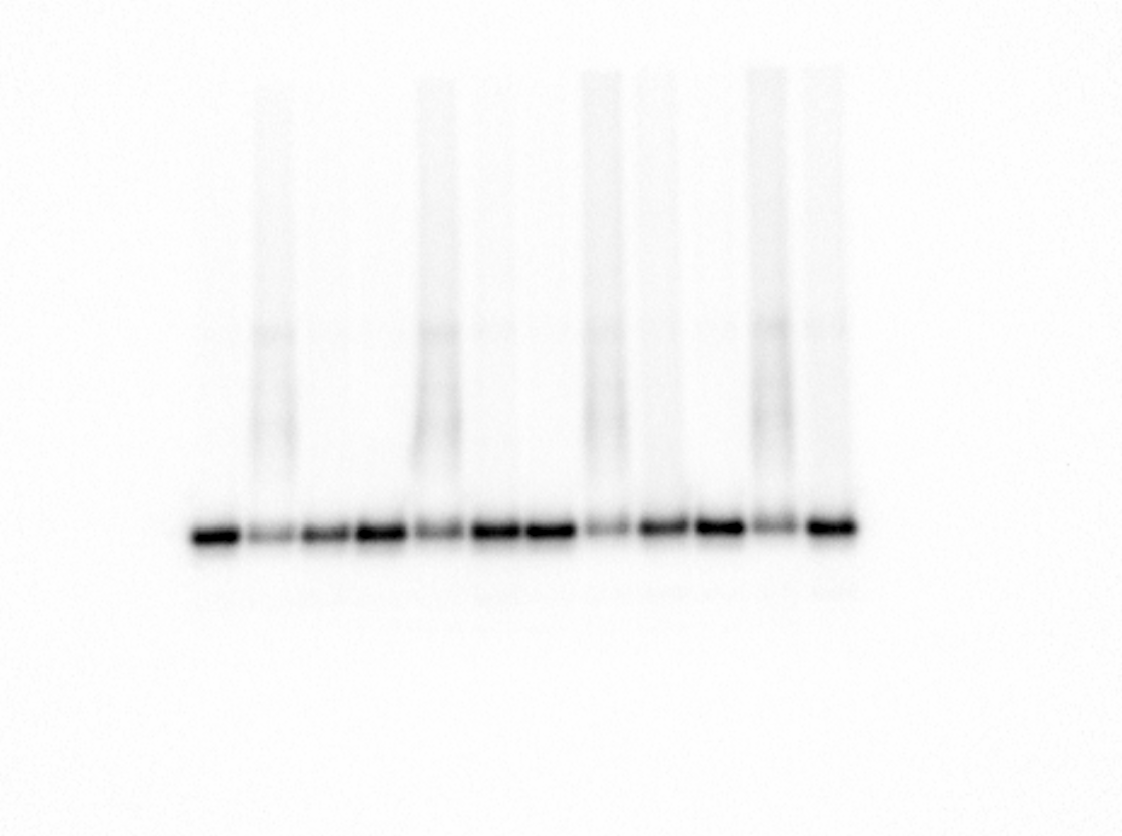

Supplement: Figure 6—figure supplement 3—source data 1. [file elife-74275-fig6-figsupp3-data1.zip › Figure 6-figure supplement 3-source data 1/figure supplement 3C/pSO111 15-60min anti-FLAG Set2.tif]

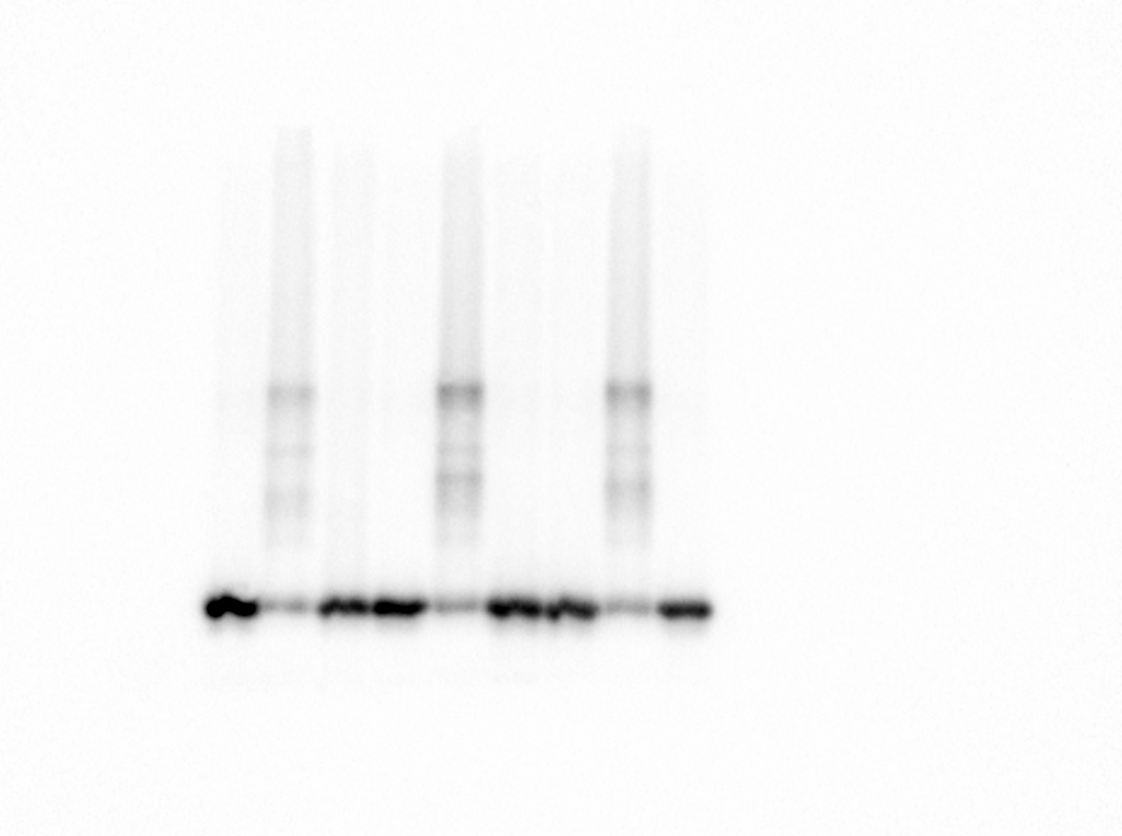

Supplement: Figure 6—figure supplement 3—source data 1. [file elife-74275-fig6-figsupp3-data1.zip › Figure 6-figure supplement 3-source data 1/figure supplement 3E/pSO230_ 236 and 237 15min anti-FLAG Set1.tif]

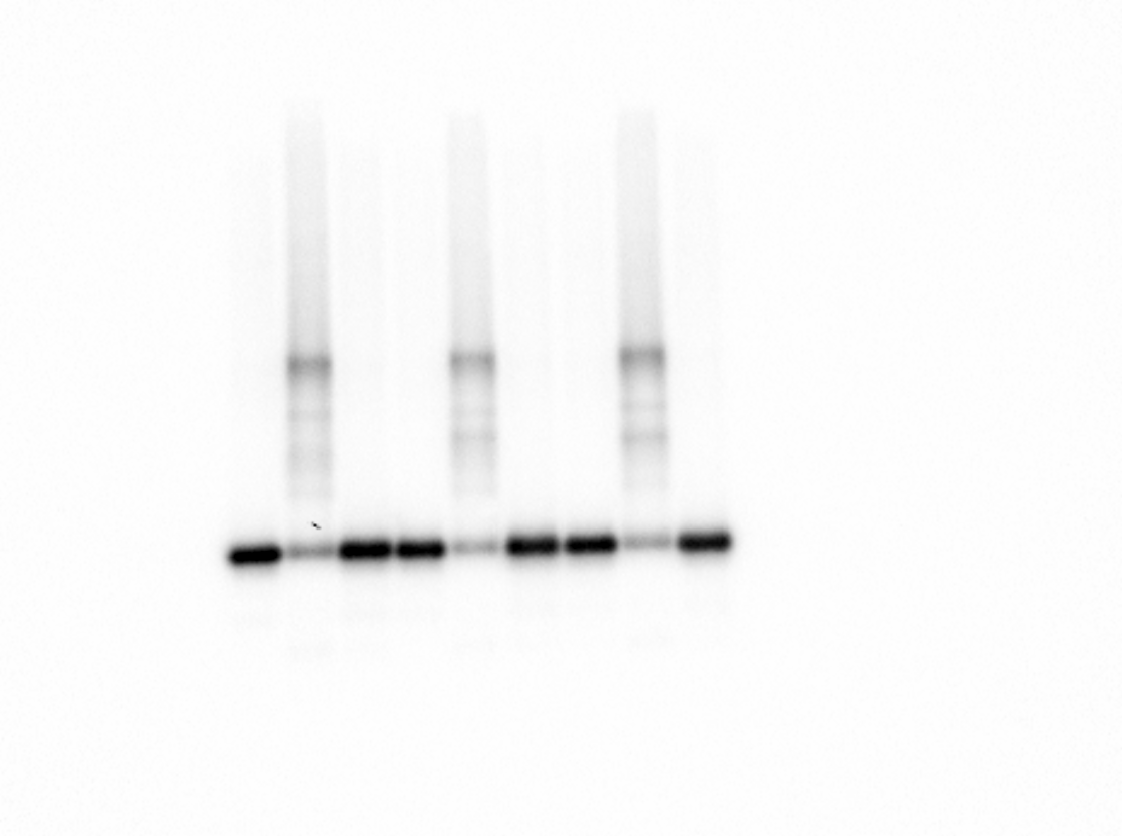

Supplement: Figure 6—figure supplement 3—source data 1. [file elife-74275-fig6-figsupp3-data1.zip › Figure 6-figure supplement 3-source data 1/figure supplement 3E/pSO230_ 236 and 237 15min anti-FLAG Set2.tif]

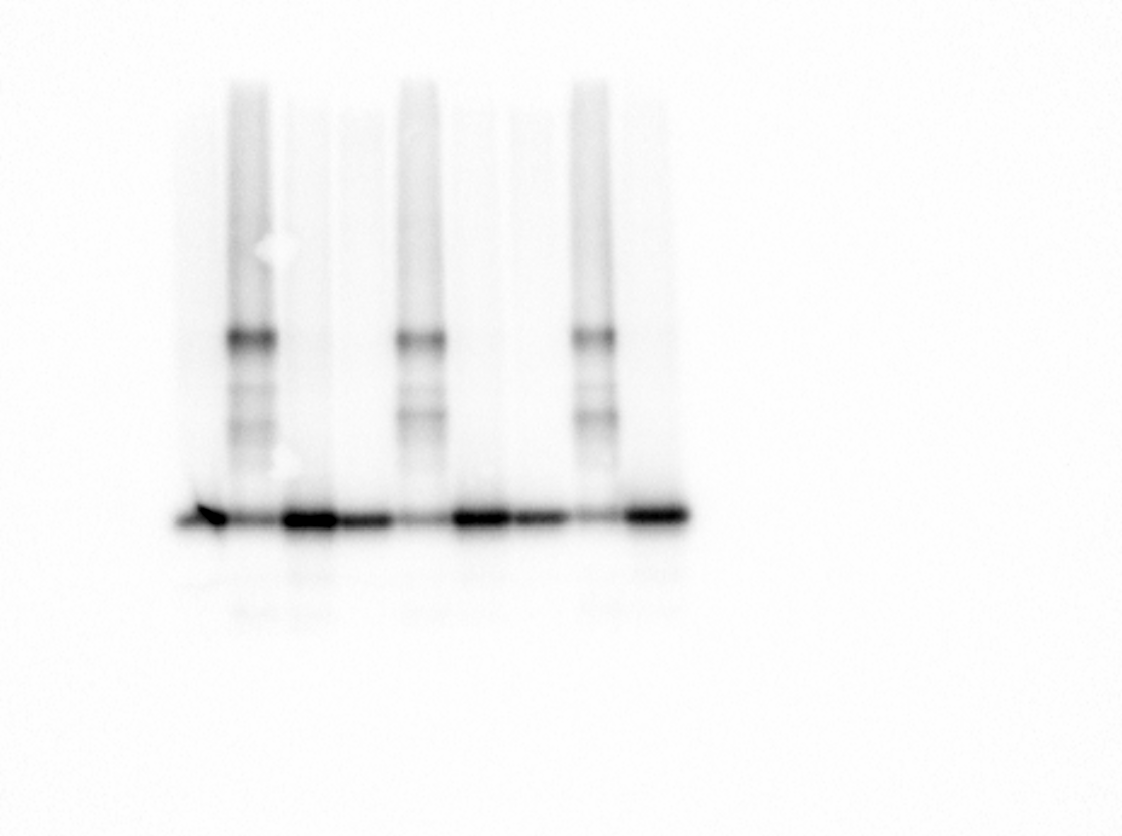

Supplement: Figure 6—figure supplement 3—source data 1. [file elife-74275-fig6-figsupp3-data1.zip › Figure 6-figure supplement 3-source data 1/figure supplement 3E/pSO230_ 236 and 237 30min anti-FLAG Set1.tif]

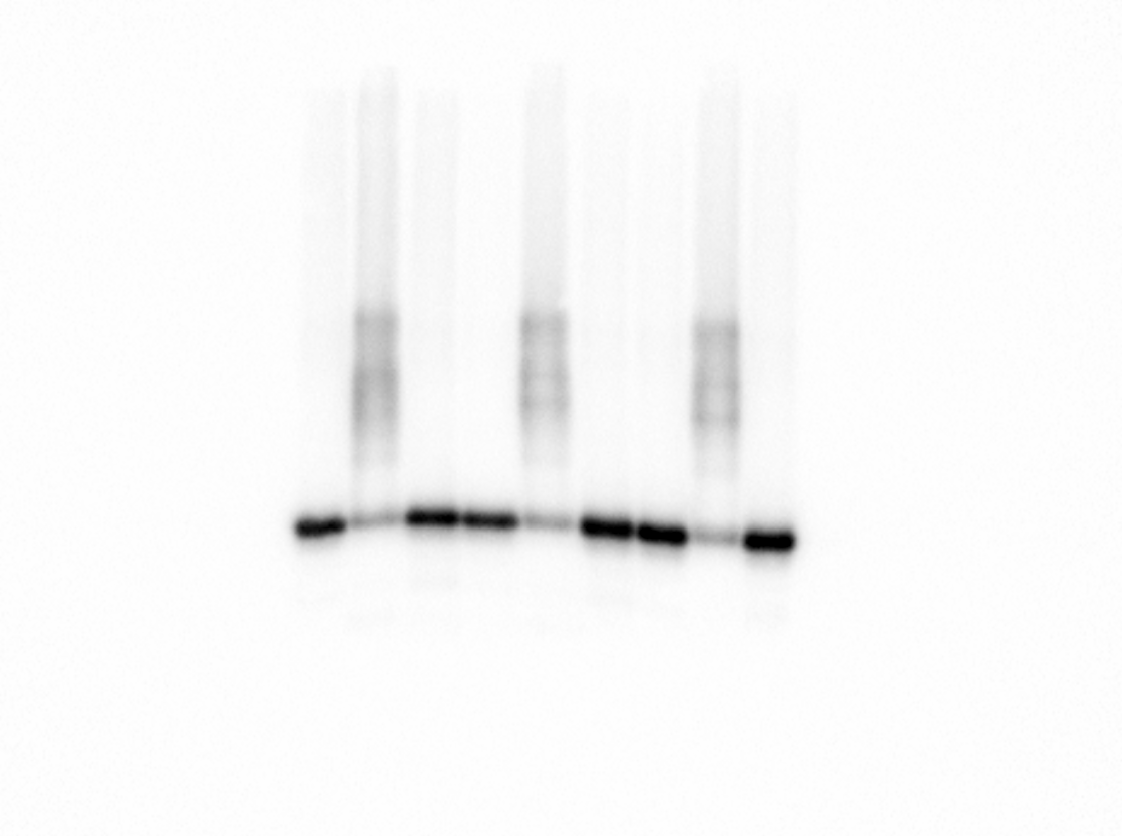

Supplement: Figure 6—figure supplement 3—source data 1. [file elife-74275-fig6-figsupp3-data1.zip › Figure 6-figure supplement 3-source data 1/figure supplement 3E/pSO230_ 236 and 237 30min anti-FLAG Set2.tif]

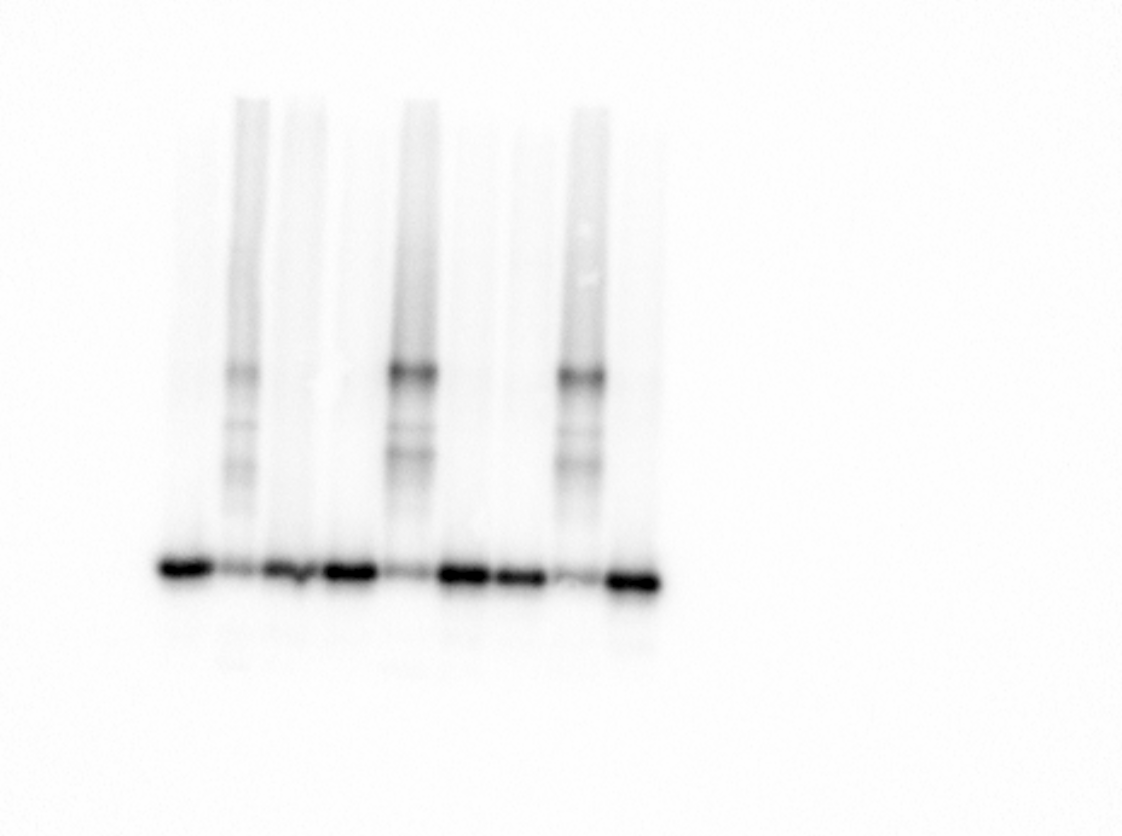

Supplement: Figure 6—figure supplement 3—source data 1. [file elife-74275-fig6-figsupp3-data1.zip › Figure 6-figure supplement 3-source data 1/figure supplement 3E/pSO230_ 236 and 237 45min anti-FLAG Set1.tif]

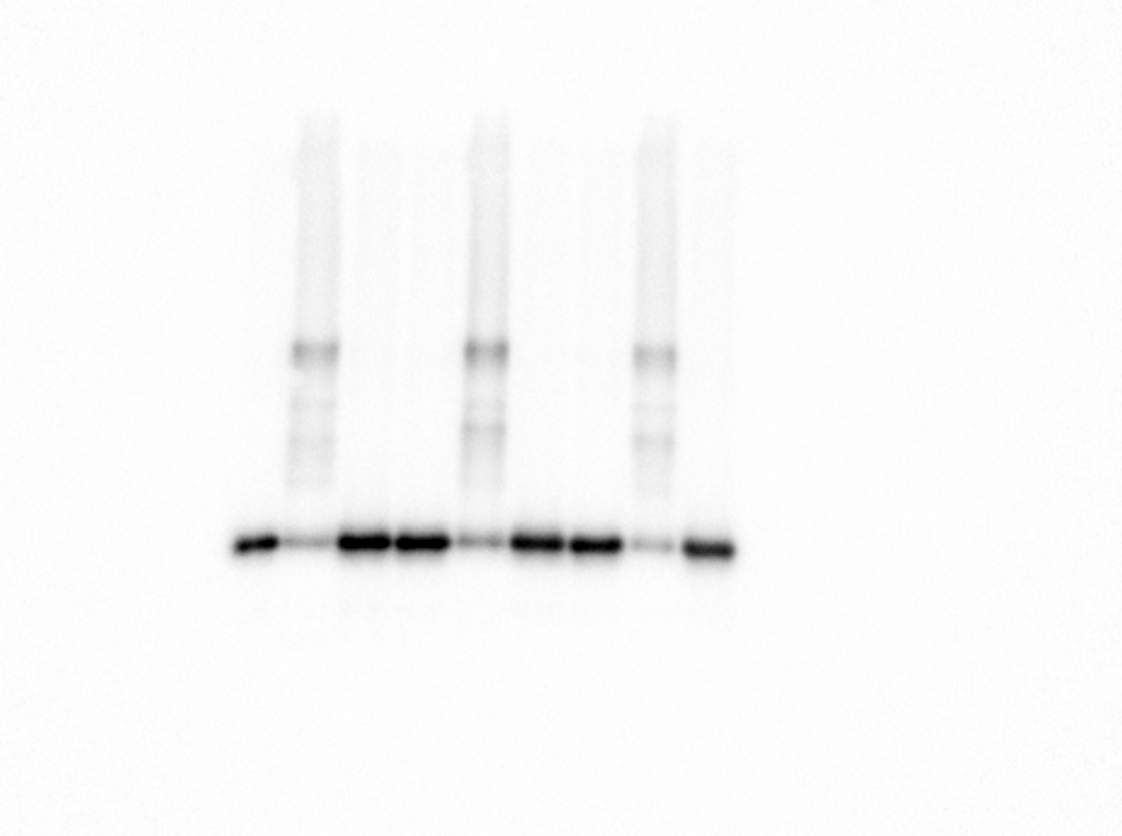

Supplement: Figure 6—figure supplement 3—source data 1. [file elife-74275-fig6-figsupp3-data1.zip › Figure 6-figure supplement 3-source data 1/figure supplement 3E/pSO230_ 236 and 237 45min anti-FLAG Set2.tif]

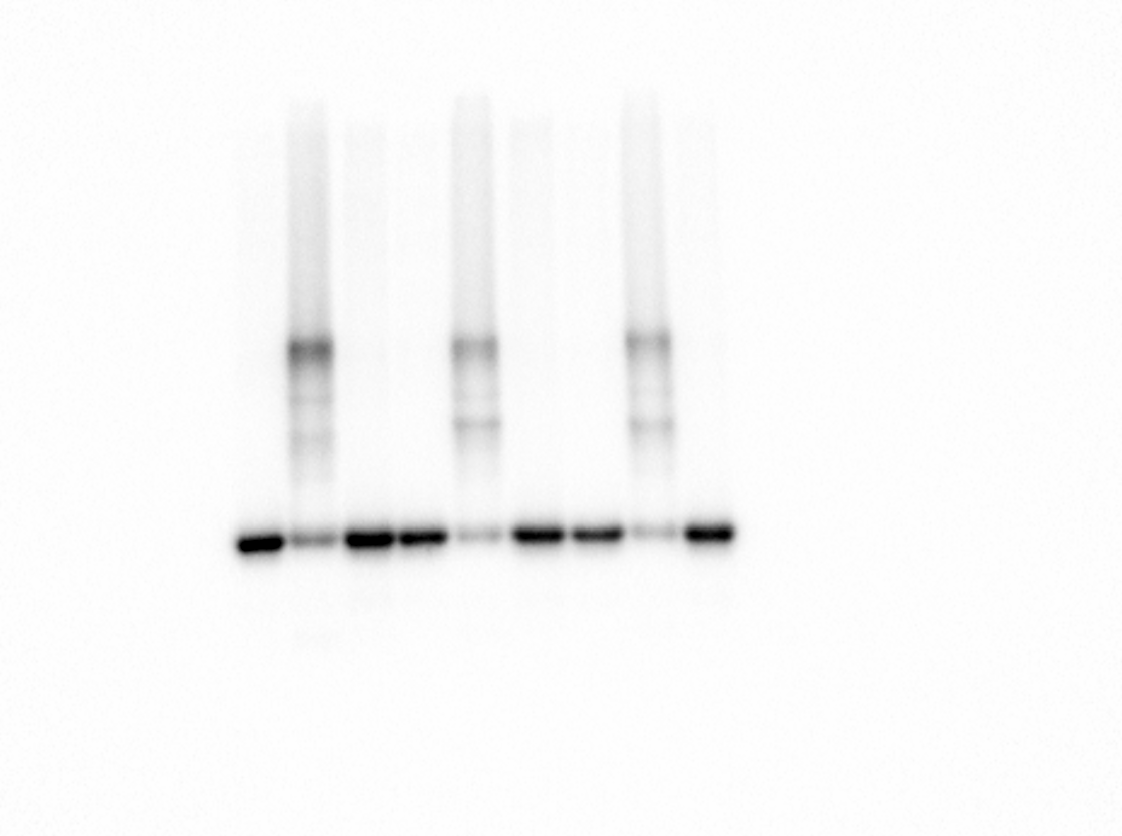

Supplement: Figure 6—figure supplement 3—source data 1. [file elife-74275-fig6-figsupp3-data1.zip › Figure 6-figure supplement 3-source data 1/figure supplement 3E/pSO230_ 236 and 237 60min anti-FLAG Set1.tif]

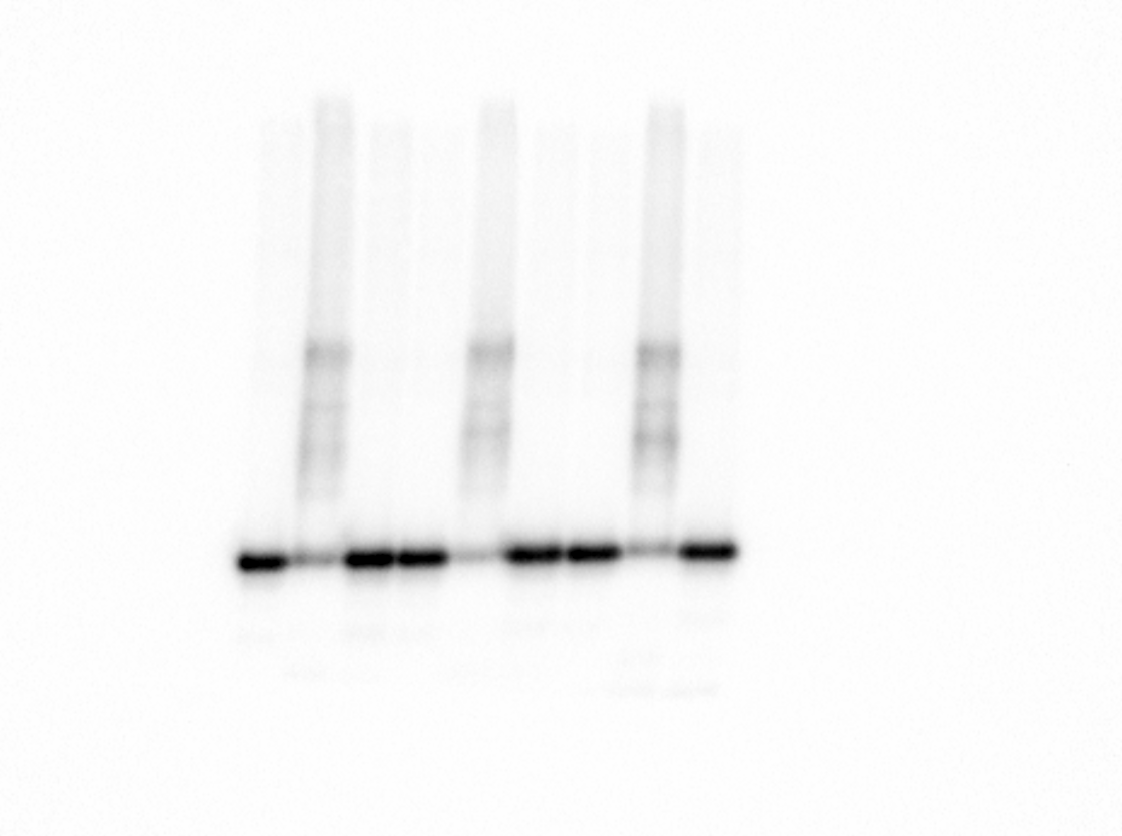

Supplement: Figure 6—figure supplement 3—source data 1. [file elife-74275-fig6-figsupp3-data1.zip › Figure 6-figure supplement 3-source data 1/figure supplement 3E/pSO230_ 236 and 237 60min anti-FLAG Set2.tif]

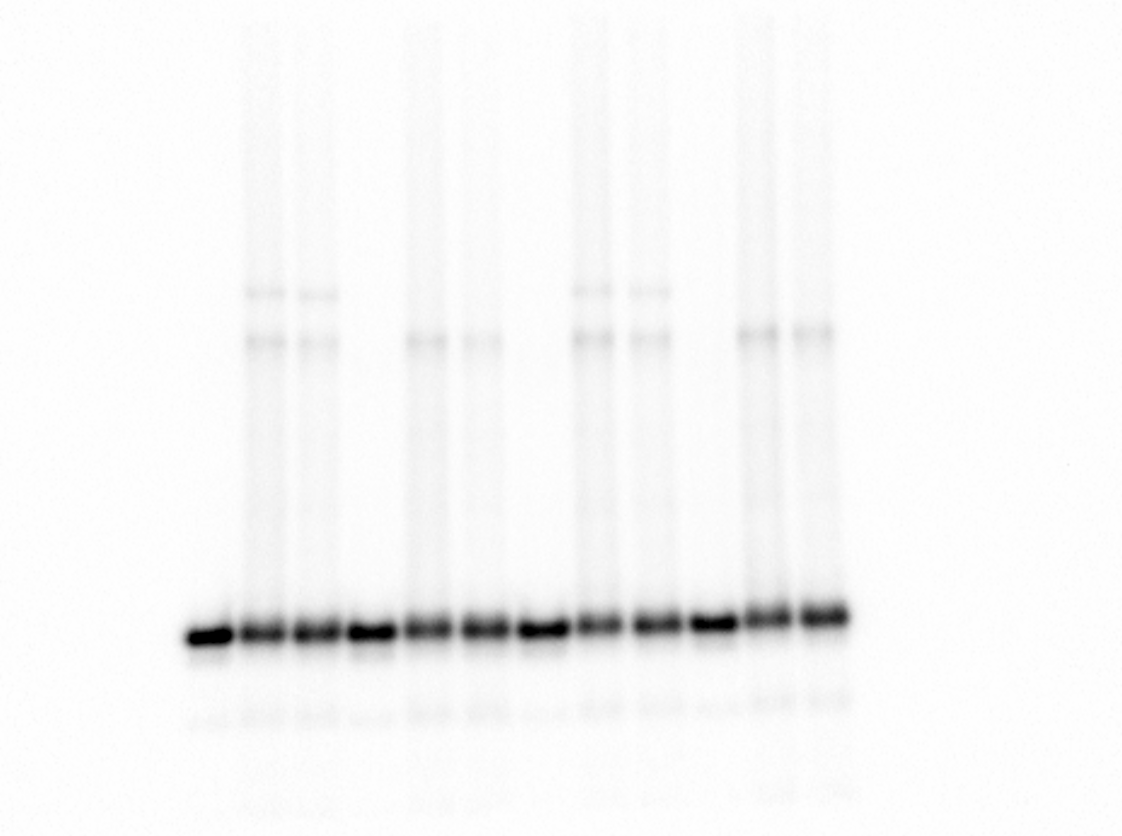

Supplement: Figure 6—figure supplement 4—source data 1. [file elife-74275-fig6-figsupp4-data1.zip › Figure 6-figure supplement 4-source data 1/fig sup 4A anti-FLAG 15-30min Set2.tif]

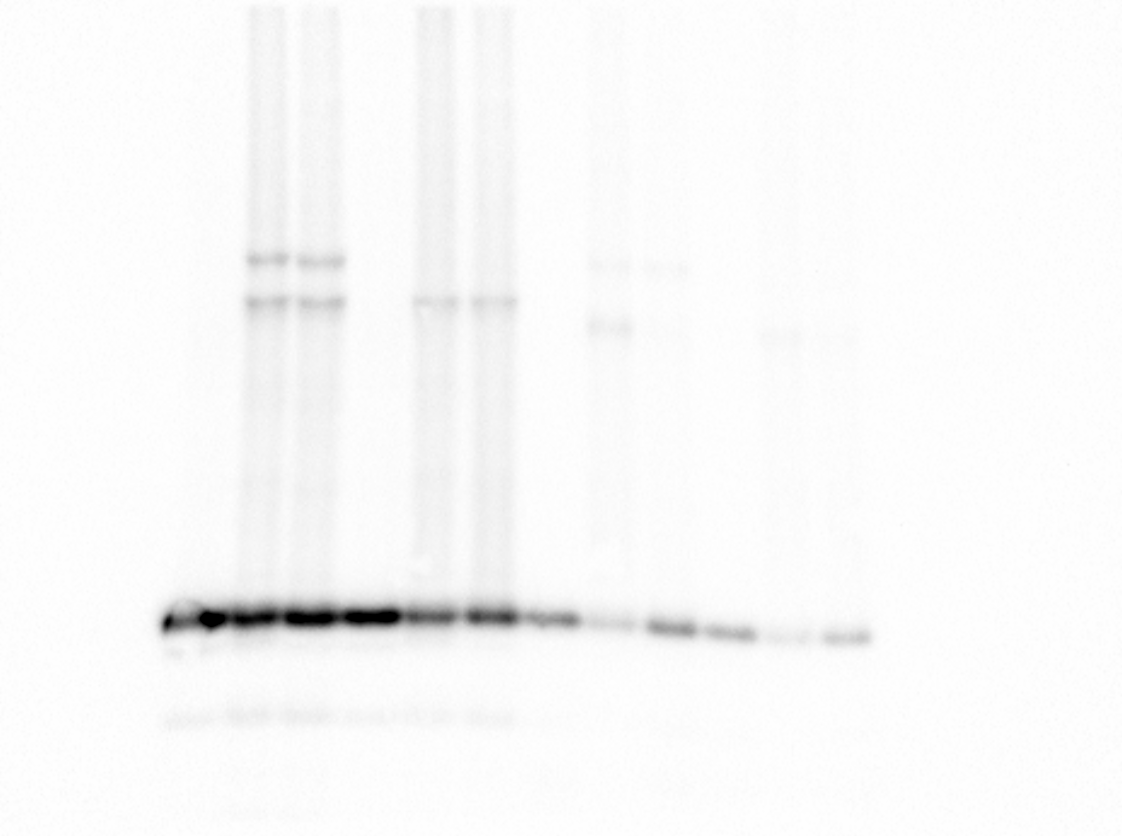

Supplement: Figure 6—figure supplement 4—source data 1. [file elife-74275-fig6-figsupp4-data1.zip › Figure 6-figure supplement 4-source data 1/fig sup 4A anti-FLAG 15min Set1.tif]

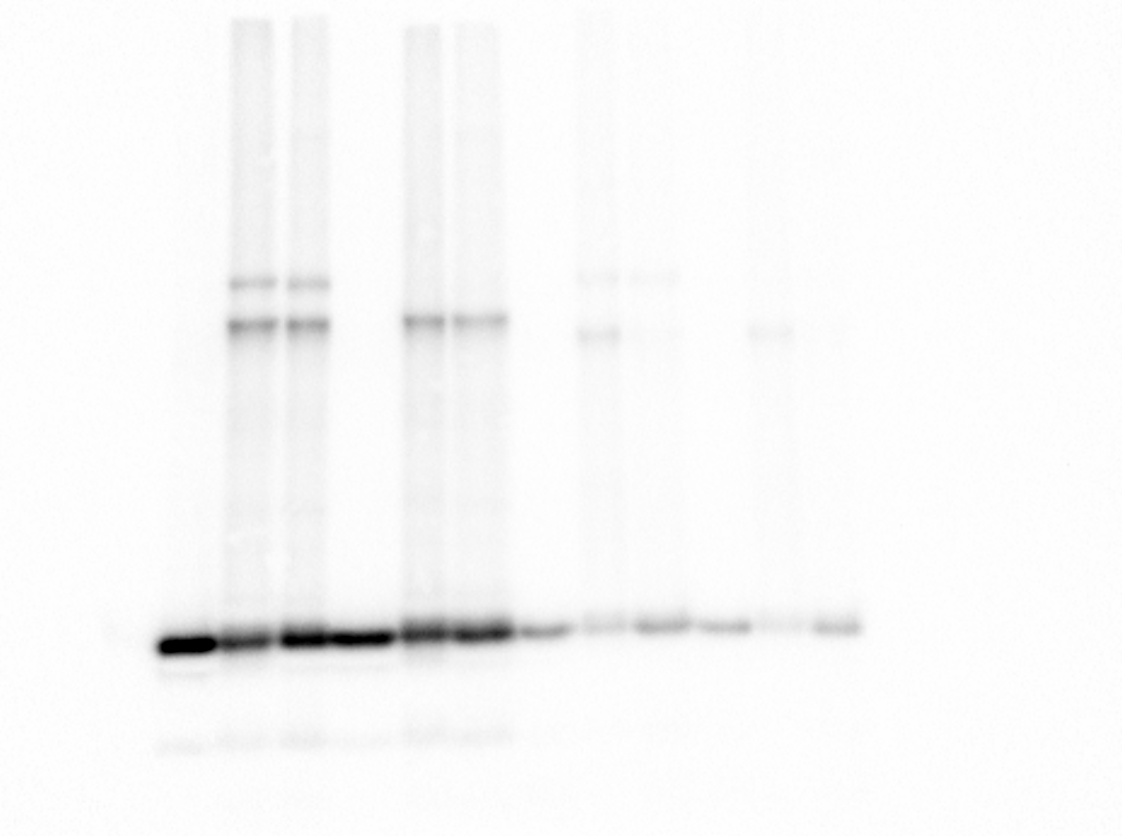

Supplement: Figure 6—figure supplement 4—source data 1. [file elife-74275-fig6-figsupp4-data1.zip › Figure 6-figure supplement 4-source data 1/fig sup 4A anti-FLAG 30min Set1.tif]

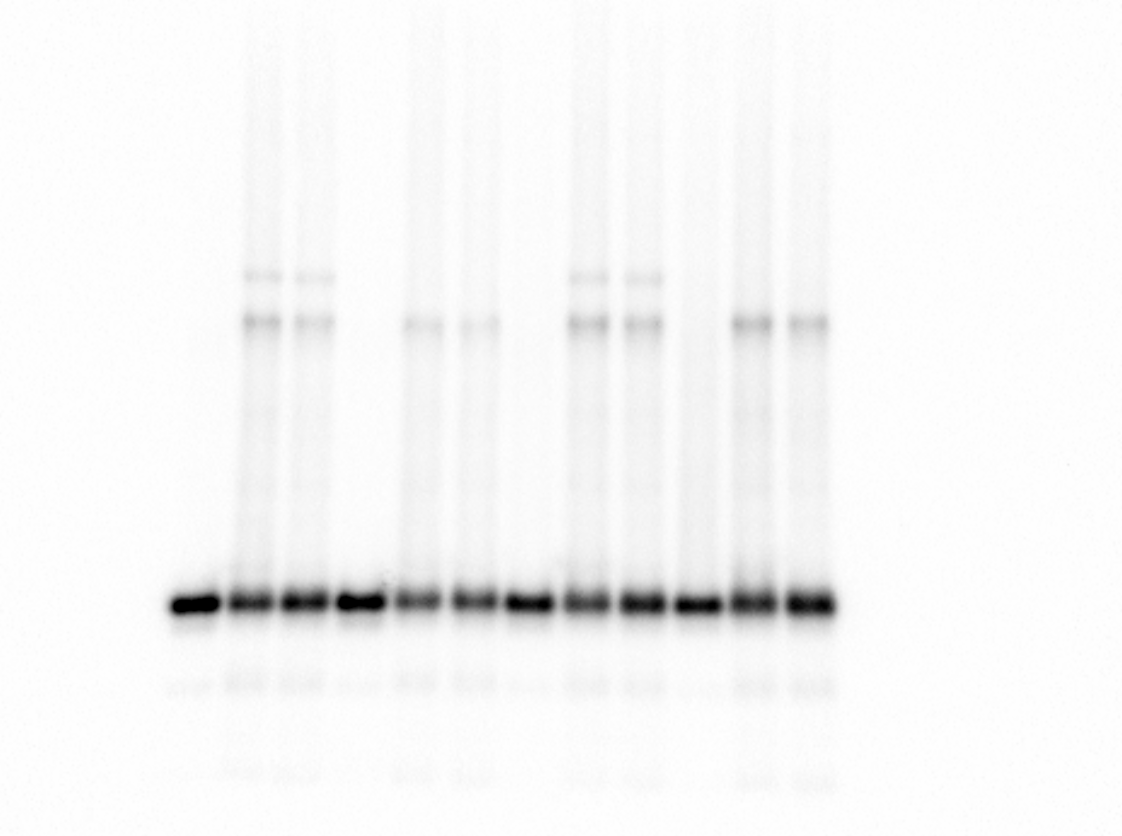

Supplement: Figure 6—figure supplement 4—source data 1. [file elife-74275-fig6-figsupp4-data1.zip › Figure 6-figure supplement 4-source data 1/fig sup 4A anti-FLAG 45-60min Set2.tif]

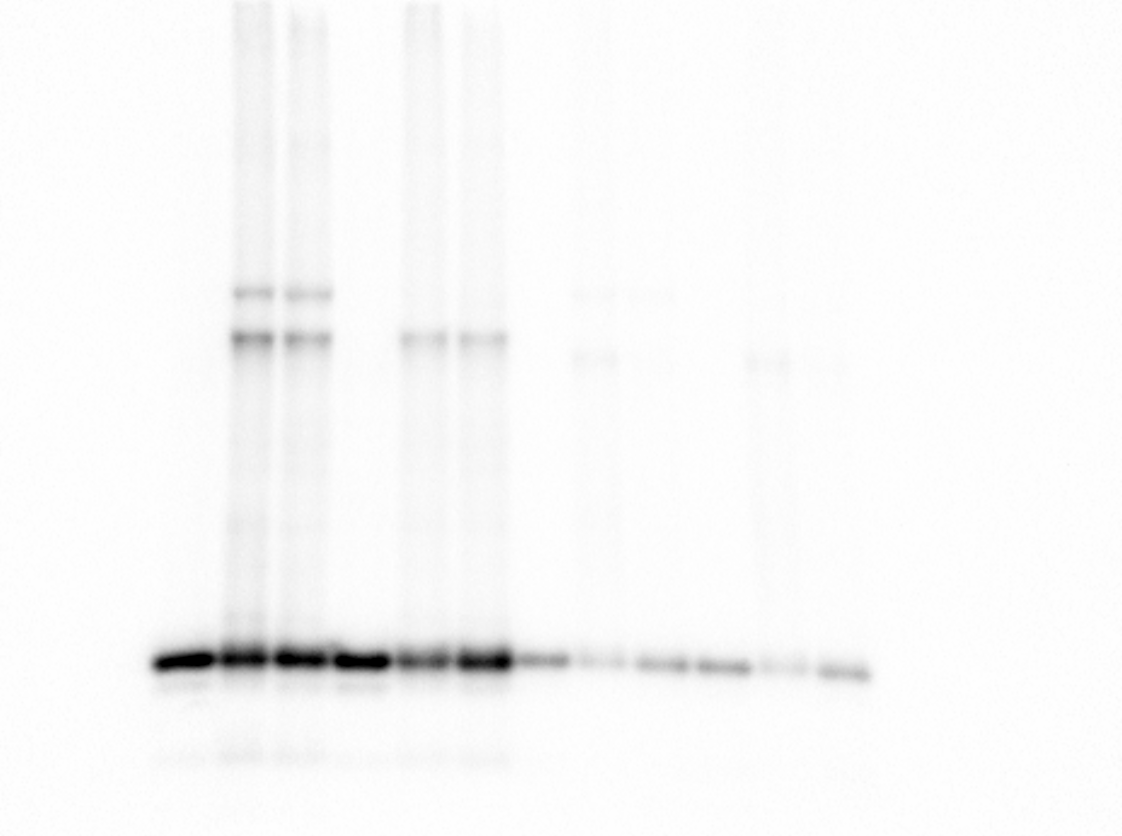

Supplement: Figure 6—figure supplement 4—source data 1. [file elife-74275-fig6-figsupp4-data1.zip › Figure 6-figure supplement 4-source data 1/fig sup 4A anti-FLAG 45min Set1.tif]

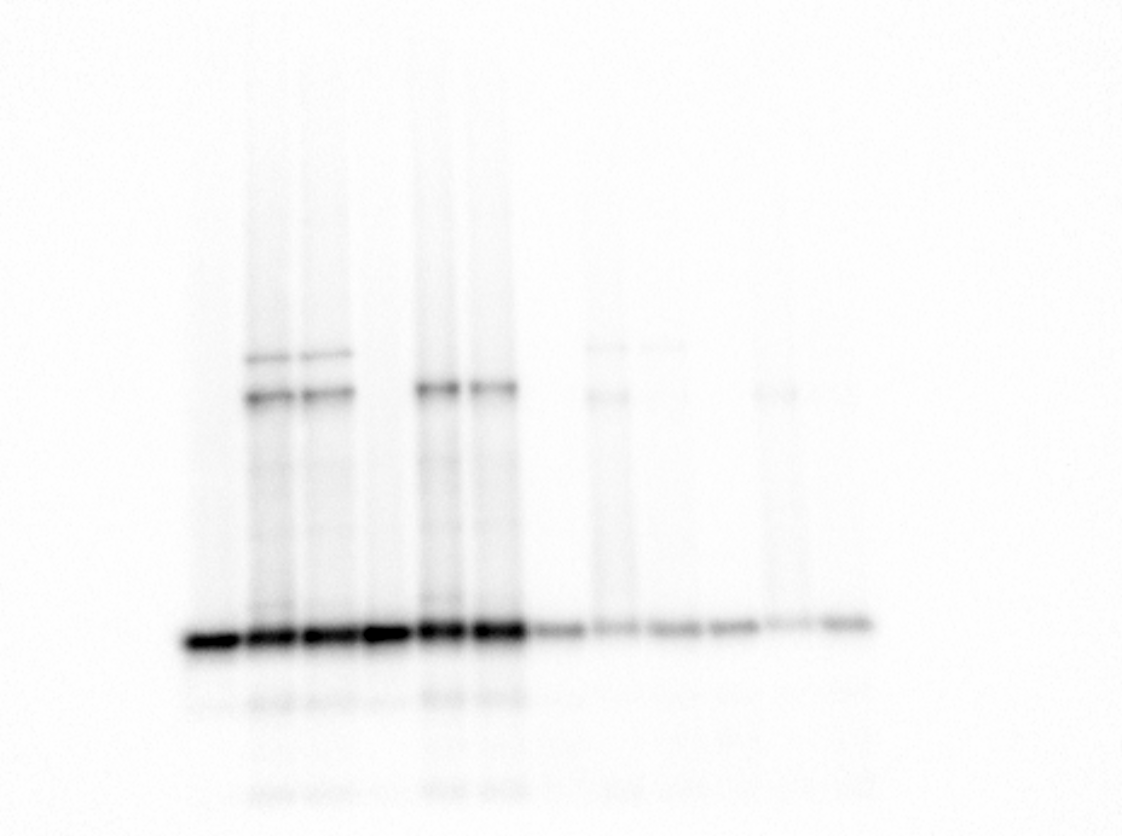

Supplement: Figure 6—figure supplement 4—source data 1. [file elife-74275-fig6-figsupp4-data1.zip › Figure 6-figure supplement 4-source data 1/fig sup 4A anti-FLAG 60min Set1.tif]

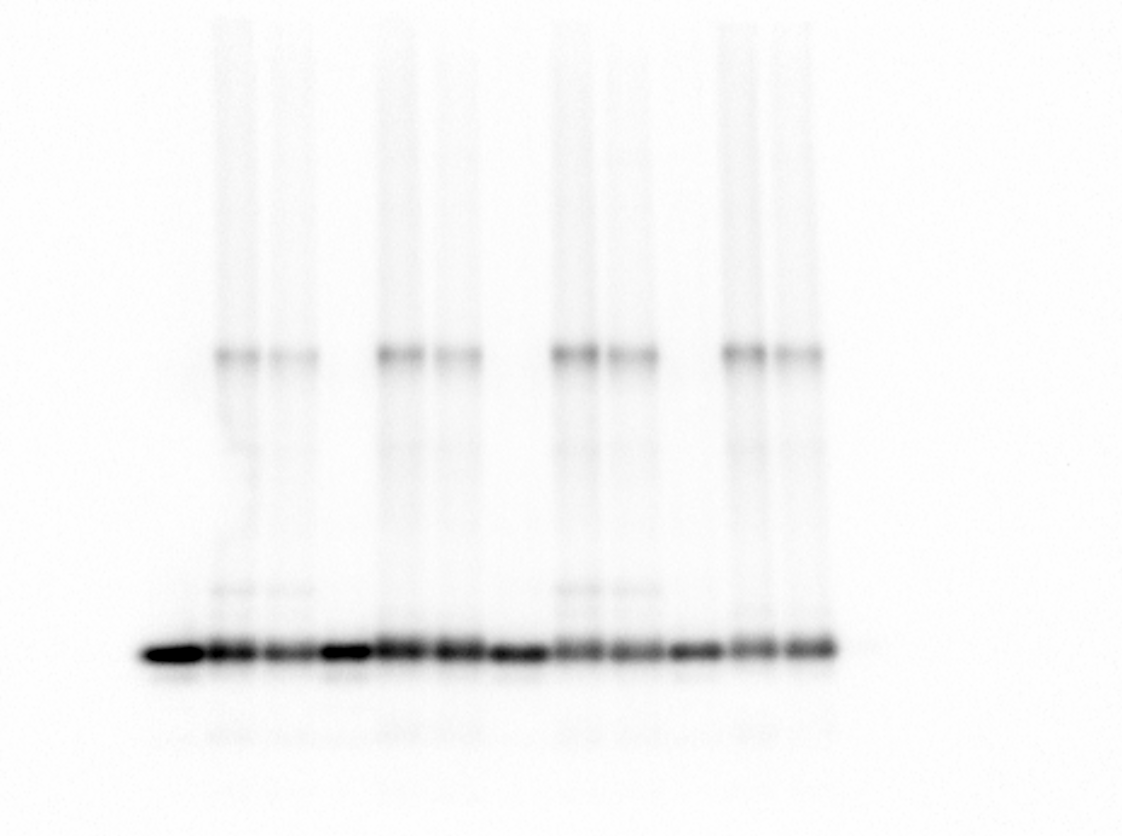

Supplement: Figure 6—figure supplement 4—source data 1. [file elife-74275-fig6-figsupp4-data1.zip › Figure 6-figure supplement 4-source data 1/fig sup 4B anti-FLAG 15-30min Set1.tif]

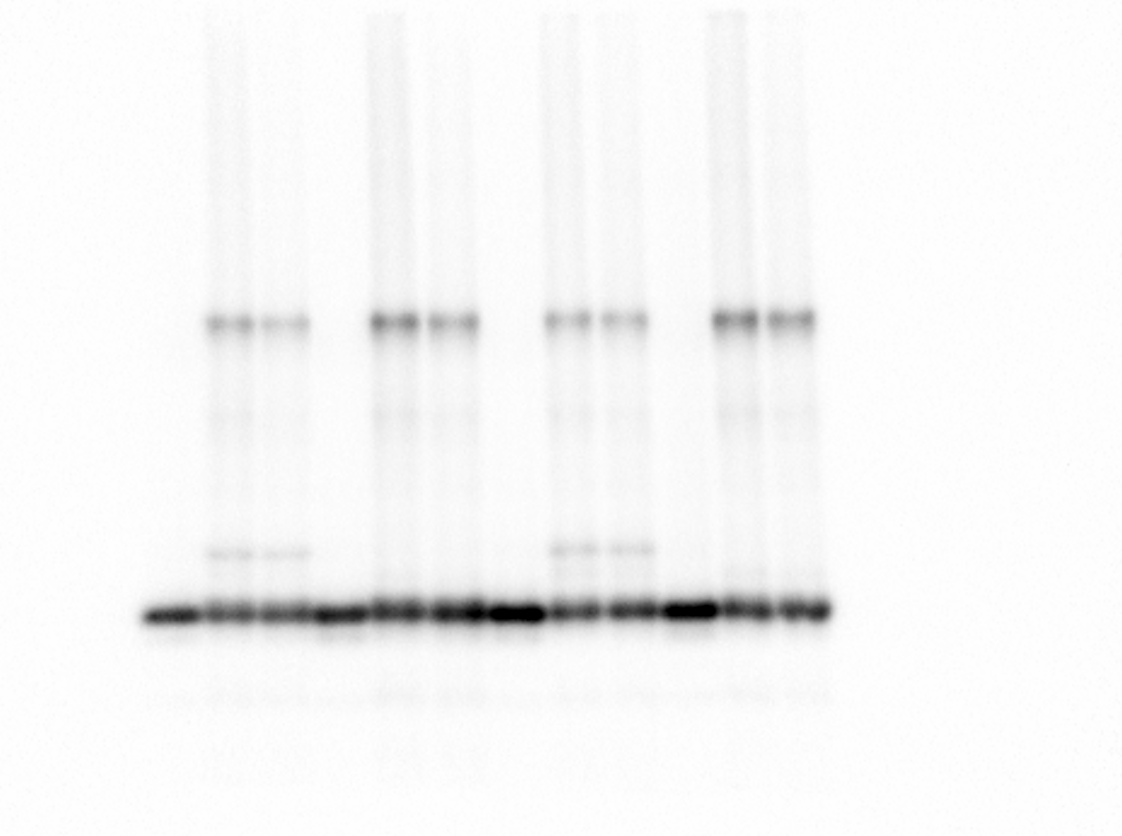

Supplement: Figure 6—figure supplement 4—source data 1. [file elife-74275-fig6-figsupp4-data1.zip › Figure 6-figure supplement 4-source data 1/fig sup 4B anti-FLAG 15-30min Set2.tif]

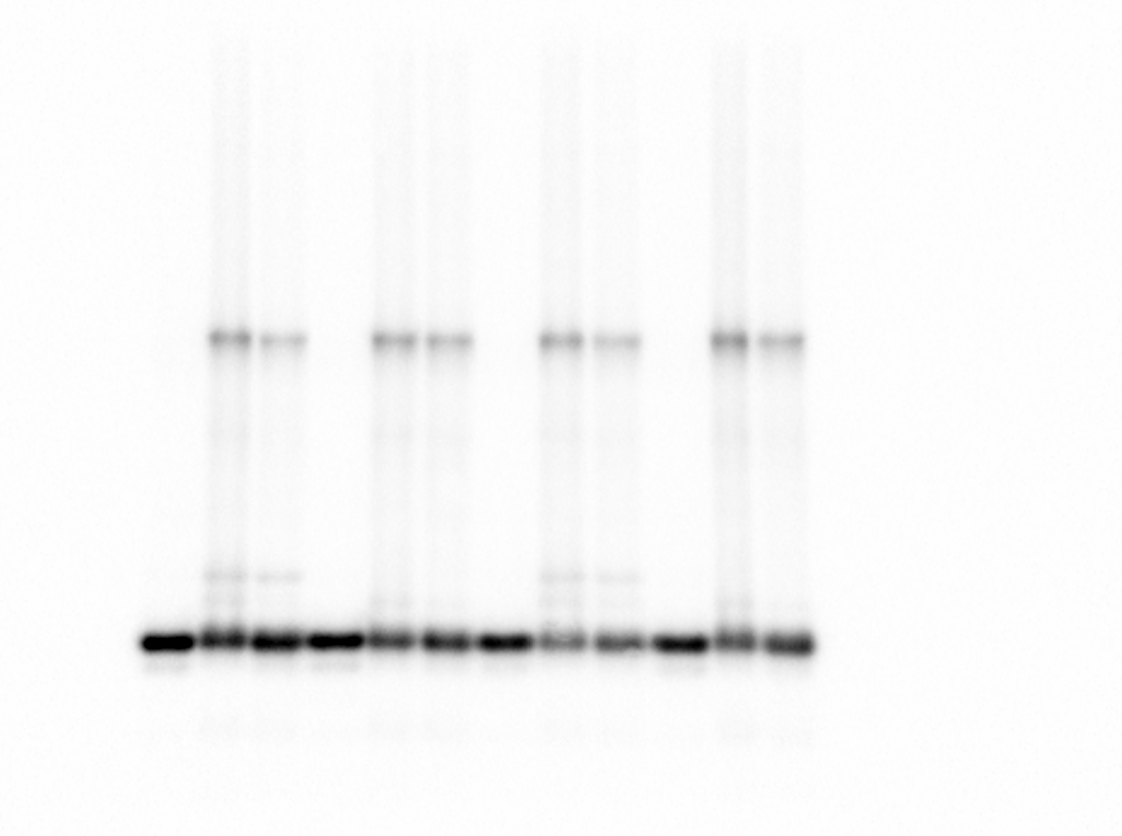

Supplement: Figure 6—figure supplement 4—source data 1. [file elife-74275-fig6-figsupp4-data1.zip › Figure 6-figure supplement 4-source data 1/fig sup 4B anti-FLAG 45-60min Set1.tif]

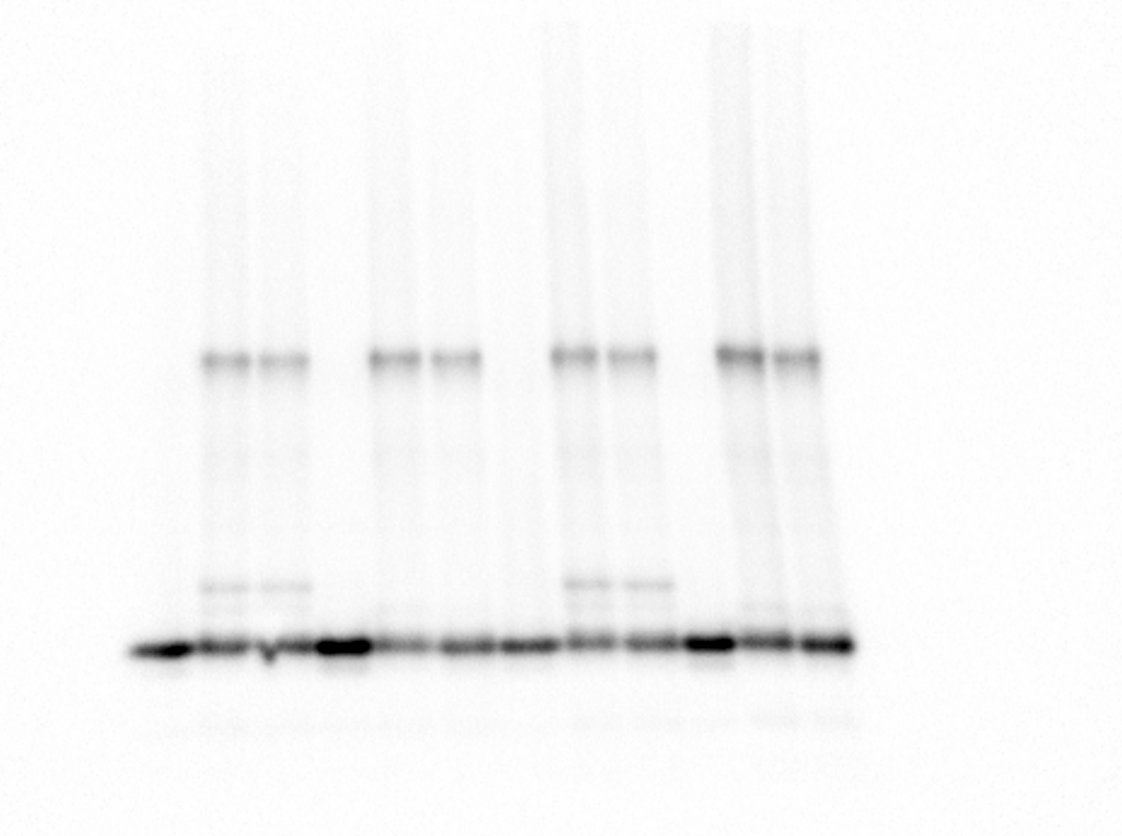

Supplement: Figure 6—figure supplement 4—source data 1. [file elife-74275-fig6-figsupp4-data1.zip › Figure 6-figure supplement 4-source data 1/fig sup 4B anti-FLAG 45-60min Set2.tif]

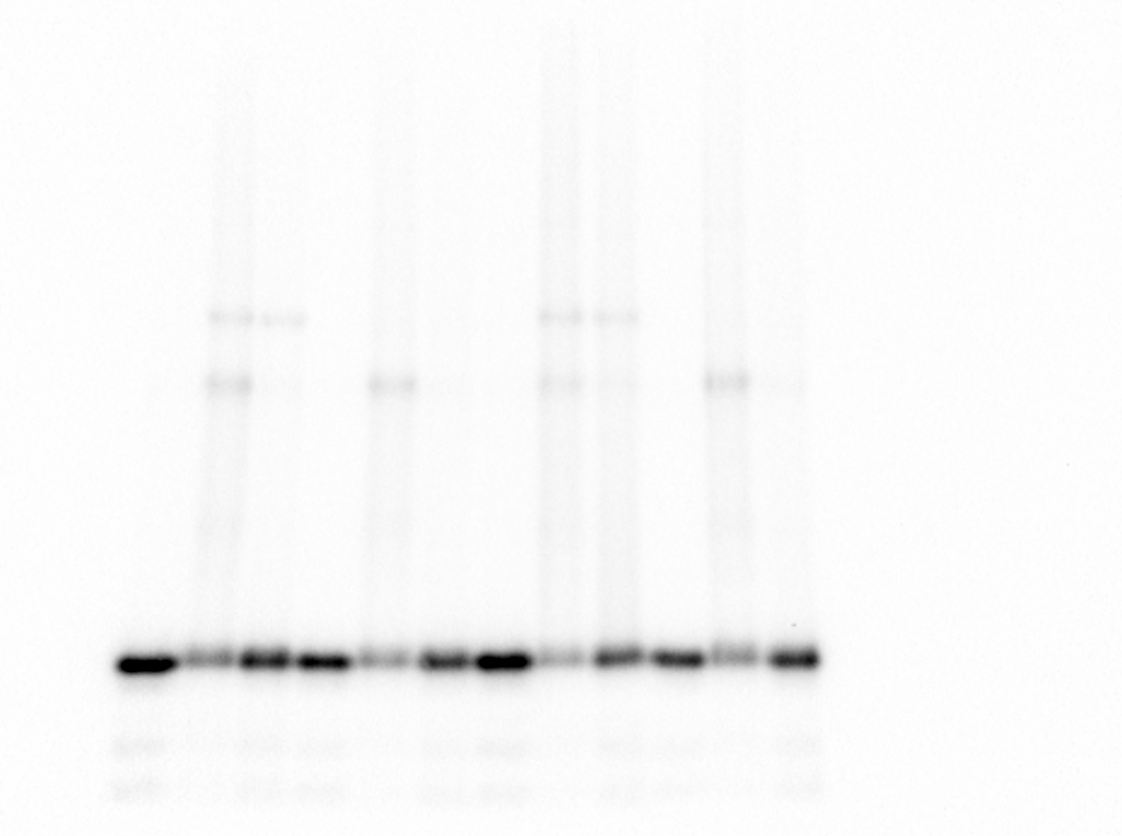

Supplement: Figure 6—figure supplement 4—source data 1. [file elife-74275-fig6-figsupp4-data1.zip › Figure 6-figure supplement 4-source data 1/fig sup 4D anti-FLAG 15-30min Set2.tif]

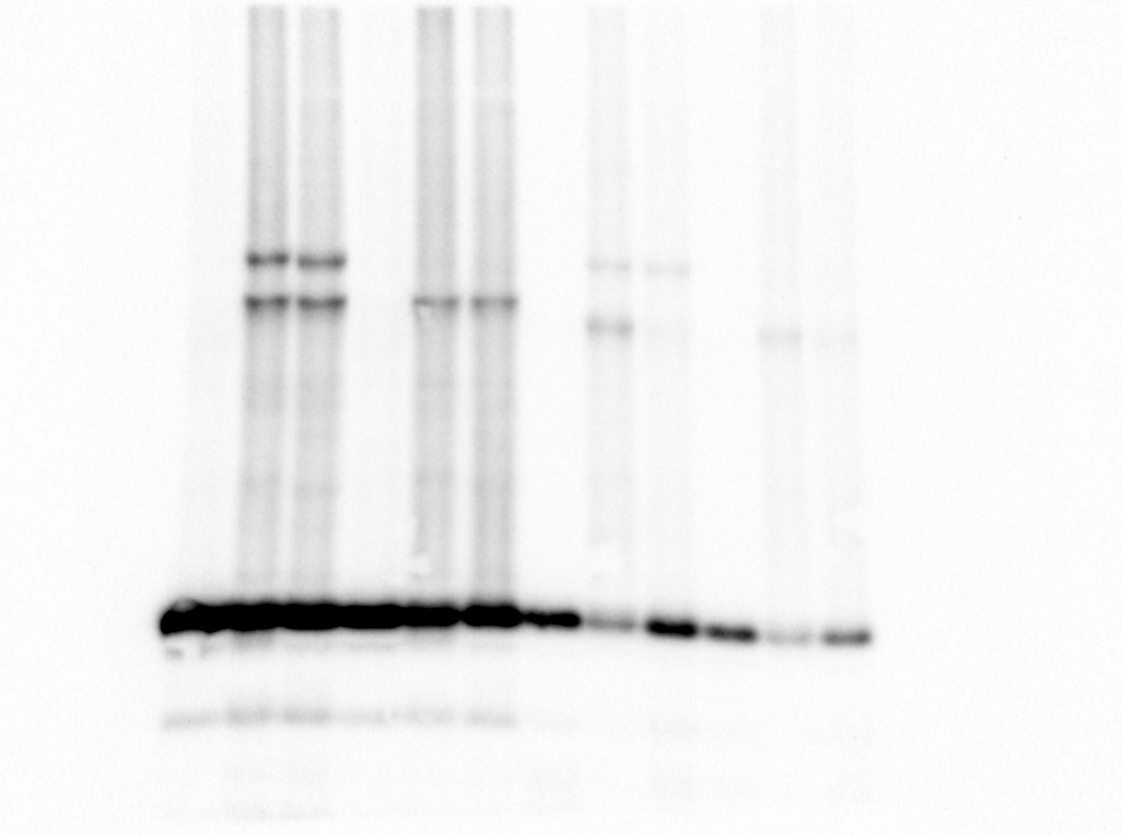

Supplement: Figure 6—figure supplement 4—source data 1. [file elife-74275-fig6-figsupp4-data1.zip › Figure 6-figure supplement 4-source data 1/fig sup 4D anti-FLAG 15min Set1.tif]

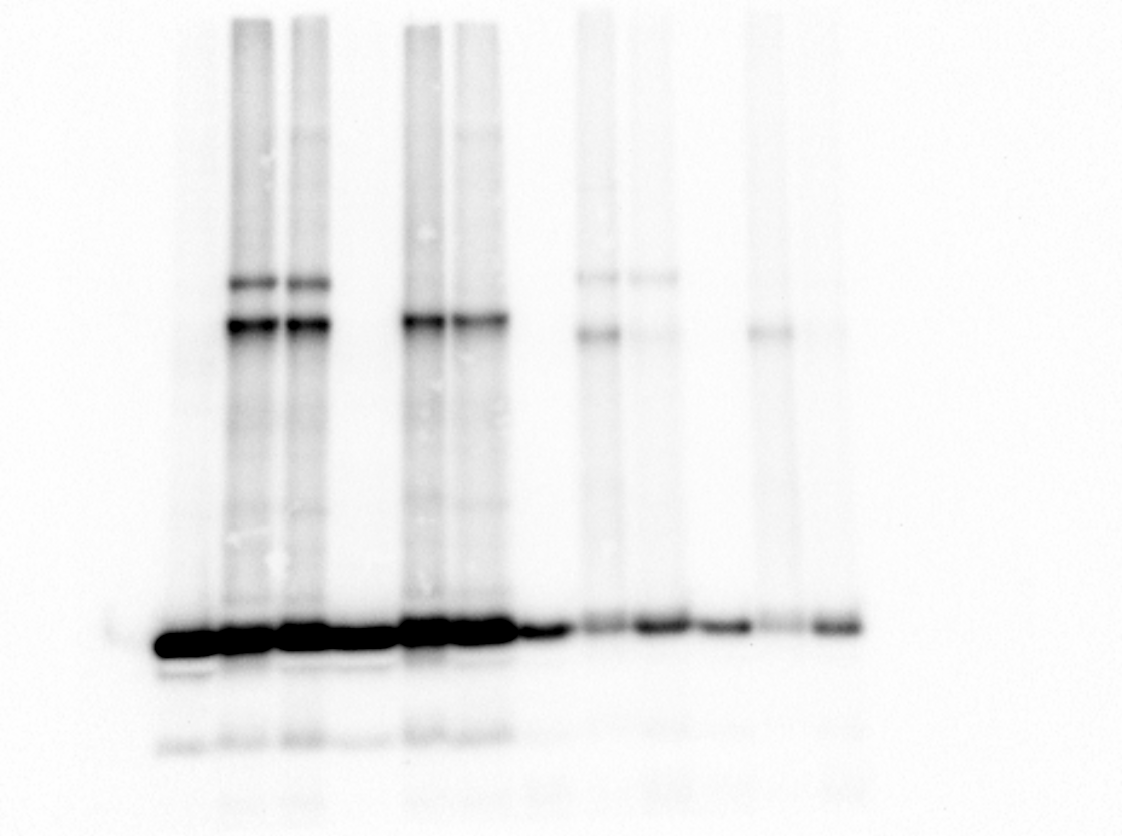

Supplement: Figure 6—figure supplement 4—source data 1. [file elife-74275-fig6-figsupp4-data1.zip › Figure 6-figure supplement 4-source data 1/fig sup 4D anti-FLAG 30min Set1.tif]

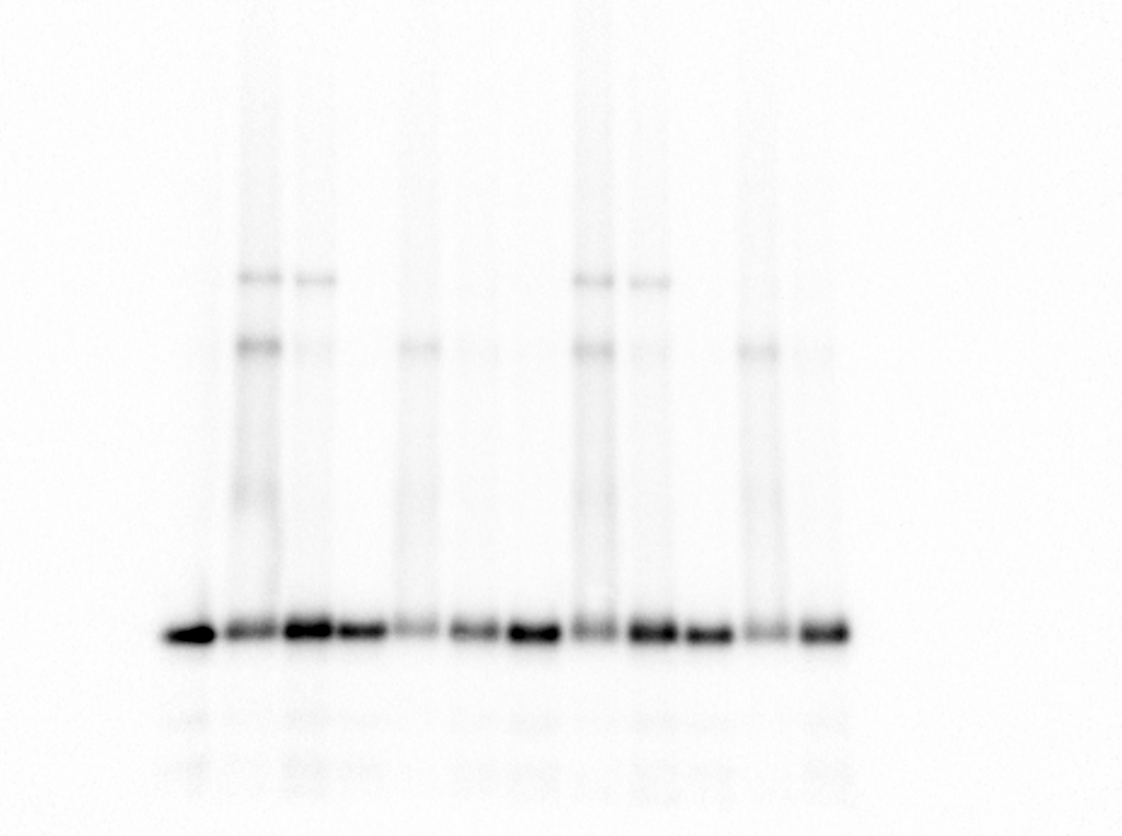

Supplement: Figure 6—figure supplement 4—source data 1. [file elife-74275-fig6-figsupp4-data1.zip › Figure 6-figure supplement 4-source data 1/fig sup 4D anti-FLAG 45-60min Set2.tif]

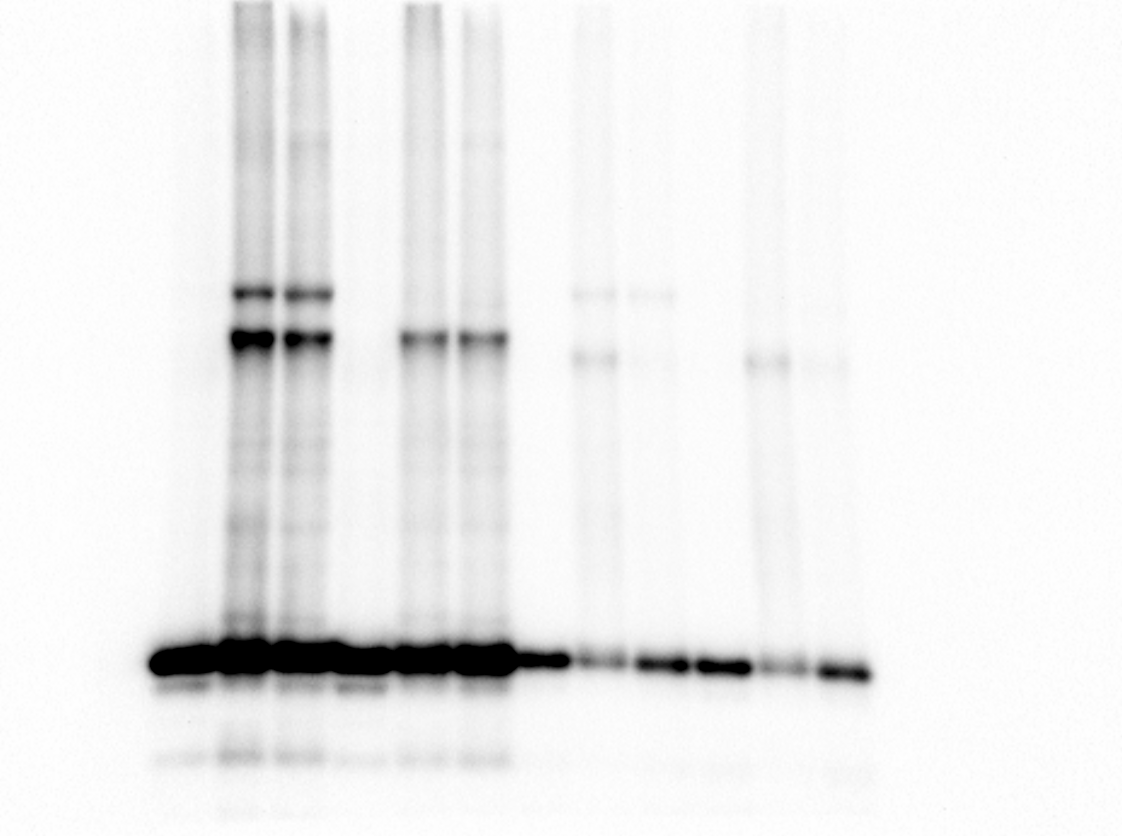

Supplement: Figure 6—figure supplement 4—source data 1. [file elife-74275-fig6-figsupp4-data1.zip › Figure 6-figure supplement 4-source data 1/fig sup 4D anti-FLAG 45min Set1.tif]

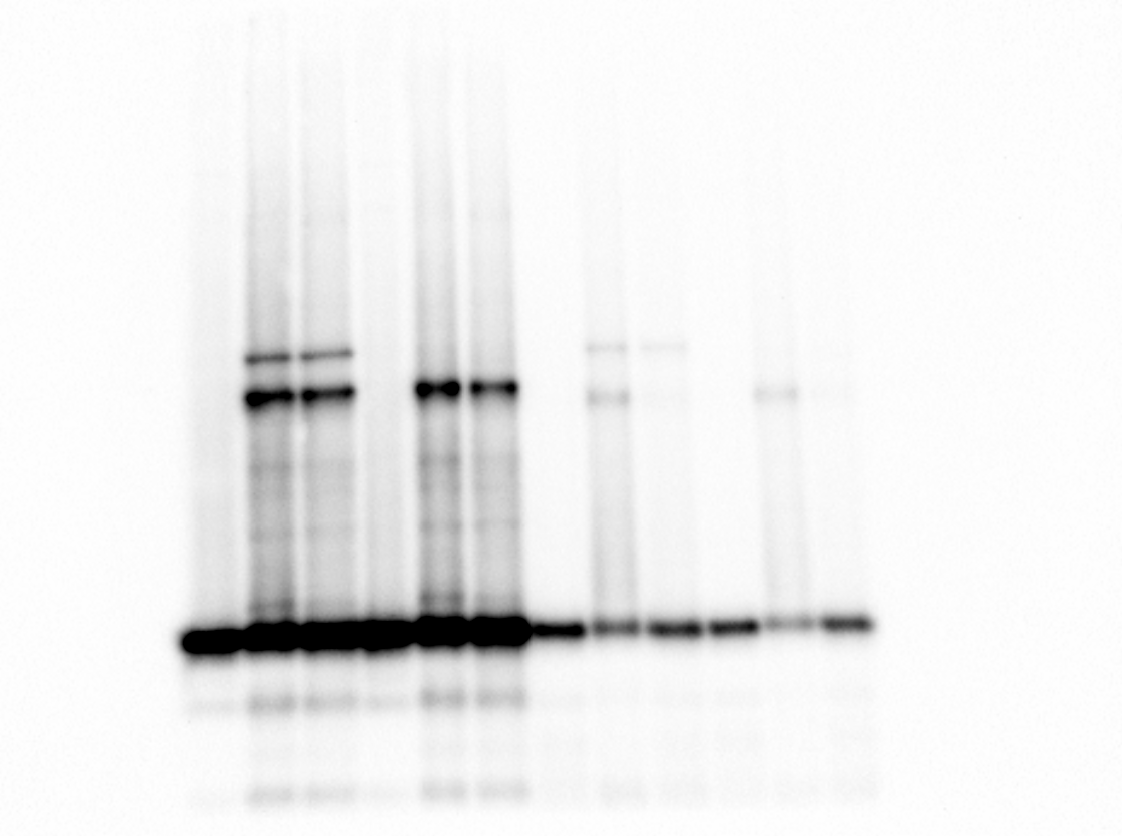

Supplement: Figure 6—figure supplement 4—source data 1. [file elife-74275-fig6-figsupp4-data1.zip › Figure 6-figure supplement 4-source data 1/fig sup 4D anti-FLAG 60min Set1.tif]

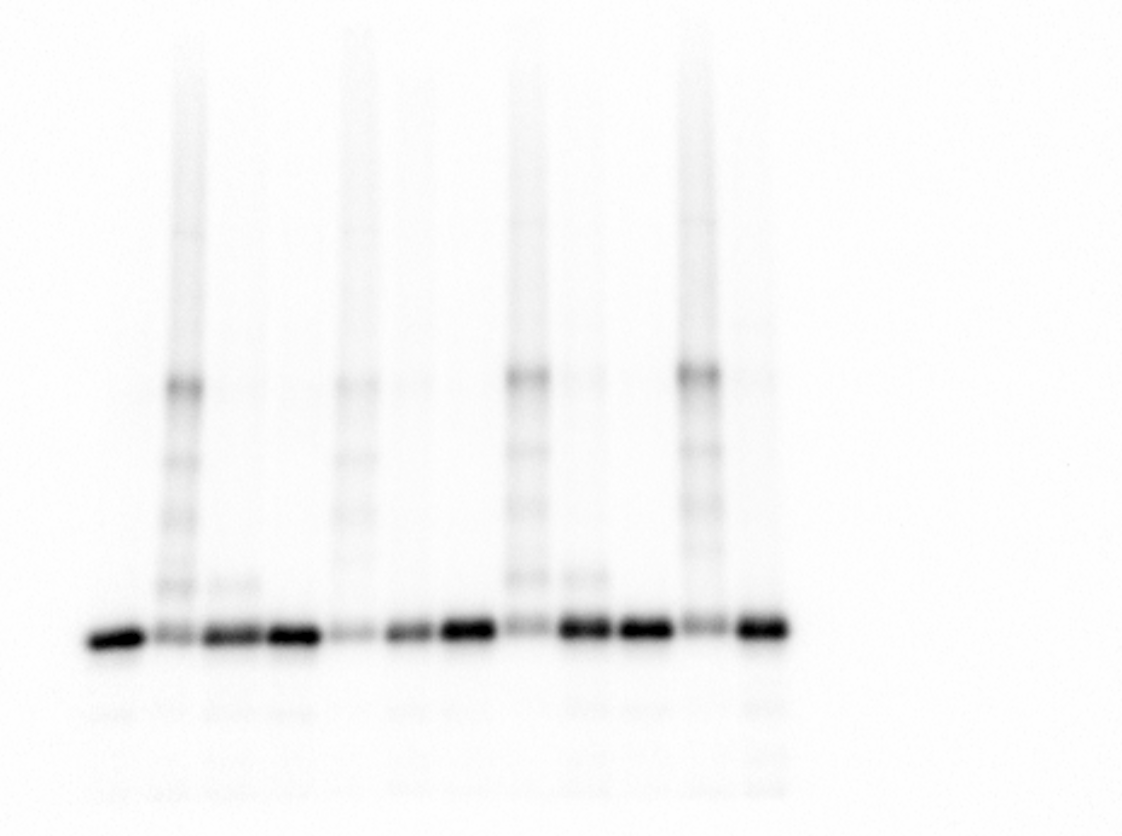

Supplement: Figure 6—figure supplement 4—source data 1. [file elife-74275-fig6-figsupp4-data1.zip › Figure 6-figure supplement 4-source data 1/fig sup 4E anti-FLAG 15-30min Set1.tif]

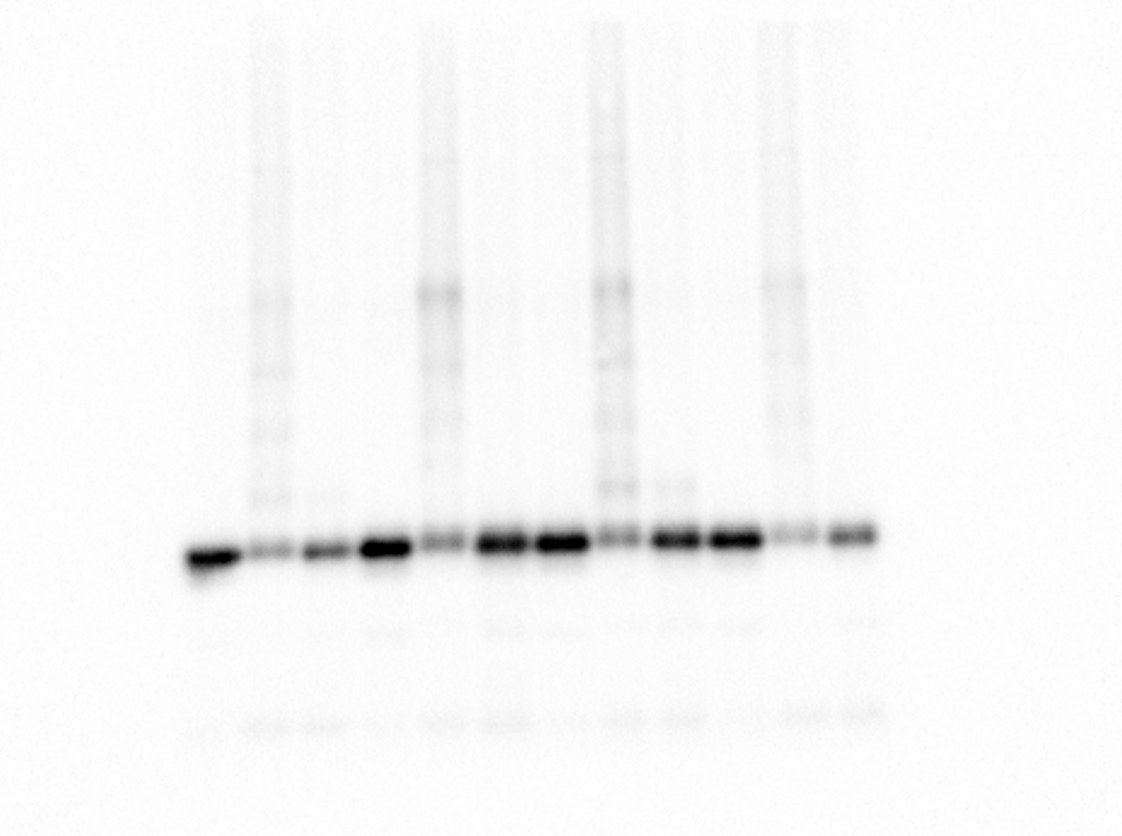

Supplement: Figure 6—figure supplement 4—source data 1. [file elife-74275-fig6-figsupp4-data1.zip › Figure 6-figure supplement 4-source data 1/fig sup 4E anti-FLAG 15-30min Set2.tif]

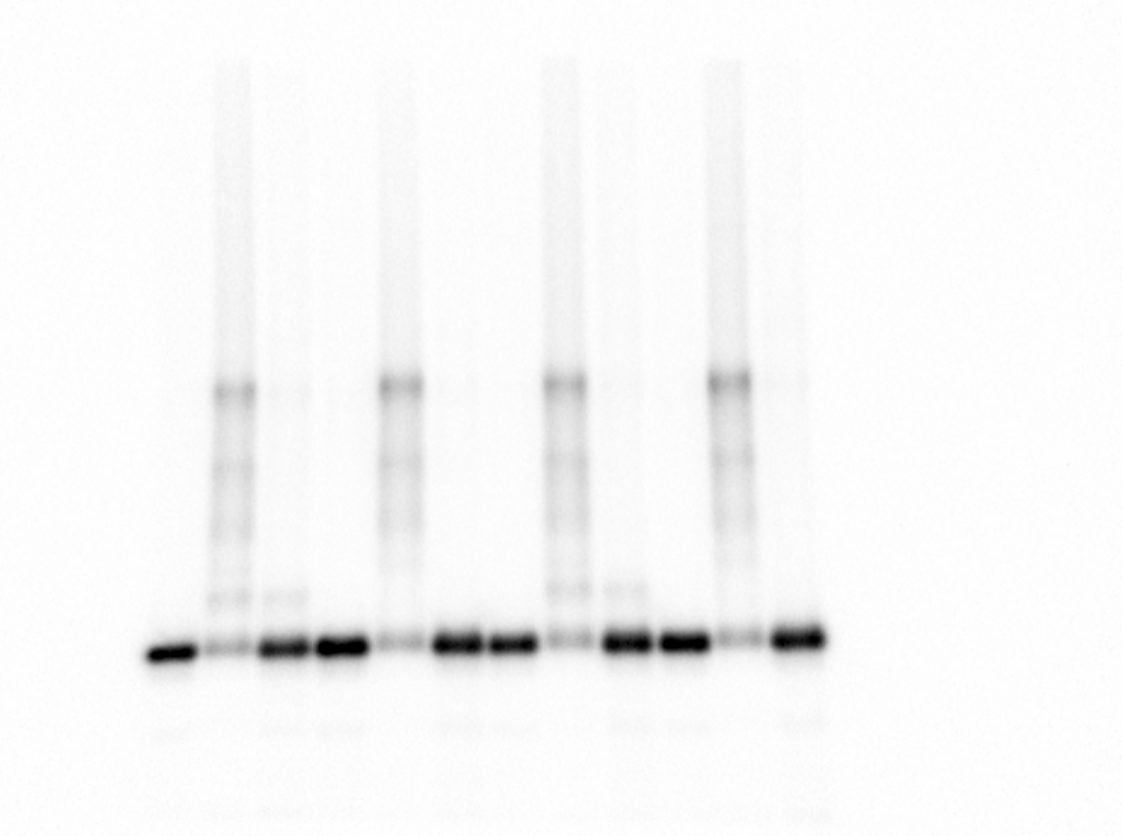

Supplement: Figure 6—figure supplement 4—source data 1. [file elife-74275-fig6-figsupp4-data1.zip › Figure 6-figure supplement 4-source data 1/fig sup 4E anti-FLAG 45-60min Set1.tif]

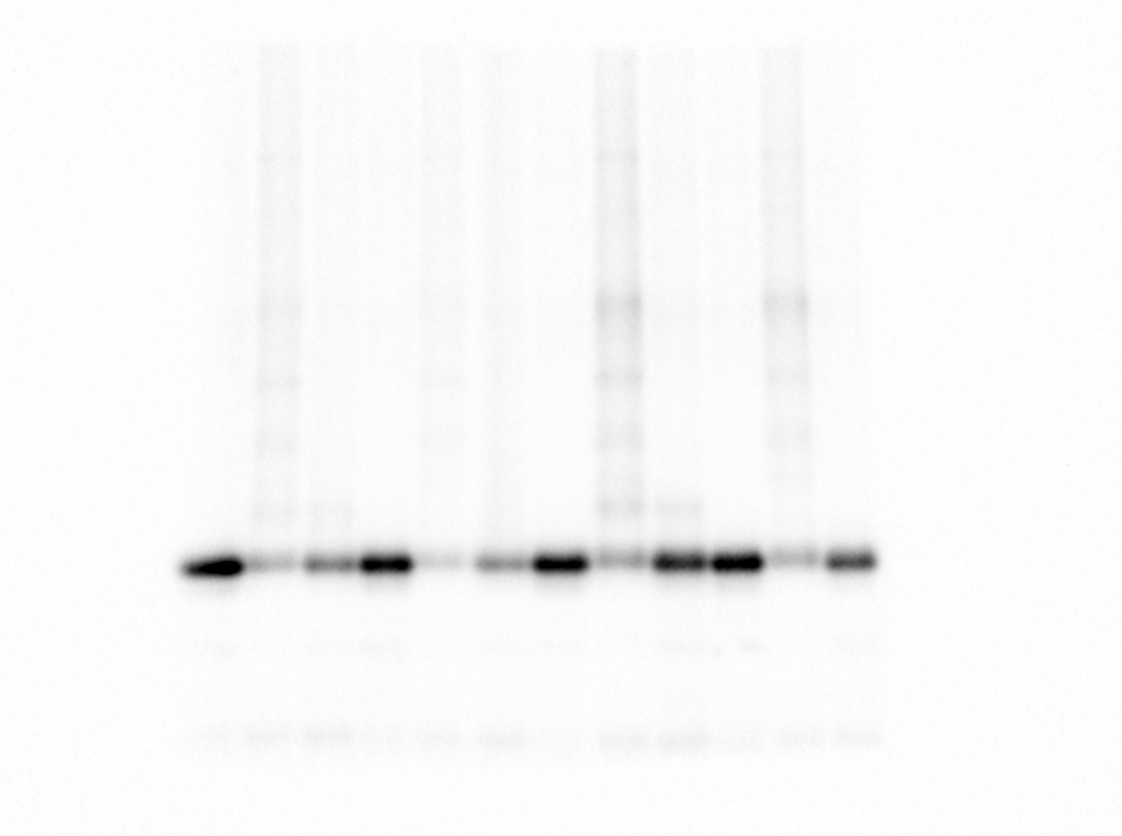

Supplement: Figure 6—figure supplement 4—source data 1. [file elife-74275-fig6-figsupp4-data1.zip › Figure 6-figure supplement 4-source data 1/fig sup 4E anti-FLAG 45-60min Set2.tif]

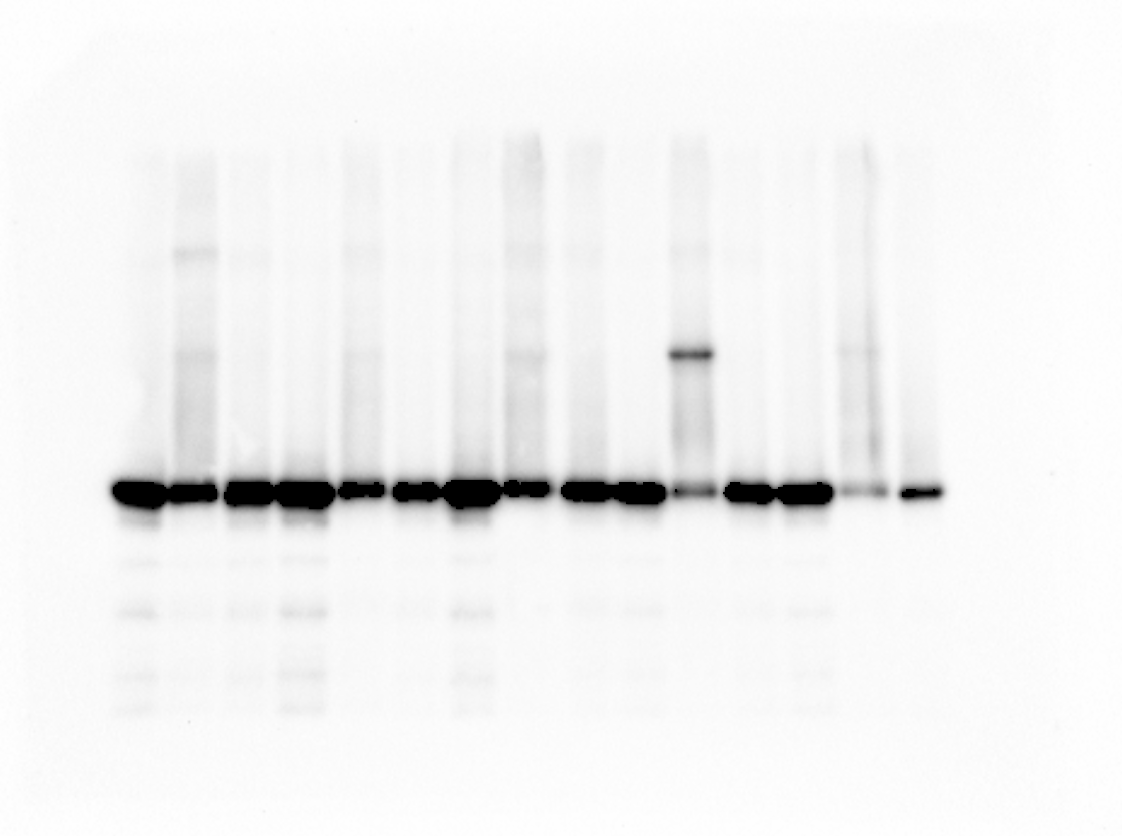

Supplement: Figure 6—figure supplement 5—source data 1. [file elife-74275-fig6-figsupp5-data1.zip › Figure 6-figure supplement 5-source data 1/figure supplement 5A/fig sup 5A anti-FLAG.tif]

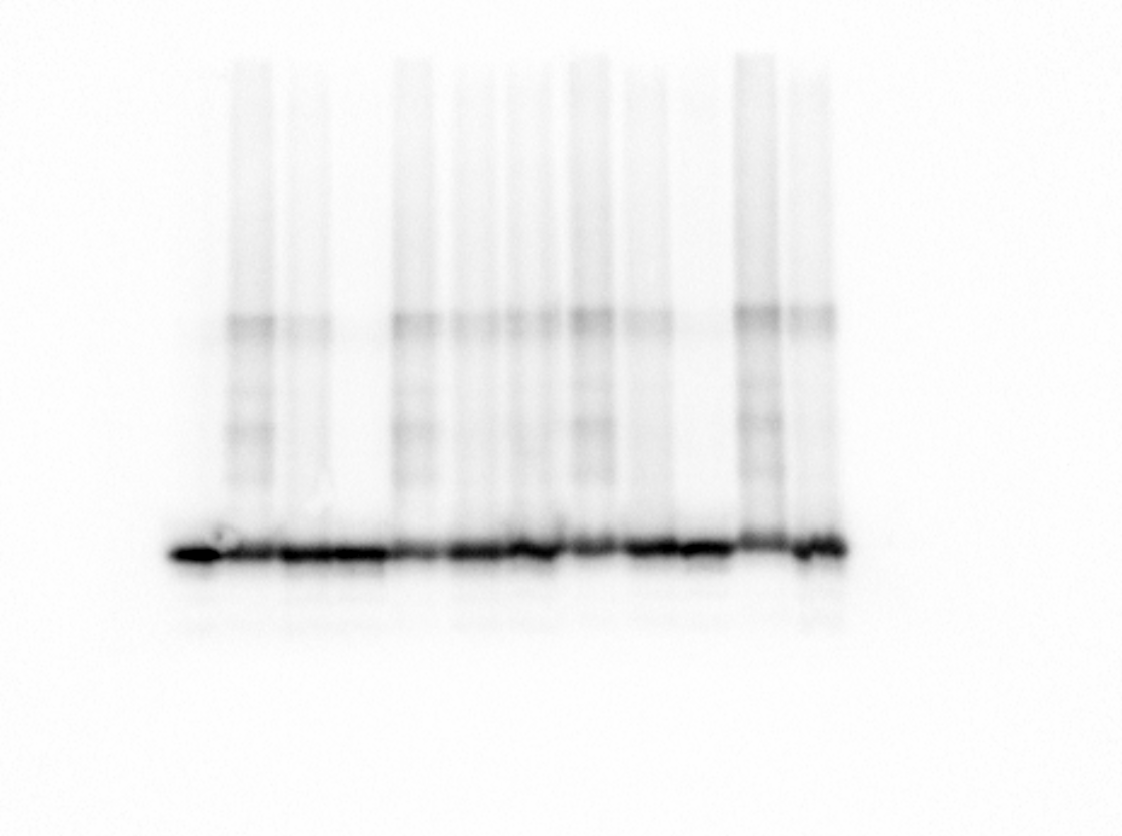

Supplement: Figure 6—figure supplement 5—source data 1. [file elife-74275-fig6-figsupp5-data1.zip › Figure 6-figure supplement 5-source data 1/figure supplement 5B/pSO114 anti-FLAG 15-60min Set1.tif]

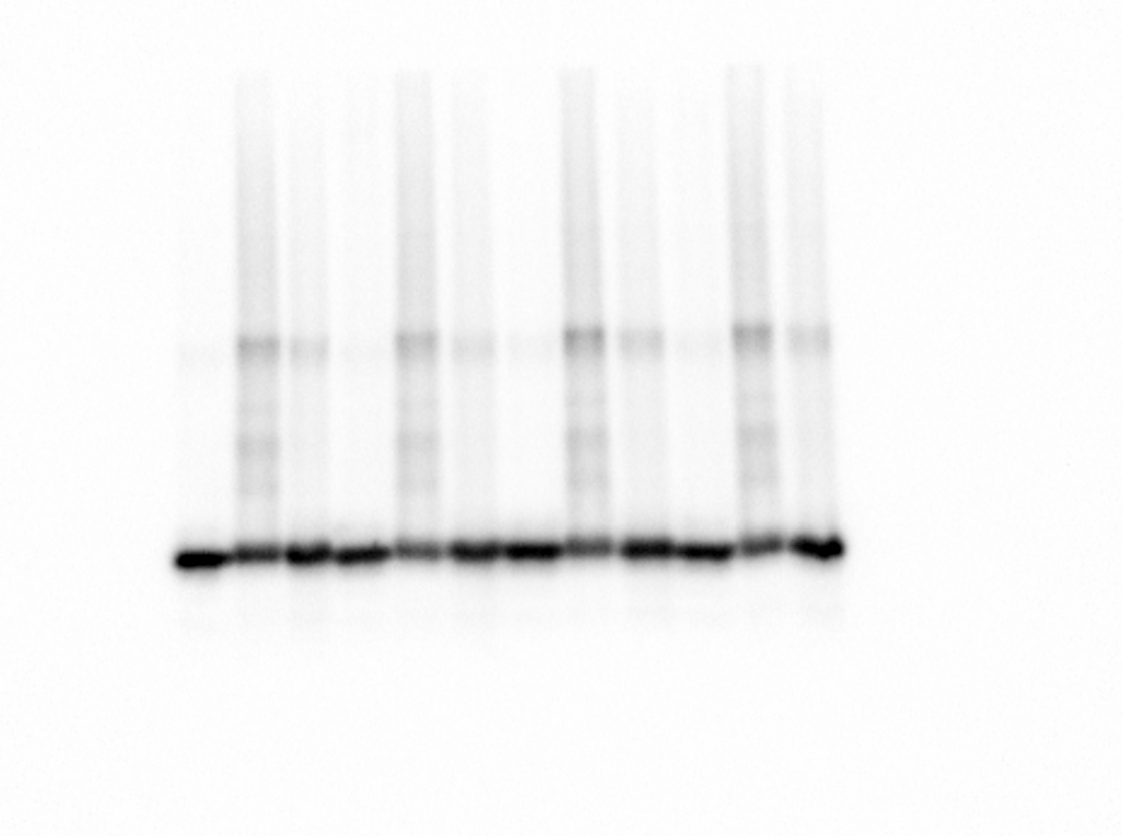

Supplement: Figure 6—figure supplement 5—source data 1. [file elife-74275-fig6-figsupp5-data1.zip › Figure 6-figure supplement 5-source data 1/figure supplement 5B/pSO114 anti-FLAG 15-60min Set2.tif]
