# Supplementary material for: Inhibitory proteins block substrate access by occupying the active site cleft of Bacillus subtilis intramembrane protease SpoIVFB
Source: eLife. 2022 Apr 26;11:e74275. doi: 10.7554/eLife.74275 (PMC9042235; doi:10.7554/eLife.74275)
Supplement: Figure 6—figure supplement 1—source data 2. [file elife-74275-fig6-figsupp1-data2.zip › Figure 6-figure supplement 1-source data 2/figure supplement 1B/fig sup 1B annotated blots.pptx]

## Slide 1
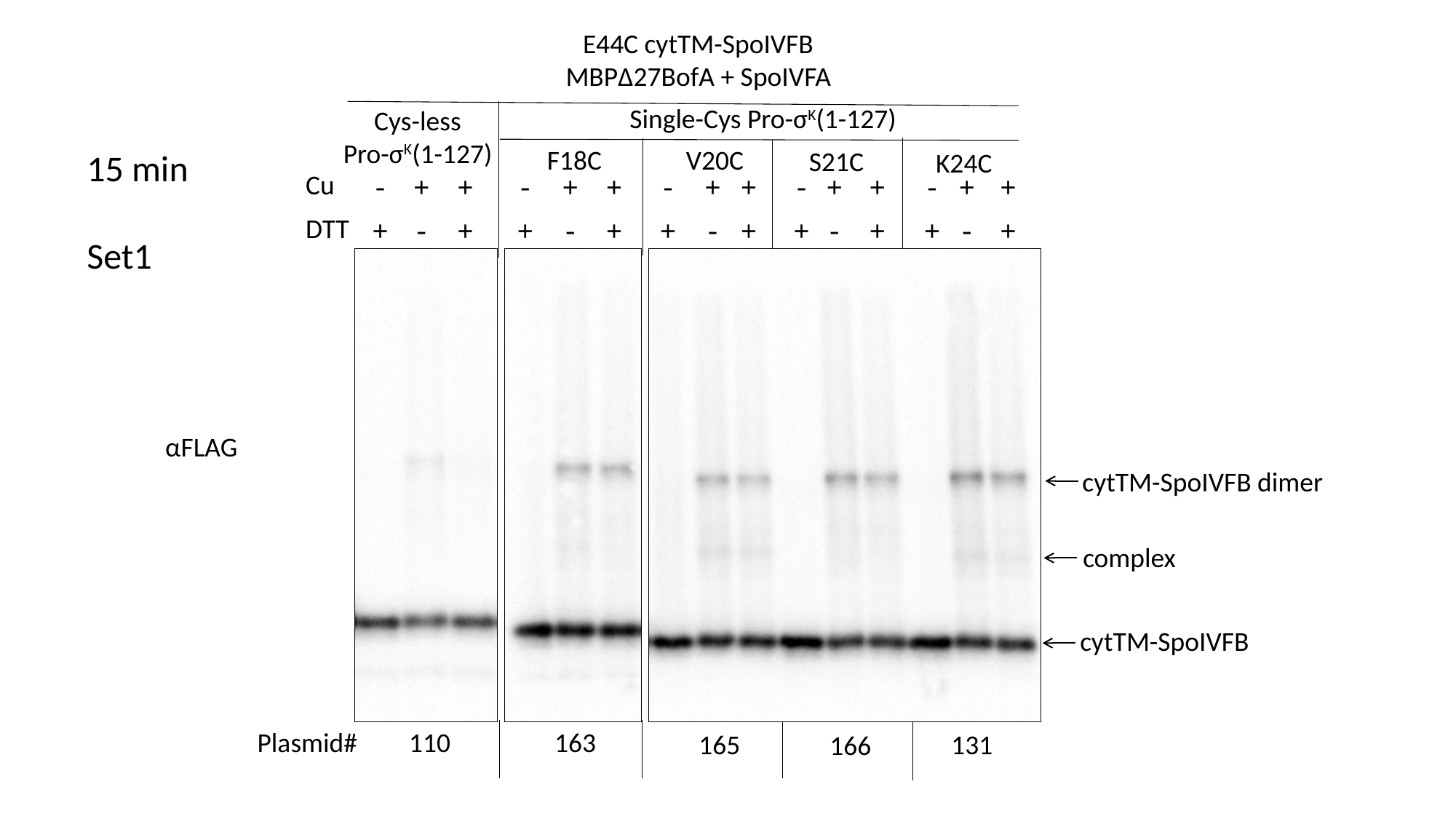

E44C cytTM-SpoIVFB
MBPΔ27BofA + SpoIVFA
Single-Cys Pro-σK(1-127)
Cys-less
Pro-σK(1-127)
F18C
V20C
S21C
15 min
Set1
K24C
| Cu | - | + | + | - | + | + | - | + | + | - | + | + | - | + | + |
| --- | --- | --- | --- | --- | --- | --- | --- | --- | --- | --- | --- | --- | --- | --- | --- |
| DTT | + | - | + | + | - | + | + | - | + | + | - | + | + | - | + |
αFLAG
cytTM-SpoIVFB dimer
complex
cytTM-SpoIVFB
Plasmid#
110
163
165
131
166

## Slide 2
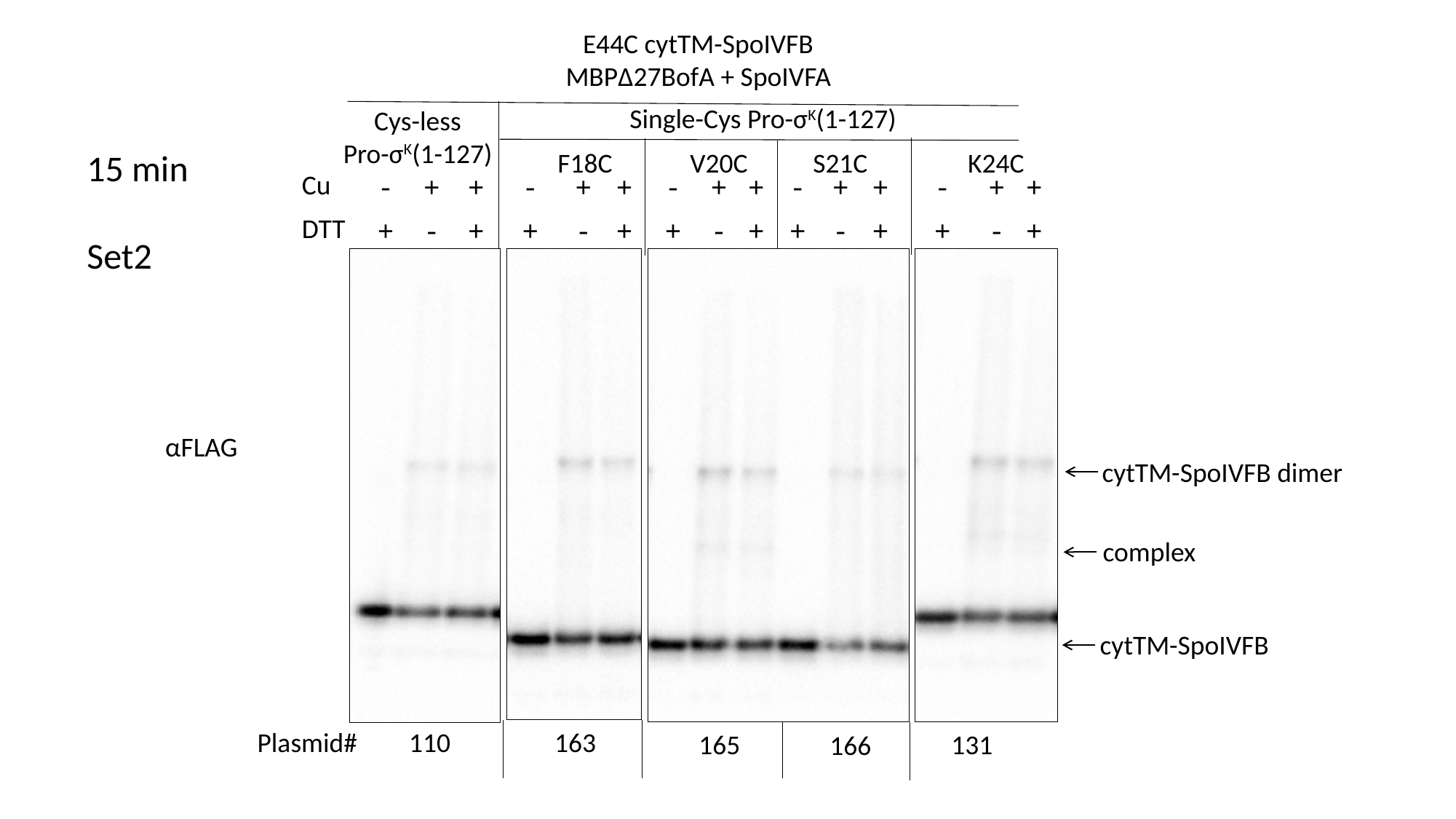

E44C cytTM-SpoIVFB
MBPΔ27BofA + SpoIVFA
Single-Cys Pro-σK(1-127)
Cys-less
Pro-σK(1-127)
15 min
Set2
F18C
V20C
S21C
K24C
| Cu | - | + | + | - | + | + | - | + | + | - | + | + | - | + | + |
| --- | --- | --- | --- | --- | --- | --- | --- | --- | --- | --- | --- | --- | --- | --- | --- |
| DTT | + | - | + | + | - | + | + | - | + | + | - | + | + | - | + |
αFLAG
cytTM-SpoIVFB dimer
complex
cytTM-SpoIVFB
Plasmid#
110
163
165
131
166

## Slide 3
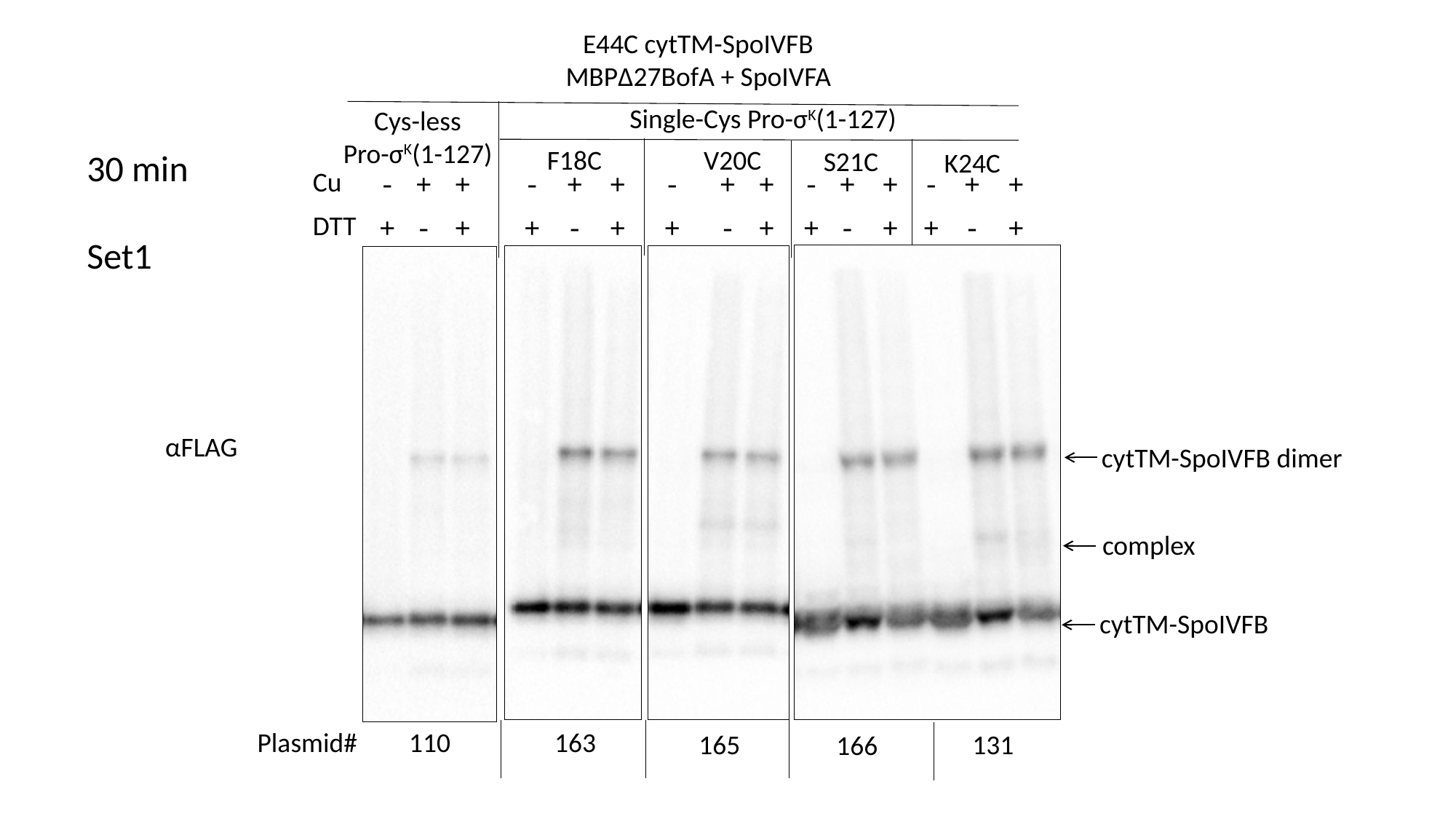

E44C cytTM-SpoIVFB
MBPΔ27BofA + SpoIVFA
Single-Cys Pro-σK(1-127)
Cys-less
Pro-σK(1-127)
F18C
V20C
S21C
30 min
Set1
K24C
| Cu | - | + | + | - | + | + | - | + | + | - | + | + | - | + | + |
| --- | --- | --- | --- | --- | --- | --- | --- | --- | --- | --- | --- | --- | --- | --- | --- |
| DTT | + | - | + | + | - | + | + | - | + | + | - | + | + | - | + |
αFLAG
cytTM-SpoIVFB dimer
complex
cytTM-SpoIVFB
Plasmid#
110
163
165
131
166

## Slide 4
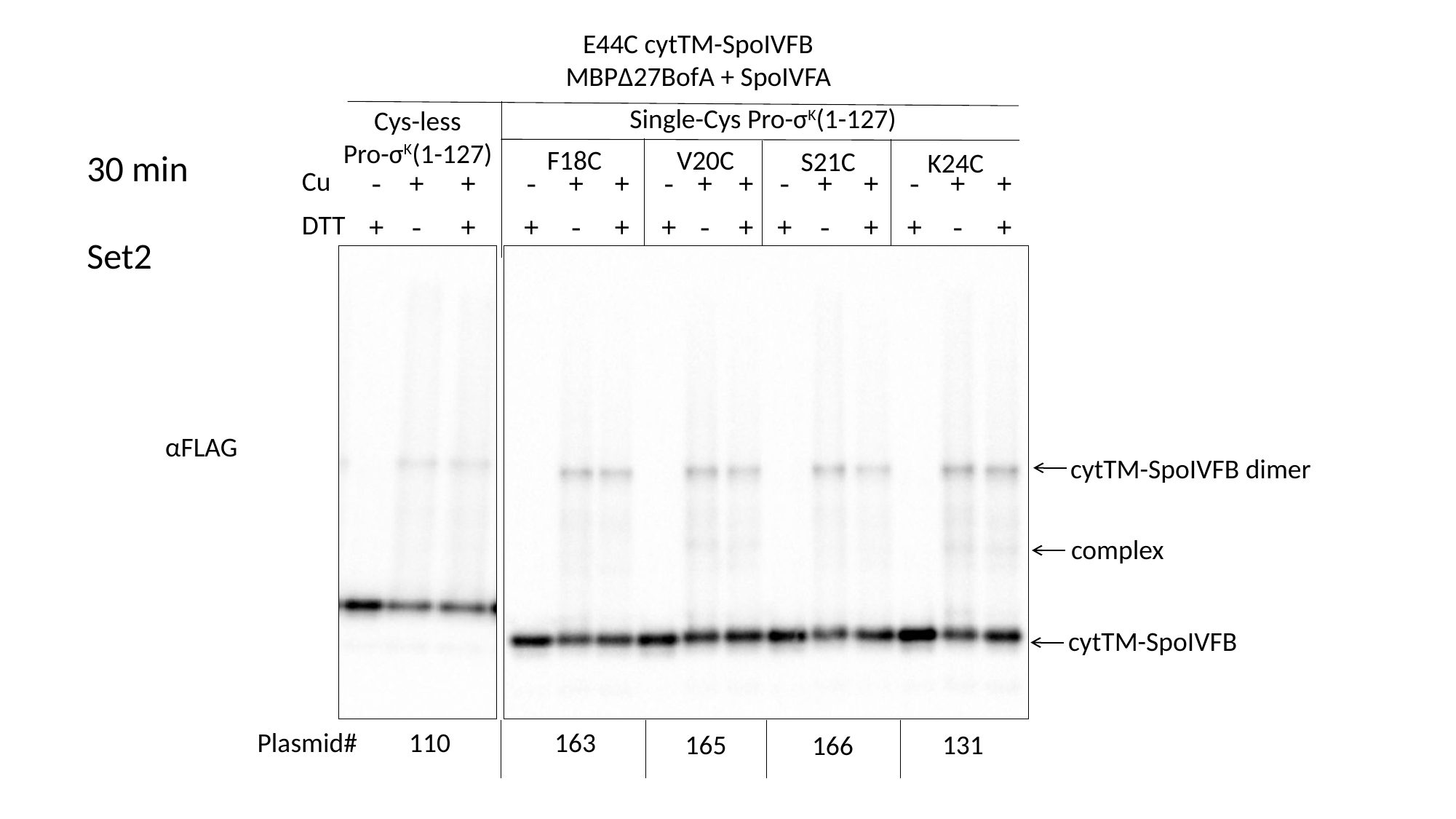

E44C cytTM-SpoIVFB
MBPΔ27BofA + SpoIVFA
Single-Cys Pro-σK(1-127)
Cys-less
Pro-σK(1-127)
F18C
V20C
S21C
30 min
Set2
K24C
| Cu | - | + | + | - | + | + | - | + | + | - | + | + | - | + | + |
| --- | --- | --- | --- | --- | --- | --- | --- | --- | --- | --- | --- | --- | --- | --- | --- |
| DTT | + | - | + | + | - | + | + | - | + | + | - | + | + | - | + |
αFLAG
cytTM-SpoIVFB dimer
complex
cytTM-SpoIVFB
Plasmid#
110
163
165
131
166

## Slide 5
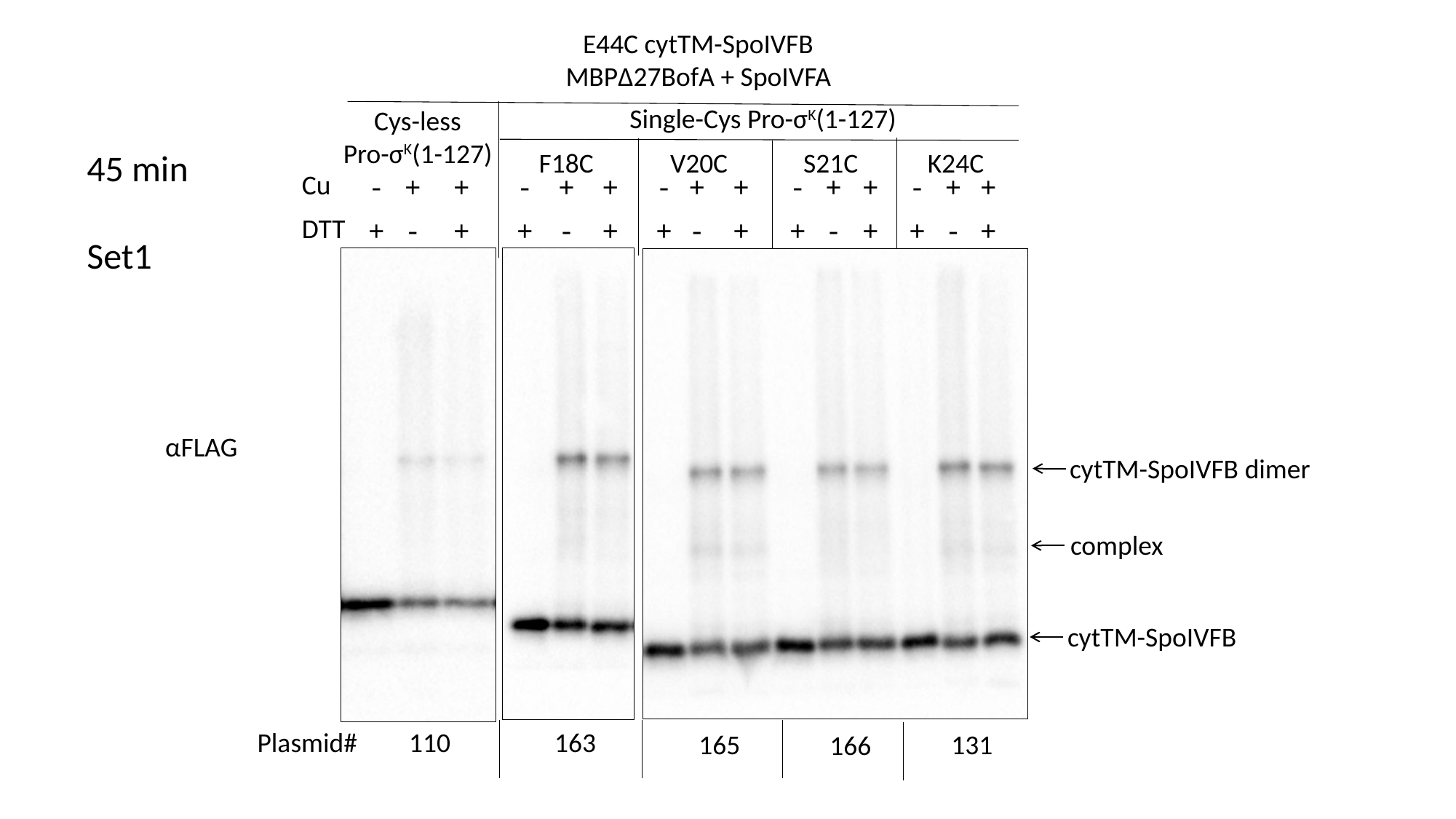

E44C cytTM-SpoIVFB
MBPΔ27BofA + SpoIVFA
Single-Cys Pro-σK(1-127)
Cys-less
Pro-σK(1-127)
45 min
Set1
F18C
V20C
S21C
K24C
| Cu | - | + | + | - | + | + | - | + | + | - | + | + | - | + | + |
| --- | --- | --- | --- | --- | --- | --- | --- | --- | --- | --- | --- | --- | --- | --- | --- |
| DTT | + | - | + | + | - | + | + | - | + | + | - | + | + | - | + |
αFLAG
cytTM-SpoIVFB dimer
complex
cytTM-SpoIVFB
Plasmid#
110
163
165
131
166

## Slide 6
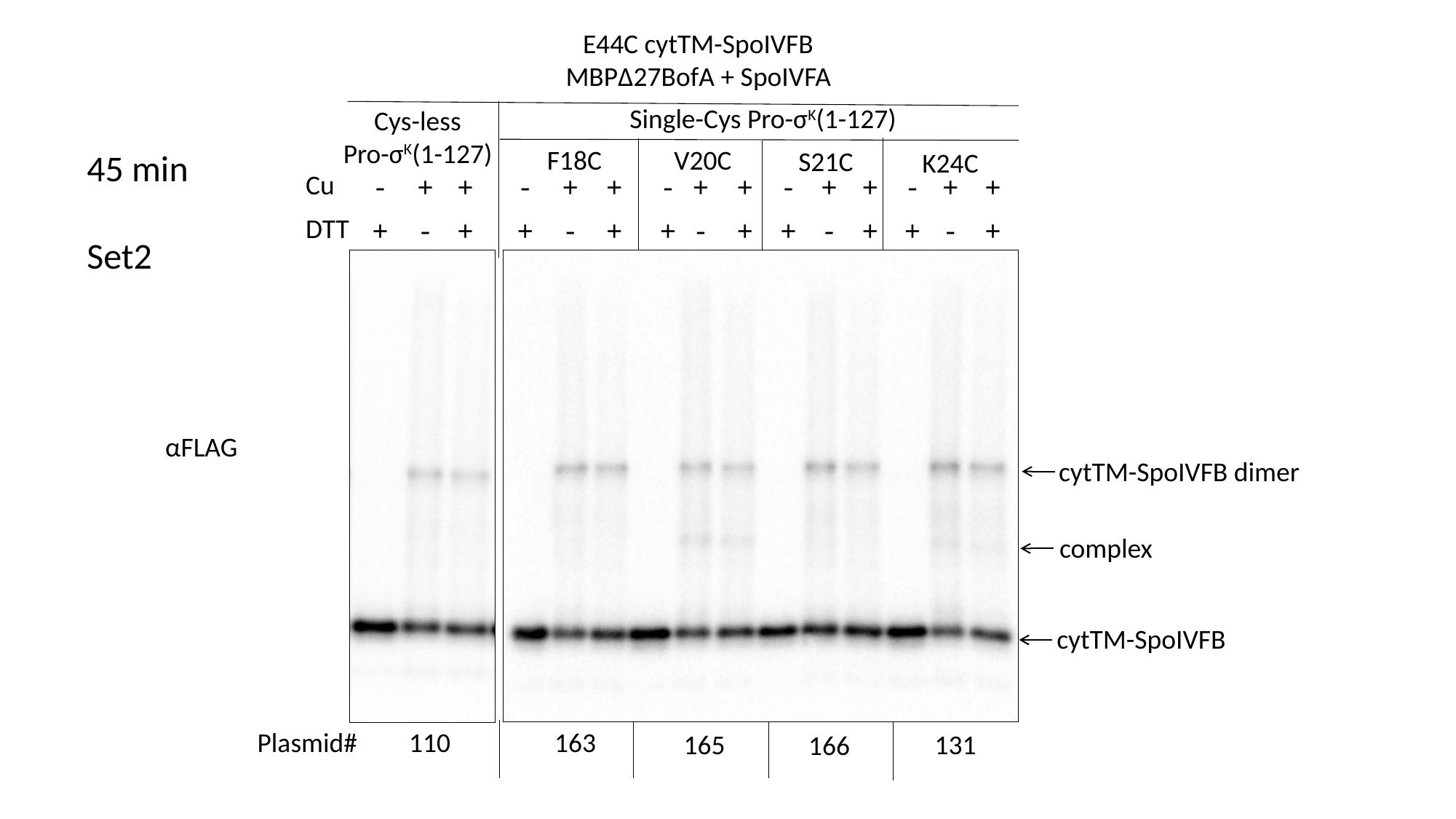

E44C cytTM-SpoIVFB
MBPΔ27BofA + SpoIVFA
Single-Cys Pro-σK(1-127)
Cys-less
Pro-σK(1-127)
F18C
V20C
S21C
45 min
Set2
K24C
| Cu | - | + | + | - | + | + | - | + | + | - | + | + | - | + | + |
| --- | --- | --- | --- | --- | --- | --- | --- | --- | --- | --- | --- | --- | --- | --- | --- |
| DTT | + | - | + | + | - | + | + | - | + | + | - | + | + | - | + |
αFLAG
cytTM-SpoIVFB dimer
complex
cytTM-SpoIVFB
Plasmid#
110
163
165
131
166

## Slide 7
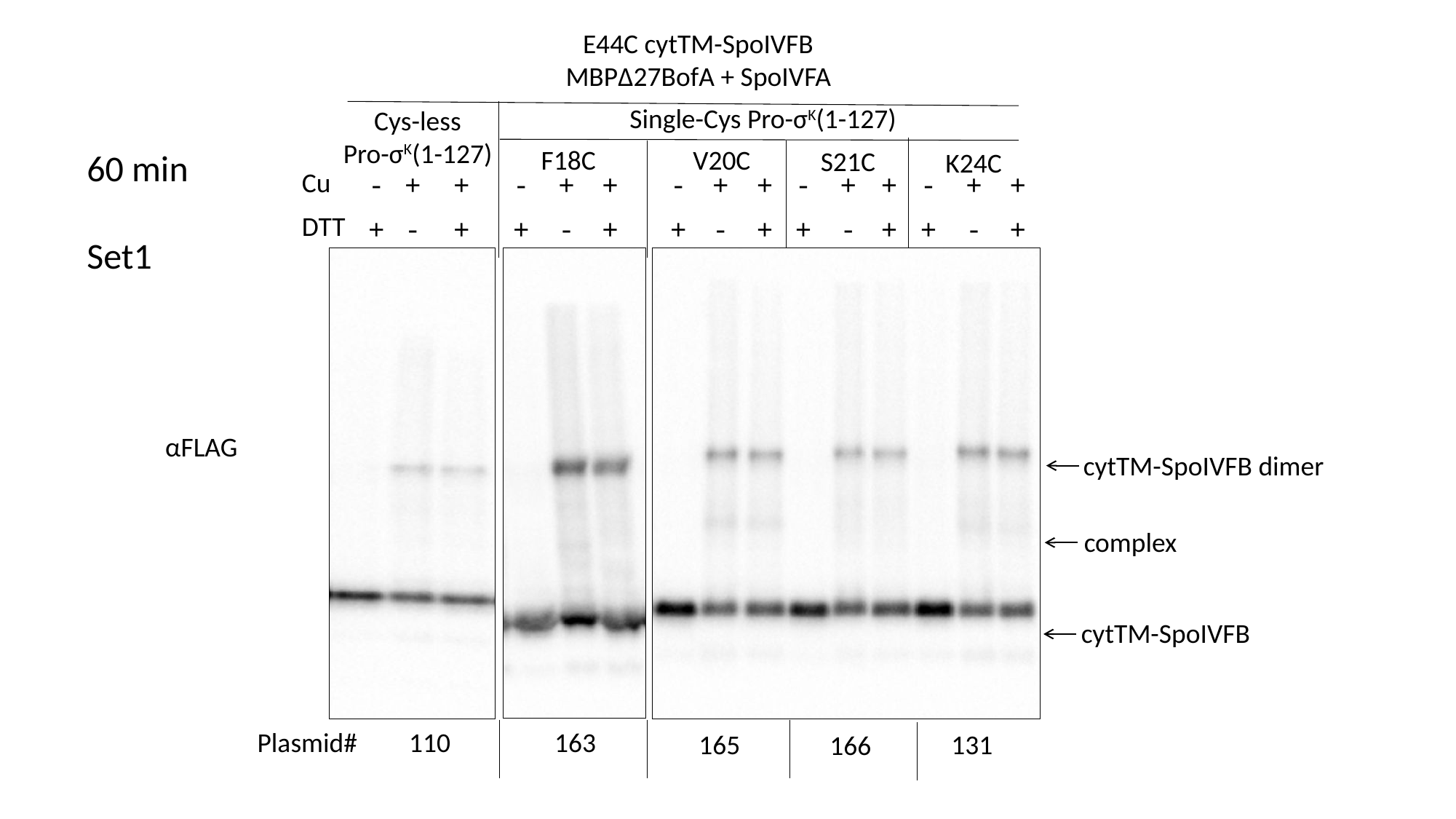

E44C cytTM-SpoIVFB
MBPΔ27BofA + SpoIVFA
Single-Cys Pro-σK(1-127)
Cys-less
Pro-σK(1-127)
F18C
V20C
S21C
60 min
Set1
K24C
| Cu | - | + | + | - | + | + | - | + | + | - | + | + | - | + | + |
| --- | --- | --- | --- | --- | --- | --- | --- | --- | --- | --- | --- | --- | --- | --- | --- |
| DTT | + | - | + | + | - | + | + | - | + | + | - | + | + | - | + |
αFLAG
cytTM-SpoIVFB dimer
complex
cytTM-SpoIVFB
Plasmid#
110
163
165
131
166

## Slide 8
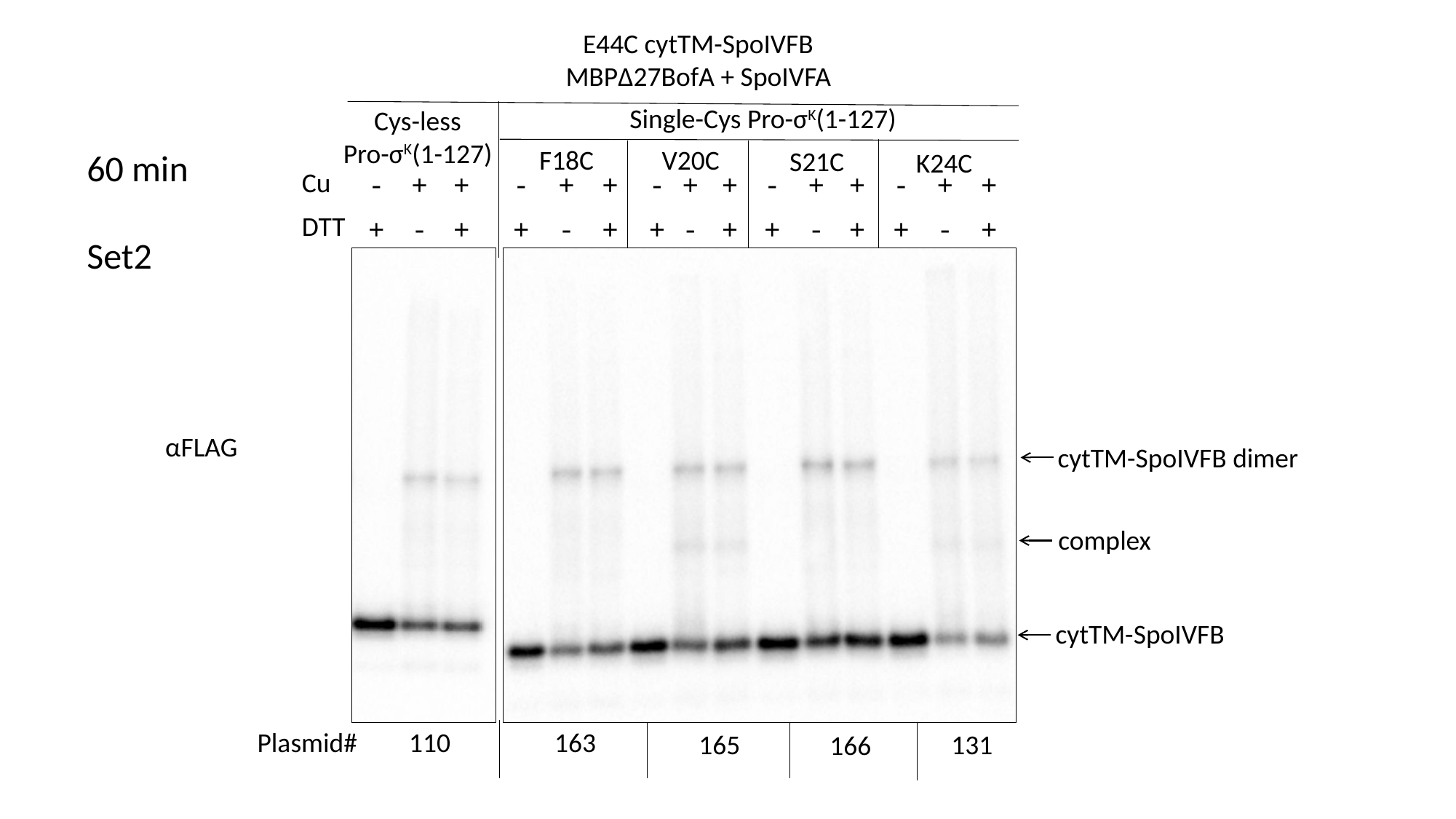

E44C cytTM-SpoIVFB
MBPΔ27BofA + SpoIVFA
Single-Cys Pro-σK(1-127)
Cys-less
Pro-σK(1-127)
F18C
V20C
S21C
60 min
Set2
K24C
| Cu | - | + | + | - | + | + | - | + | + | - | + | + | - | + | + |
| --- | --- | --- | --- | --- | --- | --- | --- | --- | --- | --- | --- | --- | --- | --- | --- |
| DTT | + | - | + | + | - | + | + | - | + | + | - | + | + | - | + |
αFLAG
cytTM-SpoIVFB dimer
complex
cytTM-SpoIVFB
Plasmid#
110
163
165
131
166
