# Supplementary material for: Inhibitory proteins block substrate access by occupying the active site cleft of Bacillus subtilis intramembrane protease SpoIVFB
Source: eLife. 2022 Apr 26;11:e74275. doi: 10.7554/eLife.74275 (PMC9042235; doi:10.7554/eLife.74275)
Supplement: Figure 6—figure supplement 3—source data 1. [file elife-74275-fig6-figsupp3-data1.zip › Figure 6-figure supplement 3-source data 1/fig sup 3G annotated blot.pptx]

## Slide 1
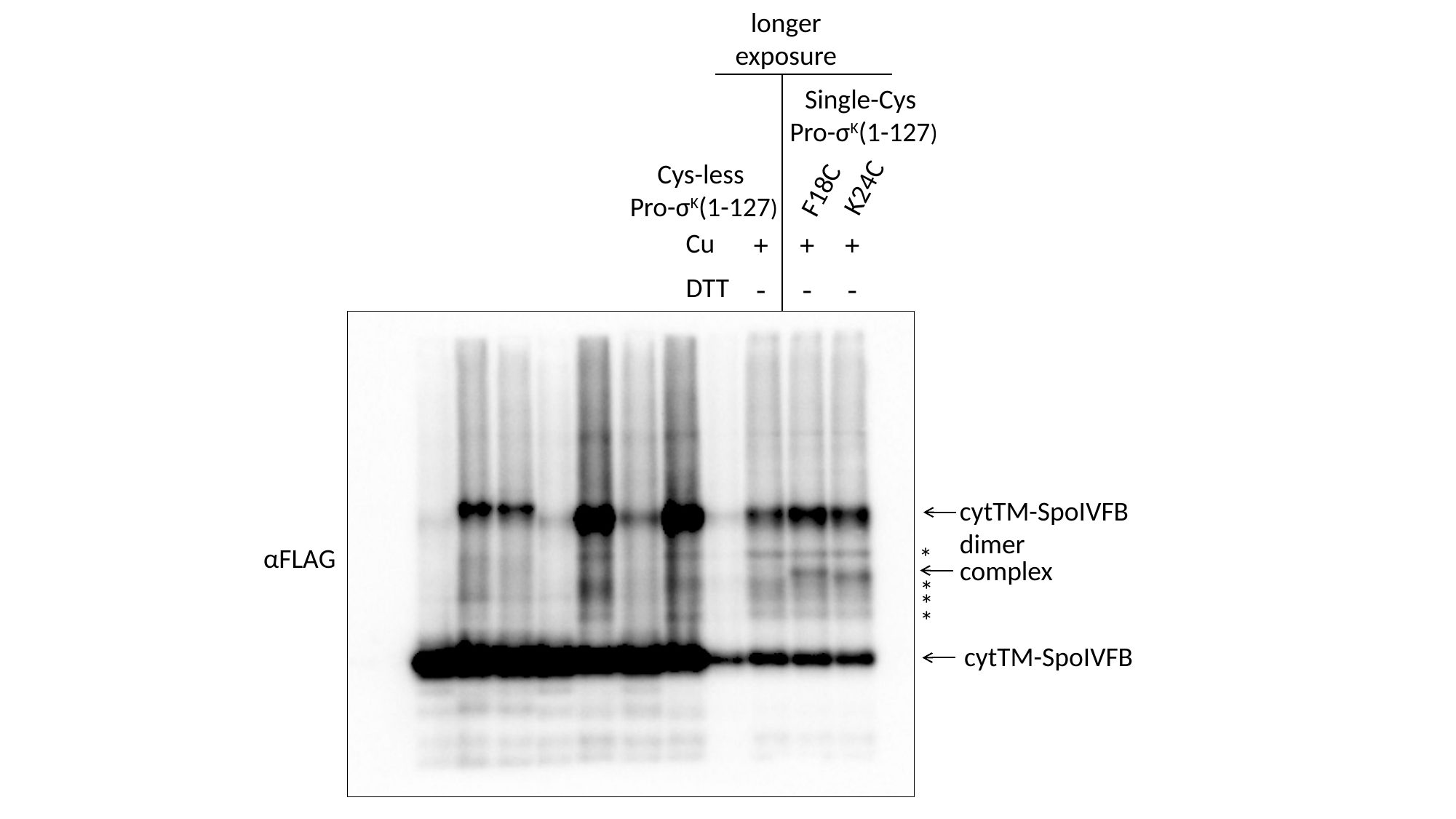

longer
exposure
Single-Cys
Pro-σK(1-127)
Cys-less
Pro-σK(1-127)
K24C
F18C
| Cu | + | + | + |
| --- | --- | --- | --- |
| DTT | - | - | - |
cytTM-SpoIVFB dimer
*
αFLAG
complex
*
*
*
 cytTM-SpoIVFB
