# Supplementary material for: Inhibitory proteins block substrate access by occupying the active site cleft of Bacillus subtilis intramembrane protease SpoIVFB
Source: eLife. 2022 Apr 26;11:e74275. doi: 10.7554/eLife.74275 (PMC9042235; doi:10.7554/eLife.74275)
Supplement: Figure 6—figure supplement 3—source data 1. [file elife-74275-fig6-figsupp3-data1.zip › Figure 6-figure supplement 3-source data 1/figure supplement 3C/readme.docx]

Some of the immunoblot images (raw) for experiments with the following plasmids can be found in Figure 6-figure supplement 1-source data 1 and 2: pSO164, pSO132, and pSO111.
