# Supplementary material for: Inhibitory proteins block substrate access by occupying the active site cleft of Bacillus subtilis intramembrane protease SpoIVFB
Source: eLife. 2022 Apr 26;11:e74275. doi: 10.7554/eLife.74275 (PMC9042235; doi:10.7554/eLife.74275)
Supplement: Figure 6—figure supplement 3—source data 1. [file elife-74275-fig6-figsupp3-data1.zip › Figure 6-figure supplement 3-source data 1/figure supplement 3E/fig sup 3E annotated blots.pptx]

## Slide 1
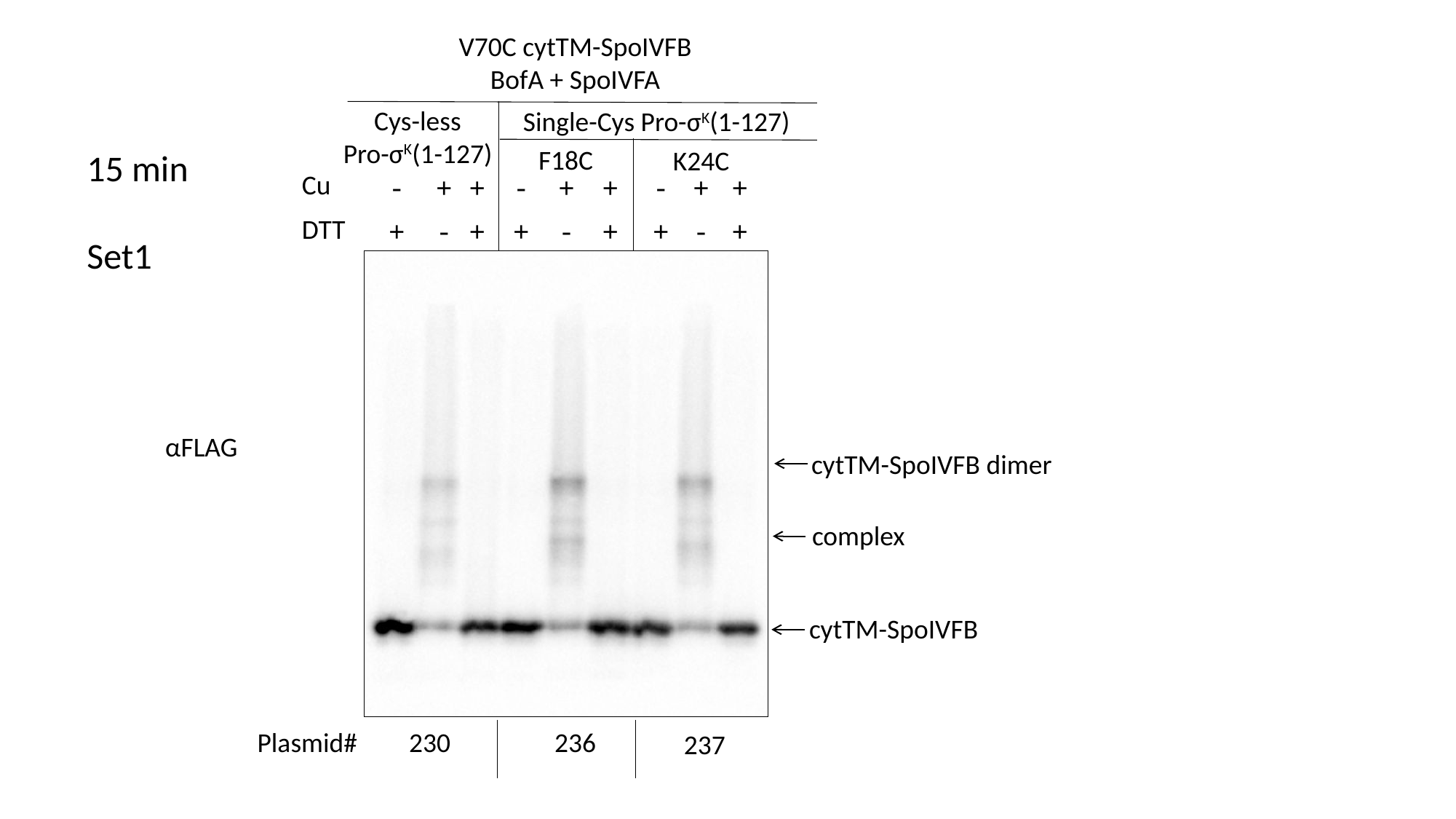

V70C cytTM-SpoIVFB
BofA + SpoIVFA
Cys-less
Pro-σK(1-127)
Single-Cys Pro-σK(1-127)
F18C
K24C
15 min
Set1
| Cu | - | + | + | - | + | + | - | + | + | | | | | | |
| --- | --- | --- | --- | --- | --- | --- | --- | --- | --- | --- | --- | --- | --- | --- | --- |
| DTT | + | - | + | + | - | + | + | - | + | | | | | | |
αFLAG
cytTM-SpoIVFB dimer
complex
cytTM-SpoIVFB
Plasmid#
230
236
237

## Slide 2
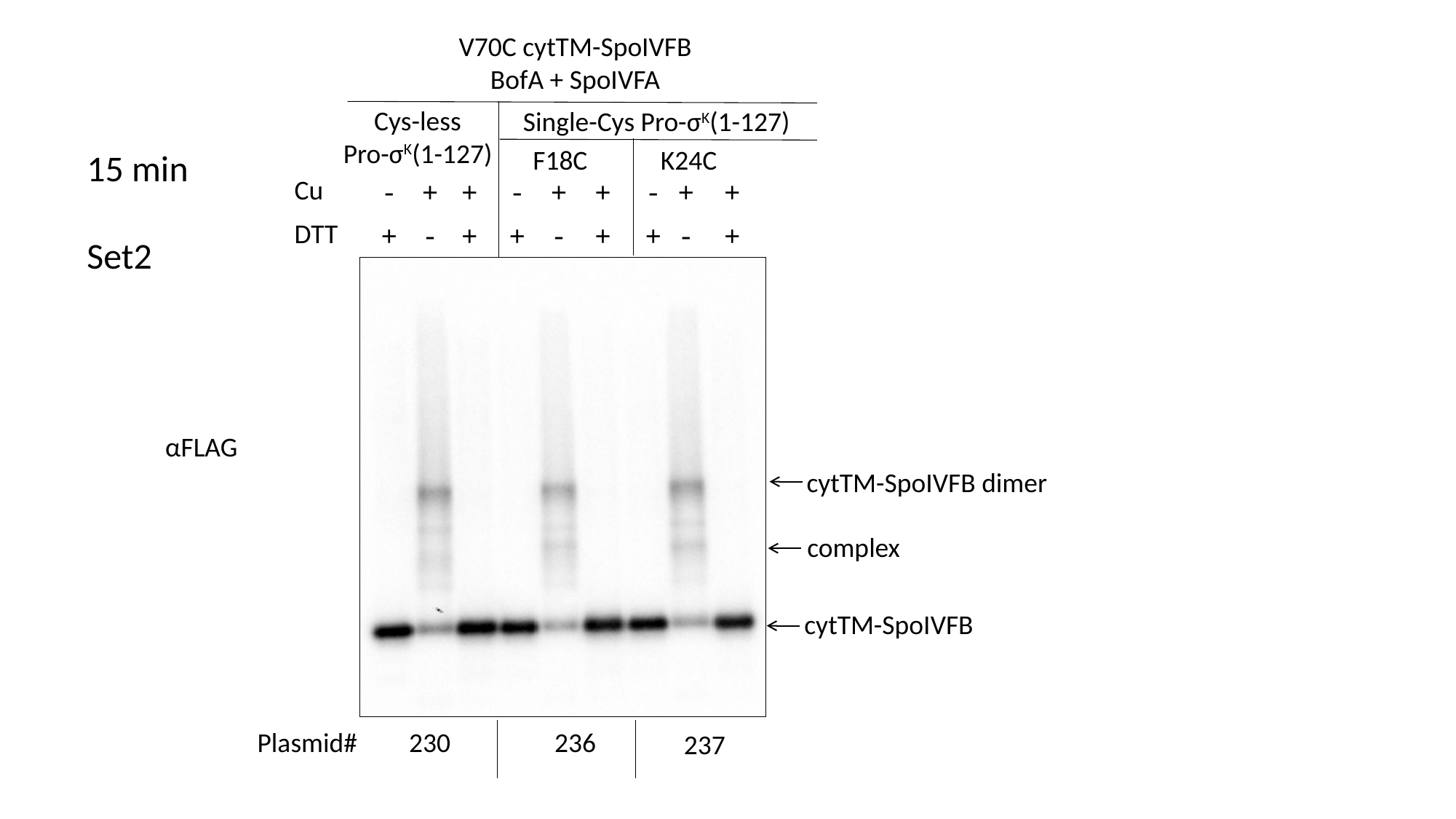

V70C cytTM-SpoIVFB
BofA + SpoIVFA
Cys-less
Pro-σK(1-127)
Single-Cys Pro-σK(1-127)
F18C
K24C
15 min
Set2
| Cu | - | + | + | - | + | + | - | + | + | | | | | | |
| --- | --- | --- | --- | --- | --- | --- | --- | --- | --- | --- | --- | --- | --- | --- | --- |
| DTT | + | - | + | + | - | + | + | - | + | | | | | | |
αFLAG
cytTM-SpoIVFB dimer
complex
cytTM-SpoIVFB
Plasmid#
230
236
237

## Slide 3
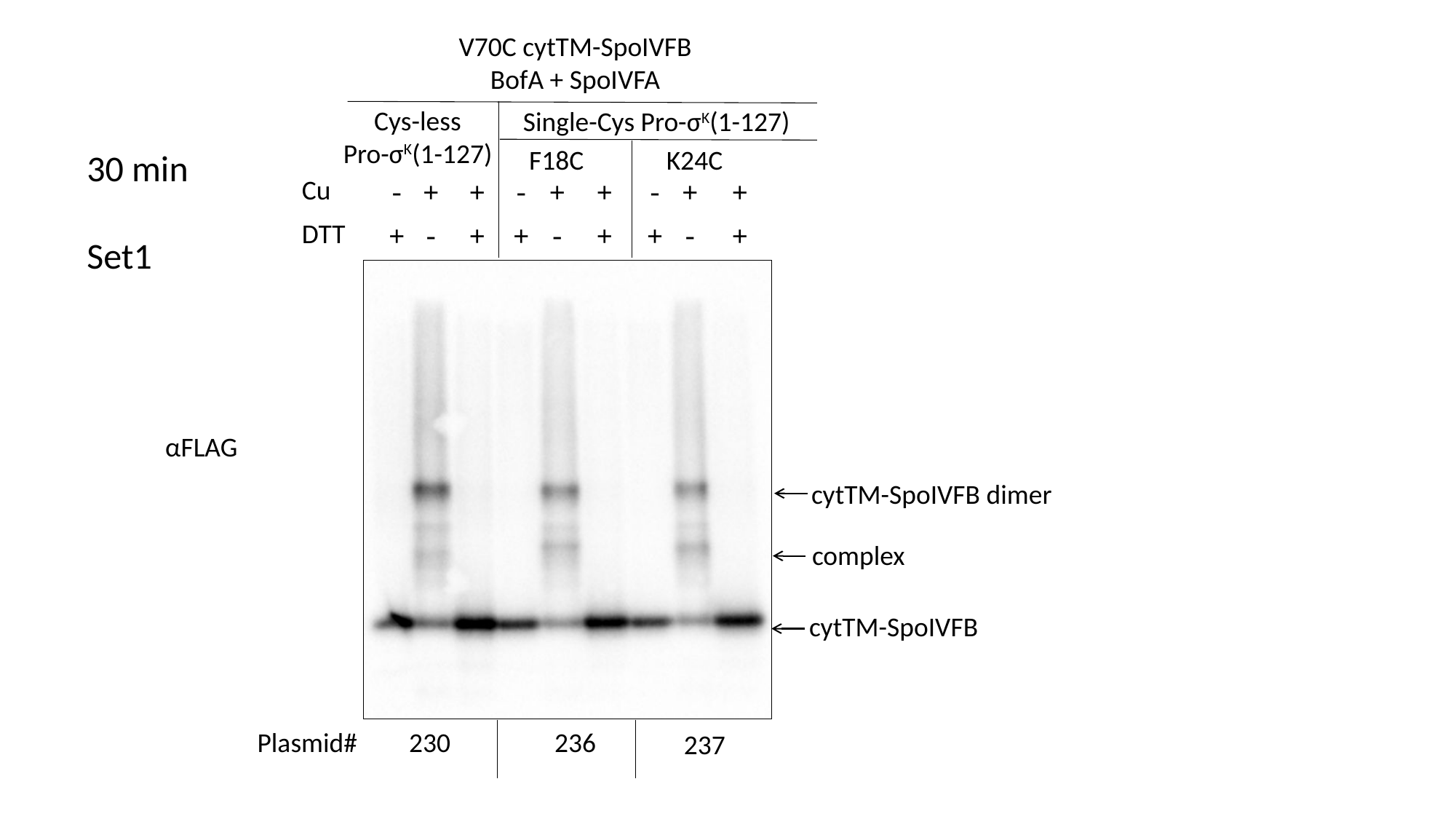

V70C cytTM-SpoIVFB
BofA + SpoIVFA
Cys-less
Pro-σK(1-127)
Single-Cys Pro-σK(1-127)
F18C
K24C
30 min
Set1
| Cu | - | + | + | - | + | + | - | + | + | | | | | | |
| --- | --- | --- | --- | --- | --- | --- | --- | --- | --- | --- | --- | --- | --- | --- | --- |
| DTT | + | - | + | + | - | + | + | - | + | | | | | | |
αFLAG
cytTM-SpoIVFB dimer
complex
cytTM-SpoIVFB
Plasmid#
230
236
237

## Slide 4
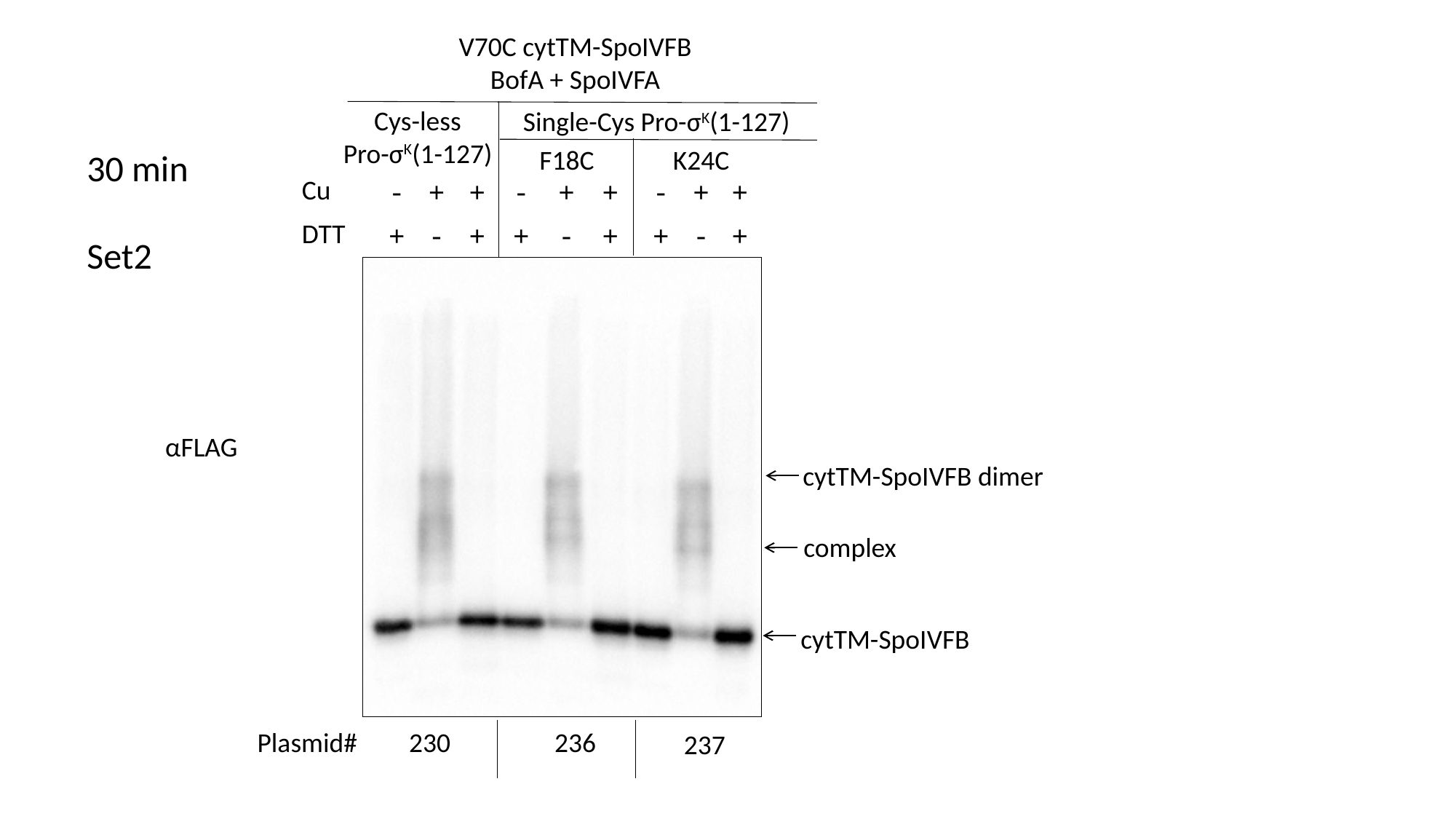

V70C cytTM-SpoIVFB
BofA + SpoIVFA
Cys-less
Pro-σK(1-127)
Single-Cys Pro-σK(1-127)
F18C
K24C
30 min
Set2
| Cu | - | + | + | - | + | + | - | + | + | | | | | | |
| --- | --- | --- | --- | --- | --- | --- | --- | --- | --- | --- | --- | --- | --- | --- | --- |
| DTT | + | - | + | + | - | + | + | - | + | | | | | | |
αFLAG
cytTM-SpoIVFB dimer
complex
cytTM-SpoIVFB
Plasmid#
230
236
237

## Slide 5
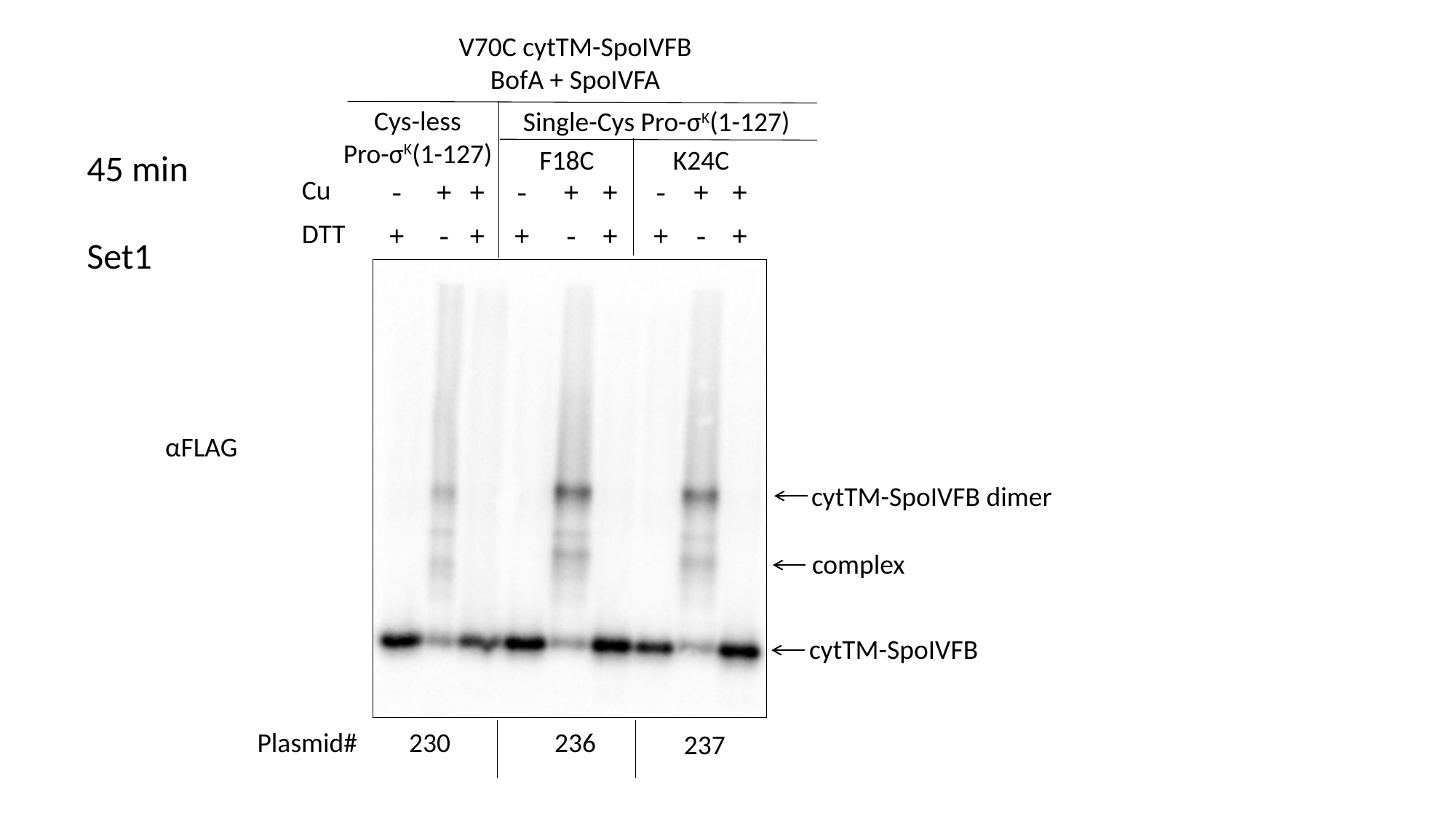

V70C cytTM-SpoIVFB
BofA + SpoIVFA
Cys-less
Pro-σK(1-127)
Single-Cys Pro-σK(1-127)
F18C
K24C
45 min
Set1
| Cu | - | + | + | - | + | + | - | + | + | | | | | | |
| --- | --- | --- | --- | --- | --- | --- | --- | --- | --- | --- | --- | --- | --- | --- | --- |
| DTT | + | - | + | + | - | + | + | - | + | | | | | | |
αFLAG
cytTM-SpoIVFB dimer
complex
cytTM-SpoIVFB
Plasmid#
230
236
237

## Slide 6
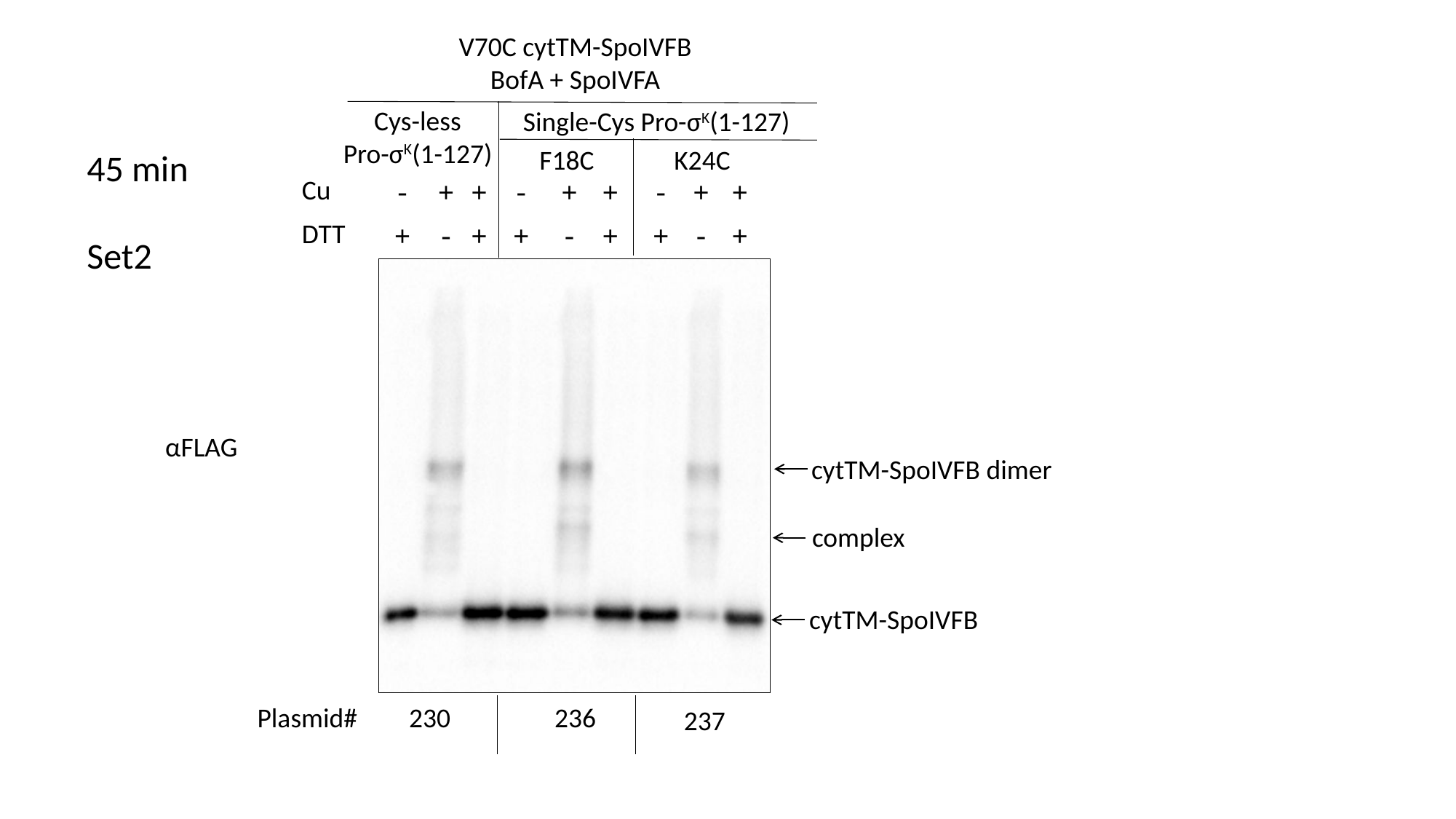

V70C cytTM-SpoIVFB
BofA + SpoIVFA
Cys-less
Pro-σK(1-127)
Single-Cys Pro-σK(1-127)
F18C
K24C
45 min
Set2
| Cu | - | + | + | - | + | + | - | + | + | | | | | | |
| --- | --- | --- | --- | --- | --- | --- | --- | --- | --- | --- | --- | --- | --- | --- | --- |
| DTT | + | - | + | + | - | + | + | - | + | | | | | | |
αFLAG
cytTM-SpoIVFB dimer
complex
cytTM-SpoIVFB
Plasmid#
230
236
237

## Slide 7
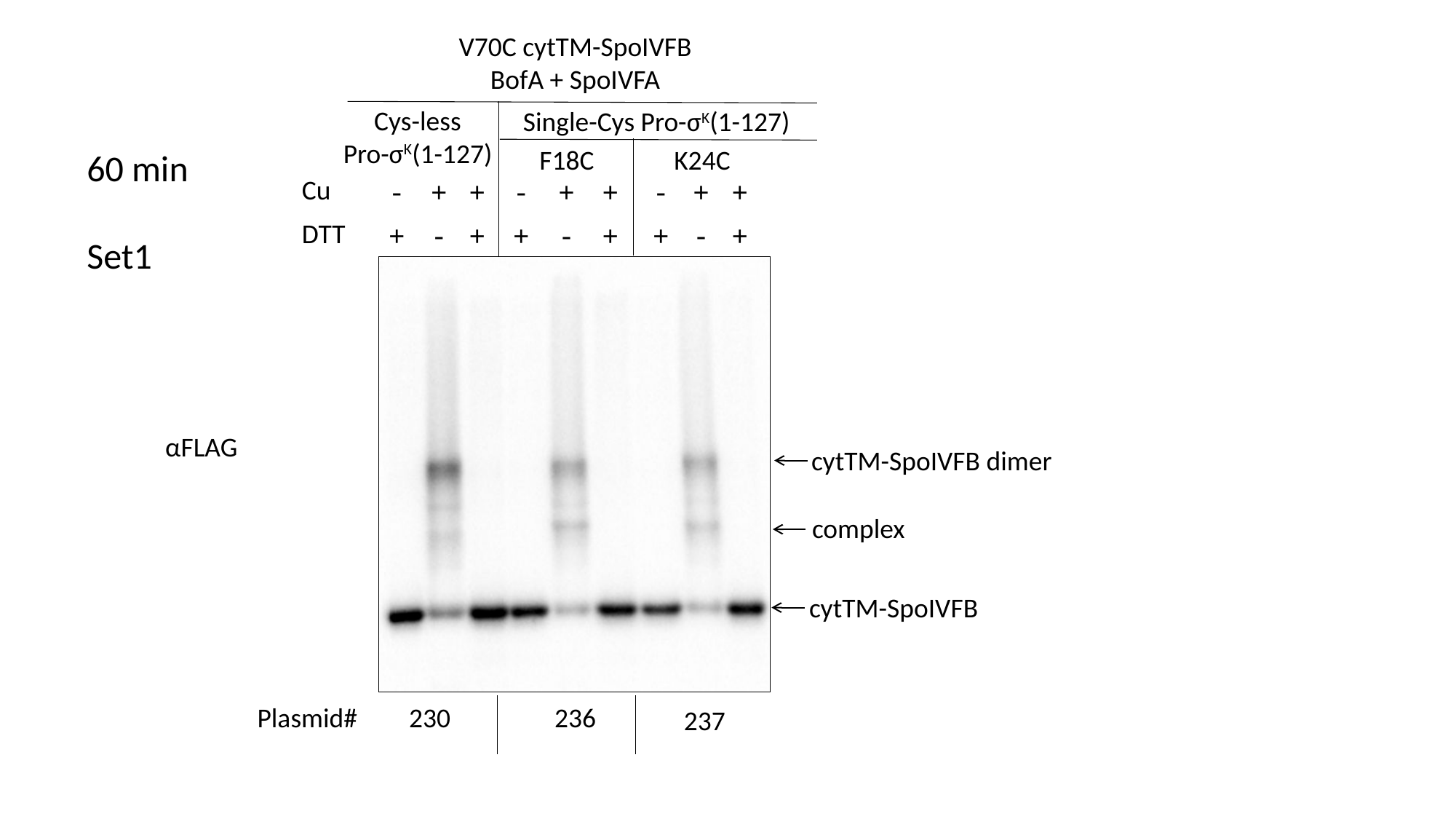

V70C cytTM-SpoIVFB
BofA + SpoIVFA
Cys-less
Pro-σK(1-127)
Single-Cys Pro-σK(1-127)
F18C
K24C
60 min
Set1
| Cu | - | + | + | - | + | + | - | + | + | | | | | | |
| --- | --- | --- | --- | --- | --- | --- | --- | --- | --- | --- | --- | --- | --- | --- | --- |
| DTT | + | - | + | + | - | + | + | - | + | | | | | | |
αFLAG
cytTM-SpoIVFB dimer
complex
cytTM-SpoIVFB
Plasmid#
230
236
237

## Slide 8
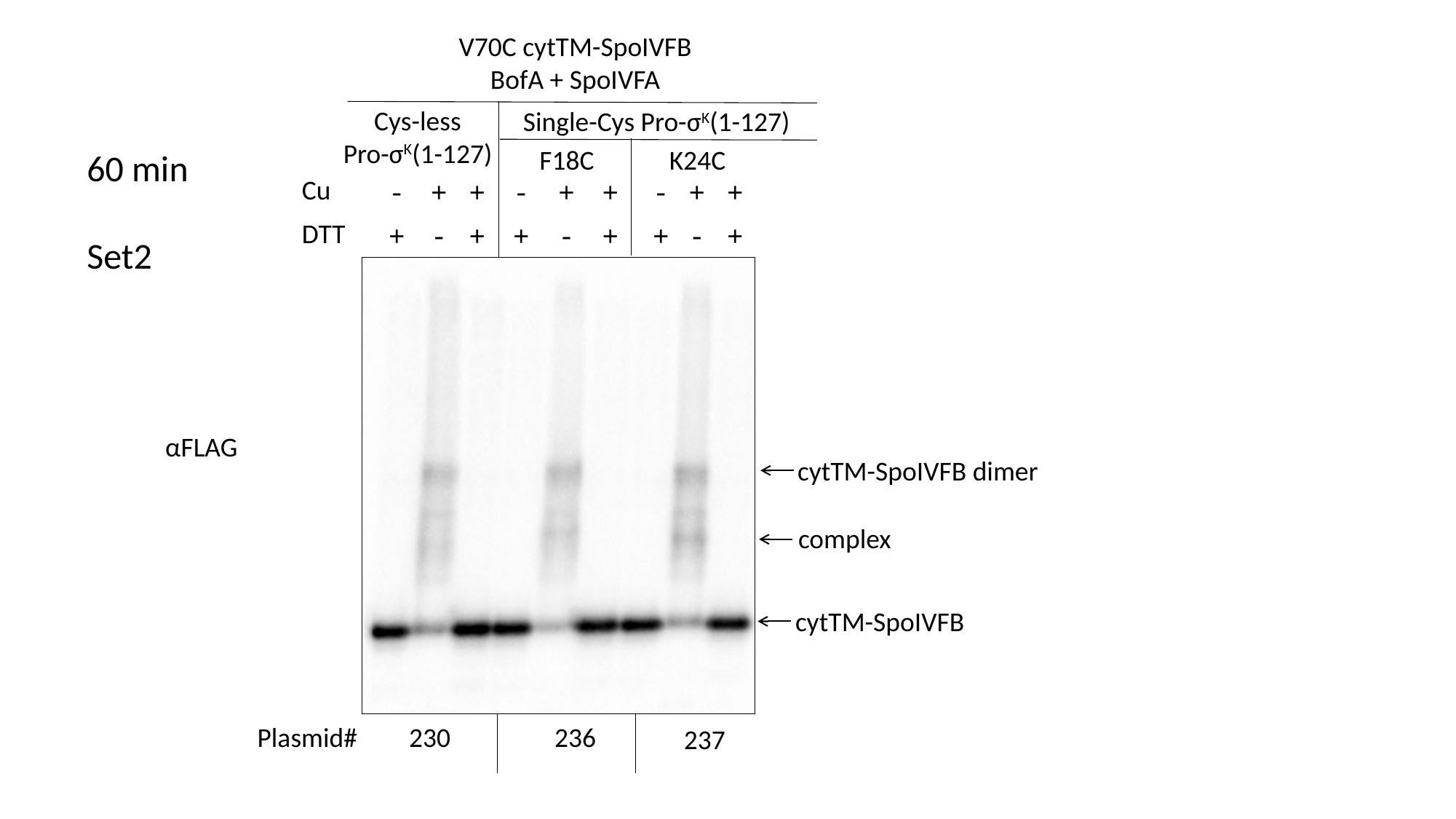

V70C cytTM-SpoIVFB
BofA + SpoIVFA
Cys-less
Pro-σK(1-127)
Single-Cys Pro-σK(1-127)
F18C
K24C
60 min
Set2
| Cu | - | + | + | - | + | + | - | + | + | | | | | | |
| --- | --- | --- | --- | --- | --- | --- | --- | --- | --- | --- | --- | --- | --- | --- | --- |
| DTT | + | - | + | + | - | + | + | - | + | | | | | | |
αFLAG
cytTM-SpoIVFB dimer
complex
cytTM-SpoIVFB
Plasmid#
230
236
237
