# Supplementary material for: Inhibitory proteins block substrate access by occupying the active site cleft of Bacillus subtilis intramembrane protease SpoIVFB
Source: eLife. 2022 Apr 26;11:e74275. doi: 10.7554/eLife.74275 (PMC9042235; doi:10.7554/eLife.74275)
Supplement: Figure 6—figure supplement 4—source data 1. [file elife-74275-fig6-figsupp4-data1.zip › Figure 6-figure supplement 4-source data 1/fig sup 4 annotated blots.pptx]

## Slide 1
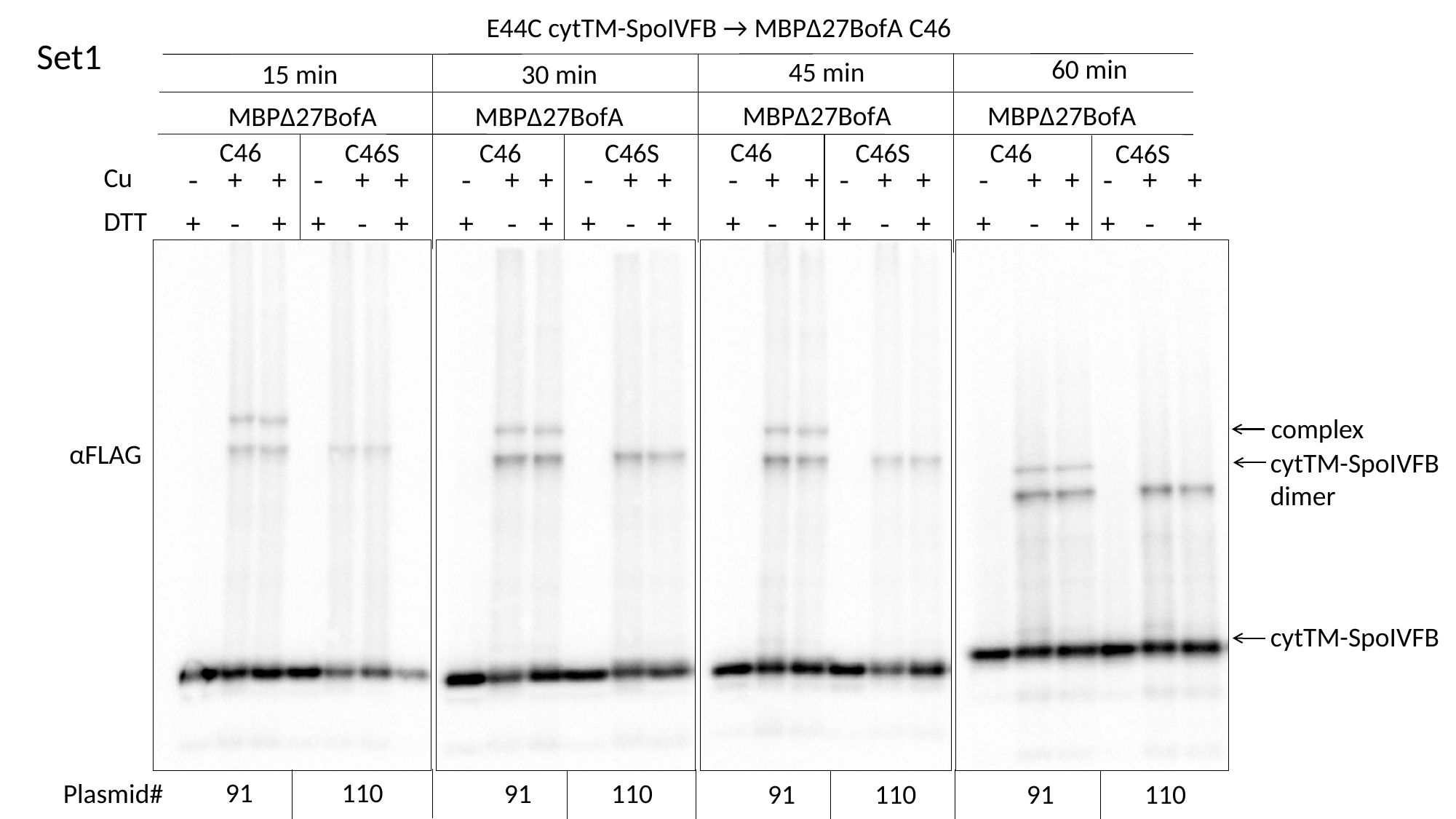

E44C cytTM-SpoIVFB → MBPΔ27BofA C46
Set1
60 min
45 min
30 min
15 min
MBPΔ27BofA
MBPΔ27BofA
MBPΔ27BofA
MBPΔ27BofA
C46
C46
C46S
C46
C46S
C46
C46S
C46S
| Cu | - | + | + | - | + | + | - | + | + | - | + | + | - | + | + | - | + | + | - | + | + | - | + | + |
| --- | --- | --- | --- | --- | --- | --- | --- | --- | --- | --- | --- | --- | --- | --- | --- | --- | --- | --- | --- | --- | --- | --- | --- | --- |
| DTT | + | - | + | + | - | + | + | - | + | + | - | + | + | - | + | + | - | + | + | - | + | + | - | + |
complex
αFLAG
cytTM-SpoIVFB
dimer
cytTM-SpoIVFB
91
110
Plasmid#
91
110
91
110
91
110

## Slide 2
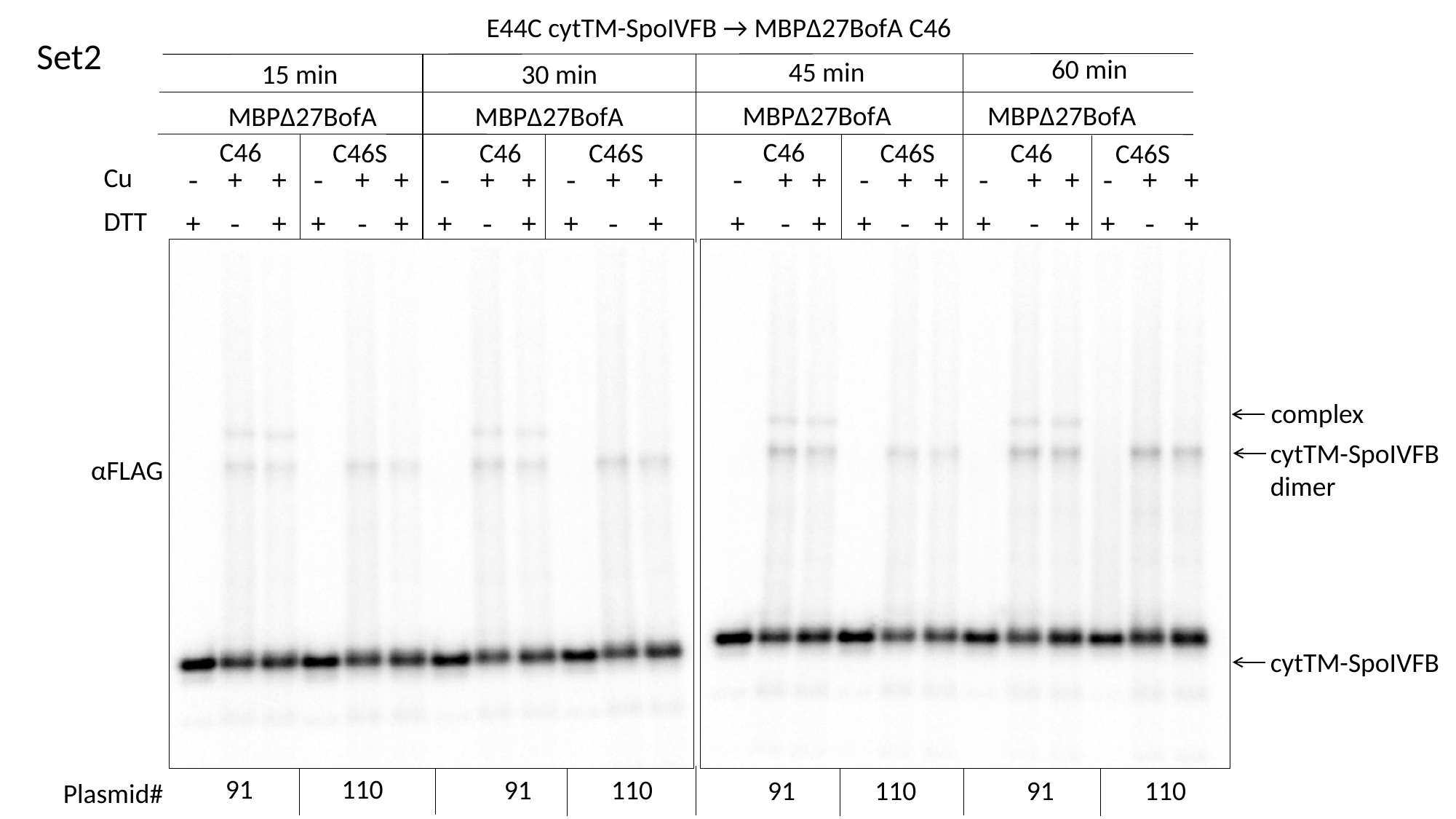

E44C cytTM-SpoIVFB → MBPΔ27BofA C46
Set2
60 min
45 min
30 min
15 min
MBPΔ27BofA
MBPΔ27BofA
MBPΔ27BofA
MBPΔ27BofA
C46
C46
C46S
C46
C46S
C46
C46S
C46S
| Cu | - | + | + | - | + | + | - | + | + | - | + | + | - | + | + | - | + | + | - | + | + | - | + | + |
| --- | --- | --- | --- | --- | --- | --- | --- | --- | --- | --- | --- | --- | --- | --- | --- | --- | --- | --- | --- | --- | --- | --- | --- | --- |
| DTT | + | - | + | + | - | + | + | - | + | + | - | + | + | - | + | + | - | + | + | - | + | + | - | + |
complex
cytTM-SpoIVFB
dimer
αFLAG
cytTM-SpoIVFB
91
110
91
110
91
110
91
110
Plasmid#

## Slide 3
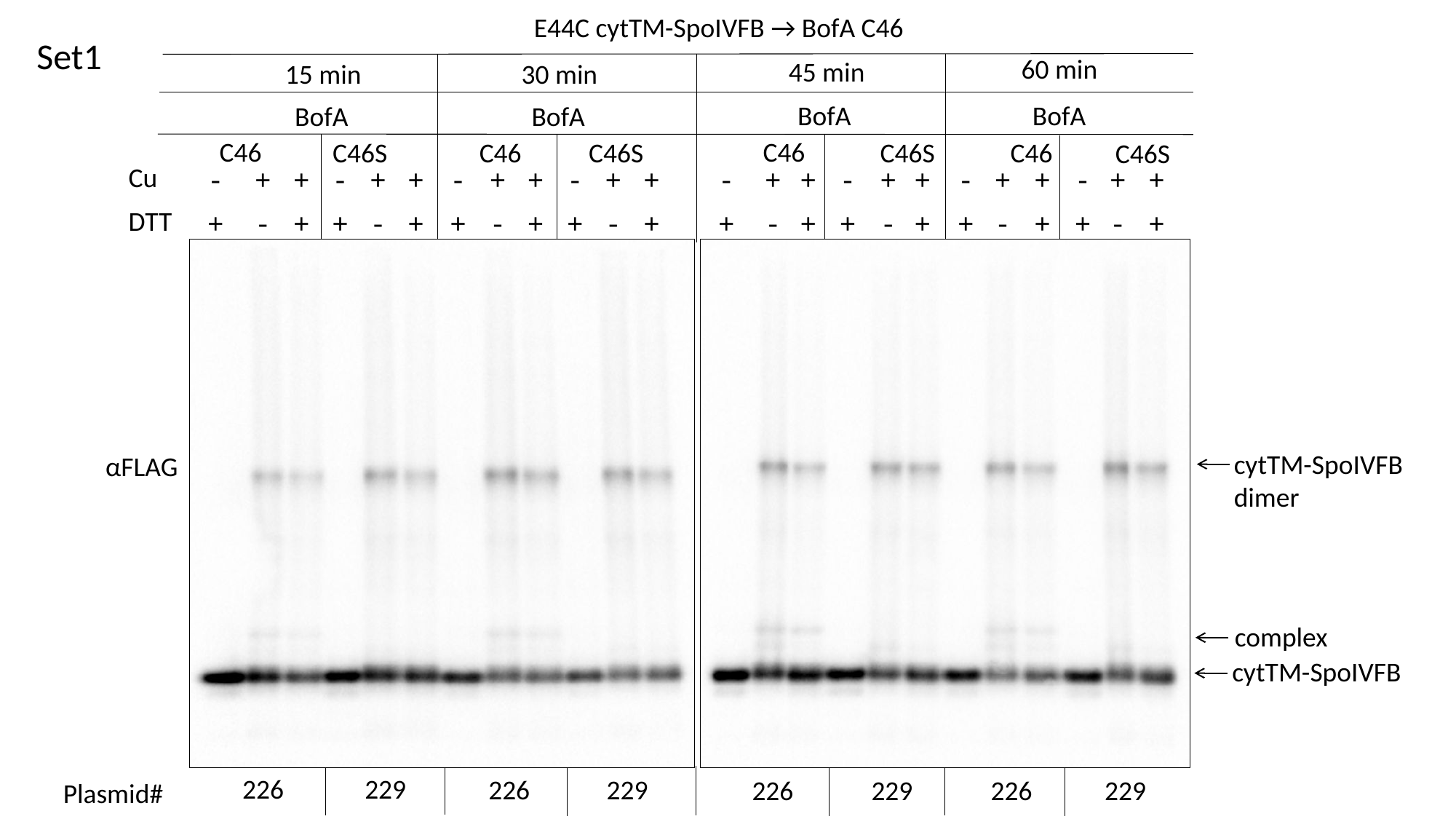

E44C cytTM-SpoIVFB → BofA C46
Set1
60 min
45 min
30 min
15 min
BofA
BofA
BofA
BofA
C46
C46
C46S
C46
C46S
C46
C46S
C46S
| Cu | - | + | + | - | + | + | - | + | + | - | + | + | - | + | + | - | + | + | - | + | + | - | + | + |
| --- | --- | --- | --- | --- | --- | --- | --- | --- | --- | --- | --- | --- | --- | --- | --- | --- | --- | --- | --- | --- | --- | --- | --- | --- |
| DTT | + | - | + | + | - | + | + | - | + | + | - | + | + | - | + | + | - | + | + | - | + | + | - | + |
cytTM-SpoIVFB
dimer
αFLAG
complex
cytTM-SpoIVFB
226
229
226
229
226
229
226
229
Plasmid#

## Slide 4
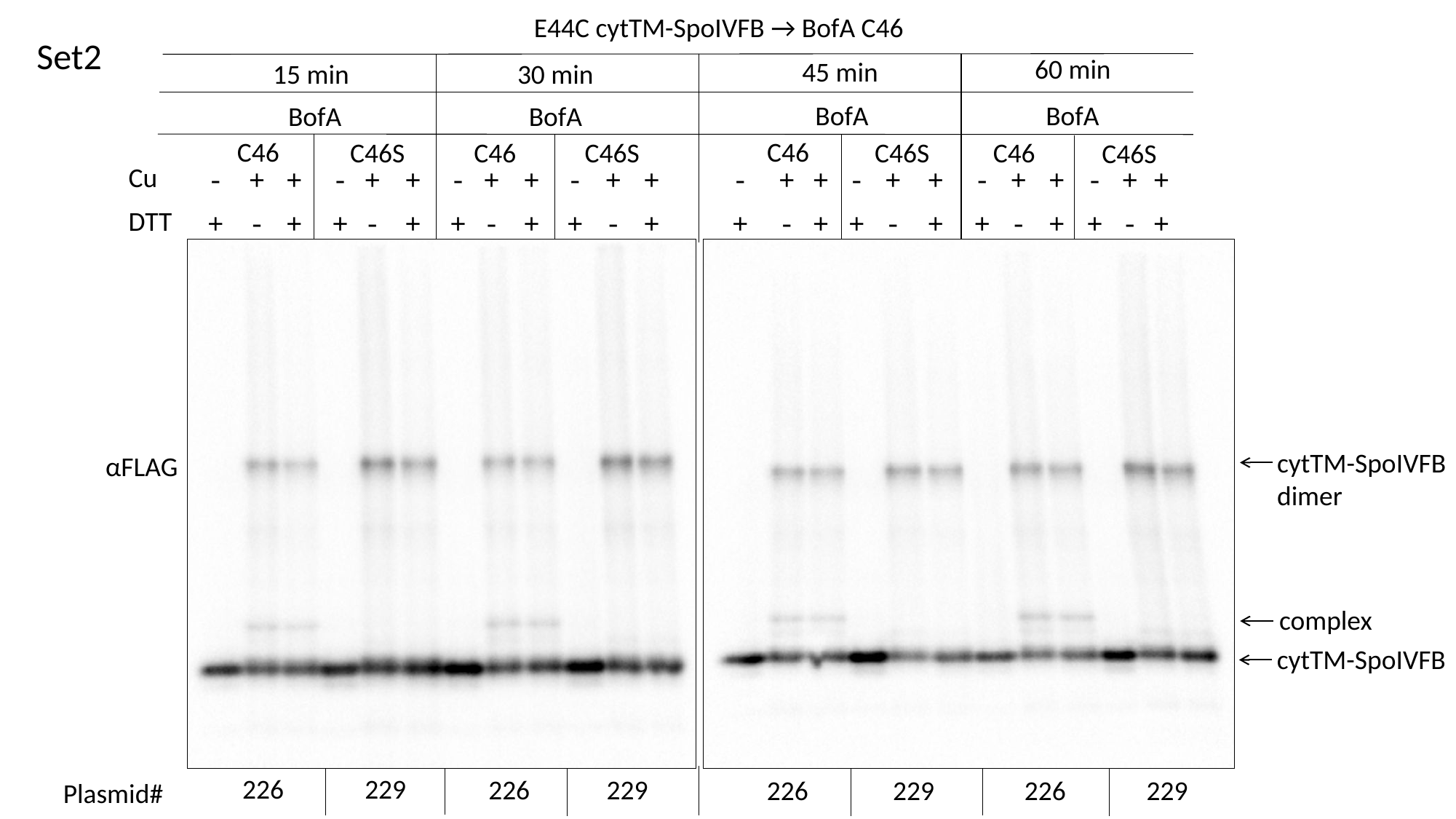

E44C cytTM-SpoIVFB → BofA C46
Set2
60 min
45 min
30 min
15 min
BofA
BofA
BofA
BofA
C46
C46
C46S
C46
C46S
C46
C46S
C46S
| Cu | - | + | + | - | + | + | - | + | + | - | + | + | - | + | + | - | + | + | - | + | + | - | + | + |
| --- | --- | --- | --- | --- | --- | --- | --- | --- | --- | --- | --- | --- | --- | --- | --- | --- | --- | --- | --- | --- | --- | --- | --- | --- |
| DTT | + | - | + | + | - | + | + | - | + | + | - | + | + | - | + | + | - | + | + | - | + | + | - | + |
cytTM-SpoIVFB
dimer
αFLAG
complex
cytTM-SpoIVFB
226
229
226
229
226
229
226
229
Plasmid#

## Slide 5
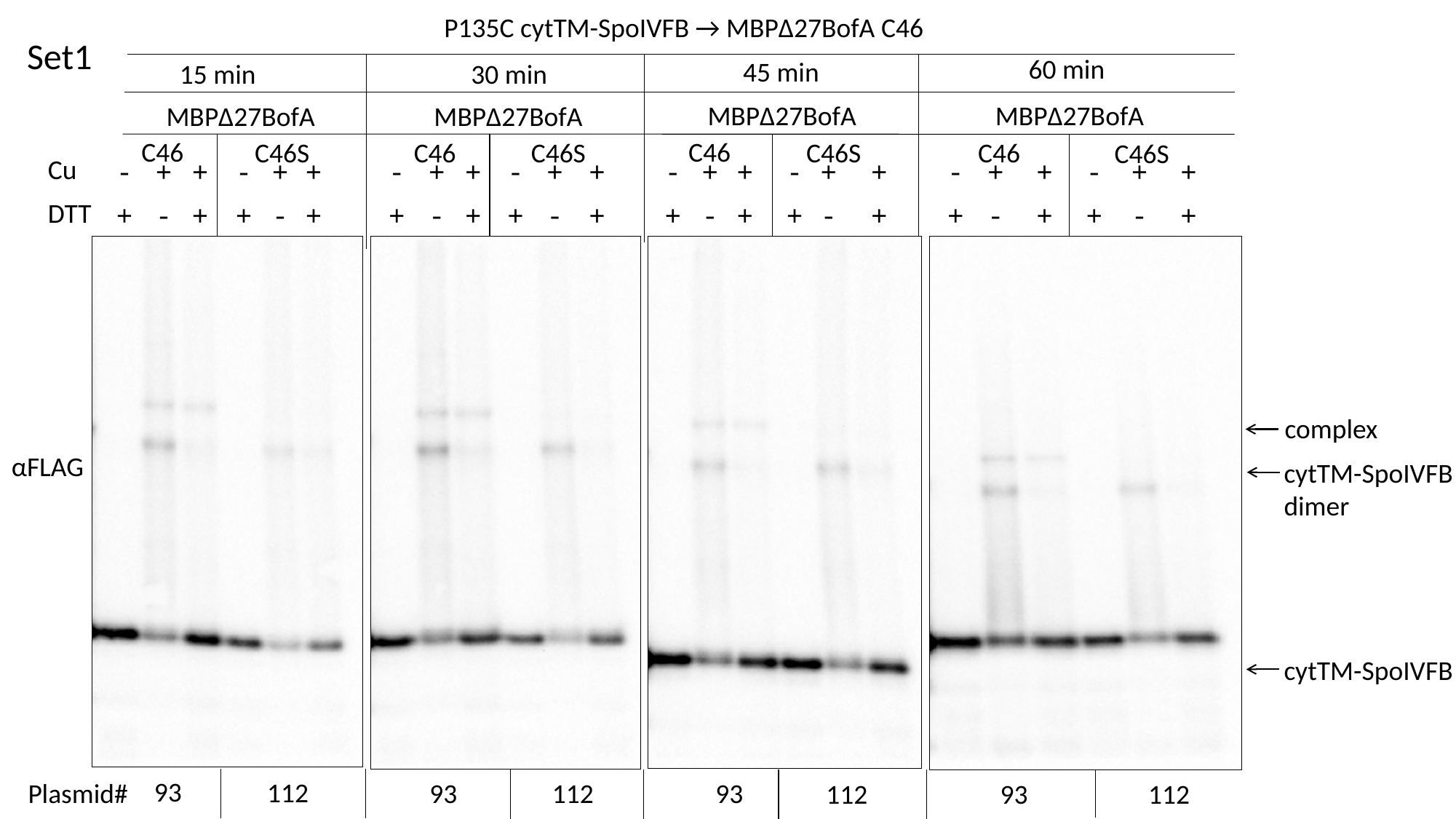

P135C cytTM-SpoIVFB → MBPΔ27BofA C46
Set1
60 min
45 min
30 min
15 min
MBPΔ27BofA
MBPΔ27BofA
MBPΔ27BofA
MBPΔ27BofA
C46
C46
C46S
C46
C46S
C46
C46S
C46S
| Cu | - | + | + | - | + | + | - | + | + | - | + | + | - | + | + | - | + | + | - | + | + | - | + | + |
| --- | --- | --- | --- | --- | --- | --- | --- | --- | --- | --- | --- | --- | --- | --- | --- | --- | --- | --- | --- | --- | --- | --- | --- | --- |
| DTT | + | - | + | + | - | + | + | - | + | + | - | + | + | - | + | + | - | + | + | - | + | + | - | + |
complex
αFLAG
cytTM-SpoIVFB
dimer
cytTM-SpoIVFB
93
112
93
Plasmid#
93
112
112
93
112

## Slide 6
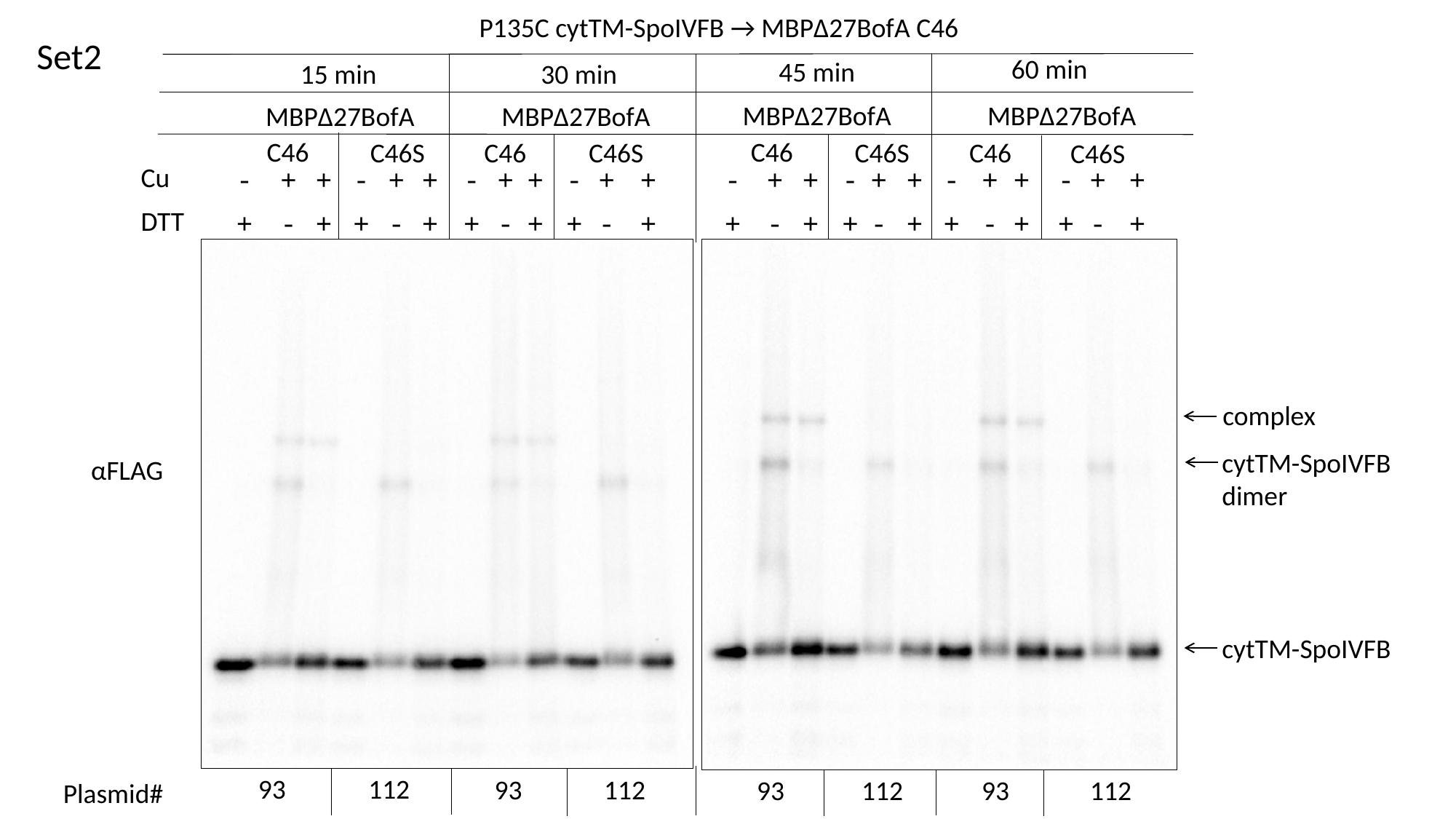

P135C cytTM-SpoIVFB → MBPΔ27BofA C46
Set2
60 min
45 min
30 min
15 min
MBPΔ27BofA
MBPΔ27BofA
MBPΔ27BofA
MBPΔ27BofA
C46
C46
C46S
C46
C46S
C46
C46S
C46S
| Cu | - | + | + | - | + | + | - | + | + | - | + | + | - | + | + | - | + | + | - | + | + | - | + | + |
| --- | --- | --- | --- | --- | --- | --- | --- | --- | --- | --- | --- | --- | --- | --- | --- | --- | --- | --- | --- | --- | --- | --- | --- | --- |
| DTT | + | - | + | + | - | + | + | - | + | + | - | + | + | - | + | + | - | + | + | - | + | + | - | + |
complex
cytTM-SpoIVFB
dimer
αFLAG
cytTM-SpoIVFB
93
112
93
112
93
112
93
112
Plasmid#

## Slide 7
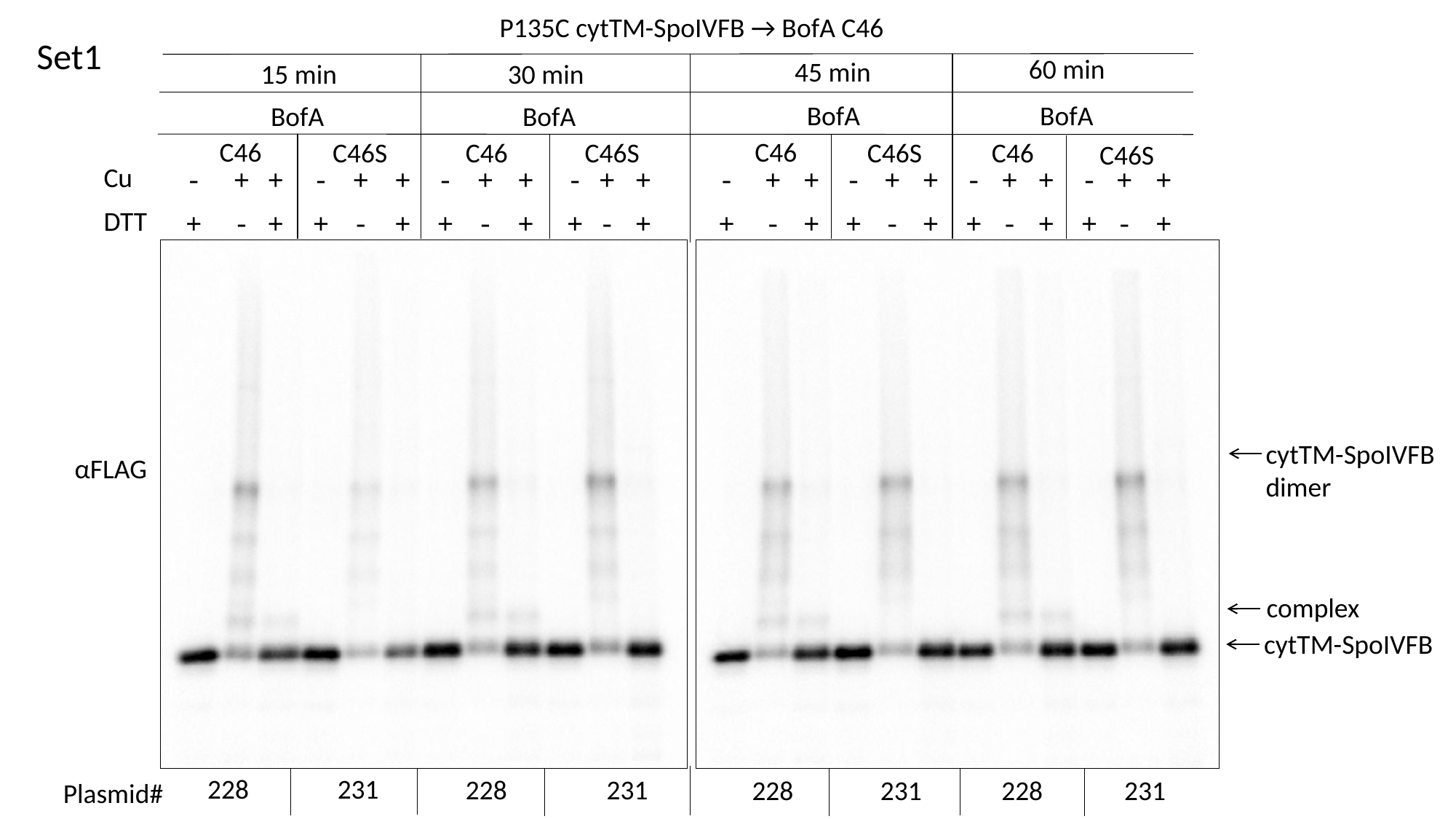

P135C cytTM-SpoIVFB → BofA C46
Set1
60 min
45 min
30 min
15 min
BofA
BofA
BofA
BofA
C46
C46
C46S
C46
C46S
C46
C46S
C46S
| Cu | - | + | + | - | + | + | - | + | + | - | + | + | - | + | + | - | + | + | - | + | + | - | + | + |
| --- | --- | --- | --- | --- | --- | --- | --- | --- | --- | --- | --- | --- | --- | --- | --- | --- | --- | --- | --- | --- | --- | --- | --- | --- |
| DTT | + | - | + | + | - | + | + | - | + | + | - | + | + | - | + | + | - | + | + | - | + | + | - | + |
cytTM-SpoIVFB
dimer
αFLAG
complex
cytTM-SpoIVFB
228
231
228
231
228
231
228
231
Plasmid#

## Slide 8
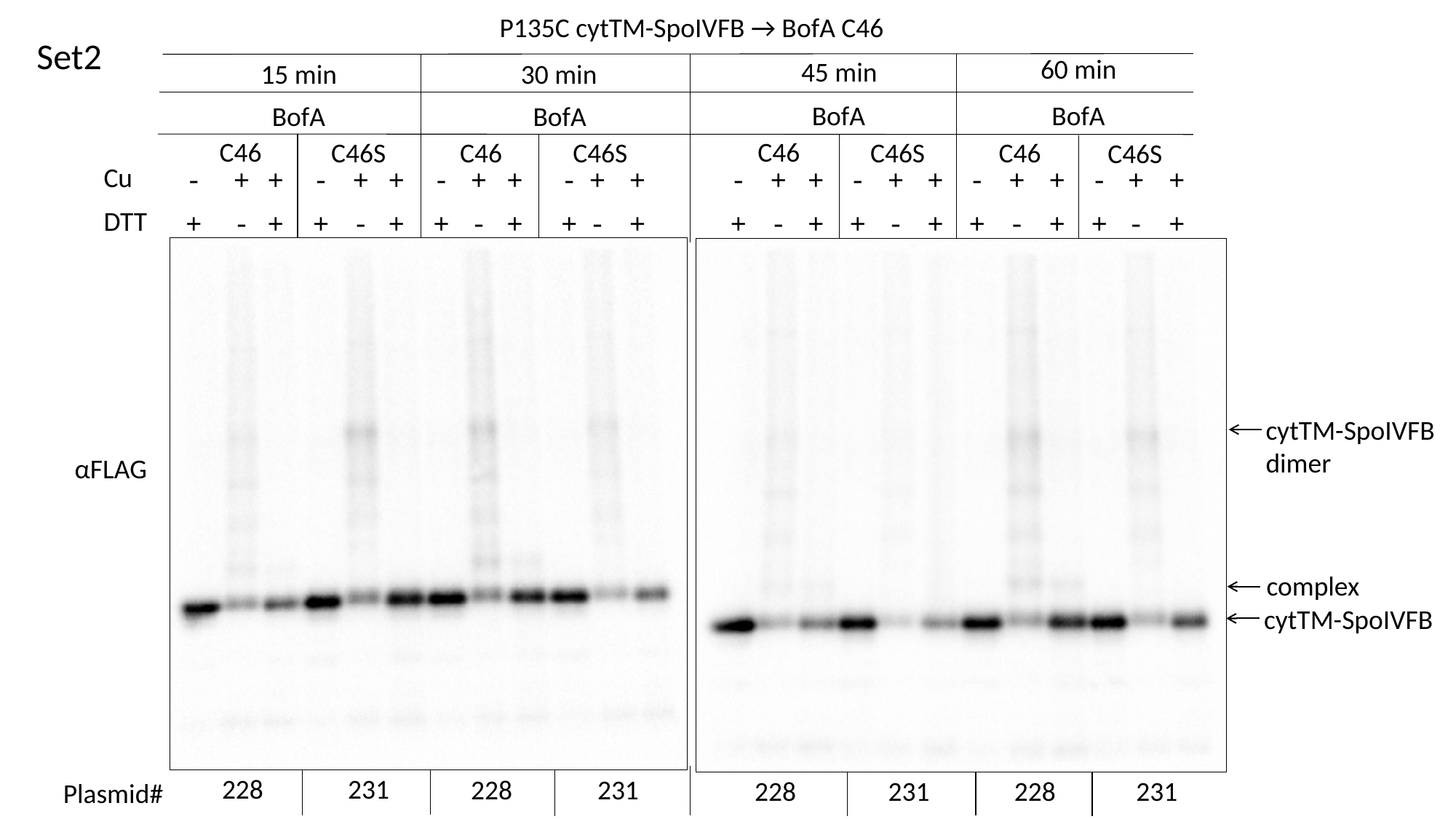

P135C cytTM-SpoIVFB → BofA C46
Set2
60 min
45 min
30 min
15 min
BofA
BofA
BofA
BofA
C46
C46
C46S
C46
C46S
C46
C46S
C46S
| Cu | - | + | + | - | + | + | - | + | + | - | + | + | - | + | + | - | + | + | - | + | + | - | + | + |
| --- | --- | --- | --- | --- | --- | --- | --- | --- | --- | --- | --- | --- | --- | --- | --- | --- | --- | --- | --- | --- | --- | --- | --- | --- |
| DTT | + | - | + | + | - | + | + | - | + | + | - | + | + | - | + | + | - | + | + | - | + | + | - | + |
cytTM-SpoIVFB
dimer
αFLAG
complex
cytTM-SpoIVFB
228
231
228
231
228
231
228
231
Plasmid#
