# Supplementary material for: Inhibitory proteins block substrate access by occupying the active site cleft of Bacillus subtilis intramembrane protease SpoIVFB
Source: eLife. 2022 Apr 26;11:e74275. doi: 10.7554/eLife.74275 (PMC9042235; doi:10.7554/eLife.74275)
Supplement: Figure 6—figure supplement 5—source data 1. [file elife-74275-fig6-figsupp5-data1.zip › Figure 6-figure supplement 5-source data 1/figure supplement 5A/fig sup 5A annotated blot.pptx]

## Slide 1
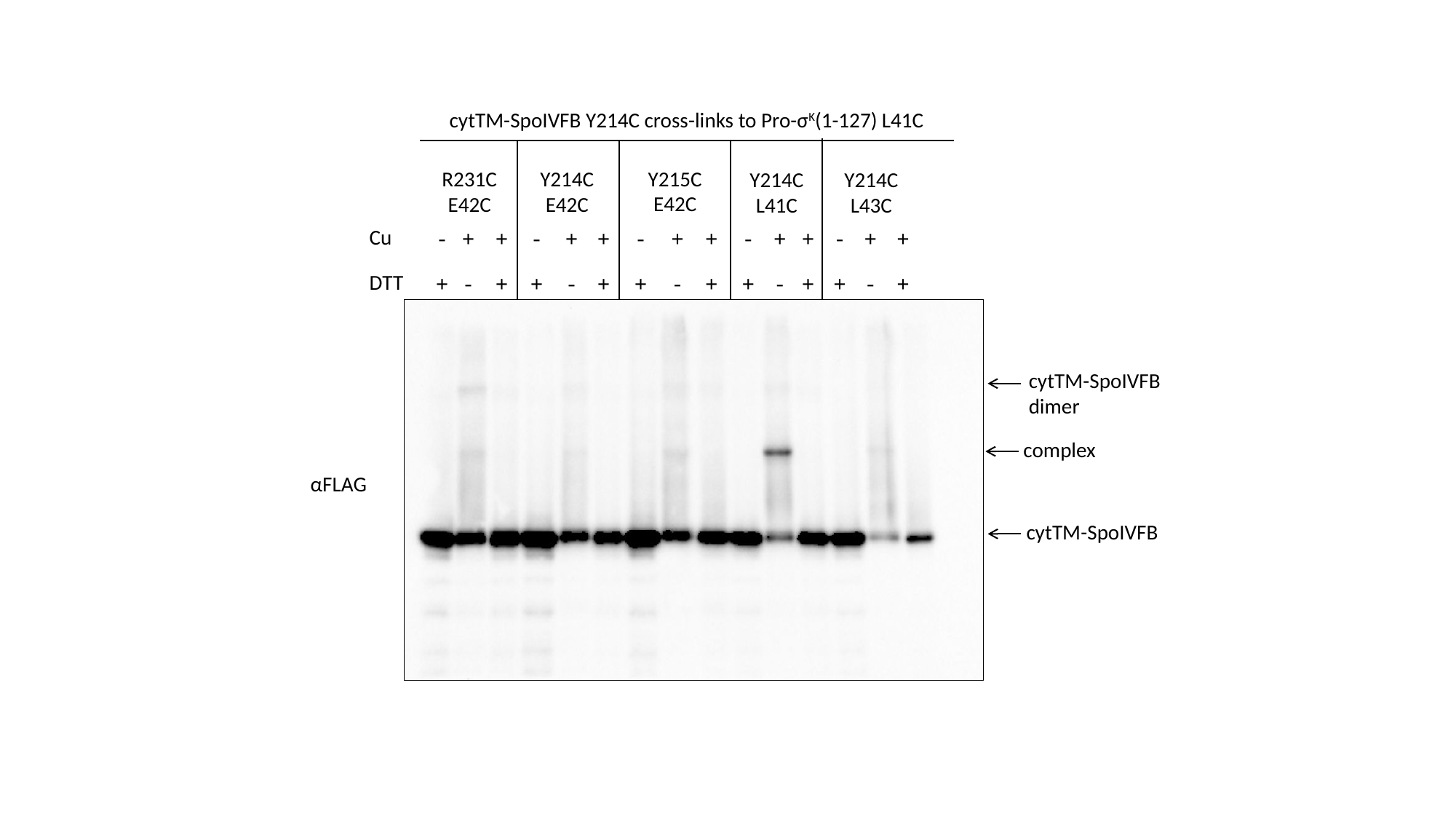

cytTM-SpoIVFB Y214C cross-links to Pro-σK(1-127) L41C
Y215C
E42C
R231C
E42C
Y214C
E42C
Y214C
L41C
Y214C
L43C
| Cu | - | + | + | - | + | + | - | + | + | - | + | + | - | + | + | | | |
| --- | --- | --- | --- | --- | --- | --- | --- | --- | --- | --- | --- | --- | --- | --- | --- | --- | --- | --- |
| DTT | + | - | + | + | - | + | + | - | + | + | - | + | + | - | + | | | |
cytTM-SpoIVFB
dimer
complex
αFLAG
 cytTM-SpoIVFB
