# Supplementary material for: Inhibitory proteins block substrate access by occupying the active site cleft of Bacillus subtilis intramembrane protease SpoIVFB
Source: eLife. 2022 Apr 26;11:e74275. doi: 10.7554/eLife.74275 (PMC9042235; doi:10.7554/eLife.74275)
Supplement: Figure 6—figure supplement 5—source data 1. [file elife-74275-fig6-figsupp5-data1.zip › Figure 6-figure supplement 5-source data 1/figure supplement 5B/fig sup 5B annotated blots.pptx]

## Slide 1
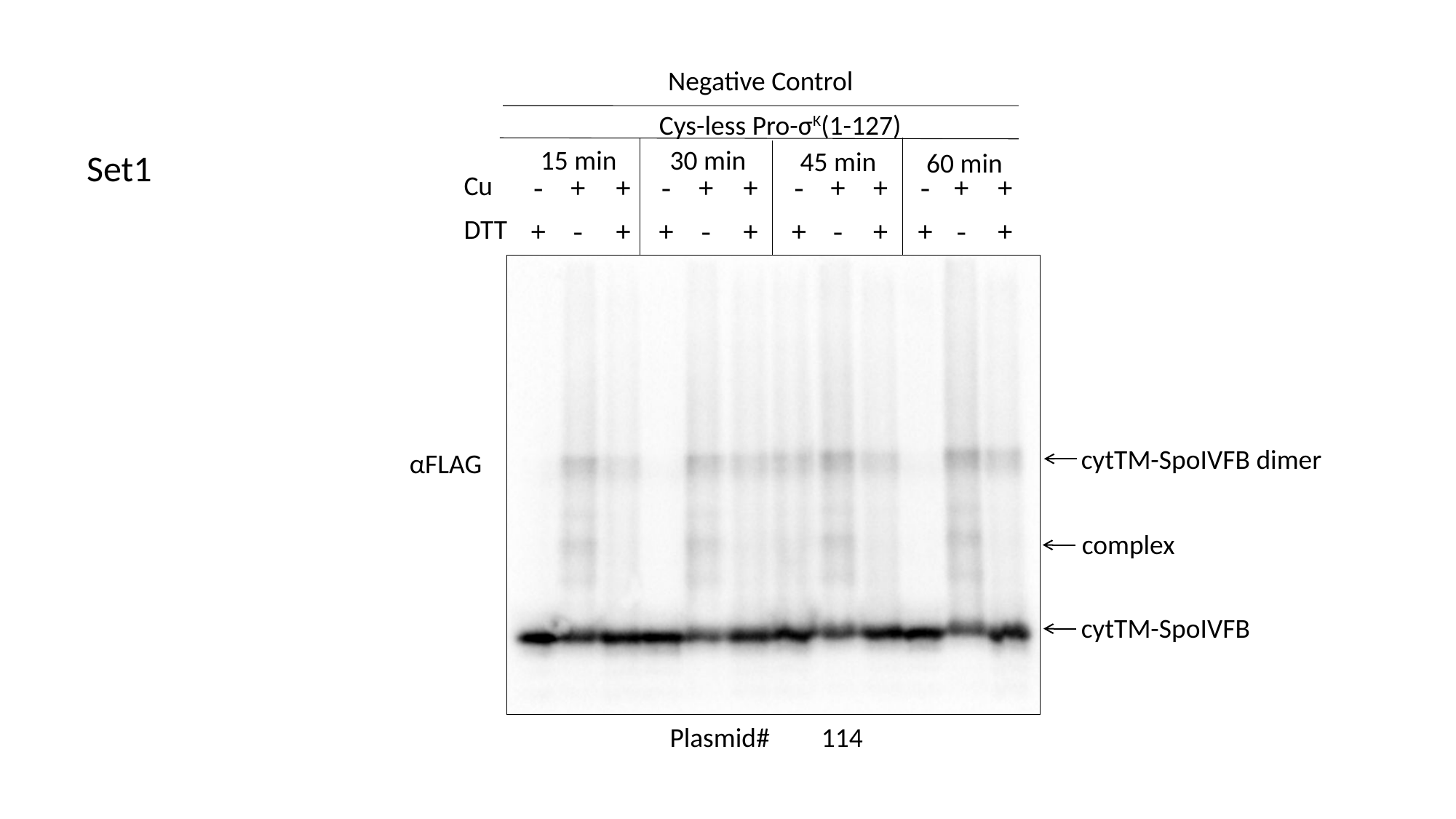

Negative Control
Cys-less Pro-σK(1-127)
15 min
30 min
45 min
Set1
60 min
| Cu | - | + | + | - | + | + | - | + | + | - | + | + | | | |
| --- | --- | --- | --- | --- | --- | --- | --- | --- | --- | --- | --- | --- | --- | --- | --- |
| DTT | + | - | + | + | - | + | + | - | + | + | - | + | | | |
cytTM-SpoIVFB dimer
αFLAG
complex
cytTM-SpoIVFB
Plasmid#
114

## Slide 2
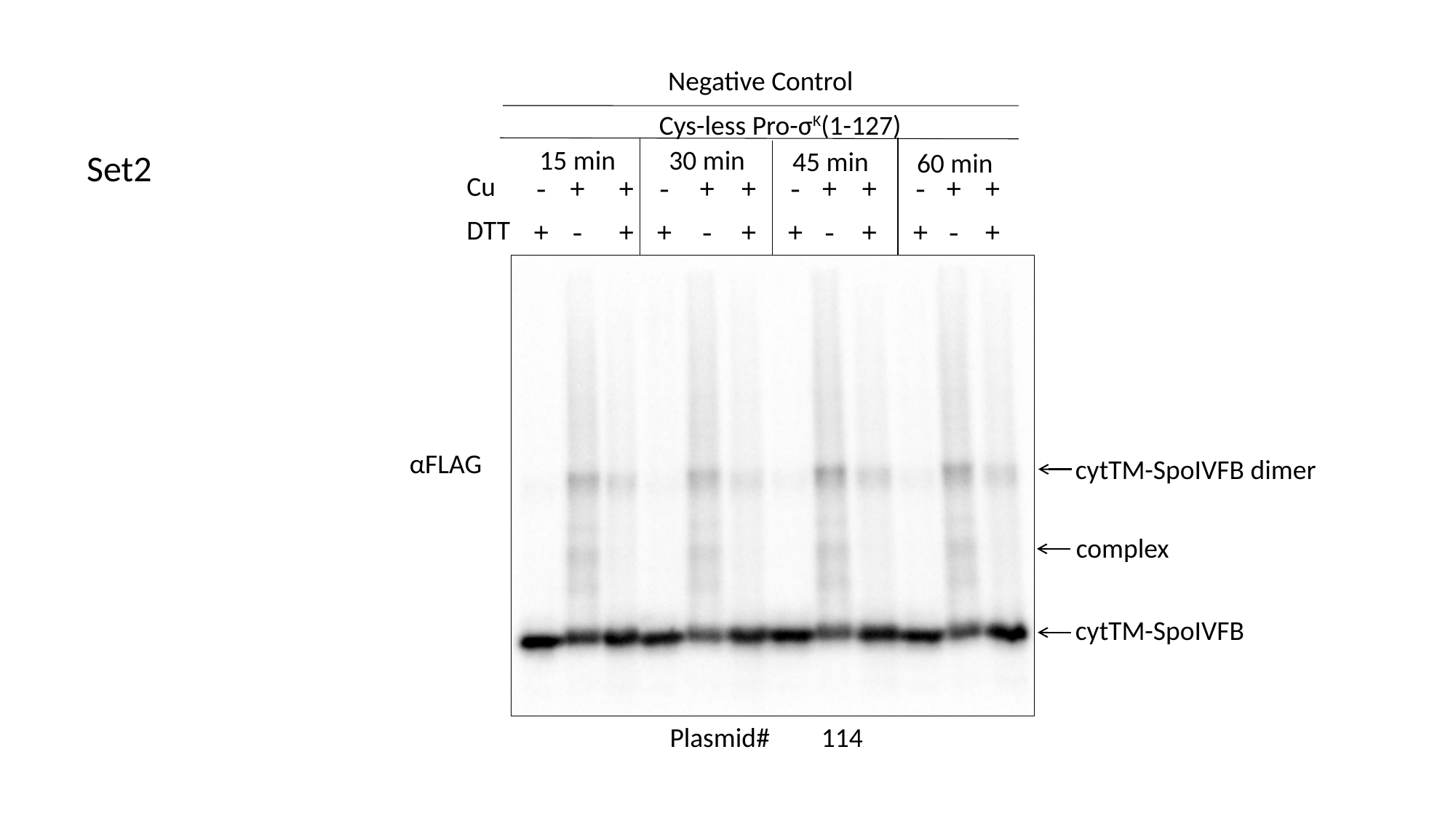

Negative Control
Cys-less Pro-σK(1-127)
15 min
30 min
45 min
Set2
60 min
| Cu | - | + | + | - | + | + | - | + | + | - | + | + | | | |
| --- | --- | --- | --- | --- | --- | --- | --- | --- | --- | --- | --- | --- | --- | --- | --- |
| DTT | + | - | + | + | - | + | + | - | + | + | - | + | | | |
αFLAG
cytTM-SpoIVFB dimer
complex
cytTM-SpoIVFB
Plasmid#
114

## Slide 3
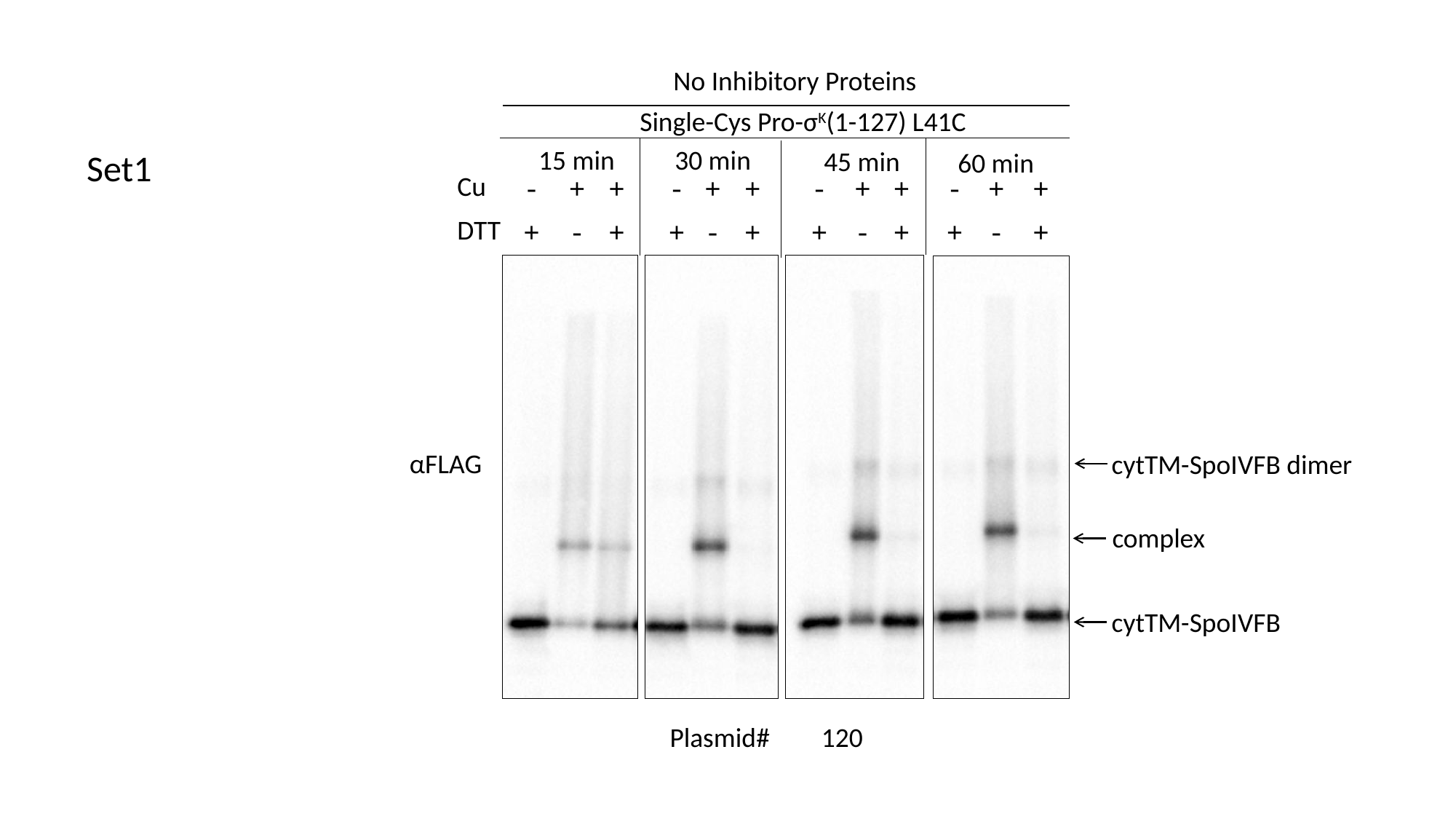

No Inhibitory Proteins
Single-Cys Pro-σK(1-127) L41C
15 min
30 min
45 min
Set1
60 min
| Cu | - | + | + | - | + | + | - | + | + | - | + | + | | | |
| --- | --- | --- | --- | --- | --- | --- | --- | --- | --- | --- | --- | --- | --- | --- | --- |
| DTT | + | - | + | + | - | + | + | - | + | + | - | + | | | |
αFLAG
cytTM-SpoIVFB dimer
complex
cytTM-SpoIVFB
Plasmid#
120

## Slide 4
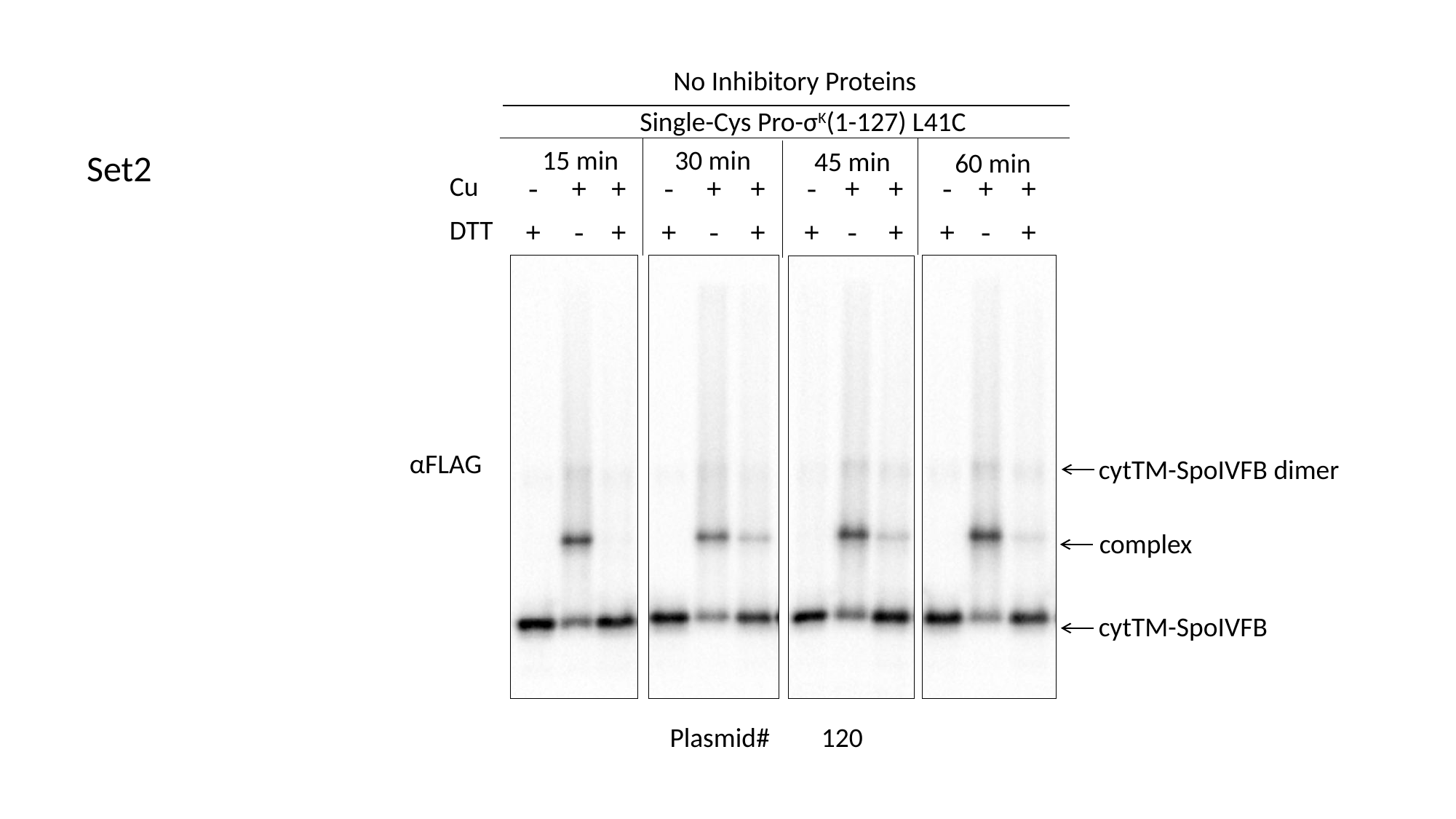

No Inhibitory Proteins
Single-Cys Pro-σK(1-127) L41C
15 min
30 min
45 min
Set2
60 min
| Cu | - | + | + | - | + | + | - | + | + | - | + | + | | | |
| --- | --- | --- | --- | --- | --- | --- | --- | --- | --- | --- | --- | --- | --- | --- | --- |
| DTT | + | - | + | + | - | + | + | - | + | + | - | + | | | |
αFLAG
cytTM-SpoIVFB dimer
complex
cytTM-SpoIVFB
Plasmid#
120

## Slide 5
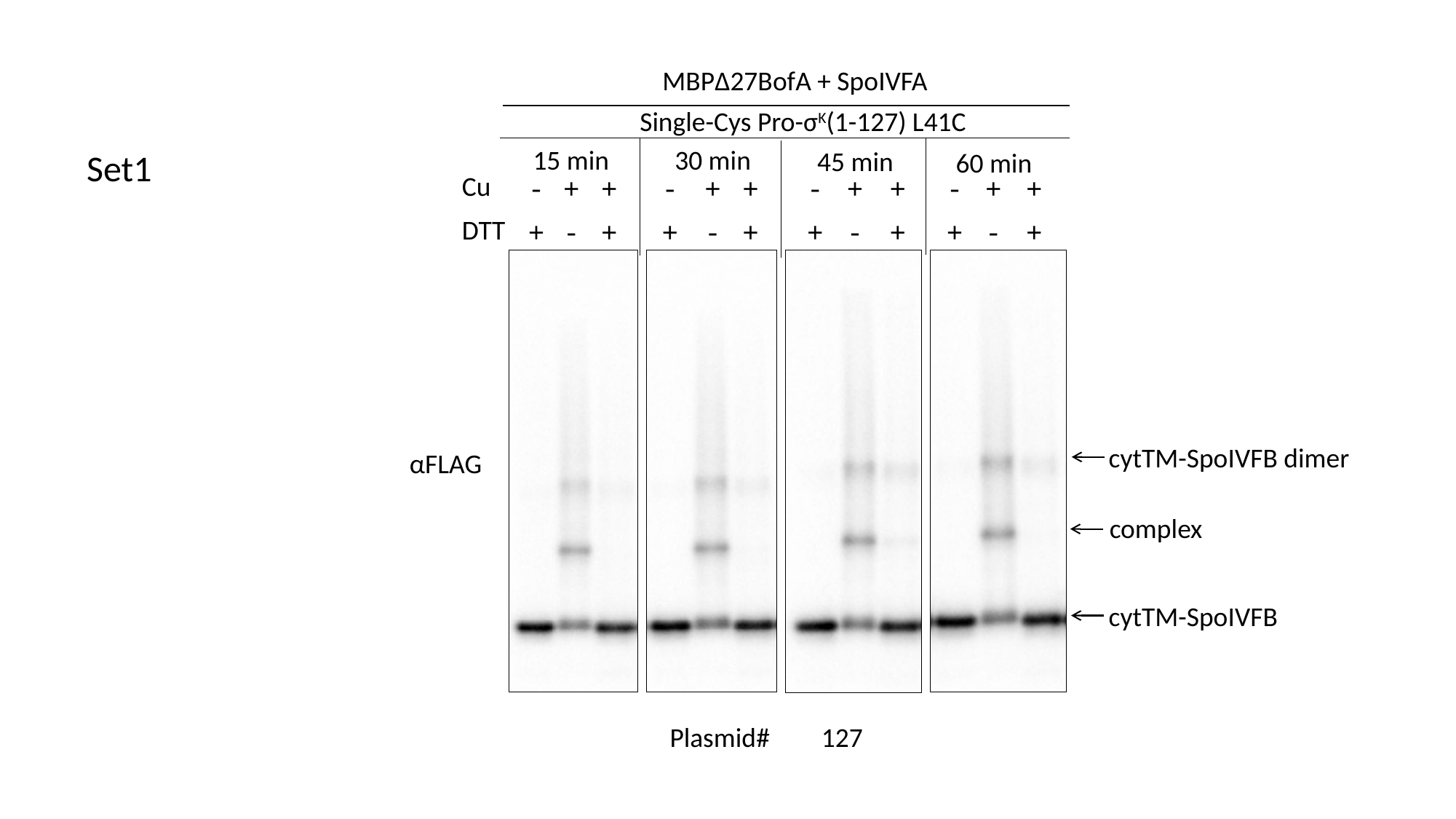

MBPΔ27BofA + SpoIVFA
Single-Cys Pro-σK(1-127) L41C
15 min
30 min
45 min
Set1
60 min
| Cu | - | + | + | - | + | + | - | + | + | - | + | + | | | |
| --- | --- | --- | --- | --- | --- | --- | --- | --- | --- | --- | --- | --- | --- | --- | --- |
| DTT | + | - | + | + | - | + | + | - | + | + | - | + | | | |
cytTM-SpoIVFB dimer
αFLAG
complex
cytTM-SpoIVFB
Plasmid#
127

## Slide 6
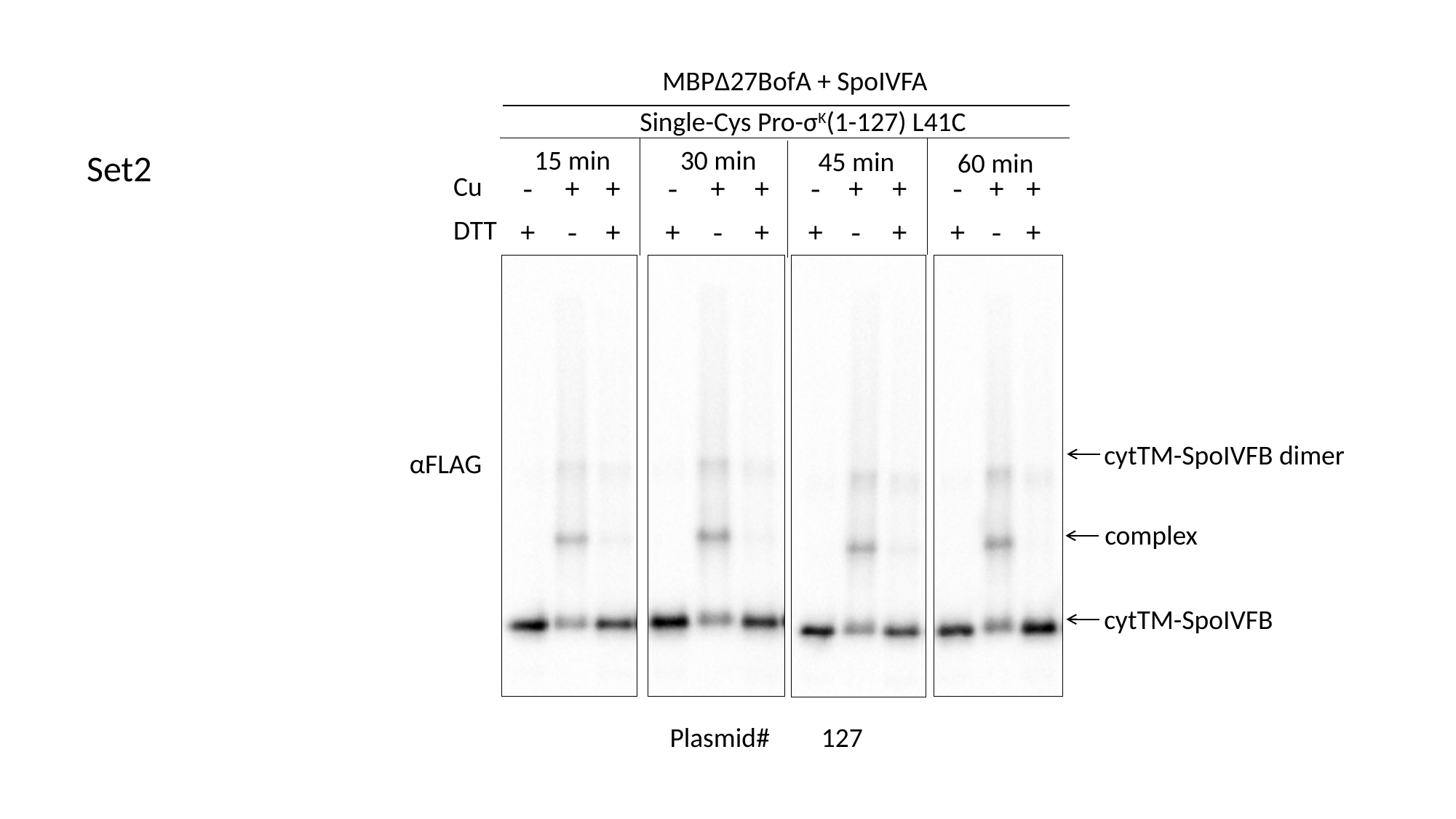

MBPΔ27BofA + SpoIVFA
Single-Cys Pro-σK(1-127) L41C
15 min
30 min
45 min
Set2
60 min
| Cu | - | + | + | - | + | + | - | + | + | - | + | + | | | |
| --- | --- | --- | --- | --- | --- | --- | --- | --- | --- | --- | --- | --- | --- | --- | --- |
| DTT | + | - | + | + | - | + | + | - | + | + | - | + | | | |
cytTM-SpoIVFB dimer
αFLAG
complex
cytTM-SpoIVFB
Plasmid#
127

## Slide 7
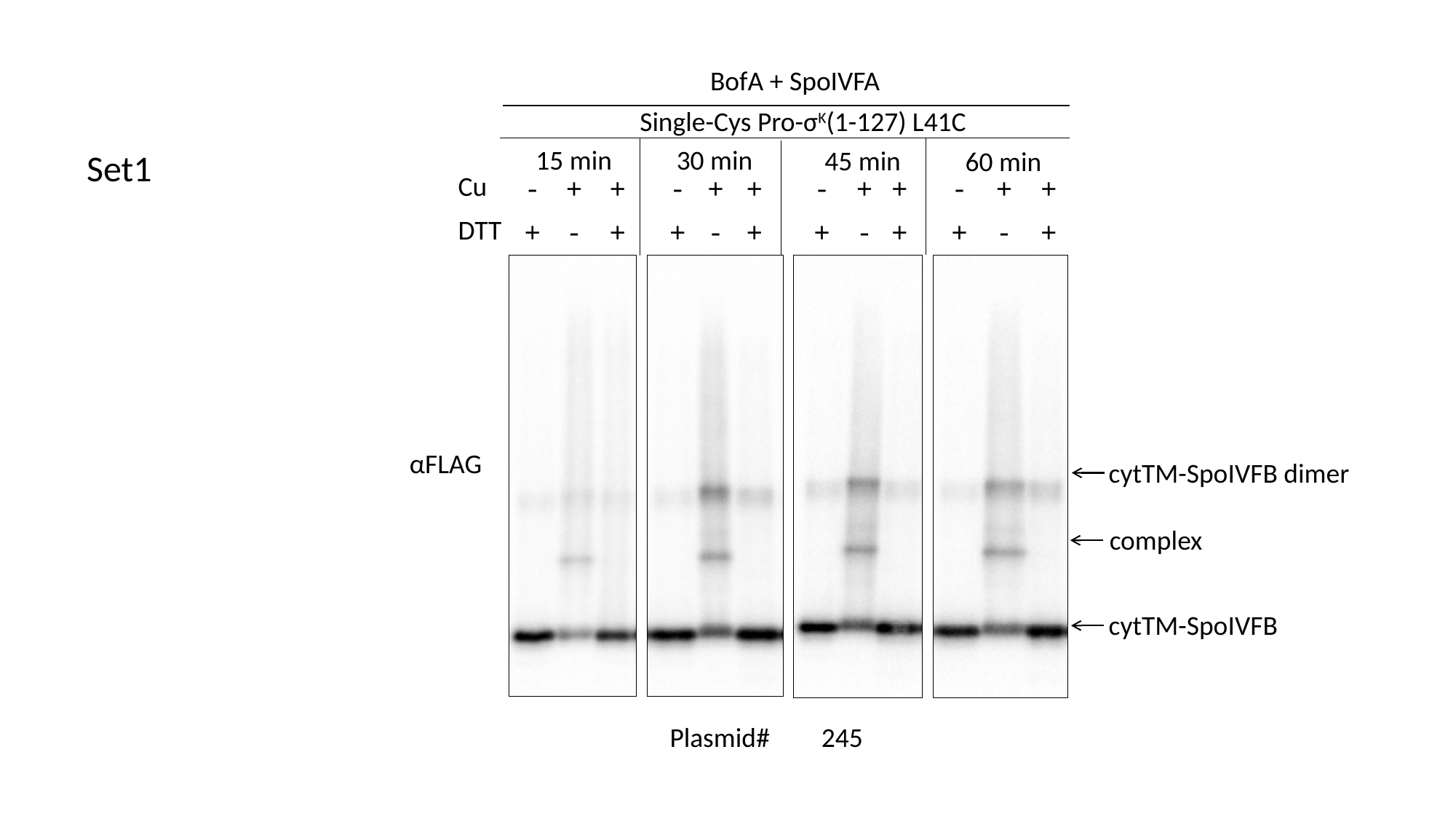

BofA + SpoIVFA
Single-Cys Pro-σK(1-127) L41C
15 min
30 min
45 min
60 min
Set1
| Cu | - | + | + | - | + | + | - | + | + | - | + | + | | | |
| --- | --- | --- | --- | --- | --- | --- | --- | --- | --- | --- | --- | --- | --- | --- | --- |
| DTT | + | - | + | + | - | + | + | - | + | + | - | + | | | |
αFLAG
cytTM-SpoIVFB dimer
complex
cytTM-SpoIVFB
Plasmid#
245

## Slide 8
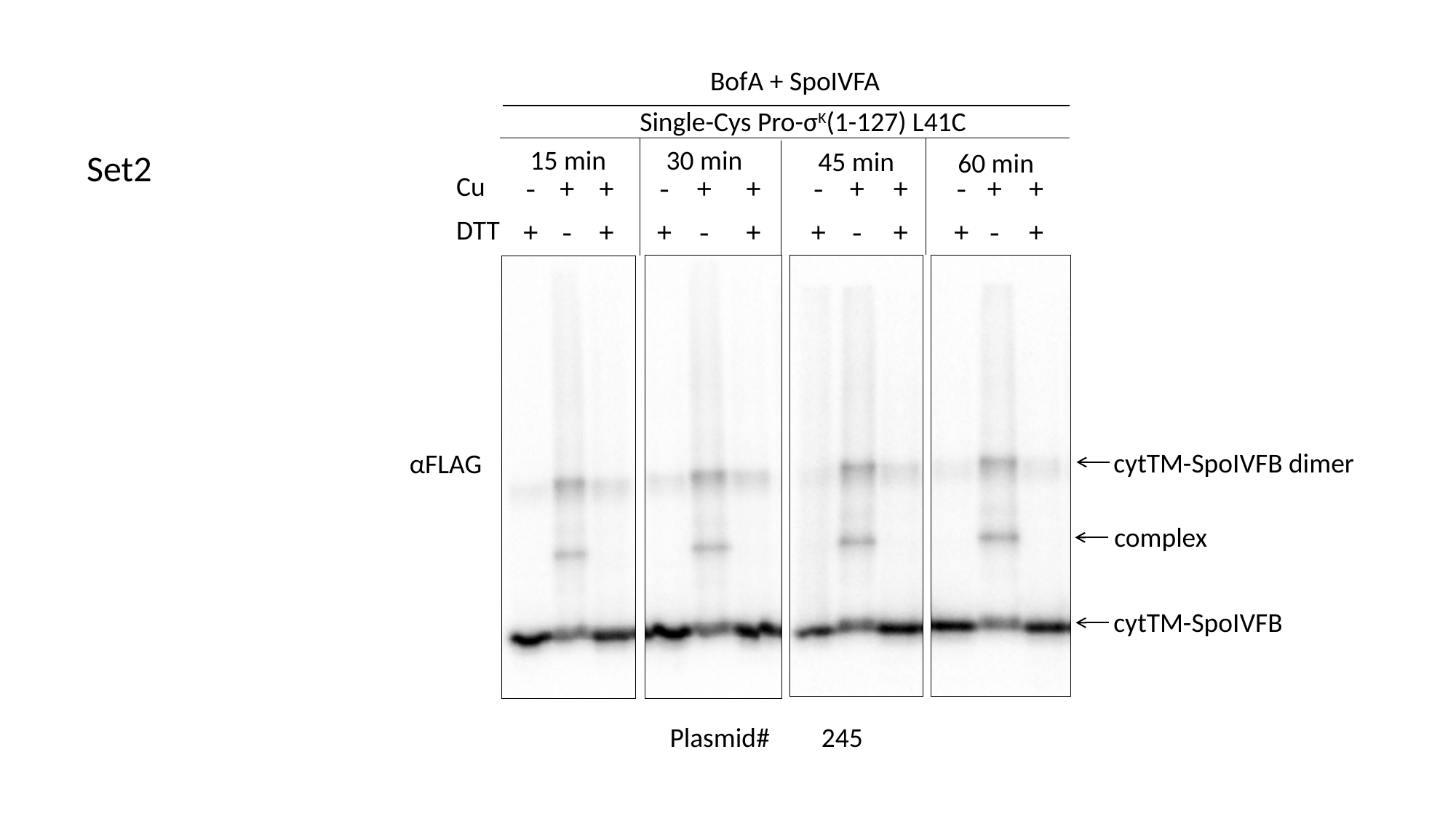

BofA + SpoIVFA
Single-Cys Pro-σK(1-127) L41C
15 min
30 min
45 min
Set2
60 min
| Cu | - | + | + | - | + | + | - | + | + | - | + | + | | | |
| --- | --- | --- | --- | --- | --- | --- | --- | --- | --- | --- | --- | --- | --- | --- | --- |
| DTT | + | - | + | + | - | + | + | - | + | + | - | + | | | |
cytTM-SpoIVFB dimer
αFLAG
complex
cytTM-SpoIVFB
Plasmid#
245
