# Supplementary material for: Inhibitory proteins block substrate access by occupying the active site cleft of Bacillus subtilis intramembrane protease SpoIVFB
Source: eLife. 2022 Apr 26;11:e74275. doi: 10.7554/eLife.74275 (PMC9042235; doi:10.7554/eLife.74275)
Supplement: Figure 7—source data 1. [file elife-74275-fig7-data1.zip › Figure 7-source data 1/readme.docx]

The PyMOL session file (Figure 7C) was derived from the model ‘fb.sigk.bofa.fa’ of a SpoIVFB tetramer in complex with one molecule each of full-length BofA and parts of Pro-σ^K^ (residues 38-114) and SpoIVFA (residues 65-111), by hiding the B, C, and D chains of SpoIVFB. The images shown in Figure 7 A and B were then derived by hiding additional parts of the model.
