# Supplementary material for: Inhibitory proteins block substrate access by occupying the active site cleft of Bacillus subtilis intramembrane protease SpoIVFB
Source: eLife. 2022 Apr 26;11:e74275. doi: 10.7554/eLife.74275 (PMC9042235; doi:10.7554/eLife.74275)
Supplement: Figure 7—figure supplement 1—source data 1. [file elife-74275-fig7-figsupp1-data1.zip › Figure 7-figure supplement 1-source data 1/readme.docx]

The PyMOL session file (fig sup 1) was derived from the model ‘fb.sigk’ of a SpoIVFB tetramer in complex with part of one molecule of Pro-σ^K^ (residues 1-114). The side view was rotated and the B, C, and D chains of SpoIVFB were hidden to produce the other images.
