# Supplementary material for: Inhibitory proteins block substrate access by occupying the active site cleft of Bacillus subtilis intramembrane protease SpoIVFB
Source: eLife. 2022 Apr 26;11:e74275. doi: 10.7554/eLife.74275 (PMC9042235; doi:10.7554/eLife.74275)
Supplement: Figure 7—figure supplement 2—source data 1. [file elife-74275-fig7-figsupp2-data1.zip › Figure 7-figure supplement 2-source data 1/readme.docx]

The PyMOL session file (fig sup 2) was derived from the Figure 7C PyMOL session file in the Figure 7-source data 1 folder by hiding all chains except full-length BofA and coloring the N48, N61, and T64 side chains blue. The side view was then enlarged, cropped, and rotated to produce the other images.
